# Evolutionary dynamics and structural consequences of de novo beneficial mutations and mutant lineages arising in a constant environment

**DOI:** 10.1186/s12915-021-00954-0

**Published:** 2021-02-04

**Authors:** Margie Kinnersley, Katja Schwartz, Dong-Dong Yang, Gavin Sherlock, Frank Rosenzweig

**Affiliations:** 1 Division of Biological Sciences, The University of Montana, Missoula, MT 59812 USA; 2 Department of Genetics, Stanford University School of Medicine, Stanford, CA 94305-5120 USA; 3 School of Biological Sciences, Georgia Institute of Technology, Atlanta, GA 30332 USA

**Keywords:** *E. coli*, Adaptation, Experimental evolution, Clonal interference, Parallelism, Resource limitation

## Abstract

**Background:**

Microbial evolution experiments can be used to study the tempo and dynamics of evolutionary change in asexual populations, founded from single clones and growing into large populations with multiple clonal lineages. High-throughput sequencing can be used to catalog de novo mutations as potential targets of selection, determine in which lineages they arise, and track the fates of those lineages. Here, we describe a long-term experimental evolution study to identify targets of selection and to determine when, where, and how often those targets are hit.

**Results:**

We experimentally evolved replicate *Escherichia coli* populations that originated from a mutator/nonsense suppressor ancestor under glucose limitation for between 300 and 500 generations. Whole-genome, whole-population sequencing enabled us to catalog 3346 de novo mutations that reached > 1% frequency. We sequenced the genomes of 96 clones from each population when allelic diversity was greatest in order to establish whether mutations were in the same or different lineages and to depict lineage dynamics. Operon-specific mutations that enhance glucose uptake were the first to rise to high frequency, followed by global regulatory mutations. Mutations related to energy conservation, membrane biogenesis, and mitigating the impact of nonsense mutations, both ancestral and derived, arose later. New alleles were confined to relatively few loci, with many instances of identical mutations arising independently in multiple lineages, among and within replicate populations. However, most never exceeded 10% in frequency and were at a lower frequency at the end of the experiment than at their maxima, indicating clonal interference. Many alleles mapped to key structures within the proteins that they mutated, providing insight into their functional consequences.

**Conclusions:**

Overall, we find that when mutational input is increased by an ancestral defect in DNA repair, the spectrum of high-frequency beneficial mutations in a simple, constant resource-limited environment is narrow, resulting in extreme parallelism where many adaptive mutations arise but few ever go to fixation.

**Supplementary Information:**

The online version contains supplementary material available at 10.1186/s12915-021-00954-0.

## Author summary

Microbial evolution experiments open a window on the tempo and dynamics of evolutionary change in asexual populations. High-throughput sequencing can be used to catalog de novo mutations, determine in which lineages they arise, and assess allelic interactions by tracking the fate of those lineages. This approach, adaptive genetics, makes it possible to discover whether clonal interactions are antagonistic or synergistic, and complements genetic screens of induced deleterious/loss-of-function mutants. Using glucose-limited chemostats, we carried out 300–500 generation evolution experiments founded by an *Escherichia coli* mutator/nonsense suppressor strain. Whole-genome, whole-population sequencing enabled us to catalog 3346 de novo mutations that reached > 1% frequency. Mutations enhancing glucose uptake rose to high frequency first, followed by global regulatory changes that modulate growth rate and assimilation of the limiting resource; later selected mutations favored energy conservation and/or mitigated pleiotropic effects of earlier regulatory changes. Several loci were highly polymorphic, with identical mutations arising independently in different lineages, both between and within replicate populations. When mutational input is increased by an ancestral defect in DNA repair but mitigated by a nonsense suppressor, the number of beneficial mutants attributable to loss-of-function involves fewer nonsense mutations relative to missense mutations. Under nutrient limitation, selection is called upon to explore sequence space for changes in protein structure that favor, for example, de-repression of genes and pathways needed to acquire the limiting nutrient. The net result of this process is extreme parallelism, where many adaptive mutations arise within a relatively small set of genes, but few of those mutations ever become fixed. The distribution of such alleles in sequence space is useful for adaptive genetics-driven studies of protein structure-function relationships.

## Background

Experimental microbial evolution has enlarged our understanding of the tempo and mode of adaptive change in asexual populations, as well as how selection, drift, and historical contingency influence their evolutionary trajectories. Using high-throughput sequencing, we can now identify substantial numbers of de novo beneficial mutations in laboratory populations, determine in which lineages they arise and the fate of those lineages, and evaluate how different alleles interact [1–3]. This approach, adaptive genetics, based on analyzing cohorts of spontaneous beneficial mutations to determine how their frequencies fluctuate over time, complements traditional genetic screening of induced deleterious/loss-of-function mutants (e.g., [4, 5]). Adaptive genetics also opens up new ways to discover constraints on protein structure and function and to discern the architecture and malleability of networks that regulate nutrient sensing and uptake and that coordinate cell division.

Microbial populations were once thought to evolve by the periodic selection of adaptive clones, each fitter than its antecedent, replacing one another over successive generations [6–9]. This model is consistent with Muller and Haldane’s view of how beneficial mutations spread in large asexual populations [10–12] governed by competitive exclusion [13]; indeed, periodic selection has been observed in nosocomial outbreaks [14] and epidemics [15], as well as in breast cancer [16] and tumor-specific T cells [17]. But clonal populations can also accumulate and retain genetic variation, much of which is beneficial [18–22]. In fact, whole-genome, whole-population sequencing has shown that even under simple laboratory conditions the amount of adaptive genetic variation arising in microbial populations can be enormous, owing to their large size and to the continuous input of neutral and adaptive mutations [20, 23].

When novel beneficial mutations arise in independent lineages and these lineages have similar fitness, a ‘Battle Royale’ ensues, producing clonal interference [18, 20, 24–26]. Clonal interference can also occur within a broader framework of stable subpopulation structure [27], especially if lineages come under balancing selection [28–31] or specialize to exploit niches created by the culture conditions [29, 32, 33] or by the organisms themselves [34–36]. Theory indicates that in a resource-limited environment the likelihood that subpopulations co-exist depends on the input of the primary resource, the output of secondary resources, and the relative fitness of clones that can profit from secondary resources [37]. The ancestral genotype may also be decisive. Glucose-limited evolution experiments carried out by Ferenci et al. using one ancestral *E. coli* K12 derivative never produced stable subpopulations [38], whereas those carried out by Adams et al. using another often did [36, 39]. Adams’ strain was later shown to carry nonsense mutations in mismatch repair enzyme MutY, housekeeping and stationary-phase transcription factors RpoD and RpoS, as well as a tRNA nonsense suppressor. While an ancestral defect in DNA repair would increase the descendant population’s mutational load [34, 40], a nonsense suppressor in that ancestor would likely mitigate the effect of any mutation that caused a premature STOP codon. This genotype could be expected not only to increase the overall number of mutations, but also the number of mutations whose beneficial effects can be traced back to structural changes in regulatory genes, especially those that encode repressor proteins, giving insight into the function of those regions whose structure has been altered.

To understand the impact that a mutator/suppressor founder has on the spectrum and fate of new beneficial mutations and on the dynamics of population structure, we repeated the classic Adams et al. experiments using the same ancestral strain and culture conditions [36]. Over the course of up to 500 generations, we monitored, at 50-generation intervals, the incidence of mutations that reached at least 1% frequency, identifying both beneficial and hitchhiking mutations. To determine which mutations co-occurred within a given lineage, we sequenced 96 clones from each population at the time point where we observed the greatest allelic diversity. We uncovered no evidence for stable subpopulation structure, but instead observed pervasive clonal interference, with only 17 out of 3346 mutations (of which a few hundred are likely beneficial) going to near fixation across replicate experiments. The temporal order in which certain mutations rose to high frequency was largely predictable, reflecting a high degree of parallelism among replicates. In general, mutations that enhanced glucose assimilation arose early, followed by mutations in global regulators and mutations that either increased efficiency of limiting resource utilization or mitigated the deleterious effects of certain earlier mutations. Our results show that when replicate populations of mutator-suppressor *E. coli* evolve under carbon limitation the number of allelic variants that exceed 1% frequency may be large, but the number of genes targeted is relatively few. We further show that in many cases the distribution of high-value mutations is clustered in regions essential for gene products to exert their regulatory function.

## Results

### Experimental design

Evolution experiments were carried out in triplicate under continuous nutrient limitation using Davis Minimal Medium [36], with glucose (0.0125% w/v) as the sole source of carbon for energy and growth. In addition to archiving samples as − 80 °C glycerol stocks, experimental populations were also monitored every 10–20 generations for culture purity by microscopy and by plating cultures onto a lawn of multiple *E. coli*-specific bacteriophage, as previously described by [41]. Chemostats (300 mL working volume) were run under aerobic conditions for 300–500 generations at constant temperature (30 °C) and at constant dilution rate (*D* = 0.2 h^−1^). Under these conditions, steady-state population density is ~ 10^8^ cells mL^−1^ and residual glucose concentration is at or below the limit of detection (Additional file 1: Fig. S1). The *E. coli* strain used to initiate these experiments, JA122, is distinguished from *E. coli* K12 by alleles likely to influence the spectrum of mutations arising during adaptive evolution (Additional file 2: Table S1 [34];). Among these is a nonsense mutation in MutY (Leu299*) that results in a mutation rate nearly 30-fold greater than K12 [34], nonsense mutations in genes that encode stationary-phase sigma factor RpoS (Gln33*) [42], and ‘housekeeping’ sigma factor RpoD (Glu26*), as well as a suppressor mutation in the *glnX* tRNA known to suppress amber, ochre, and opal mutations (Additional file 2: Table S1) [43].

To identify the mutations that arose during our experiments, we performed whole-genome, whole-population sequencing every 50 generations on each of the three chemostat populations. We generated approximately 50 million 2×100bp paired end reads per sample, yielding coverage of up to ~ 1000x for each time point (library insert sizes were selected to be short enough such that forward and reverse reads overlapped, which, while reducing coverage, increases quality; see the “Methods” section). We used these data to identify mutations that rose to an allele frequency of ~ 1% or greater. Given an effective population size of > 10^10^ and 300–500 generations of selection, it is highly improbable that any allele could reach such a frequency by drift alone [29]. We therefore assume that every mutation we identified had either come under positive selection or was hitchhiking along with one that had.

### Population sequencing reveals consistent mutation patterns across independent evolution experiments

Across all samples, 3346 SNPs were detected in 2083 unique genes or intergenic regions (Additional file 3: Table S6). The overwhelming majority (97.5%) of these SNPs were GC➔TA transversions, as expected given the ancestral strain’s defect in mismatch repair protein MutY, which encodes adenine glycosylase [44]. Consistent with the protein coding density of *E. coli* (87.8%) [45], 85% (2854) of SNPs occurred in coding regions. On average, 69.2% of these created a missense mutation, 23.4% resulted in a synonymous mutation, and 7.4% caused a nonsense mutation (Additional file 1: Fig. S2). Relative to proportions observed in mutation accumulation experiments carried out using wild-type *E. coli* [46], we observed more nonsynonymous and nonsense mutations. Given that MA experiments deliberately avoid selection pressure, through single-cell bottlenecks, while evolution experiments typically purge highly deleterious mutations, the greater fraction of nonsense mutations (7.4% vs. 3% in [46]) observed in our experiment is all the more striking. However, we note that a more appropriate comparison would be to compare a suppressed vs. a non-suppressed mutator strain to determine if an excess of nonsense mutations occurs as a result of suppression; this would require the existing, suppressed mutations in the background be reverted.

Small deletions were rarely detected (one single-nucleotide deletion in each of vessel 1 and vessel 2, and none detected in vessel 3), but we observed a single large ~ 150 kb duplication in vessel 2. The overall number of mutations in each population increased linearly over time and at approximately the same rate across replicates (Additional file 1: Fig. S2), as would be expected with a mutator phenotype.

### Comparison of population-level mutations reveals clonal interference and widespread parallelism

Despite the large number of SNPs detected across replicate populations, only 17 novel alleles ever approached fixation by exceeding 98% frequency. Moreover, the maximum frequency of most alleles never exceeded 10% (Additional file 1: Fig. S3A), and the vast majority of alleles were present at a lower frequency in the final time point than they were at their maximum (Additional file 1: Fig. S3B), as has been previously observed in population sequencing data [25]. Together, the foregoing observations suggest that in each evolution experiment population dynamics was largely driven by clonal interference [47]. A small number of loci were recurrently mutated above what would be expected by chance, indicating that variants at these loci were likely beneficial (Table 1, Additional file 2: Table S2). For example, a total of 212 mutations arose in the 10 most significantly mutated genes identified in the population sequencing data, with each gene receiving at least five mutations (Table 1). Moreover, 30 and 14 distinct allelic variants were discovered in just two: the genes encoding the DNA-binding repressor GalS and the RNA-binding protein Hfq, respectively (Additional file 2: Table S3). High-resolution population sequencing also revealed that 13 SNPs not present at the start of the experiment reached at least 1% frequency in all three vessels at various time points, while 52 SNPs recurred in two out of three chemostats (Additional file 2: Table S4). Thus, our data also provide compelling evidence for substantial parallel evolution at the genic level—indeed, with only two exceptions, genes containing beneficial mutations (as determined from the population sequencing) were mutated in either two or three of the chemostats (Additional file 1: Fig. S3C).

**Table 1:** Characteristics of frequently mutated genes

Rank	Gene	GO biological process	Observed mutations	Unique alleles	Expected mutations	Uncorrected *p* value	FDR
Population sequencing							
1	galS***	Regulation of transcription	38	30	0.78	6.55E−50	4.42E−45
2	hfq********	Regulation of Translation	24	14	0.23	6.91E−40	2.33E−35
3	pgi********	Glycolytic process	35	24	1.23	4.54E−38	1.02E−33
4	opgH**	Response to osmotic stress	31	29	1.90	8.74E−27	1.48E−22
5	malT*********	Regulation of transcription	30	19	2.02	8.10E−25	1.09E−20
6	malK*******	Carbohydrate transport	22	14	0.83	7.47E−24	8.40E−20
7	upstream mglB**	Transcription regulatory region	7	4	0.21	2.91E−09	2.81E−05
8	rho**	Transcription termination	11	9	0.94	5.49E−09	4.64E−05
9	upstream dnaG	Transcription regulatory region	5	5	0.08	3.06E−08	2.30E−04
10	fimH***	Cell adhesion/biofilm formation	9	5	0.68	4.38E−08	2.96E−04
Clonal sequencing							
1	hfq*******	Regulation of translation	26		0.1020	3.79E−53	3.68E−48
2	pgi****	Glycolytic process	17		0.5448	5.52E−20	2.68E−15
3	opgH***	Response to osmotic stress	17		0.8400	6.57E−17	2.13E−12
4	upstream mglB**	Transcription regulatory region	8		0.0925	1.22E−13	2.96E−09
5	fimH****	Cell adhesion/biofilm formation	10		0.2982	1.17E−12	2.26E−08
6	ompR***	Regulation of transcription	8		0.2377	2.05E−10	3.31E−06
7	upstream adhE*	Transcription regulatory region	6		0.1575	1.85E−08	0.000257
8	malT***	Regulation of transcription	10		0.8935	3.98E−08	0.000482
9	proQ*	Posttranscriptional regulation	6		0.2308	1.72E−07	0.001858
10	pfkA**	Glucose catabolic process	6		0.3180	1.09E−06	0.010612

Each asterisk indicates an allele that arose more than once independently, either within or between vessels

### Clonal sequencing further clarifies lineage relationships and parallelism

To establish linkage relationships between novel alleles, we sequenced 96 individual clones from each vessel. In each case, the 96 clones were isolated at random from the time point at which we detected the greatest number of mutant alleles at ≥ 5% frequency. To assess whether the frequency estimates from population sequencing were reasonable, and whether the isolated clones constituted a reasonable subsample, we compared frequencies of mutations identified in both datasets at the corresponding time point and found that they correlated well (Additional file 1: Fig. S4).

**Fig. 1:**
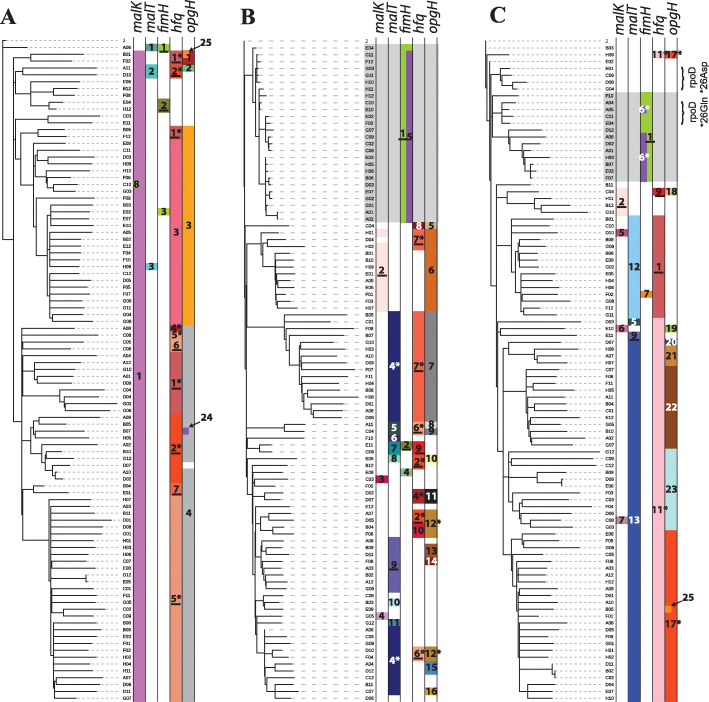
Clone phylogenies. Phylogenies depicting relationships among sequenced clones isolated from chemostats when allelic diversity attained its maximum; **a** chemostat 1, **b** chemostat 2, **c** chemostat 3. Distributions of different *malK*, *malT*, *fimH*, *hfq*, and *opgH* alleles are indicated by colored bars. For each gene, all alleles observed in the dataset are numbered (see Additional file 3: Table S6 for details of which number corresponds to which allele for each gene). Underlined numbers denote alleles independently observed in more than one chemostat, while numbers marked with an asterisk appear to have arisen more than once within the same vessel. Gray shading delineates clades comprised of clones that have not acquired the standard mutations related to enhanced glucose uptake and instead carry variant *fimH* alleles that contribute to biofilm formation. Bracketed clones in chemostat 3 exhibited mutations expected to revert the ancestral nonsense mutations in the housekeeping gene encoding sigma factor RpoD

For each set of 96 clones, we constructed a phylogeny to represent their putative evolutionary relationships (Fig. 1). Inspection of the mutations and phylogenetic trees from each vessel (i.e., each independent evolution) revealed several instances in which exactly the same mutation arose not only in different vessels, but often more than once in the same vessel on distinct branches of a given tree. In the most extreme case, 6 of the 11 *hfq* alleles detected via clone sequencing were identified in clones from different vessels, indicating independent parallel origins (Fig. 1, Additional file 4: Table S7). Furthermore, 7 of the 11 appear to have arisen more than once within the same vessel.

### Clonal dynamics are shaped by relationships among de novo alleles, hard and soft selective sweeps, and the absence of periodic selection

**Fig. 2:**
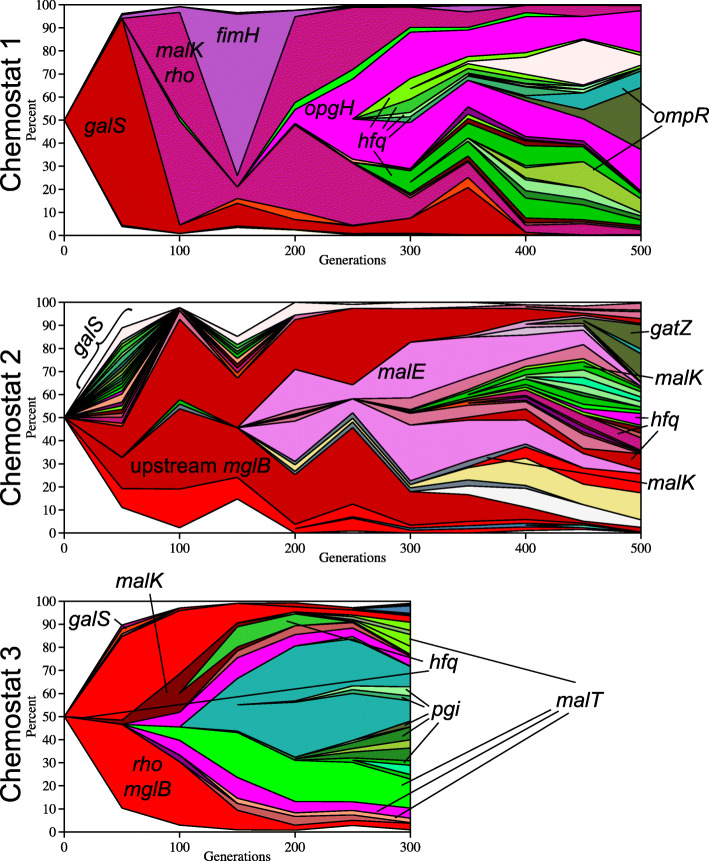
Muller diagrams. Evolutionary dynamics of adaptive lineages, deduced from combining whole-population whole-genome sequence data and whole-genome sequence data of individual clones isolated from each chemostat at the time point where allelic diversity reached its maximum value. Select genes are indicated in the plots. See Fig. 4 for further details. Also note, most mutations that went extinct by the sampling time point are not shown. See Additional file 3: Table S6 for their relative frequencies. Additional file 5: Fig. S9, Additional file 6: Fig. S10, and Additional file 7: Fig. S11 each contain a PDF with scrollable panels that depict evolutionary dynamics for > 50 individual genes in chemostats 1, 2, and 3, respectively

**Fig. 3:**
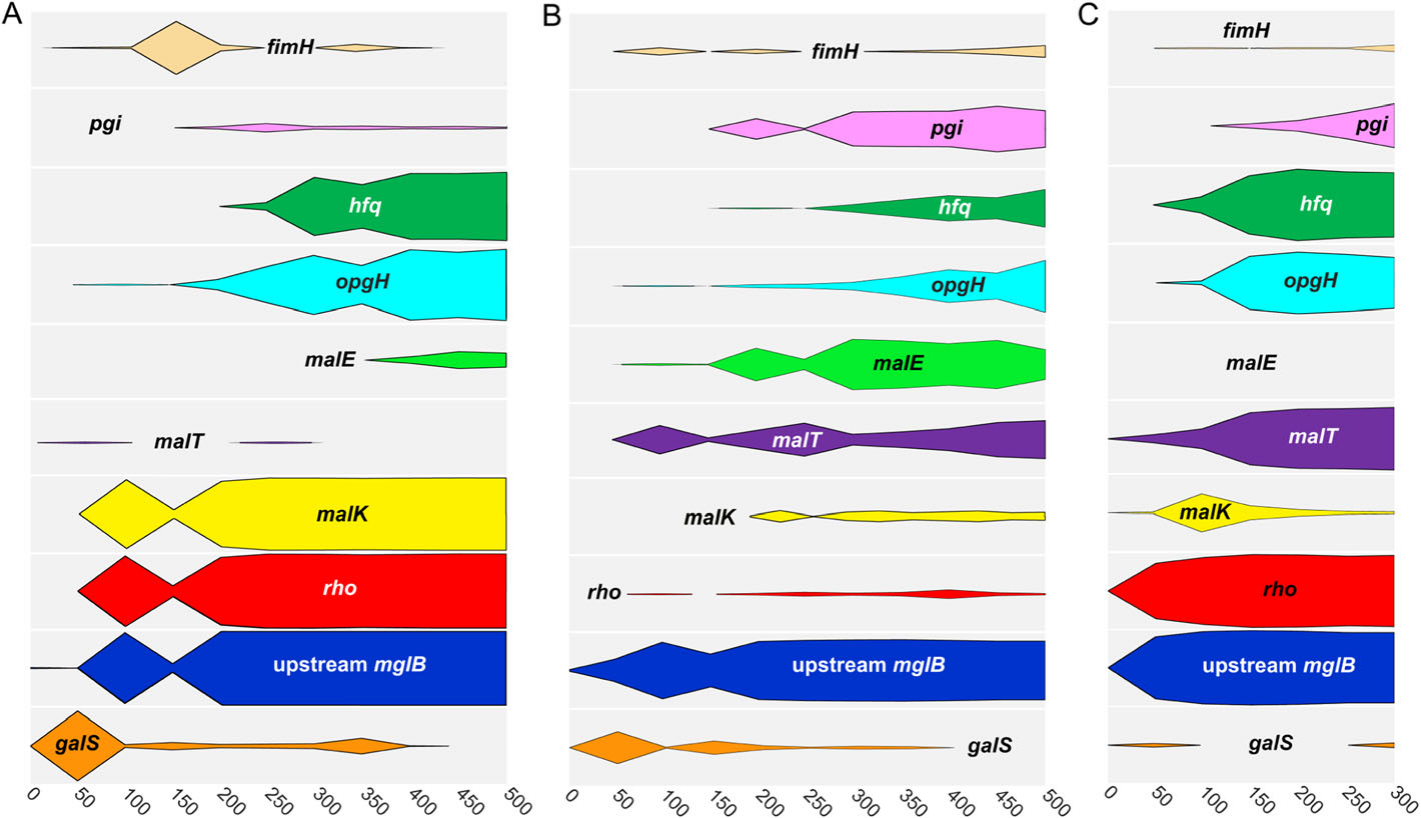
Population-level dynamics of mutations in 10 frequently hit genes show consistent patterns. For each panel, **a** chemostat 1, **b** chemostat 2, and **c** chemostat 3, the elapsed number of generations is depicted on the *x*-axis; the height of each gray box within each panel represents a frequency of 100%. Cumulative frequencies for all alleles of a given gene present in the population at each time point were calculated and are represented as colored plots

Combining population allele frequency data with linkage information inferred from clone sequencing makes it possible to depict lineage dynamics using Muller diagrams (Fig. 2, Additional file 5: Fig. S9, Additional file 6: Fig. S10, Additional file 7: File Fig. S11; Note: Additional files 5, 6, and 7 each contain a PDF with scrollable panels that depict evolutionary dynamics for > 50 individual genes). In general, we observed early, hard sweeps of highly beneficial mutations related to limiting nutrient influx, followed by soft sweeps [48–50] and multiple-origin soft sweeps that may fine-tune glucose uptake or utilization later in the experiment when diversity was higher [51–53]. Hard sweeps consistently involved mutations in regulators (*galS* in chemostat 1, transcriptional terminator *rho* in chemostats 1 and 3) or regulatory regions (upstream of *dnaG* in chemostat 1, upstream of *mglB* in chemostats 1, 2, and 3), while soft sweeps were comprised of both regulatory and operon-specific mutations (e.g., *hfq* and *opgH* in chemostats 1, 2, and 3, upstream of *adhE* in chemostat 1, *pgi* in chemostat 3) (Figs. 2 and 3, Additional file 5: Fig. S9, Additional file 6: Fig. S10, Additional file 7: Fig. S11 [49, 54]). Here, we note that multiple-origin soft sweeps may be especially prevalent in our experiments due to the ancestral mutator allele at *mutY*, as the likelihood of concurrent identical mutations in the same gene should increase with mutation rate.

### Early sweeps occur in genes that regulate influx of the limiting nutrient glucose

**Fig. 4:**
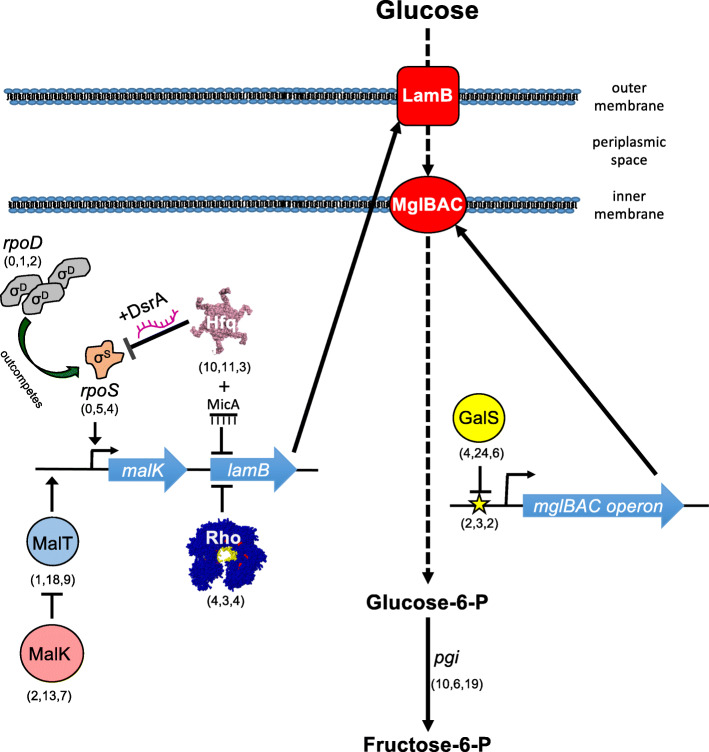
Overview of pathways relating some of the most frequently mutated genes to glucose transport and metabolism. Numbers in parentheses next to protein/gene names denote the number of mutant alleles found in each chemostat population over the course of 300–500 generations (also see Additional file 1: Table S3)

For specific growth rates between ~*μ* = 0.1 h^−1^ and *μ* = 0.9 h^−1^, glucose is most efficiently transported using a combination of the maltoporin LamB and the galactose transporter MglBAC, and glucose limitation tends to select for mutations that increase expression of these proteins [52, 55–63]. Accordingly, 7 of the top 10 frequently mutated genes/gene regions we observed (*galS*, upstream *mglB*, *malT*, *malK*, *hfq*, *rho*, and upstream *dnaG*) play a role in transcriptional regulation of LamB or MglBAC, either directly or through their interactions with global regulators (Table 1, Fig. 4).

#### Functional attributes and evolutionary dynamics of mutations in operon-specific regulators *galS* and *mglB*

When *E. coli* is cultured under glucose limitation, the chief route by which limiting substrate enters the cytoplasm is via the d-galactose/methyl-β-d-galactoside transporter MglBAC (Fig. 4 [62]). GalS is a negative regulator of *mglBAC* transcription, and in the absence of d-galactose, GalS binds the *mgl* operator to prevent open complex formation [64]. Loss-of-function mutations in *galS* or mutations upstream of *mglB* in the GalS repressor binding site (bp 2,238,647 C➔A) and/or the CRP activator site (bp 2,238,630 C➔A) have been previously observed in the early stages of a daptation to limiting glucose [40, 58, 65, 66]. In our experiments, we observed 33 mutations in *galS*, far exceeding what would be expected by chance.

**Fig. 5:**
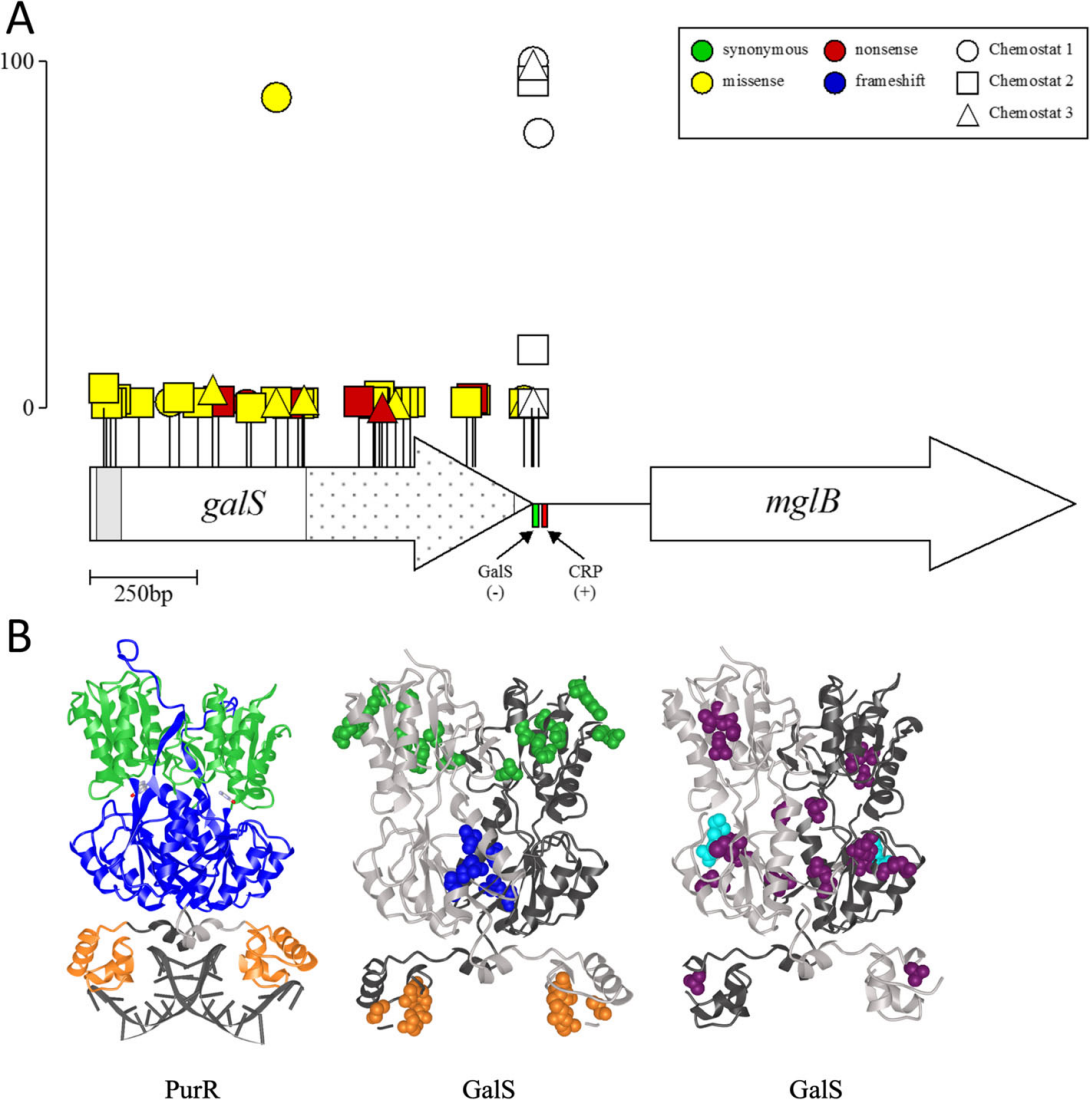
Recurrent mutations at *galS* and CRP-binding sites upstream of *mglB*. **a** Location and frequency of *galS* mutations on the primary structure. Circles represent alleles from chemostat 1, triangles represent alleles from chemostat 2, and squares represent alleles from chemostat 3. Synonymous mutations are colored green, missense mutations yellow, nonsense mutations red, and frameshift mutations blue. Scale bar (0–100) indicates frequency attained by a particular mutant in an experimental population. Gray shading indicates the GalS helix-turn-helix DNA-binding motif and stipple indicates the GalS ligand-binding domain. CRP-binding site mutations are not colored as they only alter DNA sequences. **b** Left: Ribbon diagram of dimeric *E. coli* purine repressor PurR bound to dsDNA. Three main functional regions of the protein are indicated: the N-terminal DNA-binding domain (orange), the C-terminal sub-domain involved in intramolecular signaling (blue), and the C-terminal sub-domain involved in dimer stabilization (green). The PurR ligand guanine is shown in gray cartoon style (PDBID 1WET) [67]. Middle: SWISSMODEL representation of the GalS repressor based on the structure of PurR (PDBID 1JFS, 32.53% sequence identity). Mutations grouped in the N-terminal DNA-binding domain are shown as orange spheres, while the two groups of C-terminal mutations indicated in **a** are shown in green and blue. Right: GalS model with conserved and repeatedly mutated residue Arg146 colored cyan and the remaining mutations that occurred in the middle portion of the protein colored purple

When all 33 *galS* mutations are mapped onto the primary protein sequence, many occur in both the helix-turn-helix portion of the DNA-binding domain (aa 4-23, gray shading in Fig. 5a) and in two distinct regions of the C-terminus (aa 211-252, stipple in Fig. 5a). Although no crystal structure is available for GalS, a homology model based on the PurR repressor shows that the spatial distribution of these three groups of mutations is consistent with their placement in the DNA-binding region, dimer stabilization region, and intramolecular signaling region, respectively (Fig. 5b) [67]. Moreover, the highest frequency mutation from chemostat 1 (Arg146Leu, also detected in chemostats 2 and 3) occurred in a conserved residue near or in the presumptive galactose-binding site (Fig. 5b) [68]. All are expected to result in loss of GalS function and consequently to enhance transcription of *mglBAC*.

Despite their early increase in frequency, few *galS* mutations persisted beyond generation 50 or attained a frequency greater than 5%. Instead, the majority of *galS* mutants was rapidly displaced by clones carrying highly beneficial mutations in the *mgl* operator sequence upstream of *mglB* (Figs. 2, 3, and 5a). The most successful mutation upstream of *mglB* (bp 2,238,647 C➔A) occurred in every vessel and increased in frequency to > 90% (Table S2, Additional file 5: Fig. S9, Additional file 6: Fig. S10, Additional file 7: Fig. S11). Exactly the same mutation has been observed in previous *E. coli* chemostat evolution experiments, demonstrating the enormous benefit this allele confers under glucose limitation, regardless of nuances afforded by chemostat setup, dilution rate, or strain [40, 52, 58]. Over the remainder of the experiment, only three other mutations upstream of *mglB* reached the threshold for detection: two were within 2 base pairs of the first mutation and did not rise to high frequency, while the third (chemostat 1, 2,238,630 C➔A), located in the CRP activator binding site, co-occurred with 2,238,647 C➔A and increased to ca. 80% frequency by generation 500 (Figs. 2 and 3a, Additional file 4: Table S7). These findings suggest additional mutations that affect GalS repressor binding are not of great benefit after the preferred allele has swept the population, whereas mutations that modulate the activity of other regulators (i.e., CRP) can act synergistically.

#### Functional attributes and evolutionary dynamics of mutations in genes that directly regulate LamB expression: malT and malK

**Fig. 6:**
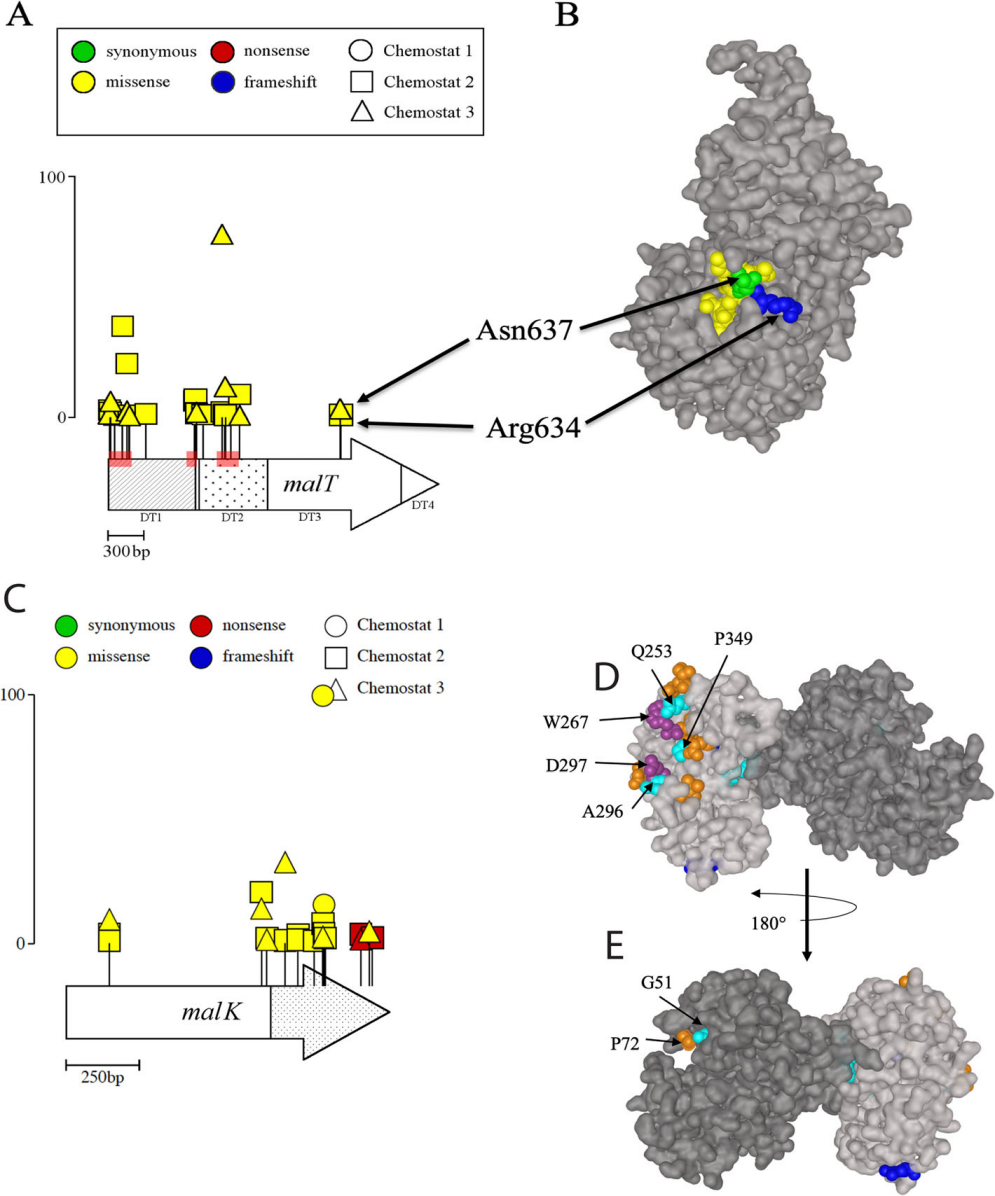
Recurrent mutations in *lamB* regulators *malT* and *malK.* **a** Location and frequency of *malT* mutations on the primary structure. Circles represent alleles from chemostat 1, triangles represent alleles from chemostat 2, and squares represent alleles from chemostat 3. Scale bar (0–100) indicates frequency attained by a particular mutant in an experimental population. The MalT protein consists of four structural domains (DT1–4) that function in nucleotide binding, effector sensing, and interaction with MalK (see text for details). **b** Crystal structure of MalT DT3 with residues identified by Richet et al. [71] as important for MalT/MalK interaction are colored. Asn637 and Arg634 were mutated in our data set and are colored green and blue, respectively. Residues that are part of the MalK contact site but were not mutated are colored yellow. **c** Location and frequency of *malK* mutations on the primary structure. The N-terminal nucleotide-binding domain is colored white, and the C-terminal regulatory domain is shown in stipple. **d** Location of mutations on the 3D structure of a single MalK monomer. The C-terminal regulatory domain is colored light gray, and the N-terminal nucleotide-binding domain is colored dark gray. Observed nonsense mutations (blue, aa 339, 352), missense mutations observed here and reported to cause constitutive *mal* expression (purple, aa 267 and 297), missense mutations observed here but not reported elsewhere (cyan, aa 51, 225, 231, 253, 286, 296, 298, 349), and missense mutations reported to cause increased *mal* expression but not seen in this study (orange, aa 72, 248, 250, 251, 262, 268, 291, 346, 350) all occur in the same region of the C-terminal regulatory domain. **e** View of a MalK monomer with domains and mutations as in **b** rotated 180° along the *y*-axis

Increased expression of the gene encoding outer membrane glycoporin LamB is another hallmark feature of *E. coli* adapted to glucose-limited chemostat growth [40, 52, 57, 59, 60, 69]. Previous experiments have shown that under glucose limitation, LamB overexpression can result from any one of the following: constitutive activation of transcriptional regulator MalT, disruption of the MalT inhibitor MalK, mutation of the RNA chaperone Hfq, alteration of sigma factor dynamics (σ^S^/σ^D^ ratio), or mutation of the *malT* repressor Mlc (Fig. 4) [40, 52, 57–60, 69, 70]. Across replicate experiments, we observed 19 unique *malT* alleles and 14 unique *malK* alleles (Fig. 6, Additional file 1: Fig. S5, Additional file 2: Table S3). Over half of the *malT* mutations (10 out of 19) are known either to cause MalT to become constitutively active, or to occur in amino acids involved in MalT/MalK interaction (Fig. 6a) [52, 71, 72]. Likewise, the majority of MalK substitutions (10 out of 14 different alleles, Fig. 6a) occurred within or just outside of the MalK/MalT interaction domain and are predicted to weaken MalT inhibition [73, 74].

The diversity of *malT* mutations reflects the diversity of signals integrated by MalT. All were missense substitutions, and all fell roughly into four clusters that correspond to a nucleotide-binding domain (aa cluster 4-60), a linker region/winged-helix domain (aa clusters 236-358 and 311-319), and a small patch of the maltotriose sensor domain (aa cluster 634-637). Mutations in the nucleotide-binding domain and winged-helix domain almost precisely delineate regions of the primary sequence previously identified by Schlegel et al. [75] as associated with a *mal* constitutive phenotype (Fig. 6a, red shading) [52, 71, 72]. The fourth cluster of mutations in the sensor domain (Arg634Ser/Arg634Leu/Asn637Lys) is on the surface of MalT in a 7 amino acid stretch of DT3 that serves as a point of contact between MalT and MalK (Fig. 6b) [71]. Mutations in this region eliminate MalK inhibition, thereby increasing transcription of *mal* genes. We recovered no mutations in the DNA-binding domain of MalT (effector domain), consistent with the expectation that our adaptive variants retain the ability to activate MalT-responsive promoters [71, 76, 77].

Ten of 14 substitutions in MalK occurred in the C-terminal 2/5 of the protein and were primarily located in or underneath predicted MalT binding sites (Fig. 6c) [73, 74]. Mutations in this region affect MalT inhibition and promote the expression of *mal* genes, but do not generally affect maltose transport, reflecting the dual role and two-domain nature of MalK [73, 78]. Comparatively few mutations (4 out of 14 alleles) occurred in the N-terminal nucleotide-binding domain. However, amino acid 51 was mutated three times over during the course of the experiment and is situated adjacent to proline 72, modification of which has also been shown to decrease MalK regulatory activity (Fig. 6d) [73, 74].

The dynamics of *malT*/*malK* allele frequency differs among experimental populations. In chemostat 1, MalK Ala296Asp rapidly sweeps to fixation, whereas in chemostat 3, early mutations in MalK (yellow) are displaced by later mutations in MalT (purple) (Fig. 3a, c). In chemostat 2, clones with either *malK* or *malT* mutations co-exist through all 500 generations (Additional file 1: Fig. S5). The reason for this contrast in dynamics cannot be attributed to emergence of a single “most fit” allele, as the majority types from chemostats 1 and 3 arose independently in chemostat 2, but did not sweep. Despite the fact that MalT and MalK are high-value targets of selection during adaptation to glucose limitation, other advantageous mutations (upstream *mglB*, *rho*, and *hfq*, discussed below) may have ultimately carried “winning” *mal* alleles in chemostats 1 and 3 to higher frequency, purging allelic diversity at this locus. Interestingly, although we observed 30 *malT* and 22 *malK* mutations in the population sequencing data (Table 1), with mutations being observed in 117 and 116 of the sequenced clones respectively, in only 5 out of the 288 sequenced clones do mutant alleles of these two genes co-occur, suggesting that there may be little or no additional advantage or even some disadvantage (due to reciprocal sign epistasis [79]) to having both. A lack of co-occurrence *of malT* and *malK* mutations has also been observed in previous evolution experiments [36], in which the primary resource (glucose) specialist carries a mutation in MalK and the secondary resource specialists share a mutation in MalT [40].

#### Functional attributes and evolutionary dynamics of mutations in global regulators that enhance glucose assimilation: Hfq, rho, and the t1 terminator

Hfq is a global regulatory protein that facilitates translation and/or RNA degradation by mediating ncRNA-mRNA interactions. It participates in a wide range of cellular processes including nutrient uptake, motility, and metabolism and is also a general regulator of stress response via interactions with mRNAs that encode sigma factors σ^S^, σ^E^, and σ^H^ [80, 81]. *hfq* mutations identified in other glucose-limited evolutions were found to be pleiotropic, increasing translation of LamB glycoporin, reducing levels of stationary-phase transcription factor RpoS, inhibiting cellular aggregation, and enhancing glucose transport via PtsG [70, 82].

*Hfq* is one of the most frequently mutated genes in our experiments: 24 *hfq-*independent mutations were recovered by population sequencing, comprising 14 unique *hfq* alleles; when the experiments were terminated, > 50% of cells in each population carried an *hfq* mutation (Additional file 2: Table S3; Table 1). Two of these alleles arose independently in all three vessels (same nucleotide position, same SNP), and six additional alleles were observed in two of three vessels (Additional file 2: Table S4). The frequency of and parallelism exhibited among *hfq* mutations is particularly striking in the context of experiments by Maharjan et al. where *hfq* mutations also arose, but remained at low frequency, being subject to negative frequency-dependent selection and epistatic interaction with *rpoS* mutations [38, 56, 69, 70].

Rho is a global regulator required for termination of nearly half of all *E. coli* transcripts. Like *hfq*, *rho* mutations can also be pleiotropic, resulting in either reduced or enhanced termination [83–88]. *rho* mutants have been recovered in *E. coli* populations adapted to high temperature [89, 90], ethanol stress [84, 85], and carbon source variation/limitation [29, 34, 91]. Mutagenesis and ChIP-chip analyses have shown that Rho-dependent terminators occur within genes that come under selection during glucose limitation, notably *lamB*, *mglA*, and *mglC* and downstream of *malT* and *mglC* [92, 93] and defective LamB expression in MalT activator mutants can be restored by compensatory mutations in *rho* [94].

**Fig. 7:**
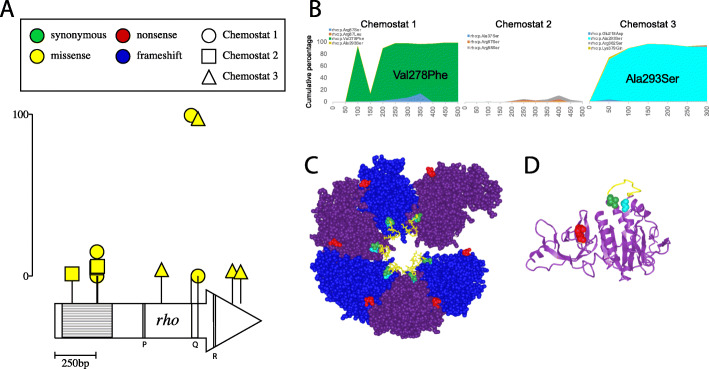
Recurrent mutations in global regulator *rho*. **a** Location and frequency of mutations along the primary structure. Circles represent alleles from chemostat 1, triangles represent alleles from chemostat 2, and squares represent alleles from chemostat 3. Scale bar (0–100) indicates frequency attained by a particular mutant in an experimental population. The N-terminal primary RNA-binding domain (aa 22-116) is shown in stipple. P-loop (aa 179-183), Q-loop (aa 278-290), and R-loop (aa 322-326) residues are indicated with corresponding letters. **b** Rho allele frequencies over time for chemostats 1, 2, and 3. **c** Crystal structure of *E. coli* Rho (PDB ID 1PVO) showing the location of high-frequency mutations in panel **b**. Subunits A–F are depicted counterclockwise from the upper right. Val278 is shown in green, Ala293 in cyan, Arg87 in red, and Q-loop residues in yellow stick representation. **d** Detail view ribbon representation of a single Rho subunit (PDB ID 2HT1) with Val278, Ala293, Arg87, and Q-loop residues colored as in panel **c**

Rho typically functions as a hexamer and termination requires binding of the RNA transcript as well as ATP hydrolysis. The primary RNA-binding domain is in the N-terminal half of the protein (aa 22-116) and the C-terminal half contains one ATP-hydrolysis domain (P-loop, aa 179-183) and two secondary RNA-binding domains (the Q-loop aa 278-290 and R-loop aa 322-326) (Fig. 7) (as reviewed in [83]). Overall, we observed 11 mutations at 8 residues in Rho, 5 of which are associated with RNA-binding domains (aa 37, 87, 88, 278, and 293) and 4 of which interact with their neighboring subunit in the hexameric protein (aa 87, 88, 218, and 278) (Fig. 7a). Primary RNA-binding domain mutations at residues 87-88 formed a cluster both in the amino acid sequence as well as the 3D structure (Table 1, Fig. 7). In addition, two high-frequency mutations (chemostat 1 Val278Phe, 93% by generation 100; chemostat 3 Ala293Ser, 68% by generation 50) occurred on either side of the Q-loop secondary RNA-binding domain (Fig. 7c, d) [95, 96]. We hypothesize that these high-frequency *rho* mutations enhance cells’ capacity to scavenge limiting glucose by contributing to increased expression of both glycoporin LamB and inner membrane transporter MglBAC [92, 93, 97].

*rho* mutations in chemostats 1 and 3 fixed or nearly fixed early and did so in concert with mutations in MalK (chemostat 1 Ala296Asp) and mutations upstream of *mglB* (chemostats 1 and 3, bp 2,238,647) (Figs. 2 and 3; Additional file 2: Table S3 and Table S5; Additional file 5: Files S3; Additional file 7: File S5). By contrast, *rho* alleles detected in chemostat 2 never exceeded 6% frequency (Figs. 2, 3**,** and 7b; Additional file 2: Table S3).

Competition between sigma factors RpoD (σ^D^) and RpoS (σ^S^) for binding to core RNA polymerase directs transcription of genes sensitive to both sigma factors [98, 99]. Because many genes important for nutrient scavenging are downregulated when σ^S^ is abundant, loss-of-function mutations in *rpoS* are frequently favored during glucose-limited chemostat growth [100–102]. Our ancestral strain contains nonsense mutations in both *rpoS* (Gln33*) and *rpoD* (Glu26*) but with limited read-through of both transcripts enabled by the *sup*E44 suppressor [34].

*rpoD* transcript abundance is partly controlled by transcriptional termination at a highly conserved 31-nt rho-independent terminator (T_1_) between *rpsU* and *dnaG* (Additional file 1: Fig. S8) [103]. T_1_ mutations predicted to decrease terminator stability were found early in chemostat 1 (bp 3,209,075 C➔A, 92% frequency by generation 50) and later in chemostat 2 (bp 3,209,076 C➔A and 3,209,082 G➔T); one of these also occurred in the Helling et al. experiments (bp 3,209,075 C➔A), suggesting it is beneficial under glucose limitation [36, 40]. We also cataloged a duplication in chemostat 2 that increases *rpoD* copy number. Although no duplications or SNPs in T_1_ were observed in chemostat 3, in this chemostat, a lineage carrying an intragenic suppressor mutation in RpoD (*26Tyr) expanded to 4.7% of the population by generation 350. Overexpression of RpoD enabled by T_1_ read-though, duplication, or intragenic suppression may ultimately increase transcription of operons positively controlled by σ^70^ (e.g., *mglBAC* and *malK-lamB-malM*), counteracting any residual σ^S^ stress response made possible by the supE44 suppressor.

### Mutations that impact energy conservation, membrane biogenesis, and transcriptional run-through are later-arising targets of selection

#### Phosphoglucoseisomerase (Pgi)

Pgi is an abundantly expressed glycolytic enzyme that catalyzes the isomerization of hexose phosphates, thereby acting in both glycolysis and gluconeogenesis, and modulating pentose phosphate pathway (PPP) flux. While *pgi* is not essential in *E. coli*, *pgi* knockout mutants grow slowly on glucose, accumulate cAMP, re-route glucose-6-phosphate through the PPP, experience redox stress due to accumulation of NADPH, and utilize the glyoxylate shunt rather than the full TCA cycle [104–108]*.* Deletion of *pgi* has also been shown to favor increased *mglBAC* and *lamB* transcription via CRP-cAMP and targeted degradation of *ptsG* [104, 109]. In most species, including *E. coli*, functional Pgi exists as a dimer and mutation of residues across the interface between monomers has been hypothesized to alter subunit interactions and catalytic center geometry [110, 111].

**Fig. 8:**
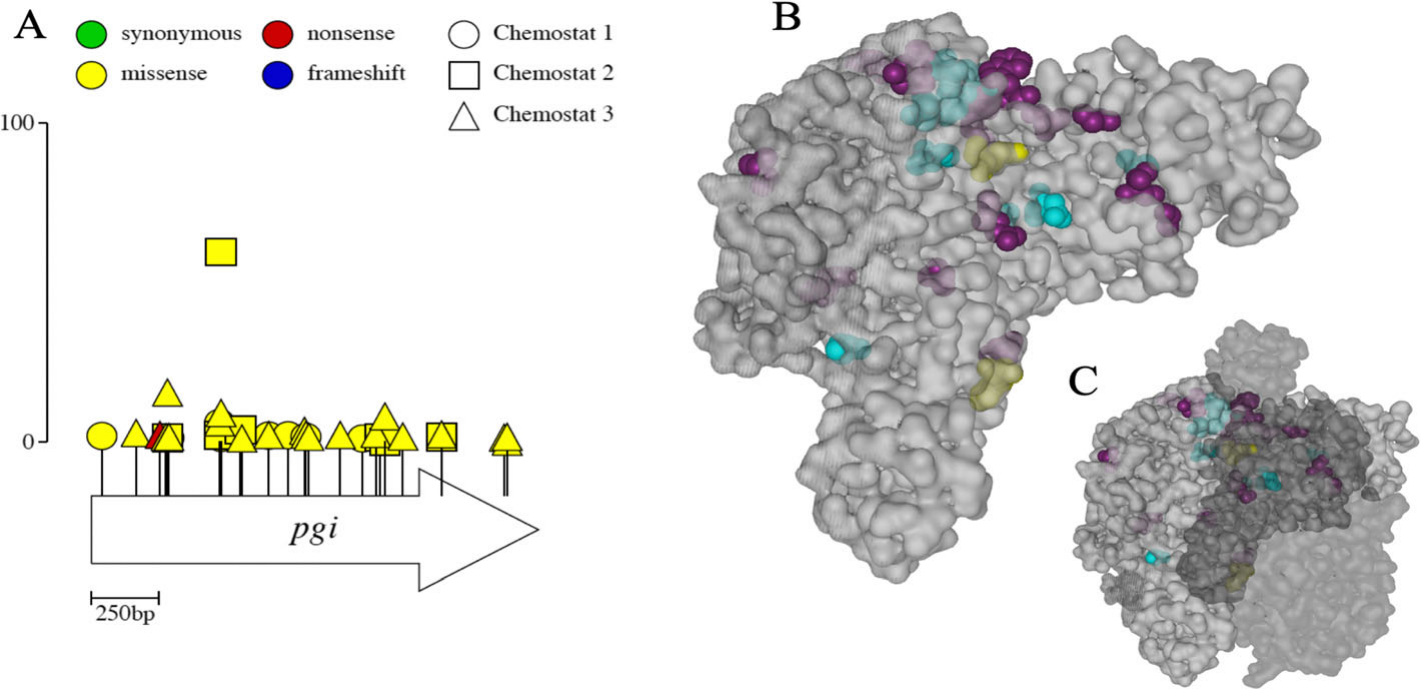
Recurrent missense mutations in *pgi*. **a** Location and frequency of mutations along the primary structure. Circles represent alleles from chemostat 1, triangles represent alleles from chemostat 2, and squares represent alleles from chemostat 3. Scale bar (0–100) indicates frequency attained by a particular mutant in an experimental population. **b** Surface representation of a single Pgi monomer with mutations observed in two or more chemostats colored cyan, those that occurred in only one chemostat colored purple, and active site residues Glu355, His386, and Lys514 colored yellow. **c** Crystal structure of the Pgi dimer. Colors are as in **b** with the second subunit shown in translucent dark gray to highlight mutations that occur at the interface between the two subunits

Over the course of three replicate evolution experiments, we detected a total of 35 *pgi* alleles, 24 of which were unique (Additional file 2: Table S3, Fig. 8a. The large number of different variants suggests there must be some adaptive benefit to mutation of *pgi* and that the benefit is more likely due to reduced rather than enhanced Pgi function. When mapped onto the protein’s 3D structure, many of the mutations localize to the dimerization interface (or just below it) and to the area near active site residues Glu355, His386, and Lys514 (Fig. 8b). Amino acid changes in either of these areas could be expected to inhibit Pgi activity.

Few *pgi* mutations rose to appreciable frequency before generation 200 (Fig. 3), suggesting their benefit may be contingent on other mutations or on some aspect of the chemostat environment that changed after this time point. *Pgi* alleles were least successful in chemostat 1, which was also the only replicate in which a large fraction of clones (79% by generation 500) acquired a second mutation upstream of *mglB*. This observation suggests that *pgi* mutations and mutations in the CRP-binding site of the *mglBAC* promoter may be functionally redundant.

#### Membrane glycosyltransferase

OpgH is involved in the synthesis of periplasmic glucans, highly branched oligosaccharides made from β-linked glucose monomers. While no *opgH* mutations are observed before generation 100, once they do appear, they rapidly increase in frequency, usually either just before or just after *hfq* mutations (Fig. 2, Additional file 1: Fig. S6, Additional file 5: Fig. S9, Additional file 6: Fig. S10, Additional file 7: Fig. S11). Novel *opgH* alleles, especially the nonsense mutations that we frequently observe (Additional file 1: Fig. S7), may constrain glucan production and serve as a glucose conservation measure. Also, a “moonlighting” function has recently been reported for OpgH: the glucosyltransferase interacts with the tubulin-like cell division protein FtsZ to delay cell division when levels of UDP-glucose are low [112]. Thus, OpgH mutations may augment the rate of cell division and thereby provide a fitness advantage under slow-growth chemostat conditions. The only *opg* operon mutation identified among strains in previous Adams et al. experiments occurred in *opgG* of the glucose scavenger, CV103 (E487*) [34]; we also observed 5 mutations in *opgG*.

### Mutations that impact cell adhesion persist at low levels throughout our experiments

#### Fimbrial protein genes (*fim*)

Genes associated with production/function of type 1 fimbriae, particularly *fimH* (fimbrial adhesion), were an unexpected and frequent target of mutation in all three chemostats (Table 1, Figs. 1 and 3; Additional file 2: Table S3; Additional file 5: Fig. S9, Additional file 6: Fig. S10, Additional file 7: Fig. S11). Though novel *fim* alleles were transient in vessels 2 and 3, in chemostat 1, a FimH Asn54Lys (corresponding to Asn33Lys in the mature protein) variant rose to a frequency of 70% by generation 150, temporarily displacing high-fitness alleles in *rho*, *malK*, and upstream *mglB* (Additional file 5: Fig. S9). FimH mutants sometimes show an increased capacity for biofilm formation [113], a recurrent issue in chemostat experiments. However, as we failed to observe *fimH* mutant lineages acquire mutations expected to enhance glucose metabolism, the selection of *fimH* mutations here was more likely related to bacterial persistence than to competition for the limiting resource.

## Discussion and conclusions

### History matters: ancestry influences evolutionary trajectory

The evolutionary trajectory taken by a clonal population depends on its genetic point of departure. Our departure point was an ancestor carrying nonsense mutations in mismatch repair and in housekeeping and stationary-phase sigma factors, RpoD and RpoS, respectively. However, the ancestor also carried an amber/ochre/opal nonsense tRNA suppressor. Populations descending from such an ancestor carry an increased mutational load, but also have a built-in mechanism to lighten that load, specifically suppressing the burden of nonsense mutations that would otherwise result in loss-of-function (LOF). Laboratory evolution studies support the notion that LOF mutations can help drive adaptation [25, 114–116]. After all, metabolic networks can be reconfigured more easily by abolishing existing function(s) than by evolving altogether new ones [115]; thus, nonsense mutations or deletions sometimes confer greater fitness benefit than missense mutations affecting the same gene [116]. However, LOF may reduce metabolic flexibility by limiting the capacity of LOF mutants to compete in alternative environments [25].

Because our genetic “point of departure” was an *rpoS* nonsense mutant, it might be viewed as being pre-adapted to life under glucose limitation. After all, *rpoS* has repeatedly been shown to be a high-value target of selection under nutrient limitation (e.g., [38, 117, 118]); RpoS normally outcompetes housekeeping sigma factor RpoD for binding to RNA polymerase, repressing genes required for growth and cell division and activating those required to enter stationary phase [102, 119]. *rpoS* mutants with impaired function therefore continue to divide under conditions where wild-type cells arrest. However, the combined phenotypic effect of ancestral *rpoS* and *rpoD* nonsense mutations in a suppressor background is murky and begs the question: Is this particular combination of mutations favorable under glucose limitation, merely tolerated, or detrimental? Further, while many of the genetic changes we observed (e.g., those in *galS*, upstream *mglB*, *hfq*) enhance glucose assimilation, occur repeatedly, and go to high frequency, we also recovered clones that carry none of these mutations. Instead, these clones carry either intragenic suppressors of the ancestral *rpoD* nonsense mutation (*26➔Asp and *26➔Gln, chemostat 3 (Fig. 1)) or a duplication that increases *rpoD* copy number. Even when adaptation to one selective pressure is facilitated by LOF, and loss makes it difficult to adapt to another selective pressure, nonsense mutations have a distinct advantage over indels, because reversion or suppression of nonsense mutations is possible should environmental conditions change [25].

Another ancestral allele having the potential to influence evolutionary trajectory was the A➔T CRP-binding site mutation 224 bp upstream of the low-*K*_*m*_ acetate-scavenging enzyme, *acs* (acetyl-CoA synthetase). This mutation alters the regulation of the *acs-pta* operon such that the ancestor poorly assimilates low levels of the overflow metabolite acetate. In previous evolution experiments, this allele sometimes selectively favored semi-constitutive *acs* mutants capable of assimilating overflow acetate and attenuating its growth-inhibitory effects [35, 36, 39]. While we uncovered no evidence for this outcome, our failure to do so was not unanticipated: cross-feeding arose in only half the experiments founded by this ancestor or its close relatives [39]. A recent model [37] defining the boundary conditions for cross-feeding to evolve in a chemostat showed that the process is sensitive to variation in dilution rate as well as to the relative fitness of mutants that gain access to secondary metabolites. Subtle differences in either of these parameters may account for the apparent absence of the interaction. It is noteworthy that we detected no mutations at any of the loci previously implicated in cross-feeding evolution, e.g., *acs*, *lpd*, and *ptsI* [34, 120].

A large body of evidence points to acetyl-CoA synthetase being the route by which *E. coli* scavenge low levels of acetate from the extracellular environment [121, 122]. Indeed, to the best of our knowledge, this is the only route, though in principle a large decrease in the *Km* for acetate kinase (*ack*) might open another. However, we observed no mutations in *ack*. We also cannot rule out the possibility that multiple, different *acs*-mutant lineages co-exist, each below our detection limit, but together summing to > 1%. However, we believe this is unlikely to have occurred in three independent replicates. Finally, we cannot exclude the possibility that a low-frequency acetate-scavenging contaminant arose and persisted in each of the three experiments, repeatedly escaping detection by our sensitive phage cocktail assay (see the “Methods” section).

A more plausible explanation lies in this: continuous glucose limitation selects not only for glucose scavenging, but also for efficiency of resource use [123, 124]. Because the latter places a premium on conserving the limiting carbon, we suggest that yet-to-be explored mutations among those we observed have the effect of restricting acetate overflow, which would confer a selective advantage. Consistent with this interpretation are observations that not all experimental populations which evolve glucose scavengers evolve acetate scavengers [39]. Adaptive lineages in the experiments reported here apparently found ways other than cross-feeding to consume all available carbon. As expected, throughout each evolution experiment, steady-state population size remained constant and residual glucose was at or below the limit of detection. Residual acetate (~ 45–90 μM) was observed early in each experiment (Additional file 1: Fig. S1), presumably owing to the ancestral *acs* regulatory mutation, but then fell below detection limit after generation 200. One possible mechanism for increased efficiency of glucose utilization lies in the proliferation of *pgi* mutants: generation 200 coincides with the emergence of novel *pgi* alleles in all three populations. Yao et al. reported that when *pgi* deletion mutants were grown in glucose-limited chemostats, glucose uptake rate dropped slightly compared to wild-type, but no overflow acetate was produced and biomass yield remained unchanged [125].

### Population and clone sequencing open up a detailed view of the full spectrum of beneficial mutations and how that spectrum changes over time

High-coverage, whole-genome, whole-population sequencing makes it possible to discover every new allele reaching > 1% frequency in a population of > 10^10^ cells. Because alleles are unlikely to reach such frequencies by drift, all were either transiently beneficial or hitchhiking with alleles that were. This depth of analysis opens up a richly detailed view of the spectrum of beneficial mutations arising in *E. coli* under constant resource limitation. Periodic whole-population sequencing allows patterns to be discerned as to how these spectra change over time, while clone sequencing makes it possible to establish linkage relations among novel alleles and represent their collective fate as evolving lineages.

Five general patterns emerge from these analyses. *First,* new alleles accumulate in replicate populations at similar rates, and the proportion of alleles that are missense, nonsense, synonymous, or noncoding remains fairly constant. *Second,* the distribution of new mutations across the genome is skewed with regard to their targets, with only a few dozen of the more than 1000 mutated genes being mutated more frequently than would be expected by chance alone; yet even among those most frequently mutated genes, few de novo mutations fix over the course of these experiments. *Third,* by clonal sequencing, we are able to establish that many, independent lineages co-exist and compete within the continuous cultures. Thus, evolutionary dynamics in these populations is governed by clonal interference and not by clonal replacement or clonal reinforcement. This conclusion is reinforced by lack of evidence for mutations that support interactions which give cross-feeding consortia higher fitness and productivity than any consortium member by itself [120]. The dynamics of *galS* replacement illustrates the effect that clonal interference can have on the fate of different alleles. In chemostat 1, clones carrying GalS Arg146Leu rapidly dropped in frequency when lineages emerged with a mutation upstream of *mglB* (position 2,238,647); however, they were not completely displaced until generation 400 and even enjoyed brief periods of expansion. In chemostat 2, clones with the same mutation upstream of *mglB* were present by generation 50, but did not surpass a 90% threshold for another 250 generations due to competition from 22 different *galS* lineages and a lineage carrying a different upstream of *mglB* allele (2,238,648 G➔T) (Figs. 2 and 3b, Additional file 4: Table S7). By contrast, in chemostat 3, a lineage with the upstream *mglB* mutation (2,238,647) experienced little competition and was nearly fixed by generation 150 (Additional file 2: Table S5).

A *fourth* pattern to emerge is widespread parallelism in regulatory evolution. Both across and within populations, the same genes are mutated again and again, often at exactly the same nucleotide position in independent replicates, and sometimes in independent lineages co-evolving within the same vessel. Many of these genes (*galS*, *malT*, *malK*, upstream *mglB*, *hfq*, *rho*) act in processes related to the transport and assimilation of the limiting nutrient, glucose. However, in most cases, the mutations recovered alter regulation of these processes, and not the structural proteins that carry them out. This finding is consistent with a number of recent experimental evolution studies using *E. coli*, where the genetic basis for adaptive change could be traced back to mutations in regulatory elements or regulatory loci, e.g., [126–129] and references therein.

A *fifth* pattern relates to the order of beneficial mutations and the influence that order has on evolutionary dynamics. Consistent with previous reports, mutations that increase glucose flux across the inner membrane (*galS*, upstream *mglB*) occur early and precede those that increase flux across the outer membrane (*malK*/*malT*, *hfq*, *rho*). In both cases, mutations in binding partners (GalS/upstream *mglB* and MalT/MalK) rarely occur in the same clone, and the order in which they occur can lead to either a sweep (upstream *mglB* clones quickly displace *galS* clones) or to clonal interference (*malT* and *malK* clones can co-exist). Other alleles emerge later and nearly always together: clones with existing mutations in the *mal* operon acquire subsequent mutations in *hfq* and *opgH*, regardless of which gene is altered first or which alleles are already present in the population. These patterns are reminiscent of genotypes described by Kinnersley et al. [22] in which glucose scavenger CV103 has mutations in *malK*, *opgG*, and *hfq* while acetate specialist CV101 only carries a mutation in *malT.*

With regard to periodic selection, rather than favorable alleles arising within a set of lineages that successively replace one another over time, we observe groups or cohorts of mutations co-evolving, with widespread clonal interference among lineages that carry different beneficial mutations [130]. For example, in chemostat 1, a spreading lineage with a cohort of mutations upstream of *mglB*/*lptA*/*opgH* (pink) is impeded by the emergence of lineages carrying mutations in *hfq* (green) (Additional file 5: Fig. S9). All of these phenomena—hard and soft sweeps, cohorts of mutations that increase or decrease in frequency together, and clonal interference—have been observed in yeast [18, 24, 131] and *E. coli* [29] populations evolving in the laboratory, as well as in *Pseudomonas aeruginosa* evolving in the cystic fibrosis lung [132].

Similar experiments carried out by Maharjan et al. [38] using *E. coli* BW2952 showed that population-level phenotypic changes in glucose-limited chemostats are often the result of multiple soft sweeps by combinations of beneficial mutations. While we did not assay clone phenotypes, multiple alleles of *galS*, *hfq*, and *opgH* appear to sweep our populations in concert suggesting a similar pattern in which a phenotypic effect (reduced expression of a particular gene) is favored, but has different genetic bases in co-existing lineages. At the clone level, BW2952 exhibits sign epistasis between mutations in *rpoS*/*hfq* and *galS*/*malT* [38, 56]. In our experiments, we found no evidence of sign epistasis between the ancestral *rpoS* allele and *hfq*: by generation 250, over 50% of clones in populations 1 and 3 carry mutations in both genes. Maharjan et al. have suggested that fitness deficits in *rpoS*/*hfq* double mutants may arise from altered cell division [69, 133], specifically, *hfq* mutations that enhance glucose uptake during slow growth, may diminish viability when cells divide rapidly. Hfq deletion mutants exhibit cell division anomalies due to elevated expression of cell division proteins, including FtsZ [134, 135]. Interestingly, during fast growth, OpgH (which in our experiments is nearly always mutated alongside *hfq*) binds FtsZ to postpone cell division [112]. Thus, it may be that in our experiments the negative fitness effects associated with *hfq-rpoS* double mutants are mitigated by mutations in *opgH*. We should also note that cells in the Maharjan et al. evolution experiments were subject to a dilution rate of *D* = 0.1 h^−1^, whereas those in experiments performed by Adams et al. were dividing twice as fast (*D* = 0.2 h^−1^). Thus, this discrepancy may be a manifestation of trade-offs between glucose uptake and cell viability. Finally, some mutations occur repeatedly and are therefore likely adaptive, yet their dynamics are unpredictable: for example, beneficial mutations in transcriptional terminator *rho* sweep when they co-occur with beneficial mutations upstream of *mglB,* but otherwise remain at low frequency (Fig. 3, Additional file 2: Table S3). This dependence on genetic context, or “quasi-hitchhiking,” of beneficial mutations was previously observed by Lang et al. in yeast and may be a feature that only becomes evident when experimental populations are sequenced to high depth of coverage and at sufficient temporal resolution [18].

Classic studies by Adams and colleagues showed that populations originating from the same ancestor used in our experiments could evolve into consortia consisting of ecotypes that co-exist via cross-feeding [35, 36] and relief from product inhibition [37]. Recent data have shown that such consortia can be more fit and are more productive than either their ancestor or clones representing each ecotype [120]. By contrast, in the populations described here, neither the observed spectrum of mutations nor the structure of clone phylogenies suggest trophic interactions that could be construed as clonal reinforcement. This finding is consistent with Treves et al. [39] who found that cross-feeding arose in only 6 of 12 replicate *E. coli* populations evolved under conditions identical to those we used and founded by closely related ancestors. In each of these populations, acetate scavenging was driven by regulatory mutations that resulted in semi-constitutive overexpression of acetyl-CoA synthetase (*acs*) [39]. These mutants were supported by glucose-scavenging small colony variants that were evident on plates between generations 88 and 381, depending on replicate. In our experiments, *acs* mutants never arose, or if they did, their frequency never exceeded our 1% level of detection. Likewise, mutations thought to contribute to the acetate excretion phenotype [34] were not observed either in our population sequencing or clone sequencing data. Thus, as was the case for half the populations examined by Treves et al. [39], the molecular mechanisms to support acetate cross-feeding were not established over the time course of our experiments.

## Methods

### Strains, media, and culture conditions

*Escherichia coli* JA122, population samples and clones were maintained as permanent frozen stocks and stored at − 80 °C in 20% glycerol. Davis minimal medium was used for all liquid cultures with 0.025% glucose added for batch cultures and 0.0125% for chemostats, as previously described [40]. Chemostat cultures were initiated using independent colonies picked from a Tryptone Agar (TA) plate inoculated with JA122, then outgrown in Davis minimal batch medium overnight. Consistent with previous evolution experiments founded by this ancestor, chemostats were maintained at 30 °C with a dilution rate of ≈ 0.2 h^−1^ for 300–500 generations. Every other day, population samples were archived in duplicate at − 80 °C; culture density and purity were assessed by measuring absorbance at A_550_ and by plating serial dilutions on TA and examining colony-forming units (CFU) following 24-h incubation at 30 °C. When necessary, chemostats were re-started from frozen stocks (chemostat 1: generation 217; chemostat 2: generation 410; chemostat 3 generation 251). At each sequencing time point, 50 mL of culture was pelleted then stored at − 80 °C for DNA extraction. For clone sequencing, entire colonies were picked from TA plates inoculated from glycerol stocks and re-archived in 96-well plate format.

#### Phage cocktail assay of culture purity

To assay chemostat cultures for contamination, lysates of bacteriophages T2, T5, and a T6 mutant were prepared using the ancestral strain JA122 as a host, following procedures first described by [41]. Lysates were filtered through a 0.2-μM filter to remove cell debris and concentrated with a 10-kDa MWCO filter to bring the concentration of each phage to 2.5 × 10^12^ mL^−1^. Every 10–20 generations, 200 μL of phage cocktail was applied to the surface of a TA agar plate, dried, and used to screen 100 μL of chemostat culture for non-*E. coli* contaminants.

### Metabolite assays

Ten milliliters of sterile, cell-free chemostat filtrate was concentrated 20-fold by lyophilization (Labconco 4.5 Liter Freeze Dry System), then re-suspended in 0.5 mL sterile Millipore water. Residual glucose and residual acetate concentrations were determined on concentrated filtrate. Glucose was assayed enzymatically using the High Sensitivity Glucose Assay Kit (Sigma-Aldrich, Cat# MAK181), while acetate concentration was determined using the Acetate Colorimetric Assay Kit (Sigma-Aldrich, Cat# MAK086). Results presented in Additional file 1: Fig. S1 represent means ± SEM of duplicate assays.

### Population sequencing

Bacterial DNA was prepared using the DNeasy Blood and Tissue Kit (Qiagen, cat. 69504) following the manufacturer’s guidelines. For population sequencing, 5 × 10^10^ cells, collected from every 50 generations in three chemostat vessels (up to 500 generations in vessels 1 and 2, and up to 300 generations in vessel 3, 29 samples total) and frozen as pellets, yielded 10–20 μg of DNA. Following Proteinase K treatment, RNaseA treatment was used (20 μL 10 mg/mL RNAse A, 2 min at room temperature) to avoid degraded RNA from visually obscuring size selection during library preparation. Samples were split into two columns to avoid overloading. Bacterial DNA was sheared to a 150–200-bp fragment size using a Covaris S2 series sonicator (6 min, duty = 5%, intensity = 3, cycles/burst = 200) and was then ligated to barcoded adapters as described [136], except that 200-bp fragments were size selected after adapter ligation (to maximize the fidelity of sequencing, by reading each fragment in both directions). Six barcoded libraries were combined and sequenced on each lane of HiSeq 2000 Illumina Sequencer.

### Variant calling from population sequencing with CLC Genomics Workbench 7.5

Illumina reads were trimmed (removing adapters on both ends) and stringently mapped (mismatch cost 2, insertion cost 3, deletion cost 3, length fraction 1.0, similarity fraction 0.97) to the reference sequence (WIS_MG1655_m56). Variants were called with the following parameters: minimum frequency 1%, minimal coverage 100, minimum count 2, and base quality filtering (neighborhood radius 5, minimum central quality 15, and minimum neighborhood quality 20). Sequencing data uncovered low-level contamination of whole population samples with *Serratia liquifaciensis.* We therefore first determined the proportion of contaminating reads by mapping population sequencing to *S. liquifaciensis* genome and then removed SNPs with frequency closely tracking the percentage of contamination (between 1 and 5%) that matched *S. liquifaciensis* sequence.

### Selection of clones for sequencing

Allele frequencies for each chemostat were examined at each time point, and the time point at which there was the largest number of alleles present at 5% or greater frequency was chosen for the isolation of clones for whole-genome sequencing. The rationale for this was that it would afford us the greatest opportunity to phase as many high-frequency alleles as possible.

### Clonal DNA preparation

A colony was re-suspended in 300 μL of sterile ddH_2_0 with 17% glycerol and stored in three aliquots at − 80 °C. One hundred microliters of glycerol stock was used for DNA preparation. After removing glycerol (using MultiScreen High Volume Filter Plates with 0.45 μm Durapore membrane, Millipore MVHVN4525), cells were re-suspended in 500 μL LB and grown overnight at 30 °C without shaking in deep well plates. Cells were collected again using filter plates and subjected to DNeasy 96 Blood and Tissue Kit (Qiagen 69581) (yielding 4–15 μg per strain).

### Clonal library preparation and sequencing

Multiplexed sequencing libraries from clones were prepared using the Nextera kit (Illumina catalog # FC-121-1031 and # FC-121-1012) as described in [137], starting with 1–4 ng of genomic DNA. Resulting libraries from each 96-well plate were pooled at an equal volume. Resulting pooled libraries were analyzed on the Qubit and Bioanalyzer platforms and sequenced on HiSeq 2000 (one lane per 96 clone pool). All raw sequencing data are available from the SRA under BioProject ID PRJNA517527.

### Variant calling from clonal sequencing with CLC Genomics Workbench 7.5

Short reads with adapters removed were mapped to the reference with the same parameters as above, except the length fraction was set to 0.5, and the similarity fraction to 0.8. Variants were called with a minimum frequency 80%, minimum count 2, and the same base quality filtering as above.

### Generation of phylogenies

For each chemostat, SNP and indel events for all 96 clones and the ancestor JA122 were concatenated and re-coded as binary characters (i.e., presence/absence with the ancestral state composed of all zeroes) and assembled into character matrices. PAUP ver. 4.0a149 was used to generate Camin-Sokal parsimony trees using the ancestor as the outgroup under the assumption that reversions were extremely unlikely due to the extreme transversion bias [138, 139]. Tree files (.tre) were loaded into the Interactive Tree of Life (iTOL) web service for character mapping and figure generation [140].

### Determining genes with an excess of mutations

To identify genes with an excess of mutations, we first determined the overall density of mutations as:

*ρ* = M/L, where M is the total number of mutations and *L* is the length of the genome.

The probability of a given mutation landing in a segment of length *l* is:

*λ* = *ρ* × *l*

To calculate the *p* value of *n* mutations landing in a segment of length *l*, we assume a Poisson sampling process of a mutation landing in a given segment and thus use:

$$ p=\sum \limits_{i=n}^{\infty}\frac{\uplambda^i\ \mathrm{x}\ {e}^{-\uplambda}}{i!} $$

though, in practice, we capped *i* arbitrarily at 50, as continually summing at *i* > 50 does not appreciably affect the calculated *p* value. For a given segment, we calculated the number of segments that would be expected to have *p* value as good or better, as the number of tested segments multiplied by the *p* value. From this, we also determined a false positive rate.

### Generation of Muller diagrams

Based on both the clonal sequencing, we were able to determine which mutations were in which lineages together, and from both the clonal and population sequencing an approximate order of those mutations (though this was not exhaustive for all mutations). Using these data, we developed a lineage file format that described which mutations occurred in which lineages, and which lineages descended from one another, and used a custom Perl script that combined this information with the allele frequencies over time from the population sequencing to generate a graphical representation of the evolutionary dynamics, often referred to as a Muller diagram.

## Supplementary Information

**Additional file 1 Fig. S1:** Cell density and residual metabolite concentrations. **Fig. S2:** Input of de novo mutations. **Fig. S3:** Most de novo mutations only reach low allele frequencies and experience pervasive clonal interference. (A) Histogram of maximum frequencies; (B) Final vs. maximum frequencies; (C) Venn diagram showing degree of genic parallelism among beneficial mutations. **Fig. S4:** Isolated clones are representative of the populations from which they are drawn. **Fig. S5:** MalK/MalT population dynamics. **Fig. S6:** Mutations in glucosyltransferase *opgH* occur repeatedly and, collectively, go to high frequency**. Fig. S7:** *opgH* has nonsense and missense mutations throughout its length. **Fig. S8:** Mutations that decrease T1 terminator stability in the macromolecular synthesis operon are expected to affect expression of *dnaG* (DNA primase) and *rpoD* (housekeeping σ-factor). Predicted ΔG values for wild-type T1 terminator from *E. coli* K12 MG1655 and two variants observed in chemostat 1 were determined using unafold.rna.albany.edu/?q=mfold/RNA-Folding-Form. The C➔A mutation at nucleotide 3,209,075 has been previously observed in chemostat-evolved *E. coli* [34].

**Additional file 2 Table S1.** Key mutations that distinguish ancestral strain JA122 from K12 (MG1655). **Table S2.** Beneficial alleles. **Table S3**. Population allele frequencies for frequently mutated genes**. Table S4.** Identical mutations arise within and among replicate evolution experiments. **Table S5.** Fixed alleles among replicate populations (“fixed” defined as > 98% at any time point between generation 50 and 500).

**Additional file 3 Table S6.** Spreadsheet containing identity and frequencies of all mutations detected via population sequencing.

**Additional file 4 Table S7**. Alleles mapped onto clone phylogenies represented in main Fig. 1.

**Additional file 5 Fig. S9.** Muller diagrams for novel alleles arising in chemostat 1, showing details for each lineage.

**Additional file 6 Fig. S10.** Muller diagrams for novel alleles arising in chemostat 2, showing details for each lineage.

**Additional file 7 Fig. S11.** Muller diagrams for novel alleles arising in chemostat 3, showing details for each lineage.

**Additional file 8 Fig. S12.** Locations of mutations in genes that were targets of adaptation, and their maximum frequencies, on both log and linear scales.

## Data Availability

All raw sequencing data are available from the SRA under BioProject ID PRJNA517527 [141].
