# Supplementary material for: Evolutionary dynamics and structural consequences of de novo beneficial mutations and mutant lineages arising in a constant environment
Source: BMC Biol. 2021 Feb 4;19:20. doi: 10.1186/s12915-021-00954-0 (PMC7863352; doi:10.1186/s12915-021-00954-0)

Figure S1

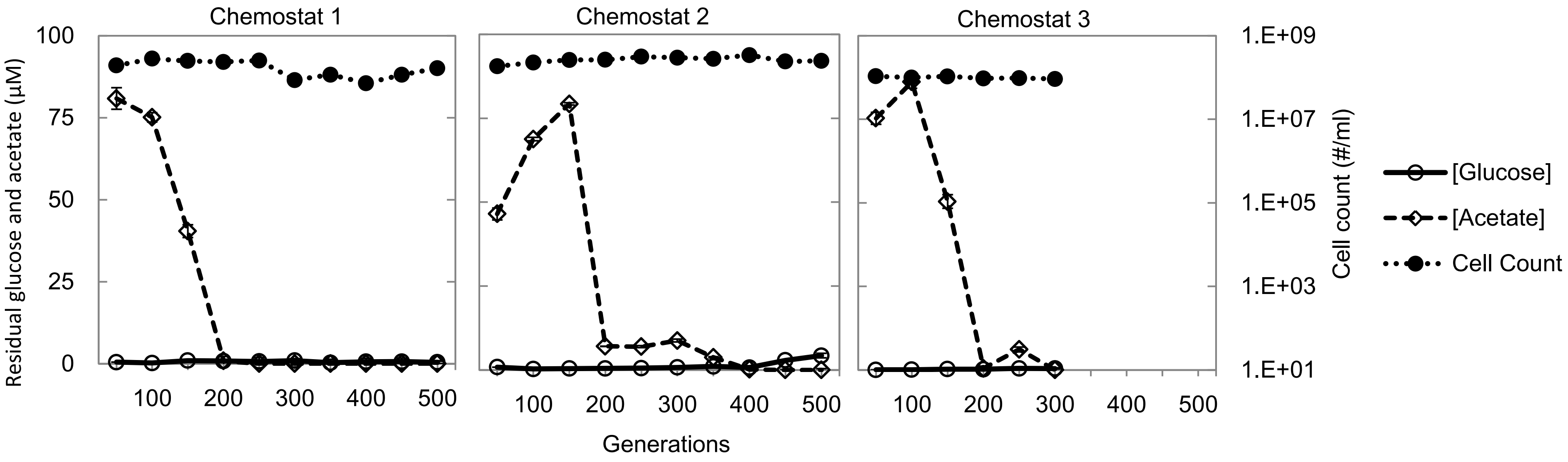

Chemostat 1
Chemostat 2
Chemostat 3
Residual glucose and acetate (µM)
100
75
50
25
0
100
200
300
400
500
Generations
1.E+09
1.E+07
1.E+05
1.E+03
1.E+01
Cell count (#/ml)
[Glucose]
[Acetate]
Cell Count

Figure S2

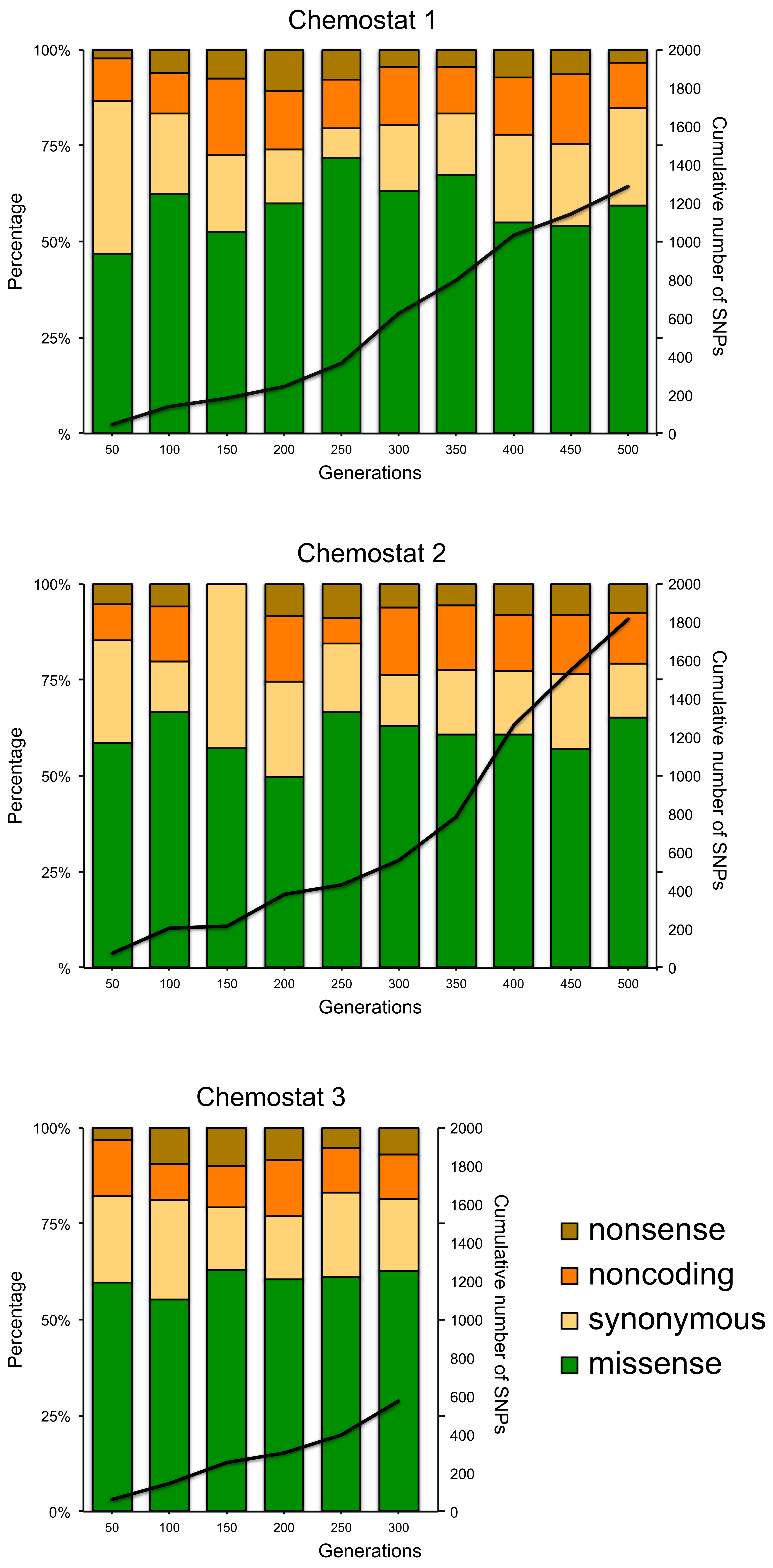
Chemostat 1
Percentage
Cumulative number of SNPs
Generations
100%
75%
50%
25%
%
50
100
150
200
250
300
350
400
450
500
2000
1800
1600
1400
1200
1000
800
600
400
200
0
Chemostat 2
Percentage
Cumulative number of SNPs
Generations
Chemostat 3
Percentage
Cumulative number of SNPs
Generations
0%
nonsense
noncoding
synonymous
missense

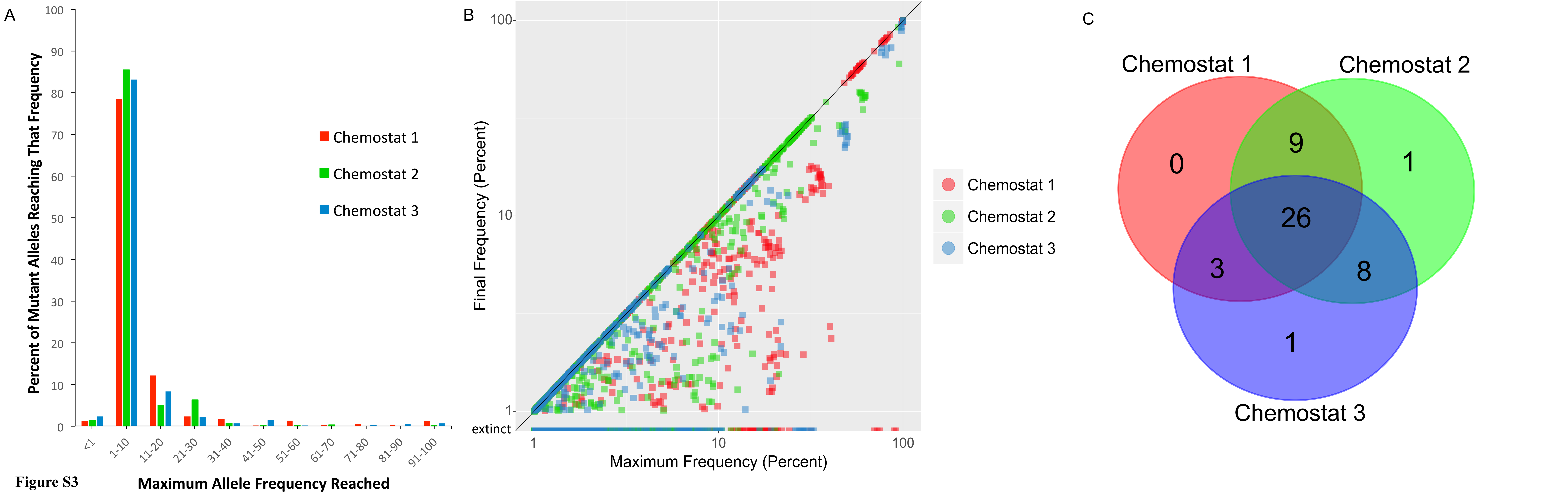
A
100
90
80
70
60
50
40
30
20
10
0
Percent of Mutant Alleles Reaching That Frequency
Chemostat 1
Chemostat 2
Chemostat 3
<1
1-10
11-20
21-30
31-40
41-50
51-60
61-70
71-80
81-90
91-100
Maximum Allele Frequency Reached
B
100
10
1
extinct
Final Frequency (Percent)
1
10
100
Maximum Frequency (Percent)
Chemostat 1
Chemostat 2
Chemostat 3
C
Chemostat 1
Chemostat 2
Chemostat 3
0
9
1
26
3
8
1

Figure S3

Figure S4

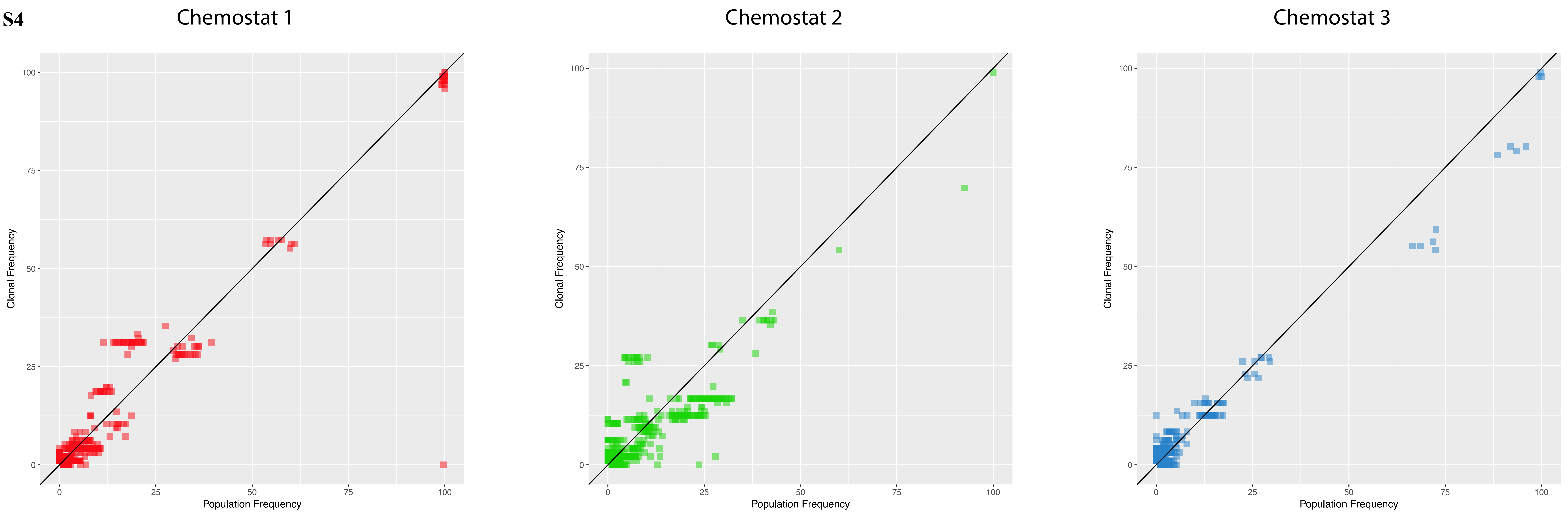
Chemostat 1
Clonal Frequency
Population Frequency
0
25
50
75
100
Chemostat 2
Clonal Frequency
Population Frequency
0
25
50
75
100
Chemostat 3
Clonal Frequency
Population Frequency
0
25
50
75
100

Figure S5

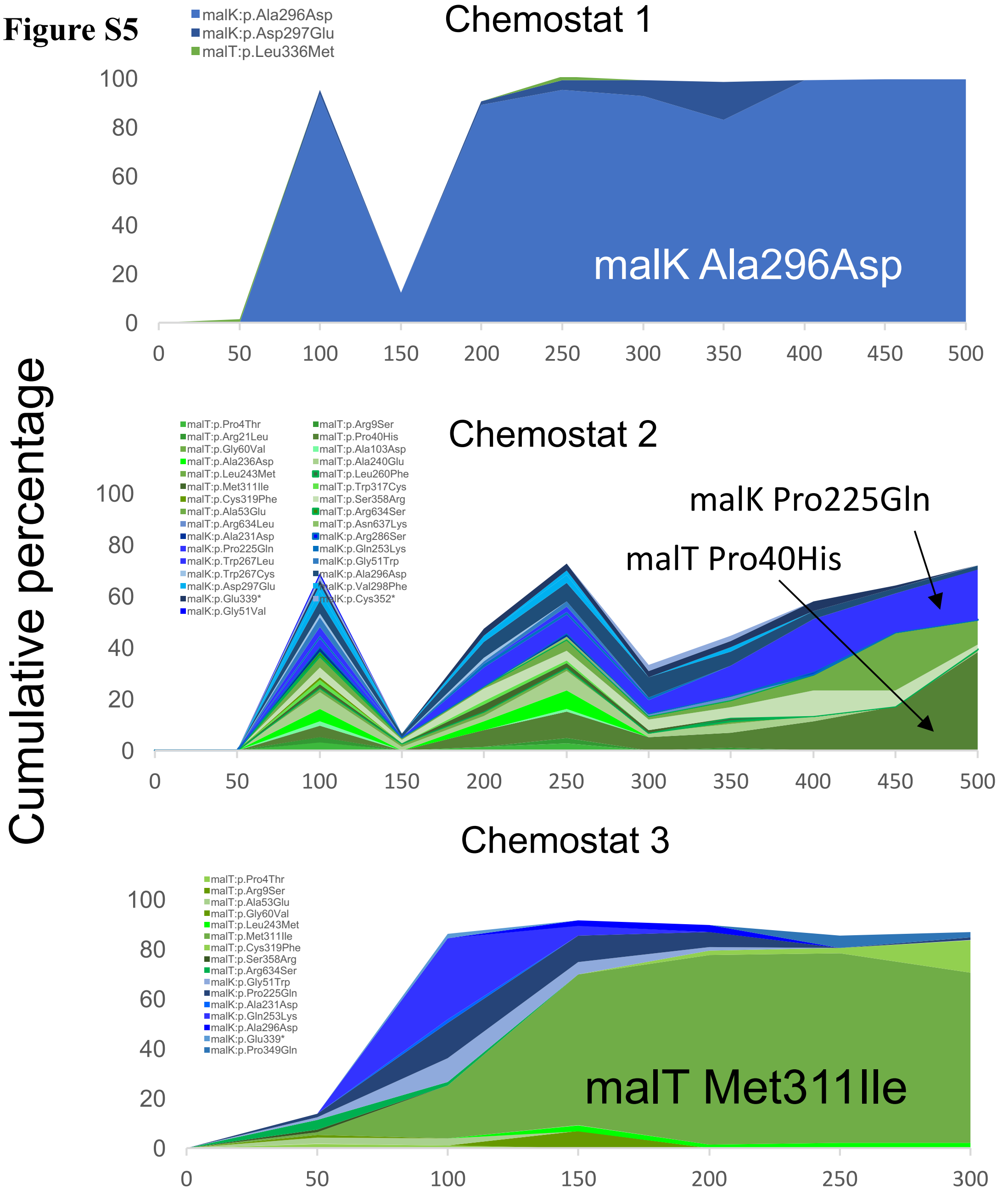

malK:p.Ala296Asp
malK:p.Asp297Glu
malT:p.Leu336Met
Chemostat 1
100
80
60
40
20
0
0
50
100
150
200
250
300
350
400
450
500
malK Ala296Asp
Cumulative percentage
malT:p.Pro4Thr
malT:p.Arg21Leu
malT:p.Gly60Val
malT:p.Ala236Asp
malT:p.Leu243Met
malT:p.Met311Ile
malT:p.Cys319Phe
malT:p.Ala53Glu
malT:p.Arg634Leu
malK:p.Ala231Asp
malK:p.Pro225Gln
malK:p.Trp267Leu
malK:p.Trp267Cys
malK:p.Asp297Glu
malK:p.Glu339*
malK:p.Gly51Val
malT:p.Arg9Ser
malT:p.Pro40His
malT:p.Ala103Asp
malT:p.Ala240Glu
malT:p.Leu260Phe
malT:p.Trp317Cys
malT:p.Ser358Arg
malT:p.Arg634Ser
malT:p.Asn637Lys
malK:p.Arg286Ser
malK:p.Gln253Lys
malK:p.Gly51Trp
malK:p.Ala296Asp
malK:p.Val298Phe
malK:p.Cys352*
Chemostat 2
malK Pro225Gln
malT Pro40His
Chemostat 3
malT:p.Pro4Thr
malT:p.Arg9Ser
malT:p.Ala53Glu
malT:p.Gly60Val
malT:p.Leu243Met
malT:p.Met311Ile
malT:p.Cys319Phe
malT:p.Ser358Arg
malT:p.Arg634Ser
malK:p.Gly51Trp
malK:p.Pro225Gln
malK:p.Ala231Asp
malK:p.Gln253Lys
malK:p.Ala296Asp
malK:p.Glu339*
malK:p.Pro349Gln
malT Met311Ile

Figure S6

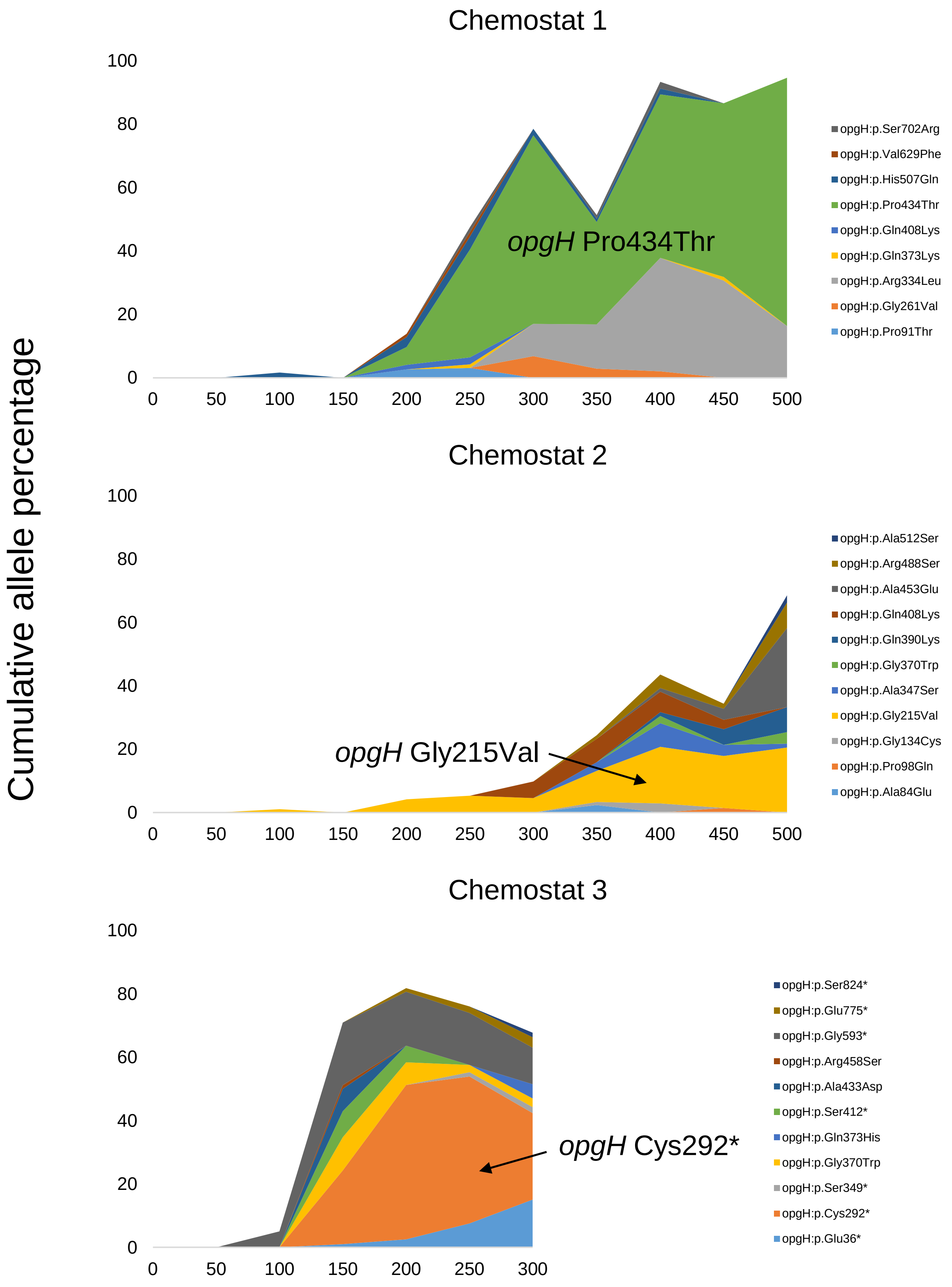
Chemostat 1
Cumulative allele percentage
100
80
60
40
20
0
0
50
100
150
200
250
300
350
400
450
500
opgH Pro434Thr
opgH:p.Ser702Arg
opgH:p.Val629Phe
opgH:p.His507Gln
opgH:p.Pro434Thr
opgH:p.Gln408Lys
opgH:p.Gln373Lys
opgH:p.Arg334Leu
opgH:p.Gly261Val
opgH:p.Pro91Thr
Chemostat 2
opgH Gly215Val
opgH:p.Ala512Ser
opgH:p.Arg488Ser
opgH:p.Ala453Glu
opgH:p.Gln408Lys
opgH:p.Gln390Lys
opgH:p.Gly370Trp
opgH:p.Ala347Ser
opgH:p.Gly215Val
opgH:p.Gly134Cys
opgH:p.Pro98Gln
opgH:p.Ala84Glu
Chemostat 3
opgH Cys292*
opgH:p.Ser824*
opgH:p.Glu775*
opgH:p.Gly593*
opgH:p.Arg458Ser
opgH:p.Ala433Asp
opgH:p.Ser412*
opgH:p.Gln373His
opgH:p.Gly370Trp
opgH:p.Ser349*
opgH:p.Cys292*
opgH:p.Glu36*

Figure S7

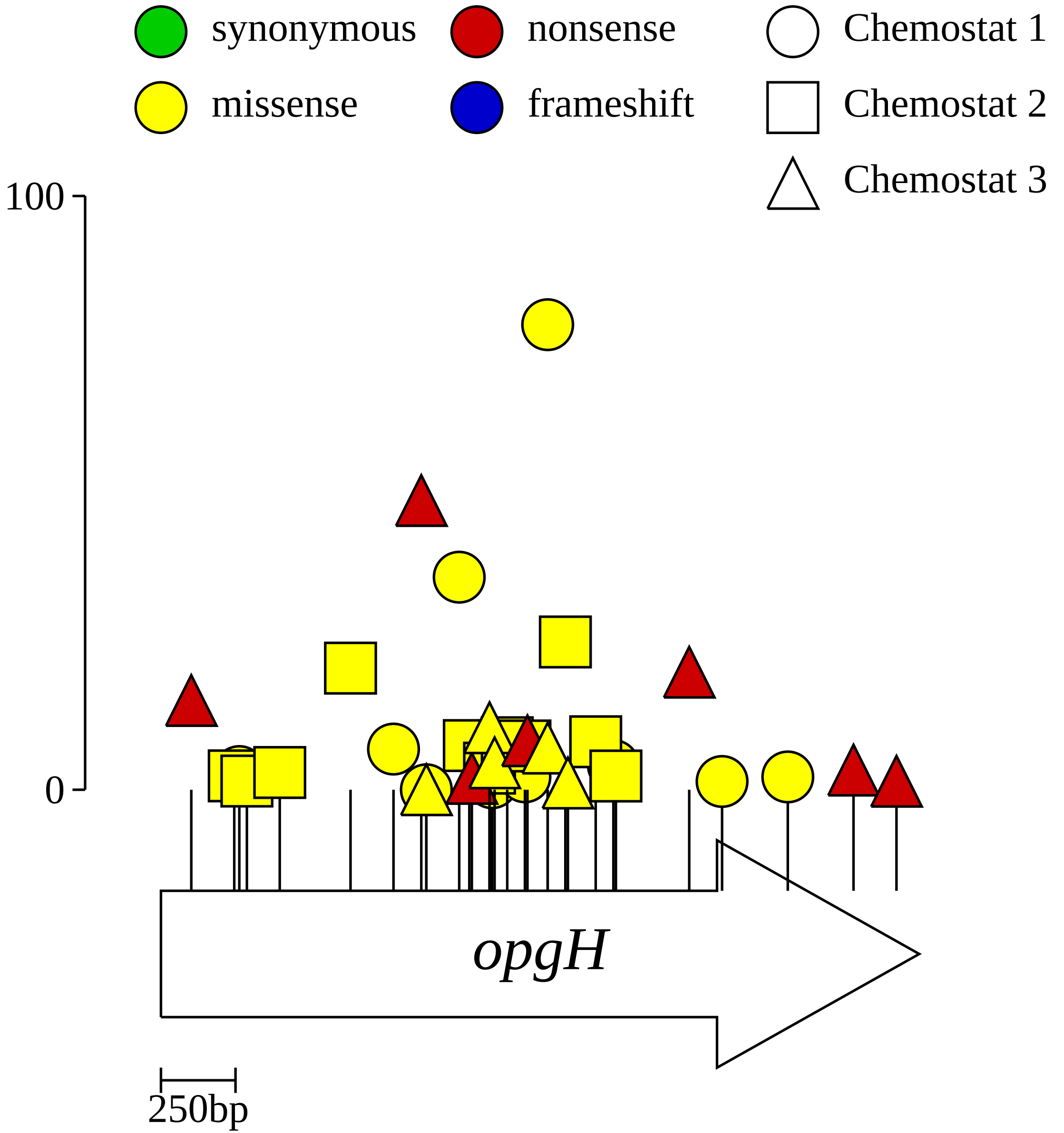
synonymous
nonsense
Chemostat 1
missense
frameshift
Chemostat 2
Chemostat 3
100
0
opgH
250bp

Figure S8

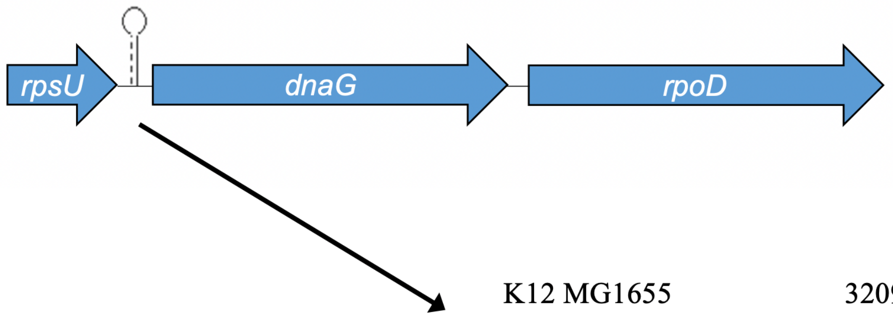

T_1 variant/mutation	ΔG (kcal/mol)
K12 MG1655 (wt)	-23.6
bp 3209075 C→A	-14.3
bp 3209076 C→A	-16.7
bp 3209081 G→T	-16.1
bp 3209082 G→T	-14.3
bp 3209091 G→T	-16.1

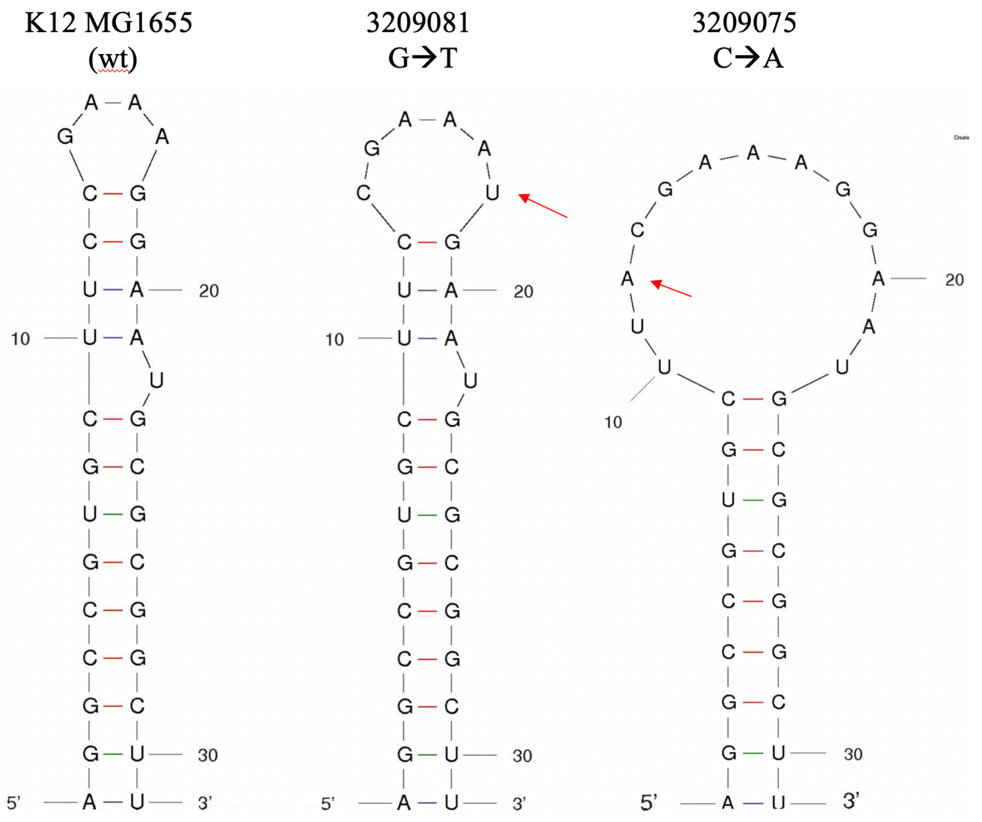

Supplement: Supplementary file 1 — Additional file 1 Fig. S1: Cell density and residual metabolite concentrations. Fig. S2: Input of de novo mutations. Fig. S3: Most de novo mutations only reach low allele frequencies and experience pervasive clonal interference. (A) Histogram of maximum frequencies; (B) Final vs. maximum frequencies; (C) Venn diagram showing degree of genic parallelism among beneficial mutations. Fig. S4: Isolated clones are representative of the populations from which they are drawn. Fig. S5: MalK/MalT population dynamics. Fig. S6: Mutations in glucosyltransferase opgH occur repeatedly and, collectively, go to high frequency. Fig. S7: opgH has nonsense and missense mutations throughout its length. Fig. S8: Mutations that decrease T1 terminator stability in the macromolecular synthesis operon are expected to affect expression of dnaG (DNA primase) and rpoD (housekeeping σ-factor). Predicted ΔG values for wild-type T1 terminator from E. coli K12 MG1655 and two variants observed in chemostat 1 were determined using unafold.rna.albany.edu/?q=mfold/RNA-Folding-Form. The C➔A mutation at nucleotide 3,209,075 has been previously observed in chemostat-evolved E. coli [34]. [file 12915_2021_954_MOESM1_ESM.pdf]
