# Supplementary material for: Evolutionary dynamics and structural consequences of de novo beneficial mutations and mutant lineages arising in a constant environment
Source: BMC Biol. 2021 Feb 4;19:20. doi: 10.1186/s12915-021-00954-0 (PMC7863352; doi:10.1186/s12915-021-00954-0)
Supplement: Supplementary file 2 — Additional file 2 Table S1. Key mutations that distinguish ancestral strain JA122 from K12 (MG1655). Table S2. Beneficial alleles. Table S3. Population allele frequencies for frequently mutated genes. Table S4. Identical mutations arise within and among replicate evolution experiments. Table S5. Fixed alleles among replicate populations (“fixed” defined as > 98% at any time point between generation 50 and 500). [file 12915_2021_954_MOESM2_ESM.pdf]

Table S1. Key mutations that distinguish ancestral strain JA122 from K12 (MG1655)

Gene	Amino acid change	Phenotype
*mutY (*adenine glycosylase)	Leu299* (ochre)	Increased rate of GC→TA transversions
rpoD (“exponential phase” sigma factor RpoD/σ_{70})	Glu26* (amber)	Unknown
rpoS (“stationary phase” sigma factor RpoS/σ_{38})	Gln33*	Largely unknown but involves suppression of “stationary-phase” gene transcription
glnX amber suppressor; reported in parent of JA122, RH101	N/A	Allows translational read-through of amber, opal and ochre nonsense codons
fimH (fimbrial adhesion)	Pro33His	unknown
fimE (recombinase, catalyzes *fim* operon switch inversion in the on-to-off direction)	IS1 insertion +8bp target site duplication	Unknown but recombinase likely inactive
lsrR (transcriptional repressor related to quorum sensing, biofilm formation, sRNA production)	A251S	Unknown; deletion upregulates sRNAs DsrA and DicF among others
acs (acetyl-CoA synthetase (AMP-forming) A→T, position -93 from START in CRP binding site	N/A	Dysregulation of the *acs-yjcG* operon, resulting in diminished capacity to scavenge limiting acetate

Table S2 Beneficial alleles

Rank	Gene	Expected	P-Value	Observed	Length	-LOG10 I	NUM_EX	FDR	TP	FN
1	galS***	0.778336	6.55E-50	38	1041	49.18344	4.42E-47	4.42E-45	1.00	0.00
2	hfq*******	0.231034	6.91E-40	24	309	39.16081	4.66E-37	2.33E-35	2.00	0.00
3	pgi*******	1.233675	4.54E-38	35	1650	37.34286	3.07E-35	1.02E-33	3.00	0.00
4	opgH**	1.902102	8.74E-27	31	2544	26.05829	5.90E-24	1.48E-22	4.00	0.00
5	malT*****	2.023226	8.10E-25	30	2706	24.09176	5.46E-22	1.09E-20	5.00	0.00
6	malK*****	0.834413	7.47E-24	22	1116	23.12675	5.04E-21	8.40E-20	6.00	0.00
7	upstream m	0.209351	2.91E-09	7	280	8.535746	1.97E-06	2.81E-05	7.00	0.00
8	rho**	0.942079	5.49E-09	11	1260	8.260065	3.71E-06	4.64E-05	8.00	0.00
9	upstream dı	0.082993	3.06E-08	5	111	7.513989	2.07E-05	0.00023	9.00	0.00
10	fimH***	0.675156	4.38E-08	9	903	7.358184	2.96E-05	0.000296	10.00	0.00
11	rpoS*	0.742448	9.71E-08	9	993	7.012954	6.55E-05	0.000596	11.00	0.00
12	upstream rl	0.124115	2.21E-07	5	166	6.65491	0.000149	0.001245	12.00	0.00
13	gatZ	0.944322	7.06E-07	9	1263	6.151132	0.000477	0.003666	13.00	0.00
14	pfkA*	0.720017	9.47E-07	8	963	6.023582	0.000639	0.004567	14.00	0.00
15	rpoA*	0.740205	1.27E-05	7	990	4.89675	0.008562	0.057077	14.99	0.01
16	lptD*	1.76079	1.61E-05	10	2355	4.792867	0.010875	0.06797	15.99	0.01
17	proQ	0.522629	1.81E-05	6	699	4.741786	0.012233	0.071956	16.99	0.01
18	lptG	0.809739	2.24E-05	7	1083	4.649919	0.015114	0.083968	17.98	0.02
19	downstrean	0.056824	2.93E-05	3	76	4.533043	0.019782	0.104114	18.98	0.02
20	yciM*	0.874787	3.64E-05	7	1170	4.439428	0.02454	0.1227	19.98	0.02
21	downstrean	0.064301	4.22E-05	3	86	4.374416	0.028503	0.135728	20.97	0.03
22	slt*	1.449007	0.000134	8	1938	3.871502	0.090741	0.412457	21.91	0.09
23	gabD*	1.083391	0.000136	7	1449	3.867359	0.09161	0.398306	22.91	0.09
24	wzzE	0.782823	0.000164	6	1047	3.784636	0.110833	0.461802	23.89	0.11
25	yobF*	0.107666	0.000192	3	144	3.716888	0.129544	0.518174	24.87	0.13
26	opgG*	1.148439	0.000193	7	1536	3.714401	0.130287	0.501105	25.87	0.13
27	fliH	0.513657	0.000195	5	687	3.710543	0.13145	0.486852	26.87	0.13
28	ompR	0.538331	0.000241	5	720	3.617477	0.162865	0.581659	27.84	0.16
29	malE	0.890489	0.000325	6	1191	3.488285	0.21929	0.756173	28.78	0.22
30	ybaL*	1.253862	0.000326	7	1677	3.486737	0.220073	0.733576	29.78	0.22
31	upstream ac	0.356644	0.000508	4	477	3.29443	0.342668	1.10538	30.66	0.34
32	yiaO	0.737962	0.000992	5	987	3.003644	0.669359	2.091748	31.33	0.67
33	lptC	0.430665	0.001018	4	576	2.992182	0.687261	2.082609	32.31	0.69
34	fliG*	0.744691	0.001032	5	996	2.986323	0.696595	2.04881	33.30	0.70
35	lpxD	0.767121	0.001175	5	1026	2.929845	0.793339	2.266681	34.21	0.79
36	valY tRNA	0.056824	0.001555	2	76	2.808383	1.049352	2.914866	34.95	1.05
37	fliP	0.551789	0.002495	4	738	2.603011	1.683809	4.550836	35.32	1.68
38	hyaB	1.341341	0.0026	6	1794	2.584989	1.755154	4.618827	36.24	1.76
39	glpR	0.56749	0.002757	4	759	2.559618	1.860742	4.771133	37.14	1.86
40	upstream m	0.085983	0.003491	2	115	2.457006	2.356663	5.891658	37.64	2.36
41	ydgI*	1.034044	0.004209	5	1383	2.375839	2.840958	6.929167	38.16	2.84
42	rbsB	0.666184	0.004846	4	891	2.314621	3.27102	7.788143	38.73	3.27
43	ytfR	1.123765	0.005933	5	1503	2.226713	4.004888	9.313694	39.00	4.00
44	yphE	1.130494	0.00608	5	1512	2.216113	4.103845	9.32692	39.90	4.10
45	lpxM	0.726746	0.006547	4	972	2.183989	4.418901	9.819781	40.58	4.42
46	ydcT	0.758149	0.007566	4	1014	2.121125	5.107146	11.10249	40.89	5.11
47	phnD	0.760392	0.007643	4	1017	2.116752	5.15884	10.97625	41.84	5.16
48	dppA	1.202272	0.007805	5	1608	2.107623	5.26842	10.97588	42.73	5.27
49	pgsA	0.410477	0.0085	3	549	2.070582	5.737489	11.70916	43.26	5.74
50	sgrR	1.238161	0.008784	5	1656	2.056323	5.928996	11.85799	44.07	5.93
51	lptA	0.417206	0.008881	3	558	2.051541	5.994632	11.75418	45.01	5.99
52	recF	0.80301	0.009196	4	1074	2.036413	6.207128	11.93679	45.79	6.21
53	yohD	0.432908	0.009808	3	579	2.00841	6.620548	12.4916	46.38	6.62
54	downstrean	0.15477	0.01081	2	207	1.966176	7.296728	13.51246	46.70	7.30
55	cspE	0.157013	0.011109	2	210	1.954319	7.498686	13.63397	47.50	7.50
56	flhB	0.859086	0.011533	4	1149	1.938054	7.784847	13.90151	48.22	7.78
57	nagA	0.859086	0.011533	4	1149	1.938054	7.784847	13.65763	49.22	7.78
58	dgoD	0.859086	0.011533	4	1149	1.938054	7.784847	13.42215	50.22	7.78
59	yqgC	0.161499	0.011718	2	216	1.931131	7.90993	13.40666	51.09	7.91

60	ydhP	0.874787	0.01225	4	1170	1.911876	8.268523	13.78087	51.73	8.27
61	yhdV	0.165985	0.012342	2	222	1.908614	8.330866	13.65716	52.67	8.33
62	sspA	0.477768	0.012758	3	639	1.89421	8.611802	13.89	53.39	8.61
63	emrD	0.886003	0.012779	4	1185	1.893519	8.625505	13.69128	54.37	8.63
64	yfaY	0.899461	0.013432	4	1203	1.871856	9.066671	14.16667	54.93	9.07
65	secD	1.381715	0.013544	5	1848	1.868261	9.142042	14.06468	55.86	9.14
66	asmA	1.386202	0.013716	5	1854	1.862785	9.258029	14.02732	56.74	9.26
67	ycjU	0.49347	0.013897	3	660	1.857066	9.380764	14.00114	57.62	9.38
68	ydhI	0.177201	0.013963	2	237	1.855023	9.424986	13.86027	58.58	9.42
69	upstream yj	0.189163	0.015787	2	253	1.801689	10.65651	15.44422	58.34	10.66
70	tolA	0.946565	0.015885	4	1266	1.799005	10.72258	15.31797	59.28	10.72
71	efeB	0.951051	0.016133	4	1272	1.792296	10.88949	15.33731	60.11	10.89
72	upstream d	0.192902	0.016377	2	258	1.785755	11.05474	15.3538	60.95	11.05
73	ndh*	0.975724	0.017536	4	1305	1.756076	11.83661	16.21454	61.16	11.84
74	lptB*	0.542817	0.017843	3	726	1.748526	12.04418	16.27592	61.96	12.04
75	menE	1.013856	0.01985	4	1356	1.70225	13.39843	17.86457	61.60	13.40
76	upstream p	0.214585	0.01998	2	287	1.699401	13.48662	17.74555	62.51	13.49
77	yedV	1.016099	0.019991	4	1359	1.699161	13.49405	17.52474	63.51	13.49
78	aroD	0.56749	0.020026	3	759	1.698414	13.51729	17.32986	64.48	13.52
79	hyi	0.580949	0.021275	3	777	1.672128	14.3607	18.1781	64.64	14.36
80	flgG	0.585435	0.021701	3	783	1.663523	14.6481	18.31012	65.35	14.65
81	fhuF	0.589921	0.022131	3	789	1.654993	14.93863	18.44275	66.06	14.94
82	mdtD	1.058717	0.022802	4	1416	1.642034	15.3911	18.76963	66.61	15.39
83	upstream p	0.231034	0.022913	2	309	1.63992	15.46619	18.63397	67.53	15.47
84	asr	0.231034	0.022913	2	309	1.63992	15.46619	18.41213	68.53	15.47
85	ptrA	2.160052	0.023057	6	2889	1.637201	15.56333	18.3098	69.44	15.56
86	qseE	1.067689	0.023422	4	1428	1.630371	15.81004	18.38377	70.19	15.81
87	hsrA	1.067689	0.023422	4	1428	1.630371	15.81004	18.17246	71.19	15.81
88	yggL	0.244492	0.025435	2	327	1.59456	17.16895	19.51017	70.83	17.17
89	yjfO	0.246735	0.025866	2	330	1.587264	17.45984	19.6178	71.54	17.46
90	wzxC	1.105821	0.026174	4	1479	1.582133	17.66732	19.63036	72.33	17.67
91	gspE	1.108064	0.026341	4	1482	1.579361	17.78046	19.53897	73.22	17.78
92	gatY	0.639268	0.027172	3	855	1.56588	18.34104	19.93592	73.66	18.34
93	cdsA	0.641511	0.027414	3	858	1.562022	18.50468	19.89751	74.50	18.50
94	relA	1.671068	0.027811	5	2235	1.555778	18.77267	19.97092	75.23	18.77
95	cysW*	0.654969	0.028893	3	876	1.539206	19.50285	20.52932	75.50	19.50
96	upstream y	0.268418	0.030183	2	359	1.520243	20.37326	21.22214	75.63	20.37
97	yfgD	0.269165	0.030336	2	360	1.518039	20.47694	21.11025	76.52	20.48
98	ygfK	2.317065	0.030903	6	3099	1.510005	20.85924	21.28494	77.14	20.86
99	putA	2.963062	0.031681	7	3963	1.499205	21.38451	21.60051	77.62	21.38
100	lpxC	0.686372	0.032505	3	918	1.488056	21.94055	21.94055	78.06	21.94
101	adrB	1.195543	0.033384	4	1599	1.476467	22.53394	22.31084	78.47	22.53
102	rihA	0.69983	0.034121	3	936	1.46698	23.0316	22.58	78.97	23.03
103	flgJ	0.704316	0.034669	3	942	1.460061	23.40143	22.71984	79.60	23.40
104	aidB	1.21573	0.03515	4	1626	1.45408	23.72596	22.81342	80.27	23.73
105	yqeB	1.21573	0.03515	4	1626	1.45408	23.72596	22.59615	81.27	23.73
106	downstrean	0.711045	0.035499	3	951	1.449782	23.96192	22.60558	82.04	23.96
107	ygiT	0.296082	0.03607	2	396	1.44286	24.34693	22.75414	82.65	24.35
108	fumA	1.231431	0.03656	4	1647	1.436996	24.67786	22.84987	83.32	24.68
109	upstream n	0.299073	0.03673	2	400	1.434974	24.79304	22.74591	84.21	24.79
110	hslR	0.300568	0.037063	2	402	1.431064	25.01727	22.74297	84.98	25.02
111	rnk	0.307297	0.038572	2	411	1.41373	26.03596	23.45582	84.96	26.04
112	dusA	0.742448	0.039508	3	993	1.40331	26.66818	23.81088	85.33	26.67
113	fruR	0.75142	0.040694	3	1005	1.390466	27.46866	24.30854	85.53	27.47
114	rhsC	3.135776	0.040824	7	4194	1.389082	27.55633	24.17222	86.44	27.56
115	upstream yj	0.321503	0.041833	2	430	1.37848	28.23734	24.5542	86.76	28.24
116	upstream m	0.325241	0.042708	2	435	1.369491	28.82788	24.85162	87.17	28.83
117	tdh	0.767121	0.042812	3	1026	1.36843	28.89838	24.69947	88.10	28.90
118	mdtN	0.771607	0.043428	3	1032	1.362235	29.3136	24.84203	88.69	29.31
119	yagN*	0.329728	0.043767	2	441	1.358855	29.5426	24.82571	89.46	29.54
120	yhbP	0.331971	0.0443	2	444	1.353597	29.90242	24.91868	90.10	29.90

121	tas	0.778336	0.044359	3	1041	1.353023	29.942	24.74545	91.06	29.94
122	yejM	1.316667	0.044777	4	1761	1.348947	30.22436	24.77406	91.78	30.22
123	fbaB	0.787309	0.045615	3	1053	1.340891	30.79024	25.03272	92.21	30.79
124	phnG	0.3387	0.045913	2	453	1.33806	30.99161	24.99323	93.01	30.99
125	matP	0.3387	0.045913	2	453	1.33806	30.99161	24.79329	94.01	30.99
126	yfjX	0.343186	0.047001	2	459	1.327892	31.72576	25.17917	94.27	31.73
127	pyrI	0.345429	0.047548	2	462	1.322864	32.09521	25.27182	94.90	32.10
128	narX	1.343584	0.047569	4	1797	1.32268	32.10879	25.08499	95.89	32.11
129	ulaC	0.347672	0.048098	2	465	1.317872	32.46623	25.16762	96.53	32.47
130	yiaL	0.349915	0.04865	2	468	1.312916	32.83882	25.26063	97.16	32.84
131	ybiC	0.811982	0.049161	3	1086	1.30838	33.1836	25.331	97.82	33.18
132	recD	1.366014	0.049967	4	1827	1.301317	33.72774	25.55132	98.27	33.73
133	anmK	0.829926	0.051822	3	1110	1.285489	34.97957	26.30043	98.02	34.98
134	hypD	0.838899	0.053178	3	1122	1.274272	35.89485	26.7872	98.11	35.89
135	yhhJ	0.841142	0.053519	3	1125	1.27149	36.12545	26.7596	98.87	36.13
136	yfdN	0.370102	0.05372	2	495	1.269863	36.26109	26.66256	99.74	36.26
137	yebR	0.372345	0.054295	2	498	1.265244	36.64884	26.75098	100.35	36.65
138	yafM	0.372345	0.054295	2	498	1.265244	36.64884	26.55713	101.35	36.65
139	recX	0.374588	0.054871	2	501	1.260655	37.03806	26.64609	101.96	37.04
140	phnM	0.850114	0.054896	3	1137	1.260457	37.05501	26.46787	102.94	37.06
141	downstrean	0.375336	0.055064	2	502	1.259133	37.16813	26.36038	103.83	37.17
142	ydeR	0.376831	0.05545	2	504	1.256098	37.42875	26.35827	104.57	37.43
143	mug	0.379075	0.056031	2	507	1.251572	37.82088	26.44817	105.18	37.82
144	yheS*	1.431062	0.05729	4	1914	1.241918	38.67104	26.85489	105.33	38.67
145	ygbK	0.872544	0.058412	3	1167	1.233494	39.42841	27.19201	105.57	39.43
146	yhhZ	0.881517	0.059848	3	1179	1.222951	40.39738	27.66944	105.60	40.40
147	tsgA	0.88376	0.060209	3	1182	1.220336	40.64136	27.64719	106.36	40.64
148	tufA	0.886003	0.060572	3	1185	1.217729	40.88603	27.6257	107.11	40.89
149	ybjK	0.401505	0.061956	2	537	1.207917	41.82024	28.06728	107.18	41.82
150	yagF	1.471437	0.062109	4	1968	1.206846	41.92349	27.94899	108.08	41.92
151	dnaT	0.403748	0.06256	2	540	1.203705	42.2278	27.96543	108.77	42.23
152	dacC	0.899461	0.062768	3	1203	1.20226	42.36853	27.87403	109.63	42.37
153	aegA	1.480409	0.063208	4	1980	1.19923	42.66524	27.88578	110.33	42.67
154	mdtH	0.903947	0.063508	3	1209	1.197169	42.86818	27.83648	111.13	42.87
155	hyfH	0.408234	0.063773	2	546	1.195361	43.04695	27.77223	111.95	43.05
156	dgsA	0.912919	0.065001	3	1221	1.18708	43.87564	28.12541	112.12	43.88
157	phnN	0.417206	0.066224	2	558	1.178984	44.70124	28.47213	112.30	44.70
158	hyfB	1.509569	0.066849	4	2019	1.174904	45.12321	28.559	112.88	45.12
159	cca*	0.926377	0.06727	3	1239	1.17218	45.4071	28.55792	113.59	45.41
160	ytfN	2.826236	0.067422	6	3780	1.171198	45.50988	28.44367	114.49	45.51
161	spr	0.423935	0.068082	2	567	1.166965	45.95567	28.54389	115.04	45.96
162	yfeO	0.939836	0.069574	3	1257	1.157551	46.96263	28.98928	115.04	46.96
163	yjeH*	0.939836	0.069574	3	1257	1.157551	46.96263	28.81143	116.04	46.96
164	nfuA	0.430665	0.069958	2	576	1.155163	47.22163	28.79367	116.78	47.22
165	rpoE	0.430665	0.069958	2	576	1.155163	47.22163	28.61917	117.78	47.22
166	upstream m	0.43216	0.070377	2	578	1.152569	47.50449	28.61716	118.50	47.50
167	lhgO	0.948808	0.07113	3	1269	1.147946	48.01289	28.75023	118.99	48.01
168	gabT	0.95778	0.072702	3	1281	1.138456	49.07364	29.2105	118.93	49.07
169	yeaH	0.960023	0.073097	3	1284	1.136101	49.34046	29.19554	119.66	49.34
170	yajL	0.44188	0.073121	2	591	1.135959	49.35659	29.03329	120.64	49.36
171	pinR	0.44188	0.073121	2	591	1.135959	49.35659	28.8635	121.64	49.36
172	ydiS	0.964509	0.07389	3	1290	1.131412	49.87604	28.9977	122.12	49.88
173	ynbD	0.966752	0.074289	3	1293	1.129078	50.1448	28.98543	122.86	50.14
174	upstream p	0.447114	0.074612	2	598	1.127189	50.36339	28.94448	123.64	50.36
175	upstream h	0.452347	0.076114	2	605	1.118537	51.37673	29.35813	123.62	51.38
176	yrfF	1.597048	0.078407	4	2136	1.105645	52.9248	30.07091	123.08	52.92
177	rhtB	0.46431	0.079581	2	621	1.099192	53.71696	30.34856	123.28	53.72
178	fdhF	1.60602	0.079645	4	2148	1.098839	53.76063	30.2026	124.24	53.76
179	yaiV	0.466553	0.080236	2	624	1.09563	54.15941	30.25665	124.84	54.16
180	gudX	1.002641	0.080788	3	1341	1.092654	54.53177	30.29543	125.47	54.53
181	ydhY	0.468796	0.080893	2	627	1.092087	54.60299	30.1674	126.40	54.60

182	yagK	0.468796	0.080893	2	627	1.092087	54.60299	30.00164	127.40	54.60
183	fdnG	2.278933	0.081307	5	3048	1.089871	54.88235	29.99035	128.12	54.88
184	nudF	0.471039	0.081552	2	630	1.088565	55.04771	29.91723	128.95	55.05
185	upstream ya	0.471787	0.081772	2	631	1.087395	55.19619	29.83578	129.80	55.20
186	dhaL	0.473282	0.082213	2	633	1.085061	55.49354	29.83524	130.51	55.49
187	ftsK	2.983249	0.082239	6	3990	1.084924	55.51105	29.68505	131.49	55.51
188	yfdX	0.475525	0.082875	2	636	1.081578	55.94049	29.75558	132.06	55.94
189	argO	0.475525	0.082875	2	636	1.081578	55.94049	29.59814	133.06	55.94
190	hcaE	1.018342	0.083706	3	1362	1.077243	56.50156	29.73766	133.50	56.50
191	eda	0.480012	0.084204	2	642	1.074667	56.8377	29.75796	134.16	56.84
192	adk	0.482255	0.084871	2	645	1.07124	57.28795	29.83748	134.71	57.29
193	cheZ	0.482255	0.084871	2	645	1.07124	57.28795	29.68288	135.71	57.29
194	aroP	1.027314	0.085394	3	1374	1.068575	57.64067	29.71169	136.36	57.64
195	fabR	0.484498	0.08554	2	648	1.067832	57.73929	29.60989	137.26	57.74
196	rcsB	0.486741	0.08621	2	651	1.064443	58.1917	29.68964	137.81	58.19
197	elbB	0.488984	0.086882	2	654	1.061071	58.64519	29.76913	138.35	58.65
198	hyuA	1.036287	0.087096	3	1386	1.060004	58.7895	29.69167	139.21	58.79
199	sad	1.03853	0.087523	3	1389	1.057876	59.07822	29.68755	139.92	59.08
200	upstream yj	0.491227	0.087555	2	657	1.057718	59.09974	29.54987	140.90	59.10
201	ybiO	1.664339	0.087928	4	2226	1.055872	59.35157	29.52814	141.65	59.35
202	xanP	1.040773	0.087952	3	1392	1.055755	59.36754	29.38987	142.63	59.37
203	pcnB	1.045259	0.088812	3	1398	1.051529	59.94796	29.53102	143.05	59.95
204	yedK	0.500199	0.090264	2	669	1.044484	60.92843	29.86688	143.07	60.93
205	upstream yo	0.500947	0.090491	2	670	1.043394	61.08158	29.79589	143.92	61.08
206	ybiH	0.502442	0.090945	2	672	1.041219	61.3882	29.8001	144.61	61.39
207	yiiM	0.504685	0.091628	2	675	1.037971	61.84899	29.87874	145.15	61.85
208	treB	1.063203	0.092286	3	1422	1.034862	62.29338	29.94874	145.71	62.29
209	lpd	1.065446	0.092725	3	1425	1.032804	62.5892	29.94699	146.41	62.59
210	narV	0.509171	0.092998	2	681	1.031527	62.7736	29.89219	147.23	62.77
211	rnc	0.509171	0.092998	2	681	1.031527	62.7736	29.75052	148.23	62.77
212	upstream ef	1.069185	0.093457	3	1430	1.029388	63.08353	29.75638	148.92	63.08
213	cmk	0.511414	0.093685	2	684	1.02833	63.23742	29.68893	149.76	63.24
214	ydiU	1.074418	0.094486	3	1437	1.024631	63.7783	29.80294	150.22	63.78
215	bglA	1.076661	0.094929	3	1440	1.022601	64.07702	29.80327	150.92	64.08
216	aldA	1.076661	0.094929	3	1440	1.022601	64.07702	29.66529	151.92	64.08
217	paoA	0.5159	0.095064	2	690	1.021985	64.16802	29.57052	152.83	64.17
218	modB	0.5159	0.095064	2	690	1.021985	64.16802	29.43487	153.83	64.17
219	nanE*	0.5159	0.095064	2	690	1.021985	64.16802	29.30047	154.83	64.17
220	mutH	0.5159	0.095064	2	690	1.021985	64.16802	29.16728	155.83	64.17
221	yieP	0.518143	0.095755	2	693	1.018837	64.6348	29.24652	156.37	64.63
222	torR	0.518143	0.095755	2	693	1.018837	64.6348	29.11477	157.37	64.63
223	napG	0.520386	0.096448	2	696	1.015706	65.10255	29.19397	157.90	65.10
224	frvB	1.085634	0.096708	3	1452	1.014539	65.27763	29.1418	158.72	65.28
225	yjfJ	0.522629	0.097143	2	699	1.01259	65.57127	29.14279	159.43	65.57
226	yhgF	1.736116	0.098666	4	2322	1.005832	66.5996	29.46885	159.40	66.60
227	upstream th	0.527863	0.098768	2	706	1.005382	66.66868	29.36946	160.33	66.67
228	guaB	1.096849	0.09895	3	1467	1.004585	66.7912	29.29439	161.21	66.79
229	upstream gl	0.530106	0.099468	2	709	1.002319	67.14058	29.31903	161.86	67.14
230	modF	1.101335	0.099853	3	1473	1.00064	67.40057	29.30459	162.60	67.40
231	ispD	0.531602	0.099934	2	711	1.000285	67.4557	29.2016	163.54	67.46
232	ampG	1.103578	0.100305	3	1476	0.998676	67.70609	29.18366	164.29	67.71
233	sanA	0.538331	0.102043	2	720	0.991218	68.87886	29.56174	164.12	68.88
234	puuC	1.11255	0.102124	3	1488	0.990872	68.93374	29.45886	165.07	68.93
235	amyA	1.11255	0.102124	3	1488	0.990872	68.93374	29.33351	166.07	68.93
236	baeR	0.540574	0.102748	2	723	0.988225	69.35509	29.38775	166.64	69.36
237	yafS	0.540574	0.102748	2	723	0.988225	69.35509	29.26375	167.64	69.36
238	pyrH	0.542817	0.103455	2	726	0.985248	69.83223	29.34127	168.17	69.83
239	yfiH	0.547303	0.104873	2	732	0.979337	70.7892	29.61891	168.21	70.79
240	putP	1.128251	0.105338	3	1509	0.977414	71.10328	29.62637	168.90	71.10
241	rhsD	3.200825	0.105502	6	4281	0.976739	71.21393	29.54935	169.79	71.21
242	pyrF	0.551789	0.106296	2	738	0.973484	71.74972	29.64865	170.25	71.75

243	yjbG	0.551789	0.106296	2	738	0.973484	71.74972	29.52663	171.25	71.75
244	yifB	1.137224	0.107193	3	1521	0.969836	72.35494	29.65367	171.65	72.35
245	yacF	0.556275	0.107724	2	744	0.967687	72.71376	29.67908	172.29	72.71
246	wcaE	0.558518	0.10844	2	747	0.96481	73.19707	29.75491	172.80	73.20
247	emrB	1.150682	0.109998	3	1539	0.958616	74.24844	30.0601	172.75	74.25
248	agaI	0.565247	0.110596	2	756	0.956261	74.65214	30.10167	173.35	74.65
249	znuC	0.565247	0.110596	2	756	0.956261	74.65214	29.98078	174.35	74.65
250	yagI	0.56749	0.111317	2	759	0.953439	75.13885	30.05554	174.86	75.14
251	phnK	0.56749	0.111317	2	759	0.953439	75.13885	29.9358	175.86	75.14
252	yjjI	1.159654	0.111883	3	1551	0.951234	75.52129	29.96876	176.48	75.52
253	ydiQ	0.571976	0.112763	2	765	0.947835	76.11476	30.08489	176.89	76.11
254	thiG	0.576462	0.114213	2	771	0.942283	77.09396	30.35195	176.91	77.09
255	srlR	0.578705	0.11494	2	774	0.939527	77.58477	30.4254	177.42	77.58
256	uxuR	0.578705	0.11494	2	774	0.939527	77.58477	30.30655	178.42	77.58
257	garD	1.175355	0.115213	3	1572	0.938499	77.76869	30.26019	179.23	77.77
258	ygfM	0.583192	0.116398	2	780	0.934054	78.56879	30.45302	179.43	78.57
259	ylbA	0.587678	0.117861	2	786	0.928631	79.55598	30.71659	179.44	79.56
260	ycjR	0.589921	0.118594	2	789	0.925938	80.05074	30.78875	179.95	80.05
261	nanR	0.592164	0.119328	2	792	0.923258	80.54627	30.86064	180.45	80.55
262	ssuC	0.592164	0.119328	2	792	0.923258	80.54627	30.74285	181.45	80.55
263	agaD	0.592164	0.119328	2	792	0.923258	80.54627	30.62596	182.45	80.55
264	otsB	0.598893	0.121537	2	801	0.915292	82.03745	31.07479	181.96	82.04
265	bcsC	2.597446	0.122216	5	3474	0.912874	82.49547	31.13037	182.50	82.50
266	suhB	0.601136	0.122276	2	804	0.91266	82.53601	31.02858	183.46	82.54
267	rna	0.603379	0.123015	2	807	0.910041	83.03532	31.09937	183.96	83.04
268	nikE	0.603379	0.123015	2	807	0.910041	83.03532	30.98333	184.96	83.04
269	mhpD	0.605622	0.123756	2	810	0.907433	83.53536	31.05404	185.46	83.54
270	yaeI	0.607865	0.124498	2	813	0.904838	84.03613	31.12449	185.96	84.04
271	yegX	0.612351	0.125985	2	819	0.899681	85.03985	31.38002	185.96	85.04
272	ybhA	0.612351	0.125985	2	819	0.899681	85.03985	31.26465	186.96	85.04
273	rsmA	0.614594	0.12673	2	822	0.89712	85.54278	31.33435	187.46	85.54
274	upstream e:	1.229936	0.127069	3	1645	0.895961	85.77137	31.30342	188.23	85.77
275	mqo	1.231431	0.127399	3	1647	0.894832	85.99465	31.27078	189.01	85.99
276	gadX	0.616837	0.127476	2	825	0.894571	86.04642	31.17624	189.95	86.05
277	tauC	0.61908	0.128223	2	828	0.892033	86.55076	31.24576	190.45	86.55
278	ushA*	1.235918	0.128394	3	1653	0.891456	86.66571	31.17472	191.33	86.67
279	glpG	0.621323	0.128972	2	831	0.889506	87.0558	31.2028	191.94	87.06
280	fliF	1.240404	0.129391	3	1659	0.888097	87.33864	31.19237	192.66	87.34
281	nrfE	1.240404	0.129391	3	1659	0.888097	87.33864	31.08137	193.66	87.34
282	csgG	0.623566	0.129721	2	834	0.88699	87.56153	31.05019	194.44	87.56
283	prmC	0.623566	0.129721	2	834	0.88699	87.56153	30.94047	195.44	87.56
284	asnB	1.24489	0.13039	3	1665	0.884755	88.01342	30.99064	195.99	88.01
285	hdfR	0.628052	0.131222	2	840	0.881992	88.57504	31.07896	196.42	88.58
286	purU	0.630296	0.131975	2	843	0.87951	89.08281	31.14783	196.92	89.08
287	ligB	1.258348	0.133406	3	1683	0.874826	90.04874	31.37587	196.95	90.05
288	panC	0.637025	0.134237	2	852	0.872127	90.6101	31.46184	197.39	90.61
289	upstream yi	0.637772	0.134489	2	853	0.871313	90.78017	31.41182	198.22	90.78
290	fruA	1.265077	0.134922	3	1692	0.869917	91.0725	31.40431	198.93	91.07
291	ampE	0.639268	0.134993	2	855	0.869688	91.12051	31.31289	199.88	91.12
292	ybbN	0.639268	0.134993	2	855	0.869688	91.12051	31.20565	200.88	91.12
293	ydaU	0.641511	0.13575	2	858	0.867259	91.63157	31.27357	201.37	91.63
294	ybaE	1.271806	0.136445	3	1701	0.865043	92.10027	31.32662	201.90	92.10
295	yfjP	0.645997	0.137268	2	864	0.862432	92.65559	31.40868	202.34	92.66
296	upstream pj	0.645997	0.137268	2	864	0.862432	92.65559	31.30257	203.34	92.66
297	yebT	1.969393	0.137396	4	2634	0.862025	92.74251	31.22643	204.26	92.74
298	upstream zu	1.279283	0.138144	3	1711	0.859669	93.24689	31.2909	204.75	93.25
299	rluF	0.652726	0.13955	2	873	0.85527	94.19635	31.5038	204.80	94.20
300	cvrA	1.298723	0.142593	3	1737	0.8459	96.2506	32.08353	203.75	96.25
301	yghA	0.661698	0.142606	2	885	0.845861	96.25926	31.97982	204.74	96.26
302	adhE	2.000796	0.14302	4	2676	0.844603	96.53861	31.96643	205.46	96.54
303	arnD	0.666184	0.14414	2	891	0.841216	97.29429	32.11033	205.71	97.29

304	malG	0.666184	0.14414	2	891	0.841216	97.29429	32.0047	206.71	97.29
305	argP	0.668427	0.144908	2	894	0.838909	97.81269	32.06973	207.19	97.81
306	ilvY	0.668427	0.144908	2	894	0.838909	97.81269	31.96493	208.19	97.81
307	yfeX	0.672913	0.146446	2	900	0.834322	98.8512	32.19909	208.15	98.85
308	ompG	0.677399	0.147988	2	906	0.829773	99.89198	32.43246	208.11	99.89
309	nhaR	0.677399	0.147988	2	906	0.829773	99.89198	32.3275	209.11	99.89
310	gltI	0.679643	0.14876	2	909	0.827513	100.4132	32.39136	209.59	100.41
311	fucI	1.327882	0.149356	3	1776	0.825777	100.8153	32.41649	210.18	100.82
312	upstream pl	0.683381	0.150049	2	914	0.823767	101.2831	32.46254	210.72	101.28
313	gcl	1.332368	0.150405	3	1782	0.822737	101.5237	32.43567	211.48	101.52
314	oxyR	0.686372	0.151082	2	918	0.820788	101.9802	32.47776	212.02	101.98
315	ydcI	0.690858	0.152633	2	924	0.816351	103.0275	32.70715	211.97	103.03
316	ddpF	0.693101	0.15341	2	927	0.814145	103.552	32.76961	212.45	103.55
317	alsK	0.695344	0.154188	2	930	0.811949	104.0769	32.83185	212.92	104.08
318	mmuM	0.697587	0.154967	2	933	0.809762	104.6024	32.89385	213.40	104.60
319	fixB	0.704316	0.157307	2	942	0.803253	106.182	33.28589	212.82	106.18
320	hycC	1.366014	0.15835	3	1827	0.800381	106.8864	33.402	213.11	106.89
321	paaX	0.711045	0.159653	2	951	0.796822	107.766	33.57196	213.23	107.77
322	gshB	0.711045	0.159653	2	951	0.796822	107.766	33.4677	214.23	107.77
323	corA	0.711045	0.159653	2	951	0.796822	107.766	33.36408	215.23	107.77
324	rpoD	1.377229	0.161027	3	1842	0.793102	108.693	33.54722	215.31	108.69
325	fecD	0.715531	0.161221	2	957	0.792577	108.8244	33.48443	216.18	108.82
326	yjiA	0.715531	0.161221	2	957	0.792577	108.8244	33.38172	217.18	108.82
327	paoB	0.715531	0.161221	2	957	0.792577	108.8244	33.27964	218.18	108.82
328	aes	0.717774	0.162006	2	960	0.790468	109.3543	33.33974	218.65	109.35
329	ssuA	0.717774	0.162006	2	960	0.790468	109.3543	33.2384	219.65	109.35
330	stfQ	0.720017	0.162792	2	963	0.788366	109.8847	33.2984	220.12	109.88
331	yhbE	0.72226	0.163579	2	966	0.786273	110.4156	33.35818	220.58	110.42
332	iaaA	0.72226	0.163579	2	966	0.786273	110.4156	33.2577	221.58	110.42
333	glk	0.72226	0.163579	2	966	0.786273	110.4156	33.15783	222.58	110.42
334	sapB	0.72226	0.163579	2	966	0.786273	110.4156	33.05856	223.58	110.42
335	ycjG	0.72226	0.163579	2	966	0.786273	110.4156	32.95987	224.58	110.42
336	fcl	0.72226	0.163579	2	966	0.786273	110.4156	32.86178	225.58	110.42
337	dxs	1.392931	0.164796	3	1863	0.783052	111.2376	33.00819	225.76	111.24
338	kefC	1.392931	0.164796	3	1863	0.783052	111.2376	32.91053	226.76	111.24
339	manX	0.726746	0.165154	2	972	0.782112	111.4787	32.88456	227.52	111.48
340	ybeQ	0.731233	0.166731	2	978	0.777983	112.5435	33.10104	227.46	112.54
341	rcsC	2.130892	0.167234	4	2850	0.776675	112.8832	33.10357	228.12	112.88
342	cld	0.733476	0.167521	2	981	0.775931	113.0766	33.06334	228.92	113.08
343	ydaN	0.735719	0.168311	2	984	0.773887	113.6101	33.12249	229.39	113.61
344	kdsD	0.737962	0.169102	2	987	0.77185	114.1441	33.18143	229.86	114.14
345	flk	0.744691	0.171479	2	996	0.765788	115.7485	33.5503	229.25	115.75
346	idnR	0.746934	0.172273	2	999	0.763783	116.2842	33.60814	229.72	116.28
347	yphD	0.746934	0.172273	2	999	0.763783	116.2842	33.51128	230.72	116.28
348	yoaA	1.428819	0.173509	3	1911	0.760678	117.1186	33.65478	230.88	117.12
349	dppF	0.75142	0.173862	2	1005	0.759796	117.3566	33.62655	231.64	117.36
350	fliM	0.75142	0.173862	2	1005	0.759796	117.3566	33.53047	232.64	117.36
351	hflC	0.75142	0.173862	2	1005	0.759796	117.3566	33.43494	233.64	117.36
352	ynhG	0.75142	0.173862	2	1005	0.759796	117.3566	33.33996	234.64	117.36
353	hypE	0.755906	0.175453	2	1011	0.755839	118.4307	33.54978	234.57	118.43
354	yhjD	0.758149	0.176249	2	1014	0.753872	118.9684	33.60688	235.03	118.97
355	rnb	1.446764	0.177913	3	1935	0.749792	120.0914	33.82857	234.91	120.09
356	yjjN	0.764878	0.178642	2	1023	0.748016	120.5835	33.87178	235.42	120.58
357	ytfT	0.767121	0.179441	2	1026	0.746078	121.1227	33.92792	235.88	121.12
358	purR	0.767121	0.179441	2	1026	0.746078	121.1227	33.83314	236.88	121.12
359	yfiF	0.776093	0.182641	2	1038	0.738401	123.2828	34.34063	235.72	123.28
360	yghZ	0.778336	0.183443	2	1041	0.7365	123.8238	34.39549	236.18	123.82
361	yraQ	0.778336	0.183443	2	1041	0.7365	123.8238	34.30021	237.18	123.82
362	cheA	1.469194	0.183461	3	1965	0.736456	123.8361	34.20887	238.16	123.84
363	guaC	0.78058	0.184245	2	1044	0.734605	124.3651	34.26035	238.63	124.37
364	ypfG	0.78058	0.184245	2	1044	0.734605	124.3651	34.16622	239.63	124.37

365	galT	0.782823	0.185047	2	1047	0.732718	124.9067	34.22101	240.09	124.91
366	rsxD	0.791795	0.188261	2	1059	0.725239	127.0765	34.72035	238.92	127.08
367	yphC	0.794038	0.189066	2	1062	0.723386	127.6197	34.77377	239.38	127.62
368	ycjF	0.794038	0.189066	2	1062	0.723386	127.6197	34.67928	240.38	127.62
369	queA	0.800767	0.191484	2	1071	0.717868	129.2514	35.02748	239.75	129.25
370	ugpC	0.800767	0.191484	2	1071	0.717868	129.2514	34.93281	240.75	129.25
371	glpQ	0.805253	0.193097	2	1077	0.714224	130.3407	35.13227	240.66	130.34
372	rep	1.511812	0.194124	3	2022	0.711921	131.0337	35.22412	240.97	131.03
373	malS	1.518541	0.195822	3	2031	0.708139	132.1796	35.43688	240.82	132.18
374	rlpA	0.814225	0.19633	2	1089	0.707013	132.5228	35.43391	241.48	132.52
375	ybdH	0.814225	0.19633	2	1089	0.707013	132.5228	35.33942	242.48	132.52
376	yaiW	0.818711	0.197949	2	1095	0.703447	133.6156	35.53606	242.38	133.62
377	dcp	1.529756	0.198659	3	2046	0.701892	134.0947	35.56889	242.91	134.09
378	mltA	0.820954	0.198759	2	1098	0.701673	134.1624	35.49269	243.84	134.16
379	downstrean	0.821702	0.199029	2	1099	0.701083	134.3447	35.44715	244.66	134.34
380	upstream yl	1.531999	0.199227	3	2049	0.700651	134.4786	35.38909	245.52	134.48
381	lptF	0.823197	0.19957	2	1101	0.699906	134.7094	35.35681	246.29	134.71
382	chaA	0.823197	0.19957	2	1101	0.699906	134.7094	35.26425	247.29	134.71
383	helD	1.536486	0.200366	3	2055	0.698176	135.247	35.31253	247.75	135.25
384	asd	0.82544	0.20038	2	1104	0.698145	135.2567	35.22311	248.74	135.26
385	ribD	0.82544	0.20038	2	1104	0.698145	135.2567	35.13162	249.74	135.26
386	lacZ	2.299121	0.200474	4	3075	0.697942	135.3201	35.05702	250.68	135.32
387	ybiR	0.836656	0.20444	2	1119	0.689434	137.9972	35.65818	249.00	138.00
388	fes	0.841142	0.206067	2	1125	0.685992	139.095	35.84923	248.90	139.10
389	smf	0.841142	0.206067	2	1125	0.685992	139.095	35.75708	249.90	139.10
390	yhcG	0.843385	0.20688	2	1128	0.684281	139.6443	35.80623	250.36	139.64
391	fliK	0.843385	0.20688	2	1128	0.684281	139.6443	35.71466	251.36	139.64
392	ybhS	0.847871	0.208509	2	1134	0.680875	140.7436	35.90397	251.26	140.74
393	norW	0.847871	0.208509	2	1134	0.680875	140.7436	35.81261	252.26	140.74
394	potG	0.847871	0.208509	2	1134	0.680875	140.7436	35.72172	253.26	140.74
395	fepE	0.847871	0.208509	2	1134	0.680875	140.7436	35.63128	254.26	140.74
396	yhhI	0.850114	0.209324	2	1137	0.679181	141.2935	35.68018	254.71	141.29
397	nlpD	0.852357	0.210139	2	1140	0.677494	141.8437	35.72889	255.16	141.84
398	caiA	0.8546	0.210954	2	1143	0.675812	142.3941	35.77741	255.61	142.39
399	carA	0.859086	0.212586	2	1149	0.672465	143.4955	35.96379	255.50	143.50
400	agaS	0.863572	0.214219	2	1155	0.669142	144.5977	36.14944	255.40	144.60
401	metB	0.868058	0.215853	2	1161	0.665842	145.7007	36.33435	255.30	145.70
402	ynjB	0.872544	0.217488	2	1167	0.662564	146.8045	36.51853	255.20	146.80
403	nrdE	1.603777	0.217618	3	2145	0.662305	146.8922	36.44967	256.11	146.89
404	torC	0.87703	0.219124	2	1173	0.659309	147.909	36.61114	256.09	147.91
405	iadA	0.87703	0.219124	2	1173	0.659309	147.909	36.52074	257.09	147.91
406	hybB	0.881517	0.220762	2	1179	0.656076	149.0142	36.703	256.99	149.01
407	glcB	1.623964	0.222853	3	2172	0.651982	150.4255	36.95959	256.57	150.43
408	bcr	0.890489	0.224039	2	1191	0.649676	151.2265	37.06532	256.77	151.23
409	hofC	0.899461	0.22732	2	1203	0.643362	153.4413	37.5162	255.56	153.44
410	yhiN	0.899461	0.22732	2	1203	0.643362	153.4413	37.4247	256.56	153.44
411	proV	0.899461	0.22732	2	1203	0.643362	153.4413	37.33364	257.56	153.44
412	paaJ	0.901704	0.228141	2	1206	0.641796	153.9953	37.3775	258.00	154.00
413	gltS	0.901704	0.228141	2	1206	0.641796	153.9953	37.287	259.00	154.00
414	dacA	0.90619	0.229783	2	1212	0.638681	155.1038	37.46469	258.90	155.10
415	ydhC	0.90619	0.229783	2	1212	0.638681	155.1038	37.37441	259.90	155.10
416	sucB	0.910676	0.231426	2	1218	0.635587	156.2128	37.55116	259.79	156.21
417	ybdN	0.912919	0.232248	2	1221	0.634048	156.7675	37.59413	260.23	156.77
418	cusB	0.915162	0.23307	2	1224	0.632513	157.3223	37.63692	260.68	157.32
419	deoB	0.915162	0.23307	2	1224	0.632513	157.3223	37.54709	261.68	157.32
420	eutH	0.917405	0.233892	2	1227	0.630984	157.8772	37.58982	262.12	157.88
421	yhfW	0.917405	0.233892	2	1227	0.630984	157.8772	37.50053	263.12	157.88
422	pepT	0.917405	0.233892	2	1227	0.630984	157.8772	37.41167	264.12	157.88
423	yqeG	0.919648	0.234714	2	1230	0.62946	158.4323	37.45444	264.57	158.43
424	serA	0.921891	0.235537	2	1233	0.627941	158.9874	37.49703	265.01	158.99
425	yfgF	1.677797	0.236928	3	2244	0.625384	159.9261	37.62968	265.07	159.93

426	ptsP	1.68004	0.237517	3	2247	0.624305	160.3243	37.6348	265.68	160.32
427	intA	0.92862	0.238005	2	1242	0.623414	160.6535	37.62377	266.35	160.65
428	ybhO	0.92862	0.238005	2	1242	0.623414	160.6535	37.53586	267.35	160.65
429	hypF	1.684526	0.238698	3	2253	0.622152	161.121	37.55735	267.88	161.12
430	ftsW	0.930863	0.238828	2	1245	0.621914	161.209	37.49047	268.79	161.21
431	frsA	0.930863	0.238828	2	1245	0.621914	161.209	37.40349	269.79	161.21
432	yjeP	2.485293	0.239285	4	3324	0.621085	161.5172	37.38825	270.48	161.52
433	mscM	0.933107	0.239651	2	1248	0.62042	161.7647	37.35905	271.24	161.76
434	fryC	0.933107	0.239651	2	1248	0.62042	161.7647	37.27297	272.24	161.76
435	entS	0.93535	0.240475	2	1251	0.618931	162.3204	37.31504	272.68	162.32
436	ycjT	1.695742	0.241653	3	2268	0.616808	163.1159	37.41189	272.88	163.12
437	hflK	0.942079	0.242945	2	1260	0.614491	163.9881	37.52588	273.01	163.99
438	rhaA	0.942079	0.242945	2	1260	0.614491	163.9881	37.44021	274.01	163.99
439	ydeP	1.704714	0.244022	3	2280	0.612572	164.7146	37.52042	274.29	164.71
440	puuE	0.946565	0.244593	2	1266	0.611556	165.1003	37.52279	274.90	165.10
441	uidC	0.946565	0.244593	2	1266	0.611556	165.1003	37.43771	275.90	165.10
442	sufD	0.951051	0.246241	2	1272	0.608639	166.2128	37.6047	275.79	166.21
443	hisS	0.953294	0.247065	2	1275	0.607188	166.7691	37.64539	276.23	166.77
444	yggP	0.955537	0.24789	2	1278	0.605742	167.3255	37.68592	276.67	167.33
445	livM	0.955537	0.24789	2	1278	0.605742	167.3255	37.60123	277.67	167.33
446	bioA	0.964509	0.251188	2	1290	0.600002	169.5516	38.01605	276.45	169.55
447	dcuA	0.973481	0.254487	2	1302	0.594335	171.7785	38.42919	275.22	171.78
448	ybbY	0.973481	0.254487	2	1302	0.594335	171.7785	38.34341	276.22	171.78
449	upstream y1	3.393726	0.254653	5	4539	0.594051	171.8909	38.28306	277.11	171.89
450	yhfT	0.975724	0.255312	2	1305	0.59293	172.3353	38.29673	277.66	172.34
451	gltP	0.982454	0.257786	2	1314	0.58874	174.0059	38.58223	276.99	174.01
452	murD	0.984697	0.258612	2	1317	0.587352	174.5628	38.62008	277.44	174.56
453	brnQ	0.98694	0.259437	2	1320	0.585969	175.1197	38.65777	277.88	175.12
454	ykgC	0.991426	0.261087	2	1326	0.583215	176.2336	38.81798	277.77	176.23
455	yliF	0.993669	0.261912	2	1329	0.581845	176.7906	38.85508	278.21	176.79
456	hslU	0.995912	0.262737	2	1332	0.580479	177.3476	38.89201	278.65	177.35
457	qseF	0.998155	0.263562	2	1335	0.579117	177.9046	38.92879	279.10	177.90
458	glmM	1.000398	0.264387	2	1338	0.577759	178.4616	38.9654	279.54	178.46
459	frlA	1.000398	0.264387	2	1338	0.577759	178.4616	38.88051	280.54	178.46
460	gudD	1.002641	0.265213	2	1341	0.576406	179.0185	38.91707	280.98	179.02
461	rarA	1.004884	0.266038	2	1344	0.575057	179.5755	38.95348	281.42	179.58
462	chbF	1.011613	0.268513	2	1353	0.571034	181.2464	39.23083	280.75	181.25
463	yeeF	1.016099	0.270163	2	1359	0.568373	182.3603	39.38668	280.64	182.36
464	yegQ	1.018342	0.270988	2	1362	0.567049	182.9172	39.42181	281.08	182.92
465	allB	1.018342	0.270988	2	1362	0.567049	182.9172	39.33704	282.08	182.92
466	ydbK	2.635577	0.271759	4	3525	0.565815	183.4377	39.3643	282.56	183.44
467	sdaB	1.022828	0.272638	2	1368	0.564413	184.031	39.40706	282.97	184.03
468	pgaA	1.81238	0.272696	3	2424	0.564321	184.07	39.3312	283.93	184.07
469	cpsG	1.025071	0.273463	2	1371	0.563101	184.5878	39.35774	284.41	184.59
470	glmU	1.025071	0.273463	2	1371	0.563101	184.5878	39.274	285.41	184.59
471	dbpA	1.027314	0.274288	2	1374	0.561793	185.1446	39.30883	285.86	185.14
472	fliI	1.027314	0.274288	2	1374	0.561793	185.1446	39.22555	286.86	185.14
473	ybiW	1.819109	0.274501	3	2433	0.561456	185.2884	39.17302	287.71	185.29
474	ygeH	1.029557	0.275113	2	1377	0.560489	185.7014	39.1775	288.30	185.70
475	patA	1.0318	0.275938	2	1380	0.559189	186.2581	39.21223	288.74	186.26
476	radA	1.034044	0.276763	2	1383	0.557893	186.8148	39.24681	289.19	186.81
477	atpD	1.034044	0.276763	2	1383	0.557893	186.8148	39.16453	290.19	186.81
478	fadE	1.828081	0.27691	3	2445	0.557662	186.9141	39.10337	291.09	186.91
479	narU	1.03853	0.278412	2	1389	0.555312	187.9281	39.23341	291.07	187.93
480	galP	1.043016	0.280061	2	1395	0.552747	189.0411	39.38357	290.96	189.04
481	hsdS	1.043016	0.280061	2	1395	0.552747	189.0411	39.30169	291.96	189.04
482	yhjA	1.045259	0.280885	2	1398	0.551471	189.5976	39.3356	292.40	189.60
483	gadB	1.047502	0.28171	2	1401	0.550198	190.154	39.36936	292.85	190.15
484	leuC	1.047502	0.28171	2	1401	0.550198	190.154	39.28802	293.85	190.15
485	fumC	1.049745	0.282534	2	1404	0.548929	190.7104	39.32172	294.29	190.71
486	ydcR	1.051988	0.283358	2	1407	0.547665	191.2667	39.35528	294.73	191.27

487	gnd	1.051988	0.283358	2	1407	0.547665	191.2667	39.27447	295.73	191.27
488	melB	1.054231	0.284182	2	1410	0.546403	191.8229	39.30797	296.18	191.82
489	yfjI	1.054231	0.284182	2	1410	0.546403	191.8229	39.22758	297.18	191.82
490	pykF	1.056474	0.285006	2	1413	0.545146	192.379	39.26103	297.62	192.38
491	araE	1.06096	0.286654	2	1419	0.542643	193.4912	39.40757	297.51	193.49
492	fryA	1.866213	0.287167	3	2496	0.541865	193.8379	39.39794	298.16	193.84
493	ymdC	1.063203	0.287477	2	1422	0.541397	194.0471	39.36047	298.95	194.05
494	miaB	1.065446	0.288301	2	1425	0.540154	194.603	39.39333	299.40	194.60
495	degP	1.065446	0.288301	2	1425	0.540154	194.603	39.31374	300.40	194.60
496	paaH	1.067689	0.289124	2	1428	0.538916	195.1588	39.34654	300.84	195.16
497	yehI	2.716327	0.289511	4	3633	0.538336	195.4196	39.31985	301.58	195.42
498	ybjT	1.069932	0.289948	2	1431	0.537681	195.7146	39.30011	302.29	195.71
499	dacB	1.072175	0.290771	2	1434	0.536449	196.2702	39.33271	302.73	196.27
500	cusS	1.078904	0.293239	2	1443	0.532778	197.9366	39.58733	302.06	197.94
501	uxaB	1.085634	0.295707	2	1452	0.529138	199.6023	39.84077	301.40	199.60
502	pepD	1.09012	0.297351	2	1458	0.52673	200.7122	39.98251	301.29	200.71
503	ydfI	1.092363	0.298173	2	1461	0.525531	201.267	40.01332	301.73	201.27
504	phoQ	1.092363	0.298173	2	1461	0.525531	201.267	39.93393	302.73	201.27
505	ttdT	1.094606	0.298995	2	1464	0.524336	201.8217	39.96469	303.18	201.82
506	rng	1.099092	0.300638	2	1470	0.521956	202.9308	40.1049	303.07	202.93
507	narZ	2.797077	0.307412	4	3741	0.512279	207.5032	40.92766	299.50	207.50
508	acnB	1.942477	0.307758	3	2598	0.511791	207.7367	40.89305	300.26	207.74
509	topA	1.942477	0.307758	3	2598	0.511791	207.7367	40.81271	301.26	207.74
510	ravA	1.119279	0.308023	2	1497	0.511416	207.9158	40.76781	302.08	207.92
511	lyxK	1.119279	0.308023	2	1497	0.511416	207.9158	40.68803	303.08	207.92
512	elfC	1.94472	0.308365	3	2601	0.510935	208.1462	40.65355	303.85	208.15
513	glcD	1.121522	0.308843	2	1500	0.510262	208.4691	40.63725	304.53	208.47
514	yjgR	1.123765	0.309663	2	1503	0.509111	209.0222	40.6658	304.98	209.02
515	rbsA	1.126008	0.310482	2	1506	0.507964	209.5752	40.69421	305.42	209.58
516	casA	1.128251	0.311301	2	1509	0.50682	210.1281	40.72249	305.87	210.13
517	bcsA	1.958178	0.312005	3	2619	0.505838	210.6035	40.73569	306.40	210.60
518	araG	1.132738	0.312938	2	1515	0.504541	211.2333	40.77864	306.77	211.23
519	lysU	1.134981	0.313757	2	1518	0.503407	211.7858	40.8065	307.21	211.79
520	ycgG	1.139467	0.315393	2	1524	0.501148	212.8902	40.94041	307.11	212.89
521	ybcK	1.14171	0.316211	2	1527	0.500024	213.4421	40.96778	307.56	213.44
522	narH	1.150682	0.319479	2	1539	0.495557	215.6485	41.31197	306.35	215.65
523	tyrR	1.152925	0.320296	2	1542	0.494449	216.1997	41.33837	306.80	216.20
524	aceE	1.991824	0.32111	3	2664	0.493346	216.7496	41.36442	307.25	216.75
525	ilvA	1.155168	0.321112	2	1545	0.493343	216.7507	41.28584	308.25	216.75
526	ygcB	1.994067	0.321718	3	2667	0.492525	217.1594	41.28505	308.84	217.16
527	caiC	1.161897	0.32356	2	1554	0.490046	218.4027	41.44265	308.60	218.40
528	rtn	1.16414	0.324375	2	1557	0.488953	218.9531	41.46839	309.05	218.95
529	cydA	1.173112	0.327634	2	1569	0.484612	221.1527	41.80581	307.85	221.15
530	entF	2.9025	0.330936	4	3882	0.480256	223.3816	42.14747	306.62	223.38
531	ybiP	1.184328	0.331701	2	1584	0.479253	223.8981	42.16537	307.10	223.90
532	prfC	1.188814	0.333326	2	1590	0.477131	224.995	42.29229	307.01	224.99
533	lsrK	1.191057	0.334138	2	1593	0.476074	225.5431	42.31578	307.46	225.54
534	ygiS	1.202272	0.338194	2	1608	0.470834	228.2808	42.74921	305.72	228.28
535	entE	1.204515	0.339004	2	1611	0.469795	228.8277	42.77153	306.17	228.83
536	thiP	1.204515	0.339004	2	1611	0.469795	228.8277	42.69173	307.17	228.83
537	mppA	1.206758	0.339814	2	1614	0.468759	229.3744	42.71404	307.63	229.37
538	pck	1.213487	0.342242	2	1623	0.465667	231.0132	42.93926	306.99	231.01
539	ubiB	1.226945	0.347089	2	1641	0.459559	234.2851	43.46662	304.71	234.29
540	yojI	1.229188	0.347896	2	1644	0.458551	234.8296	43.48697	305.17	234.83
541	yidC	1.231431	0.348702	2	1647	0.457545	235.3739	43.5072	305.63	235.37
542	actP	1.233675	0.349508	2	1650	0.456543	235.918	43.52731	306.08	235.92
543	yfaQ	1.233675	0.349508	2	1650	0.456543	235.918	43.44715	307.08	235.92
544	yjcE	1.233675	0.349508	2	1650	0.456543	235.918	43.36728	308.08	235.92
545	lldP	1.238161	0.351119	2	1656	0.454545	237.0055	43.48724	307.99	237.01
546	opgD	1.238161	0.351119	2	1656	0.454545	237.0055	43.40759	308.99	237.01
547	yqiK	1.242647	0.352729	2	1662	0.452559	238.092	43.52687	308.91	238.09

548	tar	1.242647	0.352729	2	1662	0.452559	238.092	43.44744	309.91	238.09
549	hyfG	1.247133	0.354337	2	1668	0.450583	239.1775	43.56603	309.82	239.18
550	hscC	1.249376	0.355141	2	1671	0.4496	239.7199	43.58544	310.28	239.72
551	glnE	2.124163	0.356889	3	2841	0.447467	240.9001	43.72053	310.10	240.90
552	bcsG	1.256105	0.357549	2	1680	0.446664	241.3457	43.72205	310.65	241.35
553	ybjL	1.260591	0.359153	2	1686	0.44472	242.4284	43.83876	310.57	242.43
554	fadD	1.260591	0.359153	2	1686	0.44472	242.4284	43.75963	311.57	242.43
555	yddA	1.260591	0.359153	2	1686	0.44472	242.4284	43.68079	312.57	242.43
556	ilvB	1.262834	0.359955	2	1689	0.443752	242.9693	43.69951	313.03	242.97
557	yedQ	1.26732	0.361556	2	1695	0.441824	244.0504	43.81516	312.95	244.05
558	xdhD	2.146594	0.362934	3	2871	0.440173	244.9803	43.90327	313.02	244.98
559	narQ	1.271806	0.363156	2	1701	0.439906	245.1305	43.85161	313.87	245.13
560	gcvP	2.148837	0.363538	3	2874	0.439451	245.3879	43.81927	314.61	245.39
561	hybC	1.274049	0.363956	2	1704	0.438951	245.6702	43.79147	315.33	245.67
562	ilvI	1.289751	0.369541	2	1725	0.432337	249.4404	44.38441	312.56	249.44
563	ptsI	1.291994	0.370338	2	1728	0.431402	249.9779	44.40106	313.02	249.98
564	yijP	1.29648	0.371929	2	1734	0.42954	251.0522	44.5128	312.95	251.05
565	dnaG	1.305452	0.375107	2	1746	0.425845	253.1974	44.8137	311.80	253.20
566	msbA	1.307695	0.375901	2	1749	0.424927	253.733	44.82915	312.27	253.73
567	ftsI	1.321153	0.380653	2	1767	0.419471	256.9406	45.3158	310.06	256.94
568	ade	1.321153	0.380653	2	1767	0.419471	256.9406	45.23602	311.06	256.94
569	nfrA	2.222857	0.383409	3	2973	0.416338	258.8009	45.48345	310.20	258.80
570	ygeV	1.330125	0.383812	2	1779	0.415881	259.0732	45.45143	310.93	259.07
571	nuoC	1.339098	0.386964	2	1791	0.412329	261.201	45.74449	309.80	261.20
572	cysJ	1.345827	0.389324	2	1800	0.409689	262.7937	45.94296	309.21	262.79
573	malZ	1.359285	0.394031	2	1818	0.40447	265.9709	46.41726	307.03	265.97
574	typA	1.363771	0.395596	2	1824	0.402748	267.0275	46.52046	306.97	267.03
575	uvrC	1.3705	0.397941	2	1833	0.400182	268.6099	46.71477	306.39	268.61
576	yfbS	1.3705	0.397941	2	1833	0.400182	268.6099	46.63367	307.39	268.61
577	btuB	1.379472	0.40106	2	1845	0.396791	270.7154	46.91775	306.28	270.72
578	sppA	1.388445	0.404171	2	1857	0.393434	272.8157	47.19995	305.18	272.82
579	ppiD	1.39966	0.40805	2	1872	0.389287	275.4336	47.57058	303.57	275.43
580	bglF	1.404146	0.409598	2	1878	0.387642	276.4785	47.6687	303.52	276.48
581	mdtF	2.32828	0.411446	3	3114	0.385687	277.7264	47.80144	303.27	277.73
582	acrD	2.32828	0.411446	3	3114	0.385687	277.7264	47.71931	304.27	277.73
583	flu	2.332766	0.412631	3	3120	0.384438	278.526	47.77462	304.47	278.53
584	deaD	1.413118	0.412688	2	1890	0.384379	278.5641	47.69933	305.44	278.56
585	acrB	2.355197	0.418543	3	3150	0.37826	282.5167	48.29344	302.48	282.52
586	nagE	1.455736	0.427252	2	1947	0.369316	288.3951	49.21418	297.60	288.40
587	gspD	1.460222	0.428774	2	1953	0.367772	289.4225	49.30536	297.58	289.42
588	topB	1.466951	0.431053	2	1962	0.365469	290.9608	49.48313	297.04	290.96
589	aaeB	1.471437	0.43257	2	1968	0.363944	291.9846	49.57293	297.02	291.98
590	speA	1.478166	0.434841	2	1977	0.36167	293.5174	49.74871	296.48	293.52
591	gmr	1.484895	0.437107	2	1986	0.359413	295.0469	49.92333	295.95	295.05
592	cirA	1.489382	0.438614	2	1992	0.357917	296.0647	50.01093	295.94	296.06
593	yghJ	3.411671	0.444193	4	4563	0.352429	299.83	50.56155	293.17	299.83
594	yphG	2.453891	0.444306	3	3282	0.352318	299.9064	50.4893	294.09	299.91
595	fadH	1.509569	0.445372	2	2019	0.351277	300.6262	50.52541	294.37	300.63
596	upstream in	1.511812	0.44612	2	2022	0.350548	301.1311	50.52536	294.87	301.13
597	uvrB	1.511812	0.44612	2	2022	0.350548	301.1311	50.44073	295.87	301.13
598	kdpB	1.531999	0.452827	2	2049	0.344068	305.6582	51.11341	292.34	305.66
599	lhr	3.452046	0.452985	4	4617	0.343916	305.7648	51.04588	293.24	305.76
600	yhjG	1.540972	0.455793	2	2061	0.341233	307.6601	51.27668	292.34	307.66
601	stfR	2.514453	0.459889	3	3363	0.337347	310.425	51.65142	290.57	310.43
602	kefA	2.514453	0.459889	3	3363	0.337347	310.425	51.56562	291.57	310.43
603	yjbH	1.567888	0.464634	2	2097	0.332889	313.6278	52.01125	289.37	313.63
604	yncD	1.572374	0.466099	2	2103	0.331522	314.6169	52.08888	289.38	314.62
605	ydhV	1.572374	0.466099	2	2103	0.331522	314.6169	52.00279	290.38	314.62
606	yhfK	1.572374	0.466099	2	2103	0.331522	314.6169	51.91697	291.38	314.62
607	rlmL	1.57686	0.467562	2	2109	0.330161	315.6043	51.99411	291.40	315.60
608	upstream y	1.58658	0.470723	2	2122	0.327234	317.7381	52.25956	290.26	317.74

609	speC	1.597048	0.474115	2	2136	0.324116	320.0275	52.54967	288.97	320.03
610	cadA	1.60602	0.477011	2	2148	0.321471	321.9828	52.78406	288.02	321.98
611	wzc	1.617235	0.480619	2	2163	0.318199	324.4175	53.09616	286.58	324.42
612	dnaE	2.604175	0.482618	3	3483	0.316397	325.7671	53.22992	286.23	325.77
613	katG	1.630693	0.484927	2	2181	0.314324	327.3255	53.3973	285.67	327.33
614	zntA	1.644152	0.489212	2	2199	0.310503	330.2184	53.78149	283.78	330.22
615	rsxC	1.662096	0.494892	2	2223	0.30549	334.0518	54.31737	280.95	334.05
616	yjdA	1.666582	0.496305	2	2229	0.304251	335.0059	54.38408	280.99	335.01
617	parC	1.689013	0.503334	2	2259	0.298143	339.7508	55.06496	277.25	339.75
618	ybhJ	1.691256	0.504034	2	2262	0.29754	340.2229	55.05225	277.78	340.22
619	clpA	1.702471	0.507521	2	2277	0.294546	342.577	55.34361	276.42	342.58
620	yfhM	3.709996	0.507931	4	4962	0.294195	342.8535	55.29895	277.15	342.85
621	pflB	1.706957	0.508912	2	2283	0.293357	343.5156	55.31652	277.48	343.52
622	nrdA	1.7092	0.509606	2	2286	0.292765	343.9842	55.30293	278.02	343.98
623	bglX	1.718172	0.512377	2	2298	0.29041	345.8544	55.51435	277.15	345.85
624	upstream a|	1.721163	0.513298	2	2302	0.28963	346.4762	55.52504	277.52	346.48
625	bisC	1.745089	0.520627	2	2334	0.283474	351.4229	56.22766	273.58	351.42
626	polB	1.758547	0.524716	2	2352	0.280076	354.1833	56.57881	271.82	354.18
627	pps	1.778734	0.530806	2	2379	0.275064	358.294	57.14418	268.71	358.29
628	gcd	1.787707	0.533495	2	2391	0.272869	360.1094	57.34226	267.89	360.11
629	malP	1.78995	0.534166	2	2394	0.272324	360.5621	57.32307	268.44	360.56
630	ybbP	1.805651	0.538842	2	2415	0.268538	363.7186	57.73311	266.28	363.72
631	gyrB	1.805651	0.538842	2	2415	0.268538	363.7186	57.64161	267.28	363.72
632	plsB	1.81238	0.540836	2	2424	0.266934	365.0646	57.76339	266.94	365.06
633	rnr	1.825838	0.544807	2	2442	0.263758	367.7446	58.09551	265.26	367.74
634	glgP	1.830324	0.546125	2	2448	0.262708	368.6342	58.1442	265.37	368.63
635	thrA	1.84154	0.549408	2	2463	0.260105	370.8506	58.40166	264.15	370.85
636	napA	1.859484	0.554627	2	2487	0.255999	374.3731	58.8637	261.63	374.37
637	purL	2.906986	0.555651	3	3888	0.255198	375.0647	58.87985	261.94	375.06
638	hrpA	2.918201	0.558236	3	3903	0.253182	376.8094	59.06103	261.19	376.81
639	copA	1.872942	0.558512	2	2505	0.252967	376.9959	58.9978	262.00	377.00
640	yraJ	1.881914	0.561089	2	2517	0.250968	378.7354	59.1774	261.26	378.74
641	nirB	1.902102	0.566848	2	2544	0.246533	382.6224	59.69148	258.38	382.62
642	torA	1.904345	0.567484	2	2547	0.246046	383.052	59.66542	258.95	383.05
643	mutS	1.91556	0.570657	2	2562	0.243625	385.1932	59.90563	257.81	385.19
644	clpB	1.924532	0.573182	2	2574	0.241707	386.8979	60.07731	257.10	386.90
645	leuS	1.931261	0.575069	2	2583	0.24028	388.1716	60.18164	256.83	388.17
646	sfmD	1.946963	0.579448	2	2604	0.236985	391.1275	60.54606	254.87	391.13
647	rpoB	3.012409	0.579584	3	4029	0.236883	391.2194	60.46667	255.78	391.22
648	fimD	1.971636	0.586262	2	2637	0.231908	395.7272	61.06901	252.27	395.73
649	ydbH	1.973879	0.586878	2	2640	0.231452	396.1426	61.03891	252.86	396.14
650	ppc	1.982851	0.589333	2	2652	0.22964	397.7995	61.19993	252.20	397.80
651	glnD	1.998553	0.593602	2	2673	0.226504	400.6815	61.54862	250.32	400.68
652	mgtA	2.016497	0.598441	2	2697	0.222979	403.9477	61.95517	248.05	403.95
653	secA	2.023226	0.600244	2	2706	0.221672	405.1649	62.04669	247.84	405.16
654	nuoG	2.038928	0.604428	2	2727	0.218655	407.989	62.38364	246.01	407.99
655	torS	2.052386	0.607988	2	2745	0.216105	410.3918	62.65523	244.61	410.39
656	yeeJ	5.291342	0.609059	5	7077	0.21534	411.1151	62.66999	244.88	411.12
657	rpoC	3.158207	0.611306	3	4224	0.213742	412.6313	62.80538	244.37	412.63
658	uvrA	2.110705	0.62313	2	2823	0.205421	420.6129	63.92293	237.39	420.61
659	mukB	3.335407	0.647635	3	4461	0.18867	437.1534	66.33588	221.85	437.15
660	upstream y|	2.269961	0.66216	2	3036	0.179037	446.958	67.72091	213.04	446.96
661	ydiJ	2.285662	0.665826	2	3057	0.176639	449.4327	67.99284	211.57	449.43
662	sbcC	2.352954	0.681175	2	3147	0.166741	459.7929	69.45513	202.21	459.79
663	rne	2.382113	0.687644	2	3186	0.162636	464.1599	70.00903	198.84	464.16
664	carB	2.40903	0.69352	2	3222	0.158941	468.1259	70.50088	195.87	468.13
665	yegE	2.480807	0.708741	2	3318	0.149512	478.4003	71.9399	186.60	478.40
666	mfd	2.577258	0.728193	2	3447	0.137754	491.53	73.8033	174.47	491.53
667	hsdR	2.626605	0.737711	2	3513	0.132114	497.9549	74.65591	169.05	497.95
668	recB	2.649036	0.741942	2	3543	0.12963	500.8112	74.97173	167.19	500.81
669	evgS	2.687167	0.749002	2	3594	0.125517	505.5761	75.57191	163.42	505.58

670	metH	2.754459	0.761053	2	3684	0.118585	513.7109	76.67328	156.29	513.71
671	yfaL	2.806049	0.76995	2	3753	0.113538	519.716	77.45395	151.28	519.72
672	upstream y(	2.950351	0.793312	2	3946	0.100556	535.4859	79.6854	136.51	535.49
673	rhsA	3.090915	0.814026	2	4134	0.089362	549.4673	81.64448	123.53	549.47
674	gltB	3.404942	0.853717	2	4554	0.068686	576.2592	85.4984	97.74	576.26
675	ypjA	3.425129	0.855984	2	4581	0.067534	577.7891	85.59838	97.21	577.79
				1939						0
				0.579498						0
										276
										0.082487

Table S3. Population allele frequencies for frequently mutated genes

		Alleles in each population				Cumulative frequency at end of experiment		
**Gene**	**Observed Mutations**	**1**	**2**	**3**	**Total unique alleles**	**1**	**2**	**3**
galS	38	4	24	6	30	0	0	0.06
hfq	24	10	11	3	14	0.91	0.50	0.86
pgi	35	10	6	19	24	0.02	0.49	0.60
opgH	31	9	11	11	29	0.95	0.69	0.68
malT	30	1	18	9	19	0	0.51	0.84
malK	22	2	13	7	14	0.99	0.21	0.03
**upstream *mglB***	7	2	3	2	4	1.00	0.94	0.94
rho	11	4	3	4	9	0.99	0.02	0.95
**upstream *dnaG***	5	2	3	0	5	1.00	0.23	0
fimH	9	4	3	2	5	0	0.16	0.09

Table S4. Identical mutations arise within and among replicate evolution experiments.

Chemostat	Nucleotide position	Ancestral	Derived	Gene/Region	Amino acid change
all 3	55258	C	A	lptD	Glu618*
	2238647	C	A	upstream mglB*	none
	2238671	G	T	galS	Asn340Lys
	2239254	C	A	galS	Arg146Leu
	2790401	G	T	gabD	Gln369His
	3481115	G	T	yheS	Cys602Phe
	4232061	G	T	pgi	Arg94Leu
	4232255	G	T	pgi	Asp159Tyr
	4232262	G	T	pgi	Gly161Val
	4245693	C	A	malK	Ala296Asp
	4398360	G	T	hfq	Arg17Leu
	4398395	G	T	hfq	Gly29Cys
	4547110	G	T	fimH	Gly94Trp
1 and 2	294752	C	A	yagN	Ala18Ser
	505592	C	A	ushA	none
	1108798	G	T	opgG	Glu81*
	1111307	C	A	opgH	Gln408Lys
	1297594	C	A	upstream adhE	N/A
	3438088	G	T	rpoA	Pro322Thr
	3964698	C	A	rho	Arg87Ser
	4232851	C	A	pgi	Asn357Lys
	4245697	C	A	malK	Asp297Glu
	4367603	G	T	yjeH	Ala278Asp
	4398388	G	T	hfq	Leu26Phe
	4398403	G	T	hfq	Lys31Asn
	4398404	C	A	hfq	Leu32Met
	4398494	G	T	hfq	Val62Phe
	4398501	C	A	hfq	Pro64Gln
	4398665	C	A	downstream hfq/upstream hflX	N/A
	4547014	C	A	fimH	Gln62Lys
	4629113	G	T	slt	Ala120Ser
1 and 3	501321	G	T	ybaL	Pro381Gln
	3368984	C	A	nanE	none
	3965316	G	T	rho	Ala293Ser
	4185625	T	C	rpoC	none
	4232066	G	T	pgi	Val96Leu
	4232436	C	A	pgi	Ala219Asp

	4232577	G	T	pgi	Trp266Leu
	4547002	G	T	fimH	Asp58Tyr
**2 and 3**	1111193	G	T	opgH	Gly370Trp
	1338741	G	T	yciM	Ala54Ser
	1905184	C	A	upstream rlmA	
	1905560	C	A	yobF	Arg19Leu
	2012958	G	T	fliG	Glu19*
	2238648	G	T	upstream mglB	
	2864679	G	T	rpoS	Arg299Ser
	3342432	C	A	lptB	Pro156Gln
	3421312	A	C	downstream yhdZ/upstream rrfF	
	3551116	C	A	malT	Pro4Thr
	3551131	C	A	malT	Arg9Ser
	3551264	C	A	malT	Ala53Glu
	3551285	G	T	malT	Gly60Val
	3551833	C	A	malT	Leu243Met
	3552039	G	T	malT	Met311Ile
	3552062	G	T	malT	Cys319Phe
	3552180	C	A	malT	Ser358Arg
	3553006	C	A	malT	Arg634Ser
	4105641	G	T	pfkA	Gly23Trp
	4233075	C	A	pgi	Ala432Glu
	4244957	G	T	malK	Gly51Trp
	4245480	C	A	malK	Pro225Gln
	4245498	C	A	malK	Ala231Asp
	4245563	C	A	malK	Gln253Lys
	4245821	G	T	malK	Glu339*
	4398489	C	A	hfq	Ser60Tyr

- present at 1.4% at gen 0 in chemostat 1, but drops to undetectable at g 50.

Table S5. Fixed alleles among replicate populations ("fixed" defined as >98% at any time point between generation 50 and 500).

						**Generation**										
**Chemostat**	**Nucleotide Position**	**Ancestral**	**Derived**	**Region**	**Amino acid change**	**0**	**50**	**100**	**150**	**200**	**250**	**300**	**350**	**400**	**450**	**500**
**1**	438,942	C	A	*dxs*	Ala154Ser	0	0	93	12	88	94	94	78	100	100	100
**1**	546,837	G	T	*fdrA*	Ala312Ser	0	0	93	11	87	96	93	82	98	100	99
**1**	922,714	C	A	*clpA*		0	0	93	12	85	95	93	78	99	99	99
**1**	1,107,984	G	T	*opgC*	Gln61Lys	0	0	92	14	85	95	93	79	100	100	100
**1**	1,361,341	C	A	*puuC*	Pro192Gln	0	0	93	13	86	95	93	81	99	100	100
**1**	2,019,771	G	T	*fliO*	Val82Phe	0	0	92	11	87	95	93	77	99	99	100
**1**	2,119,008	C	A	*wcaJ*	Gly191Cys	0	0	92	15	85	95	93	79	100	100	100
**1**	2,238,647	C	A	upstream *mglB*		1	0	95	12	88	95	92	77	99	100	100
**1**	2,408,047	G	T	*yfbS*	Ala443Asp	0	0	94	11	87	96	95	81	100	100	100
**1**	2,500,493	G	T	*fryA*	Ala672Glu	0	0	94	12	86	95	93	79	99	100	100
**1**	2,775,348	G	T	*yfjZ*	Arg71Leu	0	0	94	13	86	95	90	79	100	100	100
**1**	3,209,081	G	T	upstream *dnaG*		0	92	98	93	95	99	99	99	99	100	100
**1**	3,965,271	G	T	*rho*	Val278Phe	0	0	93	13	87	93	91	82	98	99	99
**1**	4,205,962	G	T			0	0	93	10	89	95	91	82	99	100	100
**1**	4,245,693	C	A	*malK*	Ala296Asp	0	0	94	12	89	95	93	83	99	100	99
**3**	82,6641	C	A	*ybhF*	Glu522*	9	79	91	97	98	91	89				
**3**	2,238,647	C	A	upstream *mglB*		0	81	96	99	97	94	94				
