# Supplementary material for: Evolutionary dynamics and structural consequences of de novo beneficial mutations and mutant lineages arising in a constant environment
Source: BMC Biol. 2021 Feb 4;19:20. doi: 10.1186/s12915-021-00954-0 (PMC7863352; doi:10.1186/s12915-021-00954-0)

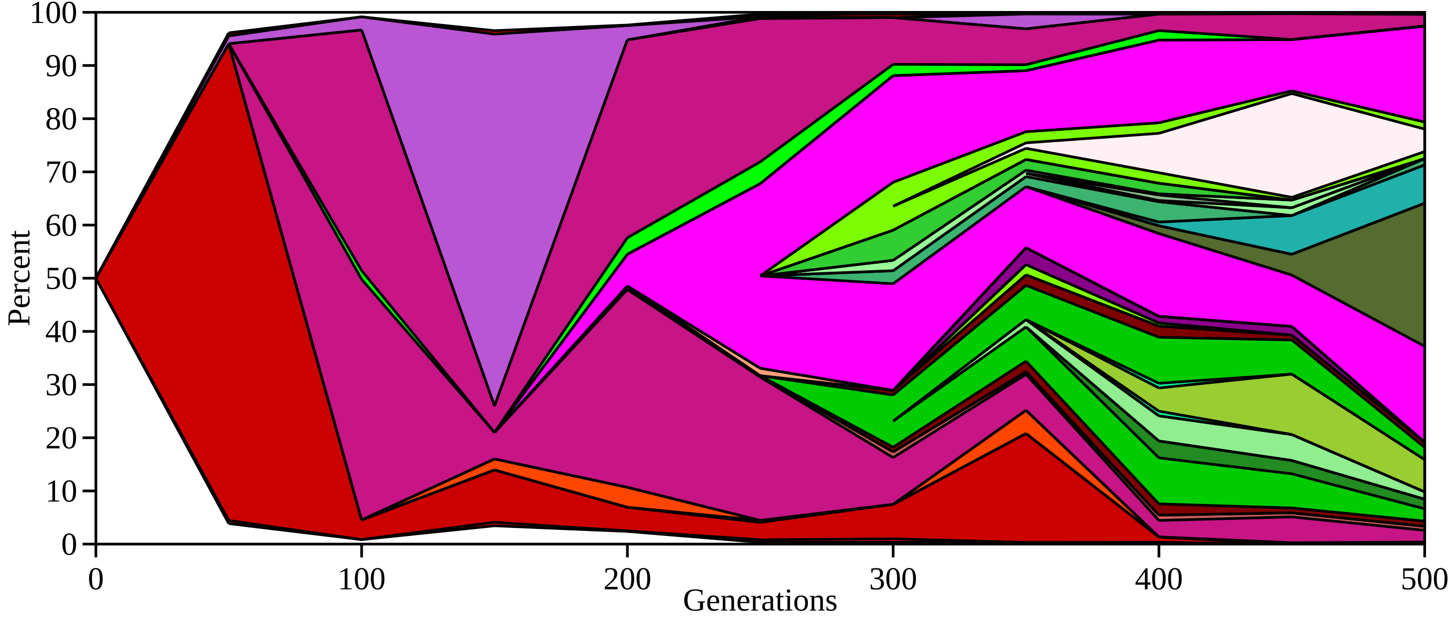

Percent
100
90
80
70
60
50
40
30
20
10
0
0
100
200
300
400
500
Generations

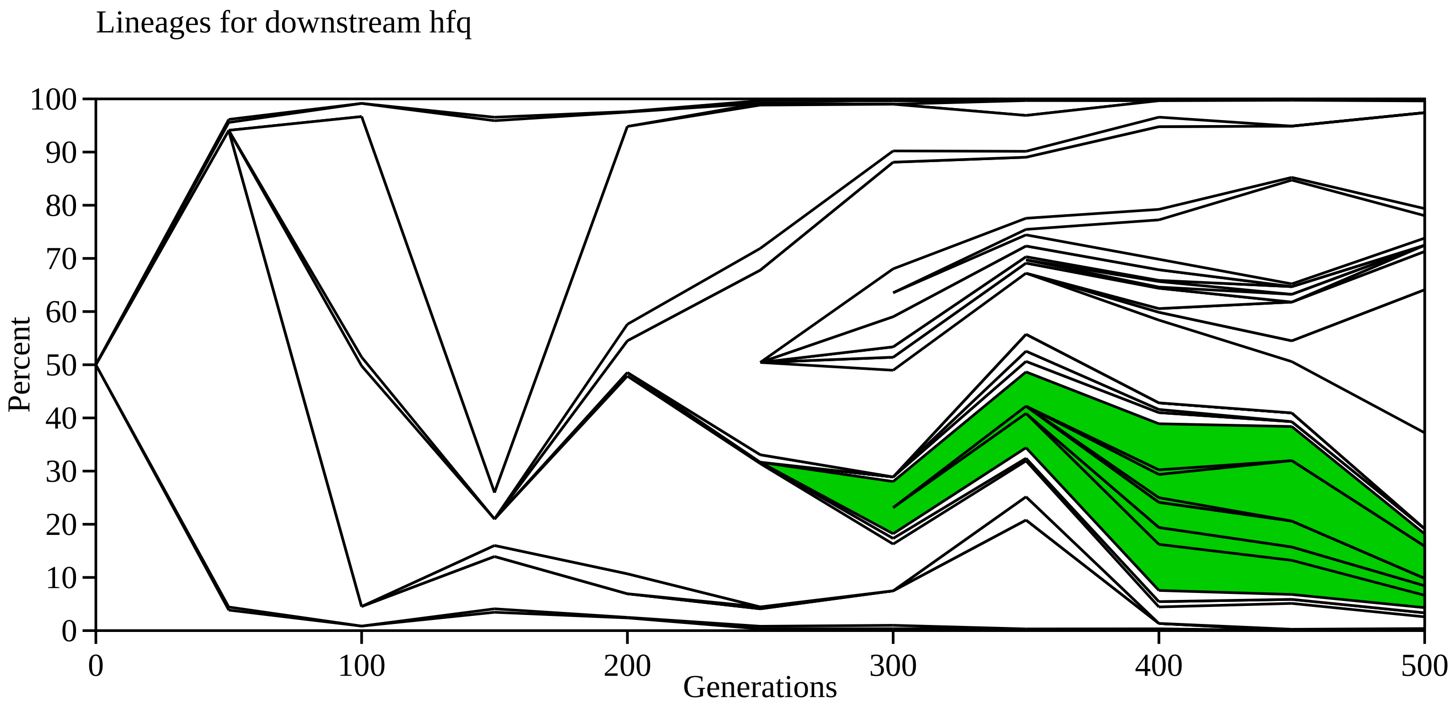
Lineages for downstream hfq
Percent
100
90
80
70
60
50
40
30
20
10
0
0
100
200
300
400
500
Generations

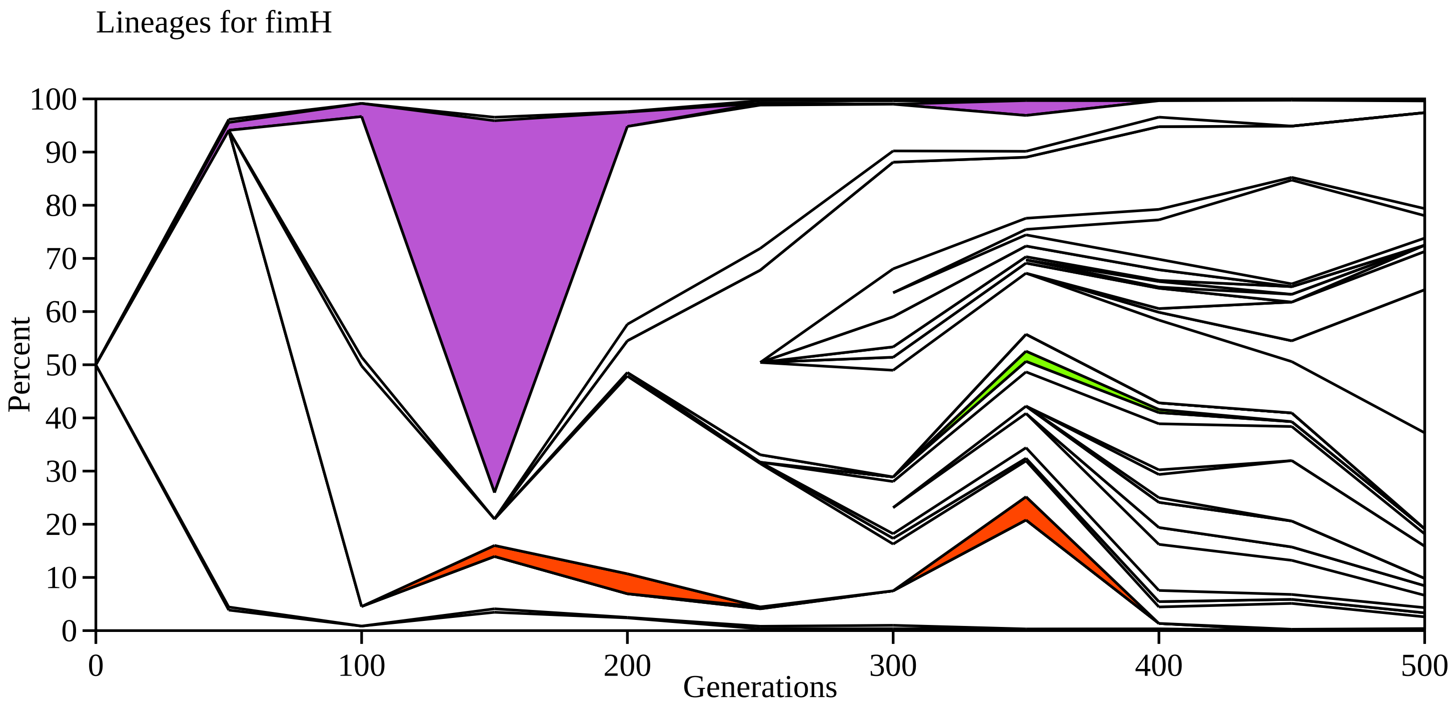
Lineages for fimH
Percent
100
90
80
70
60
50
40
30
20
10
0
0
100
200
300
400
500
Generations

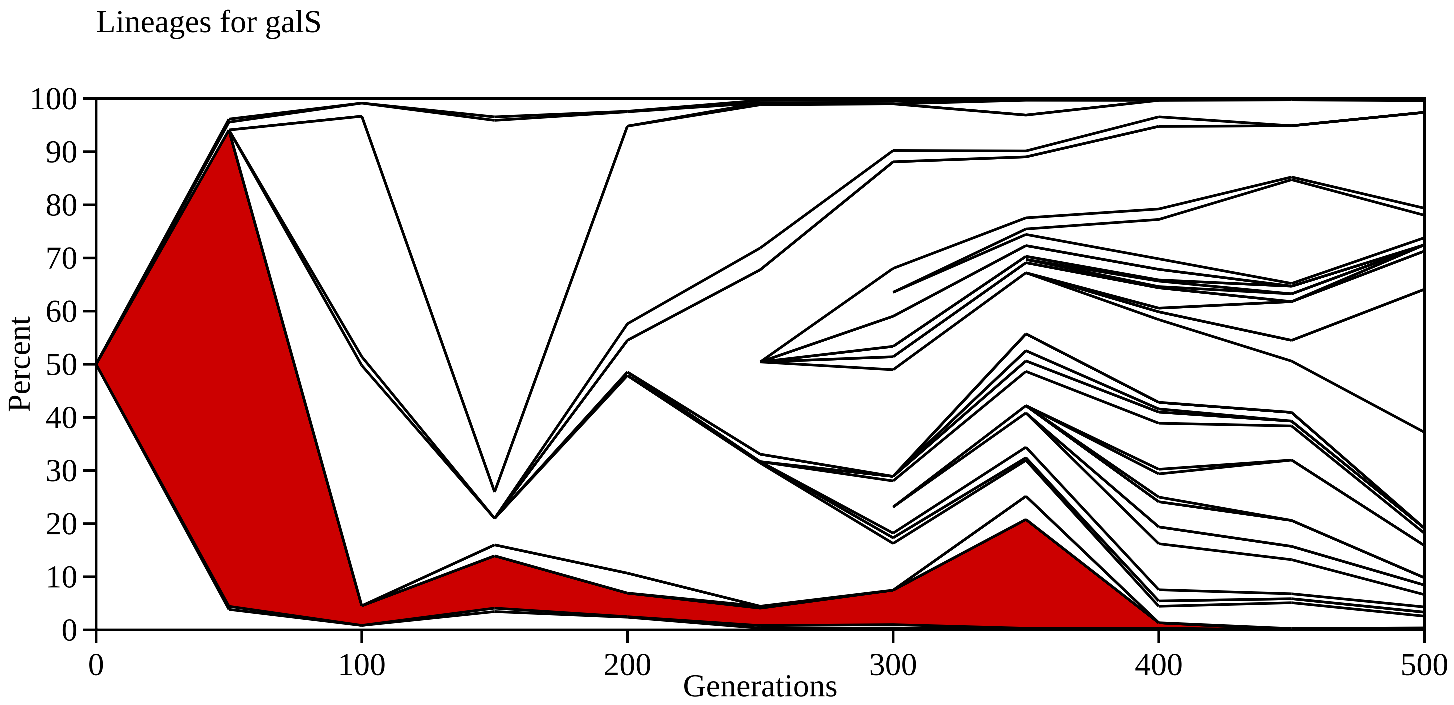

Lineages for galS
Percent
Generations
0
10
20
30
40
50
60
70
80
90
100
0
100
200
300
400
500

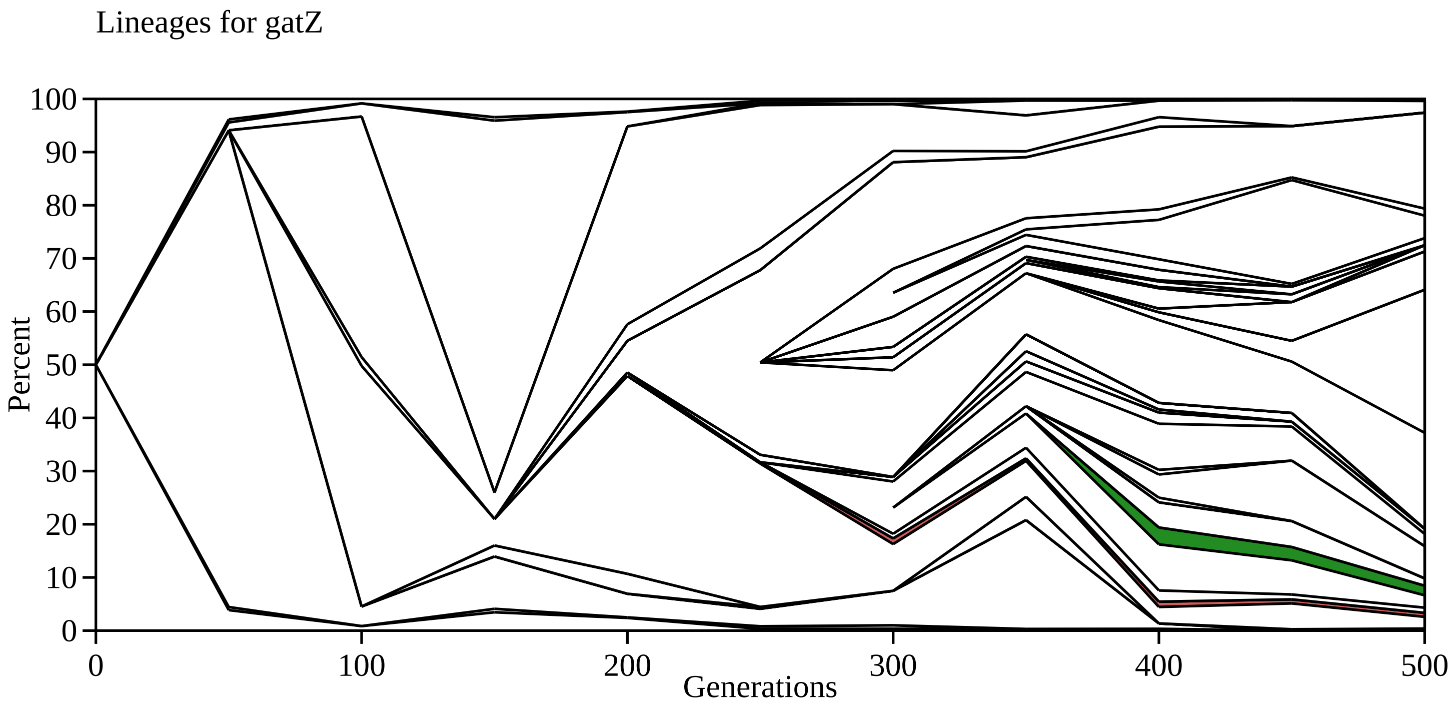

Lineages for gatZ
Percent
Generations
0
10
20
30
40
50
60
70
80
90
100
0
100
200
300
400
500

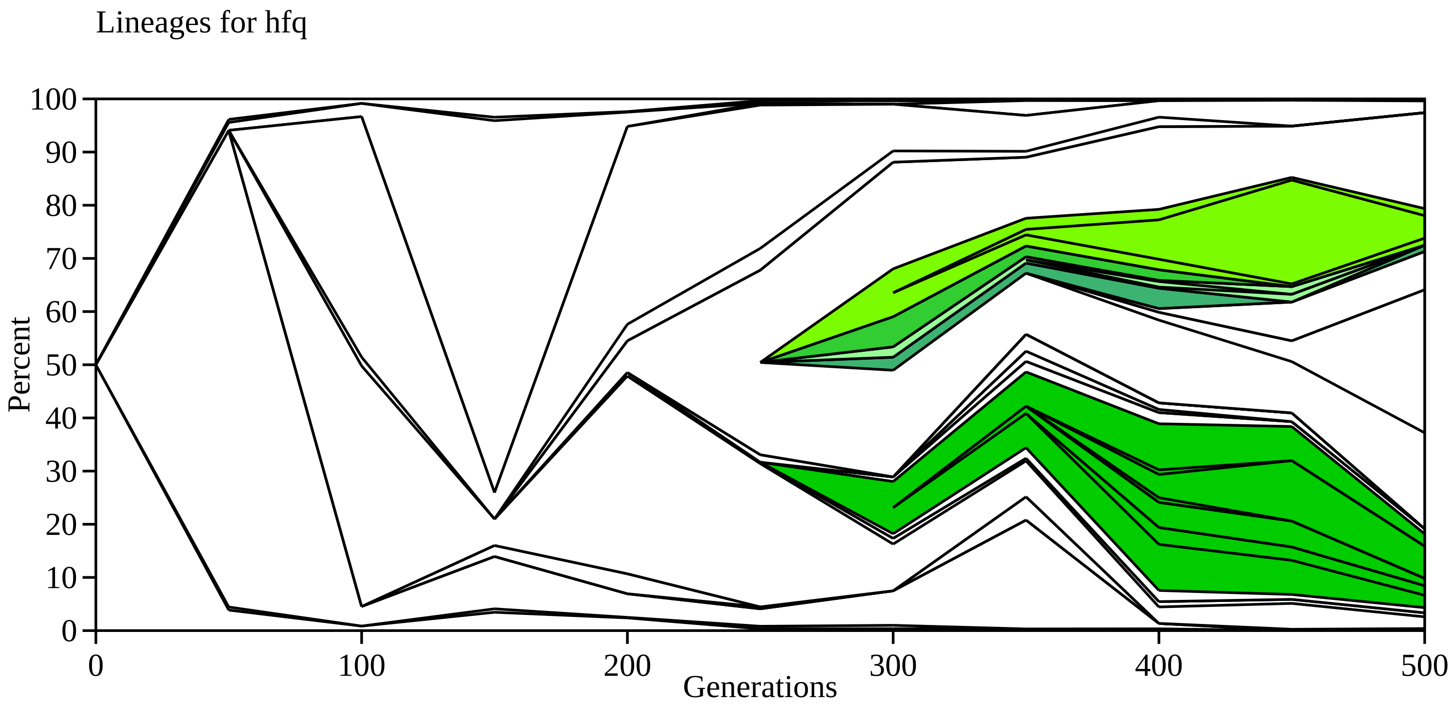

Lineages for hfq
Percent
Generations
0
10
20
30
40
50
60
70
80
90
100
0
100
200
300
400
500

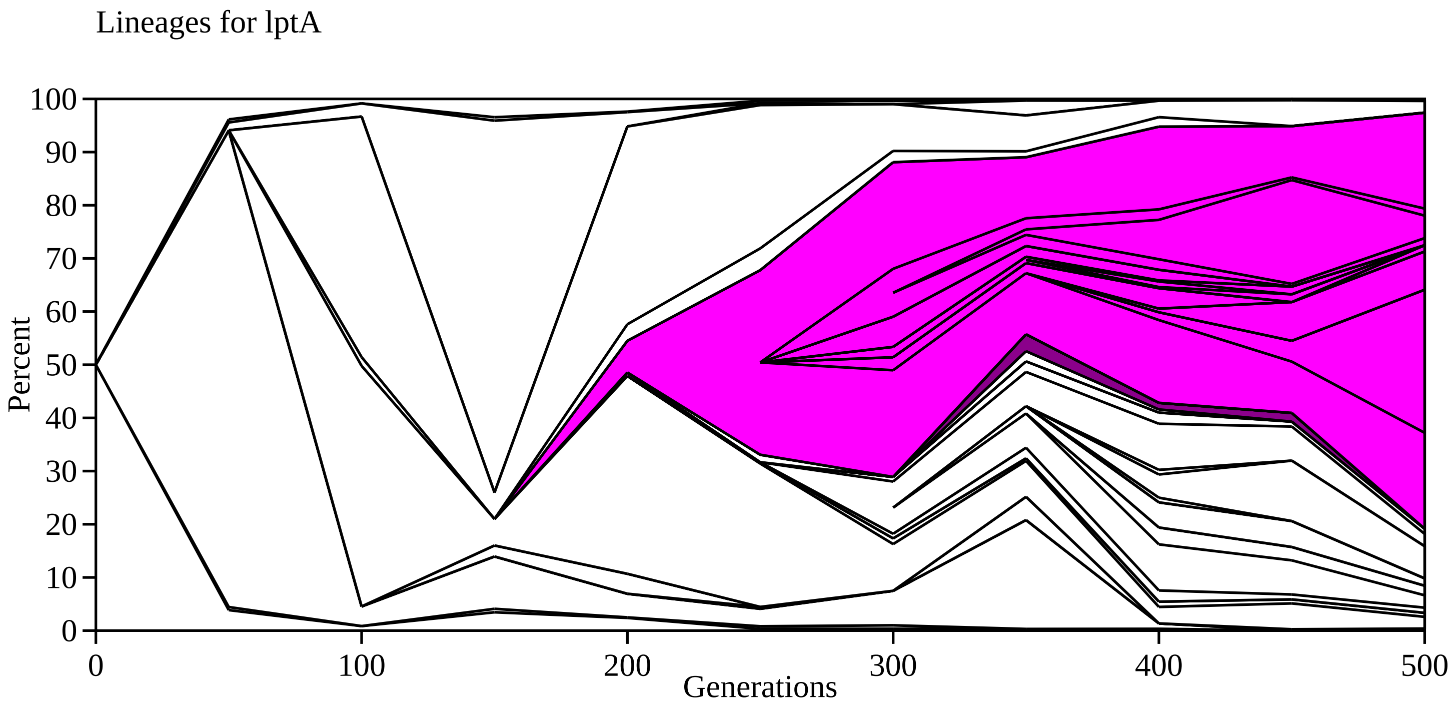
Lineages for lptA
Percent
0
10
20
30
40
50
60
70
80
90
100
0
100
200
300
400
500
Generations

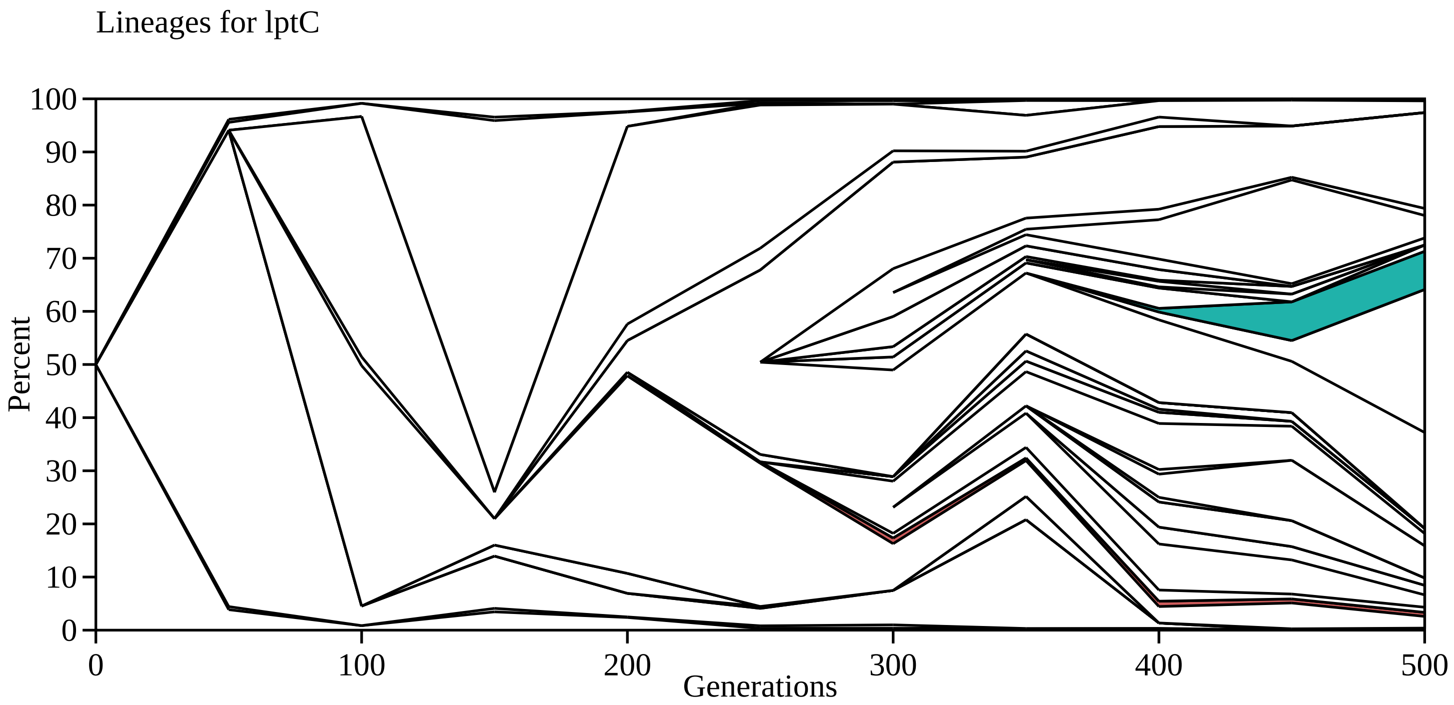
Lineages for lptC
Percent
Generations
0
10
20
30
40
50
60
70
80
90
100
0
100
200
300
400
500

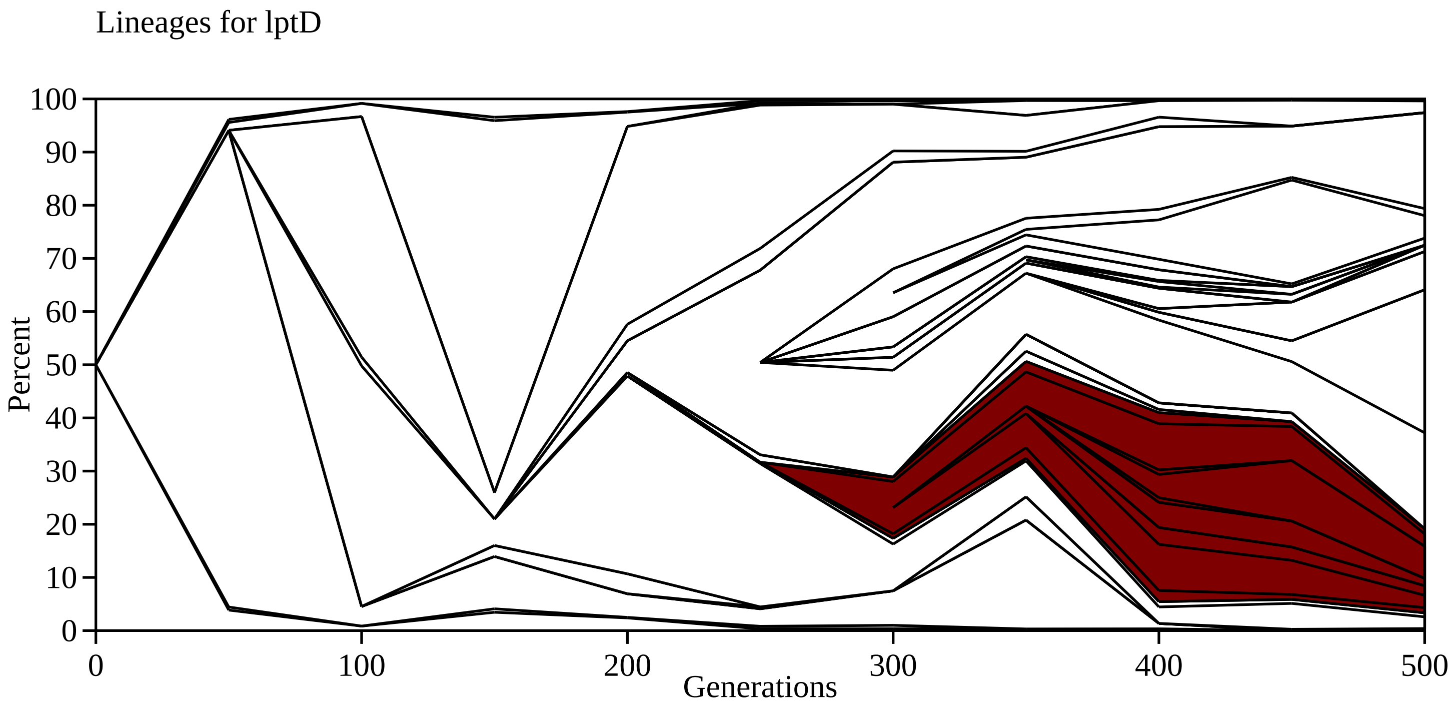
Lineages for lptD
Percent
Generations
0
10
20
30
40
50
60
70
80
90
100
0
100
200
300
400
500

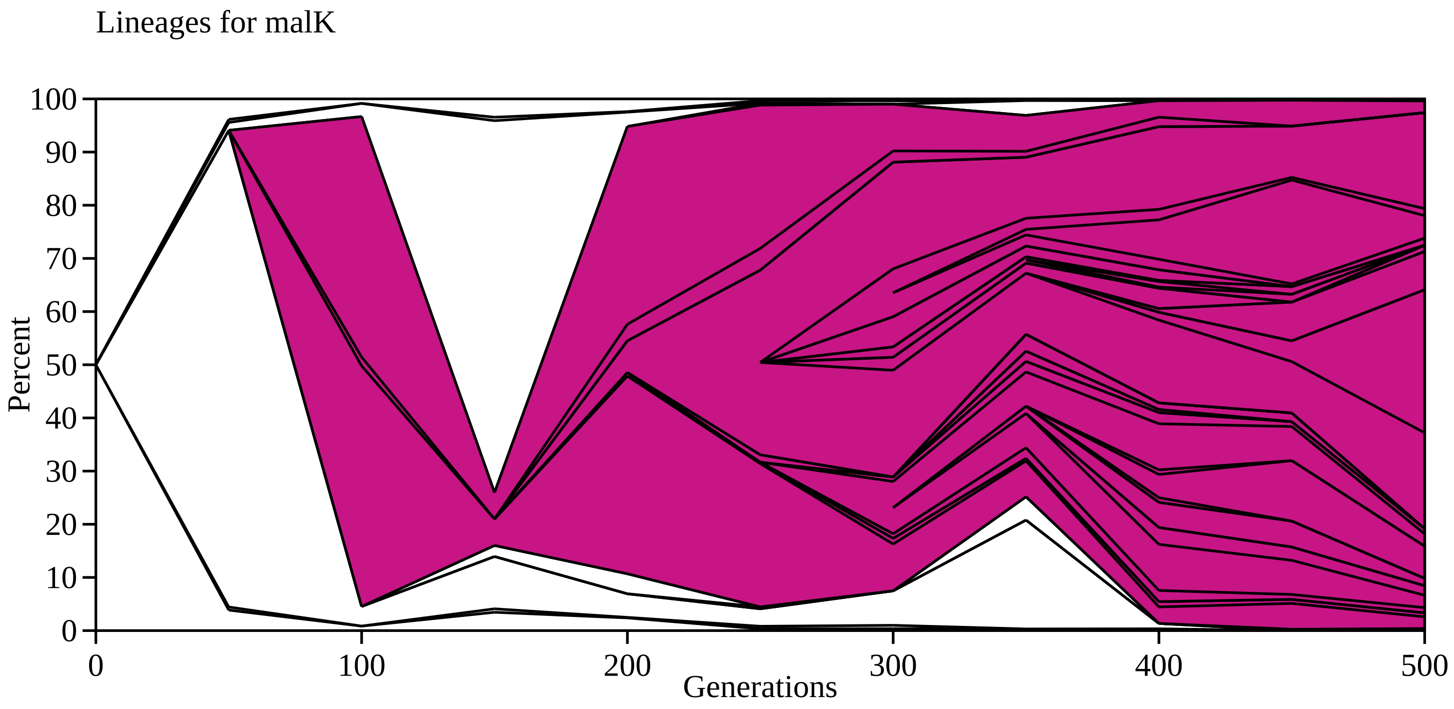
Lineages for malK
Percent
Generations
0
10
20
30
40
50
60
70
80
90
100
0
100
200
300
400
500

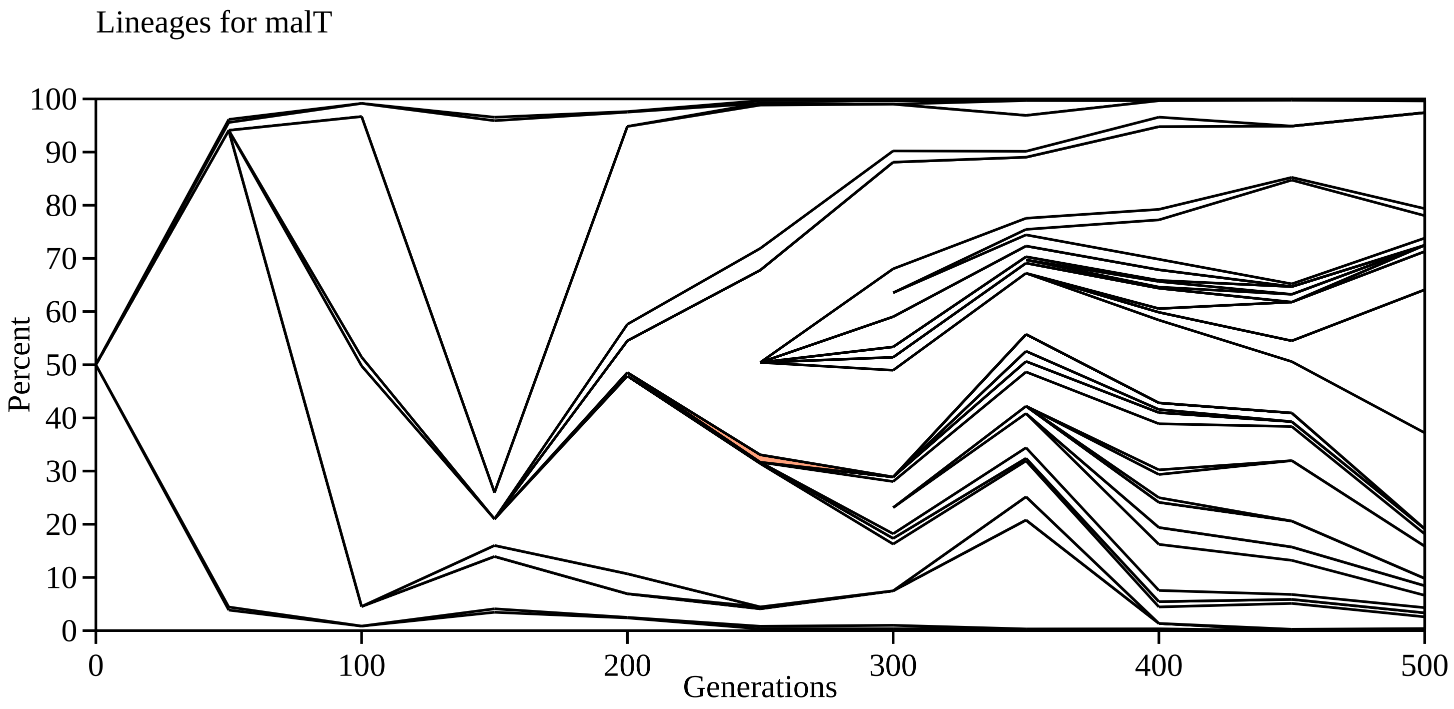

Lineages for malT
Percent
Generations
100
90
80
70
60
50
40
30
20
10
0
0
100
200
300
400
500

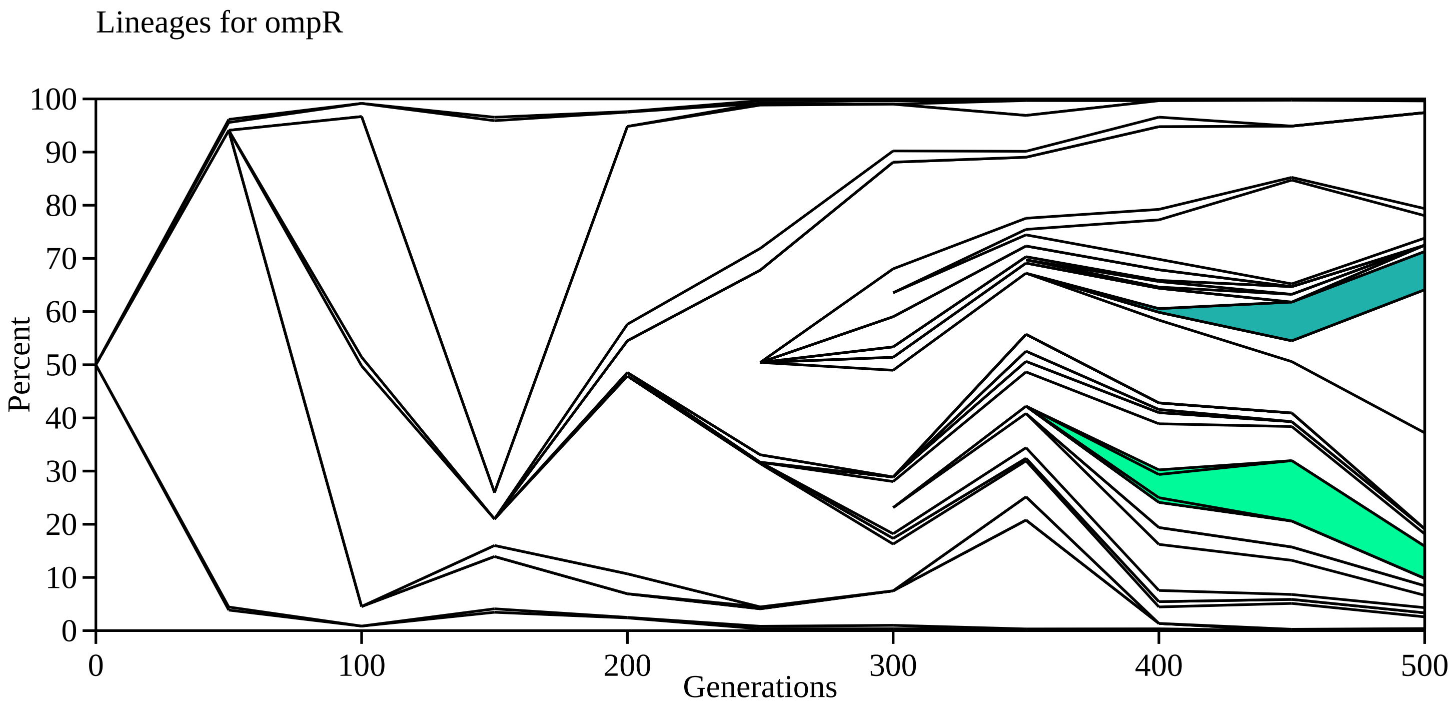
Lineages for ompR
Percent
Generations
100
90
80
70
60
50
40
30
20
10
0
0
100
200
300
400
500

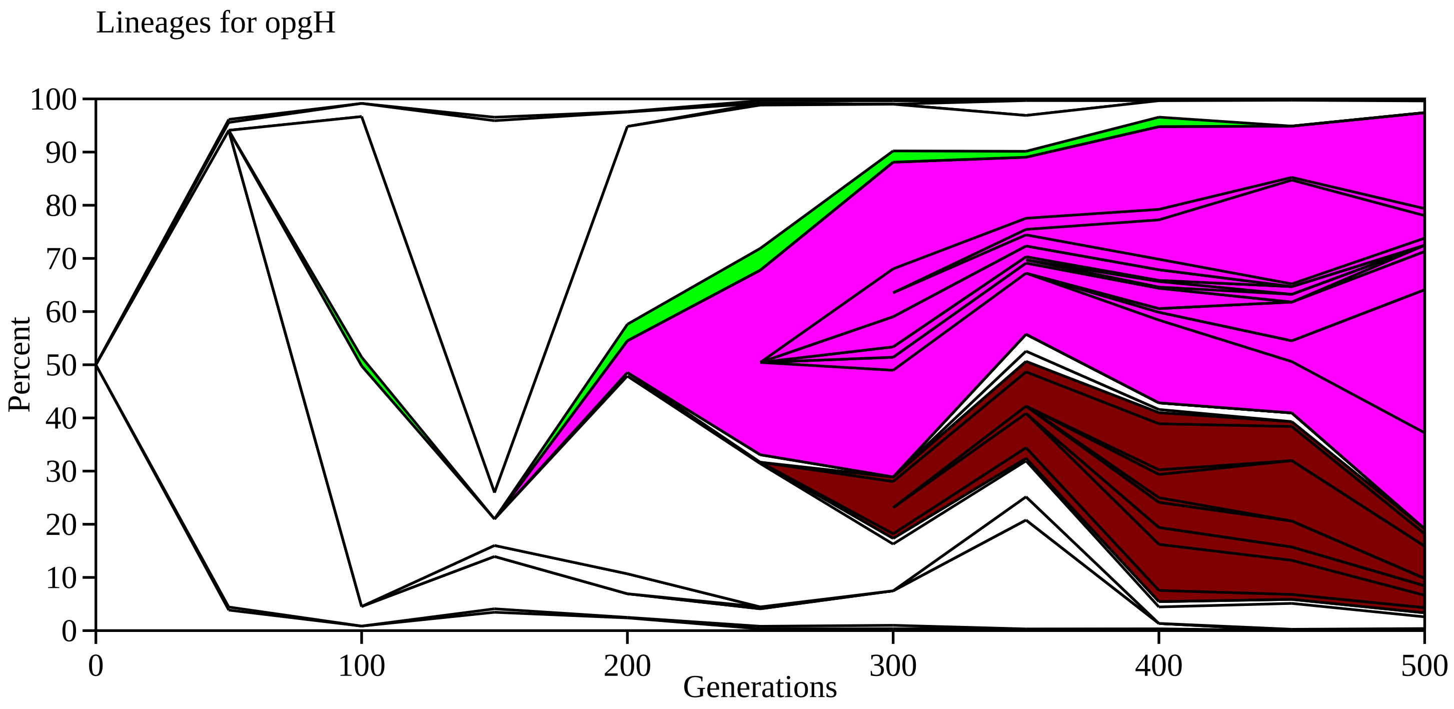
Lineages for opgH
Percent
100
90
80
70
60
50
40
30
20
10
0
0
100
200
300
400
500
Generations

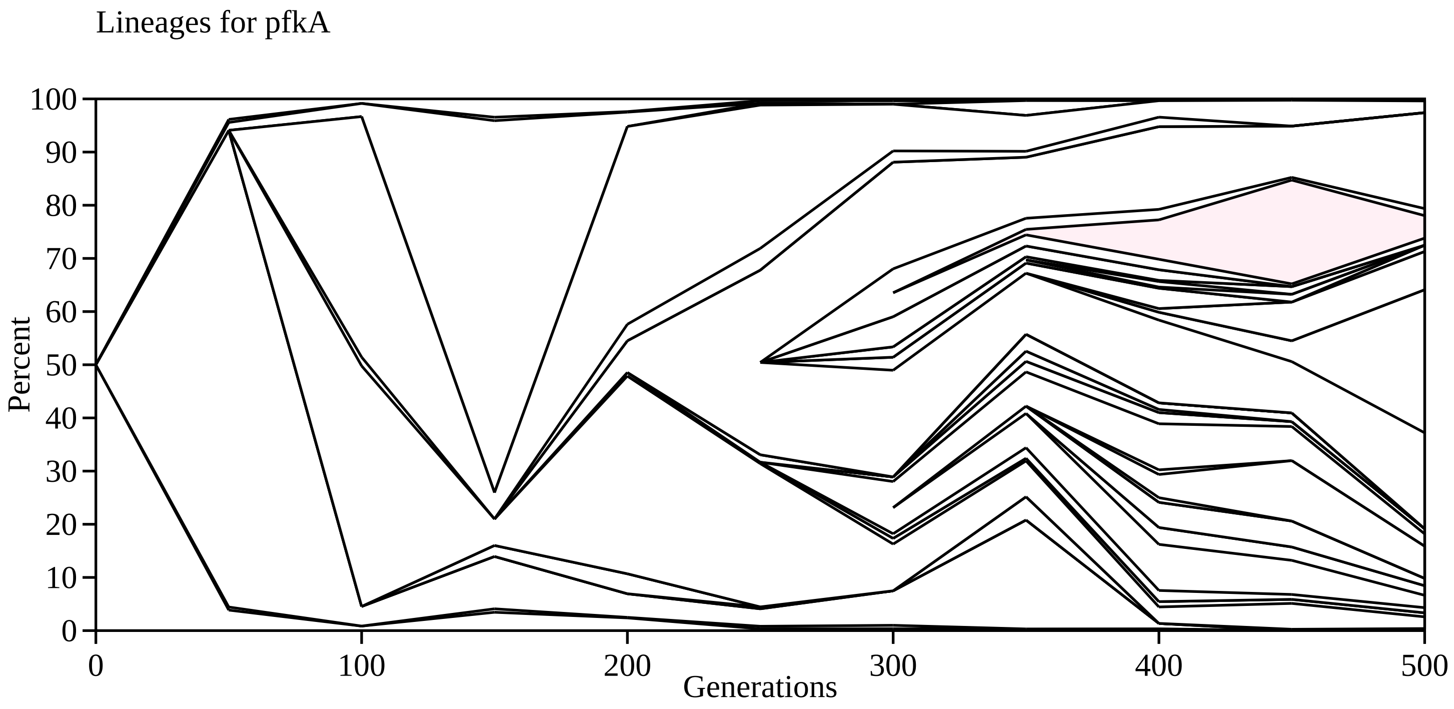

Lineages for pfkA
Percent
0
10
20
30
40
50
60
70
80
90
100
0
100
200
300
400
500
Generations

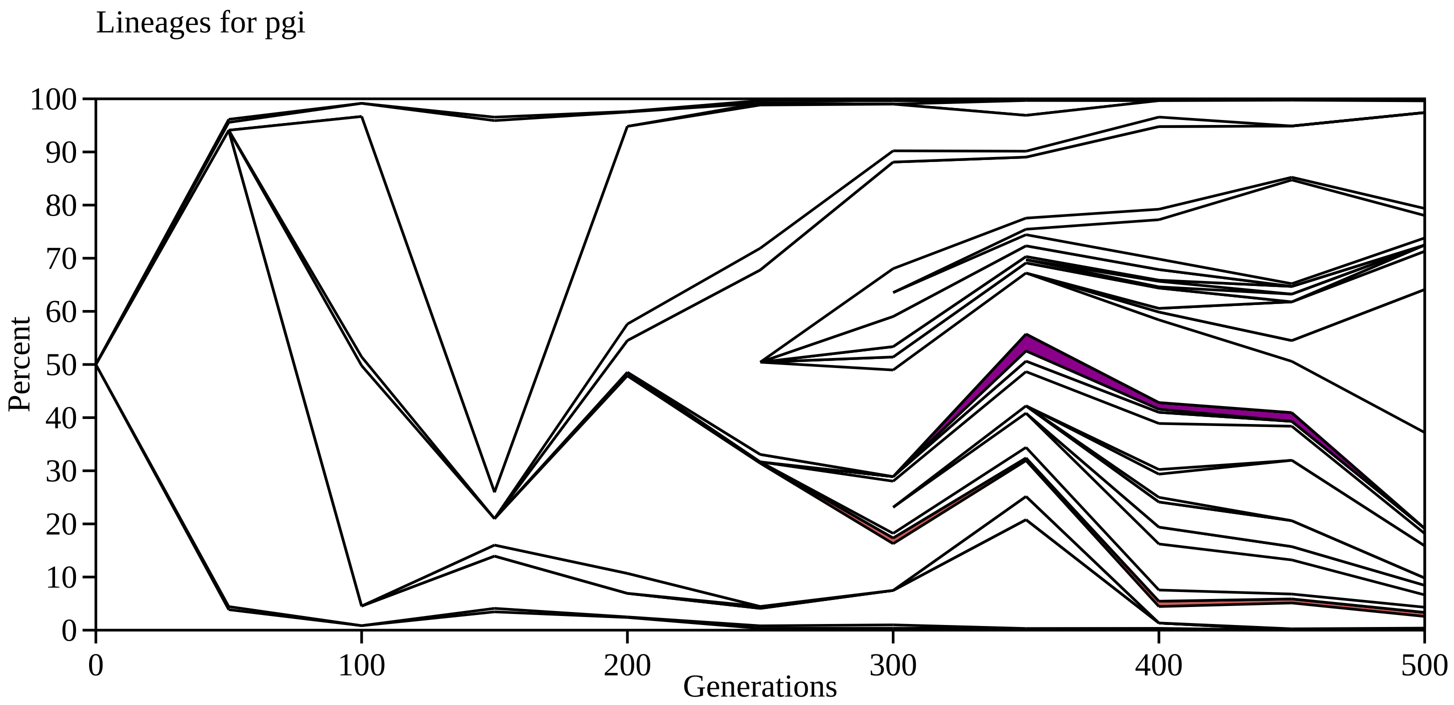

Lineages for pgi
Percent
Generations
0
10
20
30
40
50
60
70
80
90
100
0
100
200
300
400
500

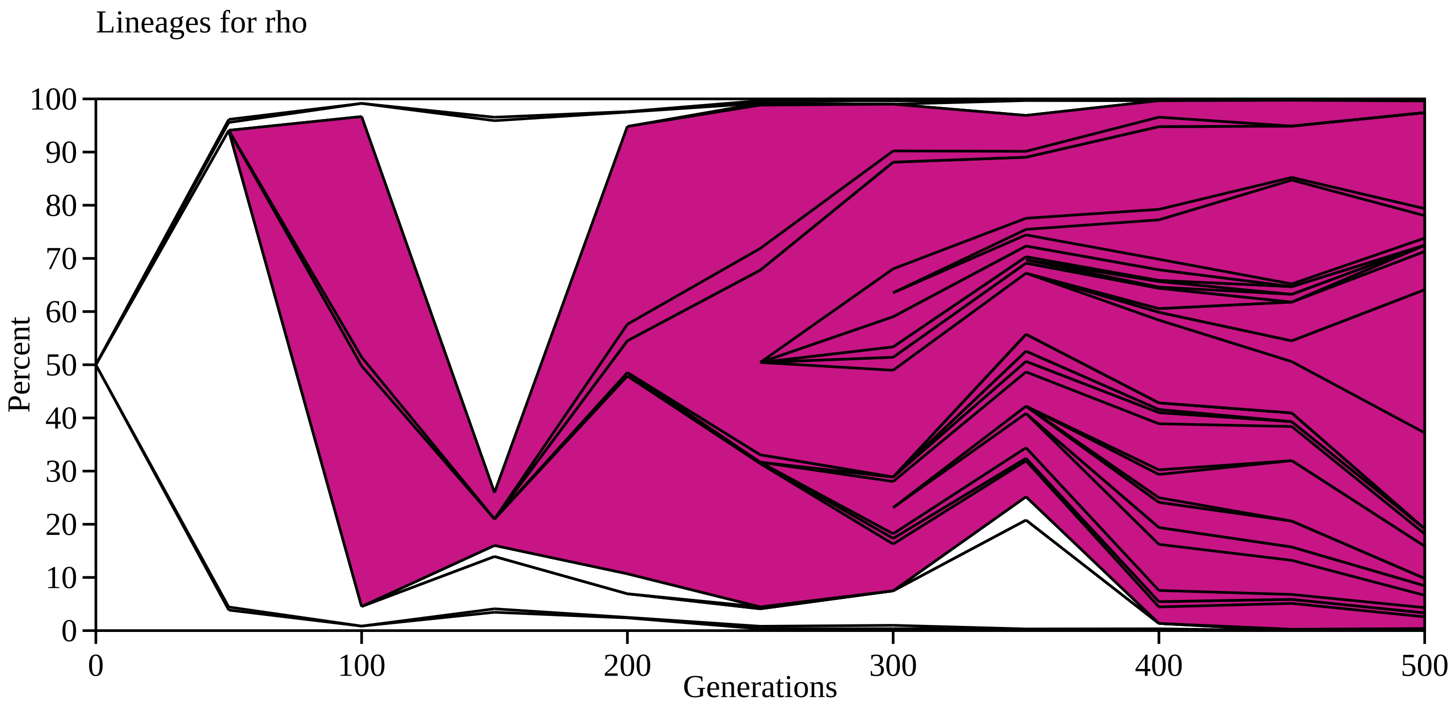

Lineages for rho
Percent
100
90
80
70
60
50
40
30
20
10
0
0
100
200
300
400
500
Generations

Lineages for rpoA

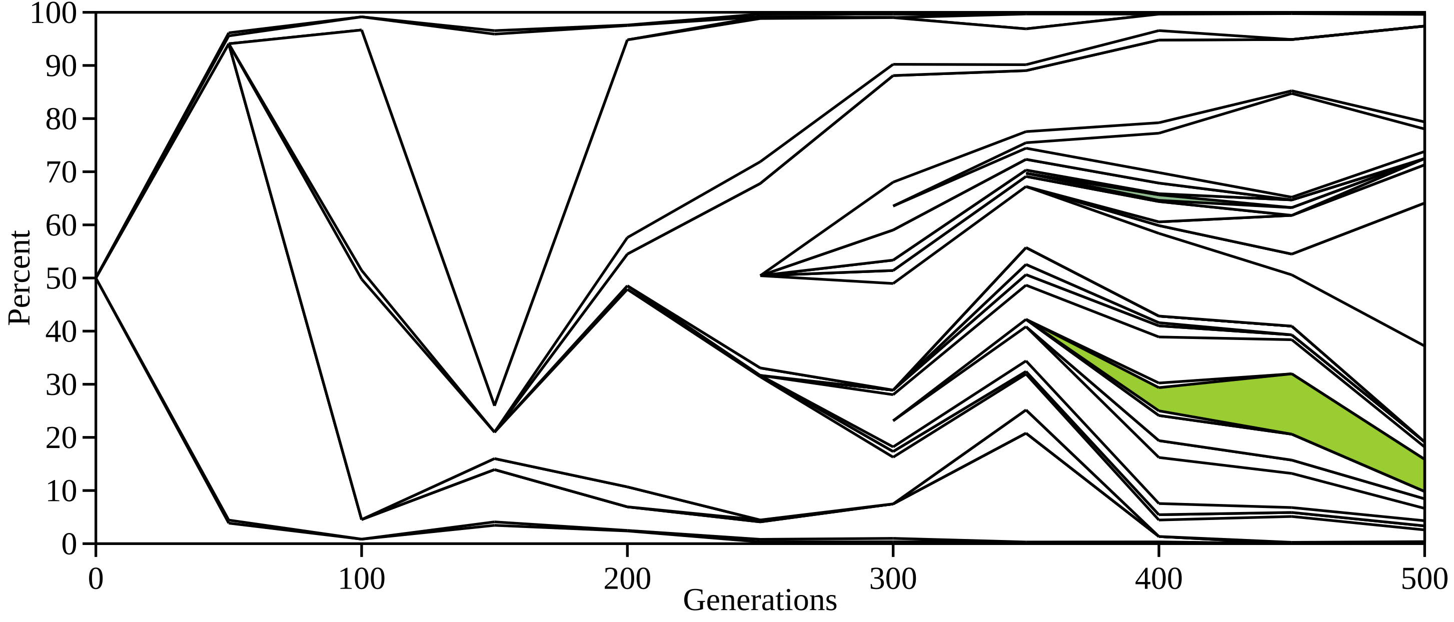

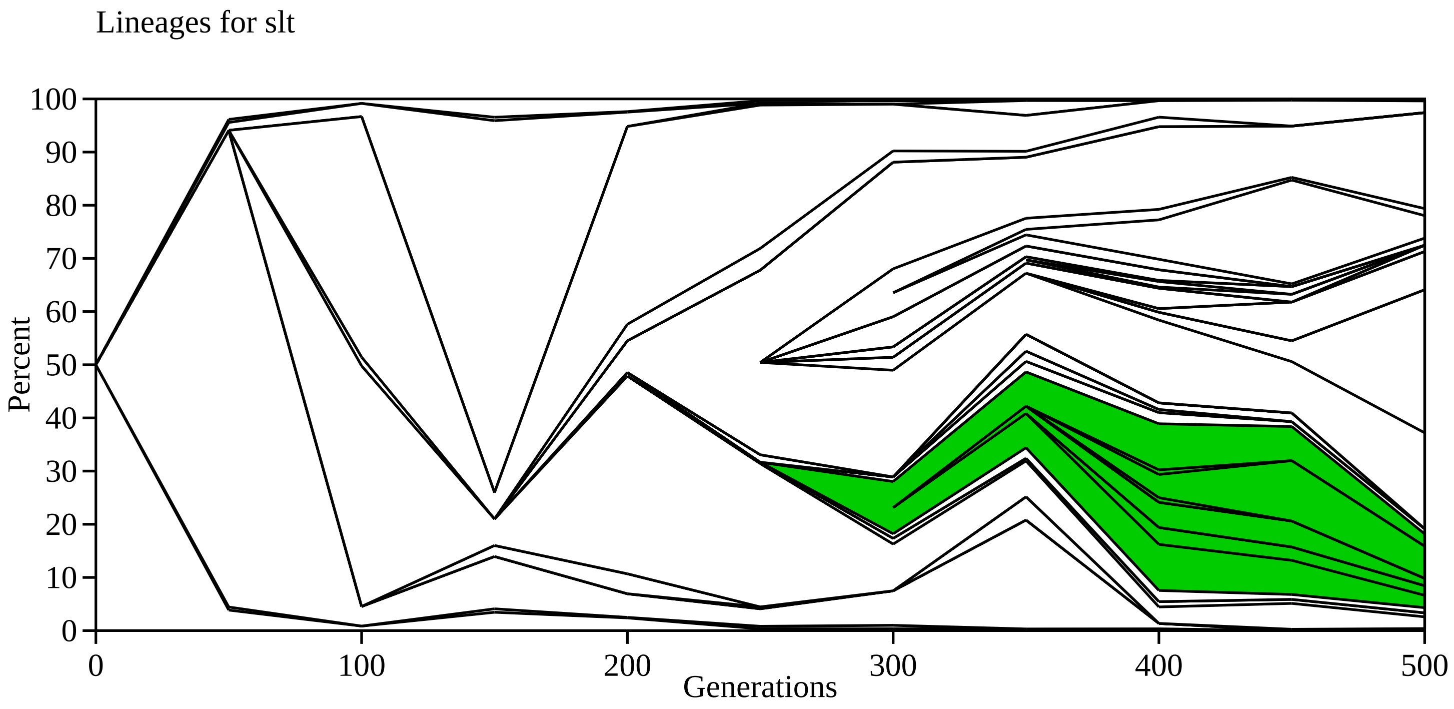
Lineages for slt
Percent
Generations
0
10
20
30
40
50
60
70
80
90
100
0
100
200
300
400
500

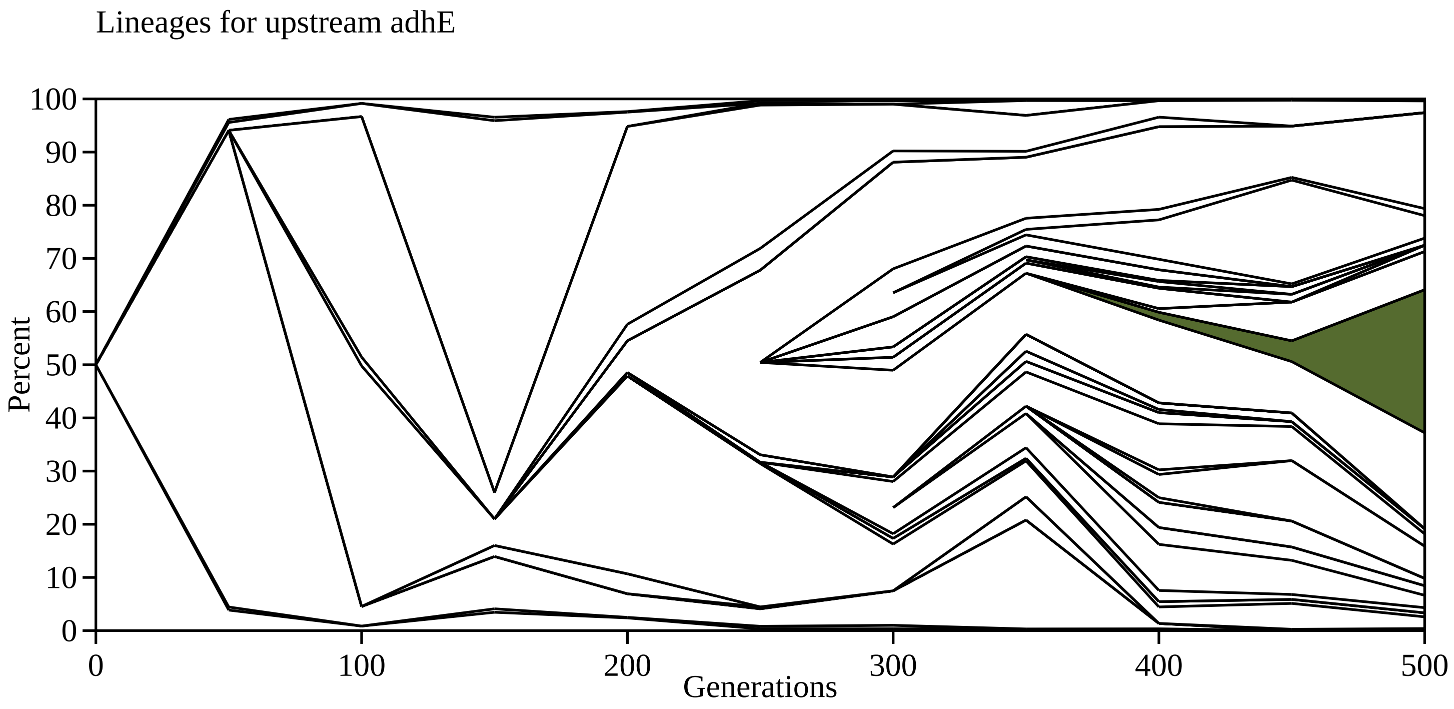

Lineages for upstream adhE
Percent
Generations
0
10
20
30
40
50
60
70
80
90
100
0
100
200
300
400
500

Lineages for upstream dnaG

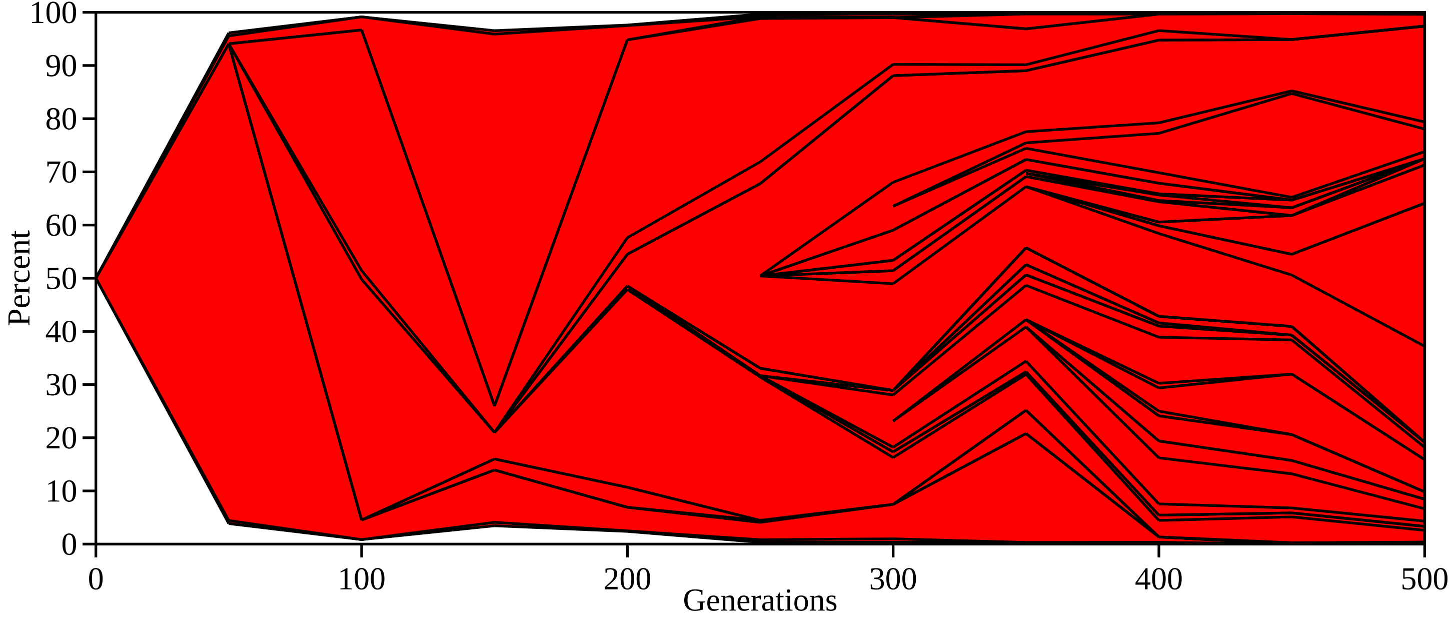

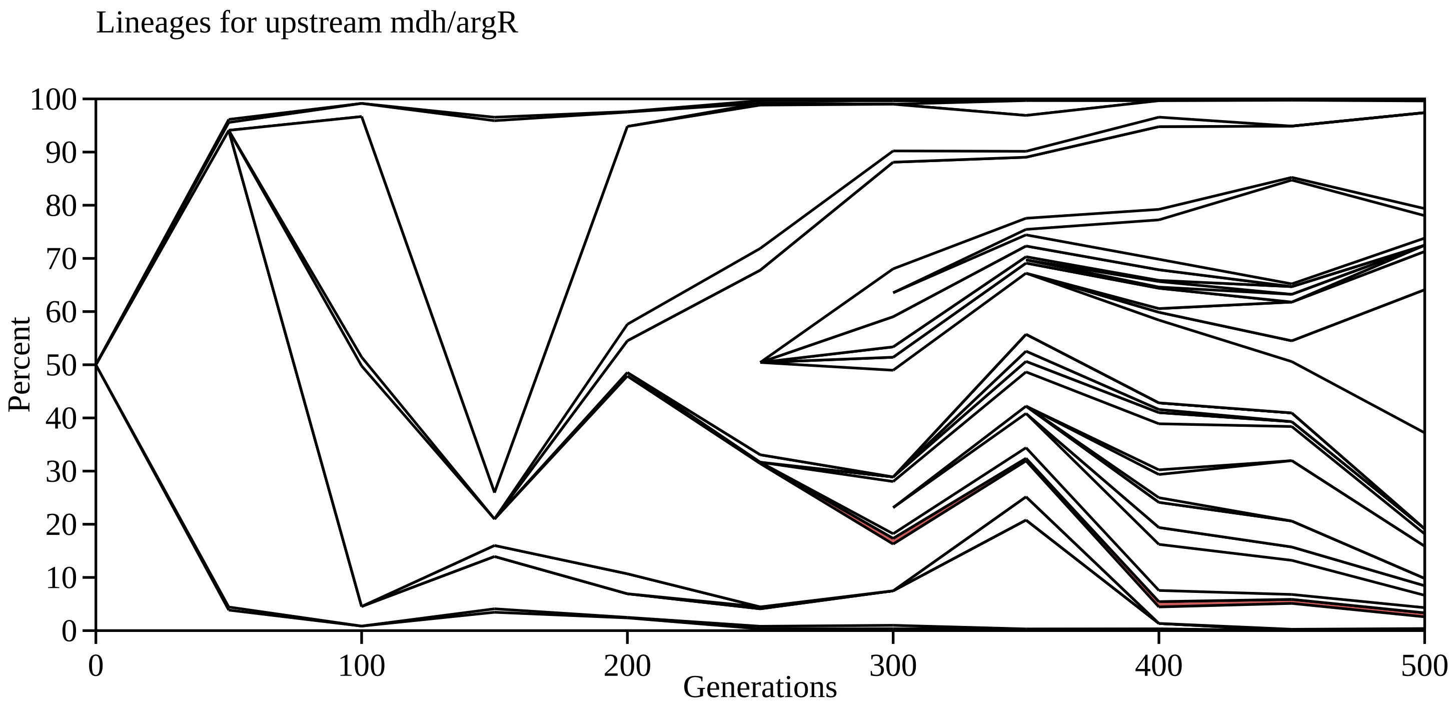

Lineages for upstream mdh/argR
Percent
0
10
20
30
40
50
60
70
80
90
100
0
100
200
300
400
500
Generations

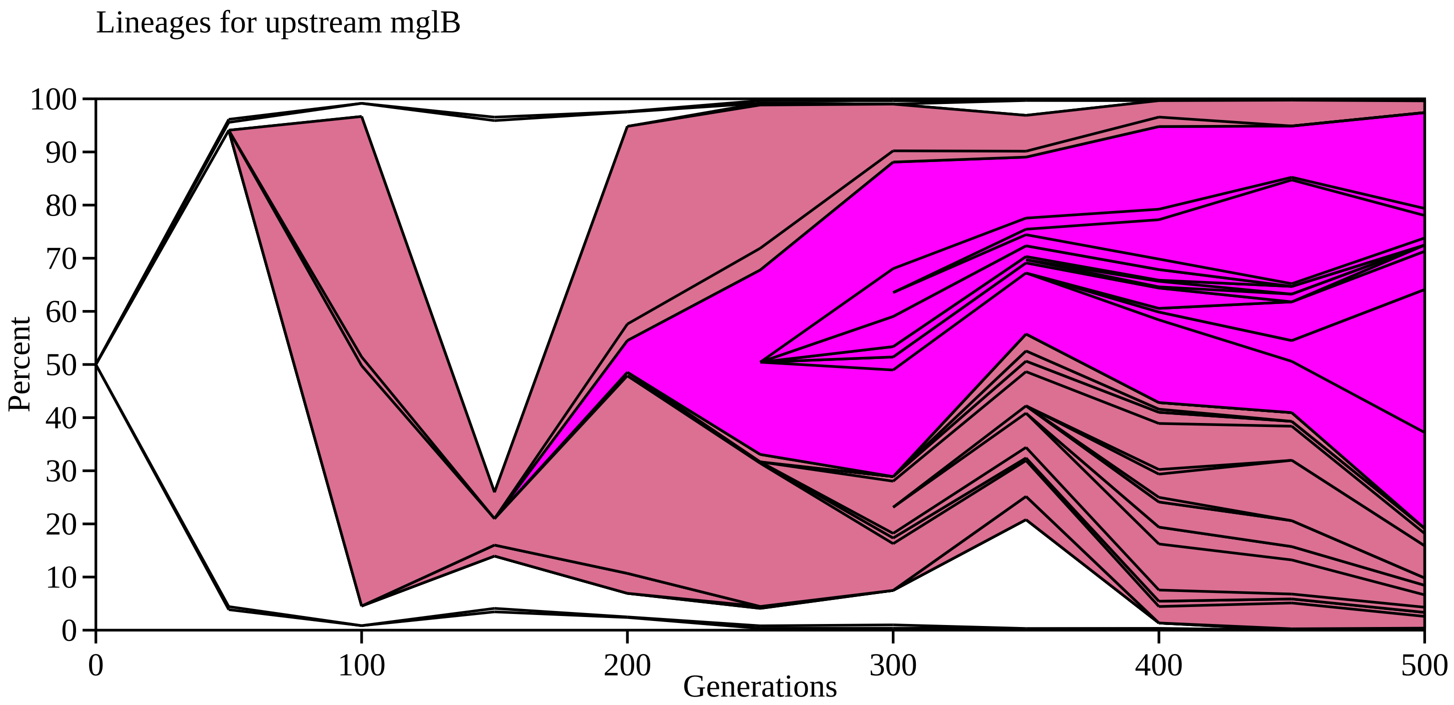

Lineages for upstream mglB
Percent
0
10
20
30
40
50
60
70
80
90
100
0
100
200
300
400
500
Generations

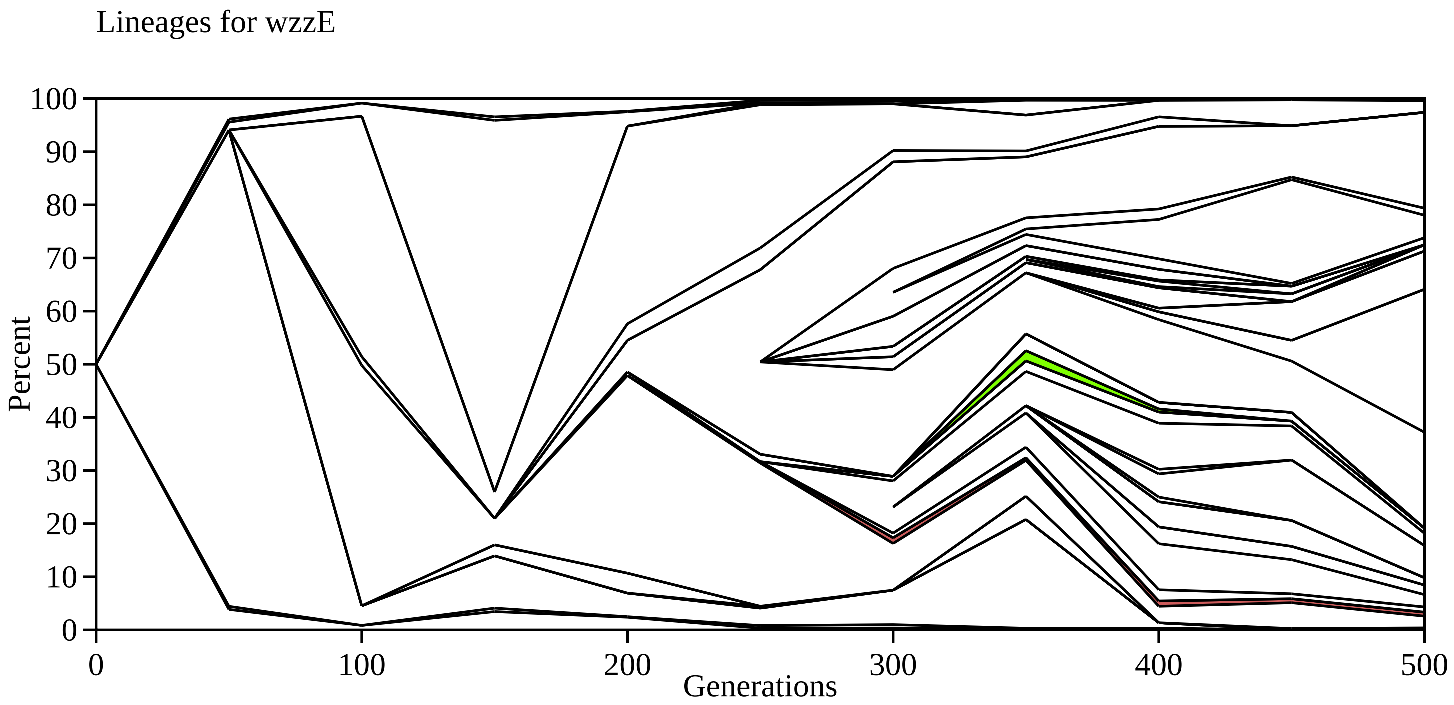

Lineages for wzzE
Percent
Generations
0
10
20
30
40
50
60
70
80
90
100
0
100
200
300
400
500

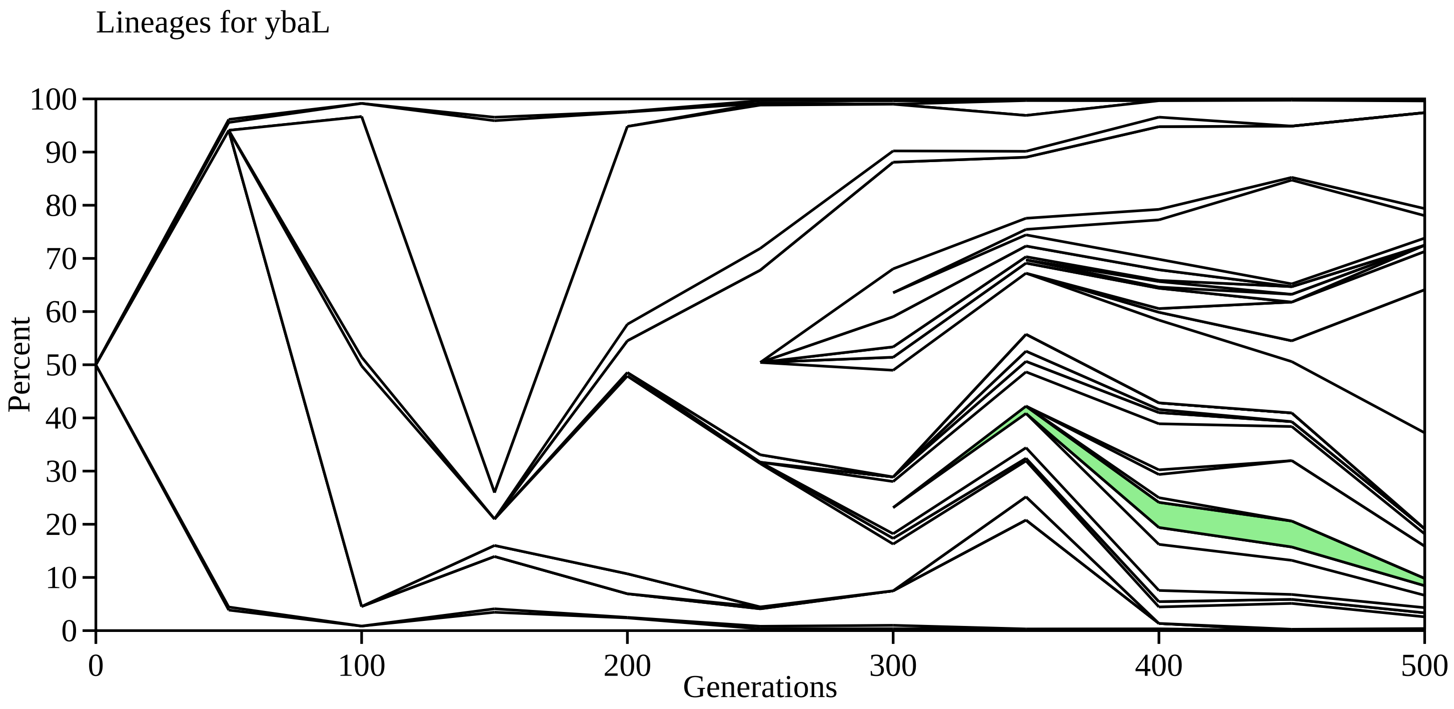

Lineages for ybaL
Percent
0
10
20
30
40
50
60
70
80
90
100
0
100
200
300
400
500
Generations

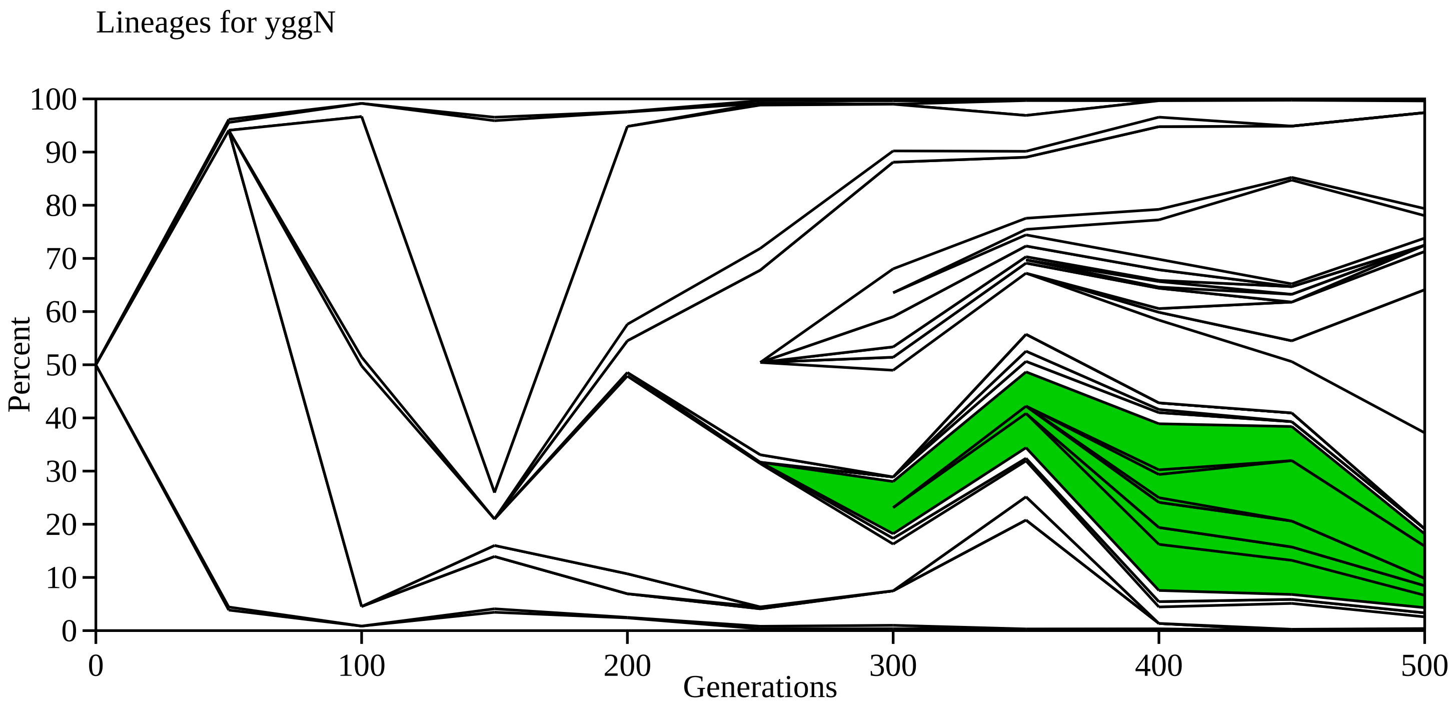
Lineages for yggN
Percent
Generations
0
10
20
30
40
50
60
70
80
90
100
0
100
200
300
400
500

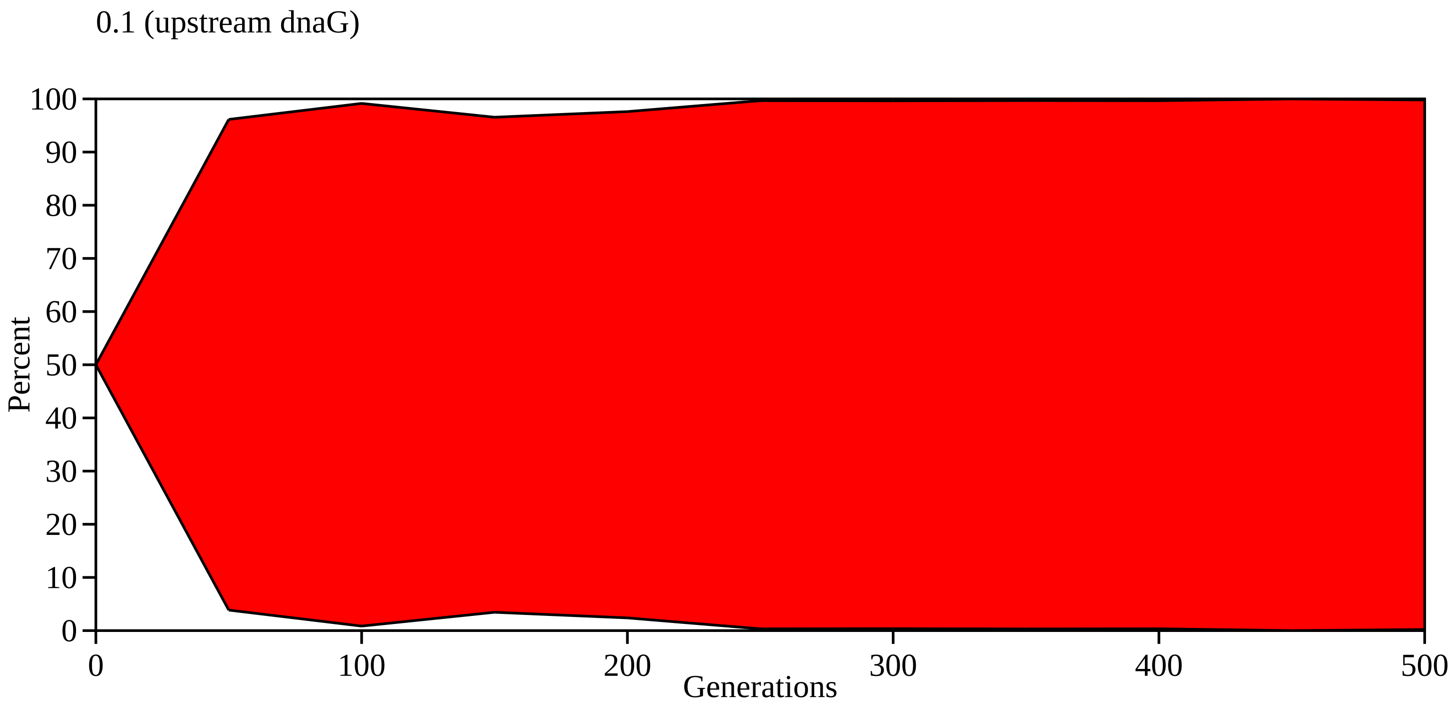
0.1 (upstream dnaG)
Percent
100
90
80
70
60
50
40
30
20
10
0
0
100
200
300
400
500
Generations

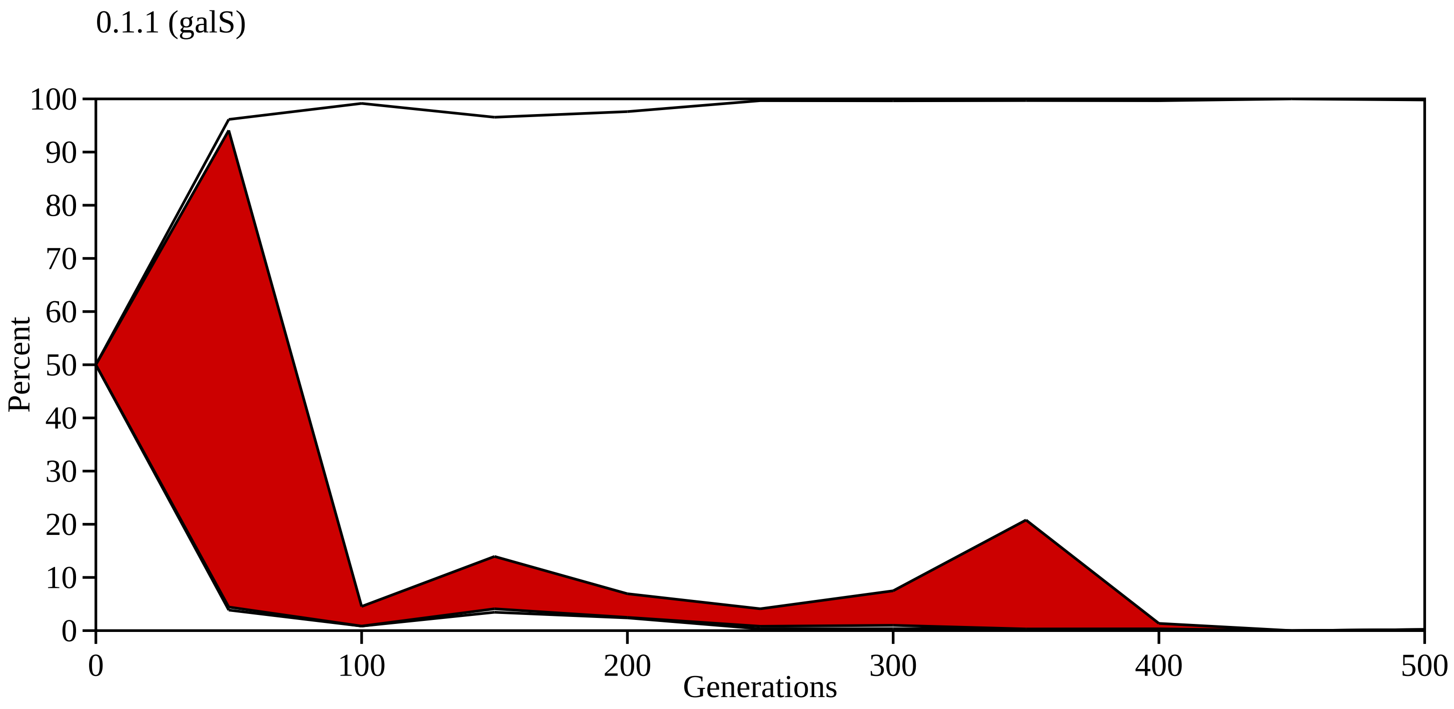
0.1.1 (galS)
Percent
100
90
80
70
60
50
40
30
20
10
0
0
100
200
300
400
500
Generations

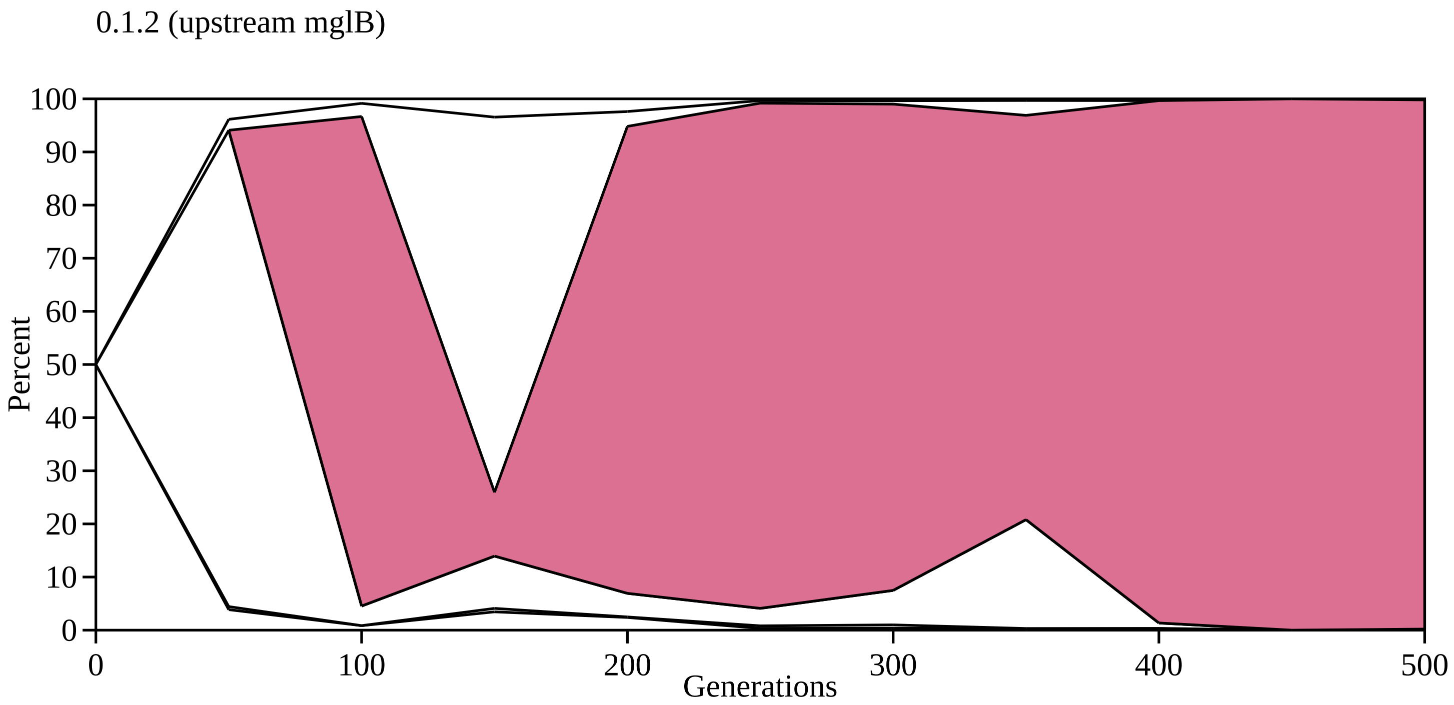
0.1.2 (upstream mglB)
100
90
80
70
60
50
40
30
20
10
0
Percent
0
100
200
300
400
500
Generations

0.1.3 (fimH)

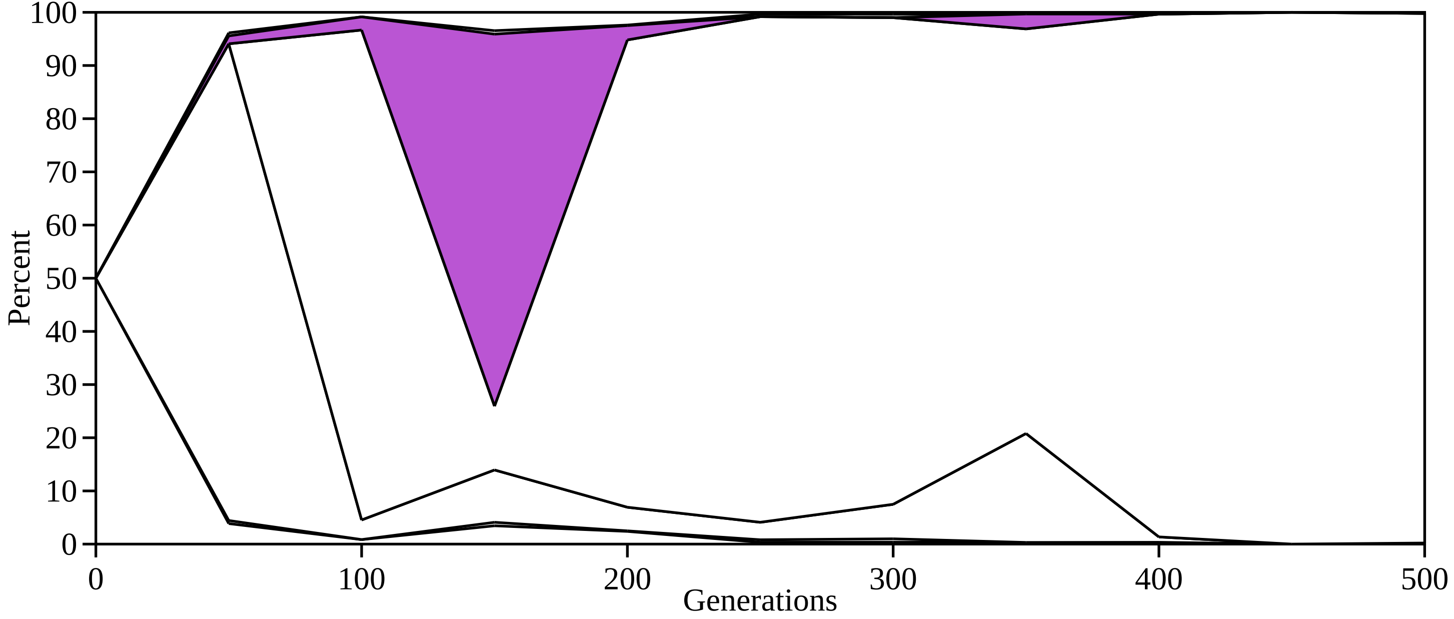

100
90
80
70
60
50
40
30
20
10
0
Percent
0
100
200
300
400
500
Generations

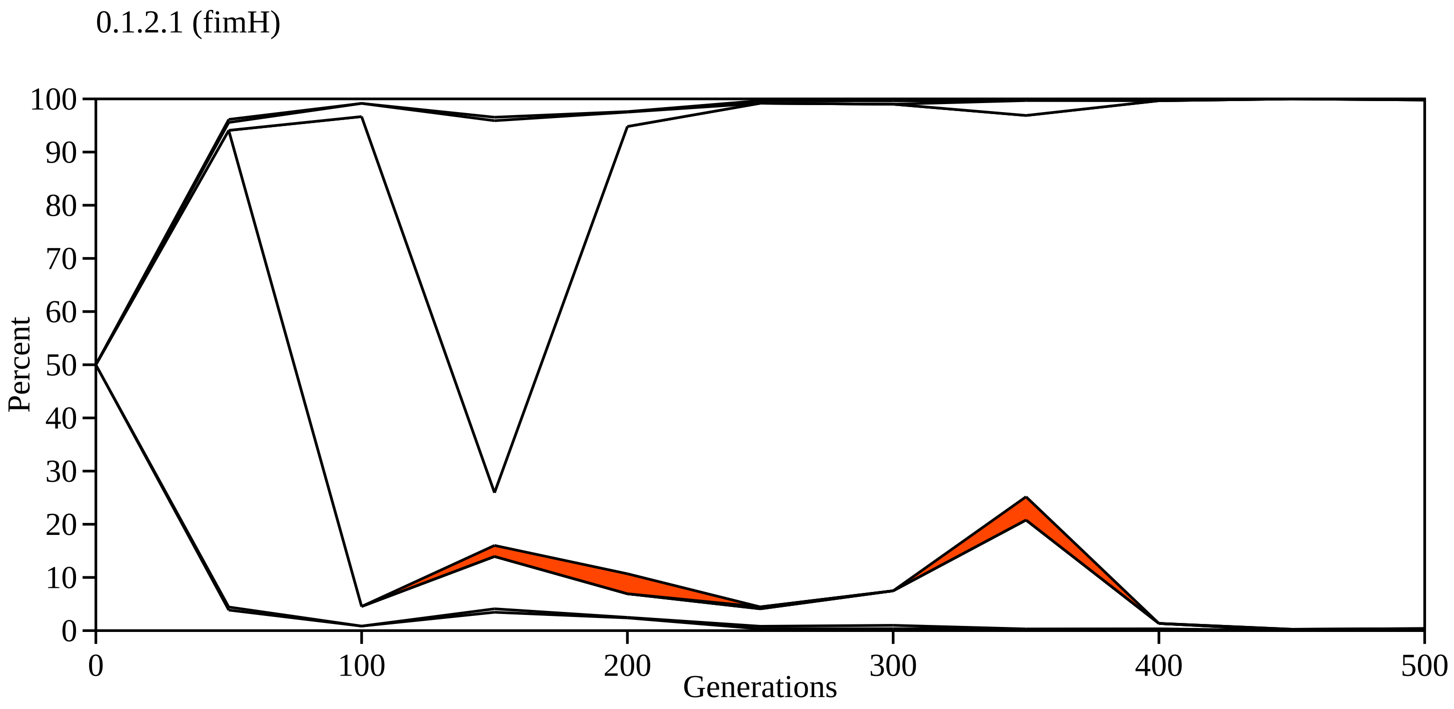

0.1.2.1 (fimH)
Percent
100
90
80
70
60
50
40
30
20
10
0
0
100
200
300
400
500
Generations

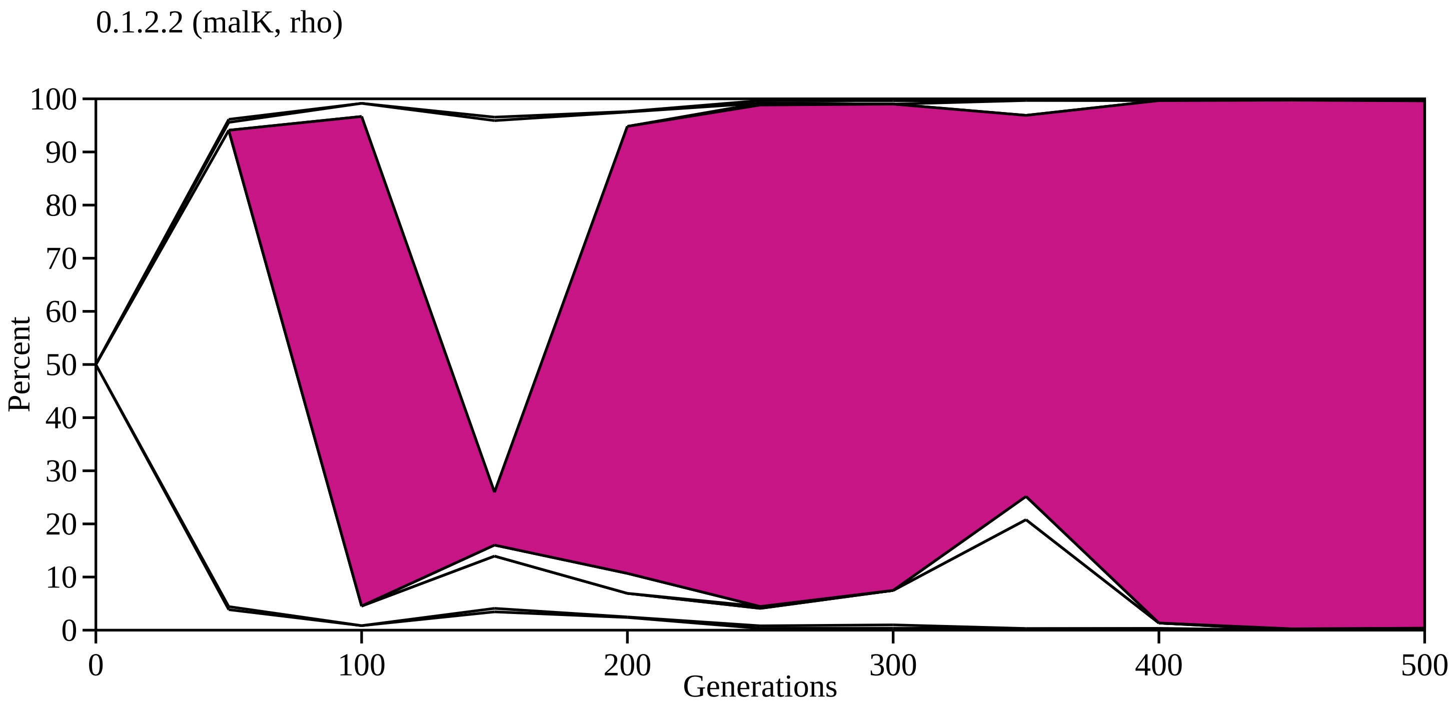

0.1.2.2 (malK, rho)
Percent
100
90
80
70
60
50
40
30
20
10
0
0
100
200
300
400
500
Generations

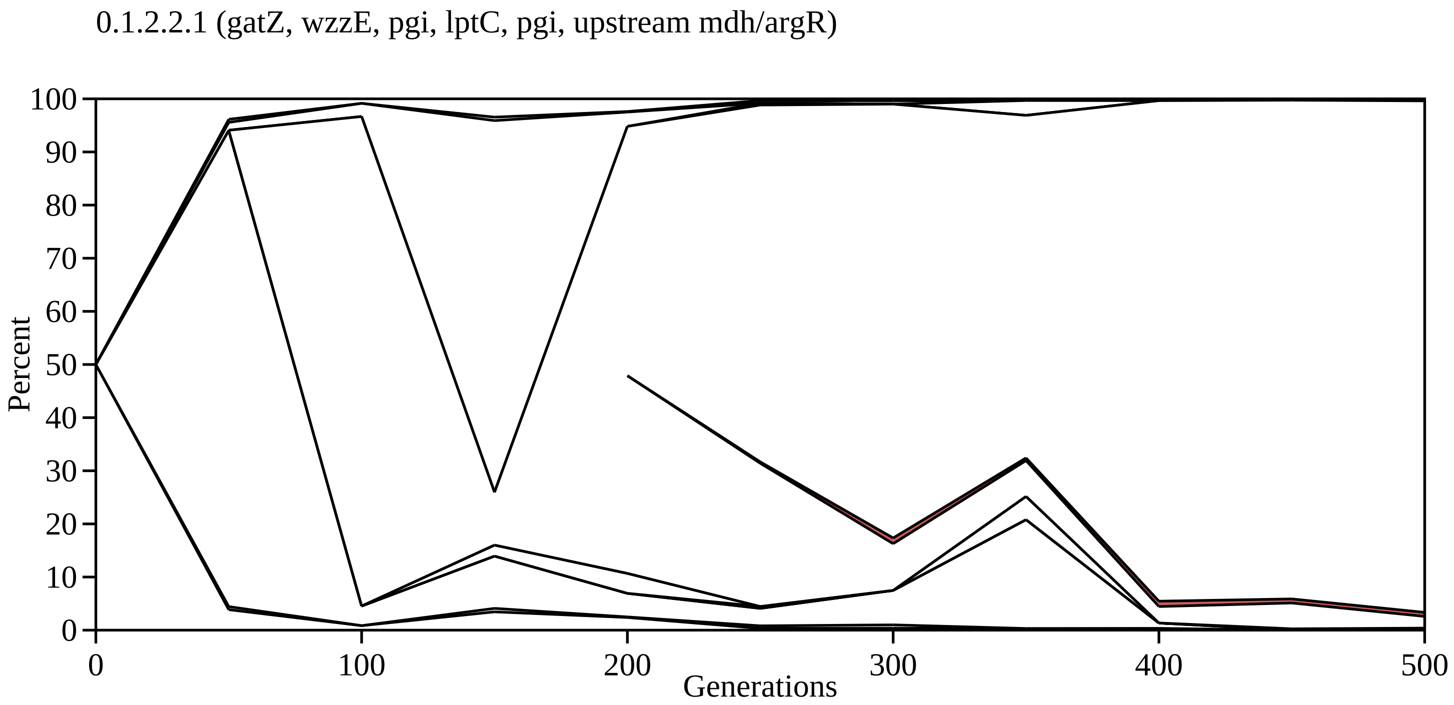
0.1.2.2.1 (gatZ, wzzE, pgi, lptC, pgi, upstream mdh/argR)
100
90
80
70
60
50
40
30
20
10
0
Percent
0
100
200
300
400
500
Generations

0.1.2.2.2 (opgH, lptD)

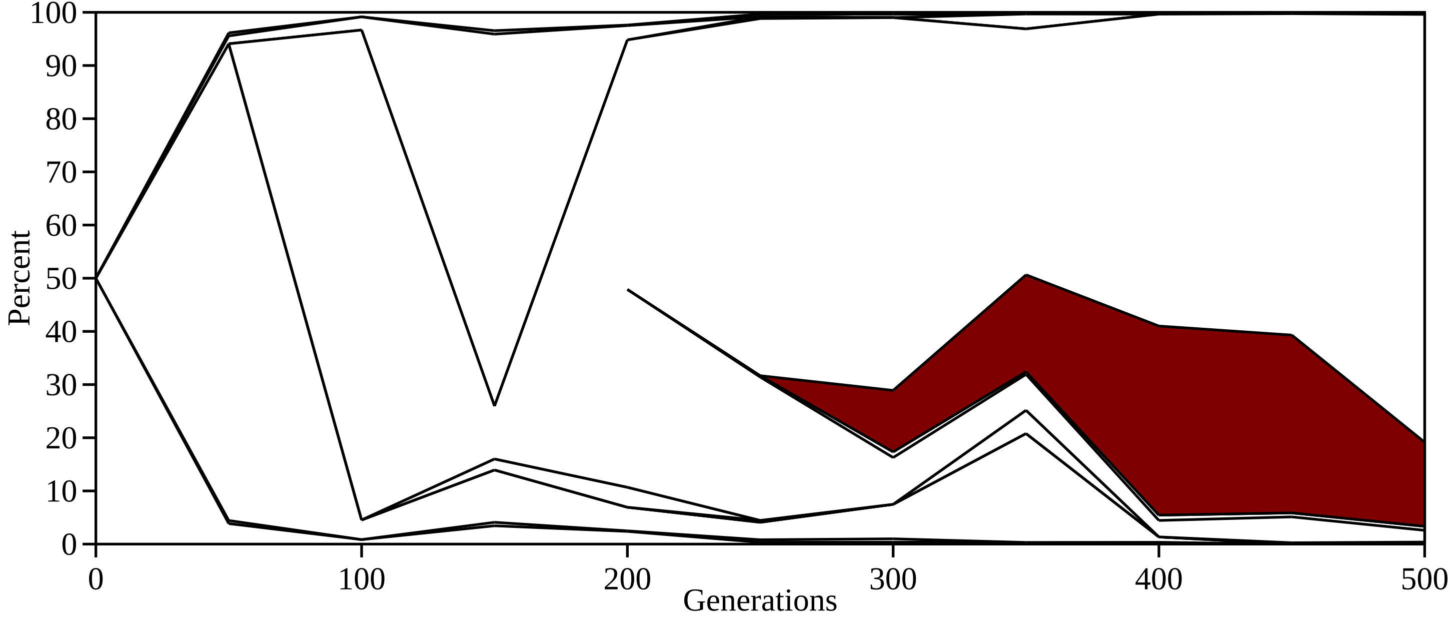

0.1.2.2.3 (fimH, wzzE)

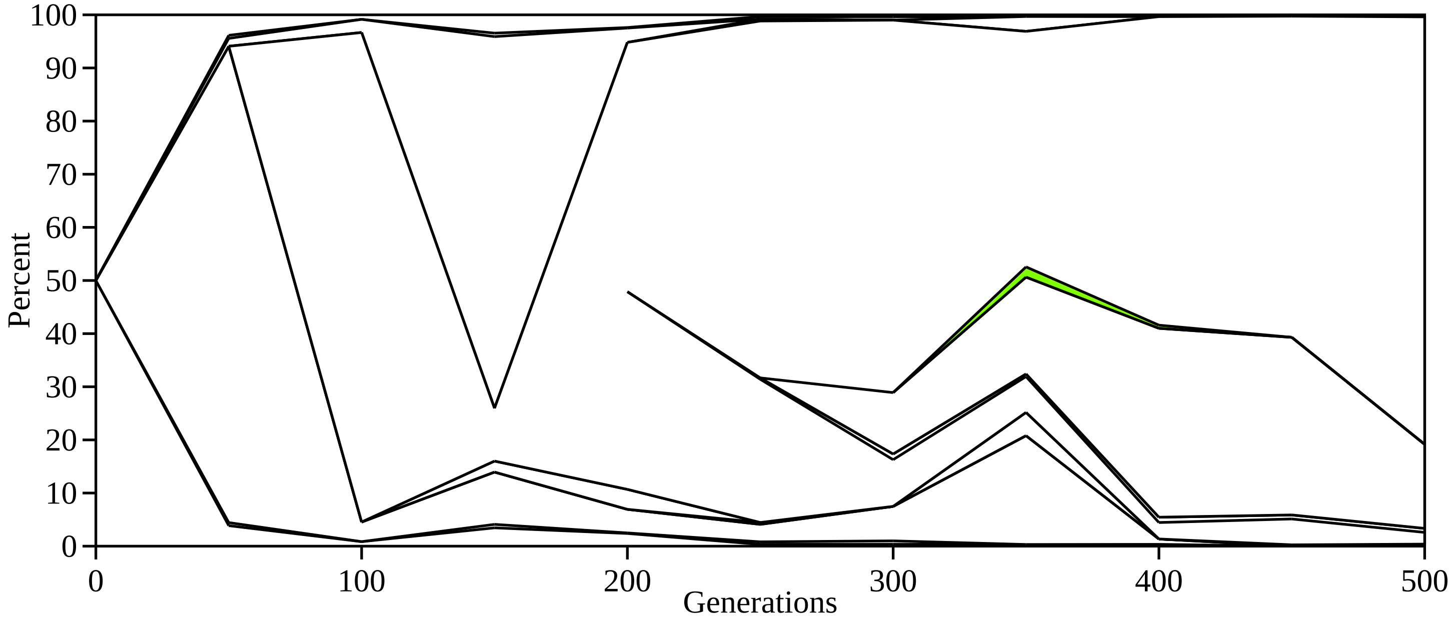

0.1.2.2.4 (lptA, pgi)

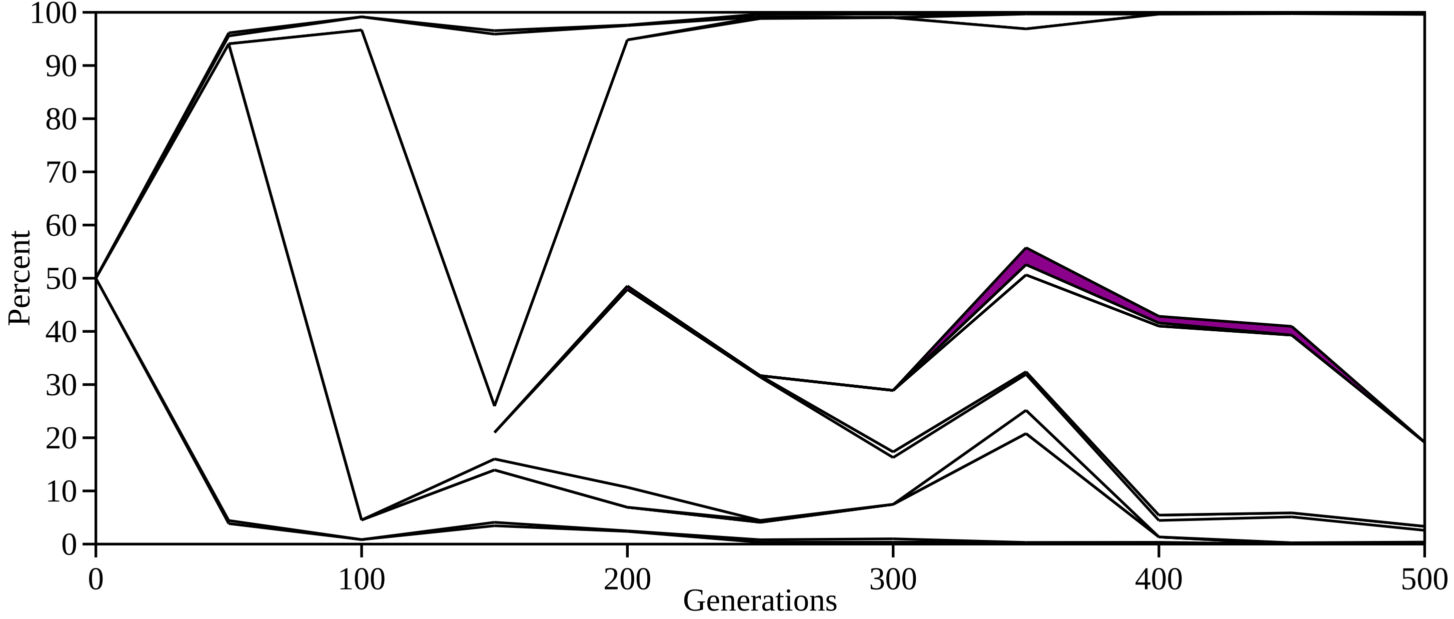

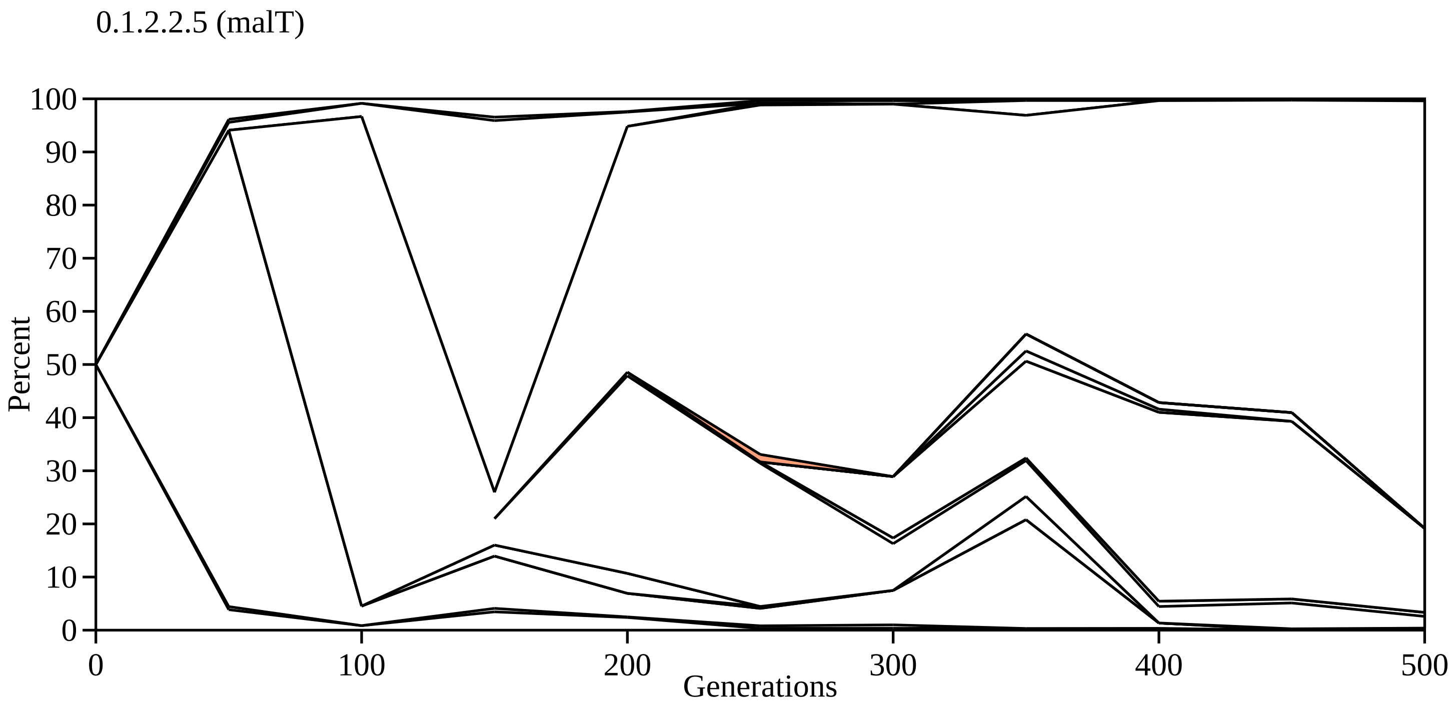
0.1.2.2.5 (malT)
100
90
80
70
60
50
40
30
20
10
0
Percent
0
100
200
300
400
500
Generations

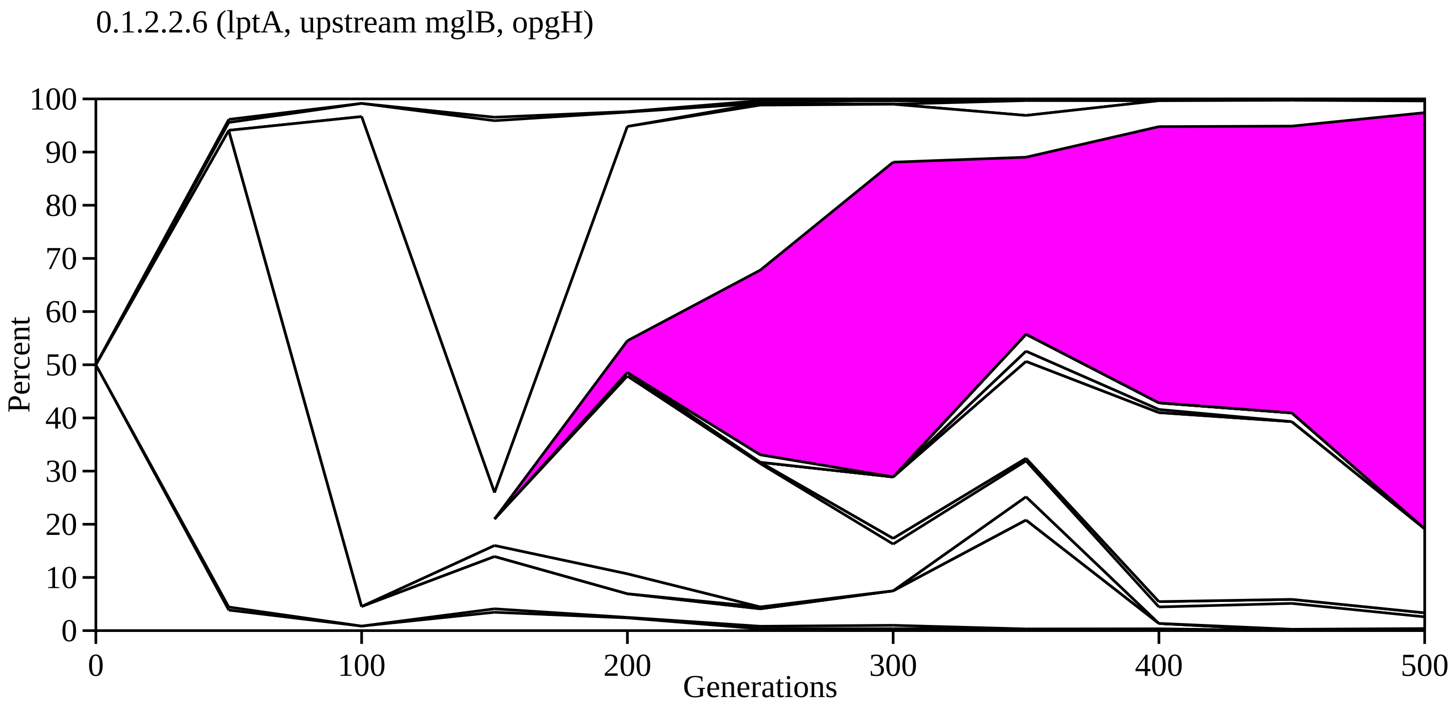
0.1.2.2.6 (lptA, upstream mglB, opgH)
Percent
100
90
80
70
60
50
40
30
20
10
0
0
100
200
300
400
500
Generations

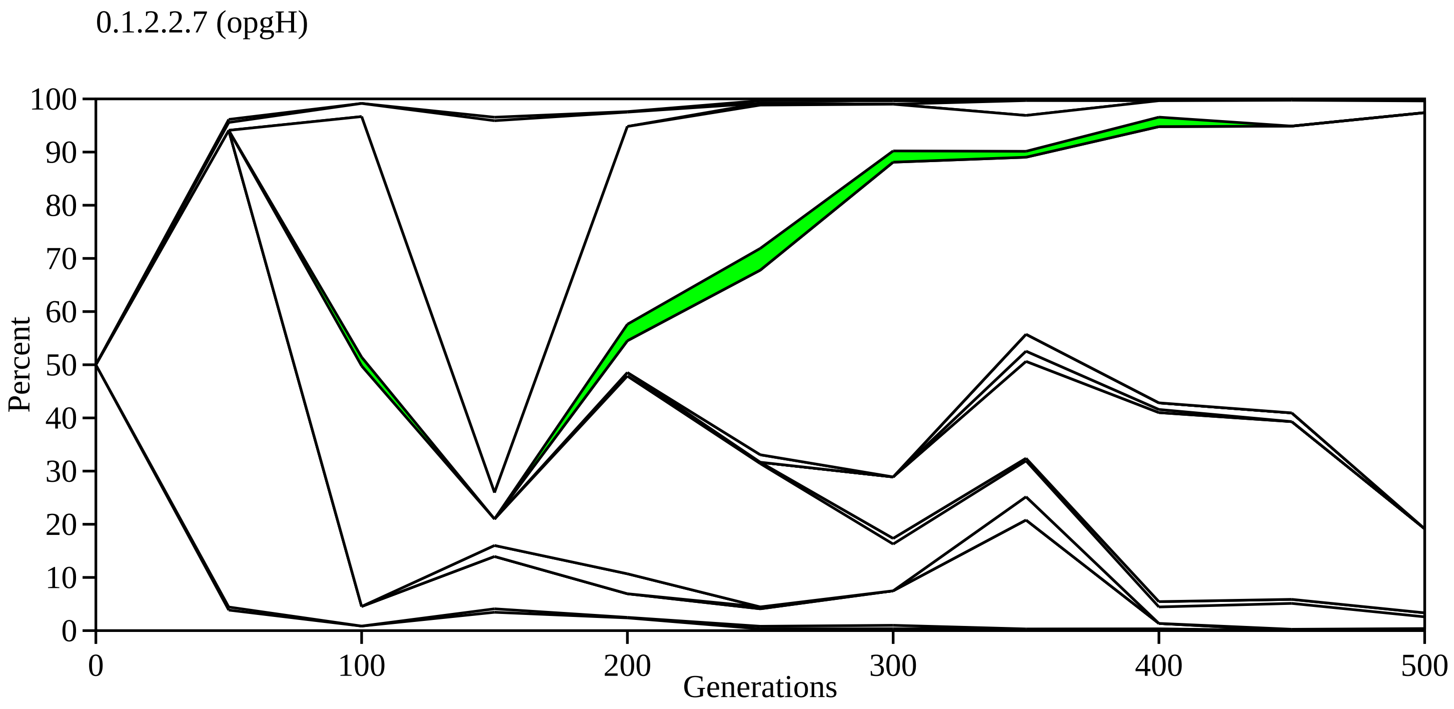
0.1.2.2.7 (opgH)
Percent
100
90
80
70
60
50
40
30
20
10
0
0
100
200
300
400
500
Generations

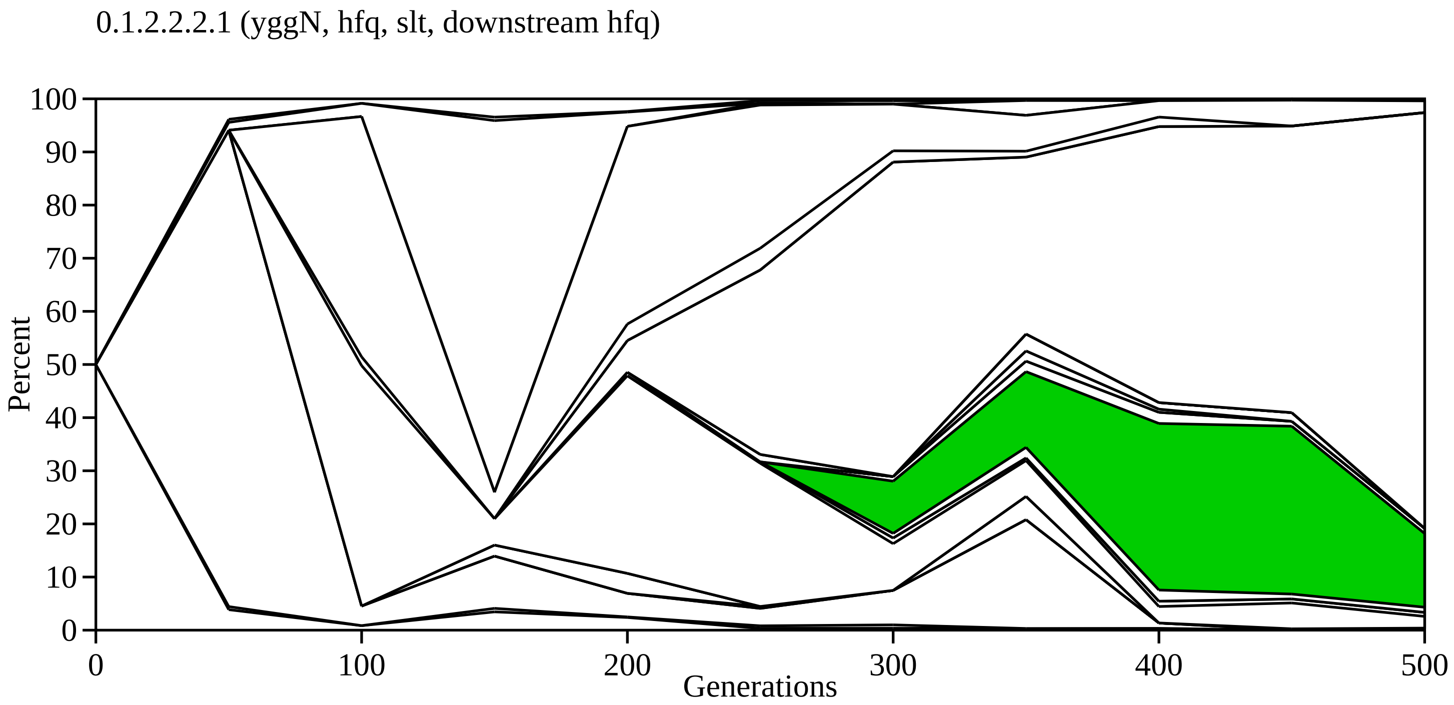
0.1.2.2.2.1 (yggN, hfq, slt, downstream hfq)
100
90
80
70
60
50
40
30
20
10
0
Percent
0
100
200
300
400
500
Generations

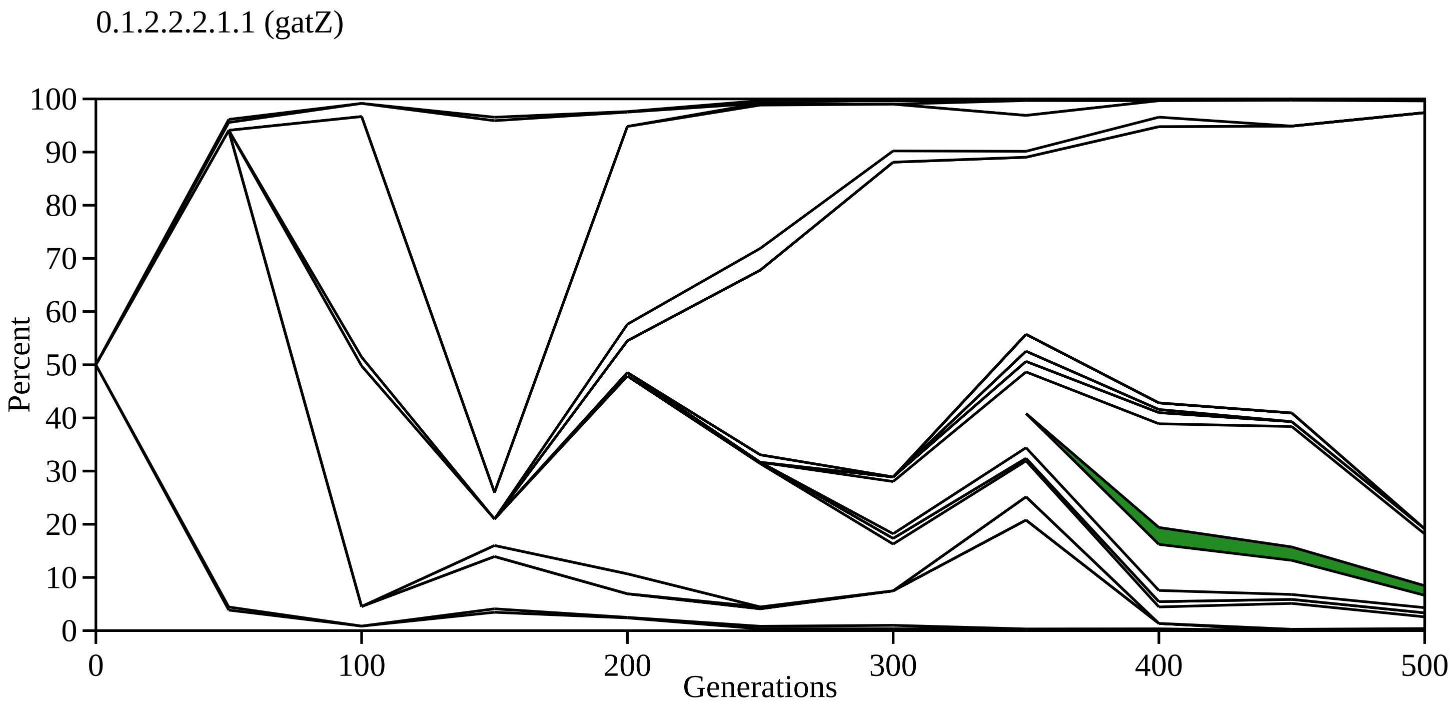
0.1.2.2.2.1.1 (gatZ)
Percent
0
10
20
30
40
50
60
70
80
90
100
0
100
200
300
400
500
Generations

0.1.2.2.2.1.2 (ybaL)

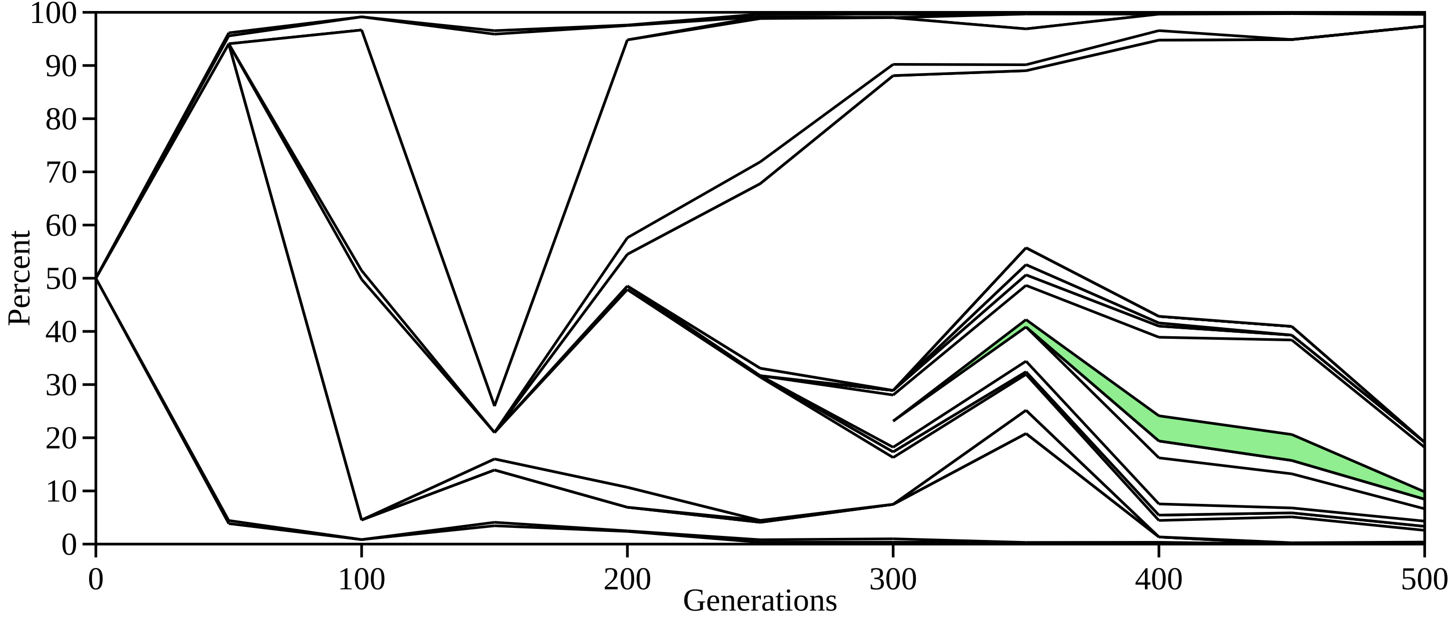

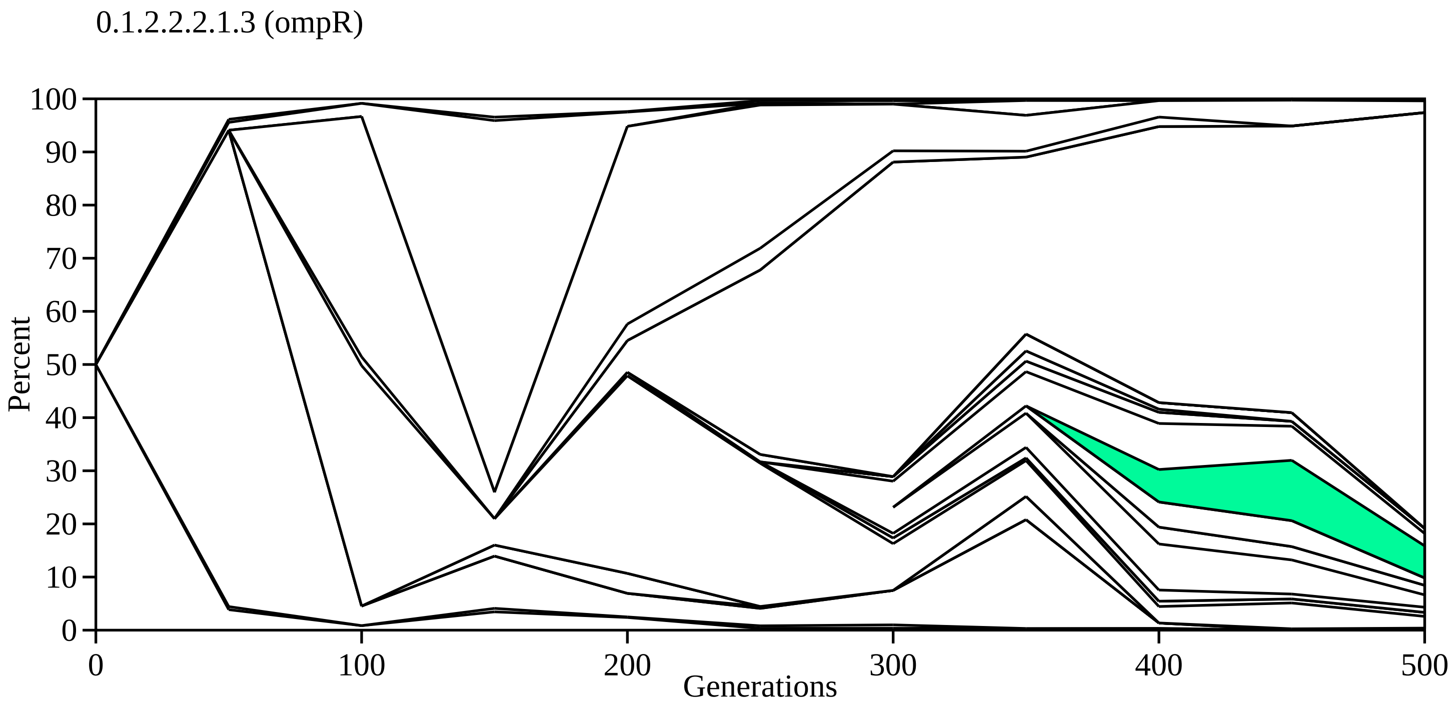
0.1.2.2.2.1.3 (ompR)
Percent
100
90
80
70
60
50
40
30
20
10
0
0
100
200
300
400
500
Generations

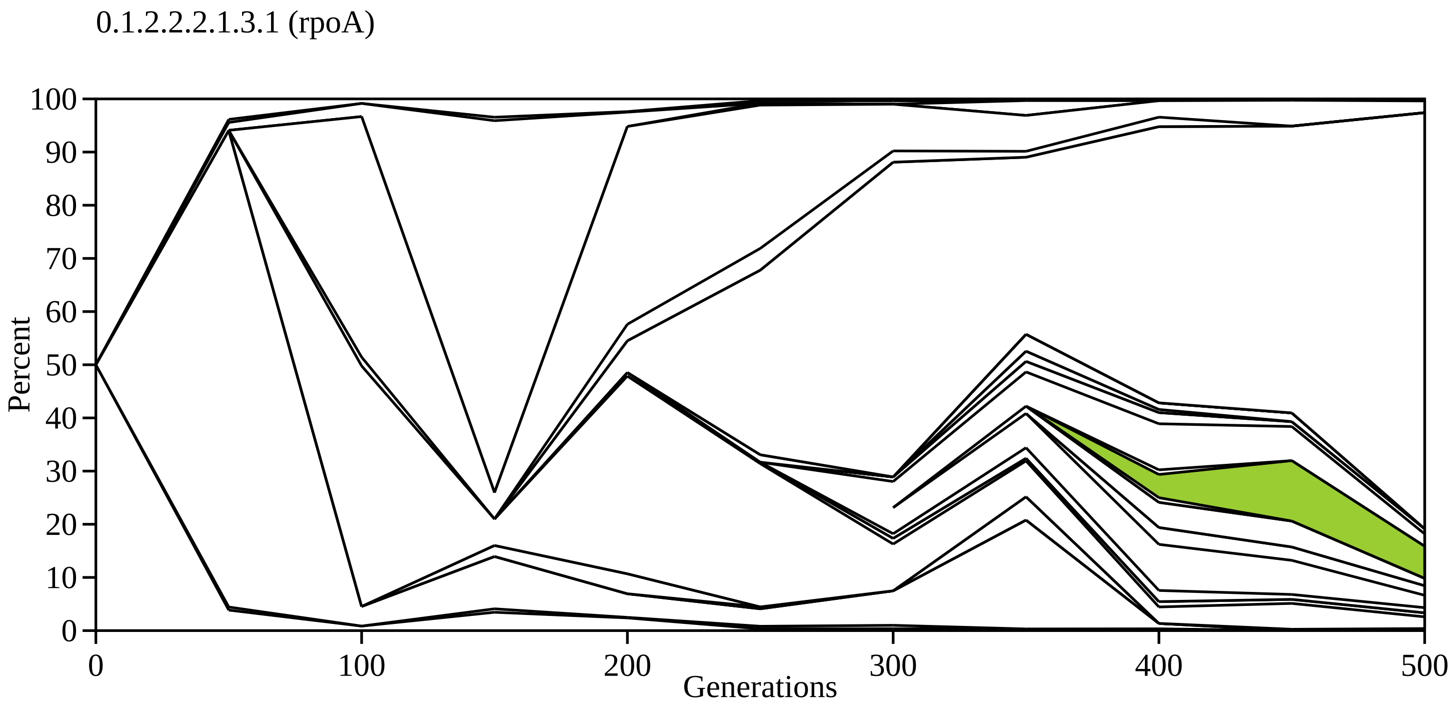
0.1.2.2.2.1.3.1 (rpoA)
Percent
100
90
80
70
60
50
40
30
20
10
0
0
100
200
300
400
500
Generations

0.1.2.2.6.1 (upstream adhE)

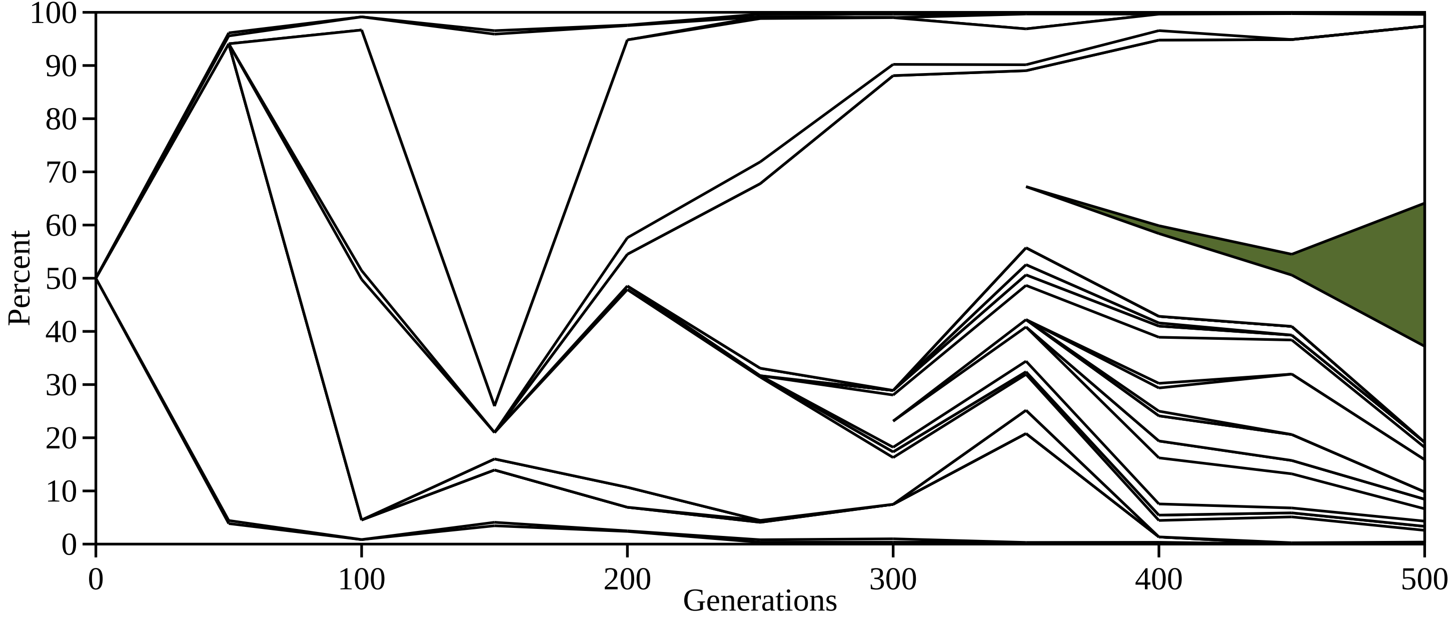

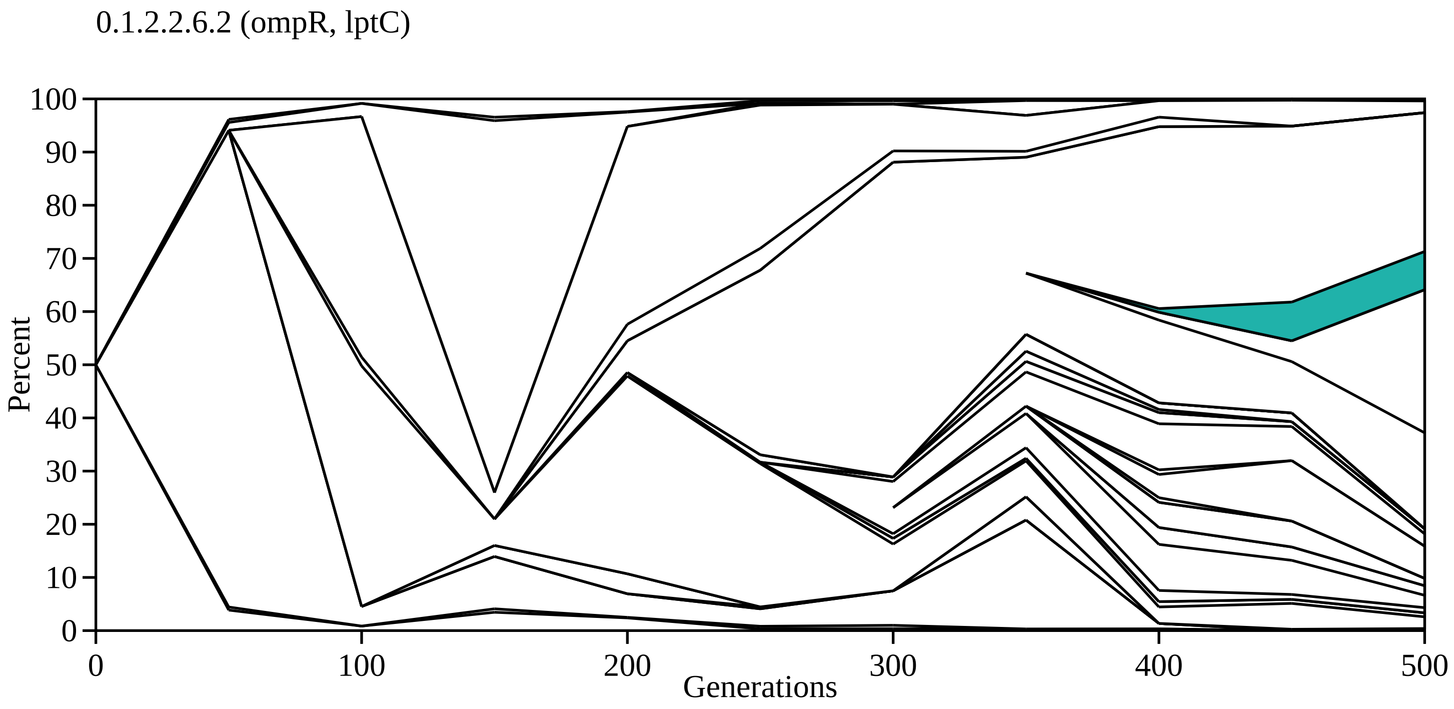

0.1.2.2.6.2 (ompR, lptC)
100
90
80
70
60
50
40
30
20
10
0
Percent
0
100
200
300
400
500
Generations

0.1.2.2.6.3 (hfq)

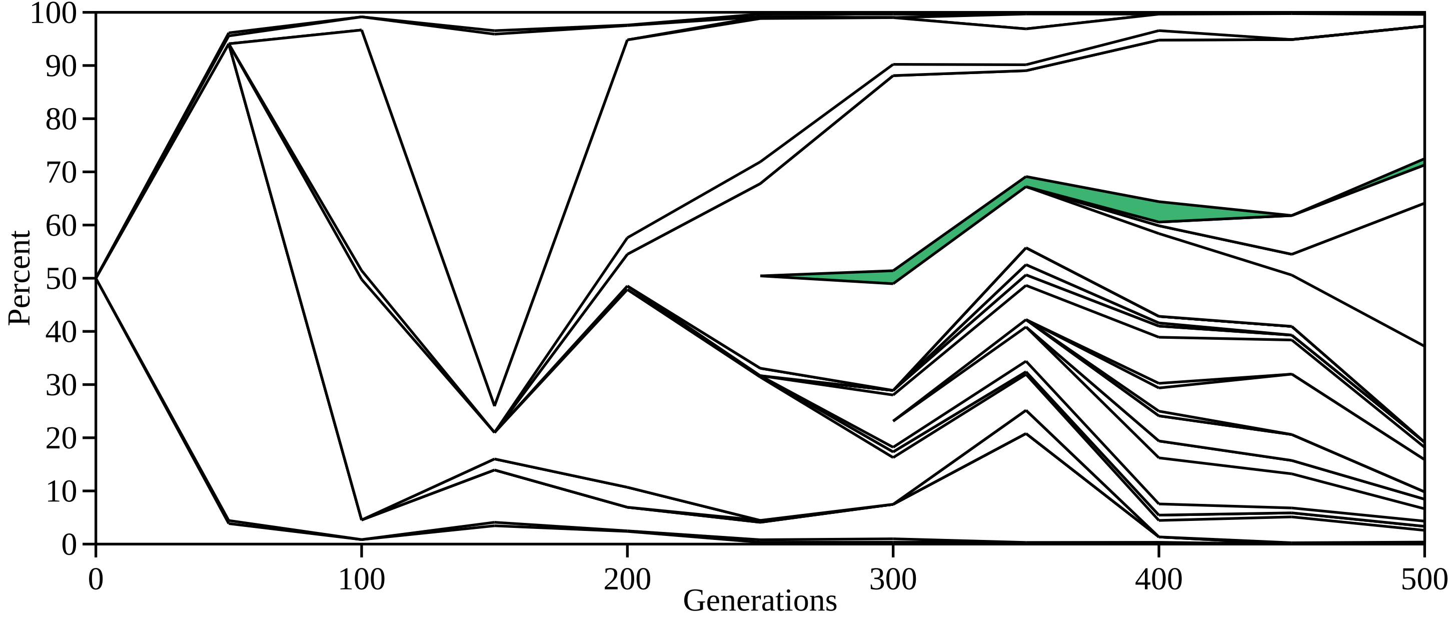

100
90
80
70
60
50
40
30
20
10
0
Percent
0
100
200
300
400
500
Generations

0.1.2.2.6.4 (hfq)

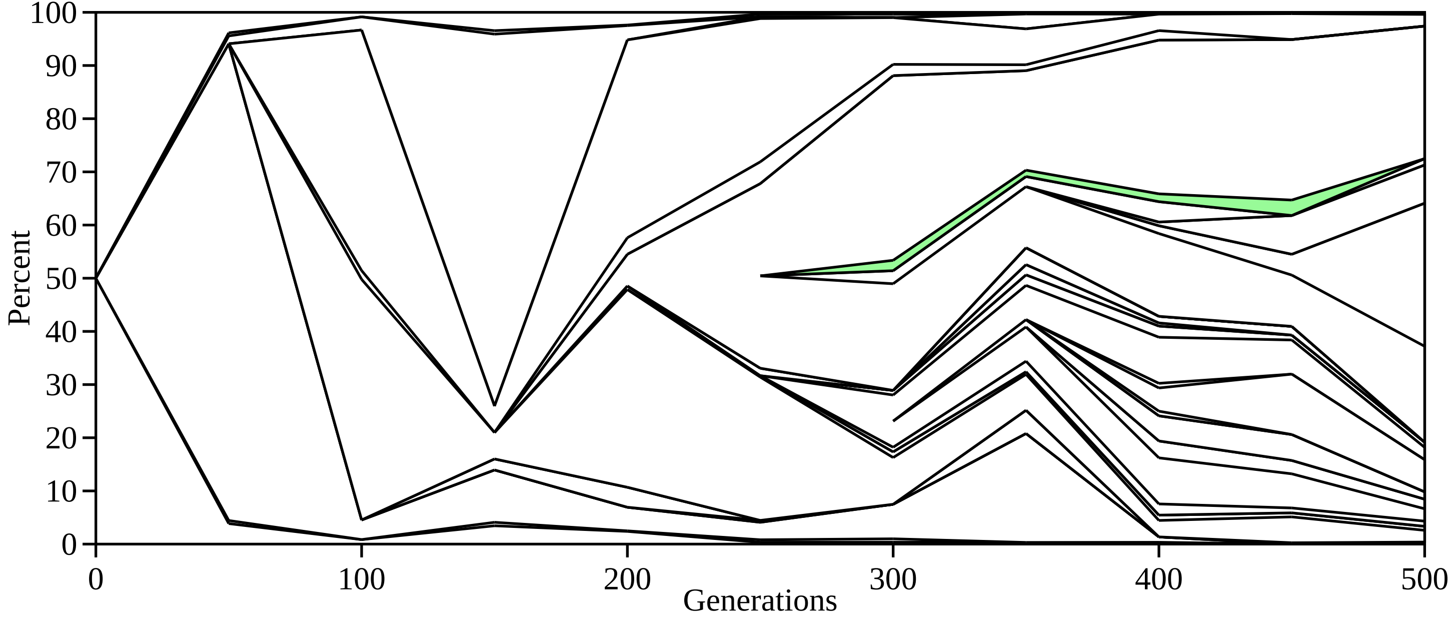
100
90
80
70
60
50
40
30
20
10
0
Percent
0
100
200
300
400
500
Generations

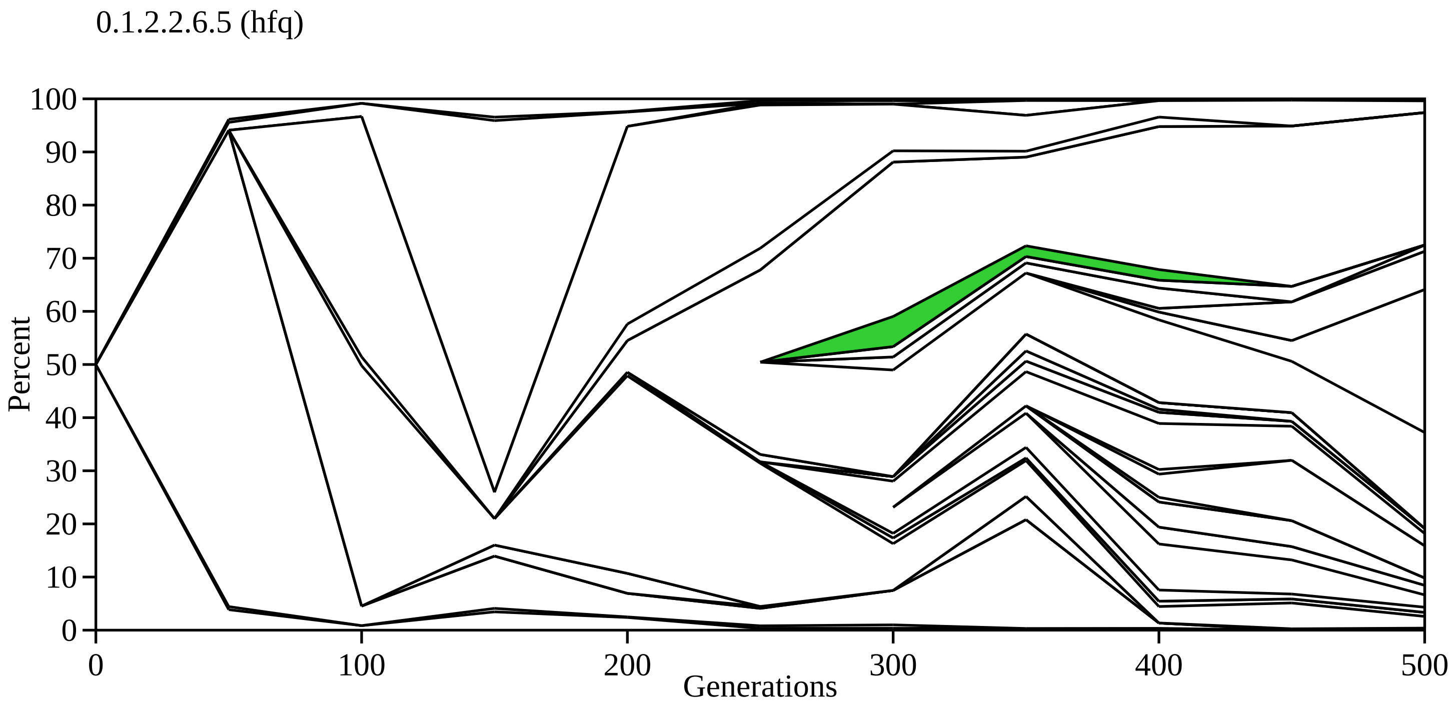
0.1.2.2.6.5 (hfq)
Percent
100
90
80
70
60
50
40
30
20
10
0
0
100
200
300
400
500
Generations

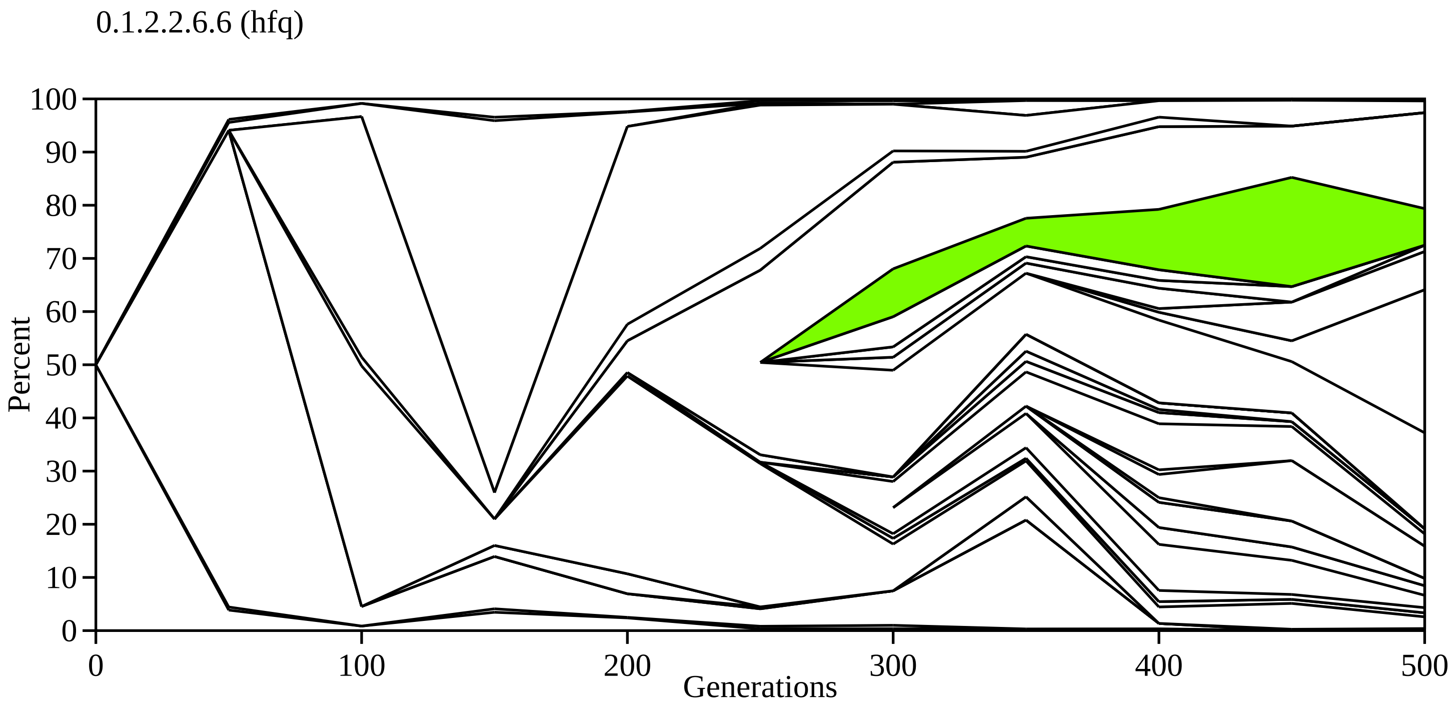
0.1.2.2.6.6 (hfq)
Percent
Generations
100
90
80
70
60
50
40
30
20
10
0
0
100
200
300
400
500

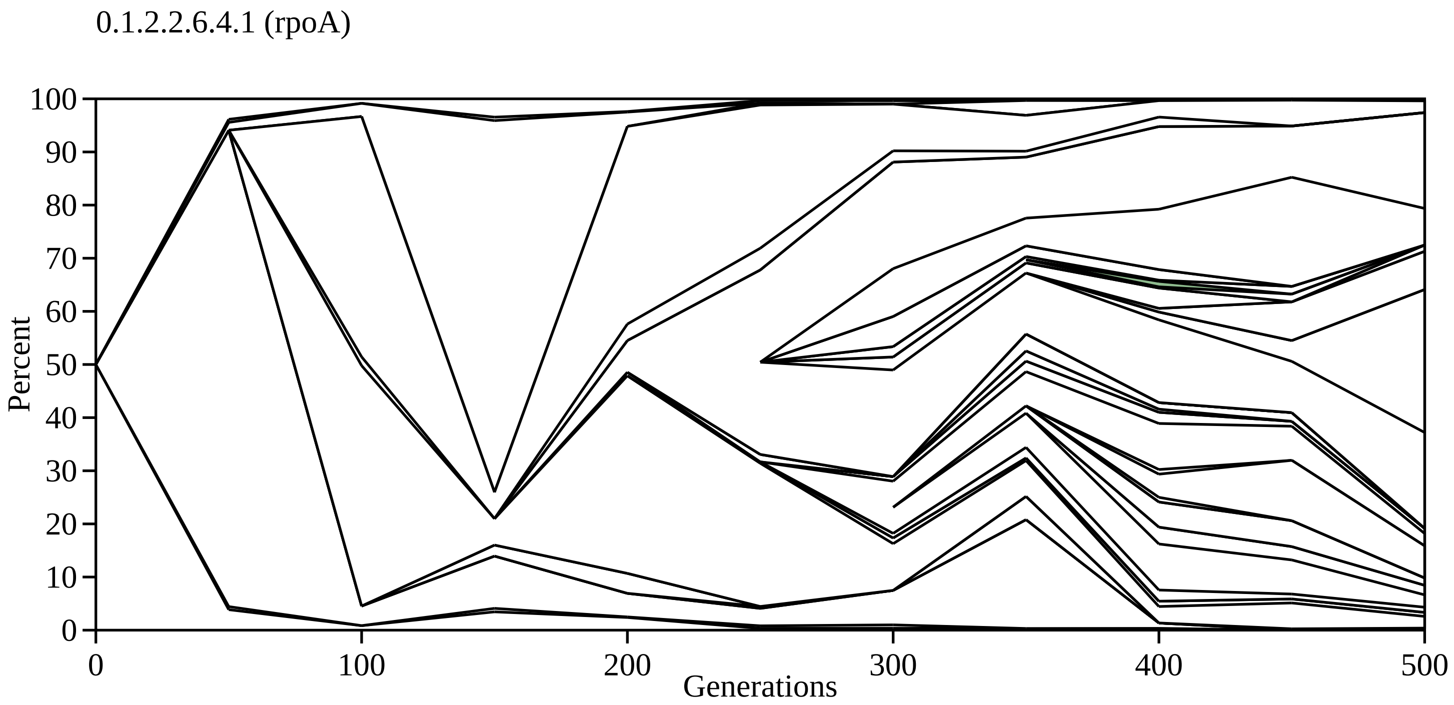

0.1.2.2.6.4.1 (rpoA)
Percent
100
90
80
70
60
50
40
30
20
10
0
0
100
200
300
400
500
Generations

0.1.2.2.6.6.1 (pfkA)

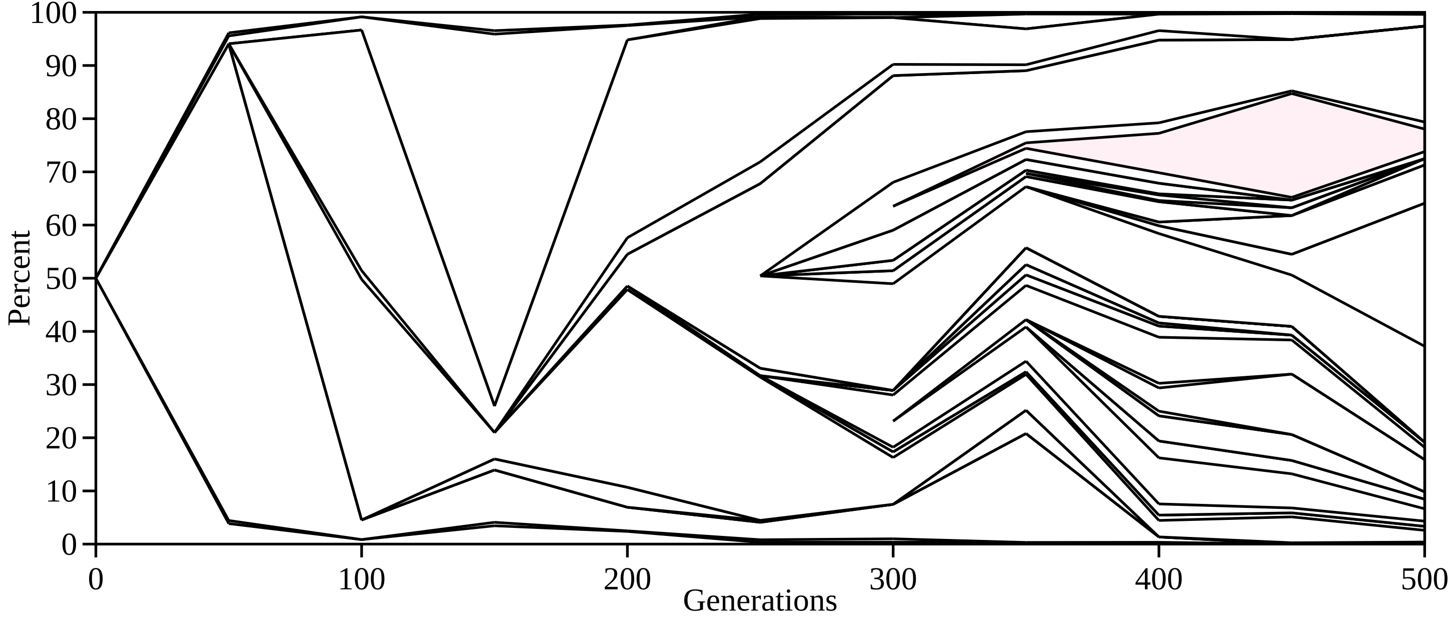

Supplement: Supplementary file 5 — Additional file 5 Fig. S9. Muller diagrams for novel alleles arising in chemostat 1, showing details for each lineage. [file 12915_2021_954_MOESM5_ESM.pdf]
