# Supplementary material for: Evolutionary dynamics and structural consequences of de novo beneficial mutations and mutant lineages arising in a constant environment
Source: BMC Biol. 2021 Feb 4;19:20. doi: 10.1186/s12915-021-00954-0 (PMC7863352; doi:10.1186/s12915-021-00954-0)
Supplement: Supplementary file 6 — Additional file 6 Fig. S10. Muller diagrams for novel alleles arising in chemostat 2, showing details for each lineage. [file 12915_2021_954_MOESM6_ESM.pdf]

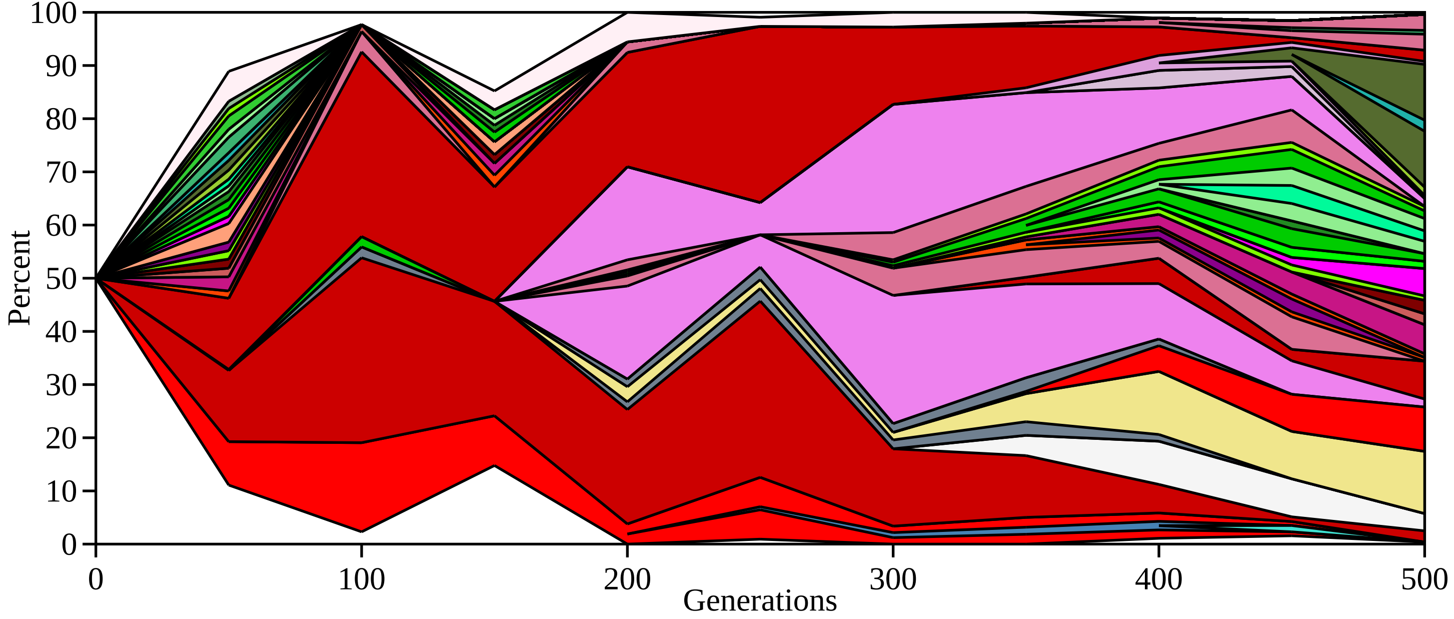

100
90
80
70
60
50
40
30
20
10
0
Percent
0
100
200
300
400
500
Generations

Lineages for deaD

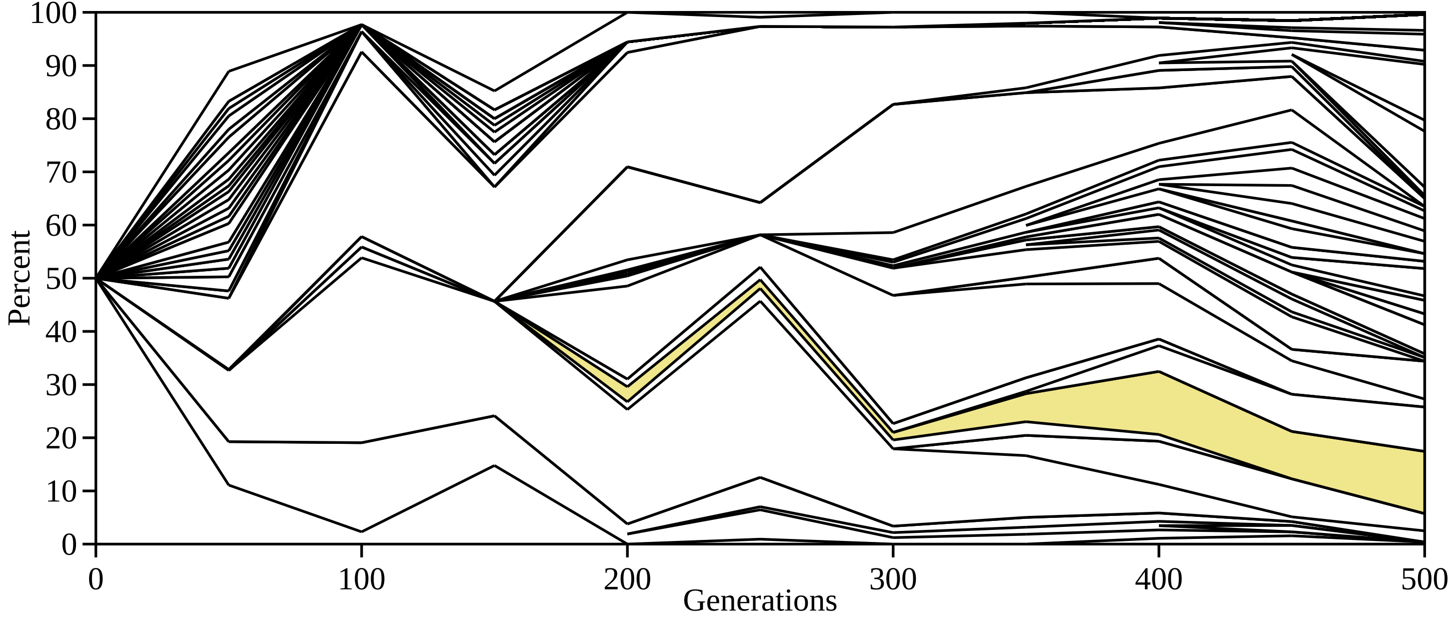

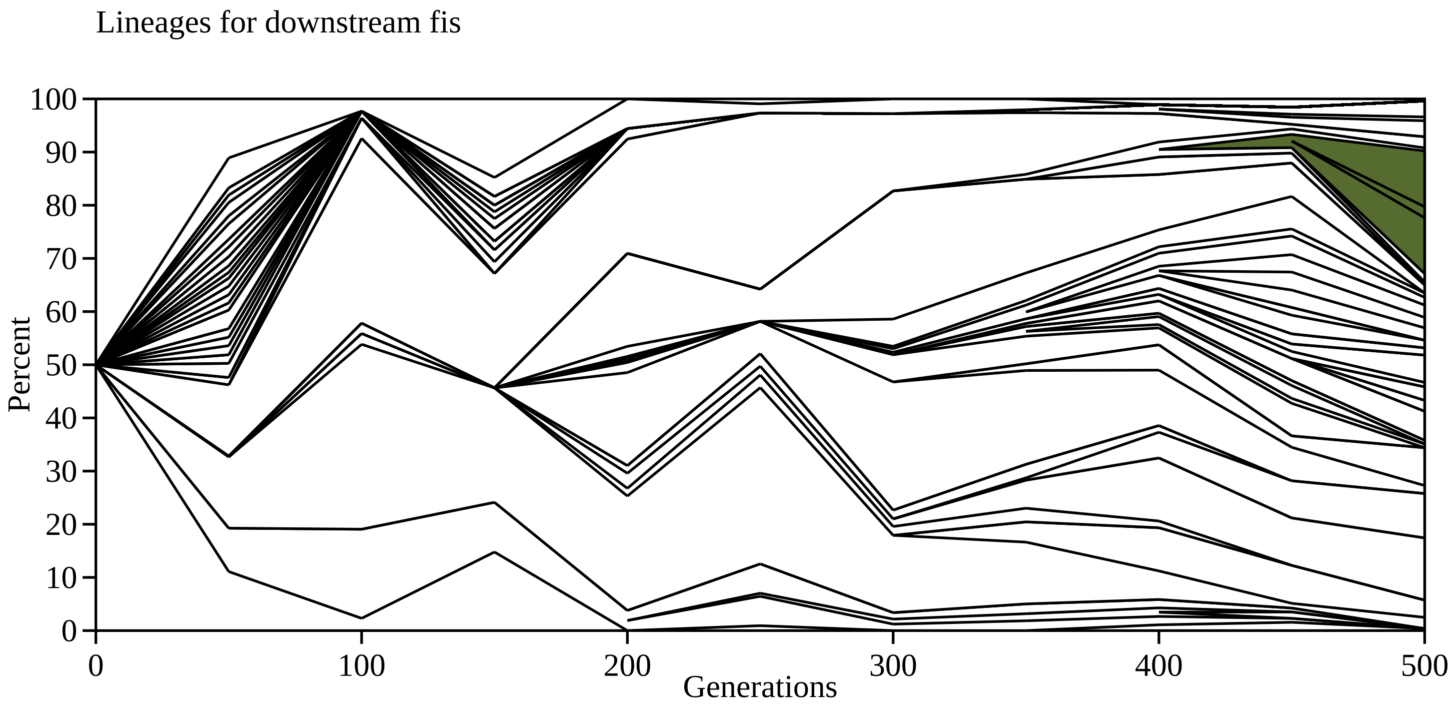
Lineages for downstream fis
100
90
80
70
60
50
40
30
20
10
0
Percent
0
100
200
300
400
500
Generations

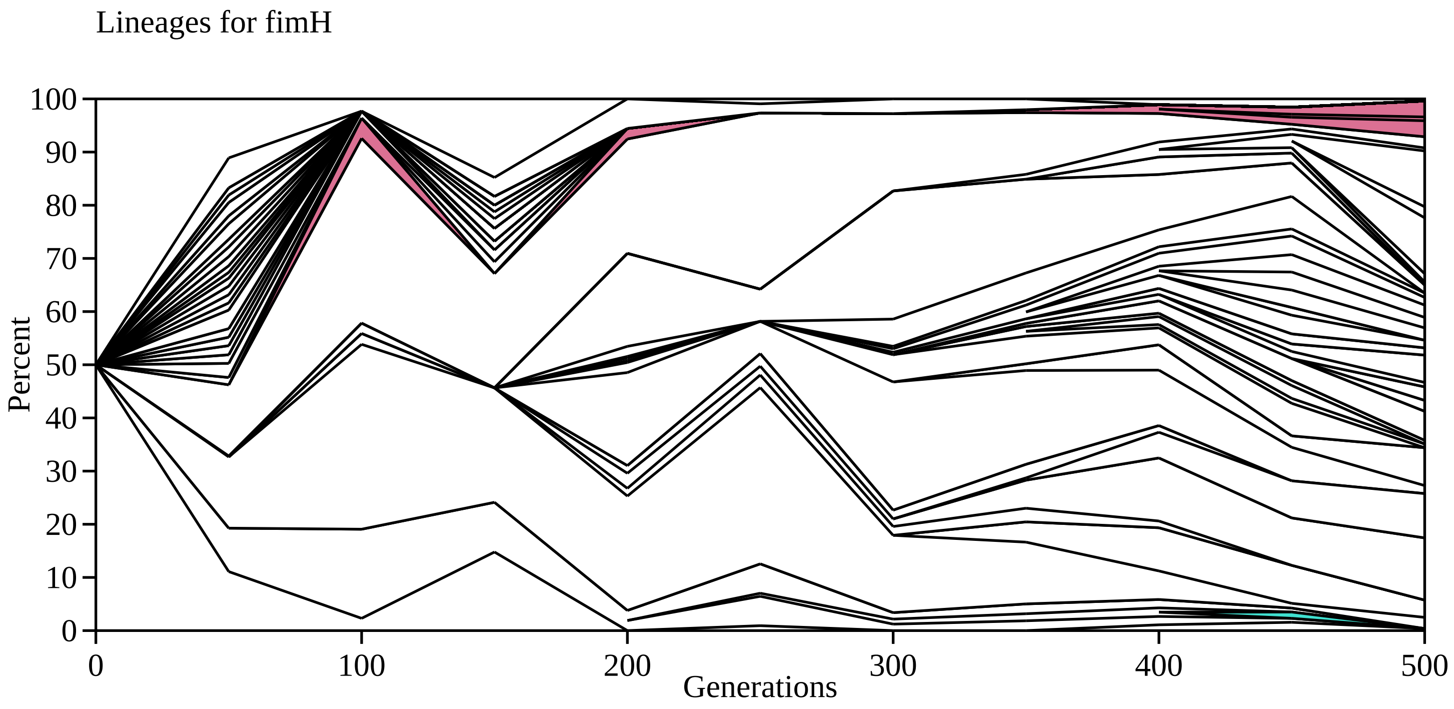

Lineages for fimH
Percent
100
90
80
70
60
50
40
30
20
10
0
0
100
200
300
400
500
Generations

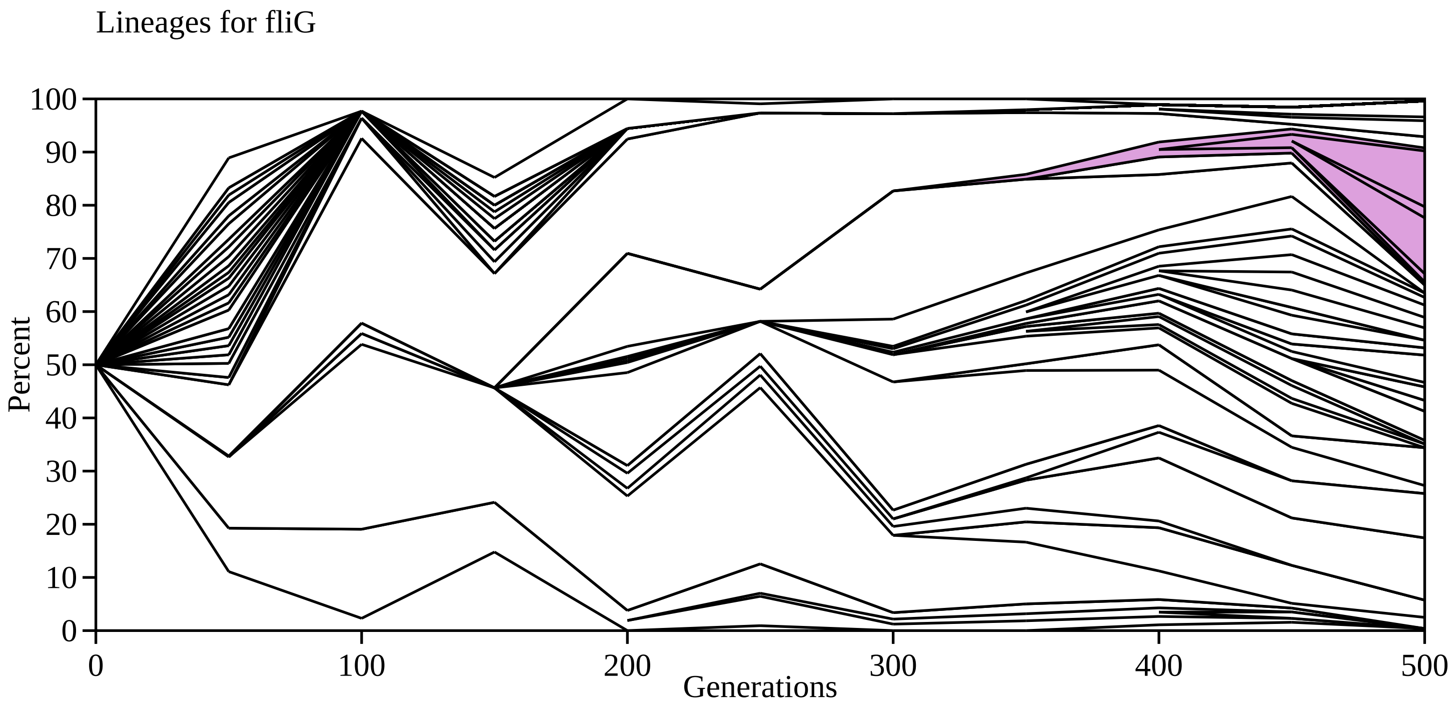

Lineages for fliG
Percent
0
10
20
30
40
50
60
70
80
90
100
0
100
200
300
400
500
Generations

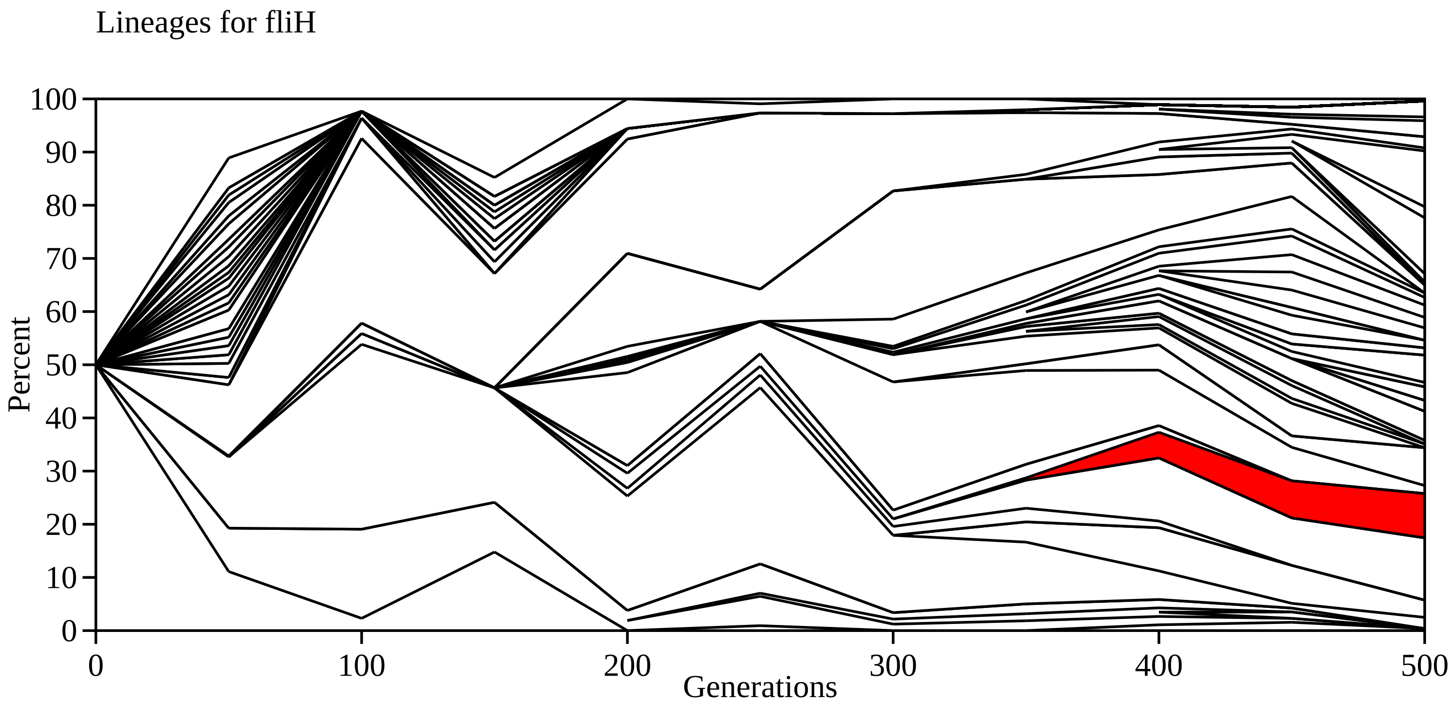

Lineages for fliH
Percent
100
90
80
70
60
50
40
30
20
10
0
0
100
200
300
400
500
Generations

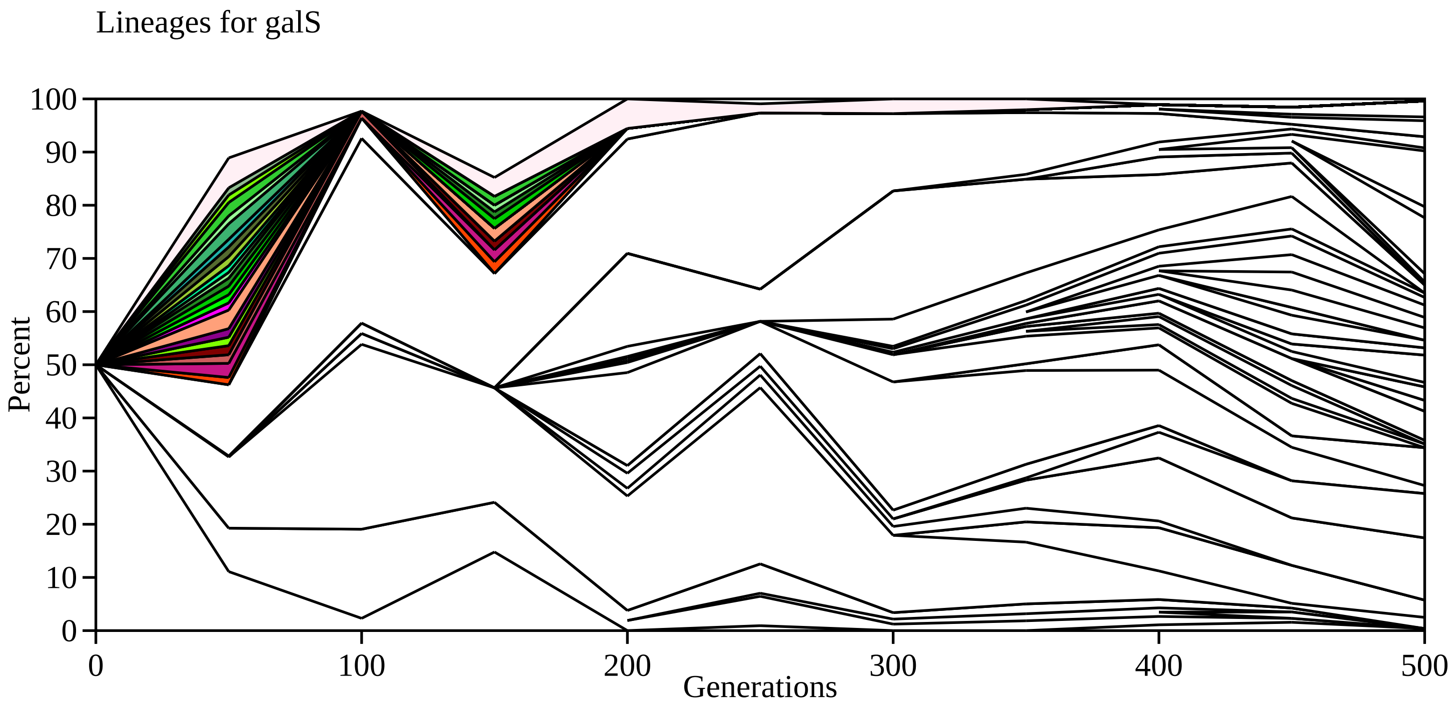

Lineages for galS
Percent
Generations
100
90
80
70
60
50
40
30
20
10
0
0
100
200
300
400
500

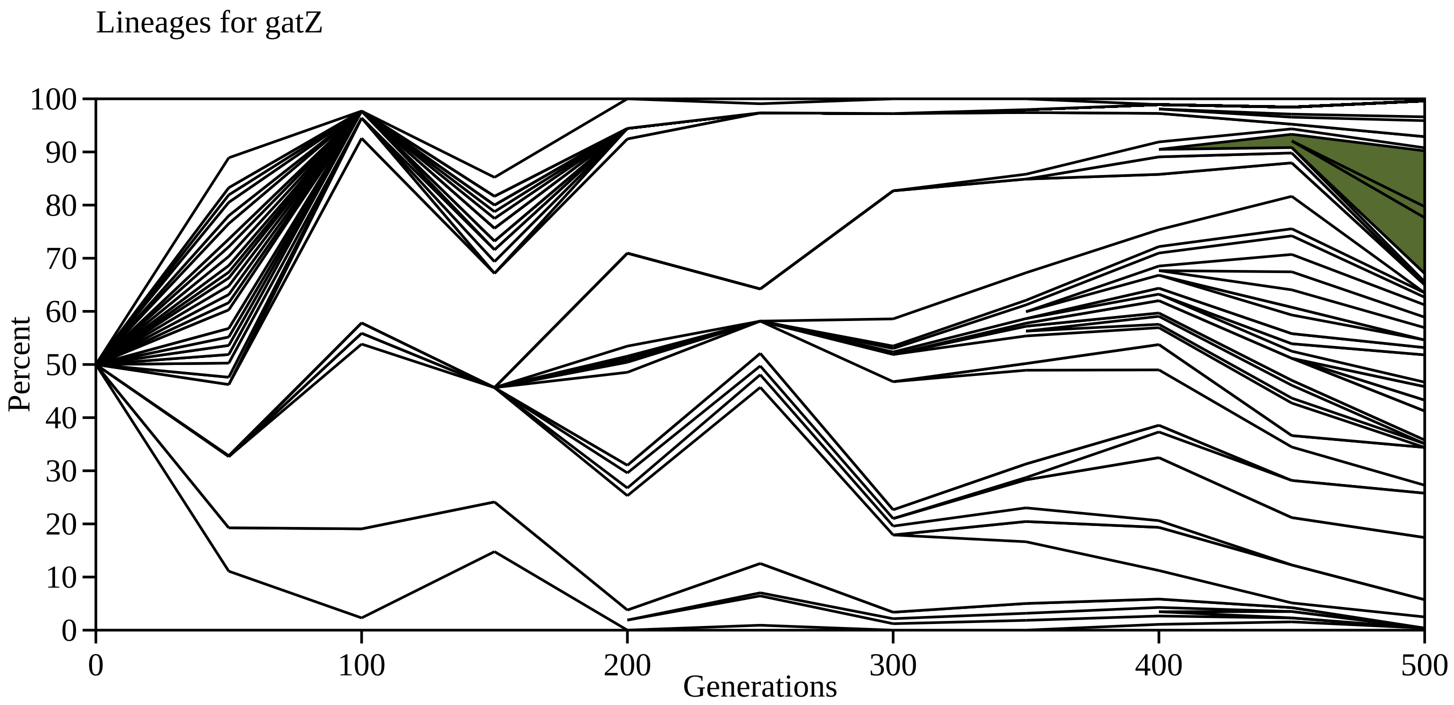
Lineages for gatZ
100
90
80
70
60
50
40
30
20
10
0
Percent
0
100
200
300
400
500
Generations

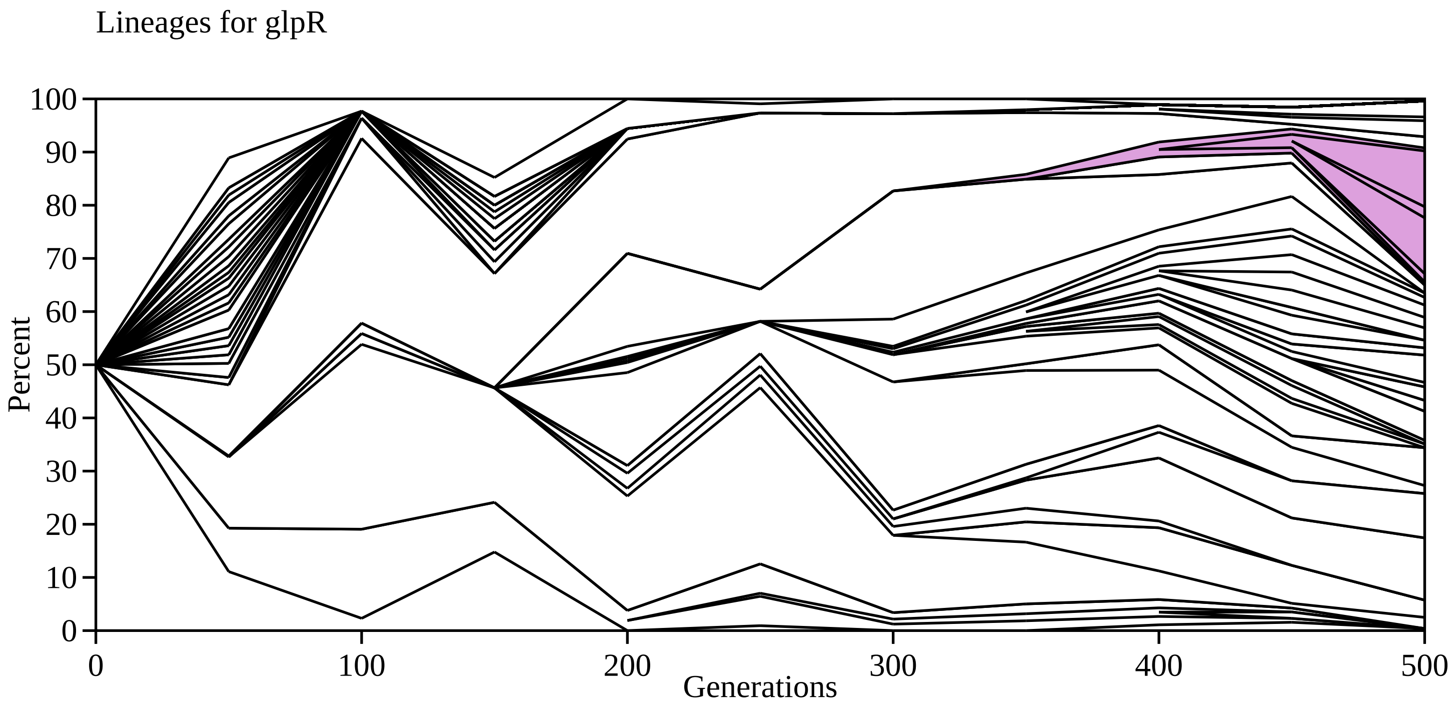

Lineages for glpR
Percent
100
90
80
70
60
50
40
30
20
10
0
0
100
200
300
400
500
Generations

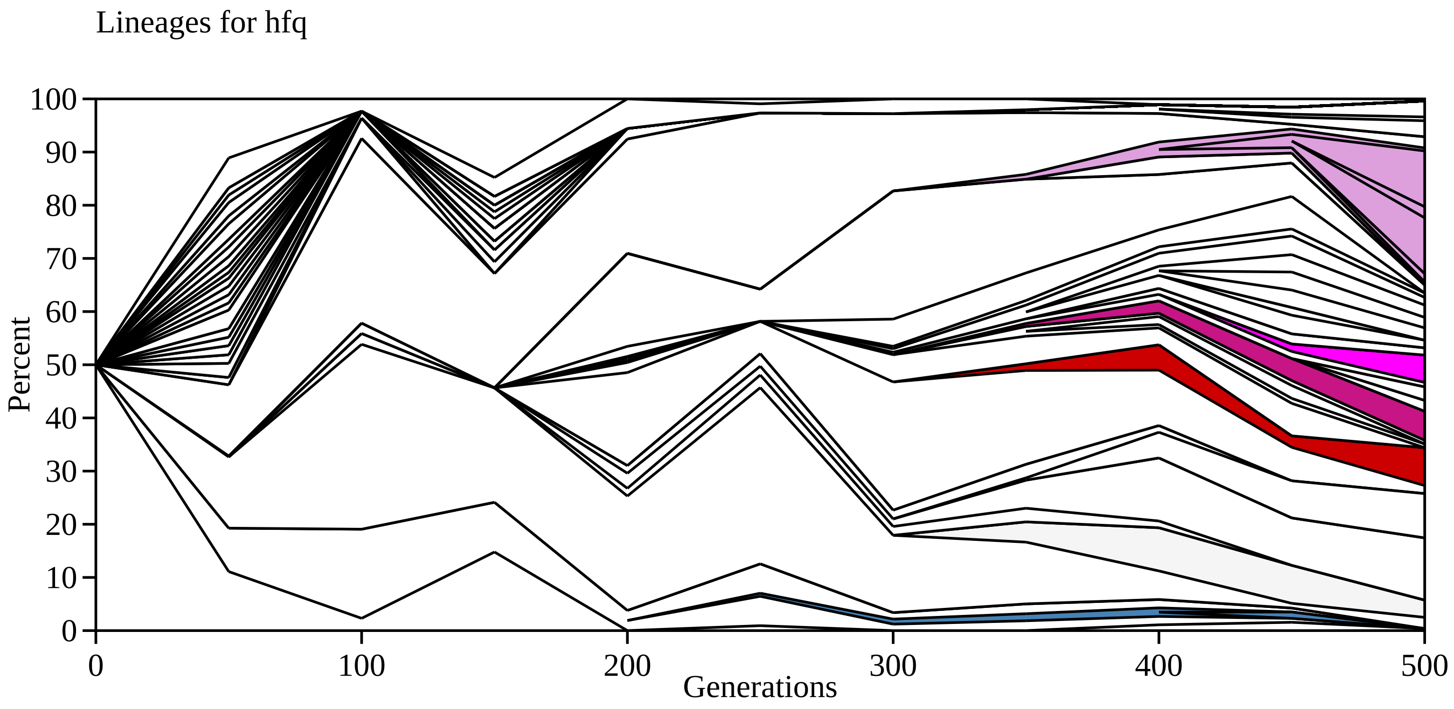
Lineages for hfq
Percent
Generations
100
90
80
70
60
50
40
30
20
10
0
0
100
200
300
400
500

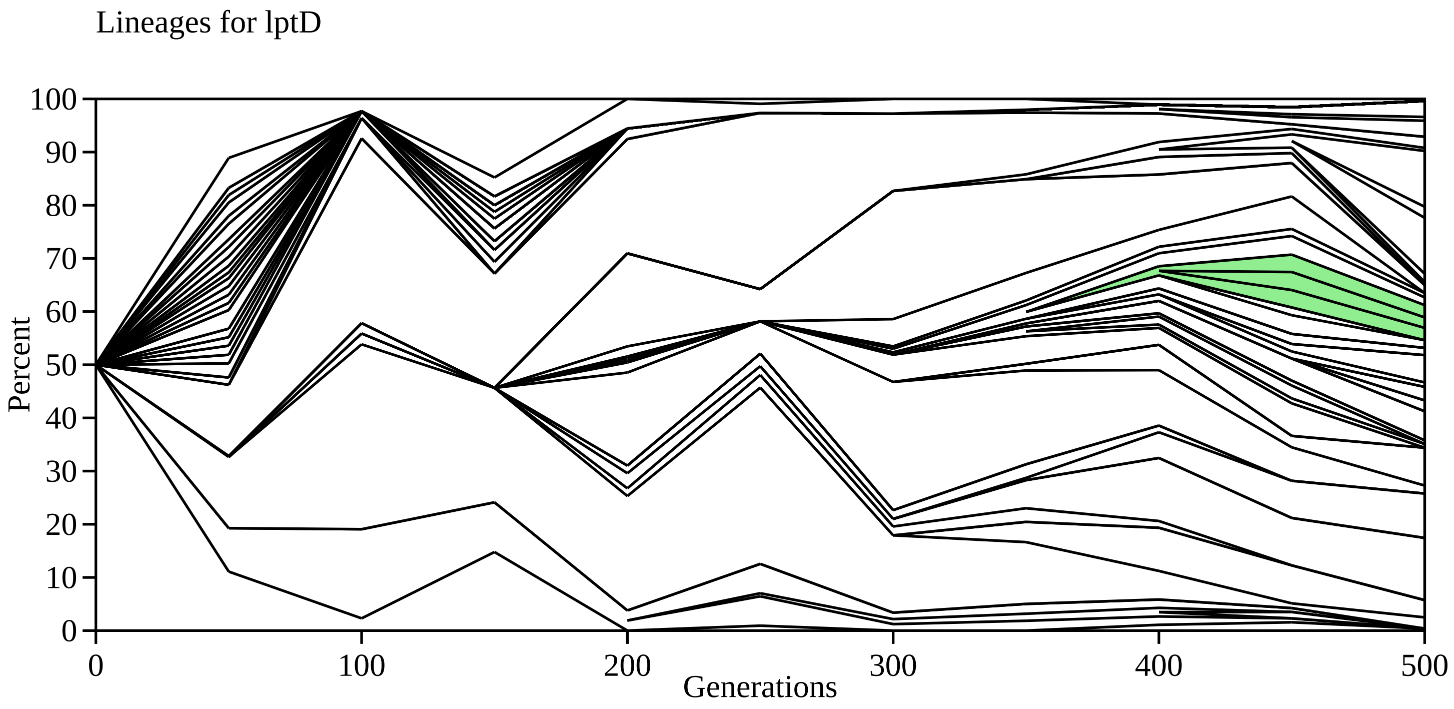

Lineages for lptD
Percent
100
90
80
70
60
50
40
30
20
10
0
0
100
200
300
400
500
Generations

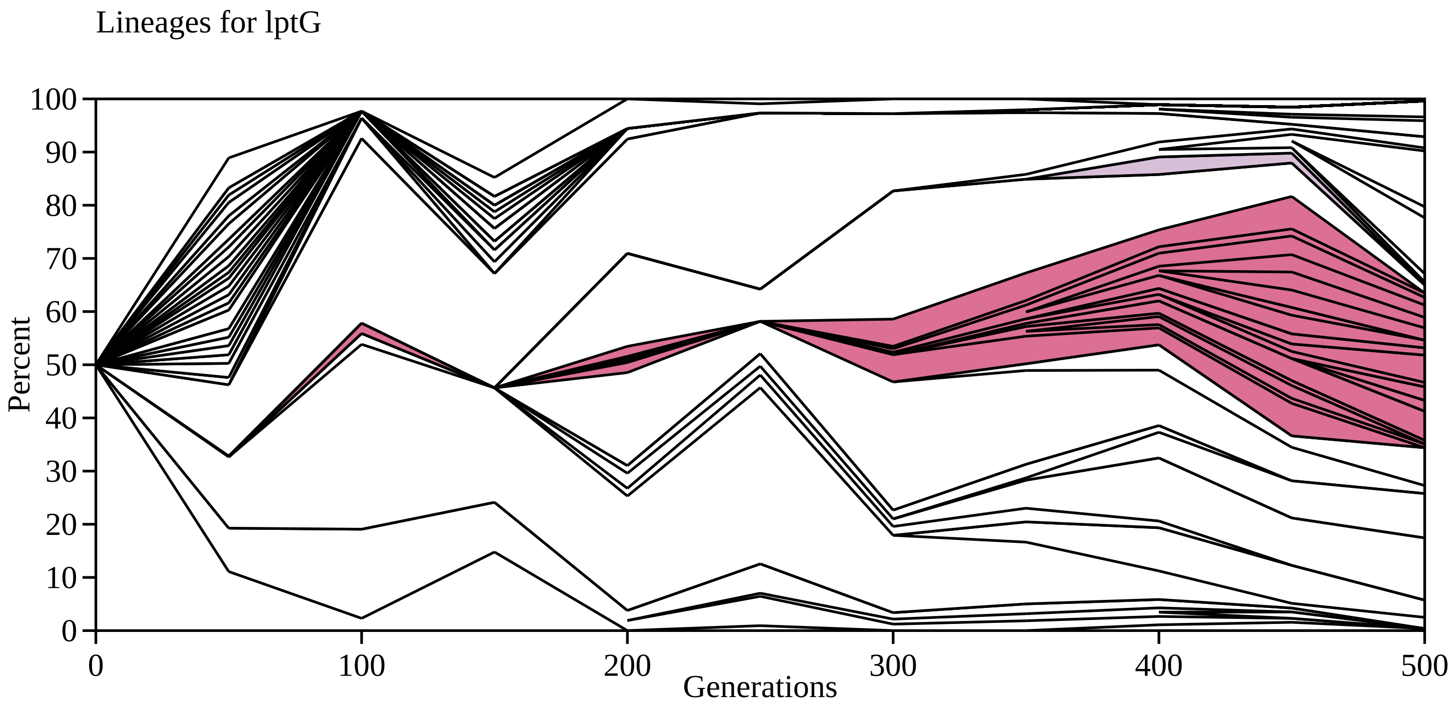

Lineages for lptG
100
90
80
70
60
50
40
30
20
10
0
Percent
0
100
200
300
400
500
Generations

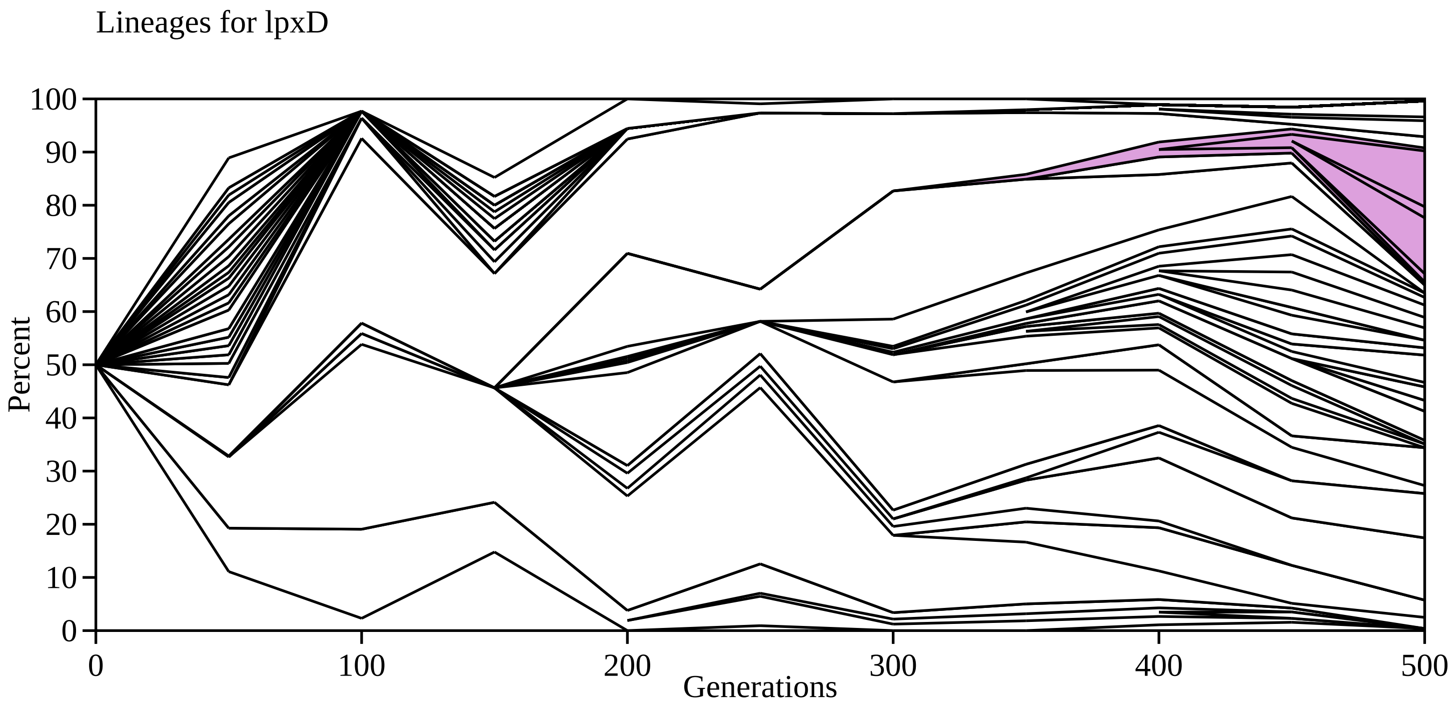

Lineages for lpxD
100
90
80
70
60
50
40
30
20
10
0
Percent
0
100
200
300
400
500
Generations

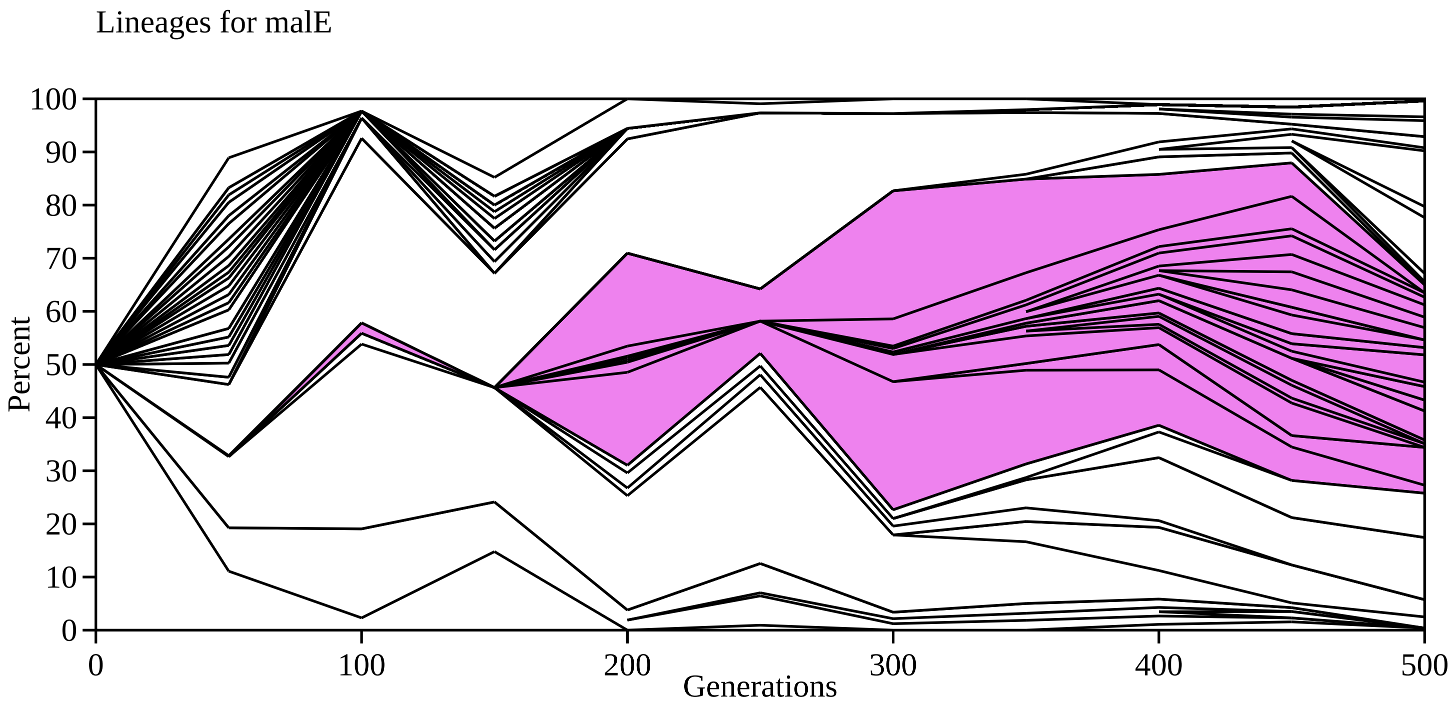

Lineages for malE
100
90
80
70
60
50
40
30
20
10
0
Percent
0
100
200
300
400
500
Generations

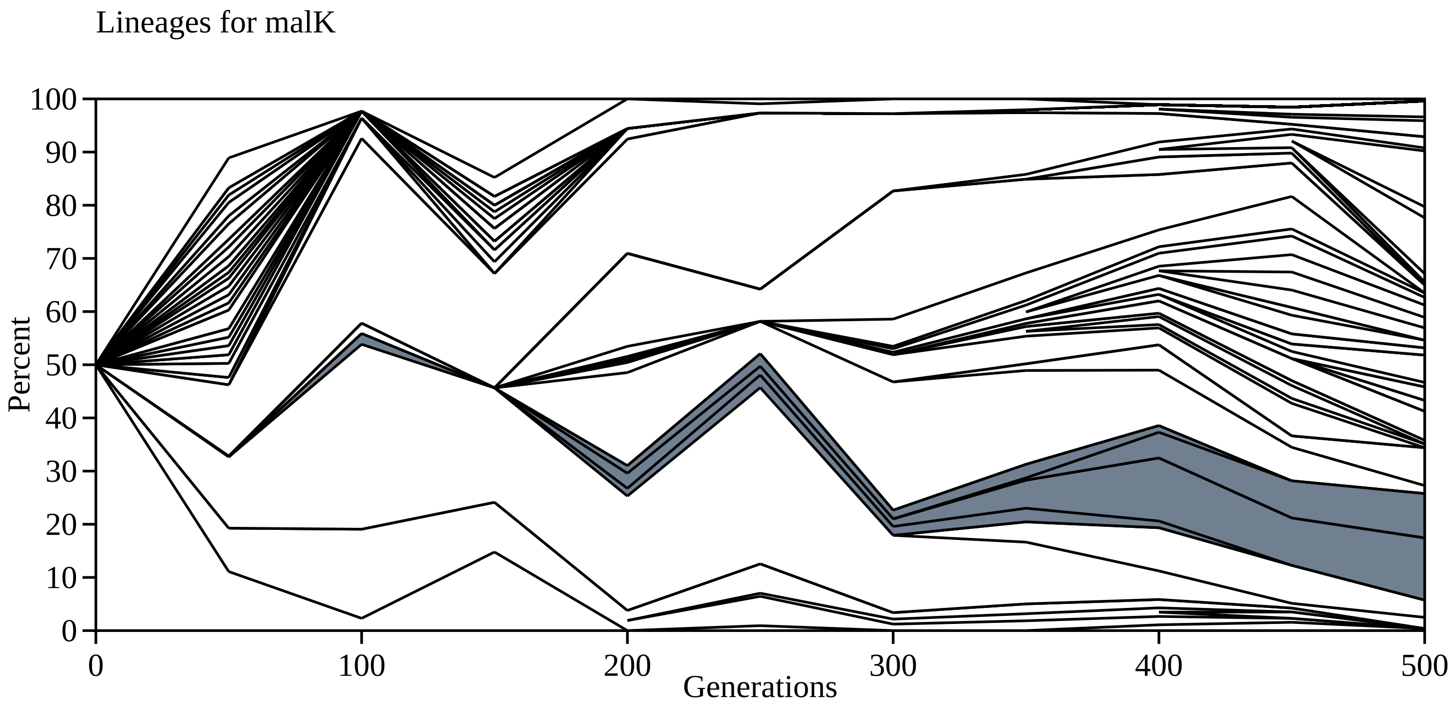

Lineages for malK
Percent
Generations
100
90
80
70
60
50
40
30
20
10
0
0
100
200
300
400
500

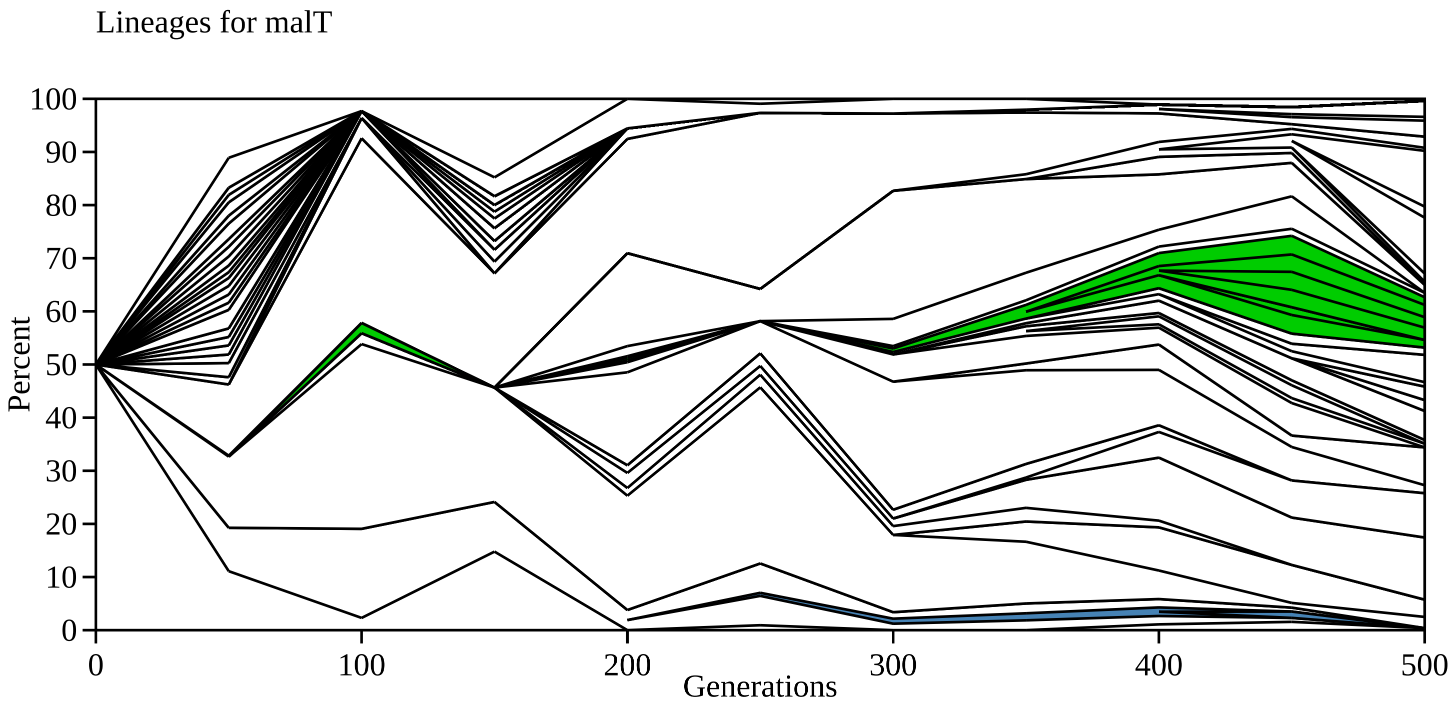

Lineages for malT
Percent
Generations
0
10
20
30
40
50
60
70
80
90
100
0
100
200
300
400
500

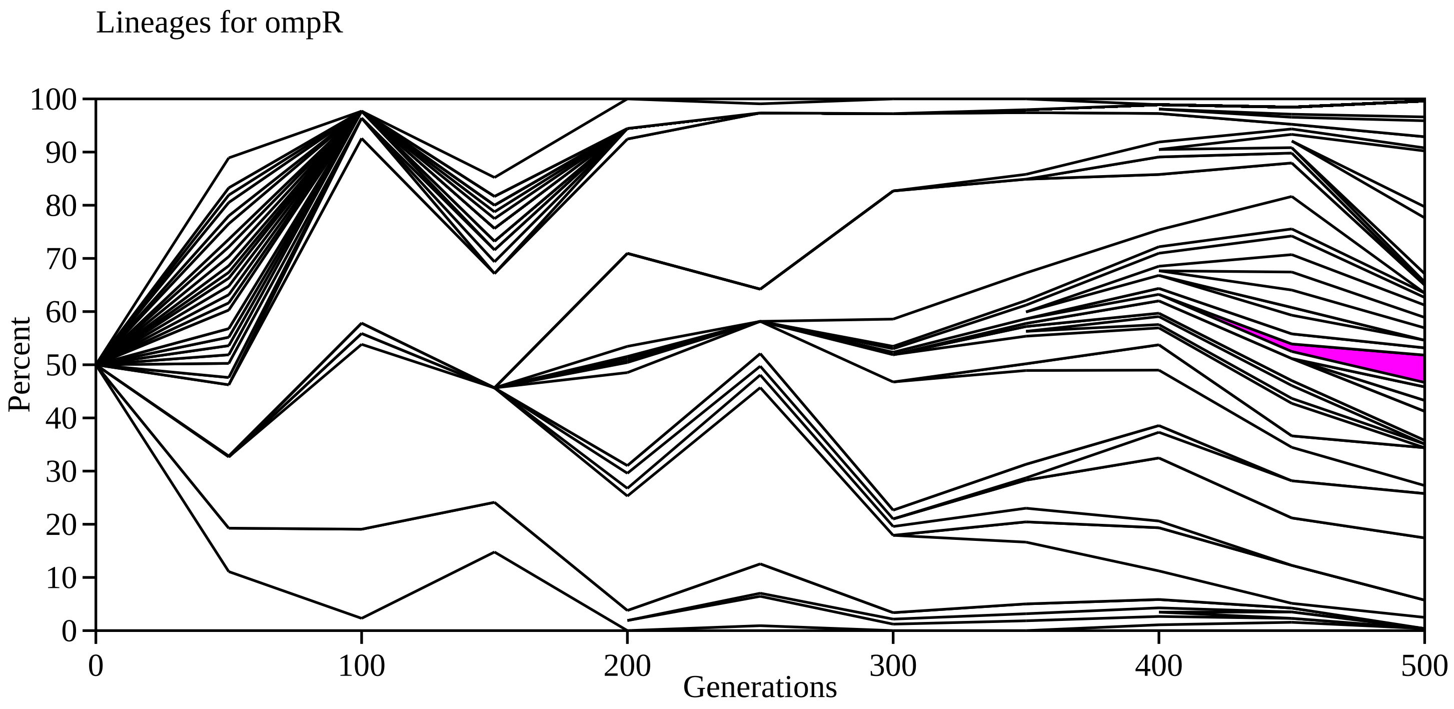

Lineages for ompR
100
90
80
70
60
50
40
30
20
10
0
Percent
0
100
200
300
400
500
Generations

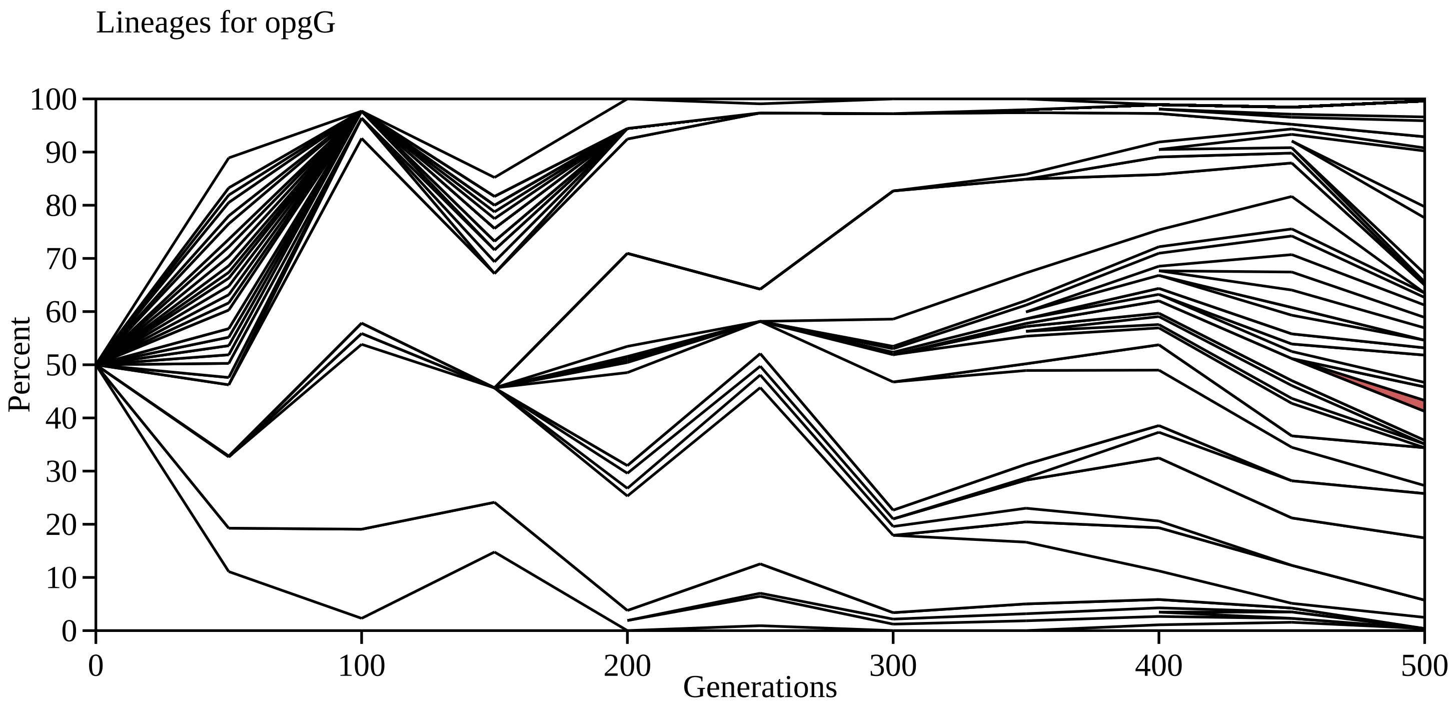
Lineages for opgG
Percent
Generations
100
90
80
70
60
50
40
30
20
10
0
0
100
200
300
400
500

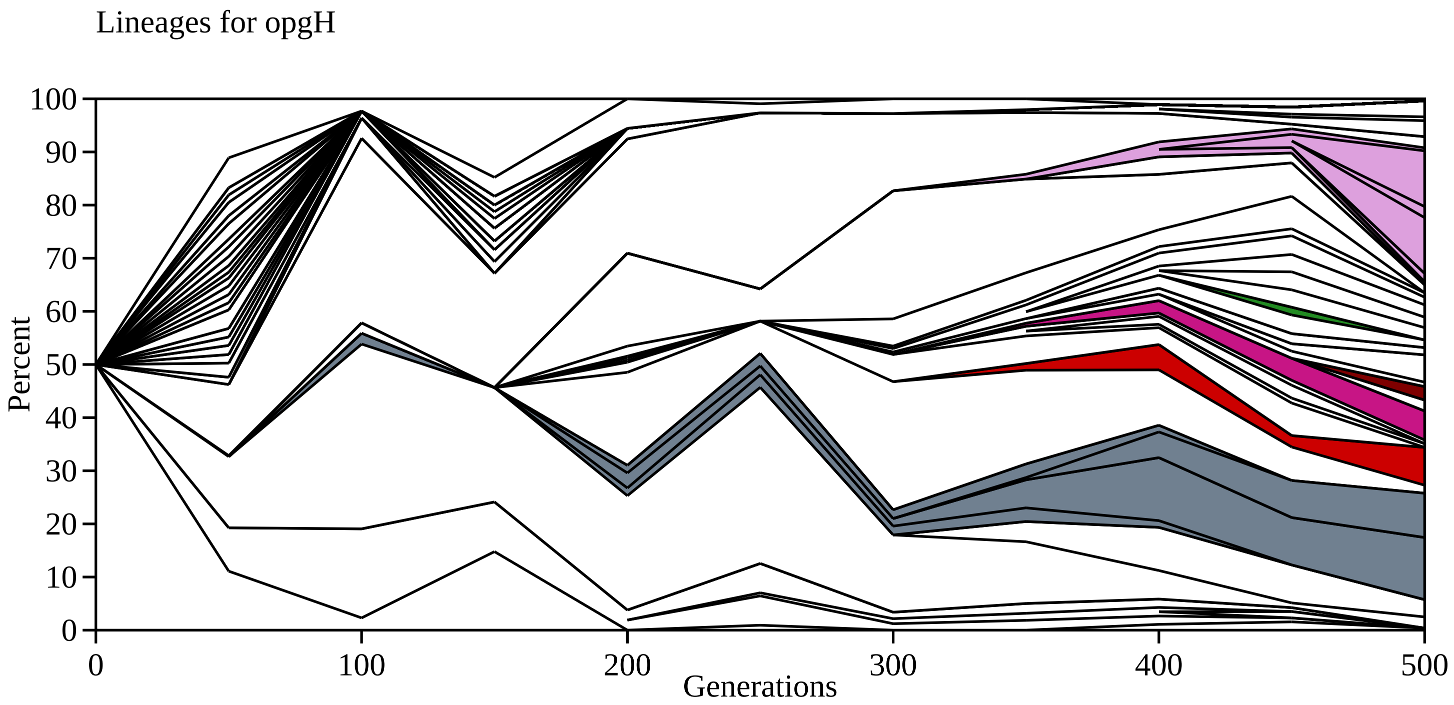

Lineages for opgH
Percent
100
90
80
70
60
50
40
30
20
10
0
0
100
200
300
400
500
Generations

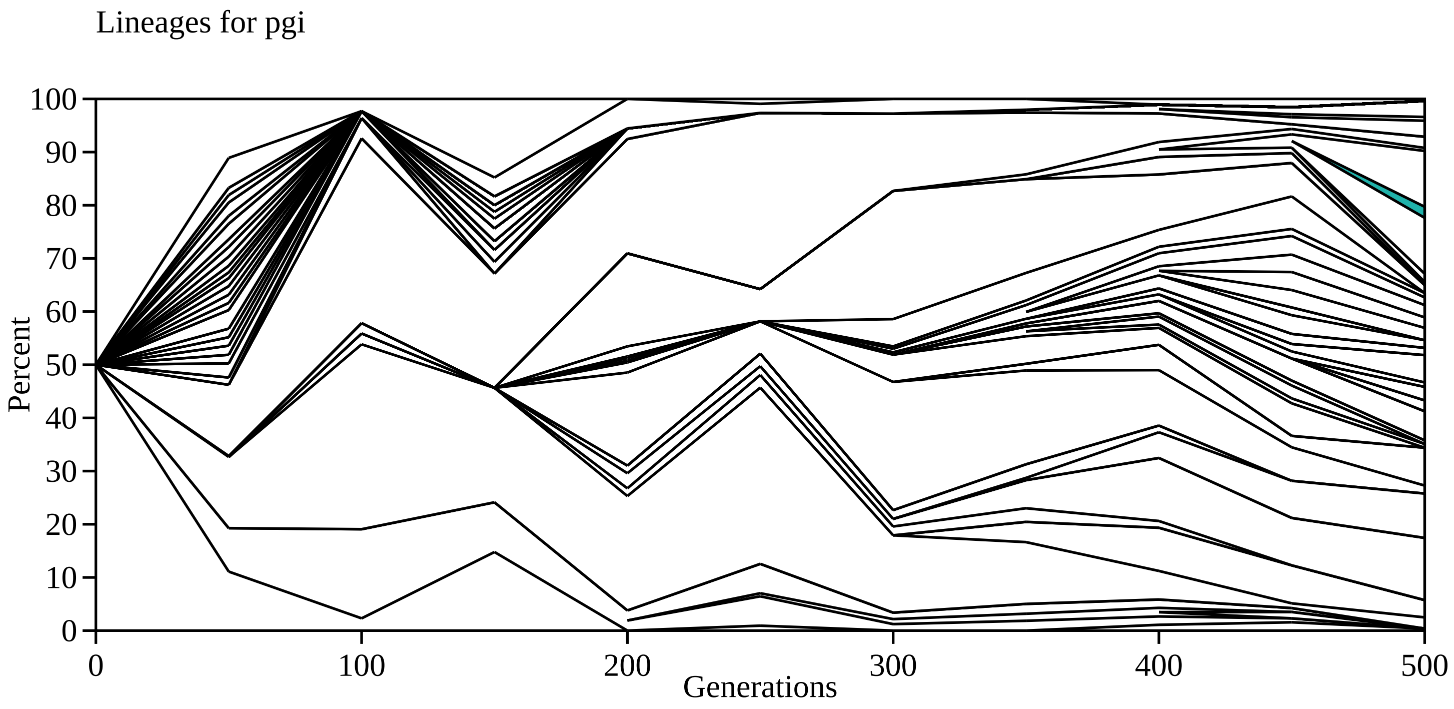

Lineages for pgi
100
90
80
70
60
50
40
30
20
10
0
Percent
0
100
200
300
400
500
Generations

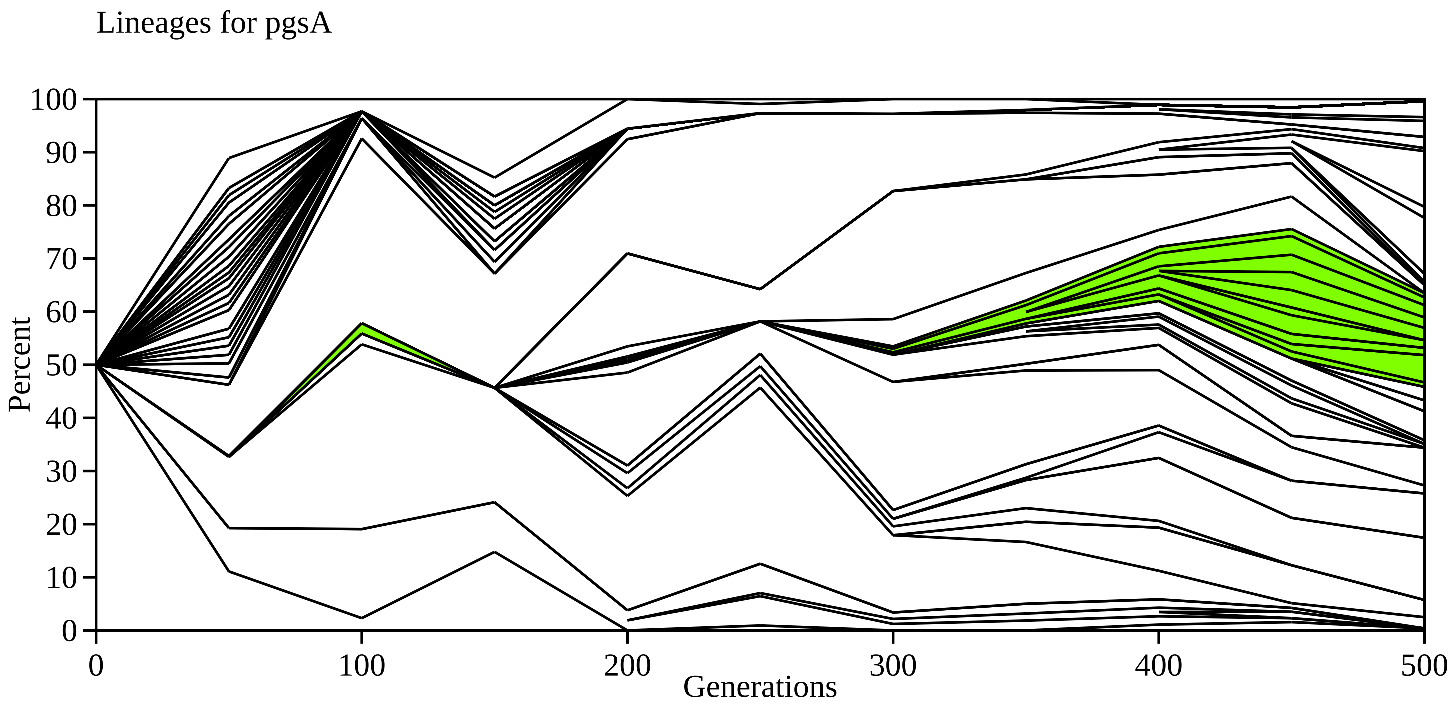

Lineages for pgsA
Percent
100
90
80
70
60
50
40
30
20
10
0
0
100
200
300
400
500
Generations

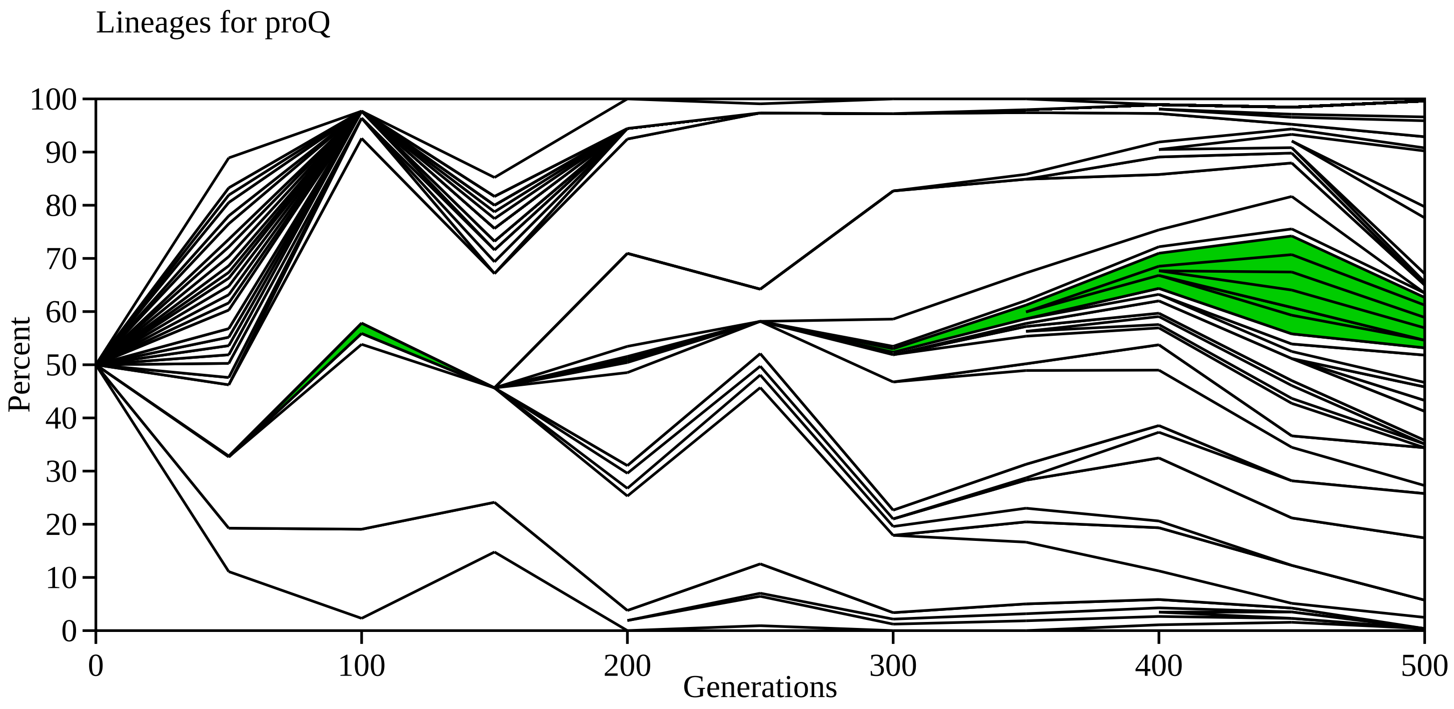

Lineages for proQ
Percent
Generations
100
90
80
70
60
50
40
30
20
10
0
0
100
200
300
400
500

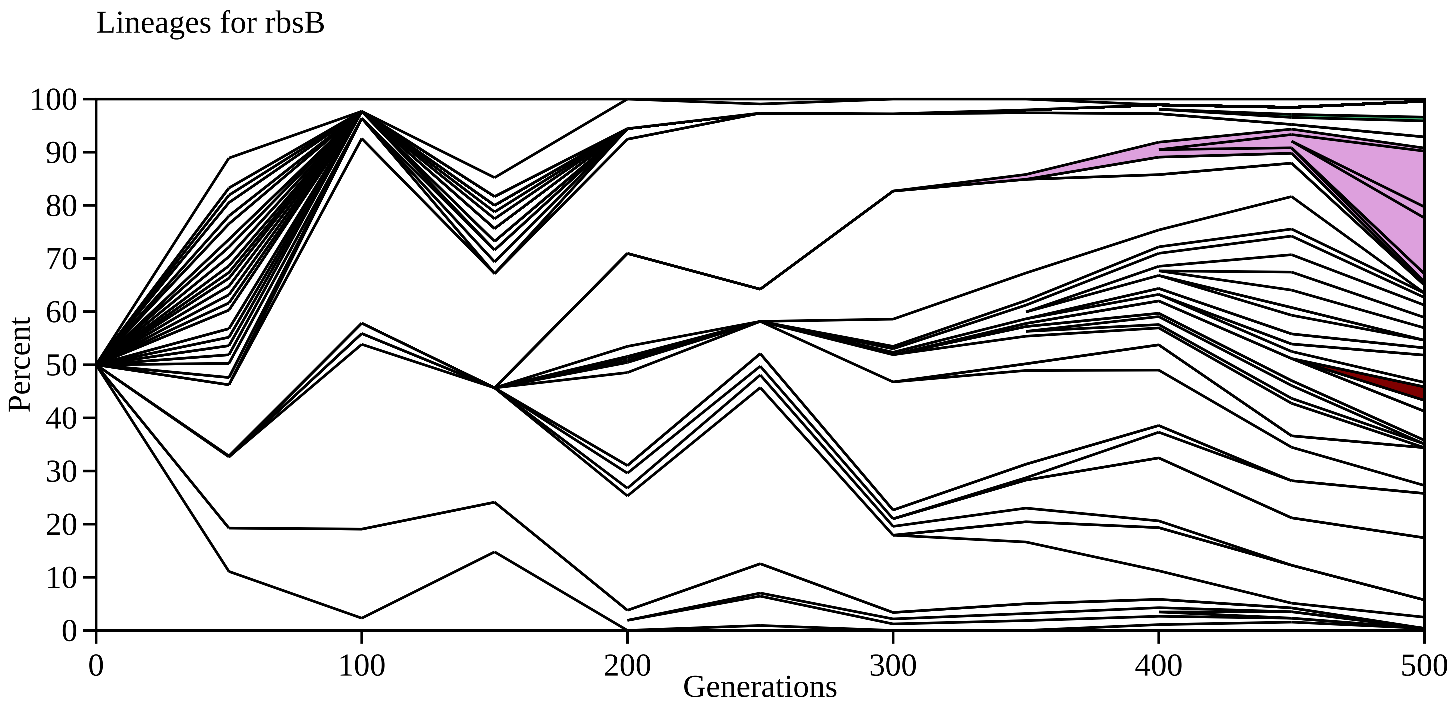

Lineages for rbsB
100
90
80
70
60
50
40
30
20
10
0
Percent
0
100
200
300
400
500
Generations

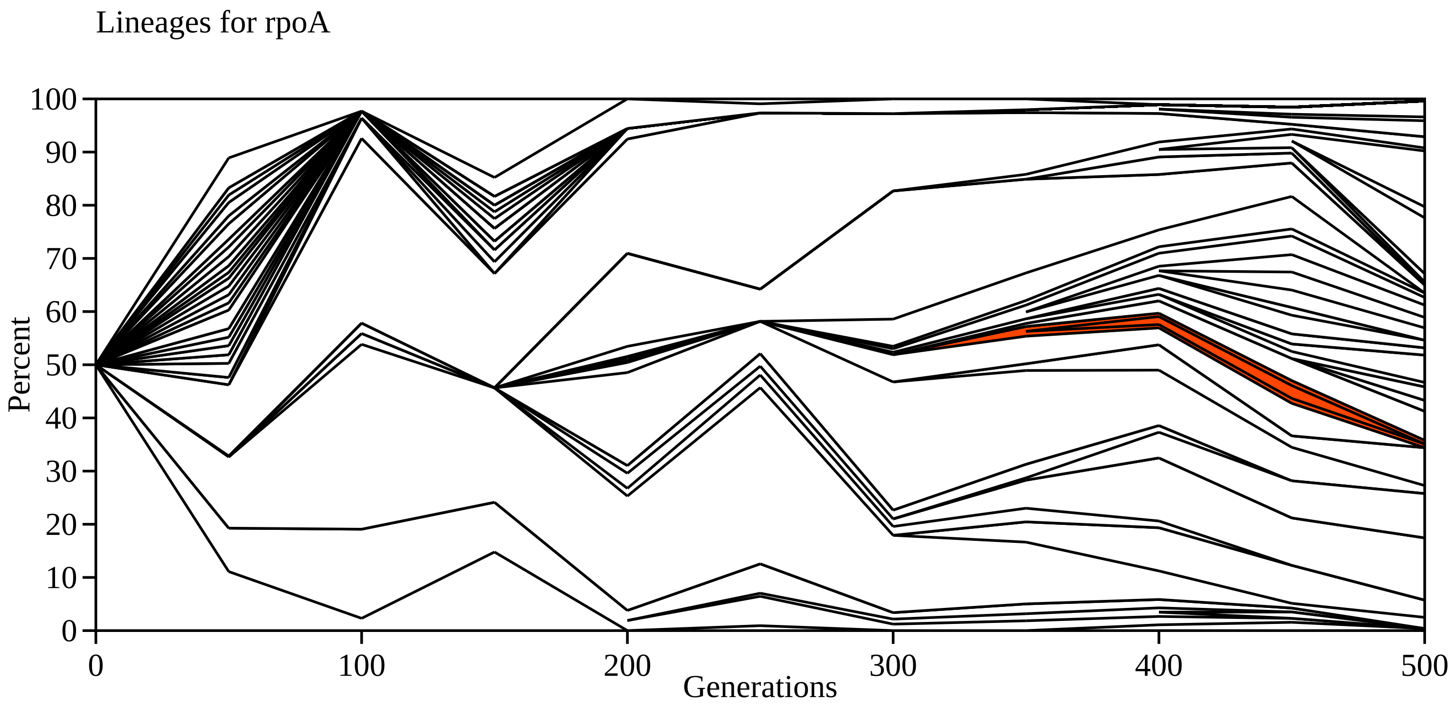
Lineages for rpoA
Percent
Generations
0
10
20
30
40
50
60
70
80
90
100
0
100
200
300
400
500

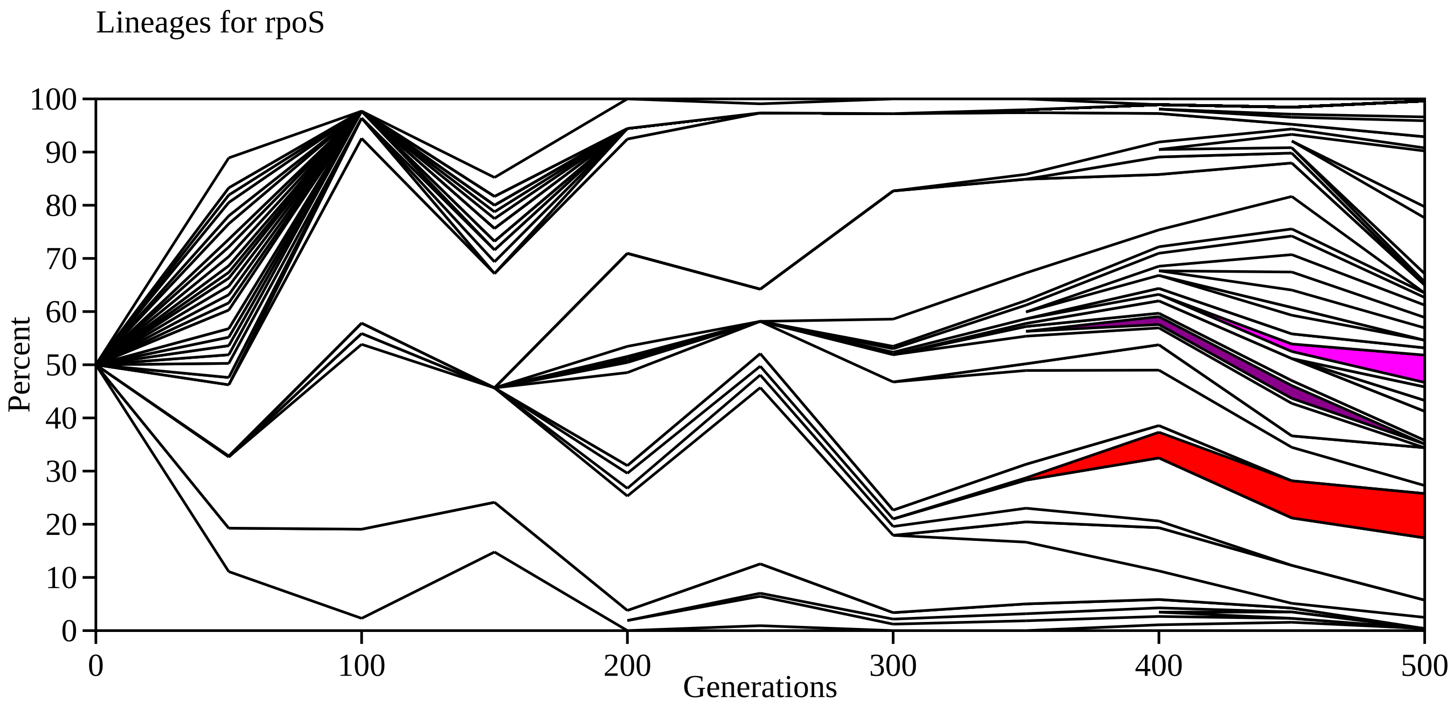

Lineages for rpoS
Percent
100
90
80
70
60
50
40
30
20
10
0
0
100
200
300
400
500
Generations

Lineages for slt

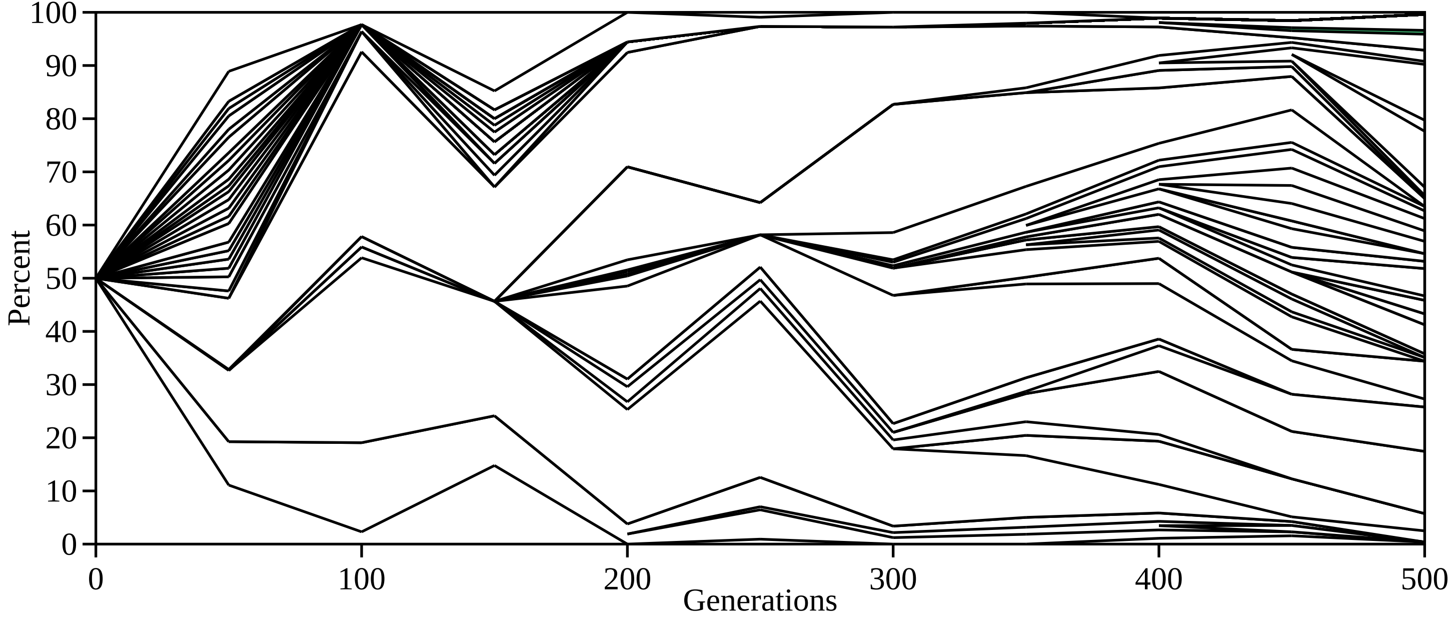

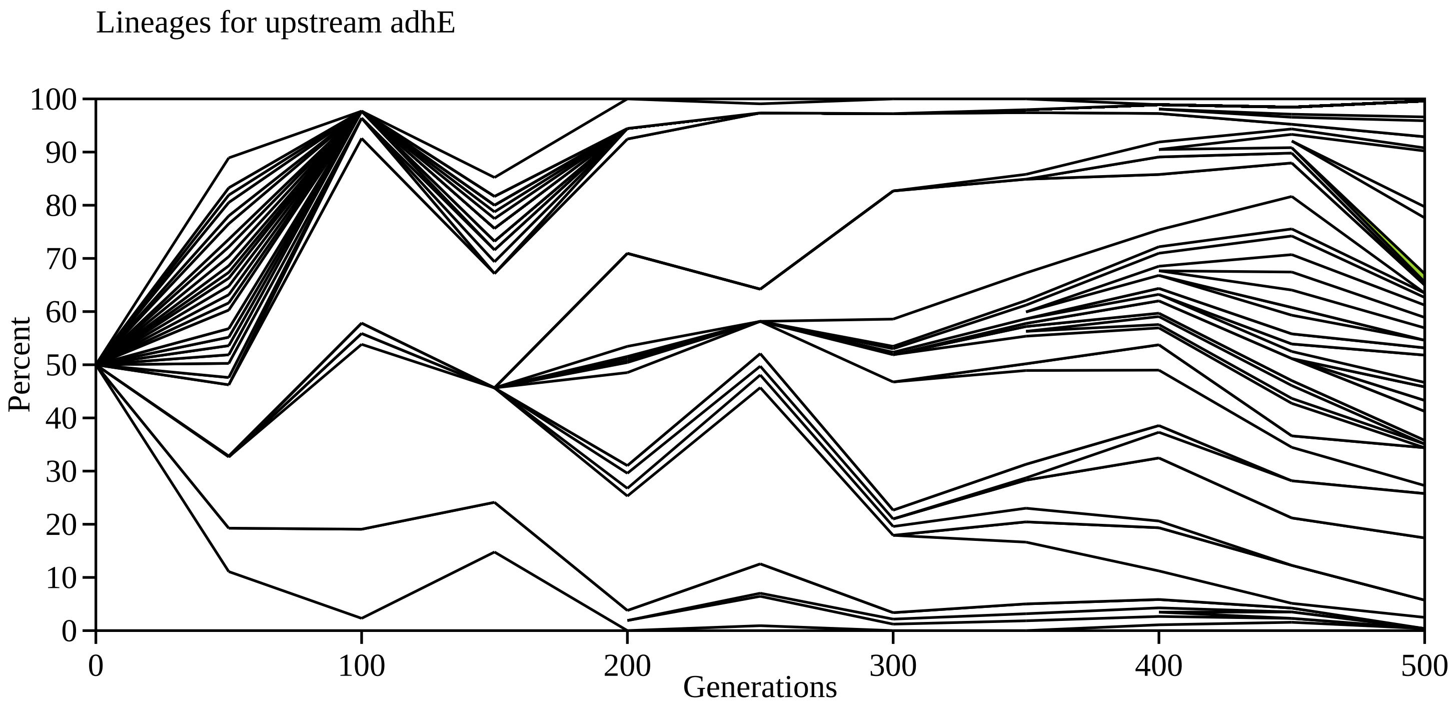

Lineages for upstream adhE
100
90
80
70
60
50
40
30
20
10
0
Percent
0
100
200
300
400
500
Generations

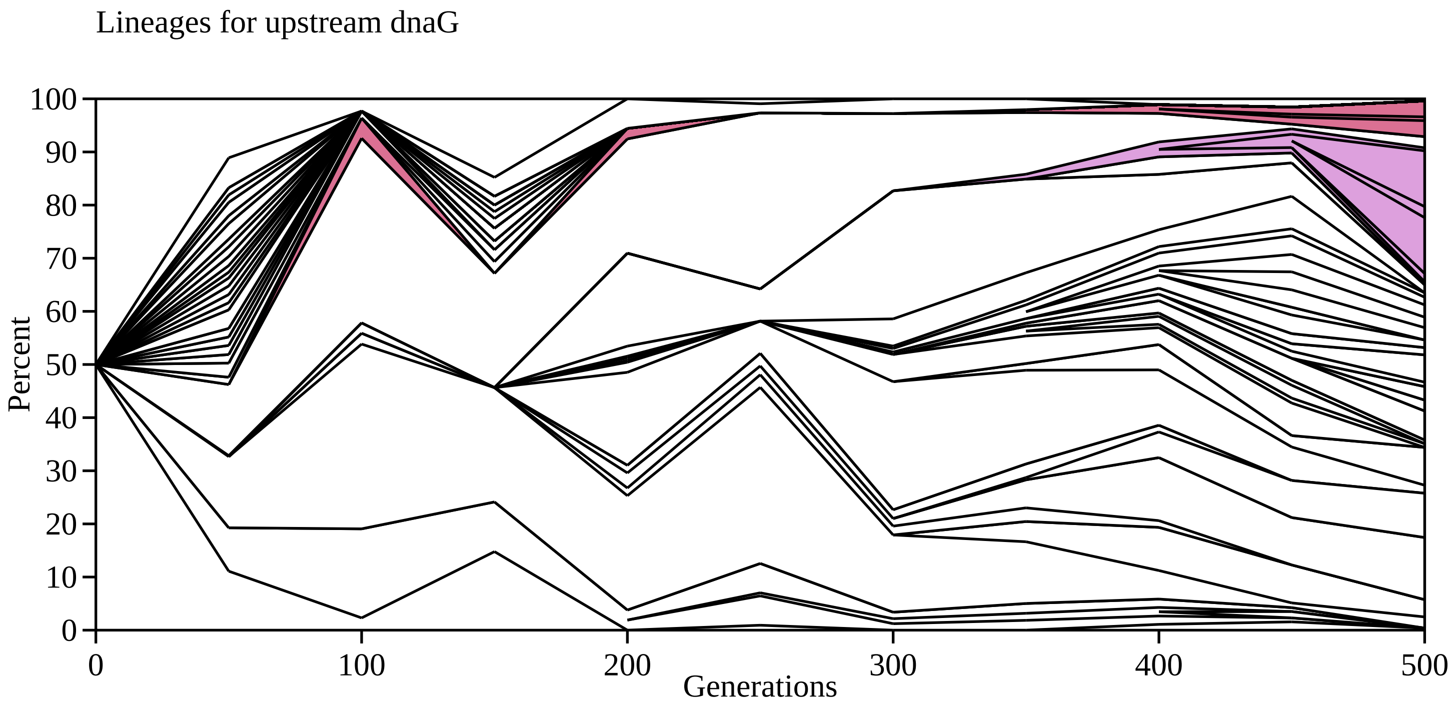
Lineages for upstream dnaG
Percent
0
10
20
30
40
50
60
70
80
90
100
0
100
200
300
400
500
Generations

Lineages for upstream mglB

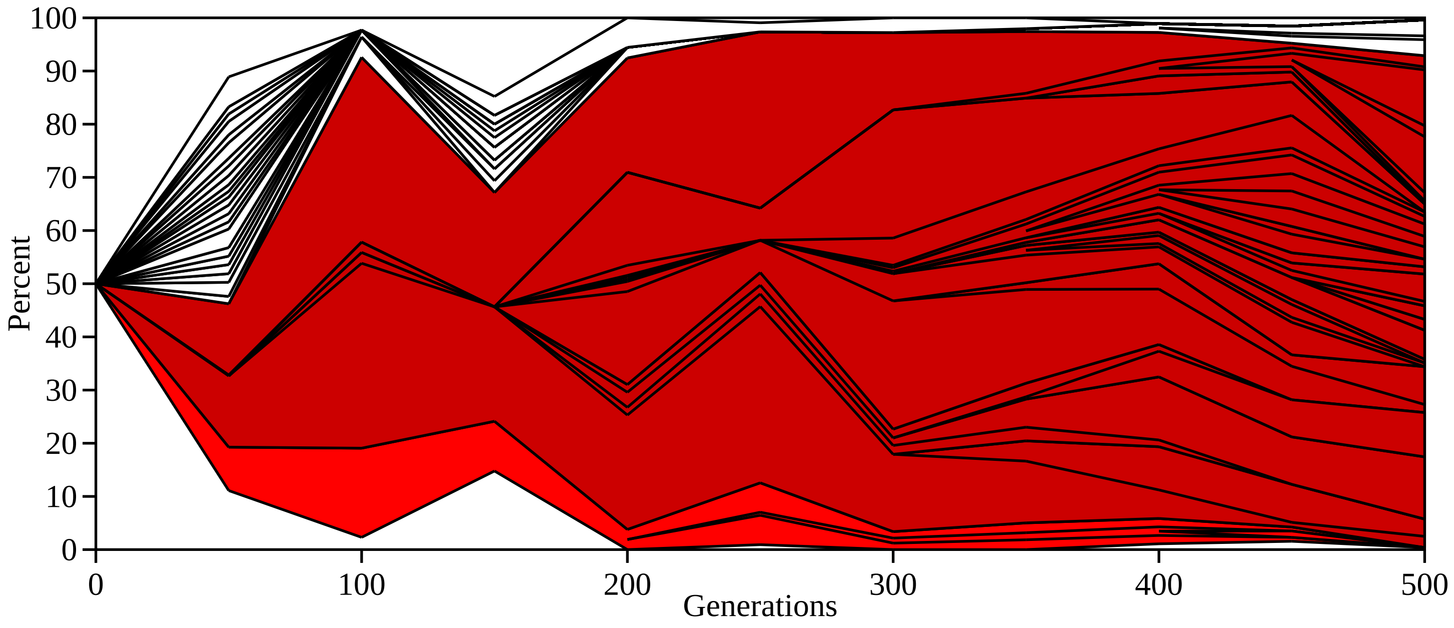

Lineages for wzzE

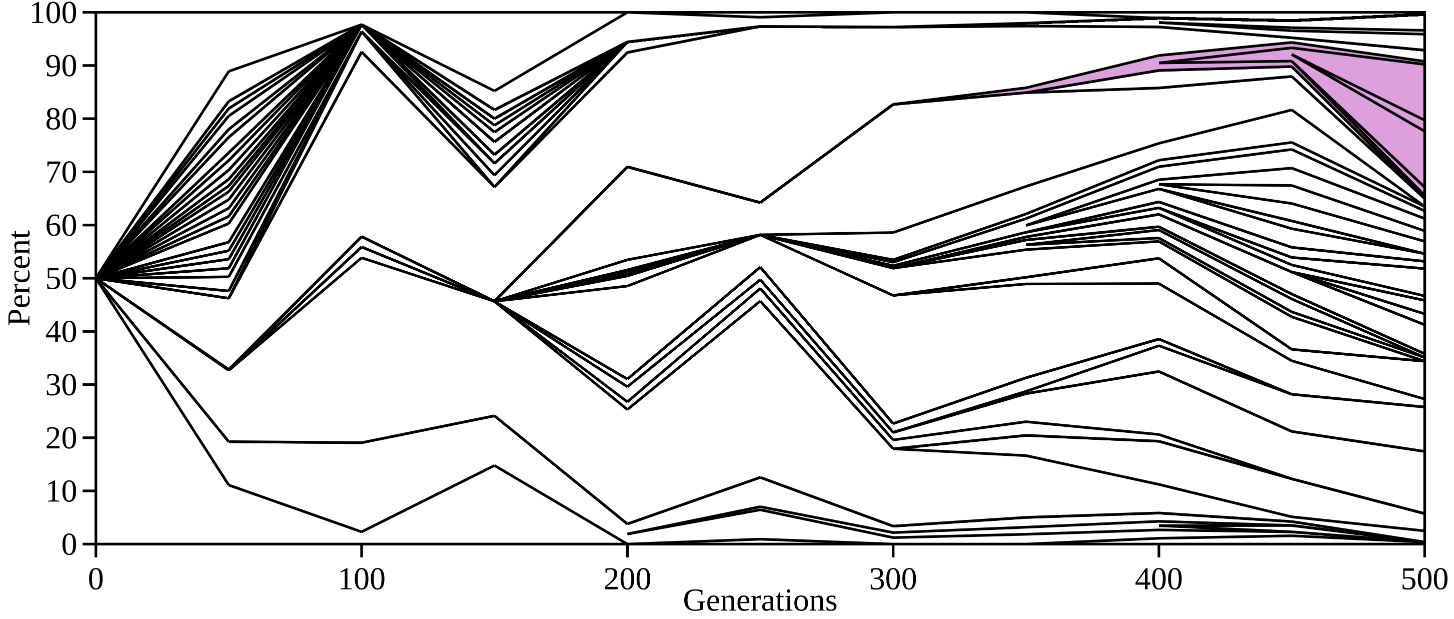

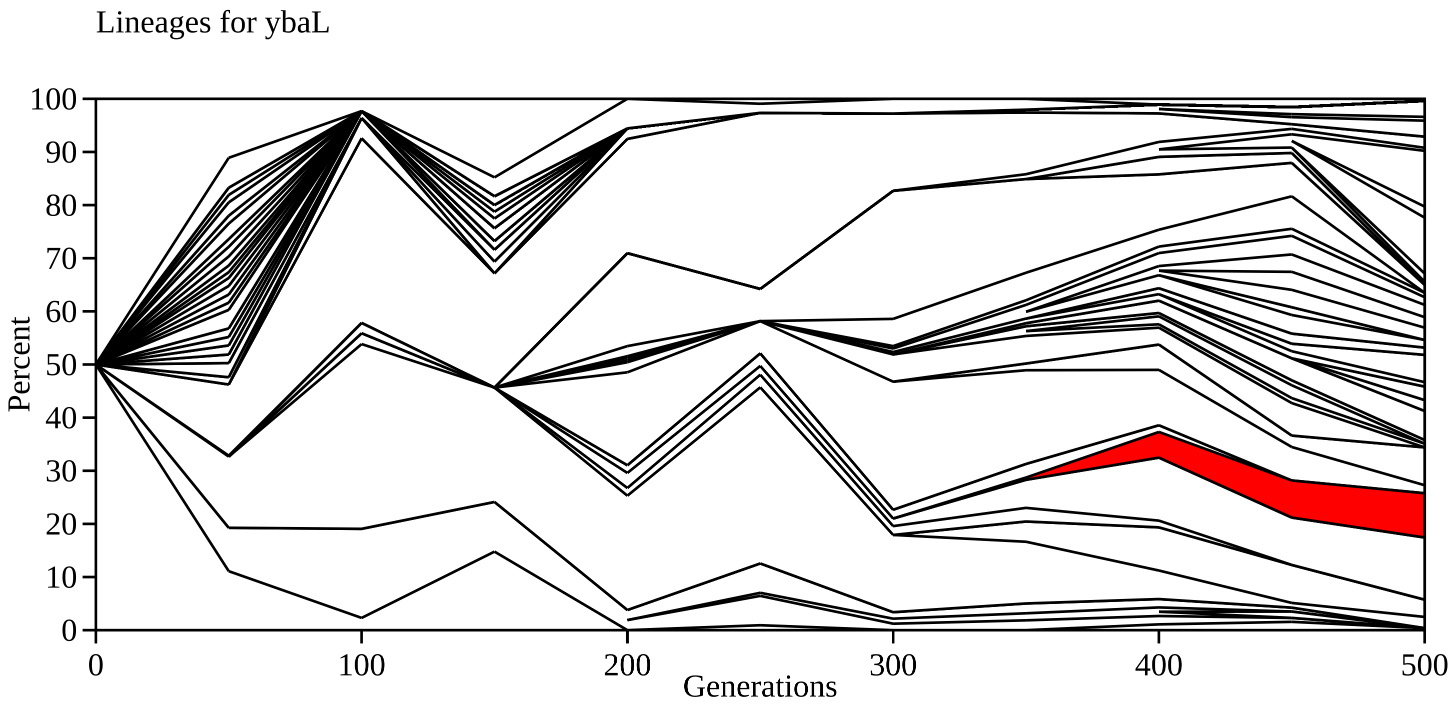

Lineages for ybaL
Percent
Generations
0
10
20
30
40
50
60
70
80
90
100
0
100
200
300
400
500

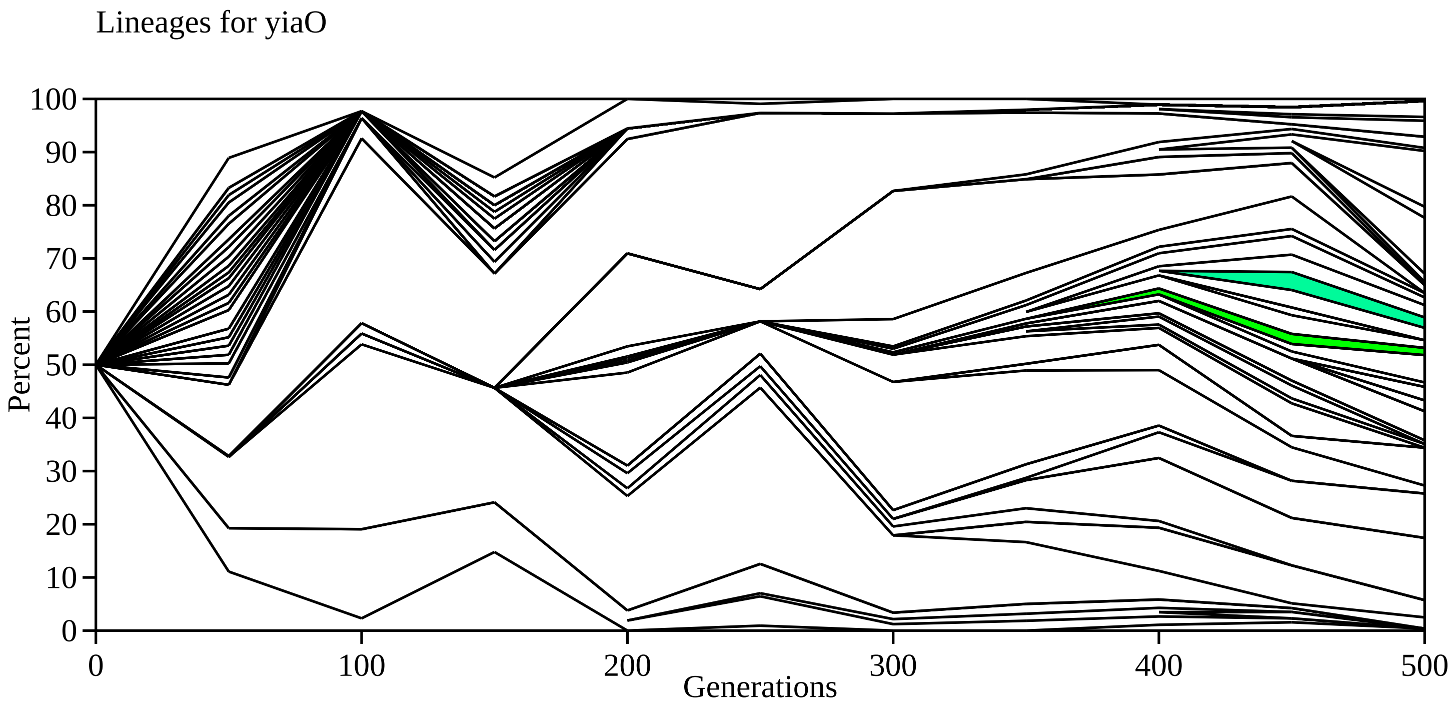

Lineages for yiaO
100
90
80
70
60
50
40
30
20
10
0
Percent
0
100
200
300
400
500
Generations

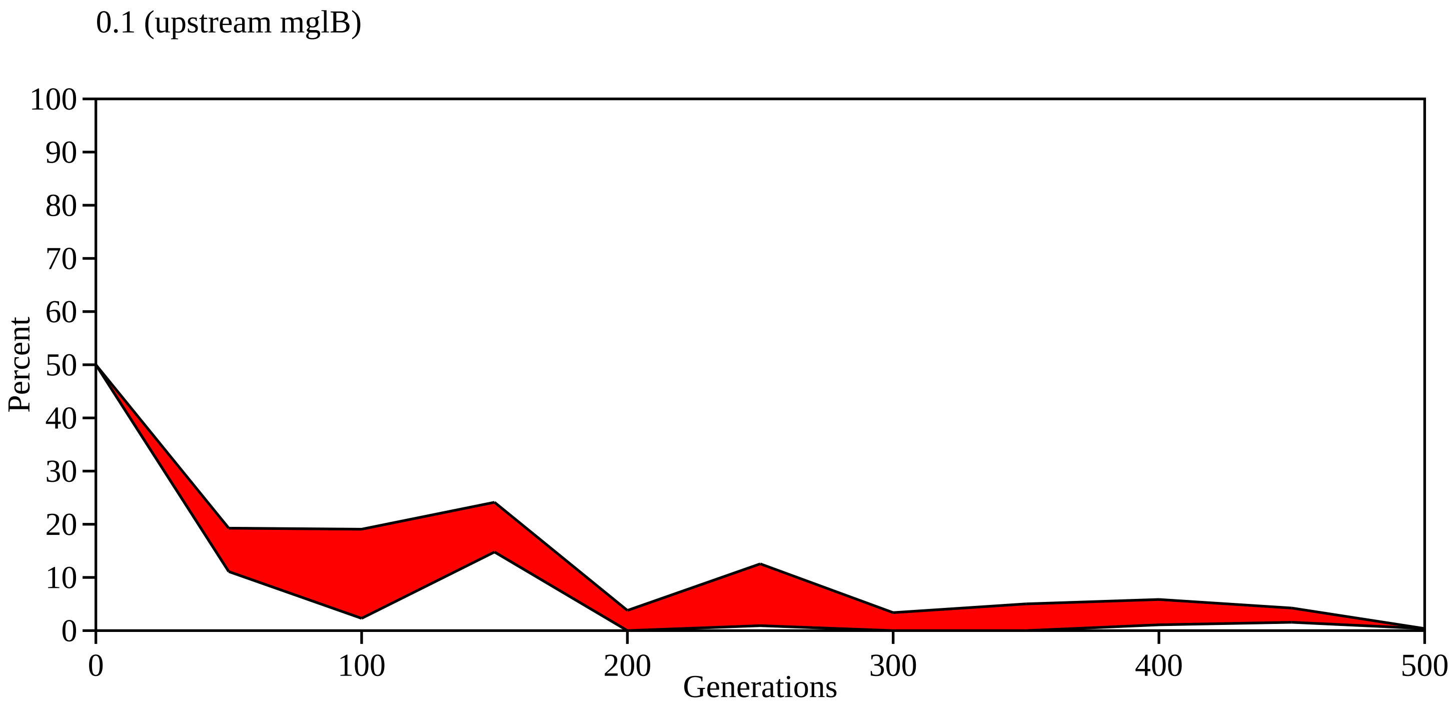
0.1 (upstream mglB)
100
90
80
70
60
50
40
30
20
10
0
Percent
0
100
200
300
400
500
Generations

0.2 (upstream mglB)

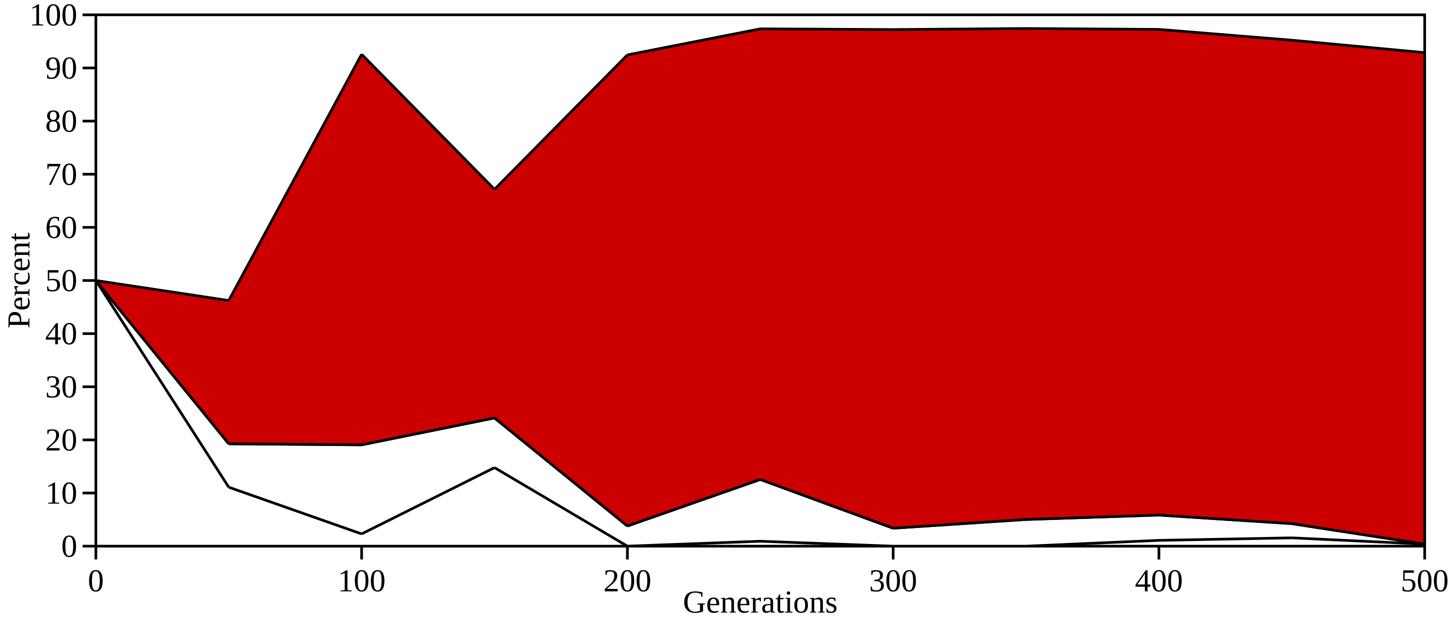

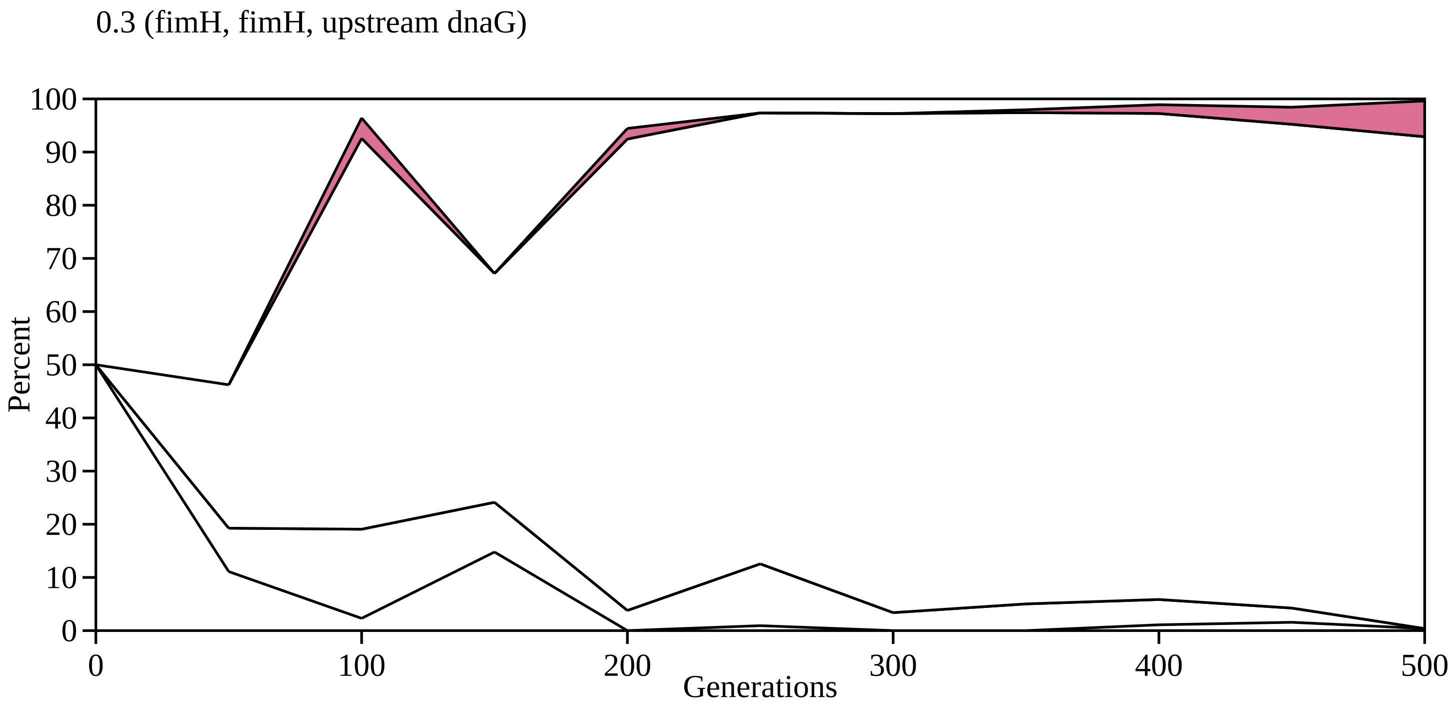
0.3 (fimH, fimH, upstream dnaG)
100
90
80
70
60
50
40
30
20
10
0
Percent
0
100
200
300
400
500
Generations

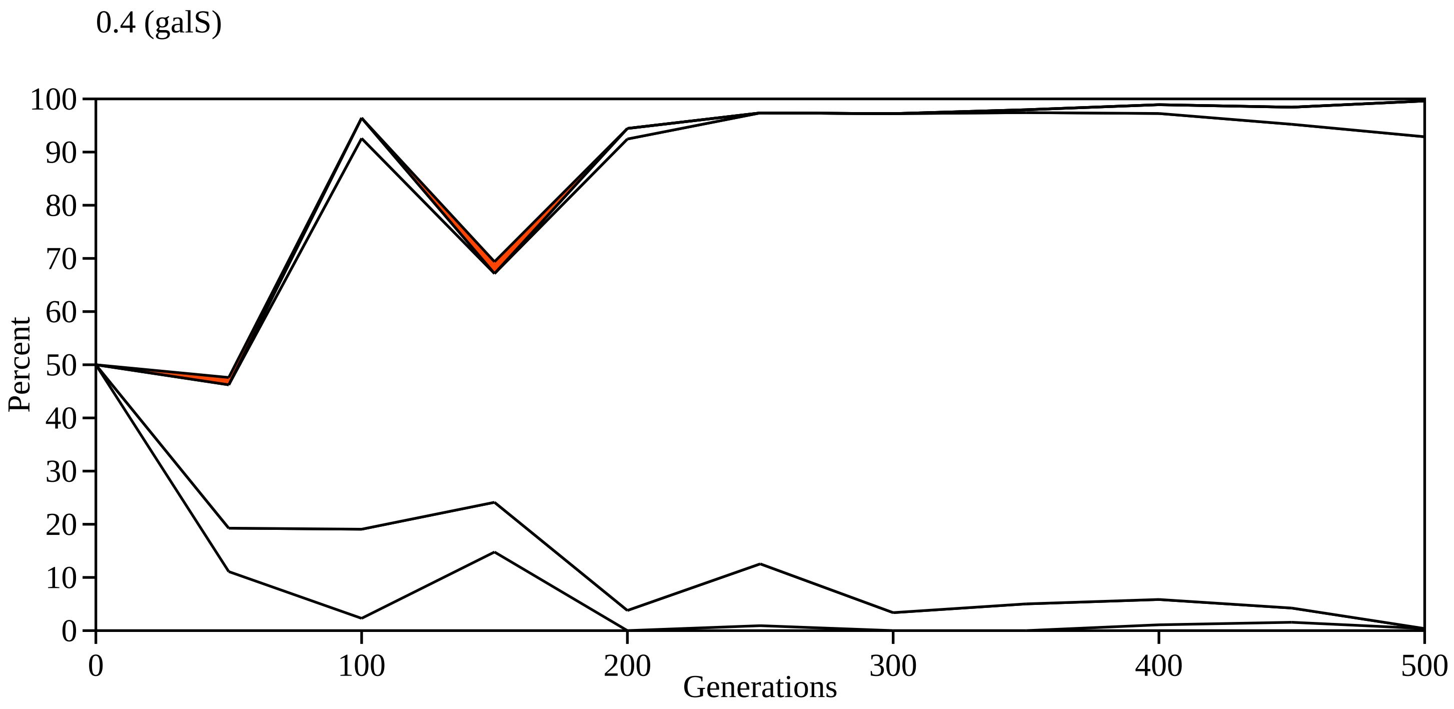
0.4 (galS)
Percent
Generations
100
90
80
70
60
50
40
30
20
10
0
0
100
200
300
400
500

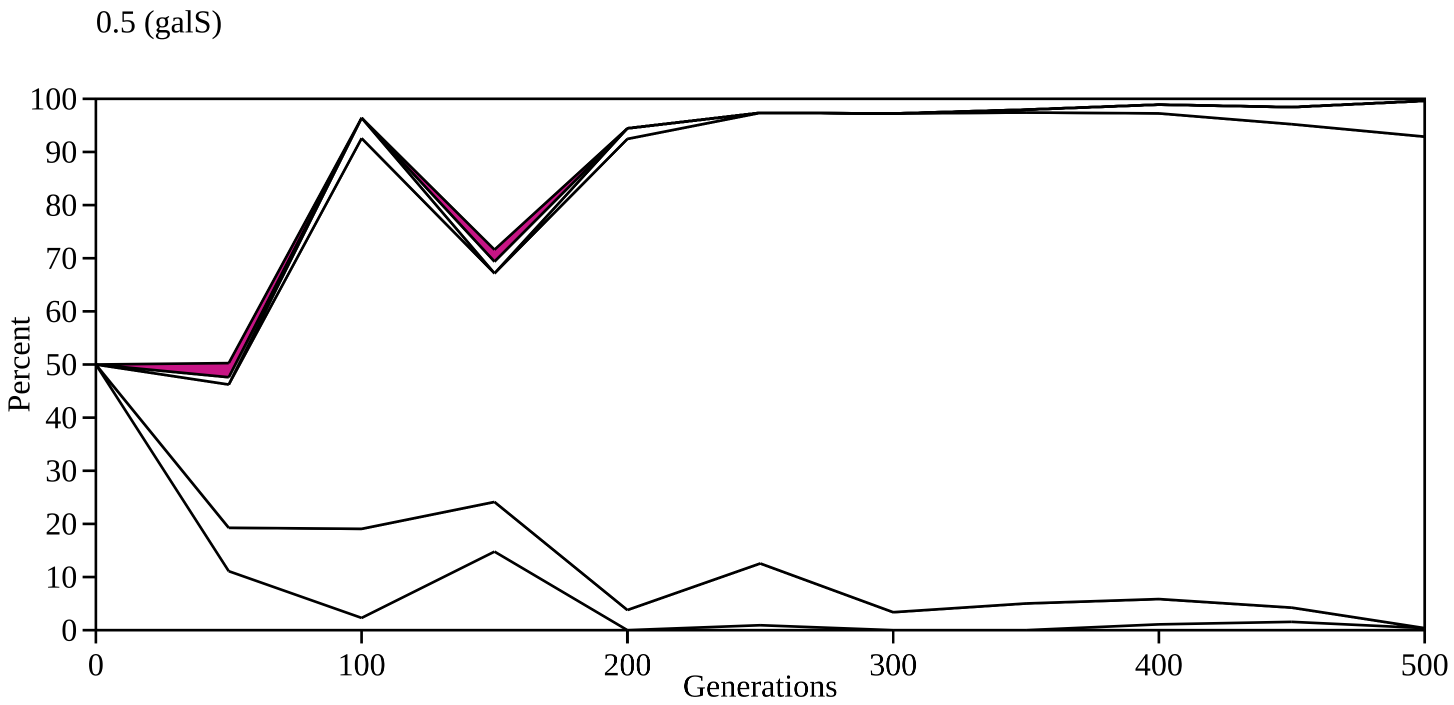
0.5 (galS)
100
90
80
70
60
50
40
30
20
10
0
Percent
0
100
200
300
400
500
Generations

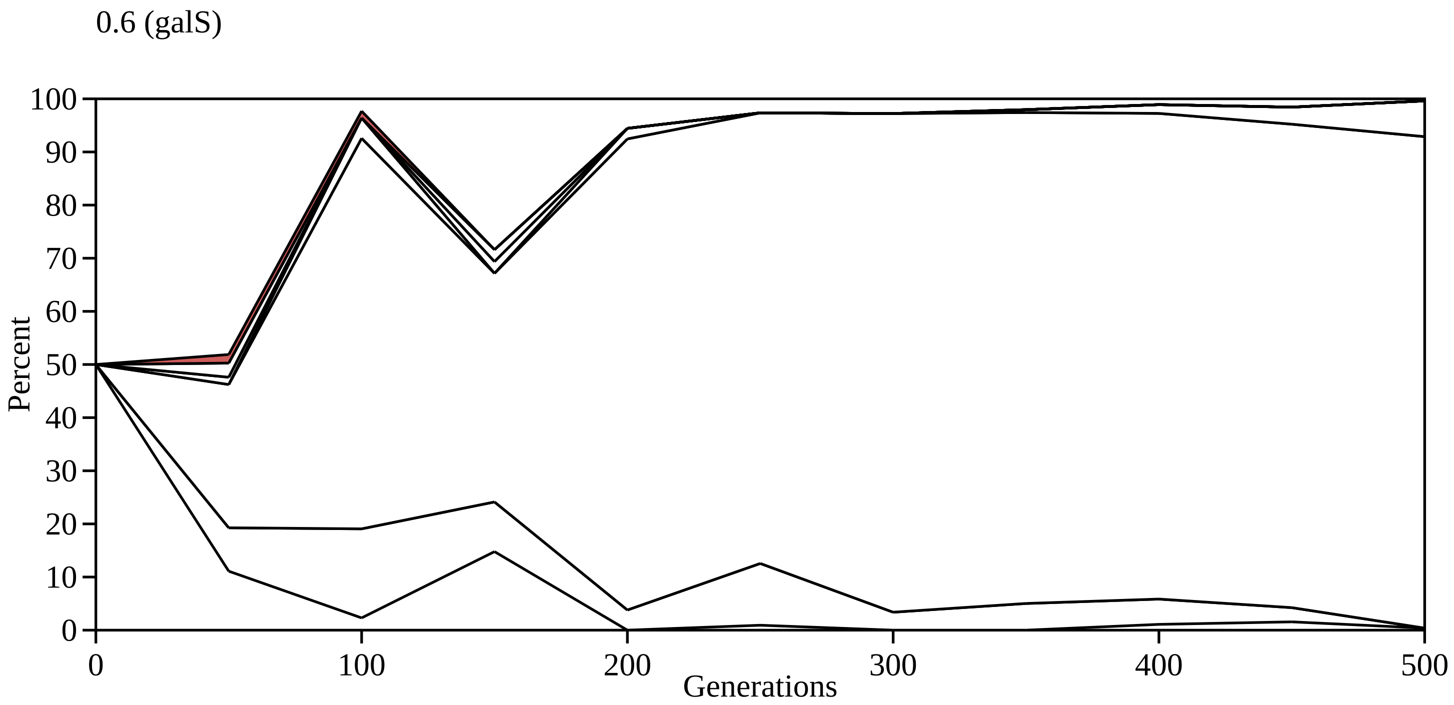
0.6 (galS)
Percent
Generations
100
90
80
70
60
50
40
30
20
10
0
0
100
200
300
400
500

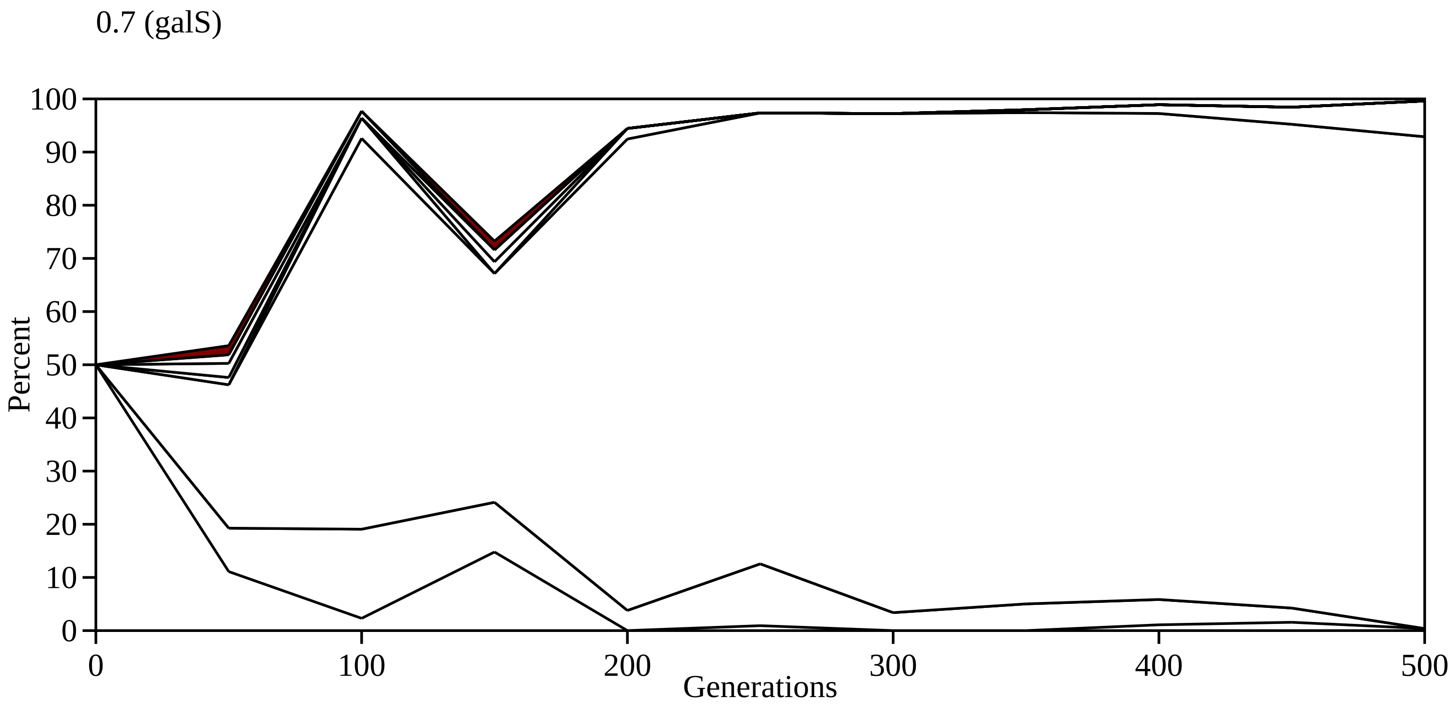

0.7 (galS)
Percent
Generations
100
90
80
70
60
50
40
30
20
10
0
0
100
200
300
400
500

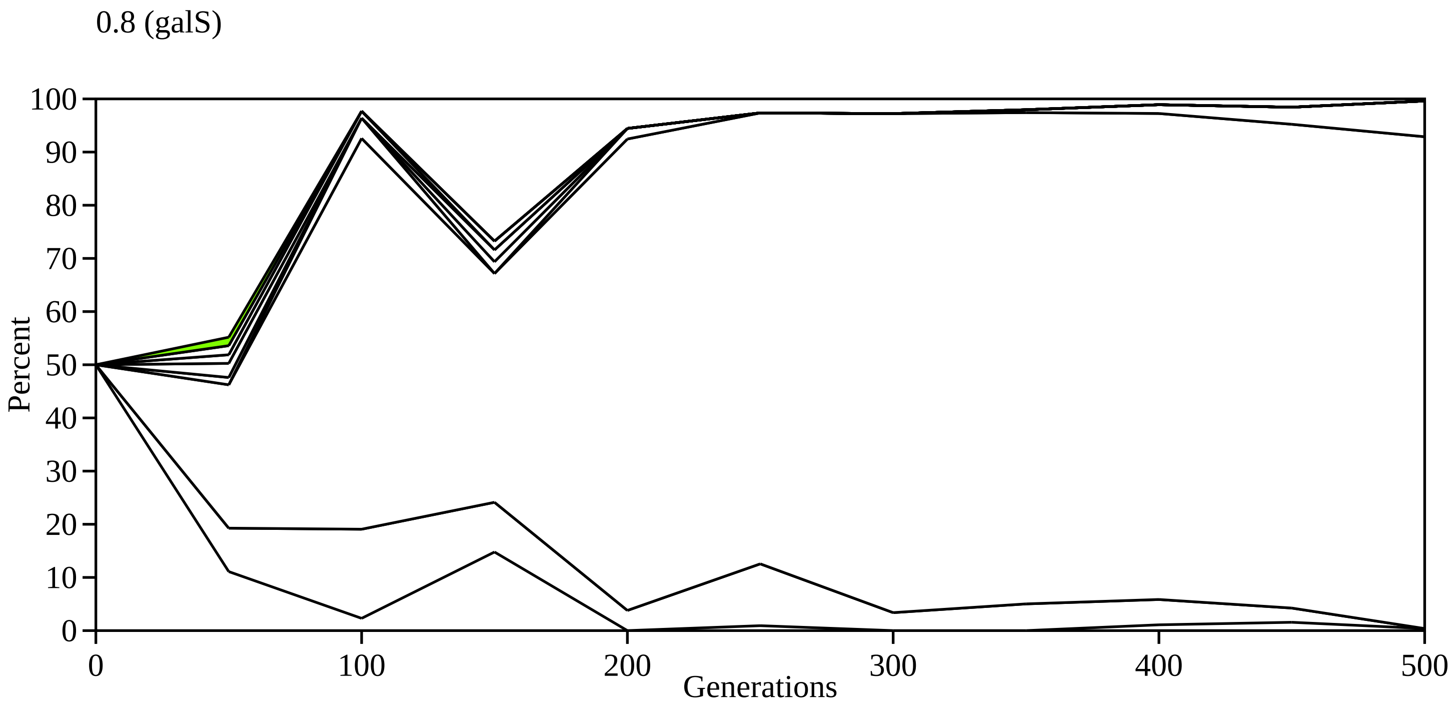
0.8 (galS)
Percent
Generations
100
90
80
70
60
50
40
30
20
10
0
0
100
200
300
400
500

0.9 (galS)

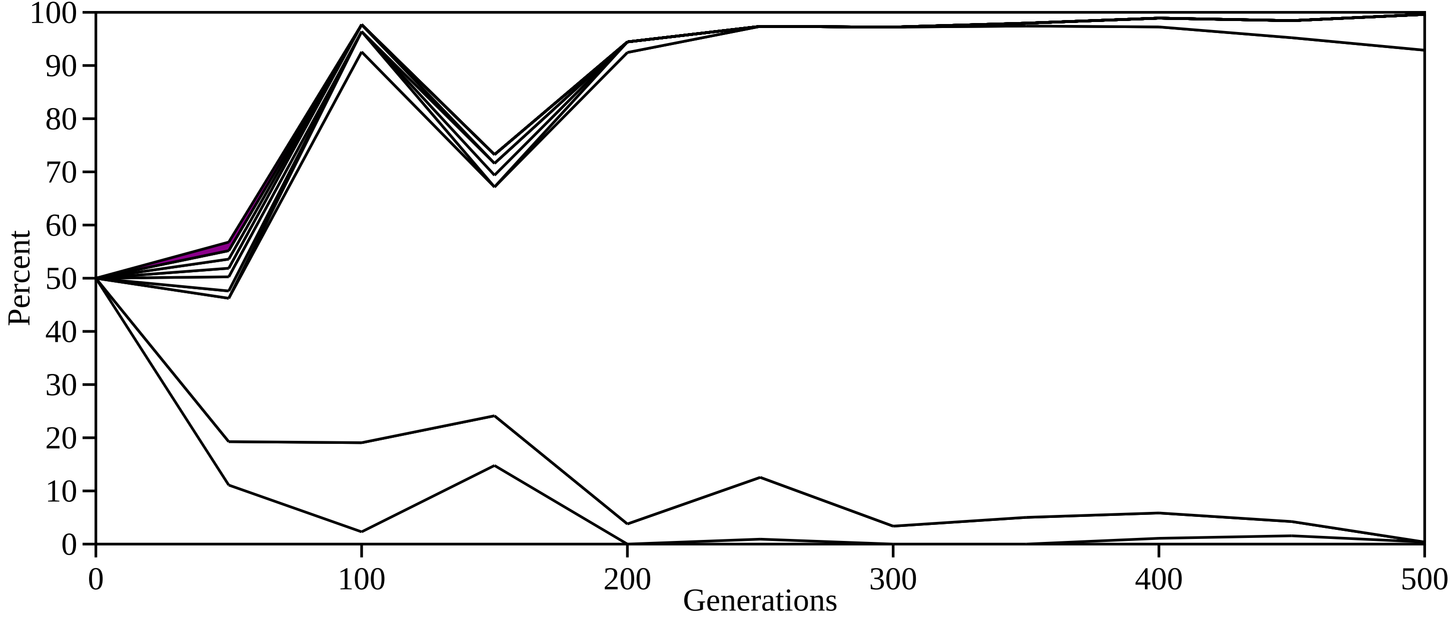

100
90
80
70
60
50
40
30
20
10
0
Percent
0
100
200
300
400
500
Generations

0.10 (galS)

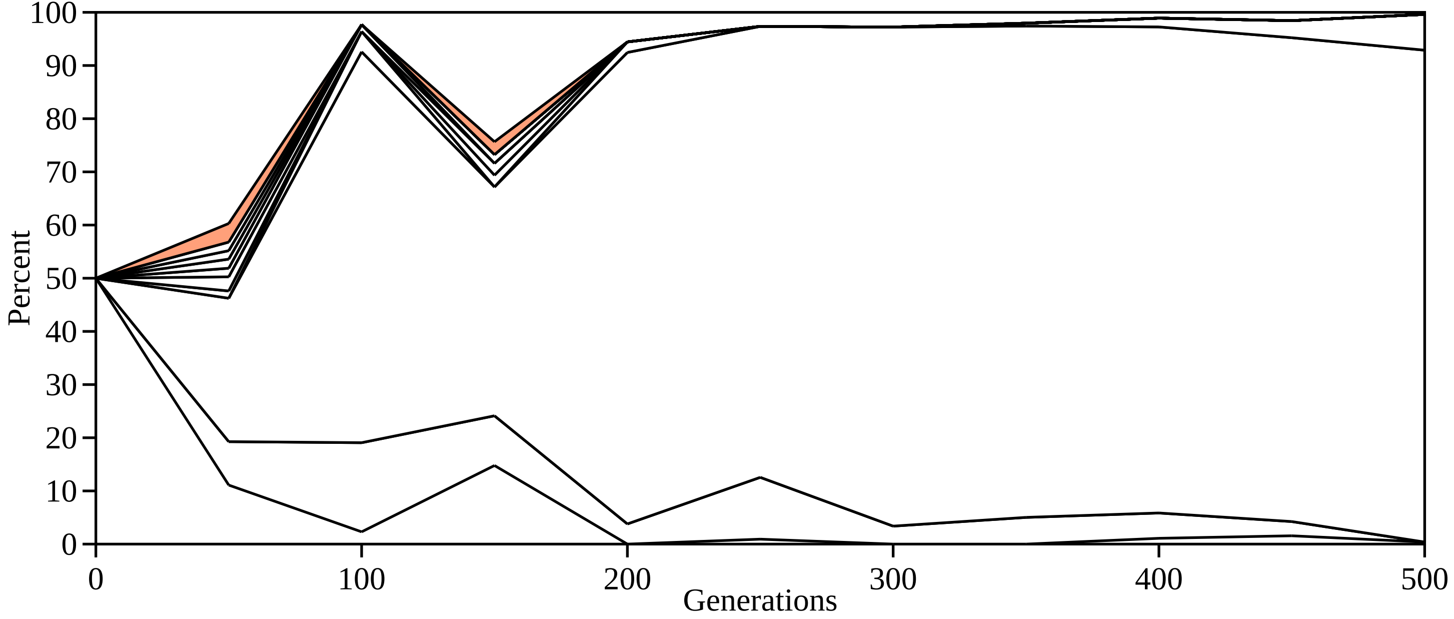

100
90
80
70
60
50
40
30
20
10
0
Percent
0
100
200
300
400
500
Generations

0.11 (galS)

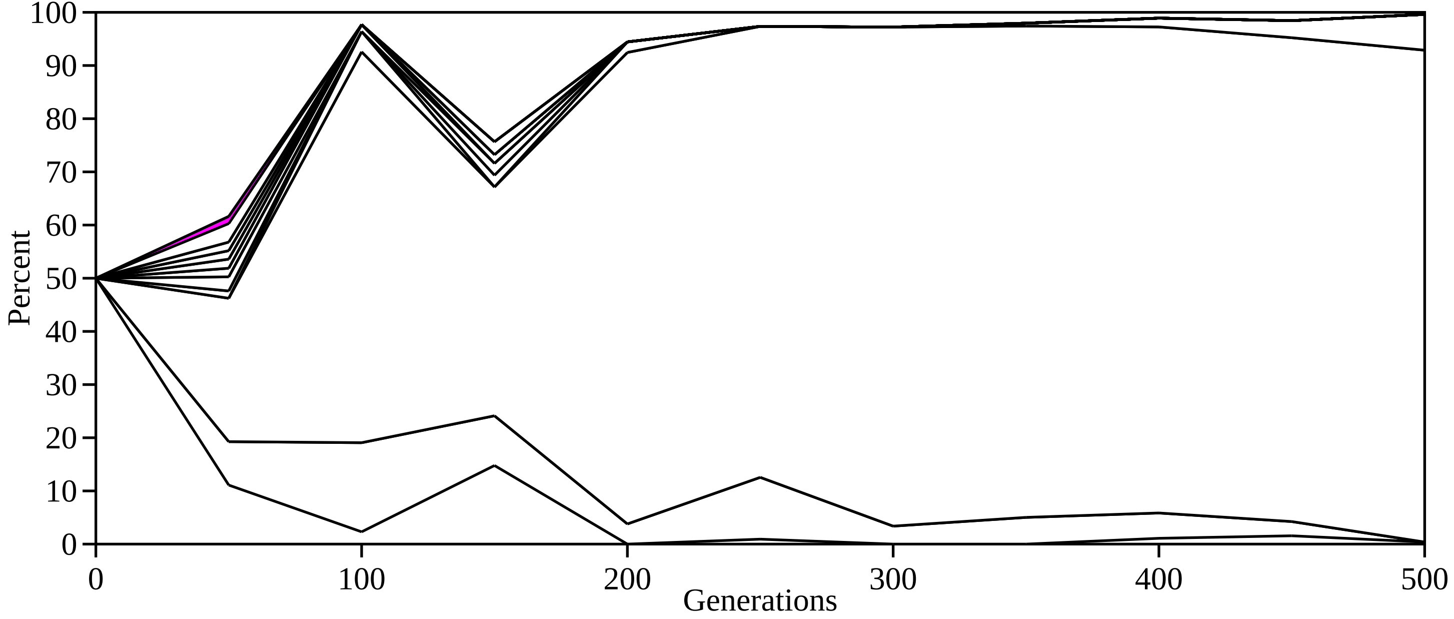

100
90
80
70
60
50
40
30
20
10
0
Percent
0
100
200
300
400
500
Generations

0.12 (galS)

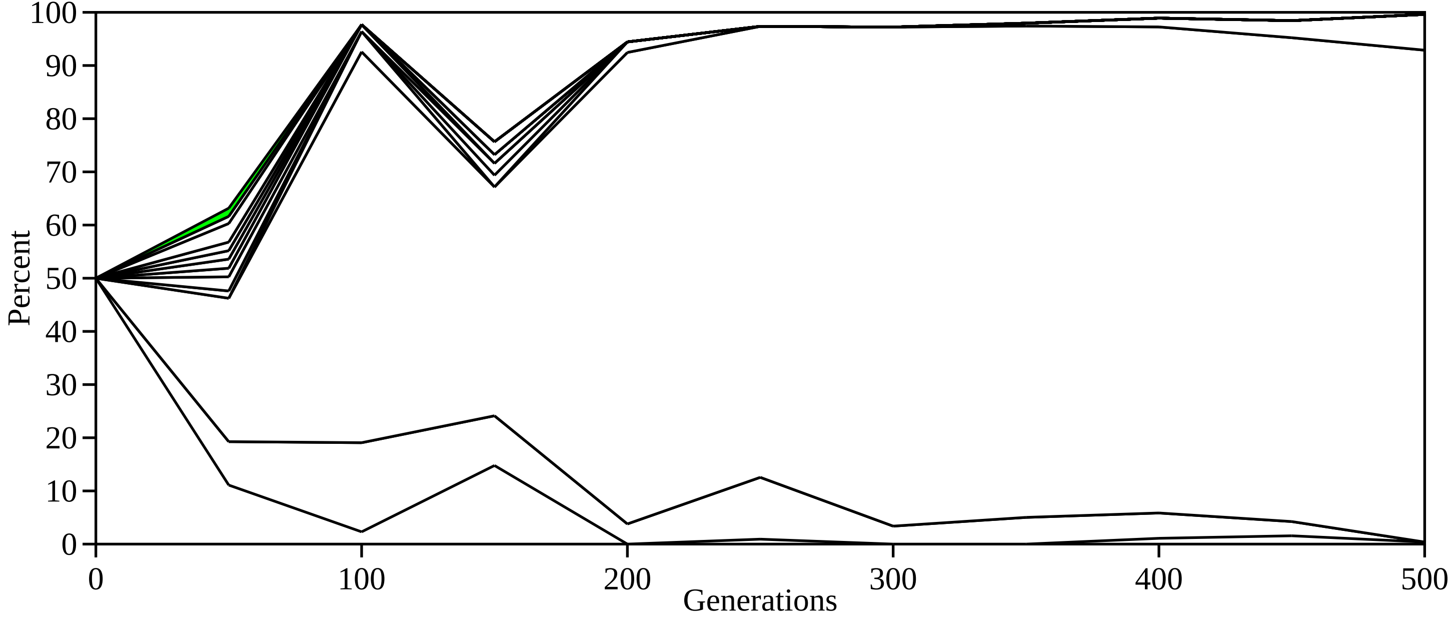

100
90
80
70
60
50
40
30
20
10
0
Percent
0
100
200
300
400
500
Generations

0.13 (galS)

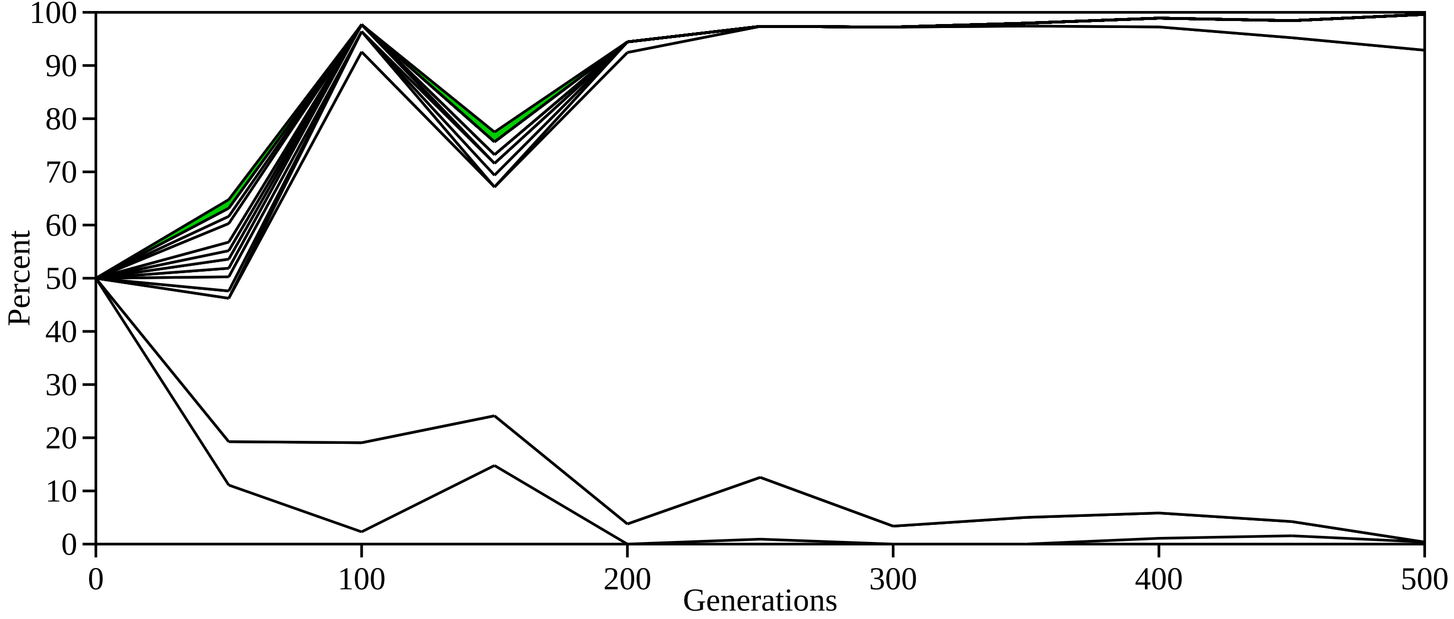

100
90
80
70
60
50
40
30
20
10
0
Percent
0
100
200
300
400
500
Generations

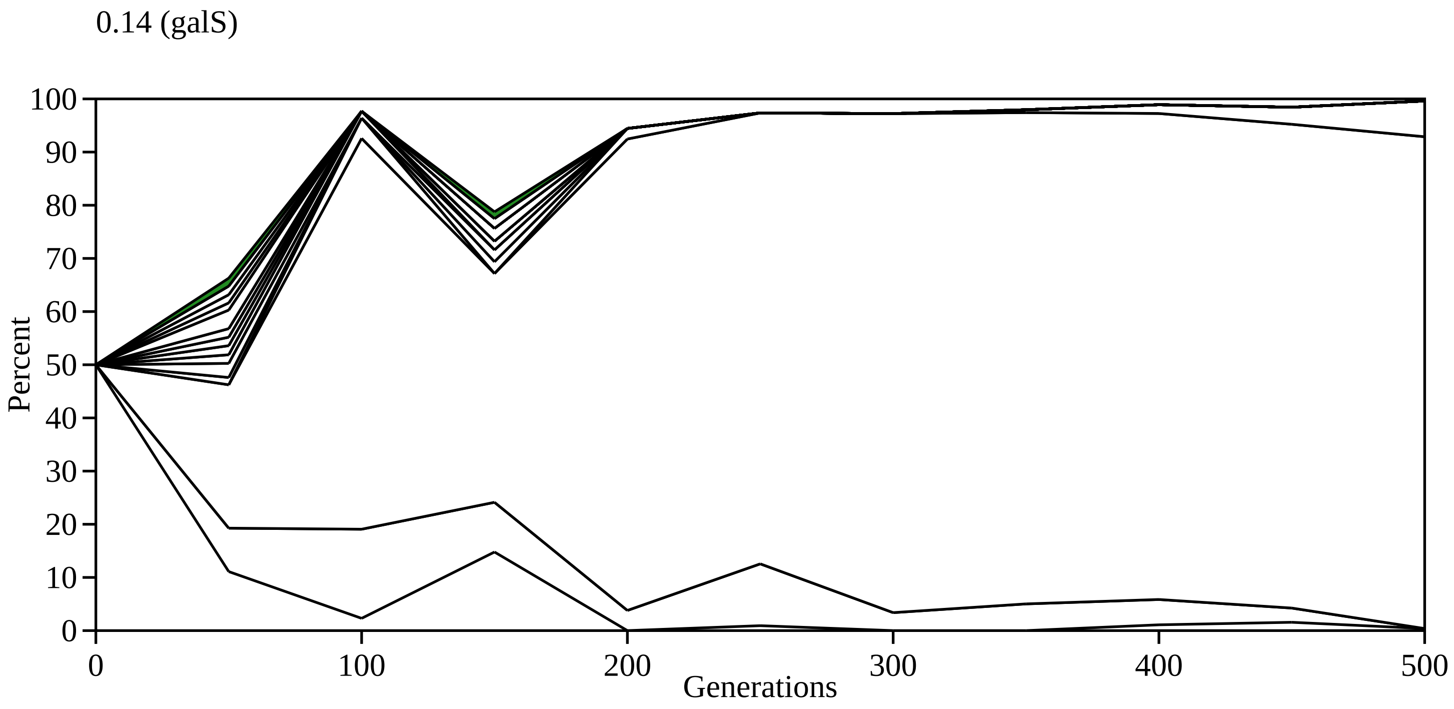

0.14 (galS)
Percent
Generations
0
10
20
30
40
50
60
70
80
90
100
0
100
200
300
400
500

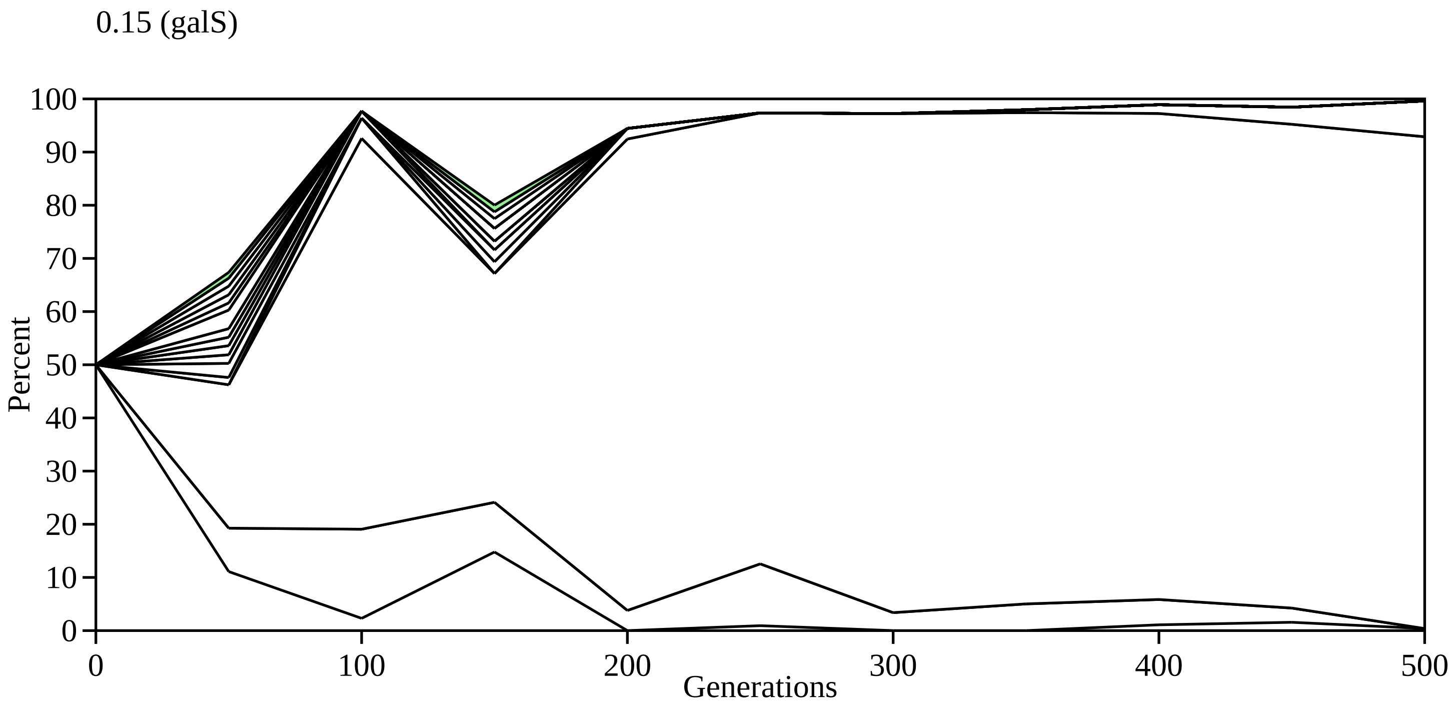
0.15 (galS)
Percent
Generations
0
10
20
30
40
50
60
70
80
90
100
0
100
200
300
400
500

0.16 (galS)

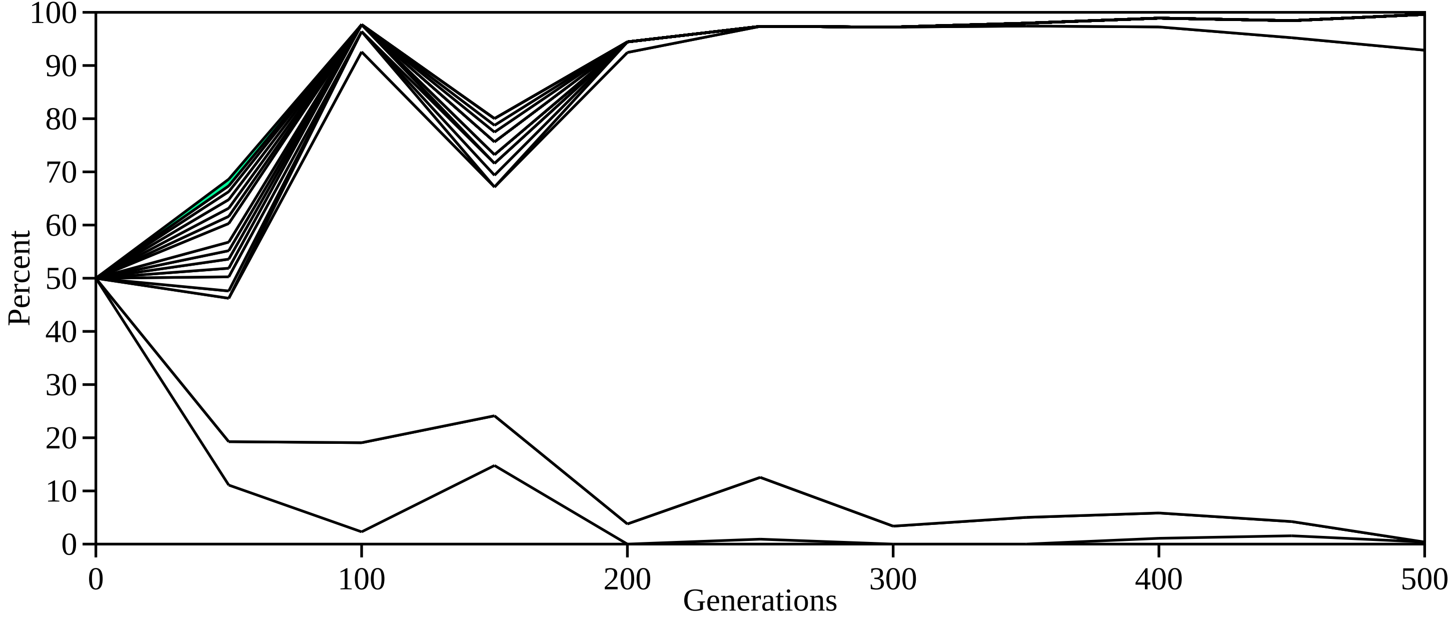

100
90
80
70
60
50
40
30
20
10
0
Percent
0
100
200
300
400
500
Generations

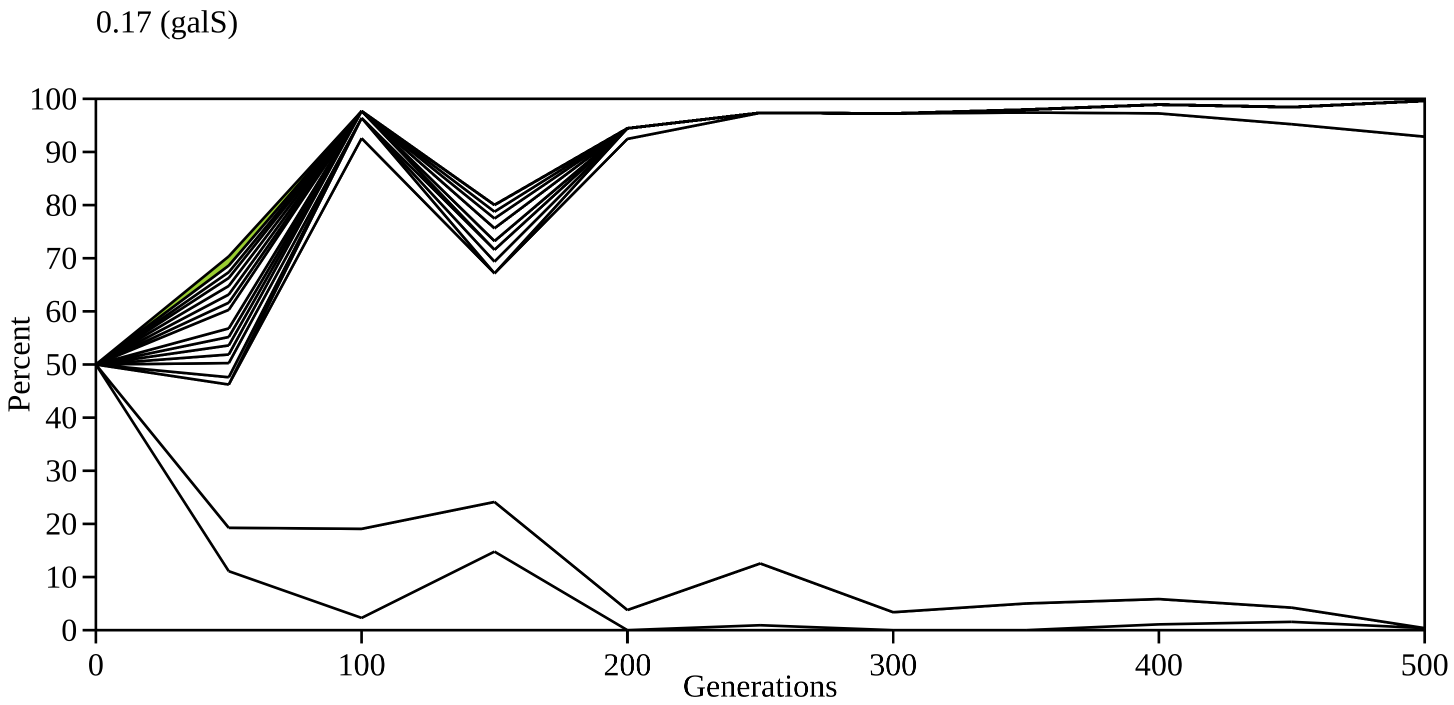
0.17 (galS)
Percent
Generations
100
90
80
70
60
50
40
30
20
10
0
0
100
200
300
400
500

0.18 (galS)

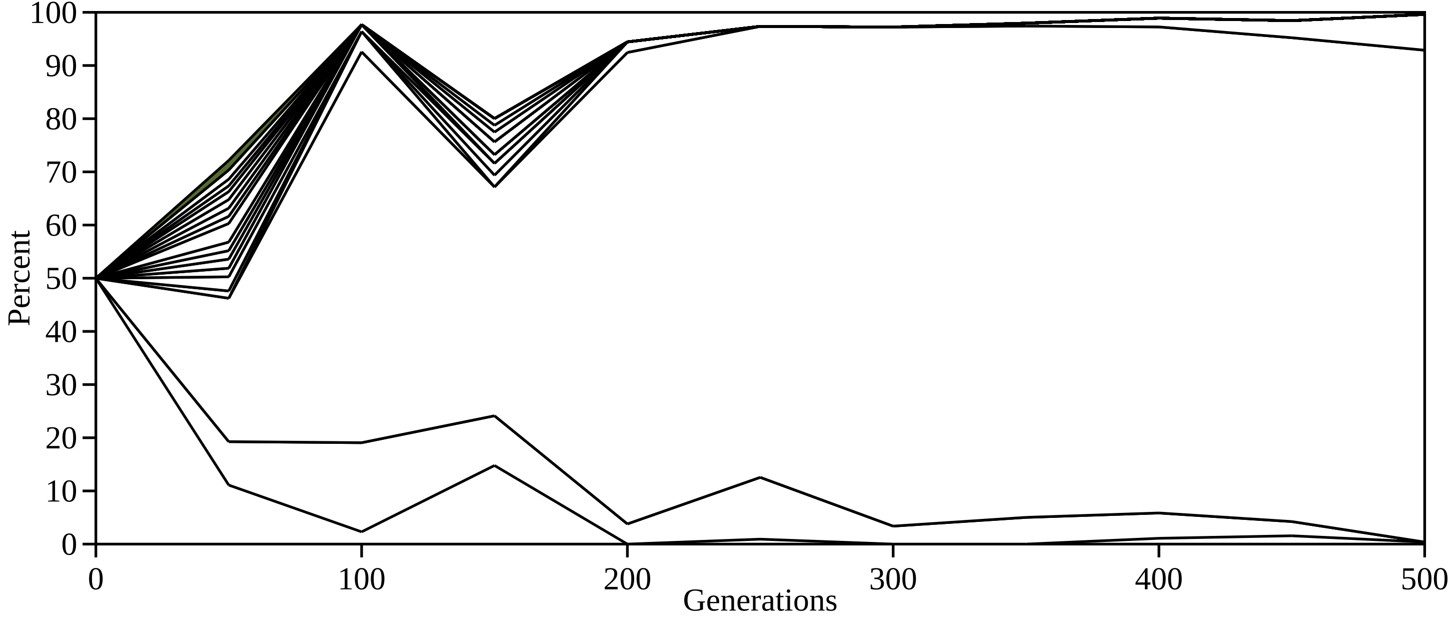

100
90
80
70
60
50
40
30
20
10
0
Percent
0
100
200
300
400
500
Generations

0.19 (galS)

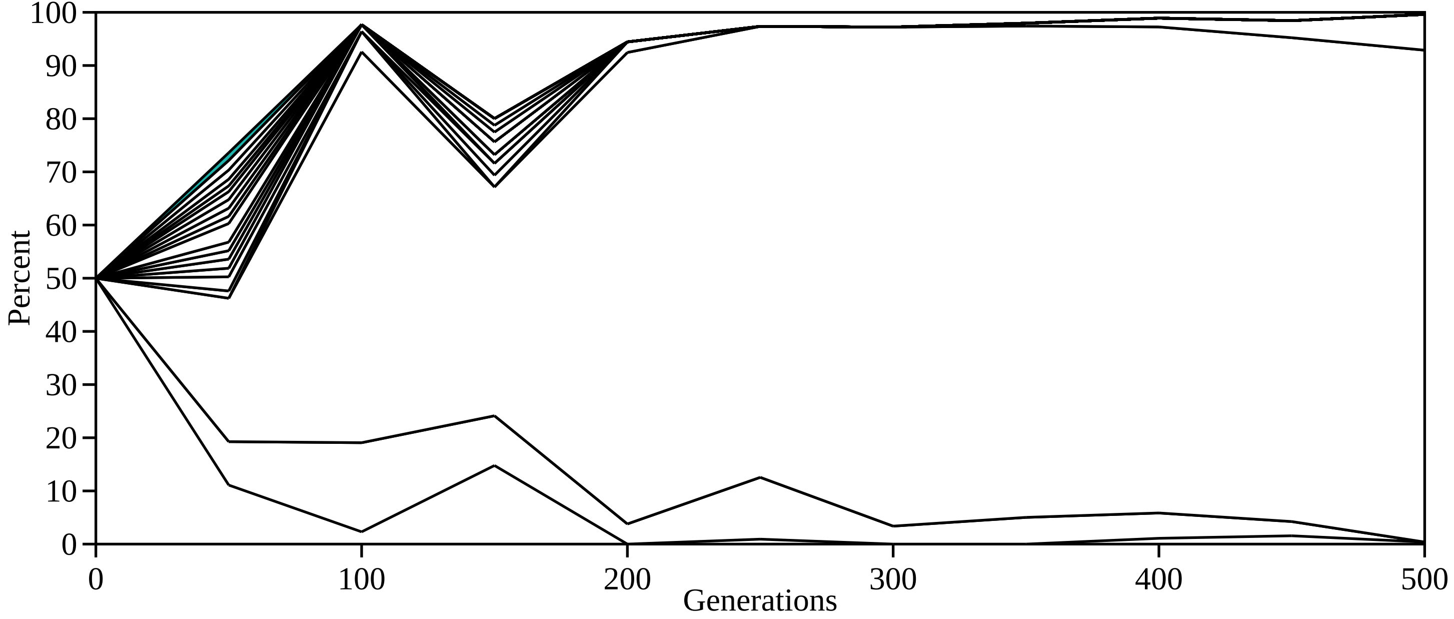

Percent
100
90
80
70
60
50
40
30
20
10
0
0
100
200
300
400
500
Generations

0.20 (galS)

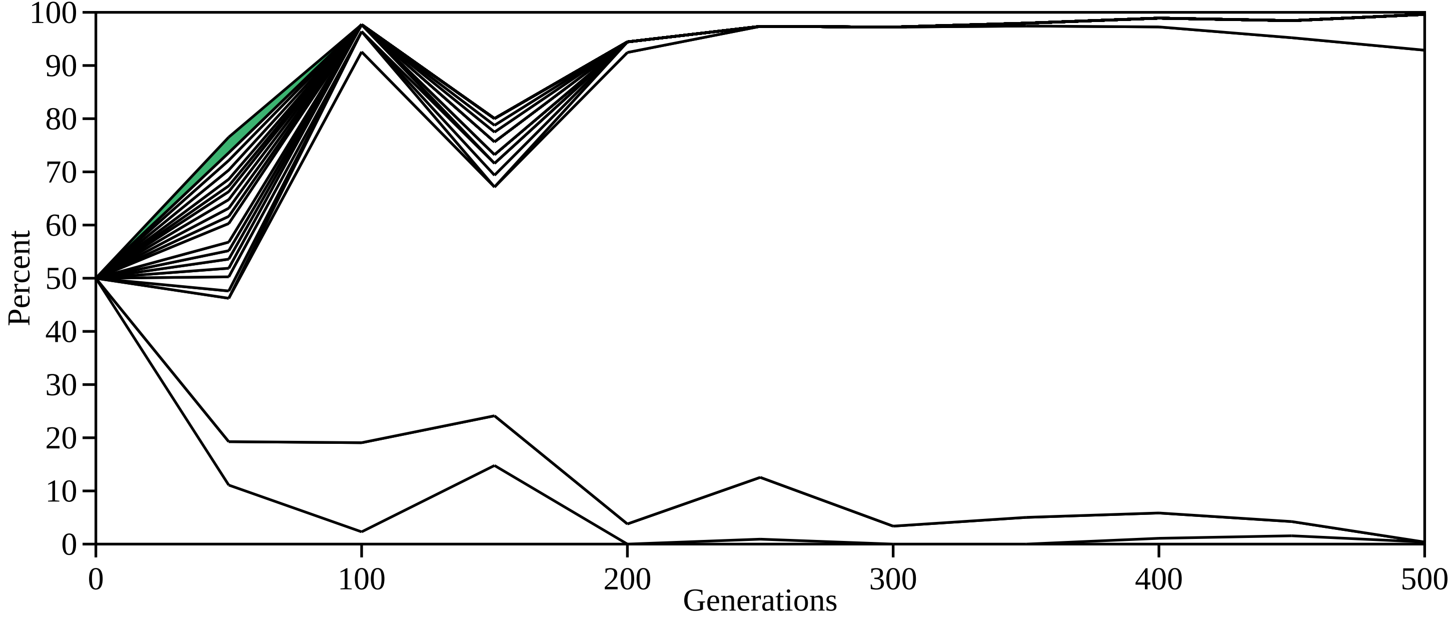

100
90
80
70
60
50
40
30
20
10
0
Percent
0
100
200
300
400
500
Generations

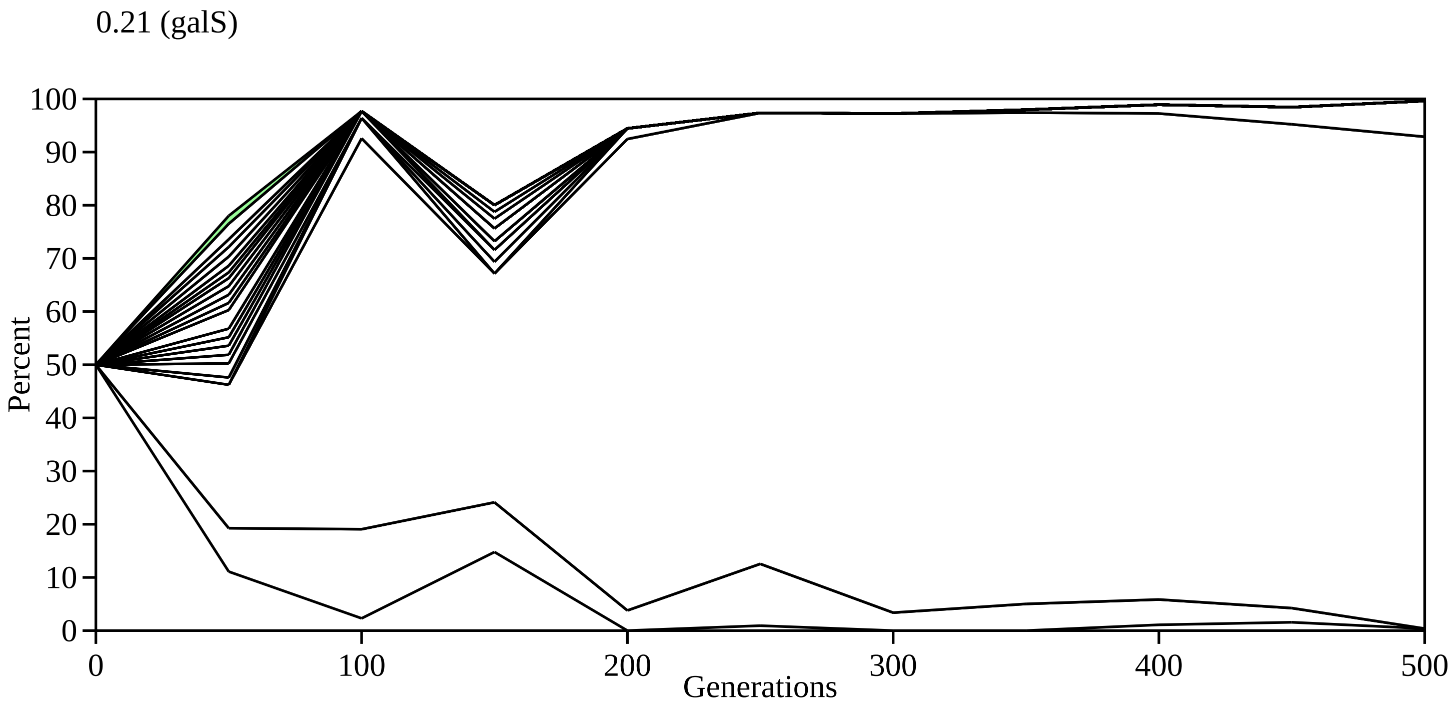
0.21 (galS)
Percent
100
90
80
70
60
50
40
30
20
10
0
0
100
200
300
400
500
Generations

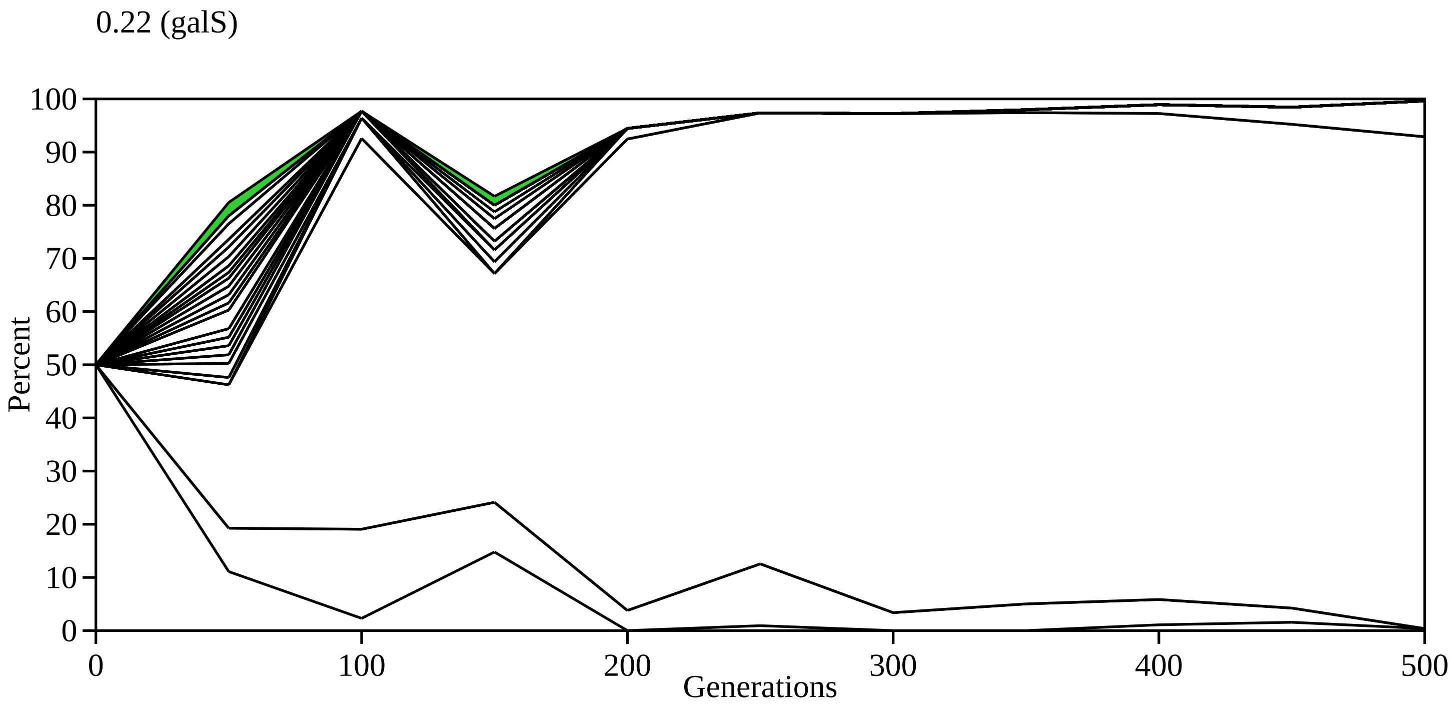
0.22 (galS)
Percent
Generations
100
90
80
70
60
50
40
30
20
10
0
0
100
200
300
400
500

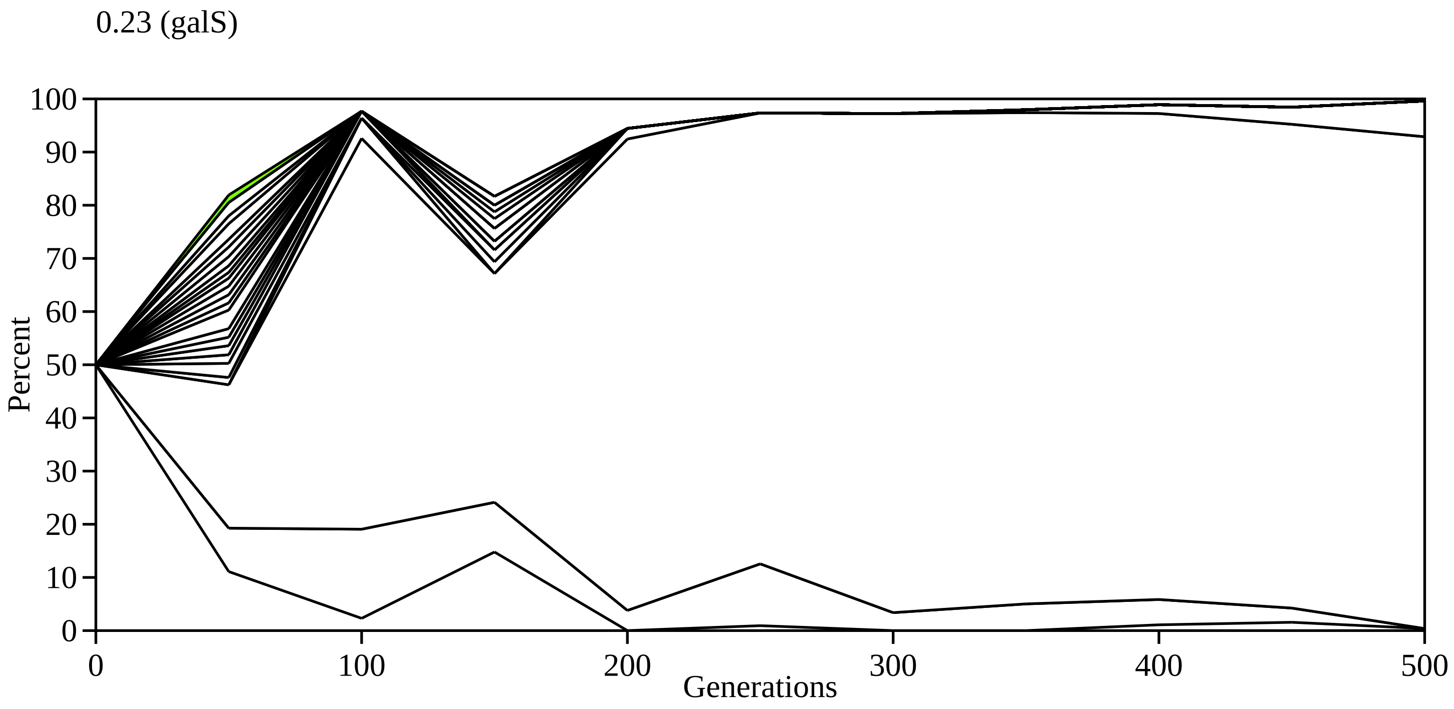

0.23 (galS)
Percent
100
90
80
70
60
50
40
30
20
10
0
0
100
200
300
400
500
Generations

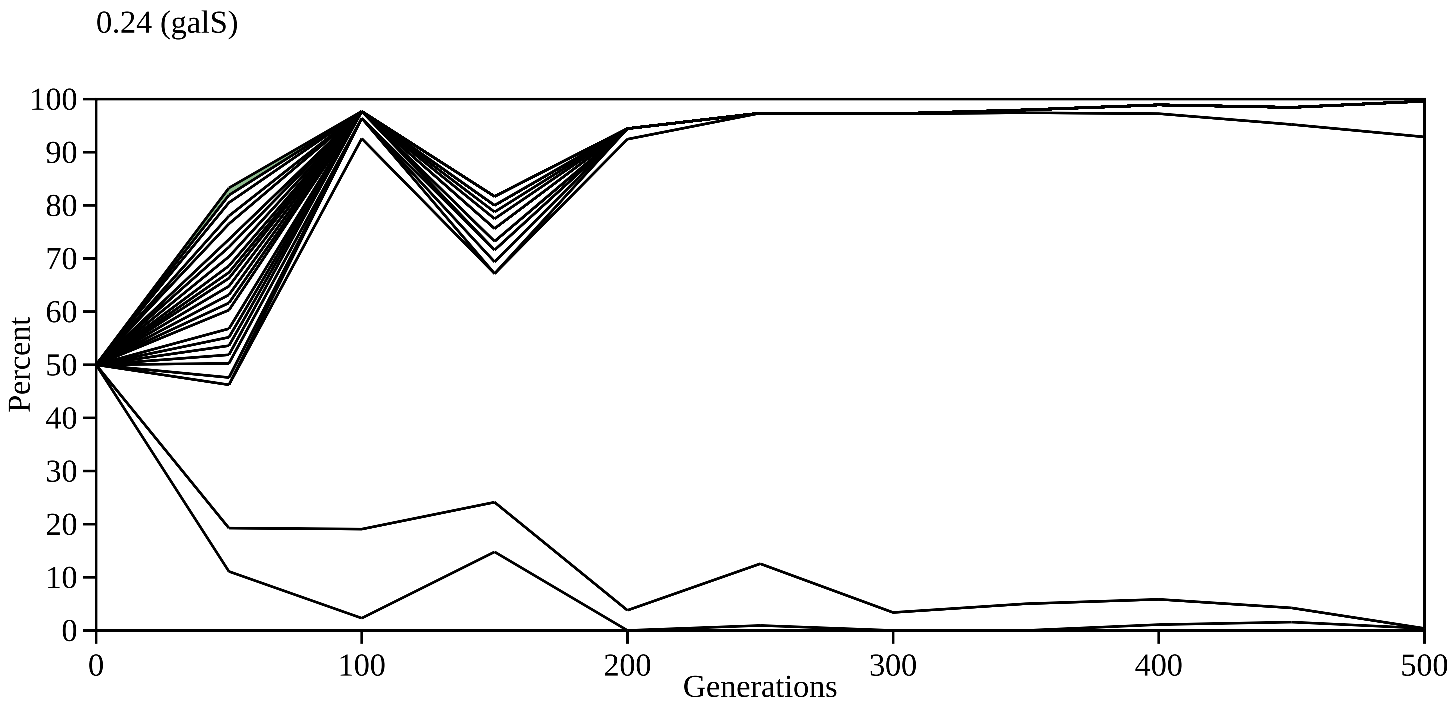

0.24 (galS)
Percent
Generations
0
10
20
30
40
50
60
70
80
90
100
0
100
200
300
400
500

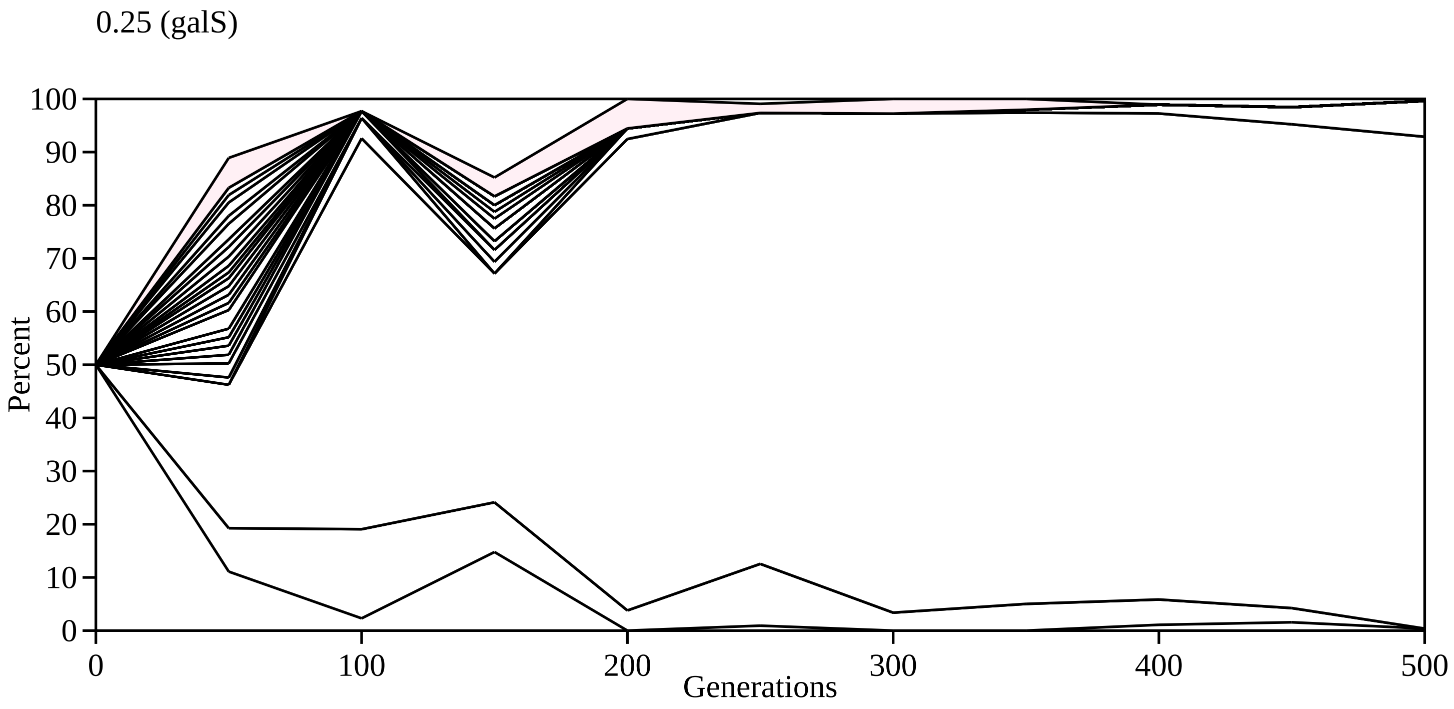

0.25 (galS)
Percent
100
90
80
70
60
50
40
30
20
10
0
0
100
200
300
400
500
Generations

0.1.1 (hfq, malT)

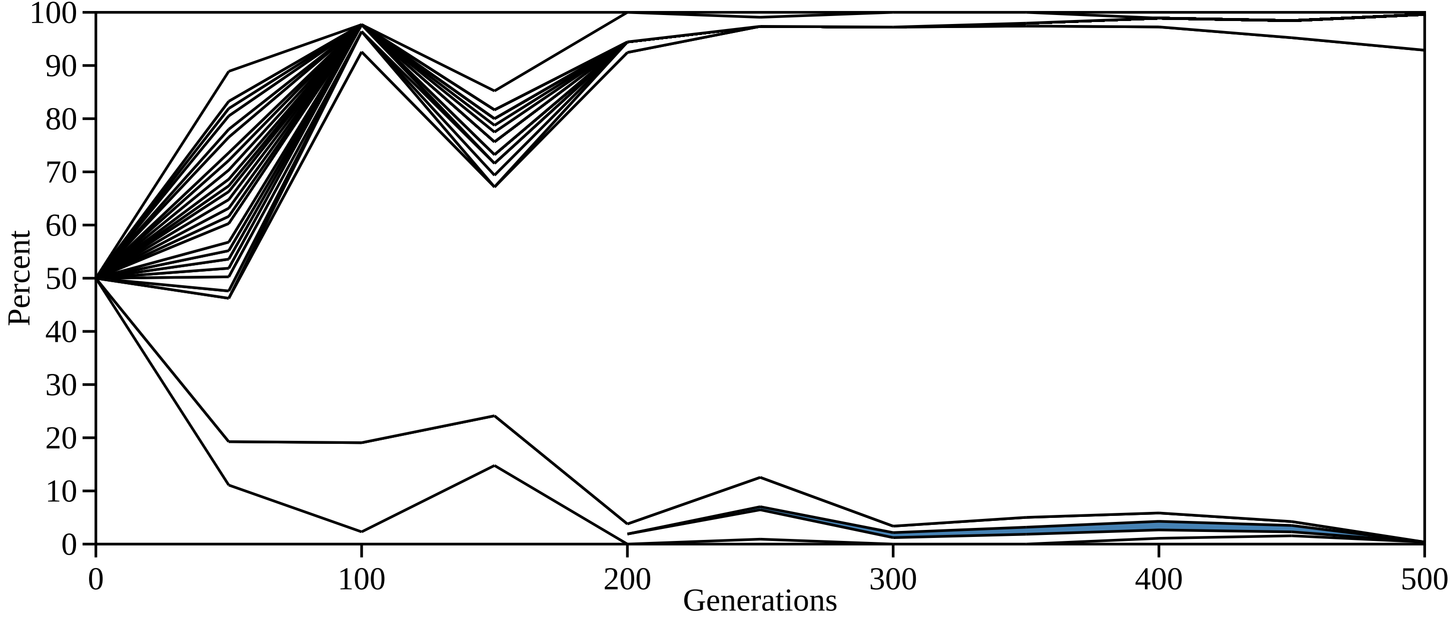

100
90
80
70
60
50
40
30
20
10
0
Percent
0
100
200
300
400
500
Generations

0.1.1.1 (fimH)

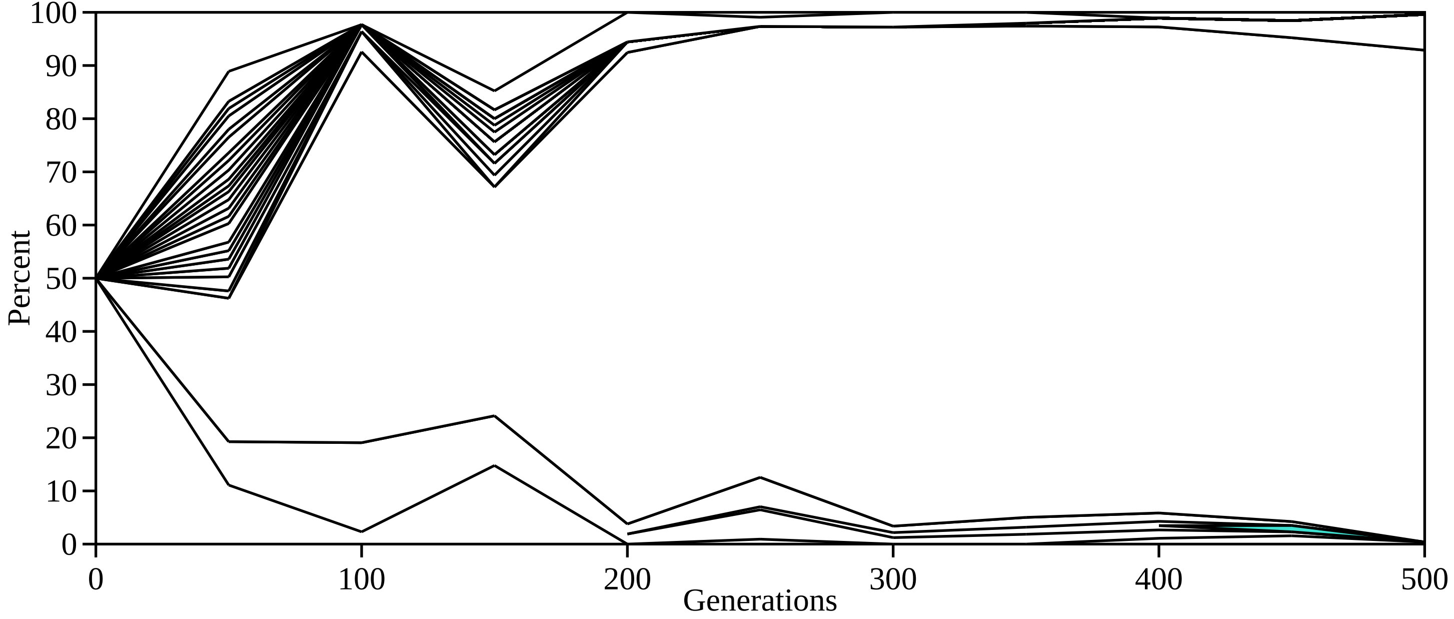

100
90
80
70
60
50
40
30
20
10
0
Percent
0
100
200
300
400
500
Generations

0.2.1 (hfq)

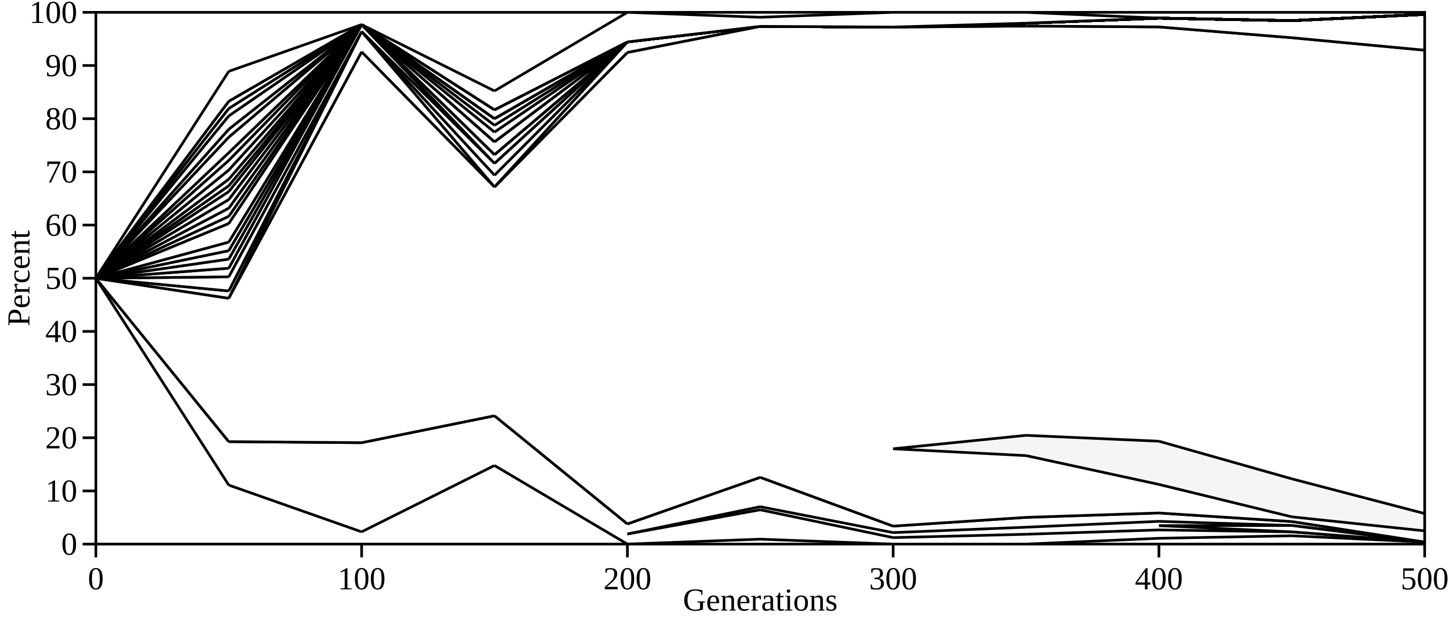

100
90
80
70
60
50
40
30
20
10
0
Percent
0
100
200
300
400
500
Generations

0.2.2 (malK, opgH)

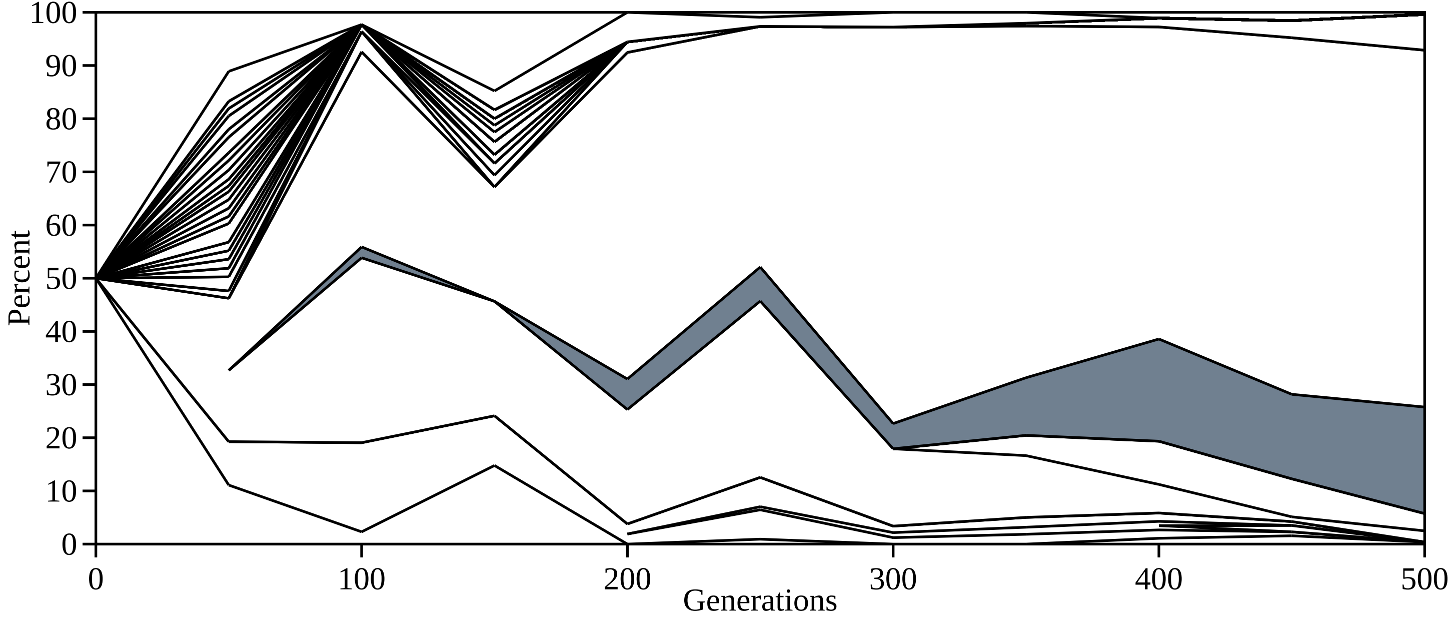

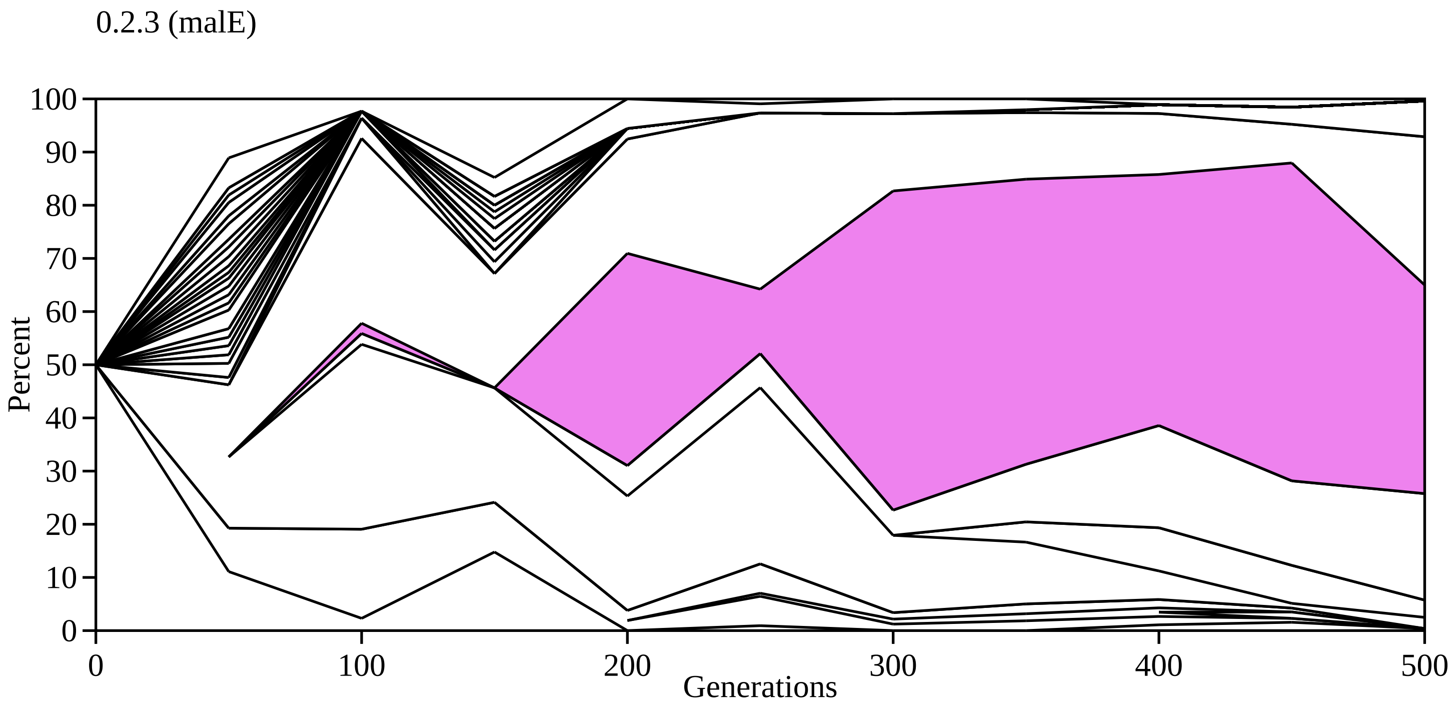

0.2.3 (malE)
100
90
80
70
60
50
40
30
20
10
0
Percent
0
100
200
300
400
500
Generations

0.2.4 (lptG)

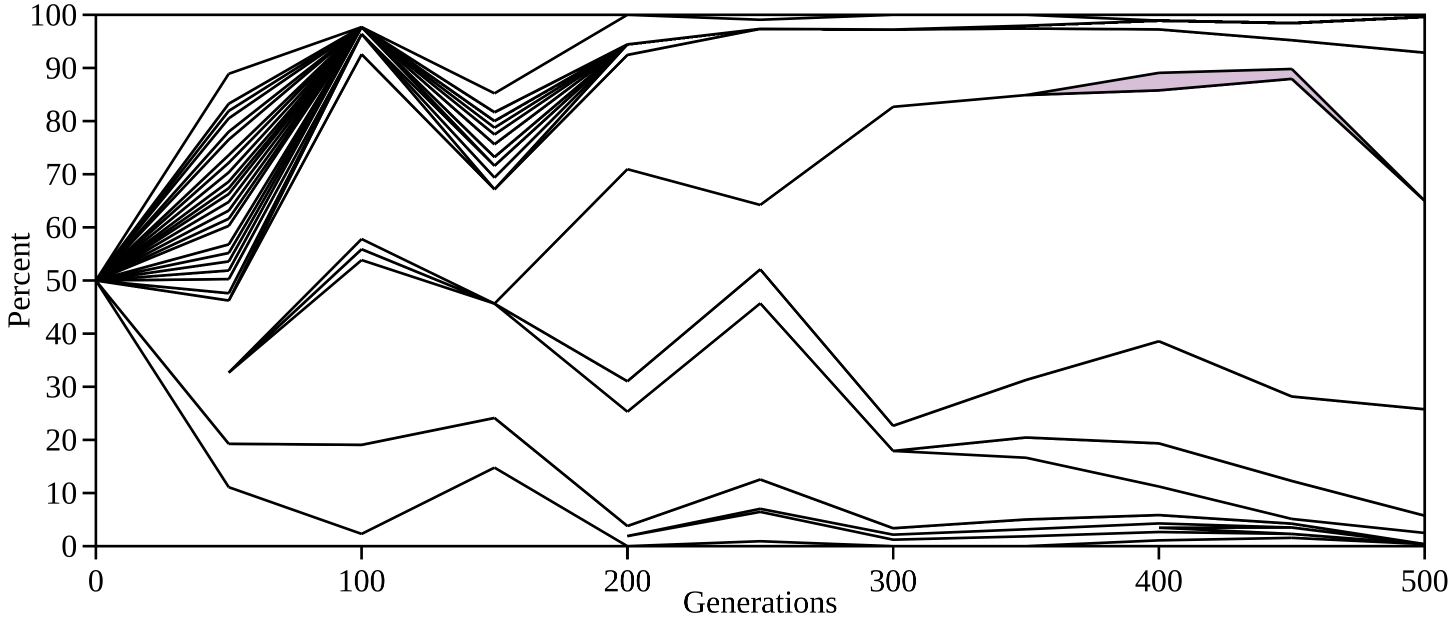
100
90
80
70
60
50
40
30
20
10
0
Percent
0
100
200
300
400
500
Generations

0.2.5 (fliG, lpxD, opgH, upstream dnaG, wzzE, glpR, rbsB, hfq)

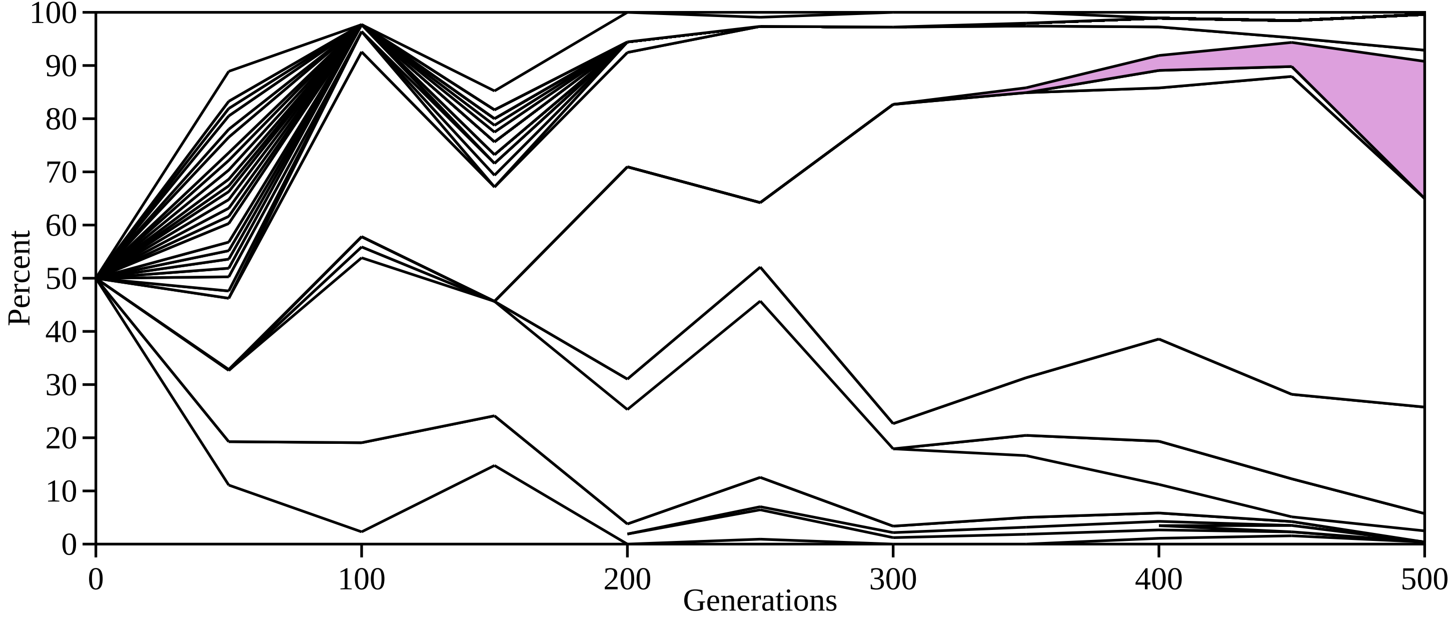

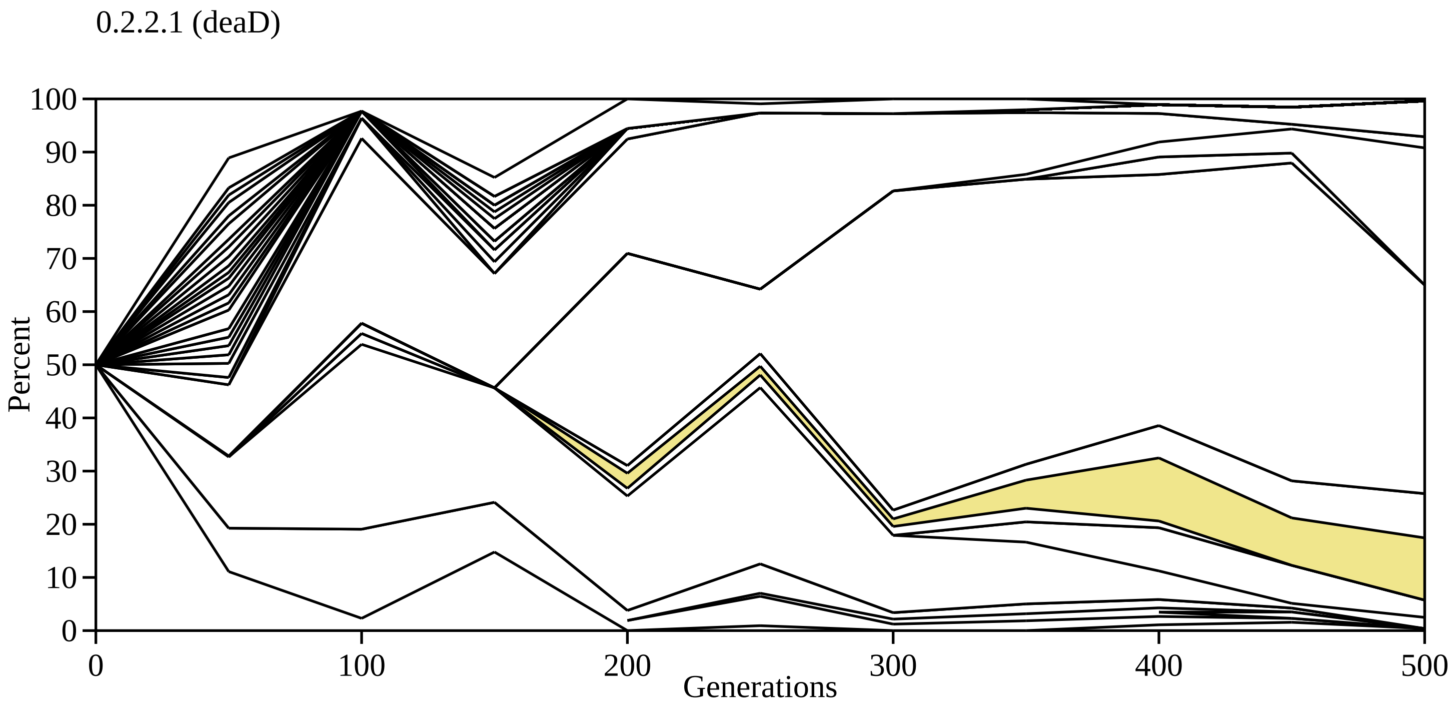
0.2.2.1 (deaD)
Percent
100
90
80
70
60
50
40
30
20
10
0
0
100
200
300
400
500
Generations

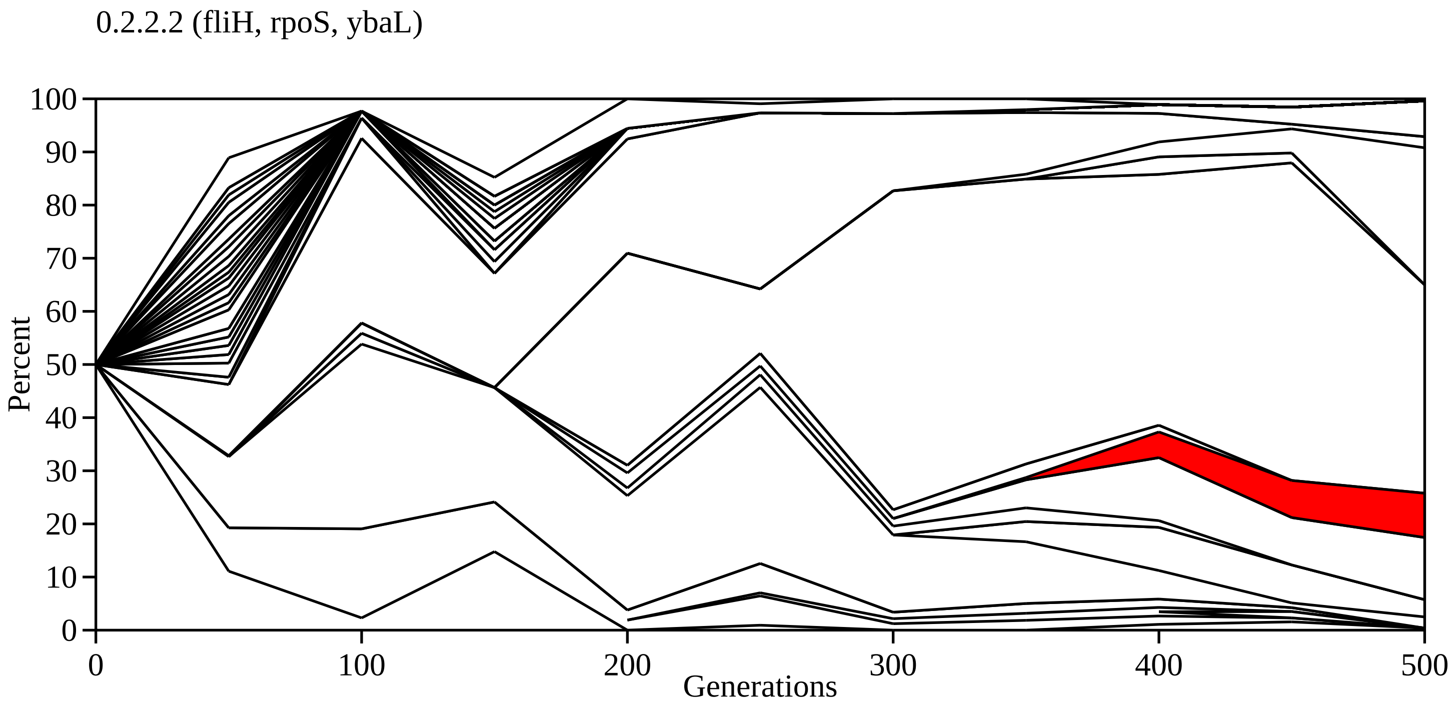
0.2.2.2 (fliH, rpoS, ybaL)
Percent
Generations
0
10
20
30
40
50
60
70
80
90
100
0
100
200
300
400
500

0.2.3.1 (hfq, opgH)

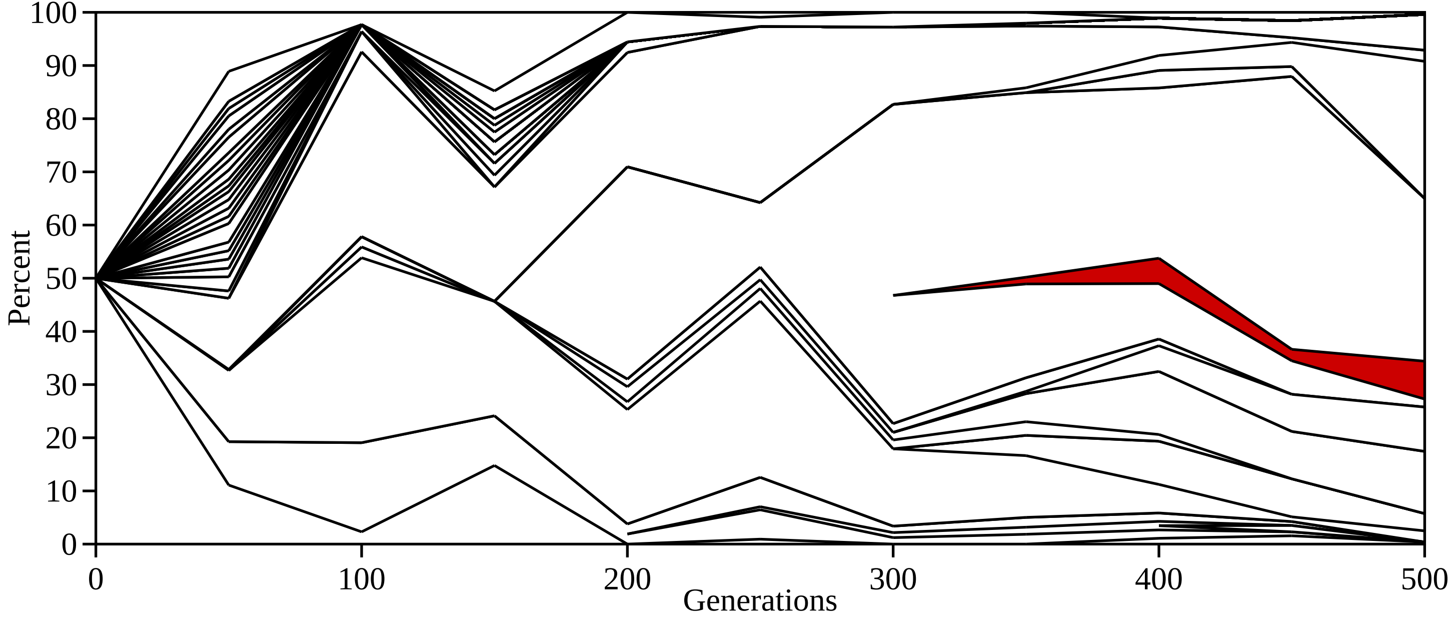

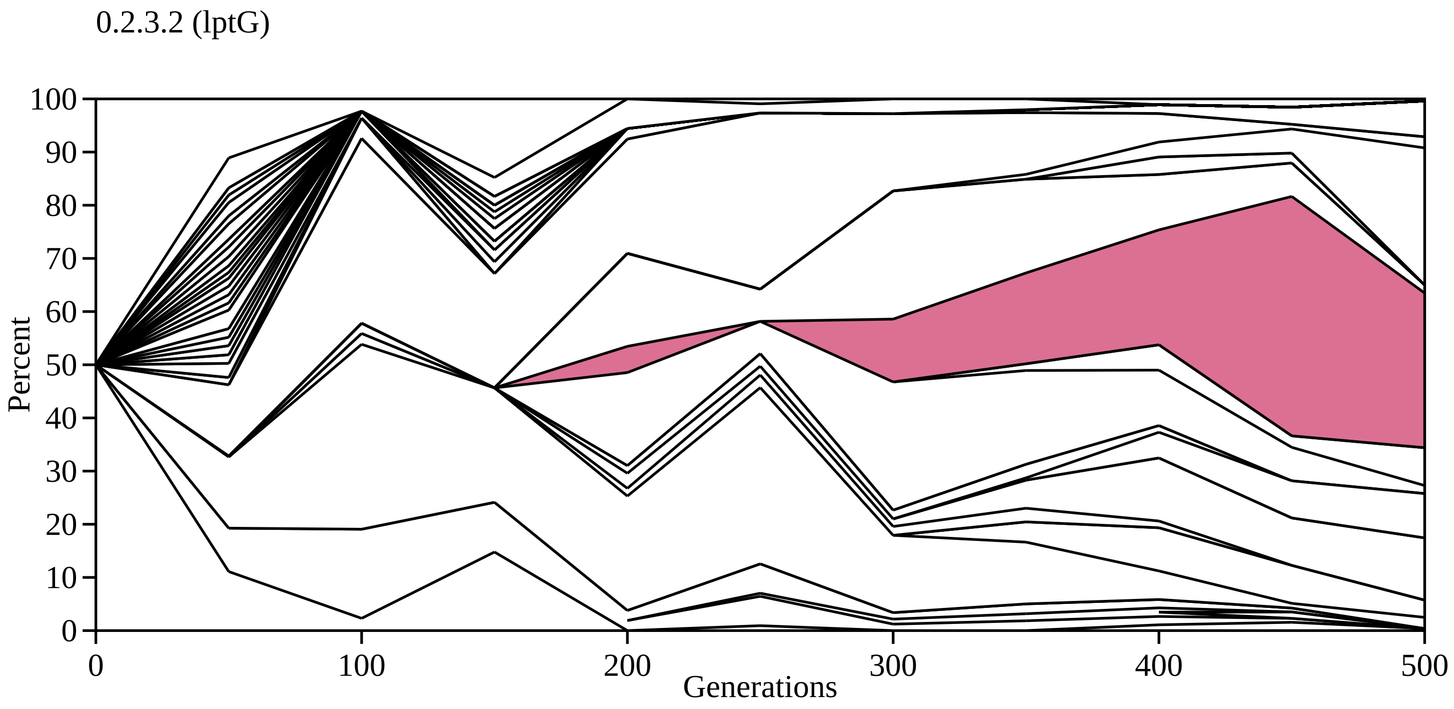
0.2.3.2 (lptG)
Percent
Generations
0
10
20
30
40
50
60
70
80
90
100
0
100
200
300
400
500

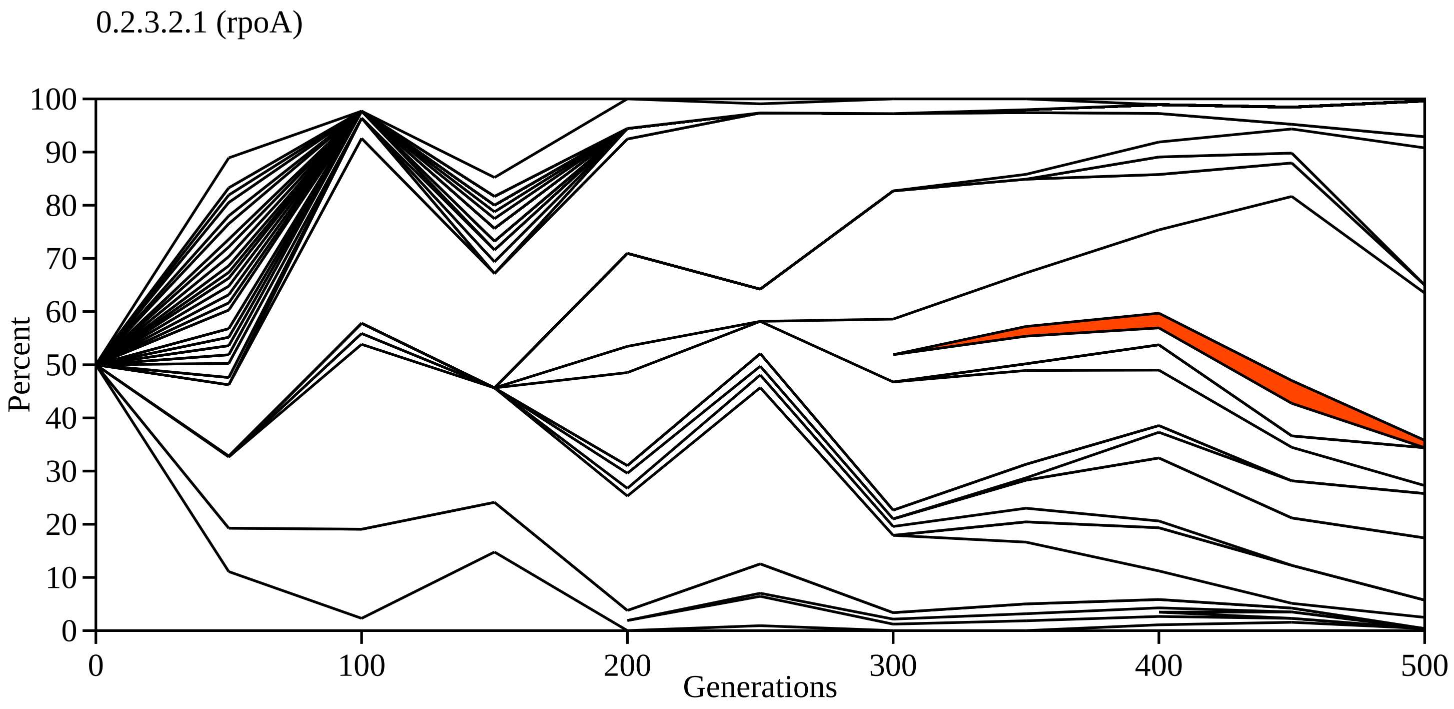

0.2.3.2.1 (rpoA)
Percent
Generations
0
10
20
30
40
50
60
70
80
90
100
0
100
200
300
400
500

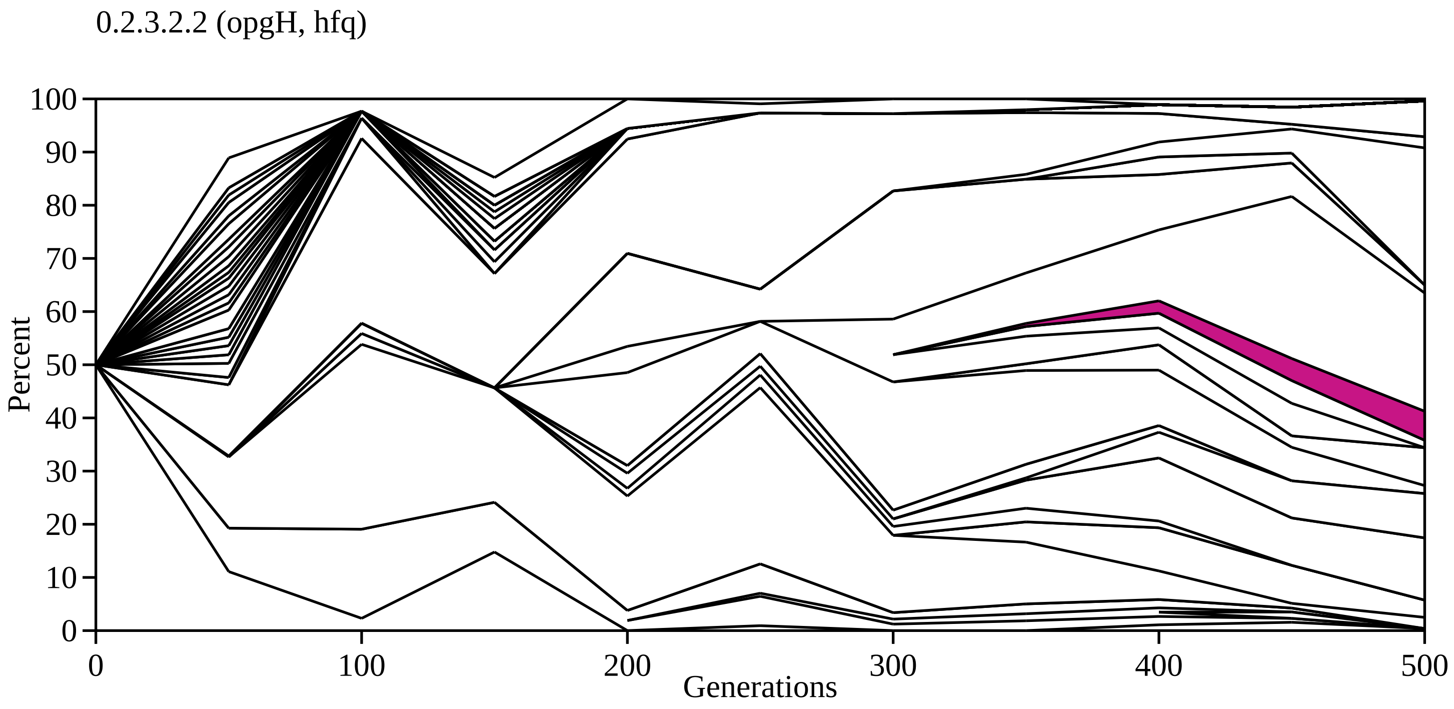
0.2.3.2.2 (opgH, hfq)
100
90
80
70
60
50
40
30
20
10
0
Percent
0
100
200
300
400
500
Generations

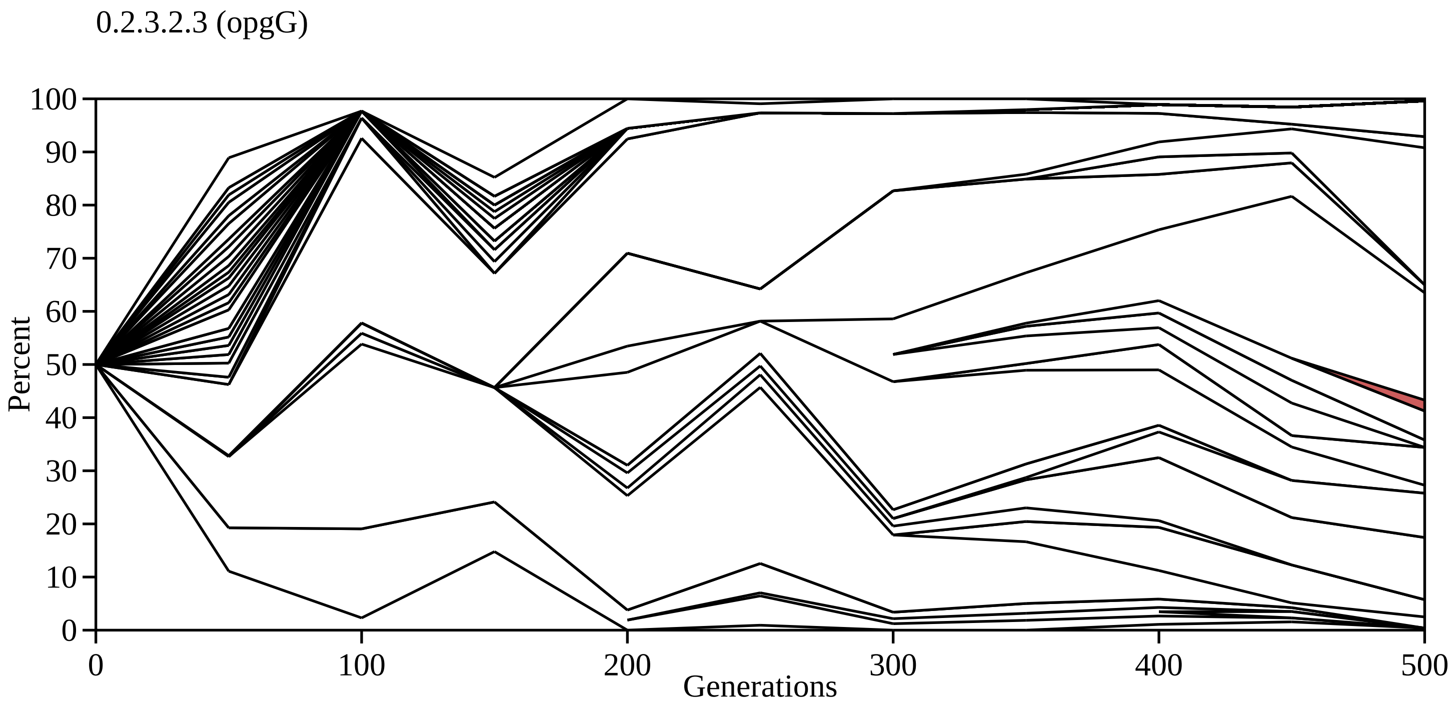
0.2.3.2.3 (opgG)
Percent
100
90
80
70
60
50
40
30
20
10
0
0
100
200
300
400
500
Generations

0.2.3.2.4 (rbsB, opgH)

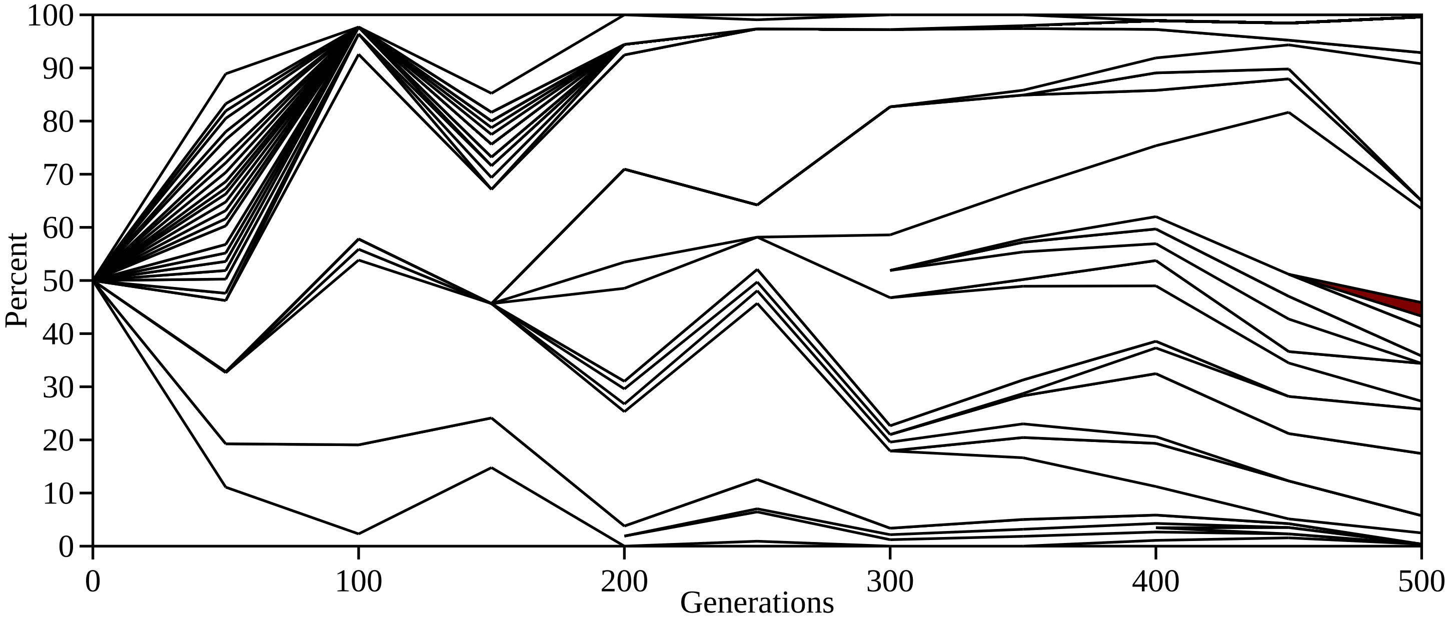

0.2.3.2.5 (pgsA)

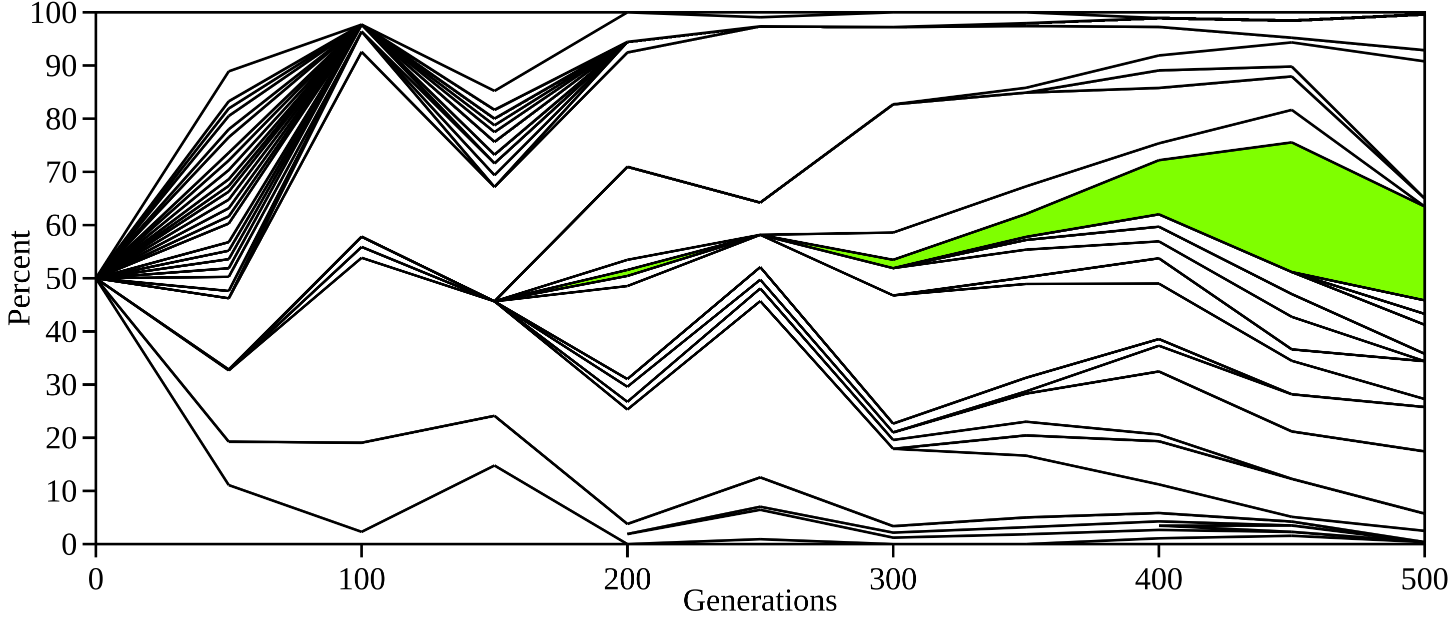

100
90
80
70
60
50
40
30
20
10
0
Percent
0
100
200
300
400
500
Generations

0.2.3.2.1.1 (rpoS)

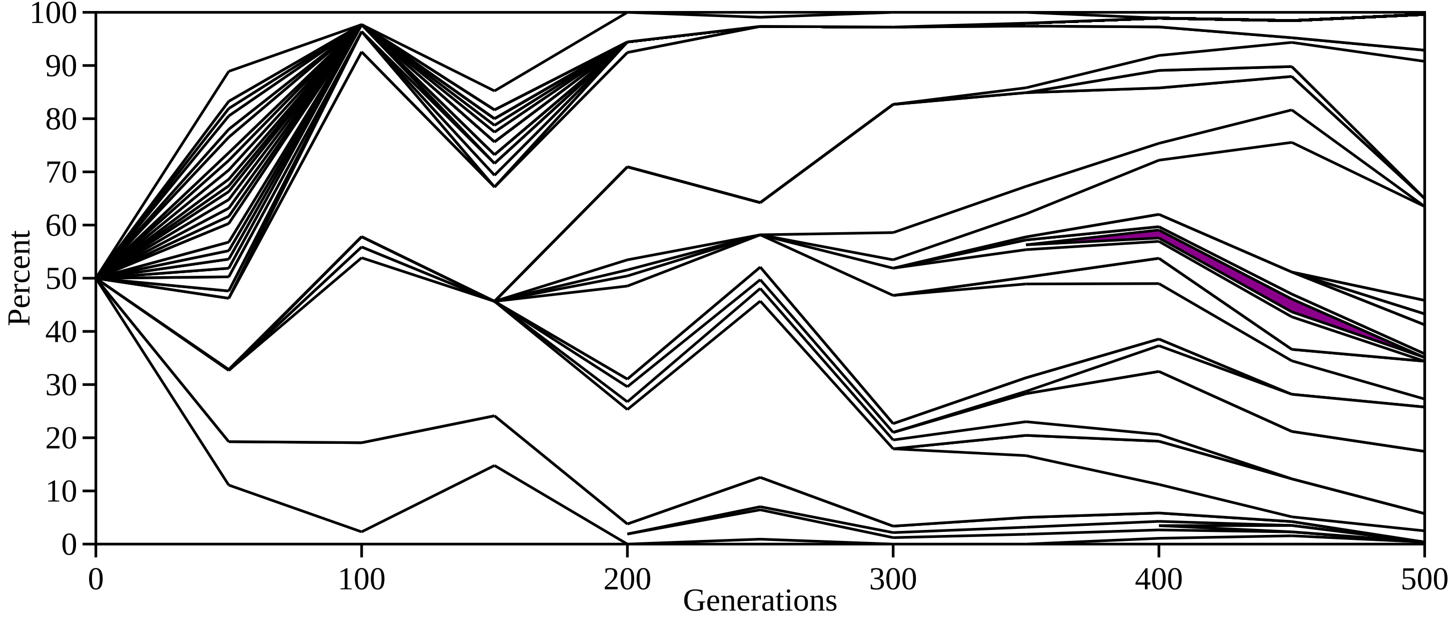
Percent
100
90
80
70
60
50
40
30
20
10
0
0
100
200
300
400
500
Generations

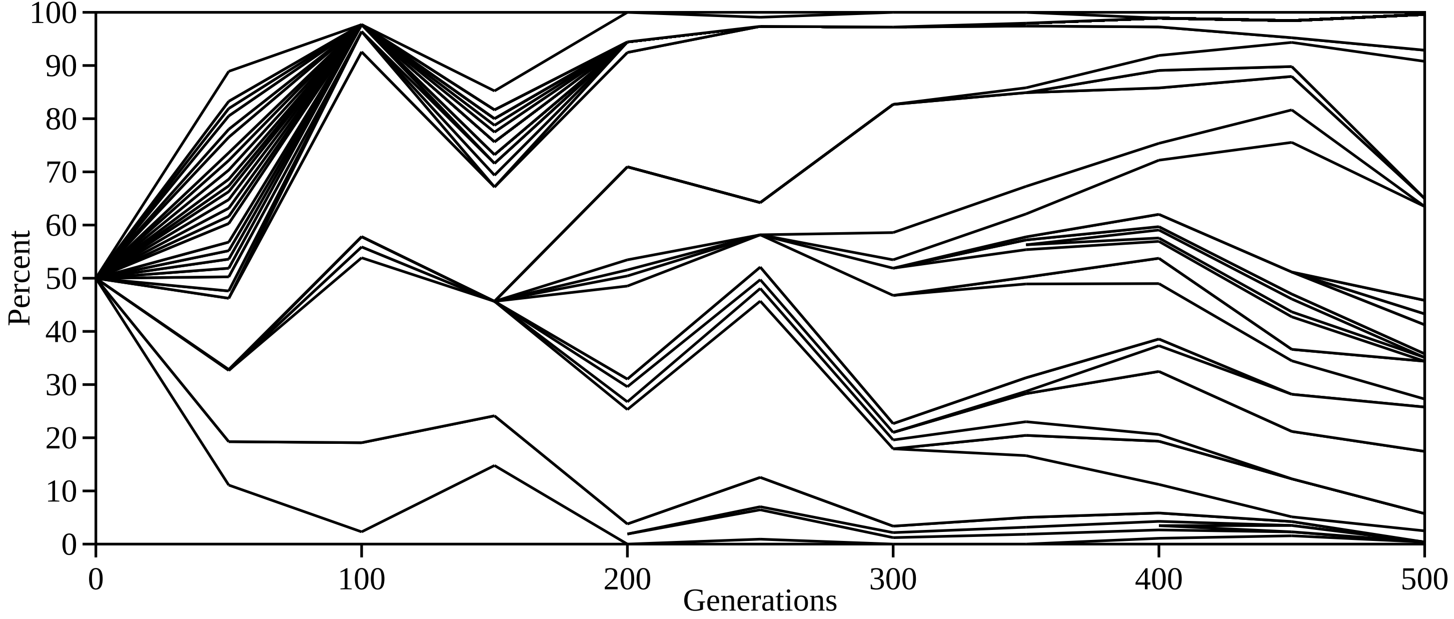

100
90
80
70
60
50
40
30
20
10
0
Percent
0
100
200
300
400
500
Generations

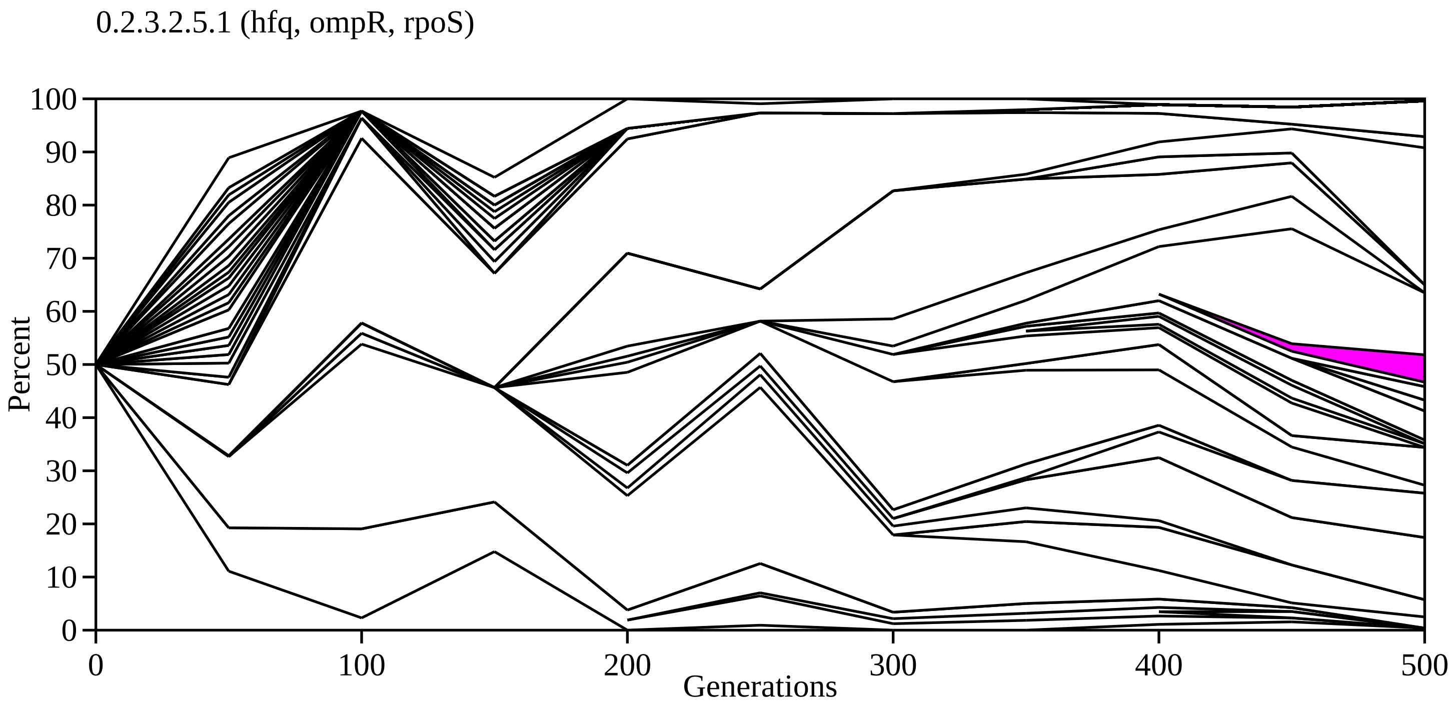

0.2.3.2.5.1 (hfq, ompR, rpoS)
Percent
Generations
0
10
20
30
40
50
60
70
80
90
100
0
100
200
300
400
500

0.2.3.2.5.2 (yiaO)

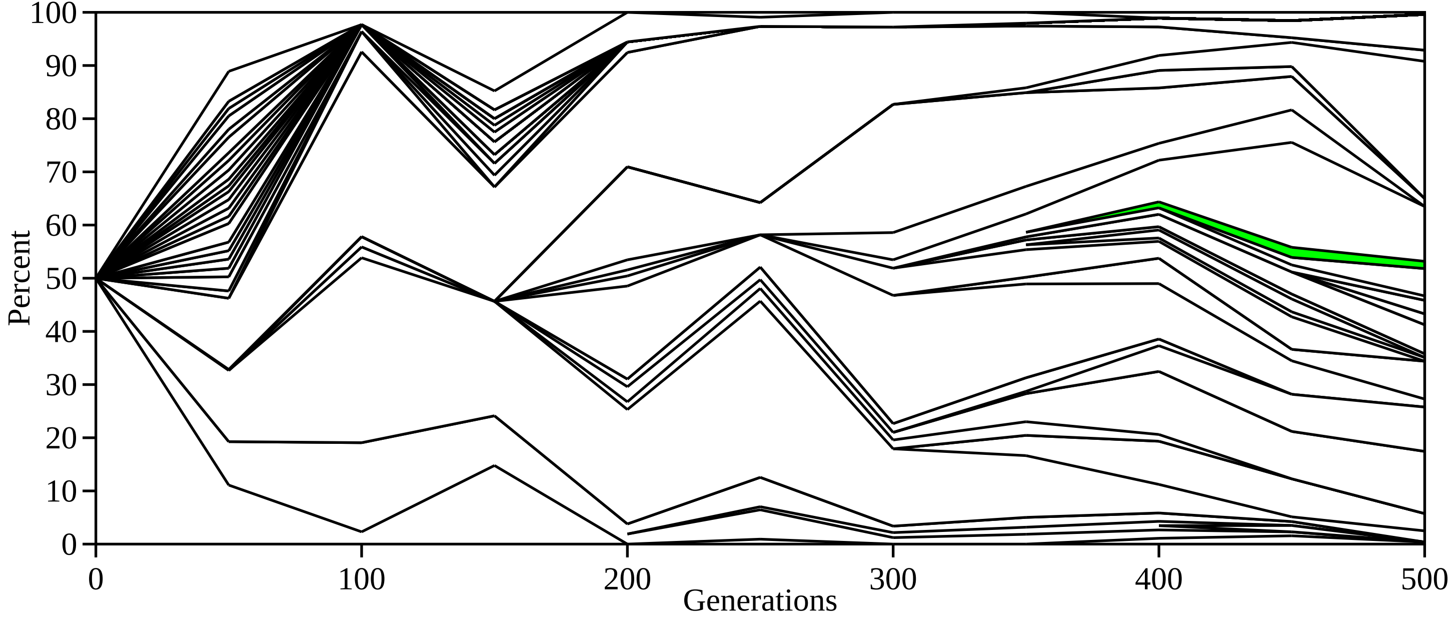

100
90
80
70
60
50
40
30
20
10
0
Percent
0
100
200
300
400
500
Generations

0.2.3.2.5.3 (proQ, malT)

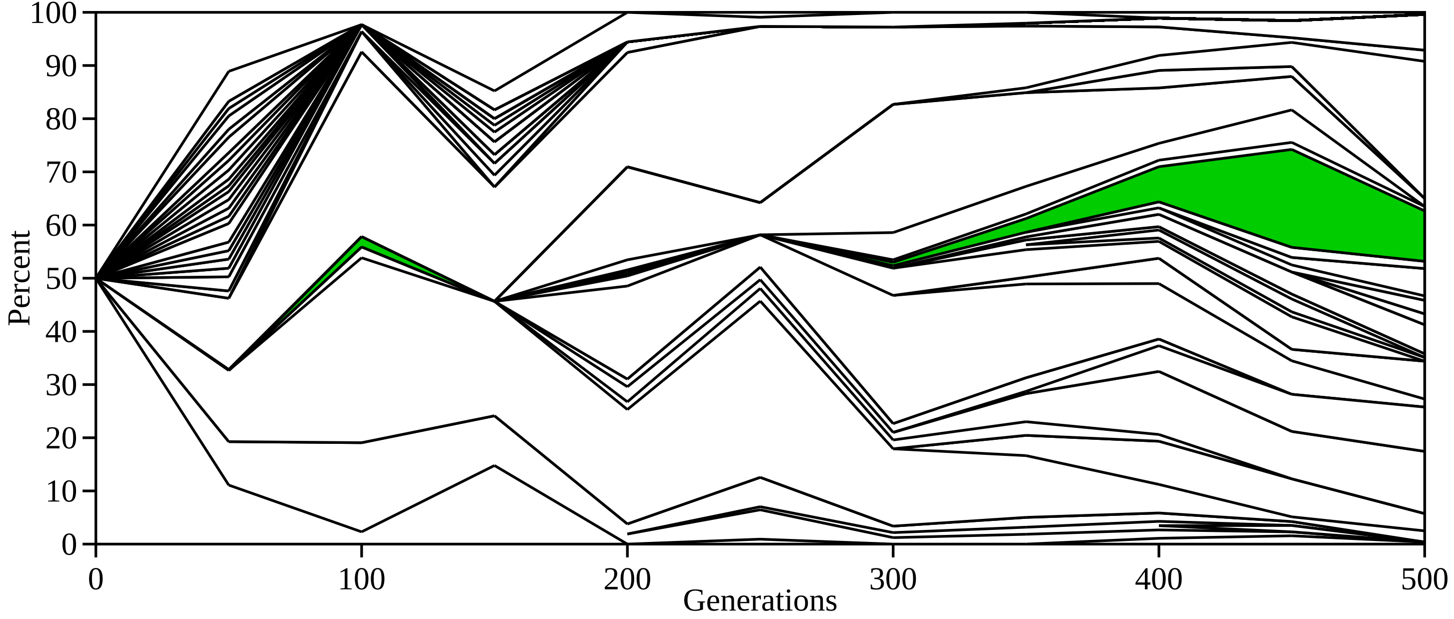

0.2.3.2.5.3.1 (opgH)

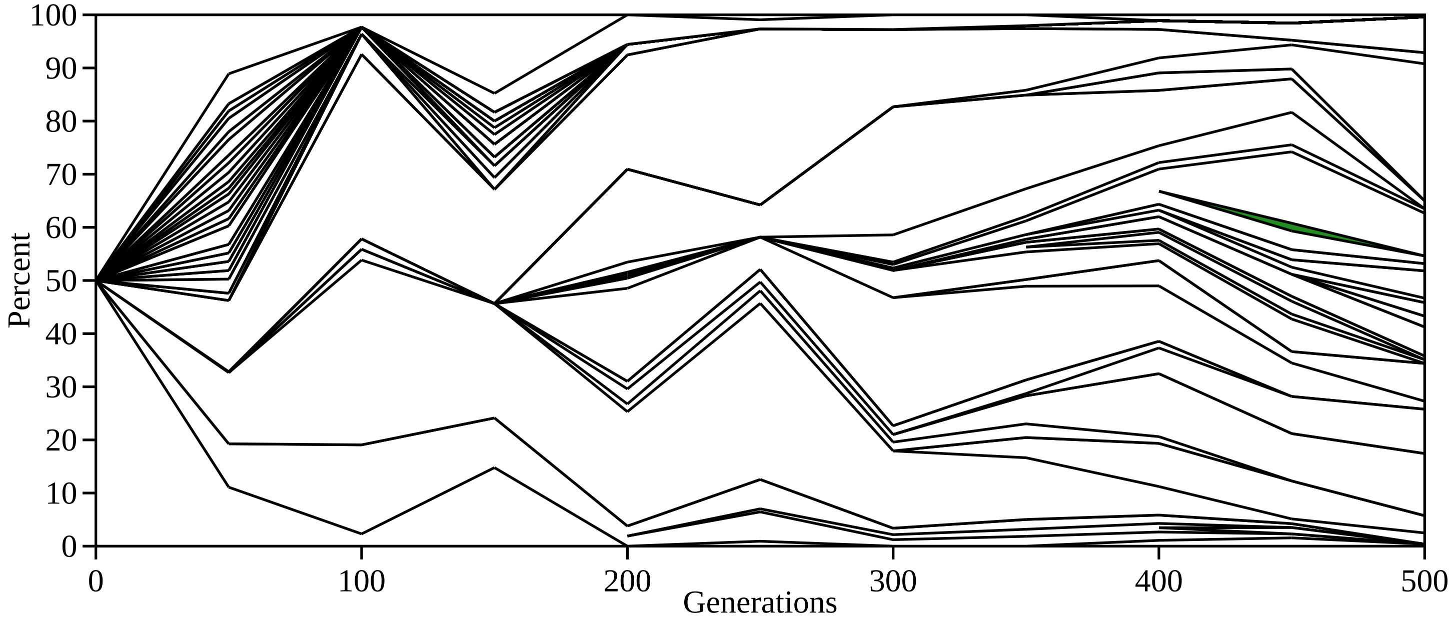

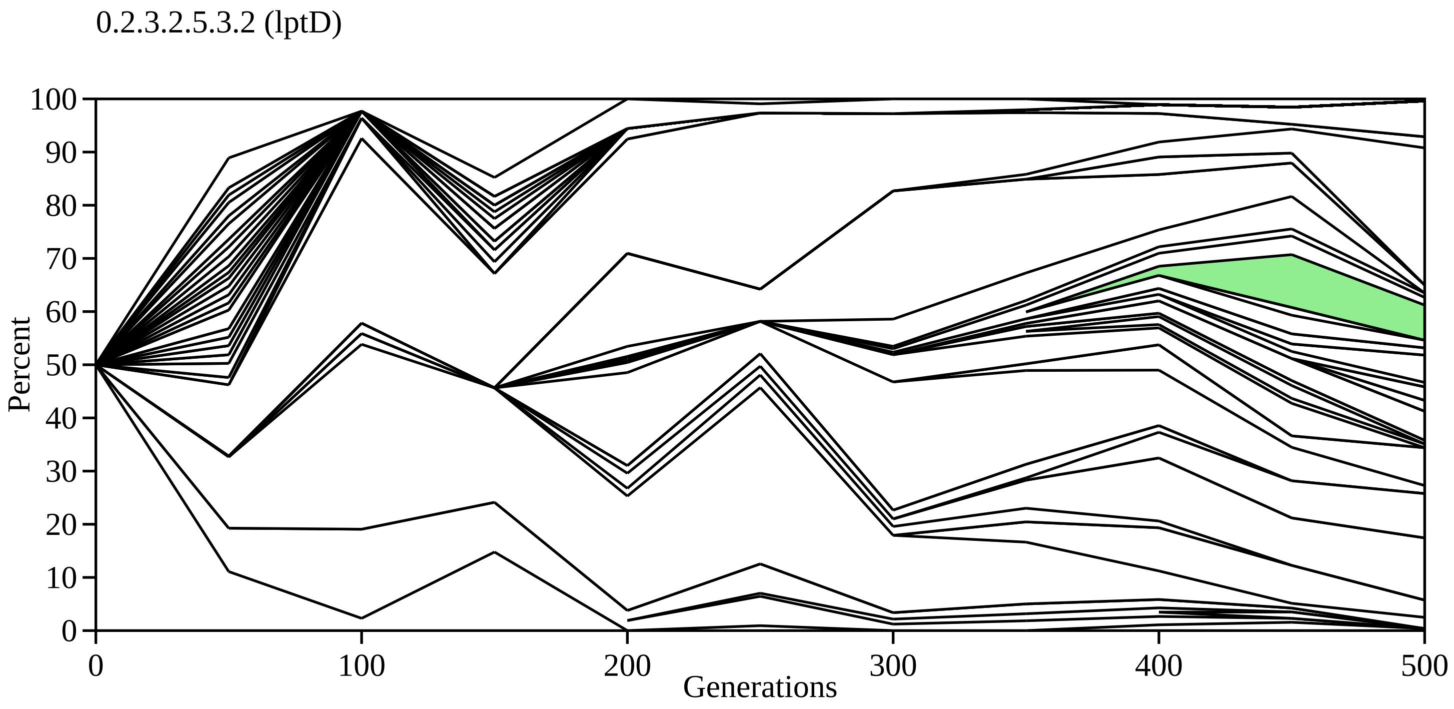
0.2.3.2.5.3.2 (lptD)
Percent
Generations
100
90
80
70
60
50
40
30
20
10
0
0
100
200
300
400
500

0.2.3.2.5.3.2.1 (yiaO)

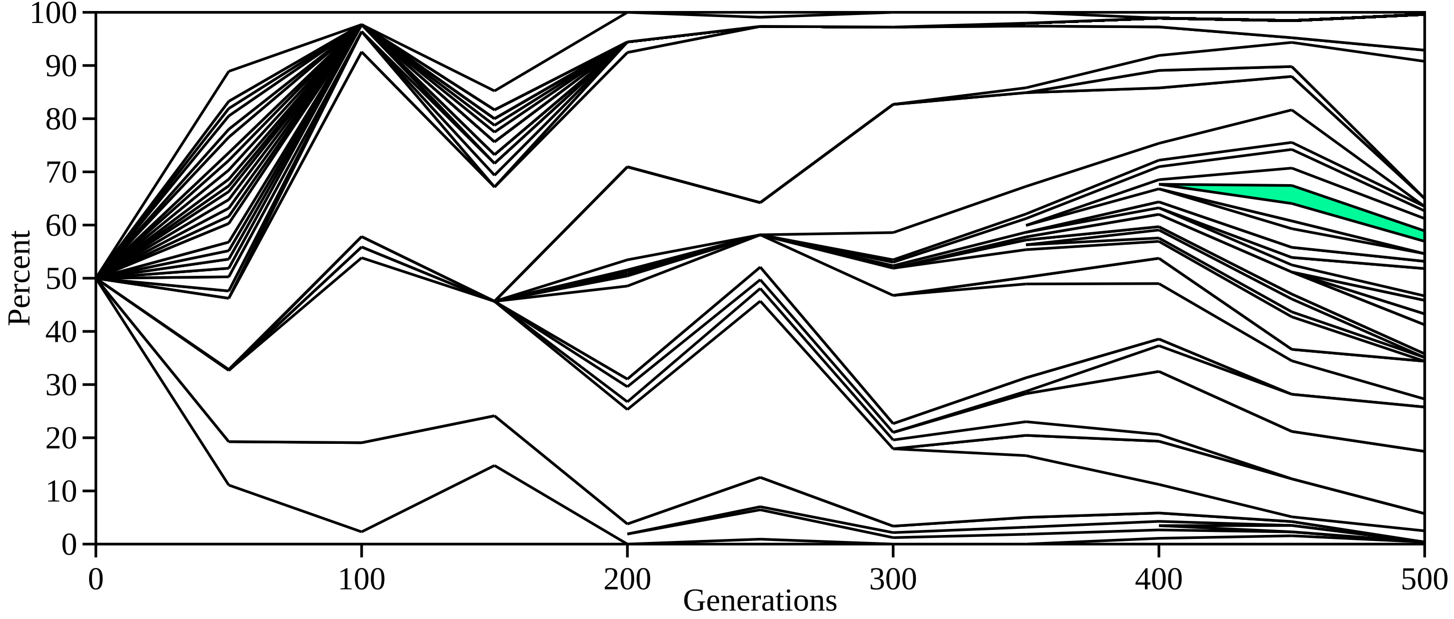

0.2.5.1 (upstream adhE)

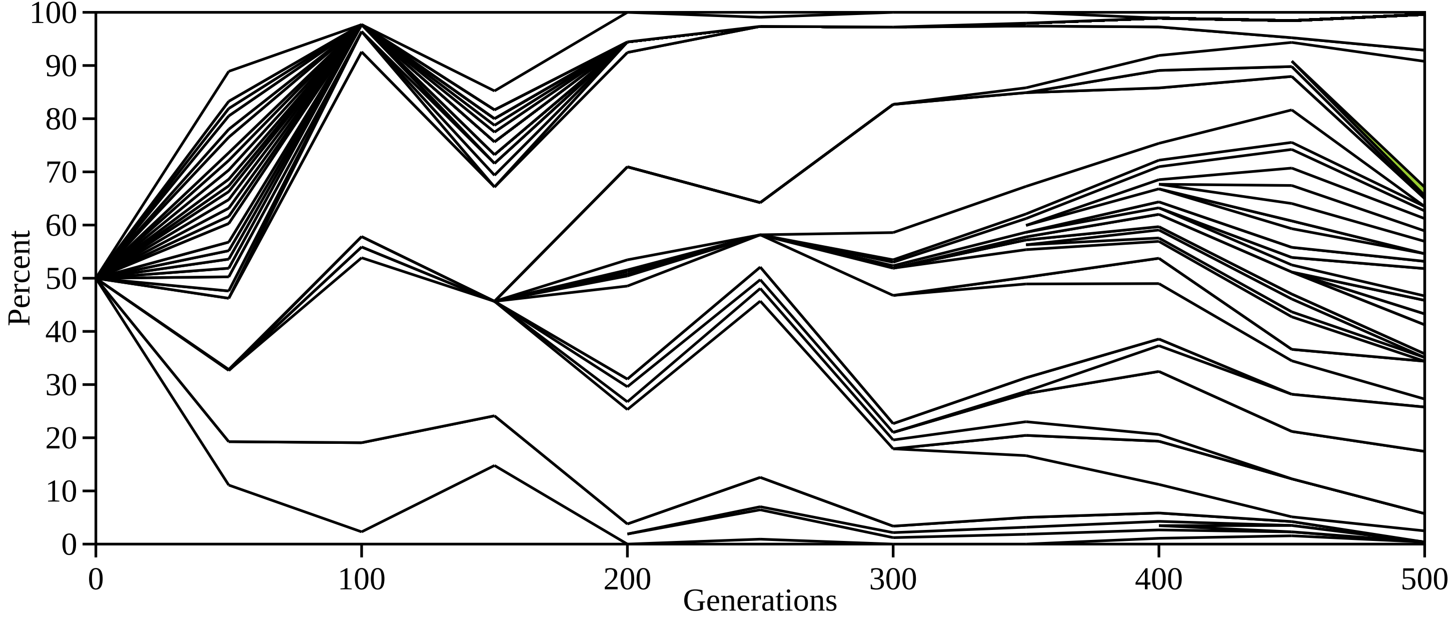

0.2.5.2 (gatZ, downstream fis)

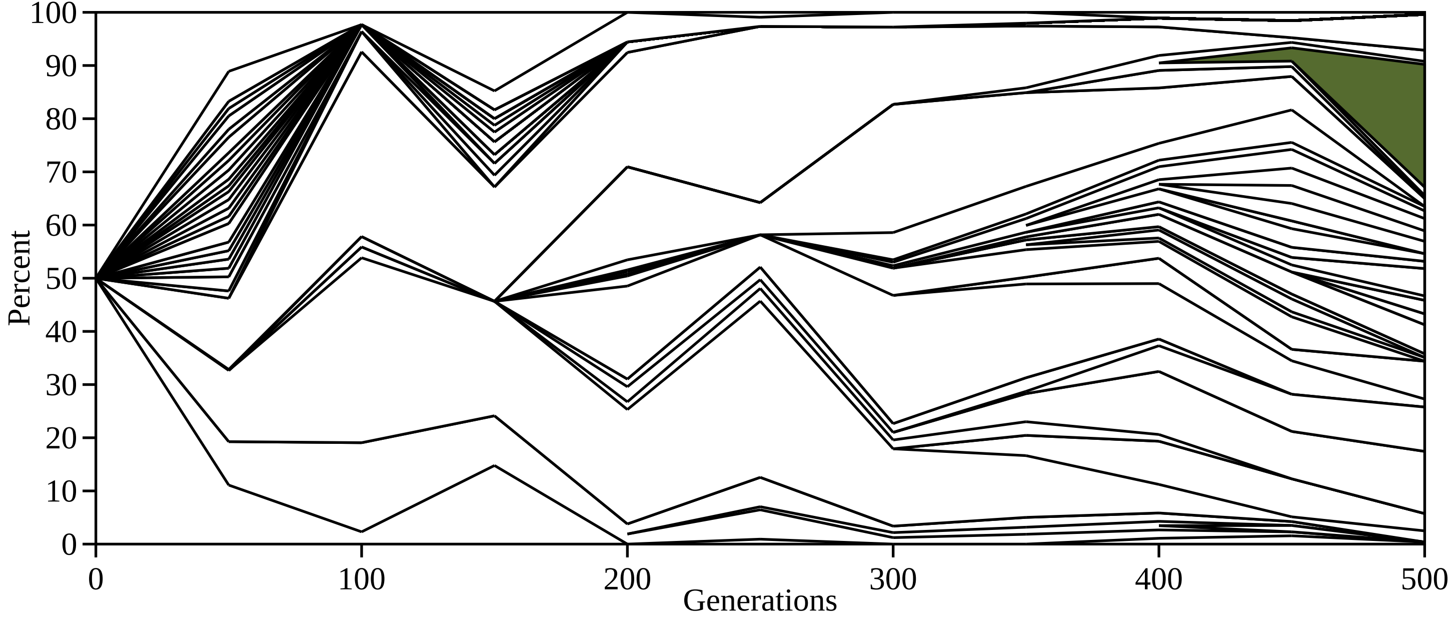

0.2.5.2.1 (pgi)

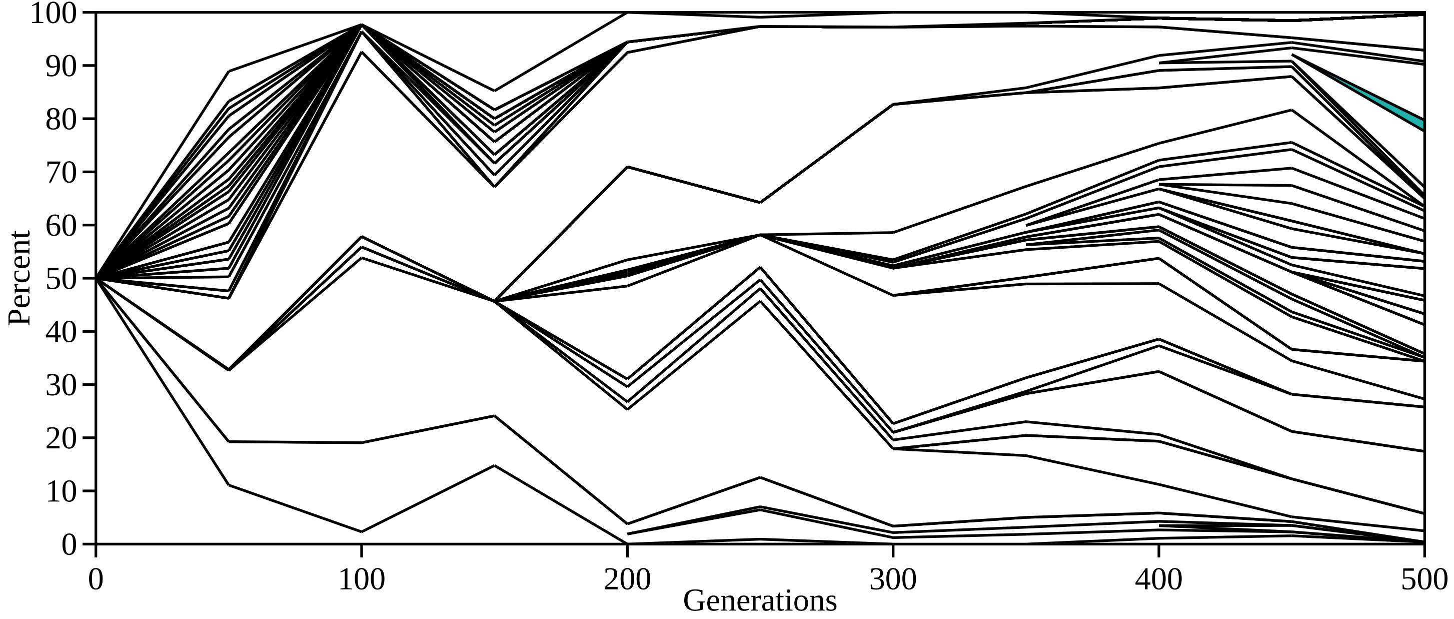
100
90
80
70
60
50
40
30
20
10
0
Percent
0
100
200
300
400
500
Generations

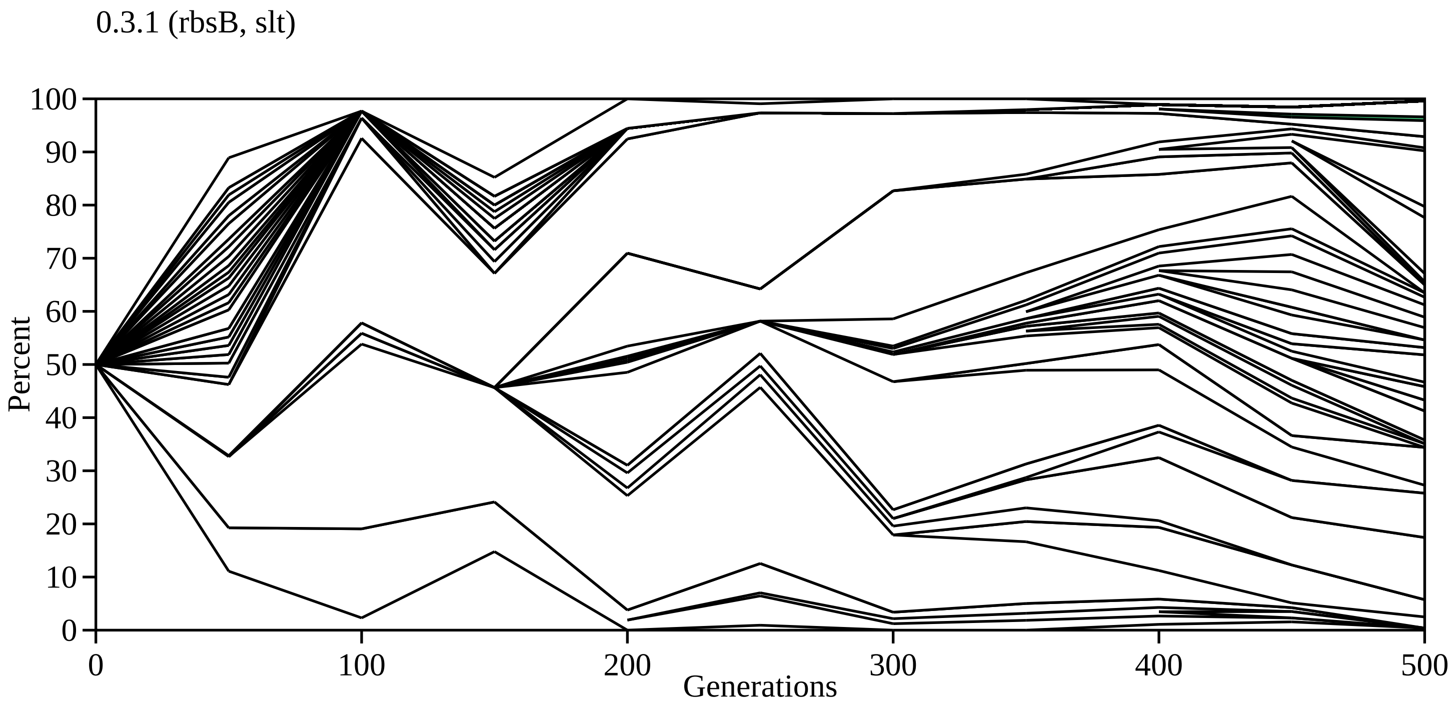
0.3.1 (rbsB, slt)
Percent
100
90
80
70
60
50
40
30
20
10
0
0
100
200
300
400
500
Generations
