# Supplementary material for: Evolutionary dynamics and structural consequences of de novo beneficial mutations and mutant lineages arising in a constant environment
Source: BMC Biol. 2021 Feb 4;19:20. doi: 10.1186/s12915-021-00954-0 (PMC7863352; doi:10.1186/s12915-021-00954-0)
Supplement: Supplementary file 7 — Additional file 7 Fig. S11. Muller diagrams for novel alleles arising in chemostat 3, showing details for each lineage. [file 12915_2021_954_MOESM7_ESM.pdf]

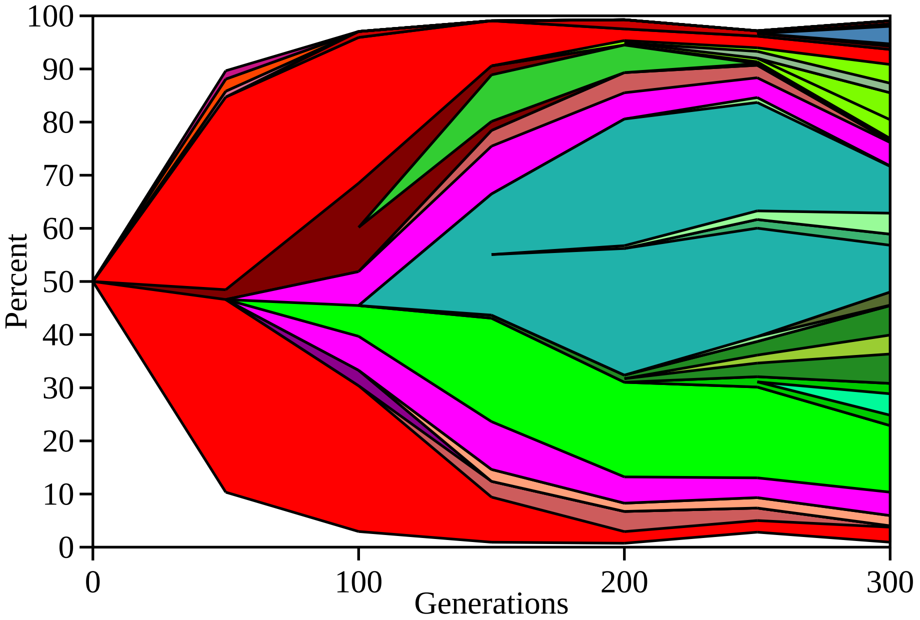

Percent
100
90
80
70
60
50
40
30
20
10
0
0
100
200
300
Generations

Lineages for fimH

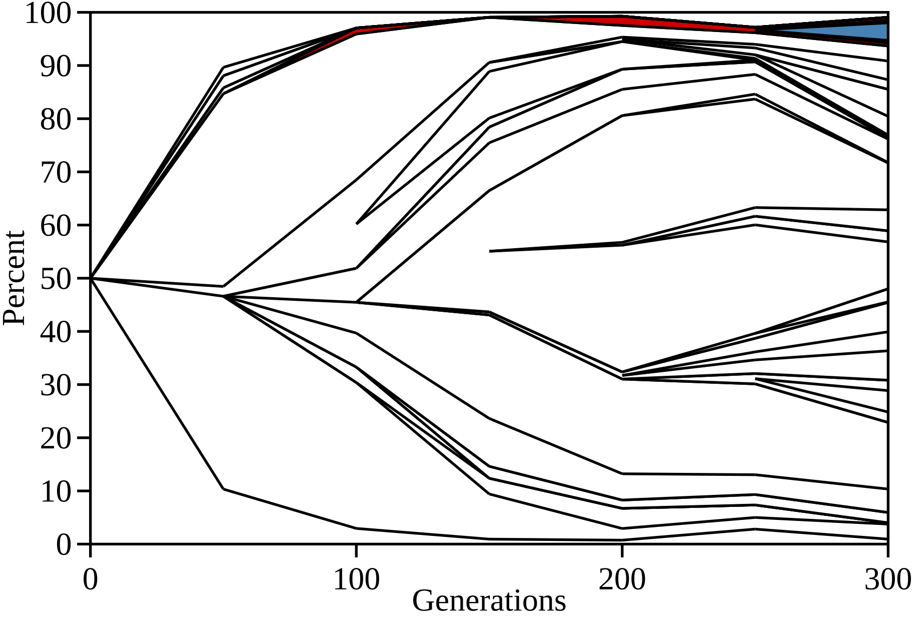

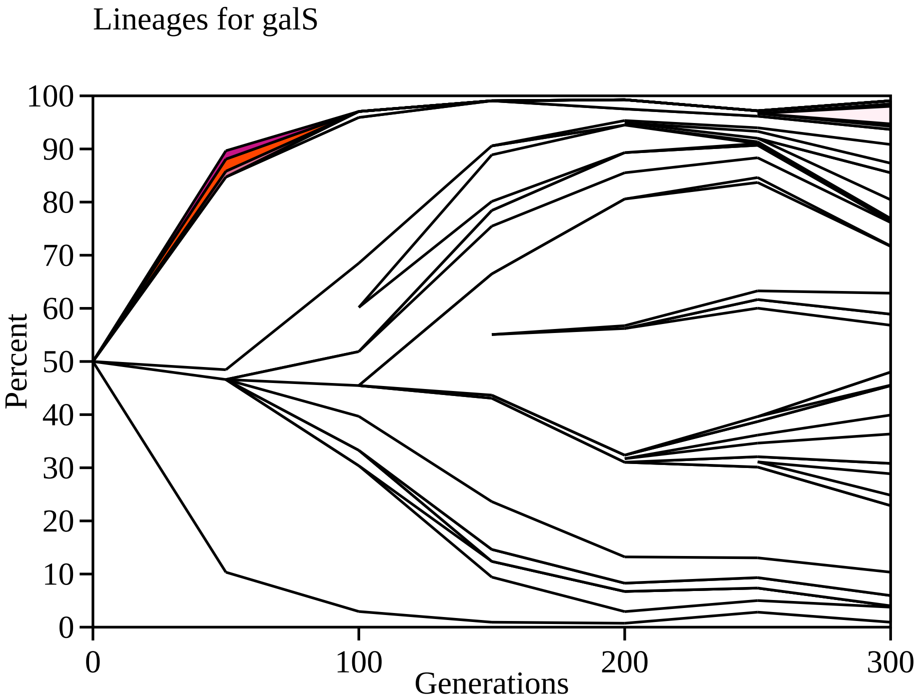
Lineages for galS
Percent
0
10
20
30
40
50
60
70
80
90
100
0
100
200
300
Generations

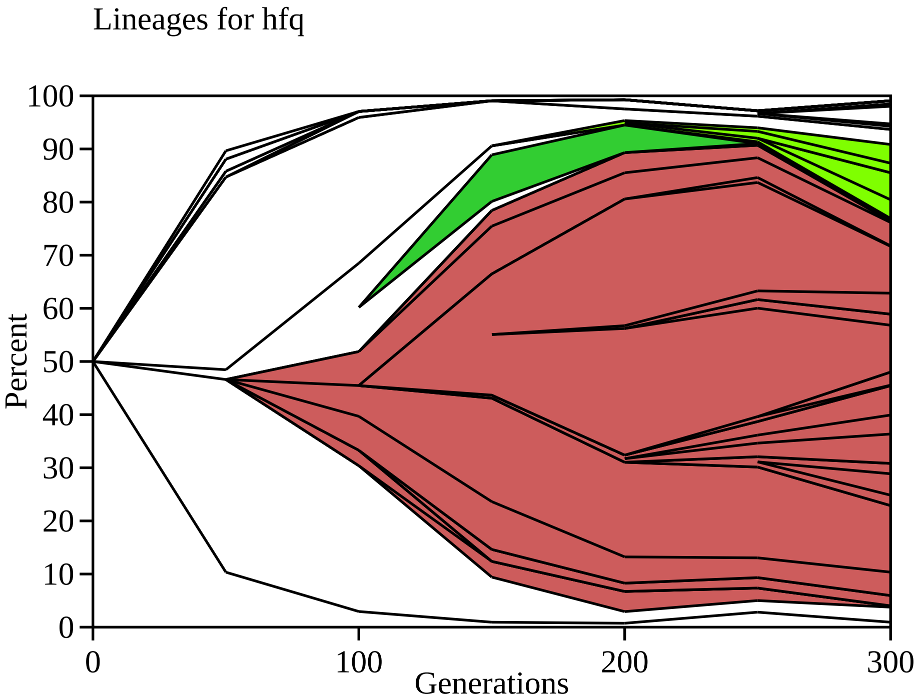

Lineages for hfq
Percent
100
90
80
70
60
50
40
30
20
10
0
0
100
200
300
Generations

Lineages for lptD

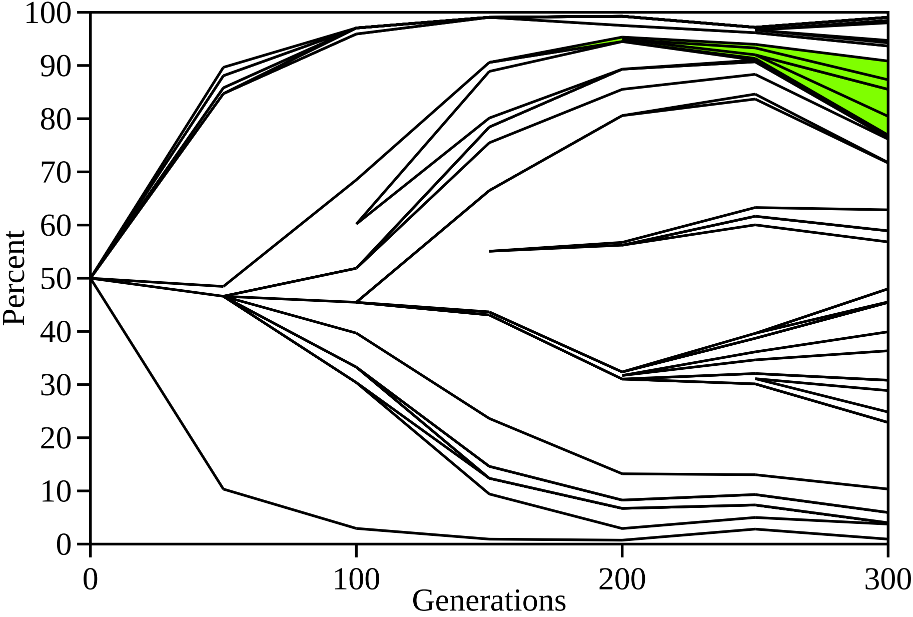

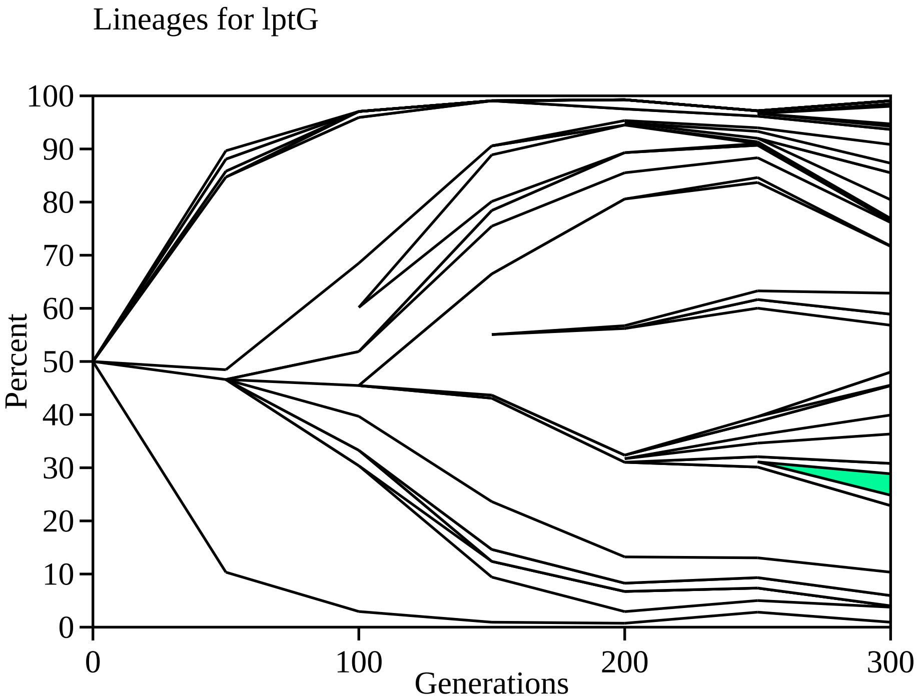

Lineages for lptG
Percent
100
90
80
70
60
50
40
30
20
10
0
0
100
200
300
Generations

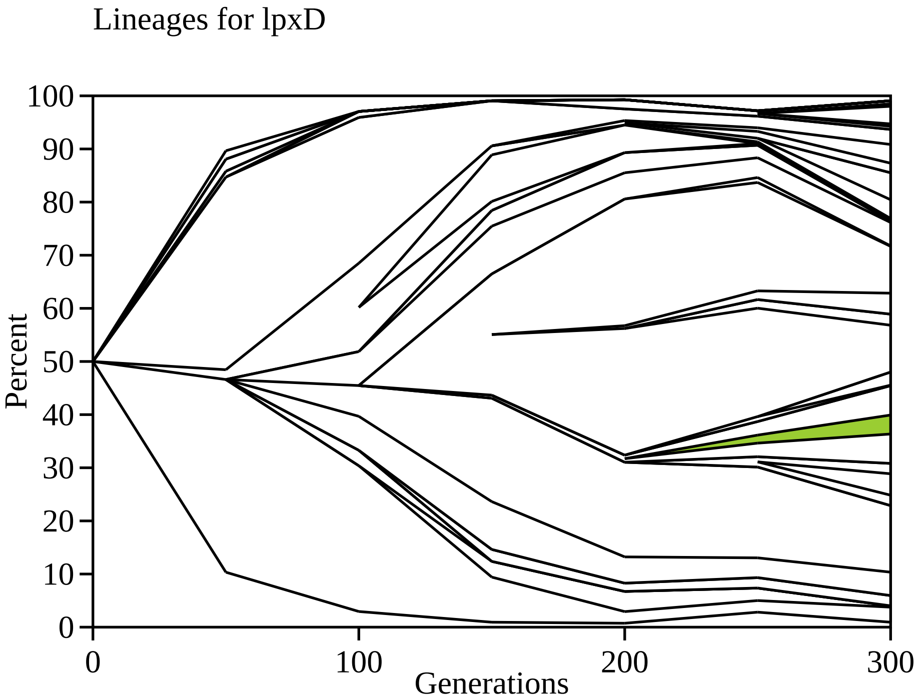
Lineages for lpxD
Percent
100
90
80
70
60
50
40
30
20
10
0
0
100
200
300
Generations

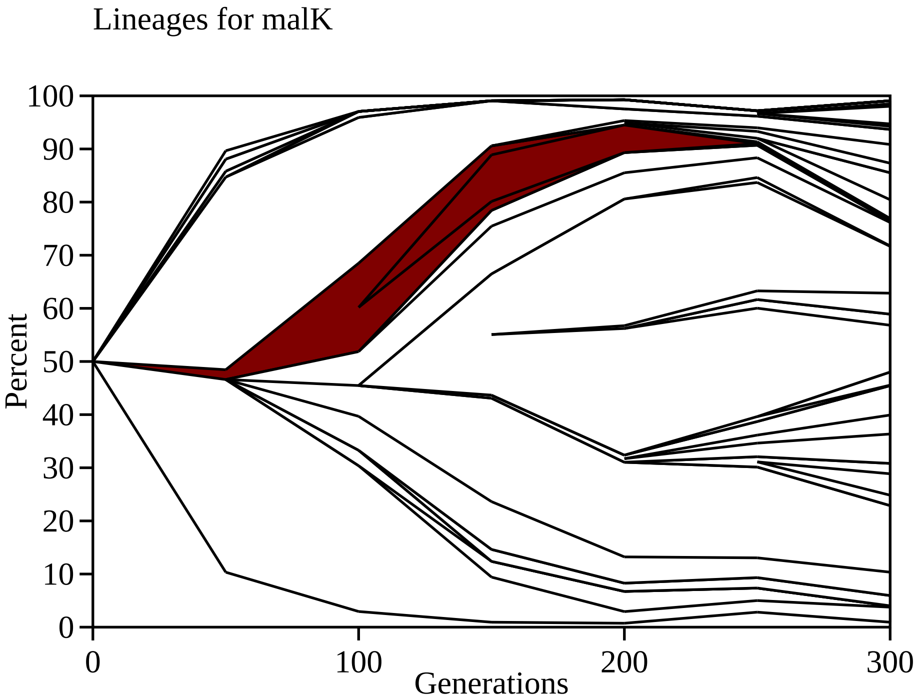

Lineages for malK
100
90
80
70
60
50
40
30
20
10
0
Percent
0
100
200
300
Generations

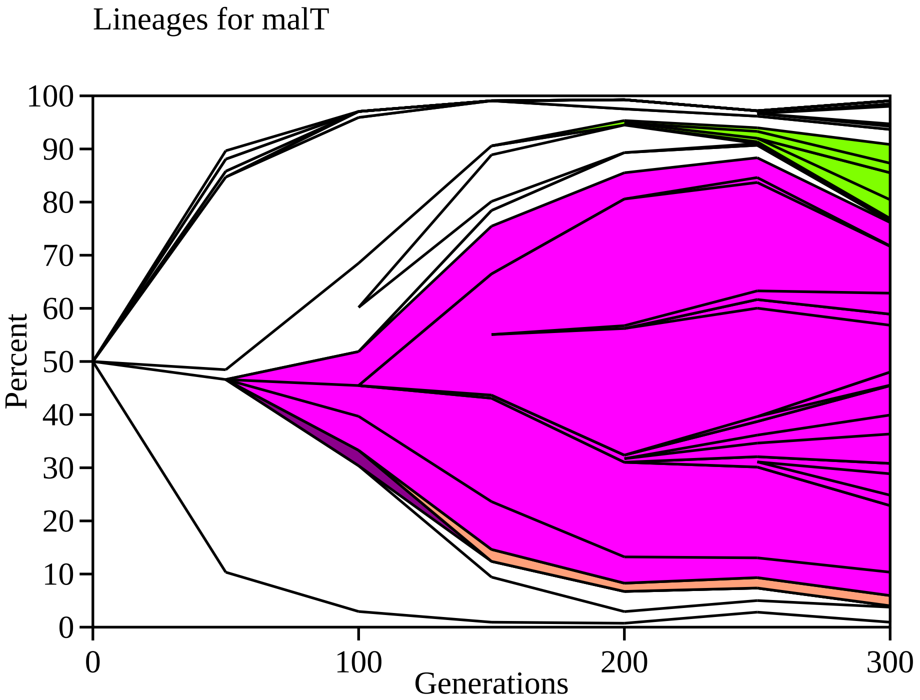
Lineages for malT
Percent
0
10
20
30
40
50
60
70
80
90
100
Generations
0
100
200
300

Lineages for opgG

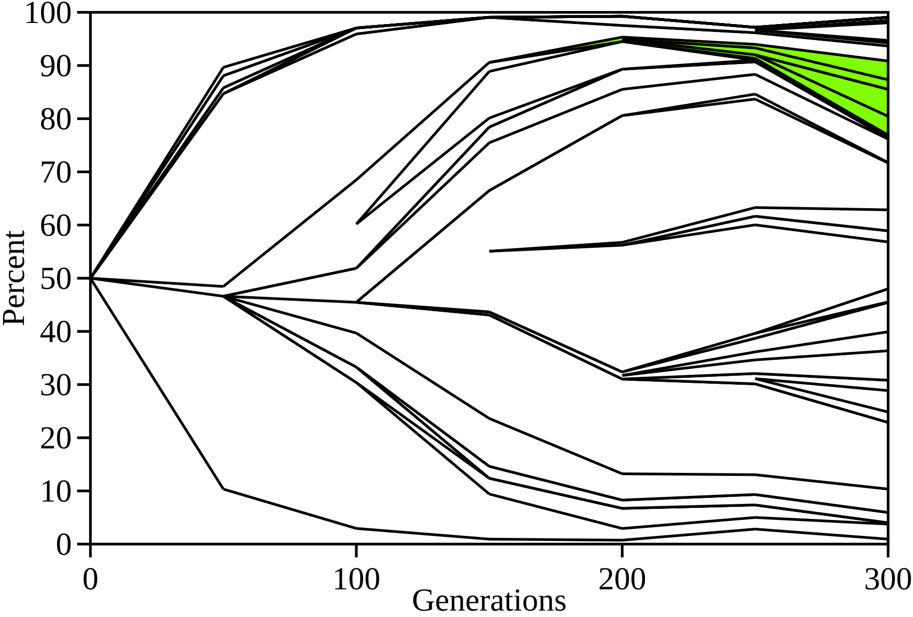

Lineages for opgH

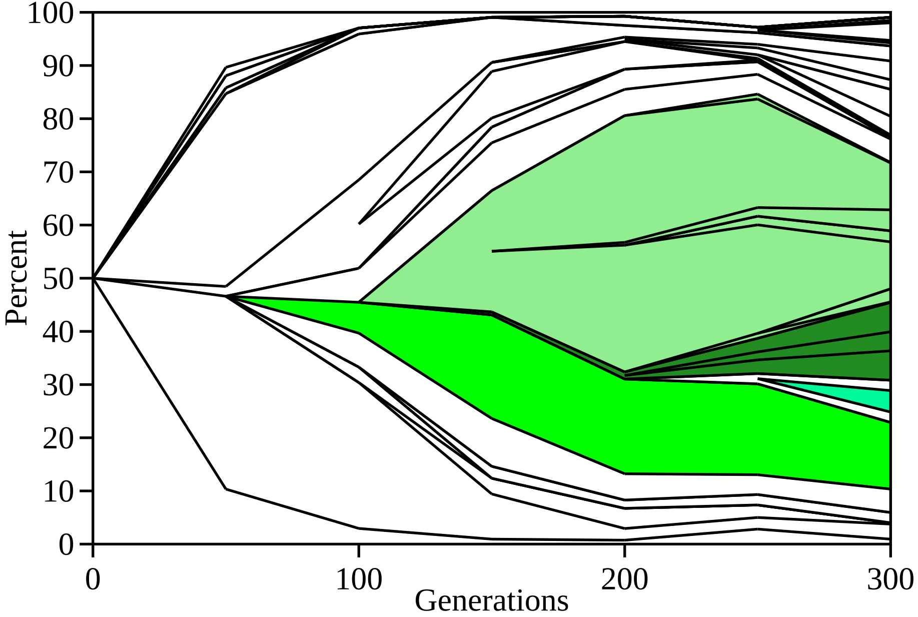

Lineages for pfkA

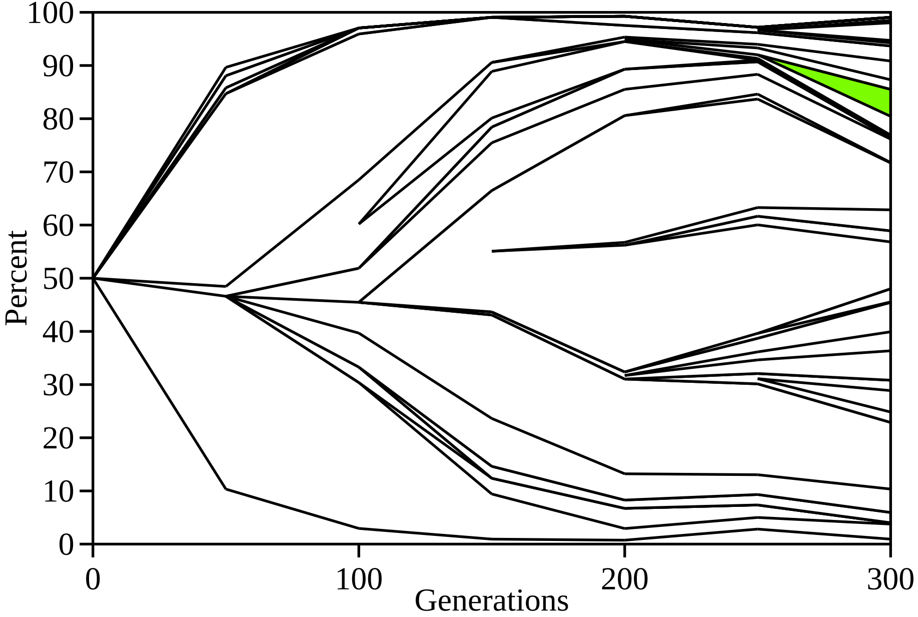

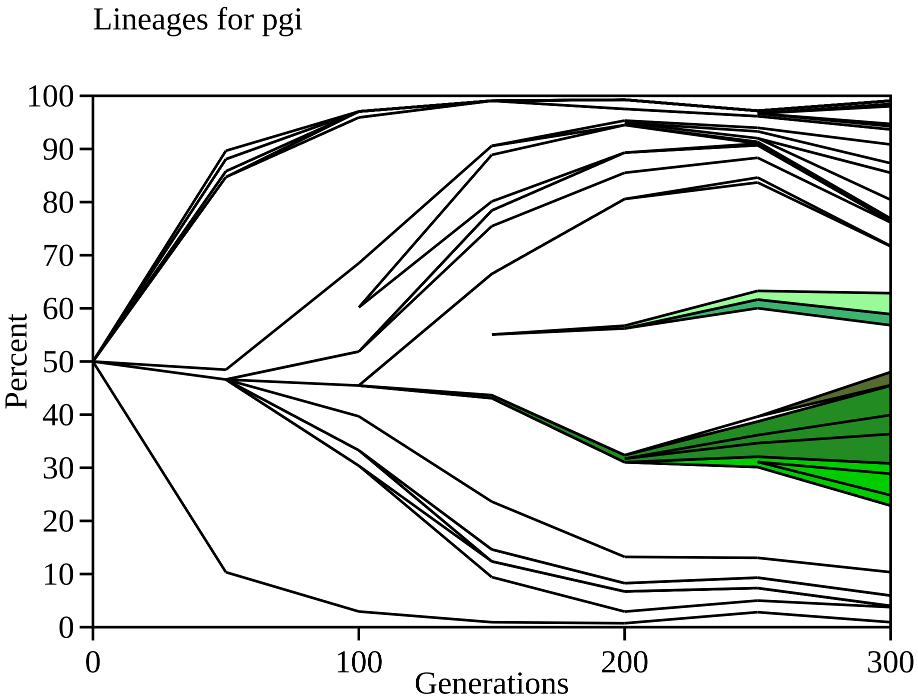
Lineages for pgi
Percent
Generations
0
10
20
30
40
50
60
70
80
90
100
0
100
200
300

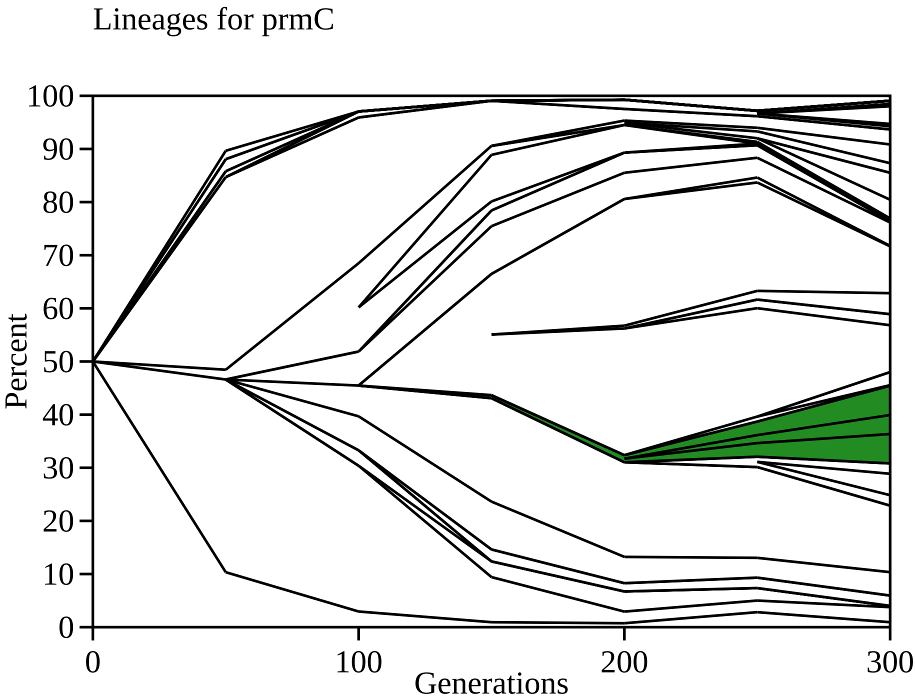

Lineages for prmC
Percent
Generations
100
90
80
70
60
50
40
30
20
10
0
0
100
200
300

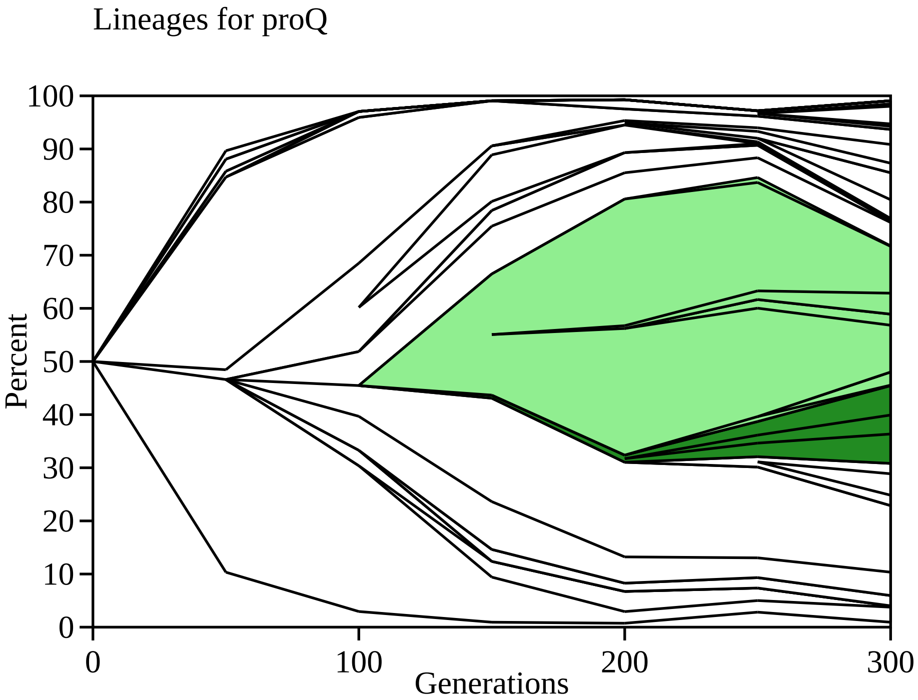

Lineages for proQ
Percent
Generations
0
10
20
30
40
50
60
70
80
90
100
0
100
200
300

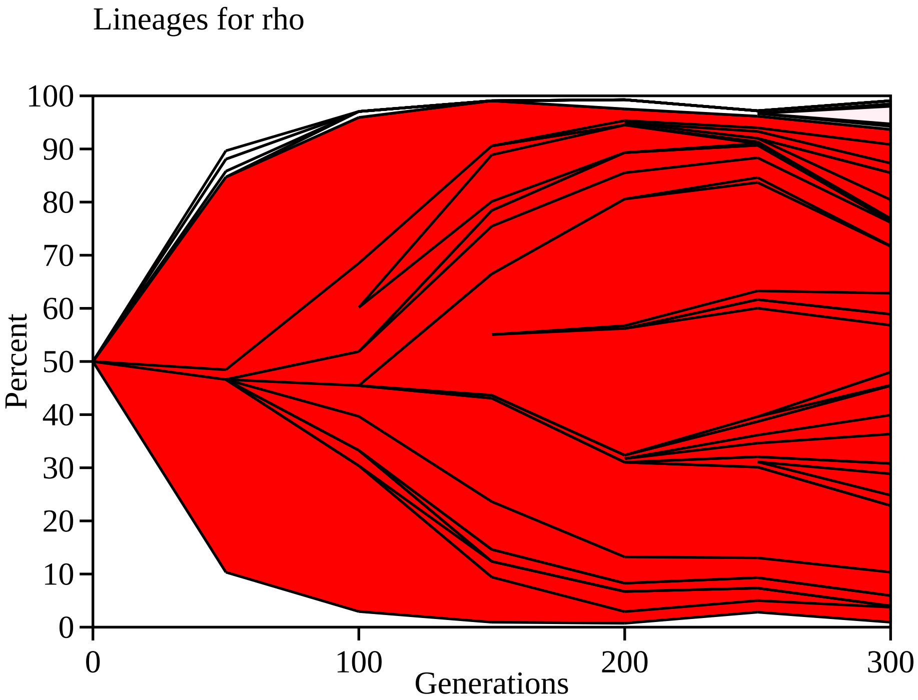

Lineages for rho
Percent
Generations
100
90
80
70
60
50
40
30
20
10
0
0
100
200
300

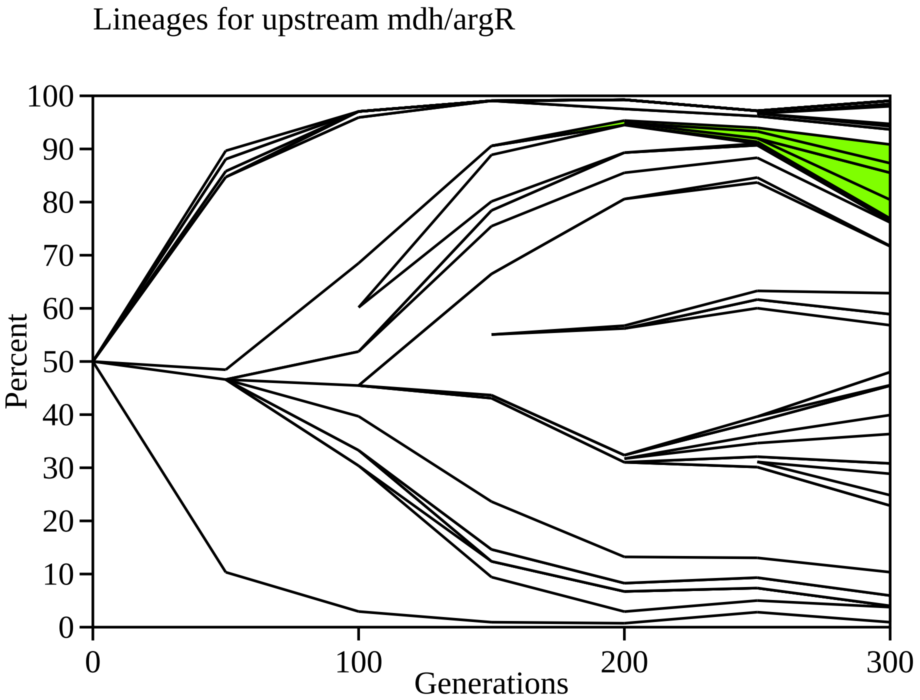

Lineages for upstream mdh/argR
Percent
100
90
80
70
60
50
40
30
20
10
0
0
100
200
300
Generations

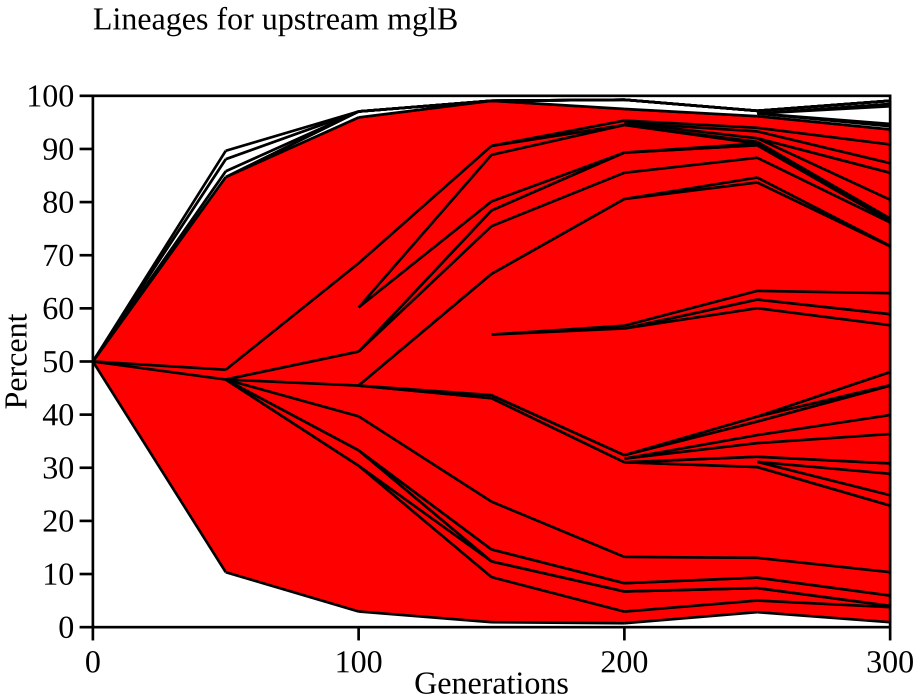
Lineages for upstream mglB
Percent
100
90
80
70
60
50
40
30
20
10
0
0
100
200
300
Generations

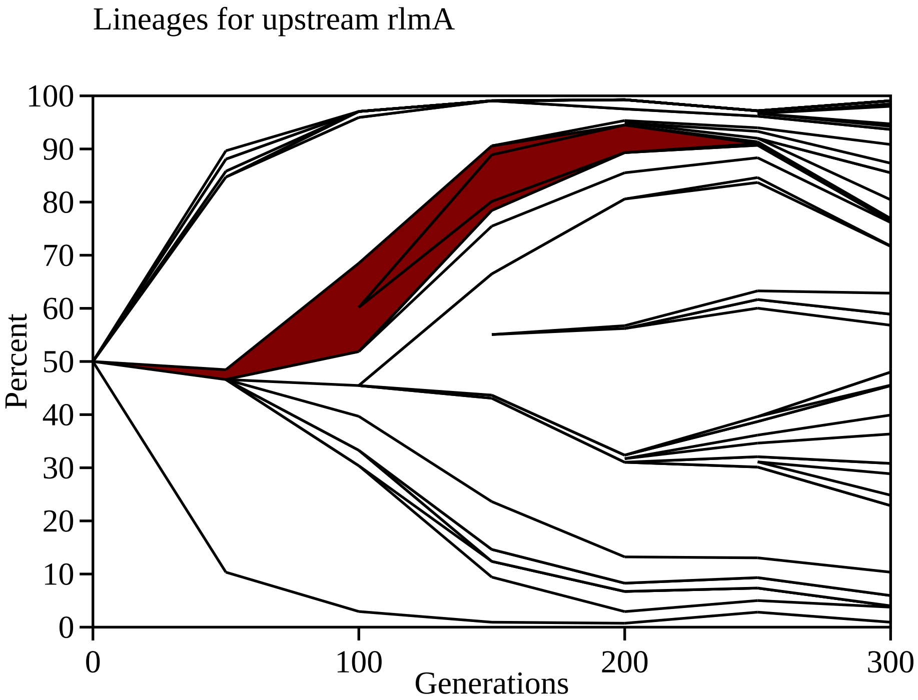
Lineages for upstream rlmA
Percent
100
90
80
70
60
50
40
30
20
10
0
0
100
200
300
Generations

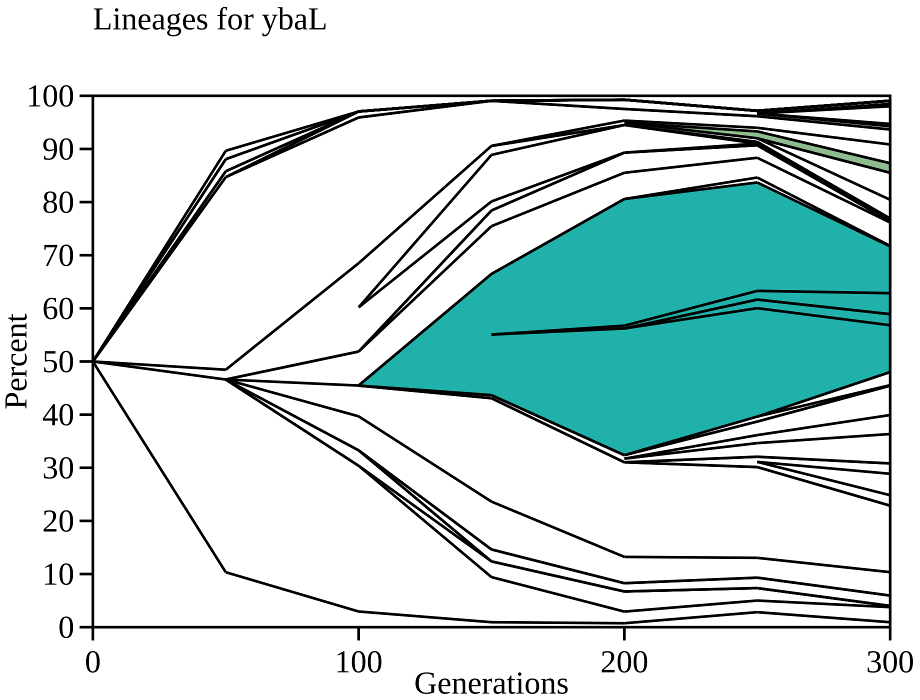

Lineages for ybaL
Percent
Generations
0
10
20
30
40
50
60
70
80
90
100
0
100
200
300

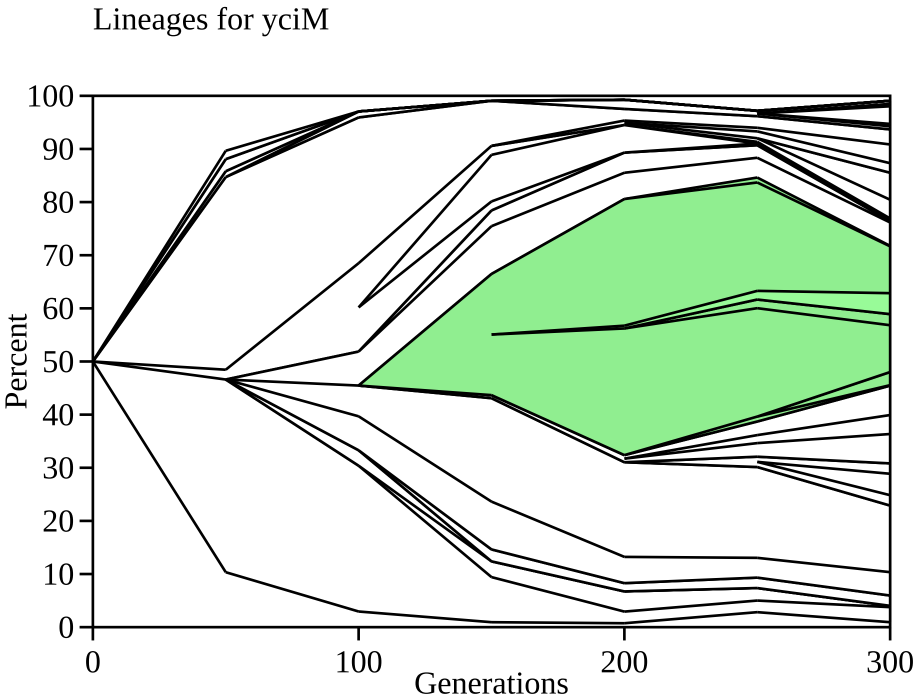

Lineages for yciM
100
90
80
70
60
50
40
30
20
10
0
Percent
0
100
200
300
Generations

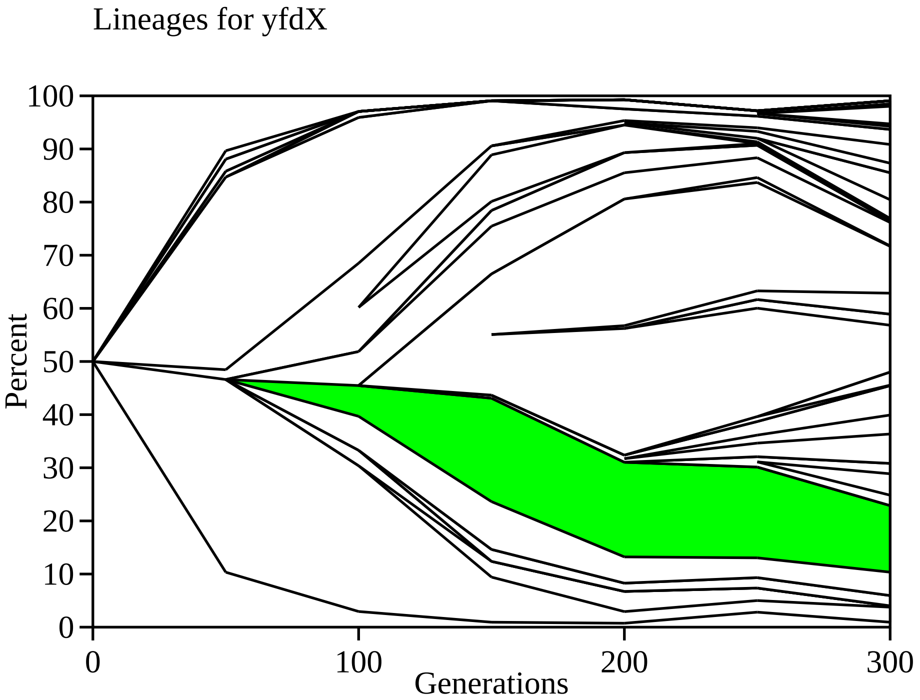
Lineages for yfdX
Percent
100
90
80
70
60
50
40
30
20
10
0
0
100
200
300
Generations

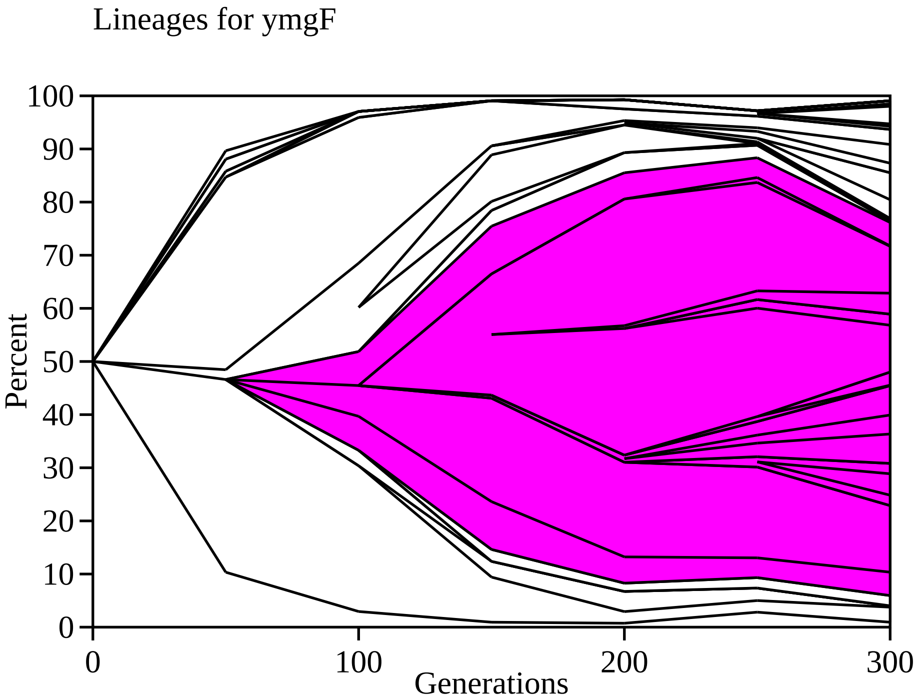

Lineages for ymgF
Percent
Generations
0
10
20
30
40
50
60
70
80
90
100
0
100
200
300

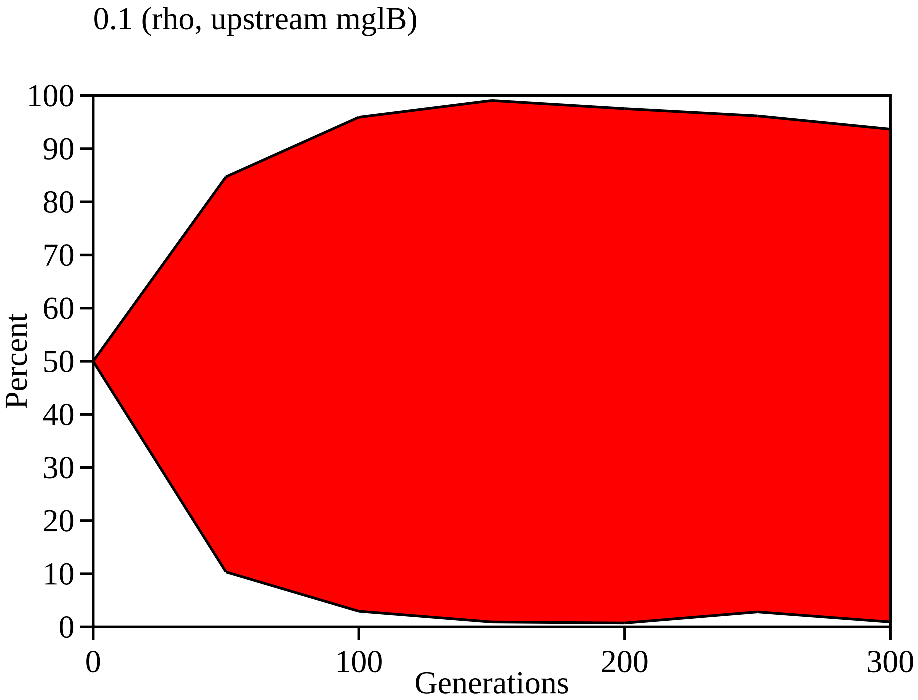
0.1 (rho, upstream mglB)
100
90
80
70
60
50
40
30
20
10
0
Percent
0
100
200
300
Generations

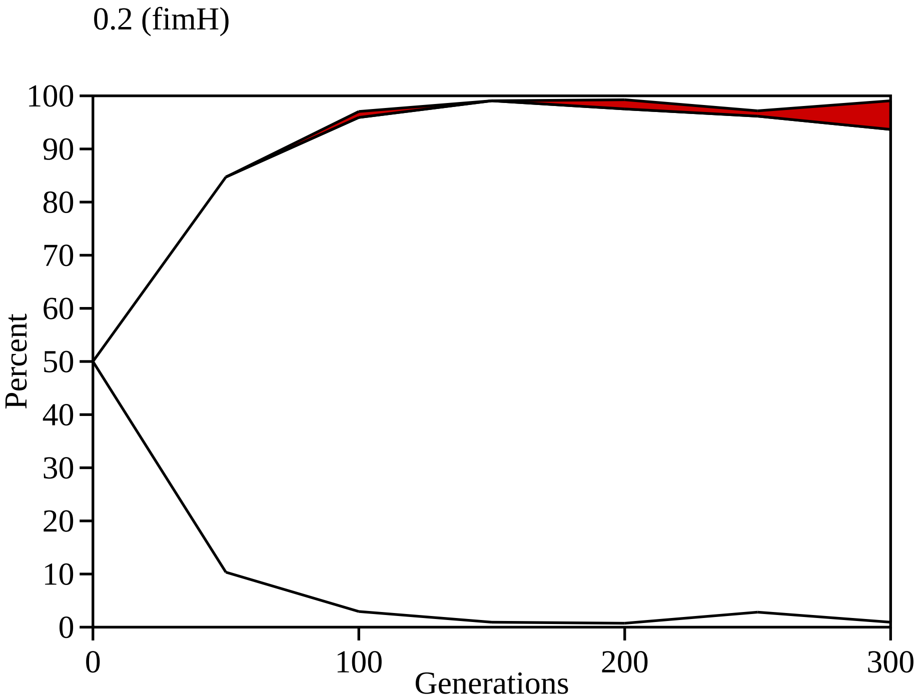
0.2 (fimH)
Percent
Generations
100
90
80
70
60
50
40
30
20
10
0
0
100
200
300

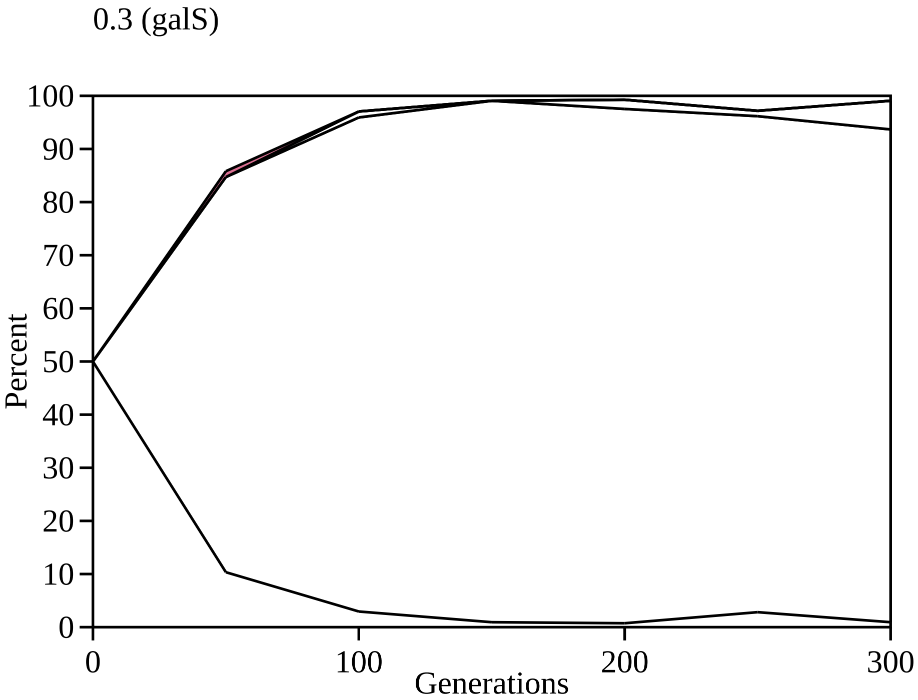
0.3 (galS)
Percent
100
90
80
70
60
50
40
30
20
10
0
0
100
200
300
Generations

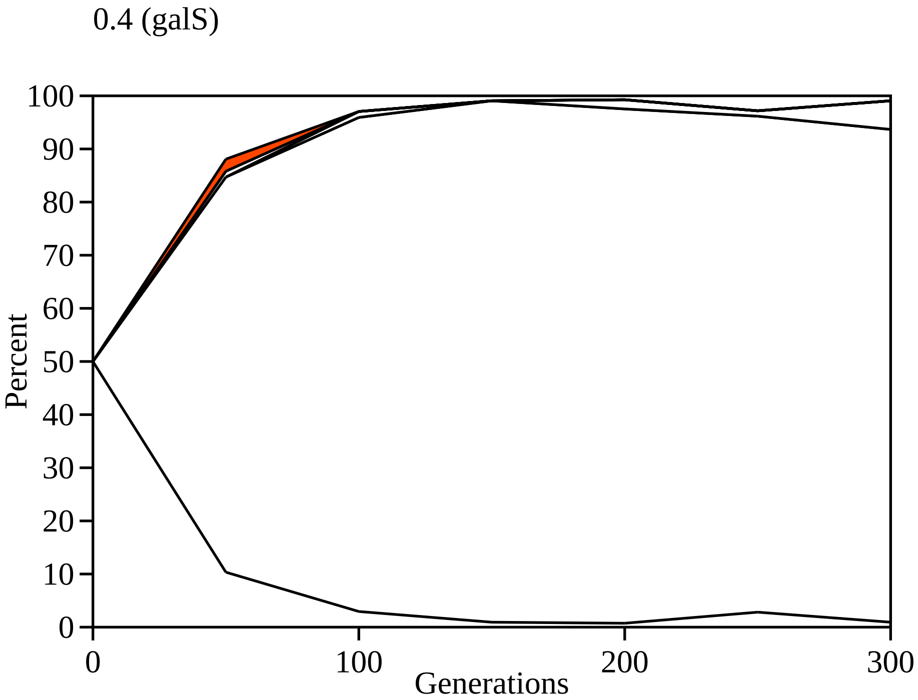

0.4 (galS)
100
90
80
70
60
50
40
30
20
10
0
Percent
0
100
200
300
Generations

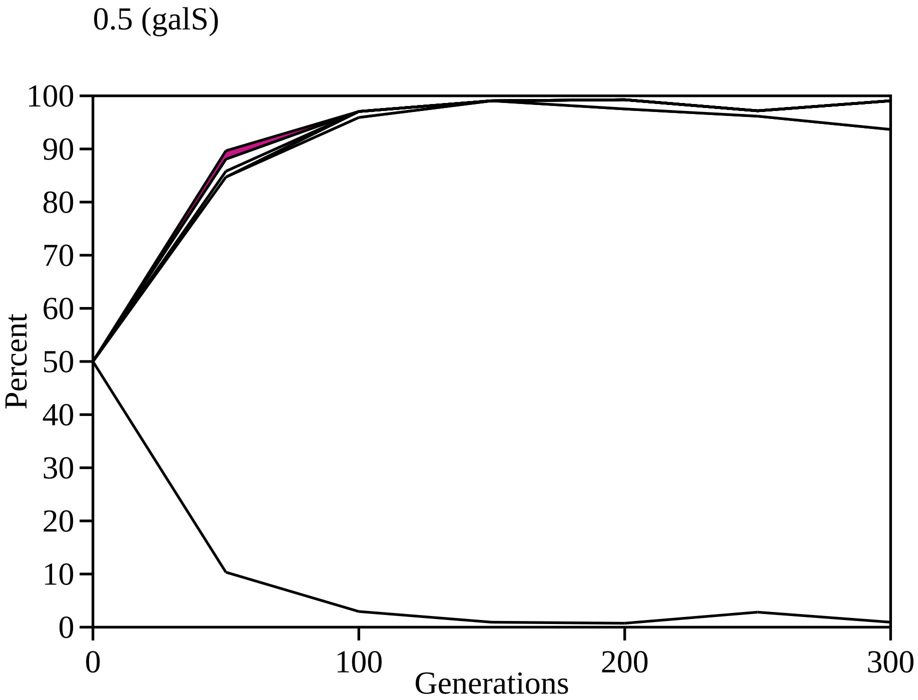
0.5 (galS)
Percent
100
90
80
70
60
50
40
30
20
10
0
0
100
200
300
Generations

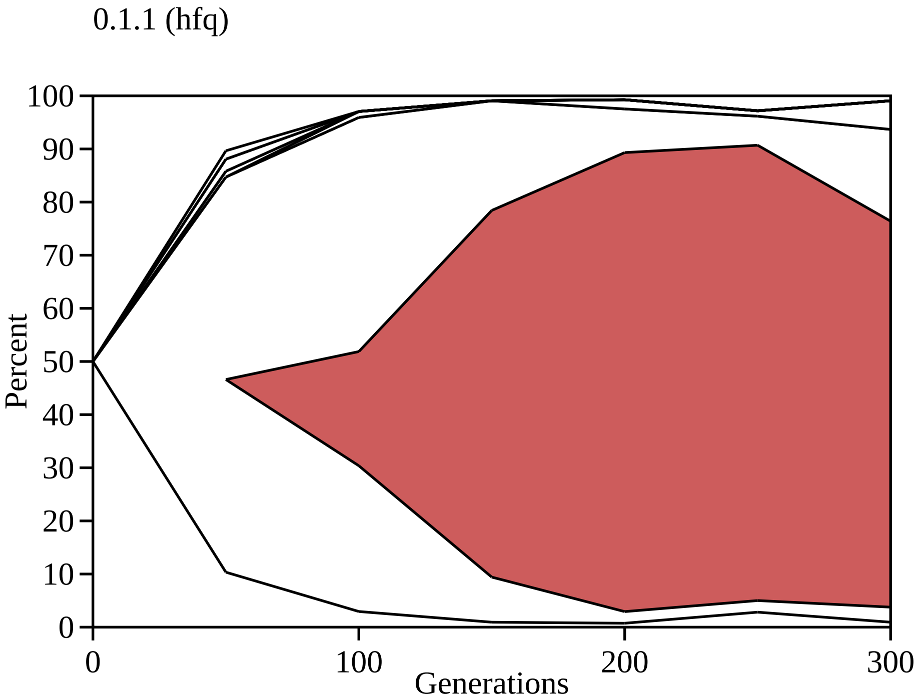
0.1.1 (hfq)
Percent
100
90
80
70
60
50
40
30
20
10
0
0
100
200
300
Generations

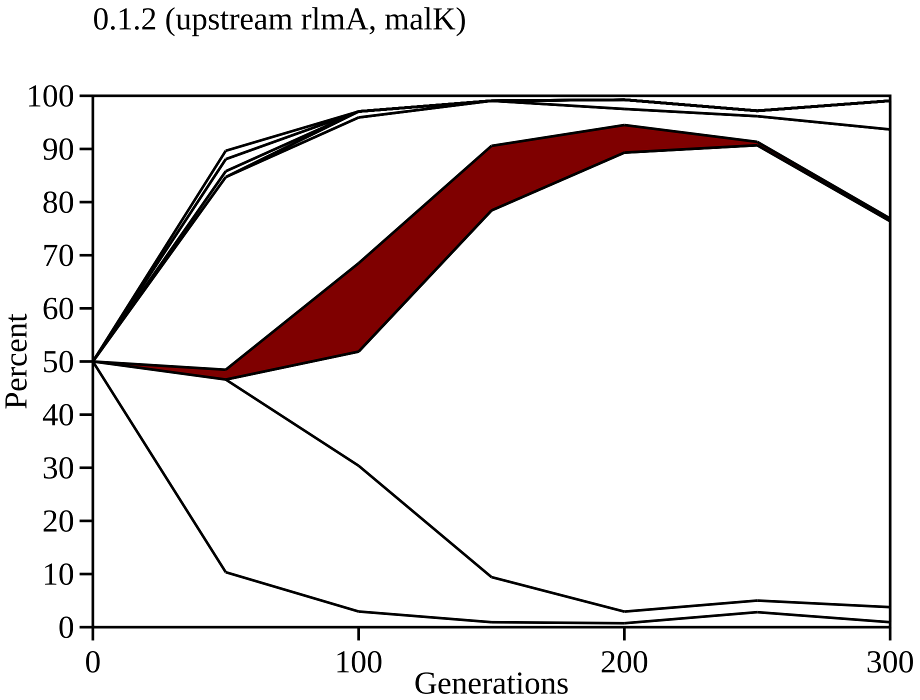
0.1.2 (upstream rlmA, malK)
Percent
Generations
0
10
20
30
40
50
60
70
80
90
100
0
100
200
300

0.1.3 (opgG, lptD, hfq, upstream mdh/argR, malT)

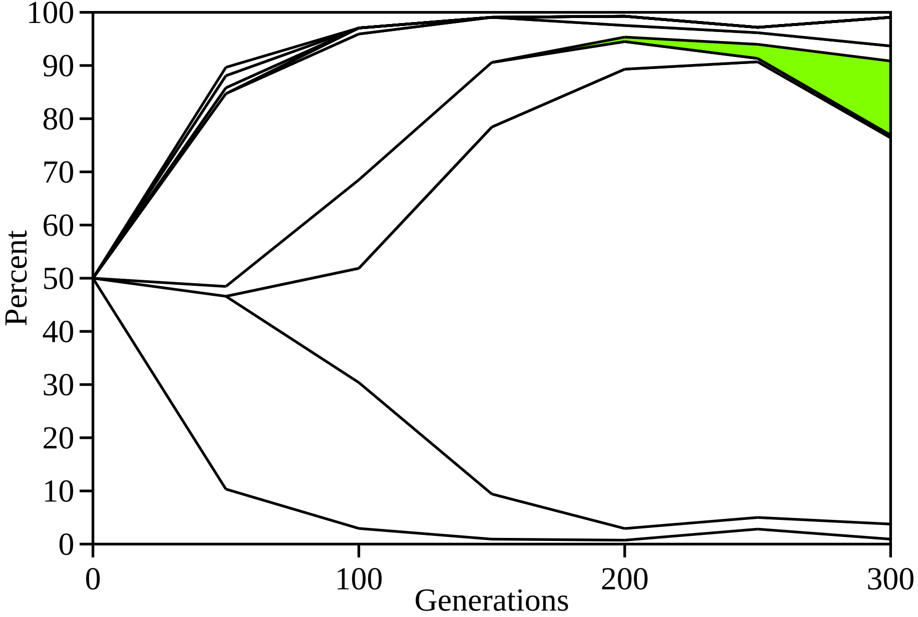

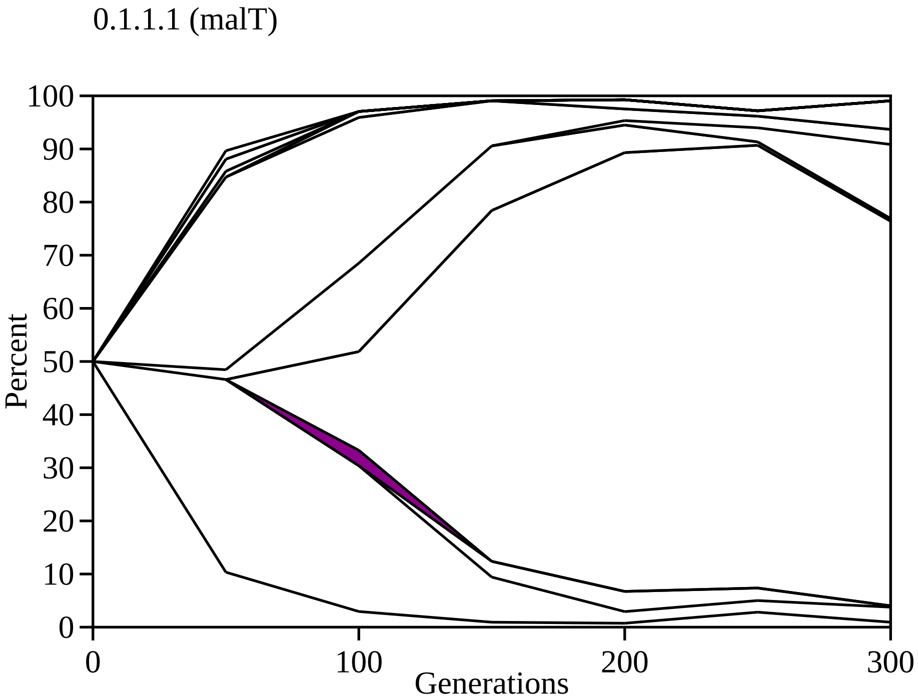

0.1.1.1 (malT)
Percent
100
90
80
70
60
50
40
30
20
10
0
0
100
200
300
Generations

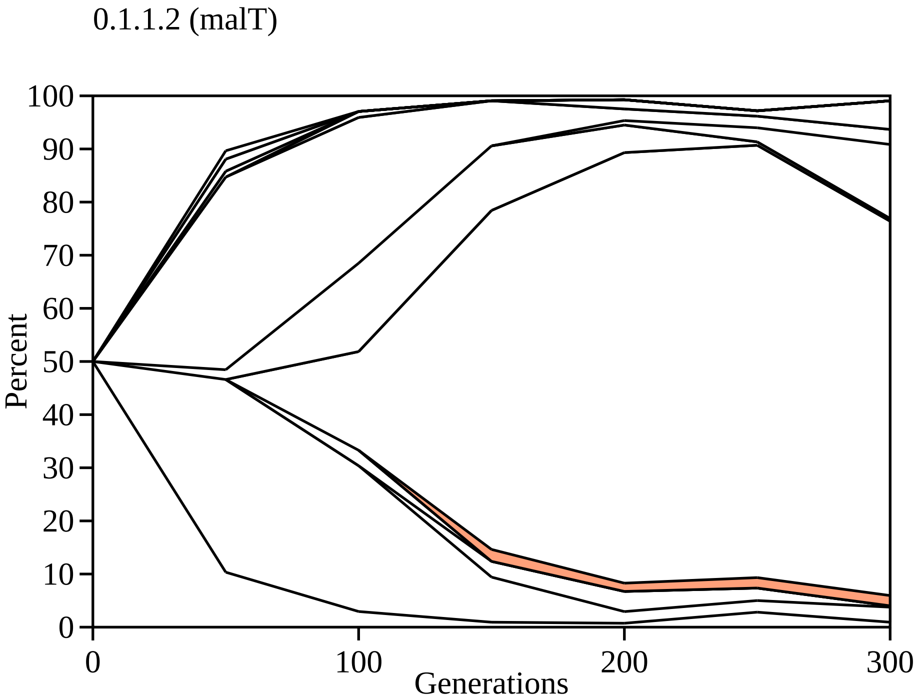

0.1.1.2 (malT)
Percent
Generations
0
10
20
30
40
50
60
70
80
90
100
0
100
200
300

0.1.1.3 (ymgF, malT)

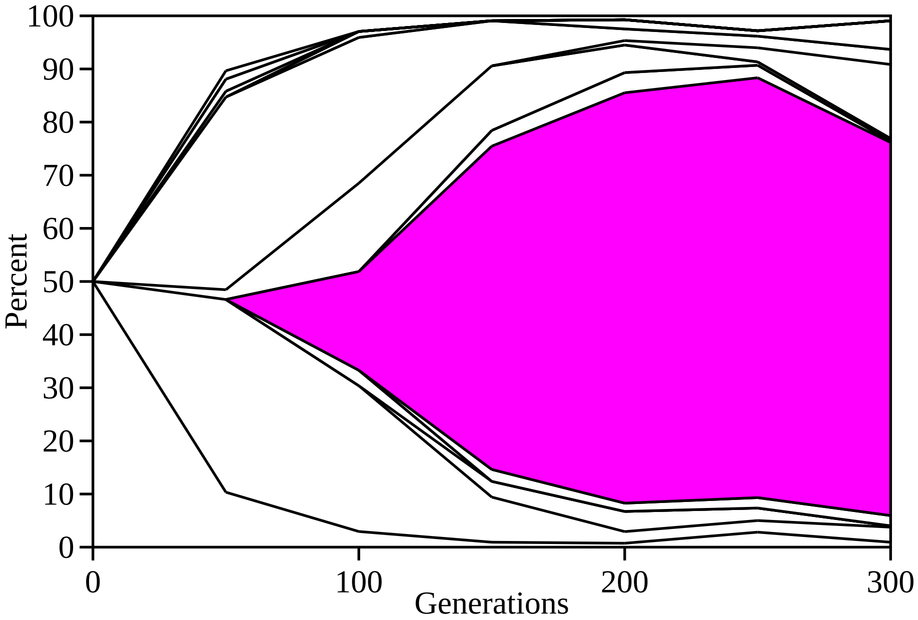

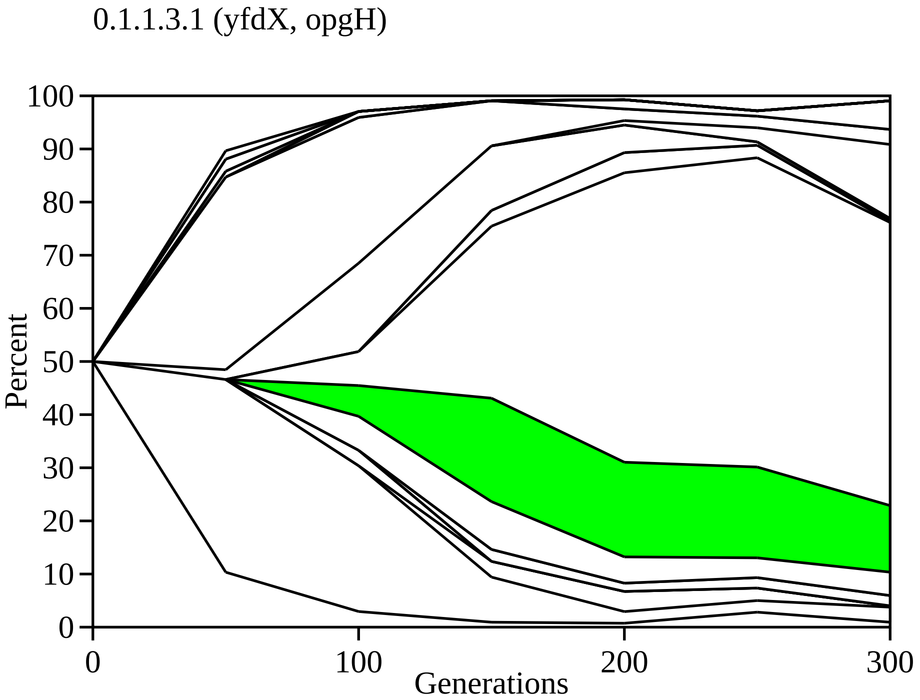
0.1.1.3.1 (yfdX, opgH)
Percent
100
90
80
70
60
50
40
30
20
10
0
0
100
200
300
Generations

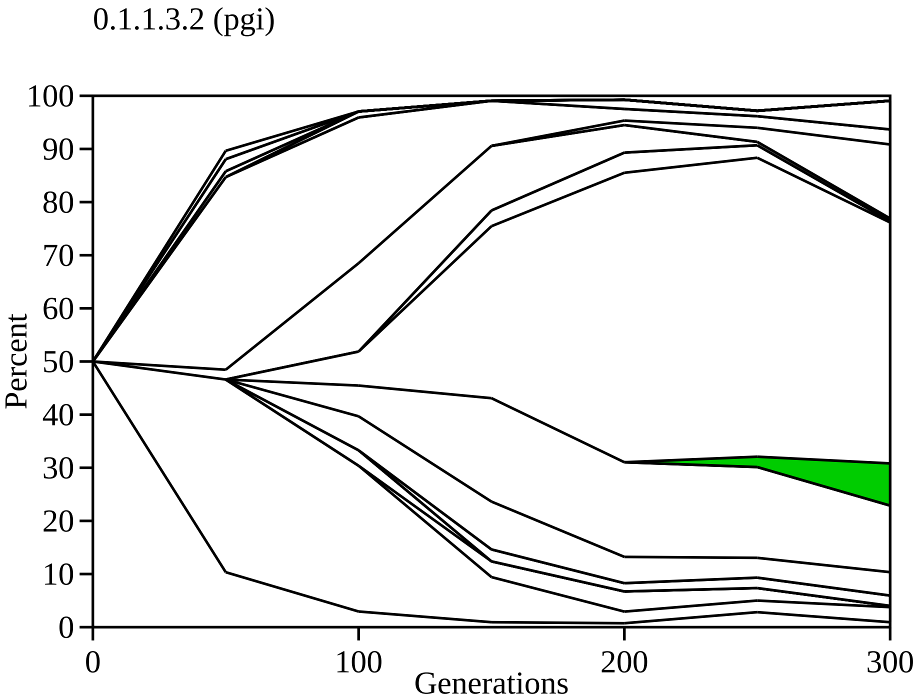

0.1.1.3.2 (pgi)
Percent
100
90
80
70
60
50
40
30
20
10
0
0
100
200
300
Generations

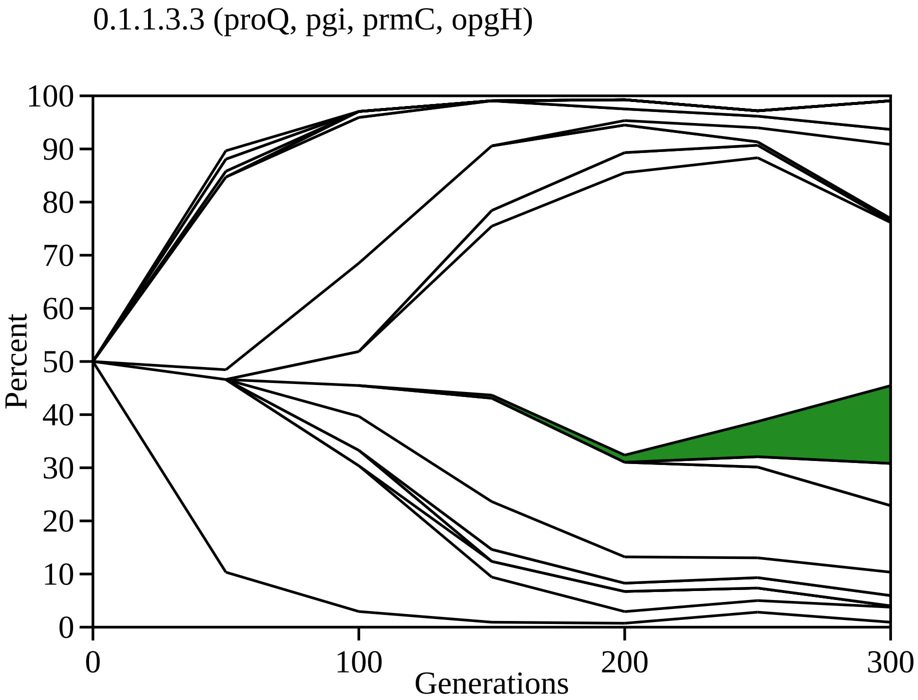
0.1.1.3.3 (proQ, pgi, prmC, opgH)
Percent
Generations
0
10
20
30
40
50
60
70
80
90
100
0
100
200
300

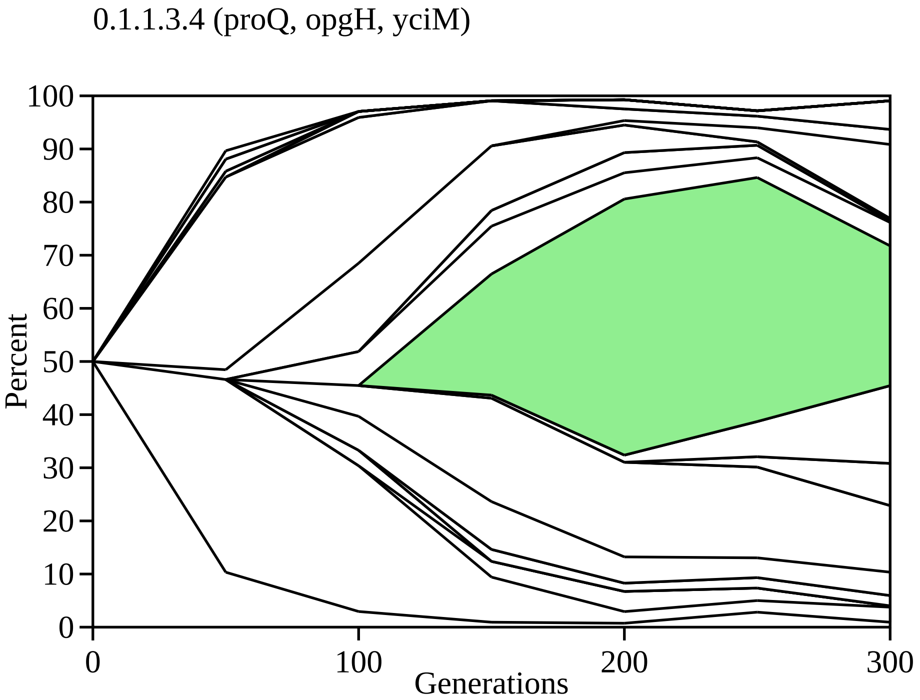
0.1.1.3.4 (proQ, opgH, yciM)
Percent
100
90
80
70
60
50
40
30
20
10
0
0
100
200
300
Generations

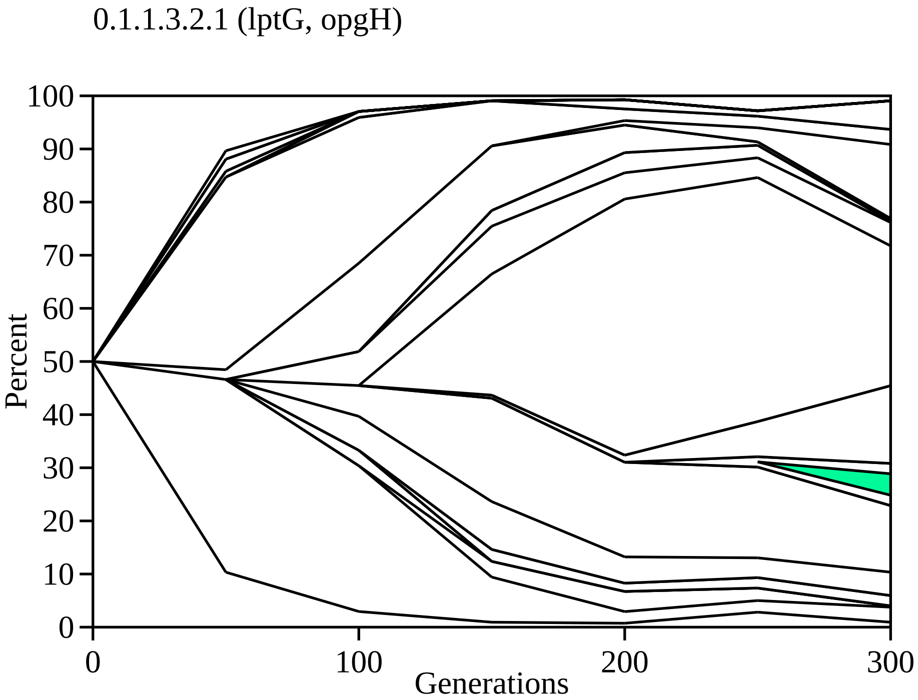

0.1.1.3.2.1 (lptG, opgH)
100
90
80
70
60
50
40
30
20
10
0
Percent
0
100
200
300
Generations

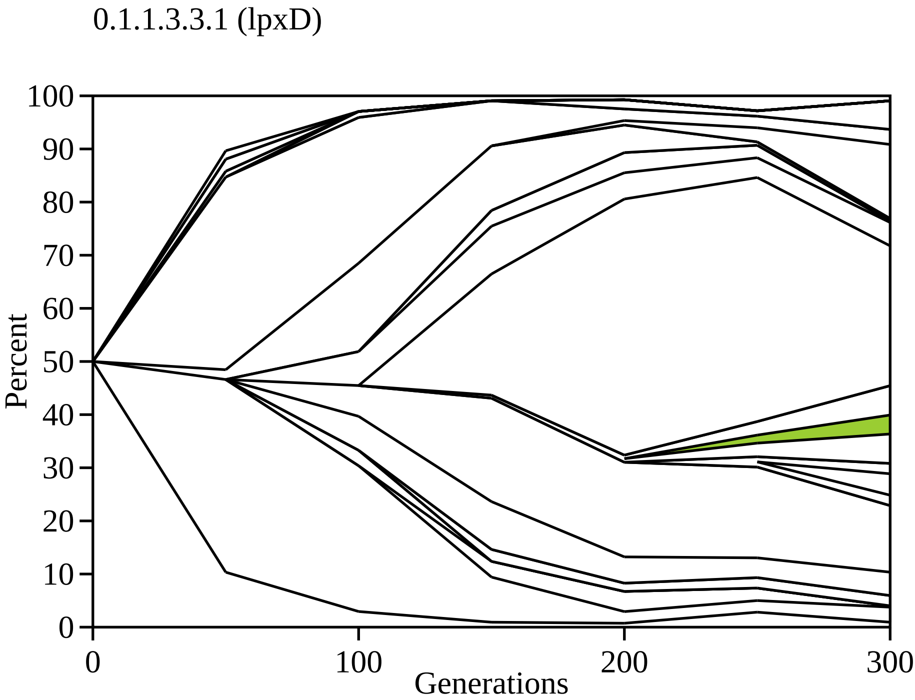
0.1.1.3.3.1 (lpxD)
Percent
100
90
80
70
60
50
40
30
20
10
0
0
100
200
300
Generations

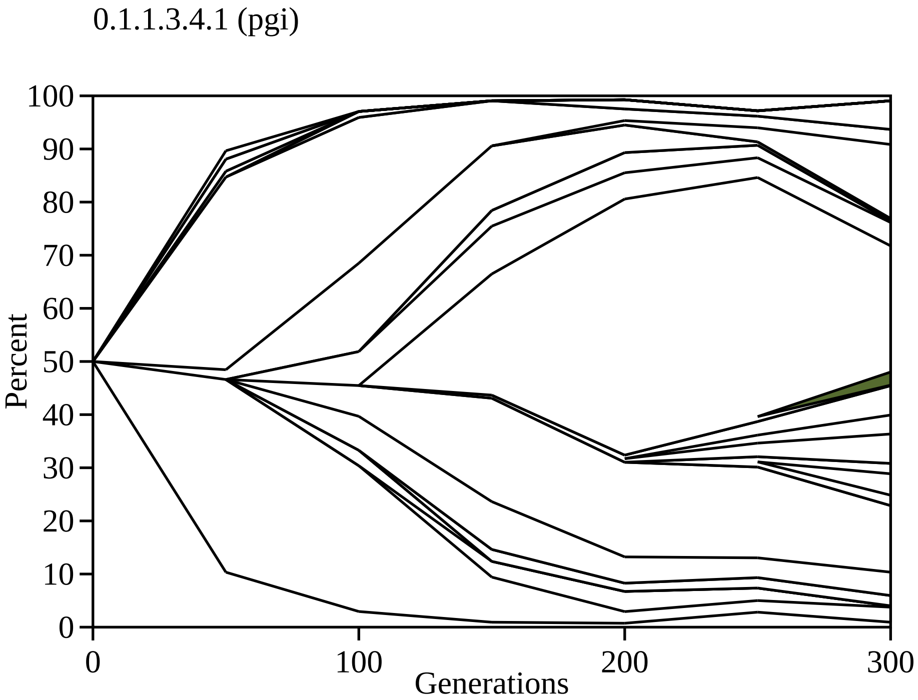
0.1.1.3.4.1 (pgi)
Percent
100
90
80
70
60
50
40
30
20
10
0
0
100
200
300
Generations

0.1.1.3.4.2 (ybaL)

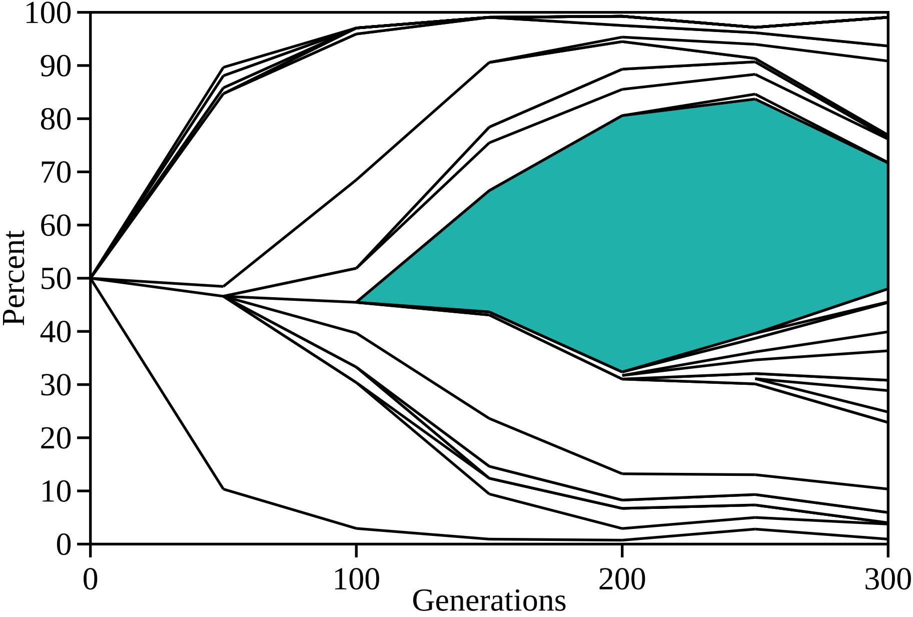
100
90
80
70
60
50
40
30
20
10
0
Percent
0
100
200
300
Generations

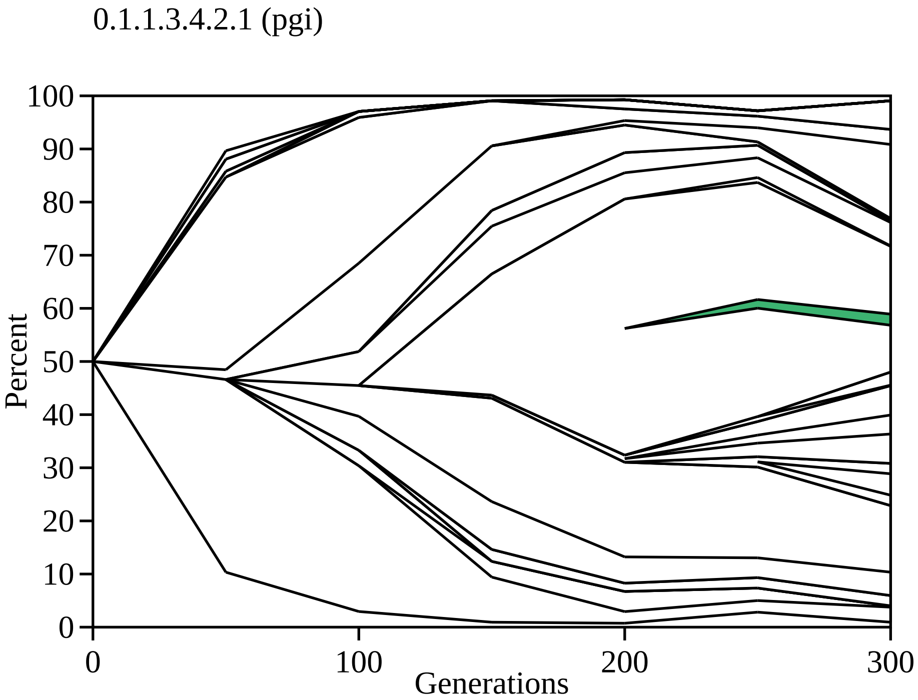
0.1.1.3.4.2.1 (pgi)
Percent
100
90
80
70
60
50
40
30
20
10
0
0
100
200
300
Generations

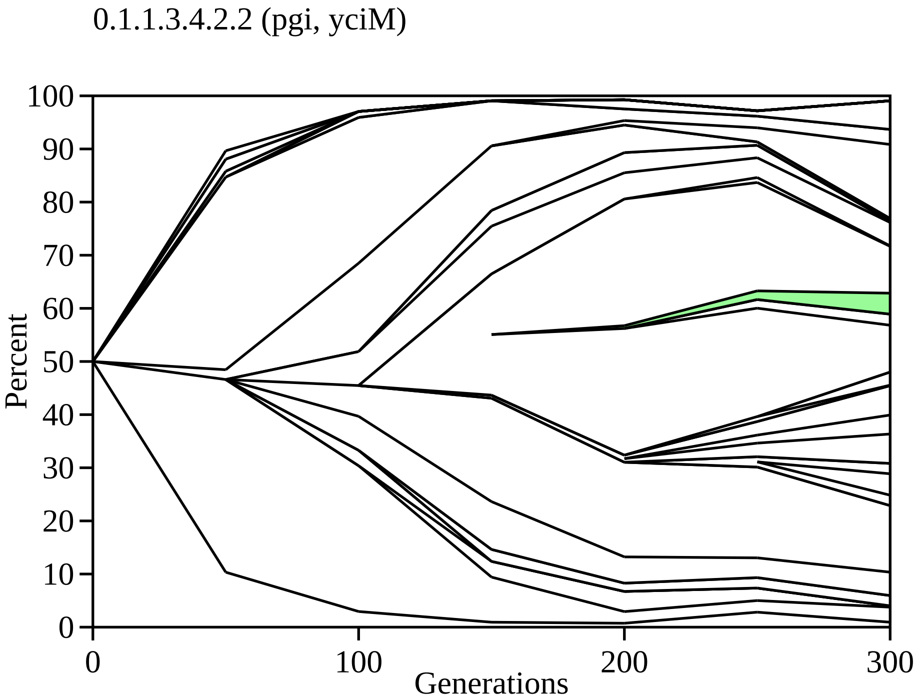
0.1.1.3.4.2.2 (pgi, yciM)
Percent
100
90
80
70
60
50
40
30
20
10
0
0
100
200
300
Generations

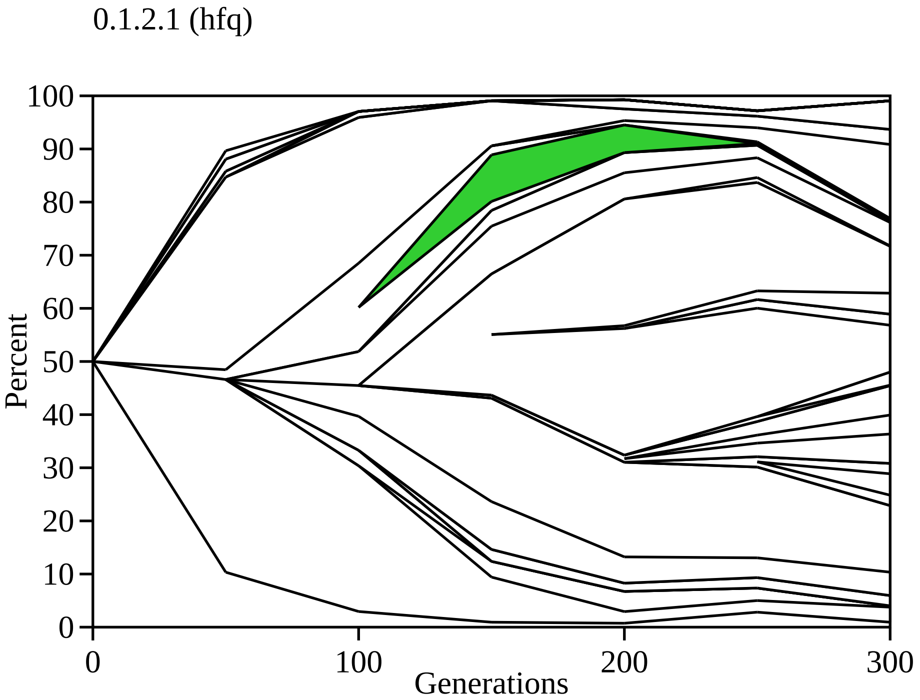

0.1.2.1 (hfq)
Percent
0
10
20
30
40
50
60
70
80
90
100
0
100
200
300
Generations

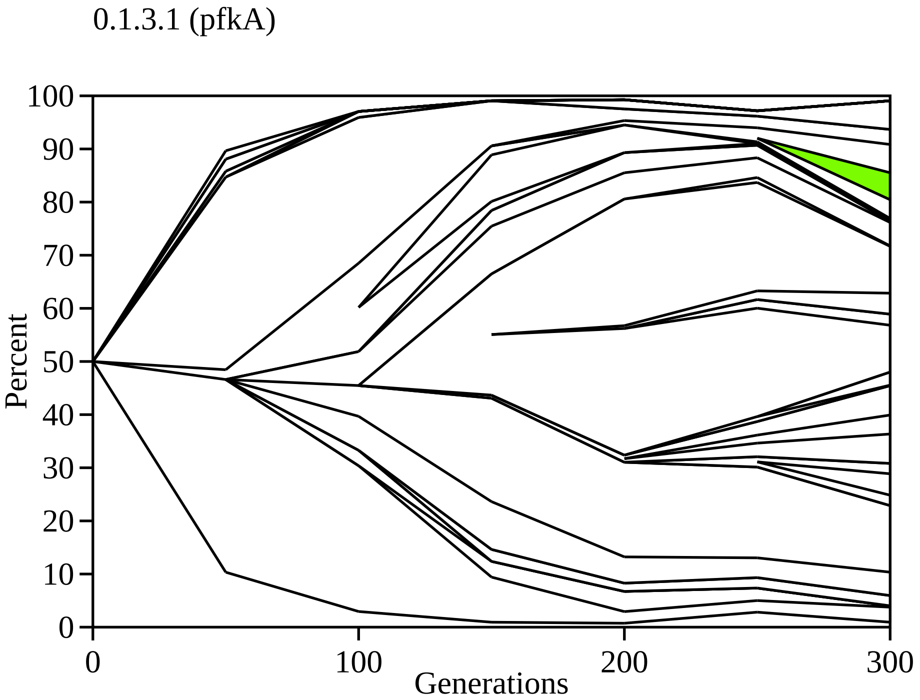
0.1.3.1 (pfkA)
100
90
80
70
60
50
40
30
20
10
0
Percent
0
100
200
300
Generations

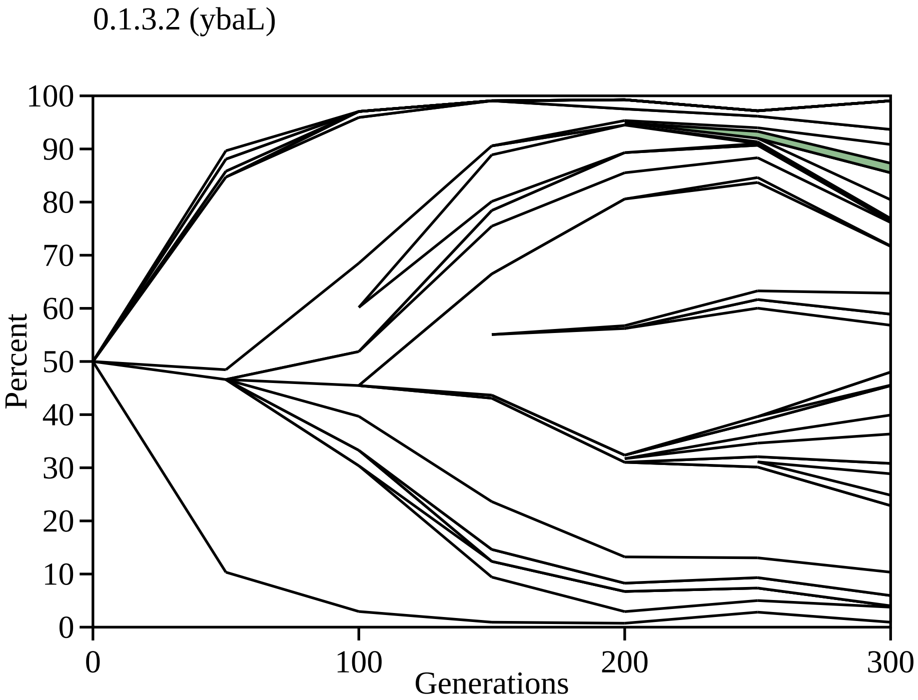
0.1.3.2 (ybaL)
Percent
Generations
0
10
20
30
40
50
60
70
80
90
100
0
100
200
300

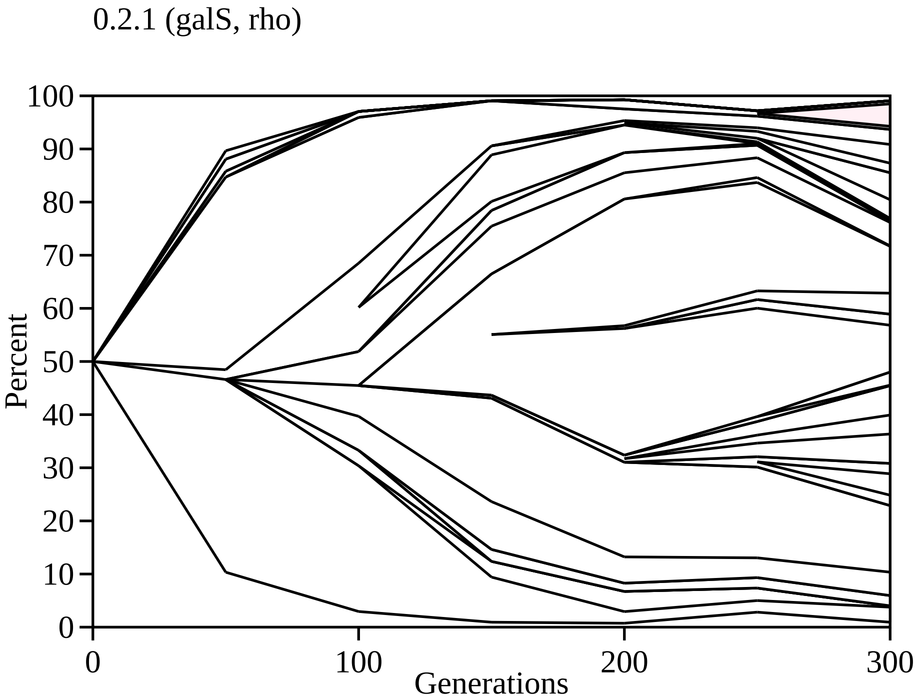

0.2.1 (galS, rho)
Percent
100
90
80
70
60
50
40
30
20
10
0
0
100
200
300
Generations

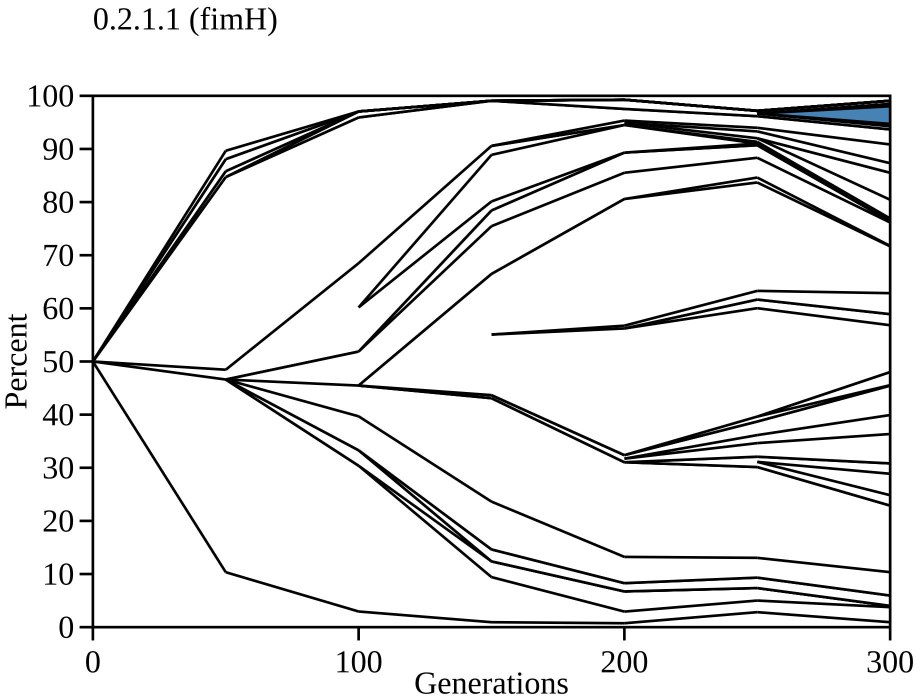
0.2.1.1 (fimH)
100
90
80
70
60
50
40
30
20
10
0
Percent
0
100
200
300
Generations

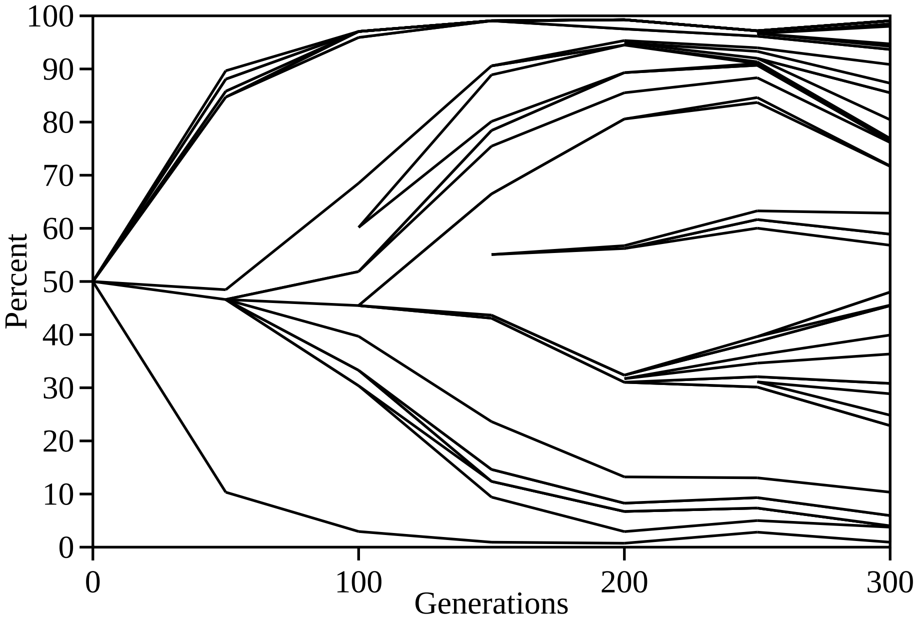

Percent
100
90
80
70
60
50
40
30
20
10
0
0
100
200
300
Generations
