# Supplementary material for: Evolutionary dynamics and structural consequences of de novo beneficial mutations and mutant lineages arising in a constant environment
Source: BMC Biol. 2021 Feb 4;19:20. doi: 10.1186/s12915-021-00954-0 (PMC7863352; doi:10.1186/s12915-021-00954-0)
Supplement: Supplementary file 8 — Additional file 8 Fig. S12. Locations of mutations in genes that were targets of adaptation, and their maximum frequencies, on both log and linear scales. [file 12915_2021_954_MOESM8_ESM.pdf]

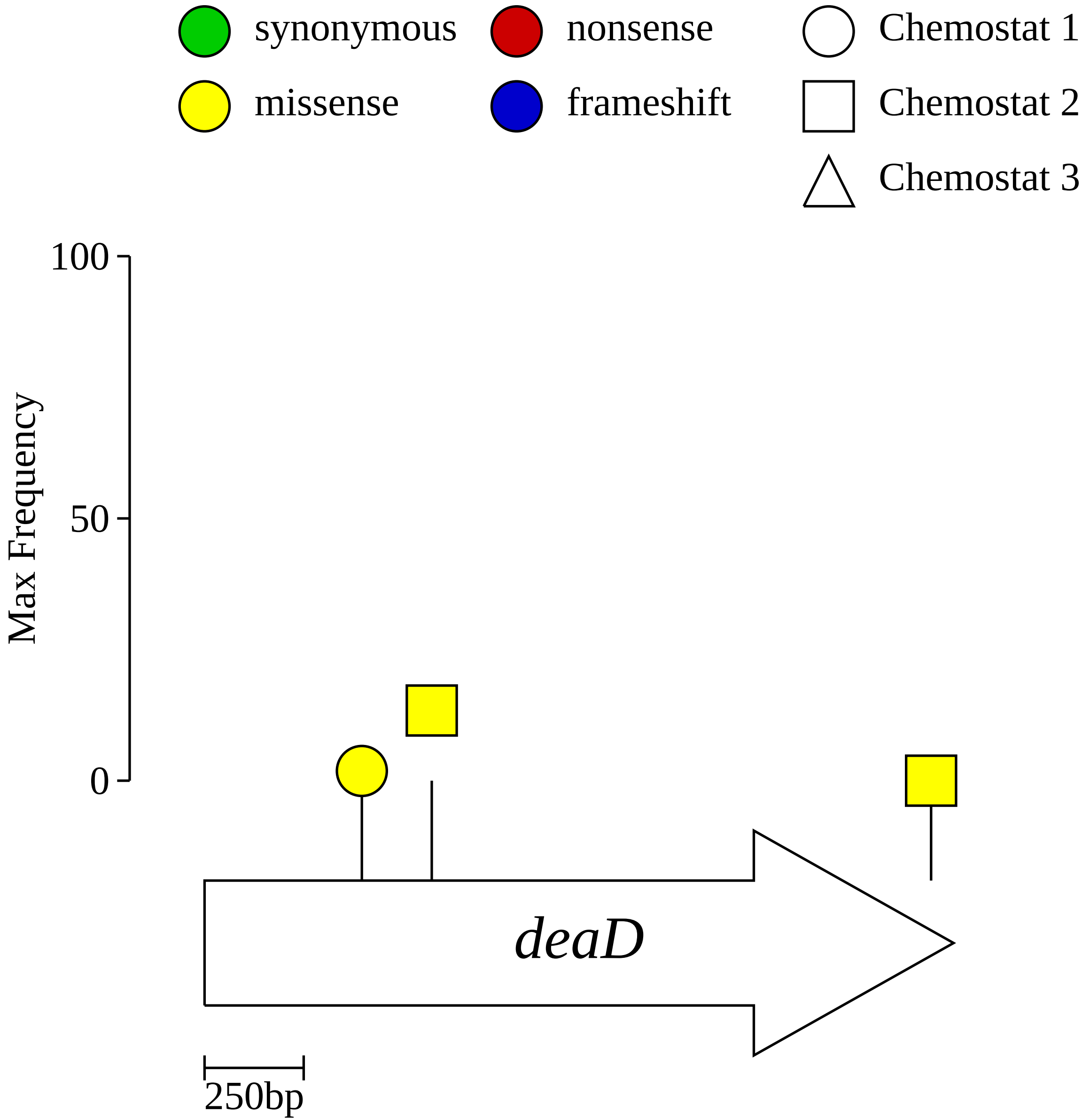
synonymous
nonsense
Chemostat 1
missense
frameshift
Chemostat 2
Chemostat 3
100
50
0
Max Frequency
deaD
250bp

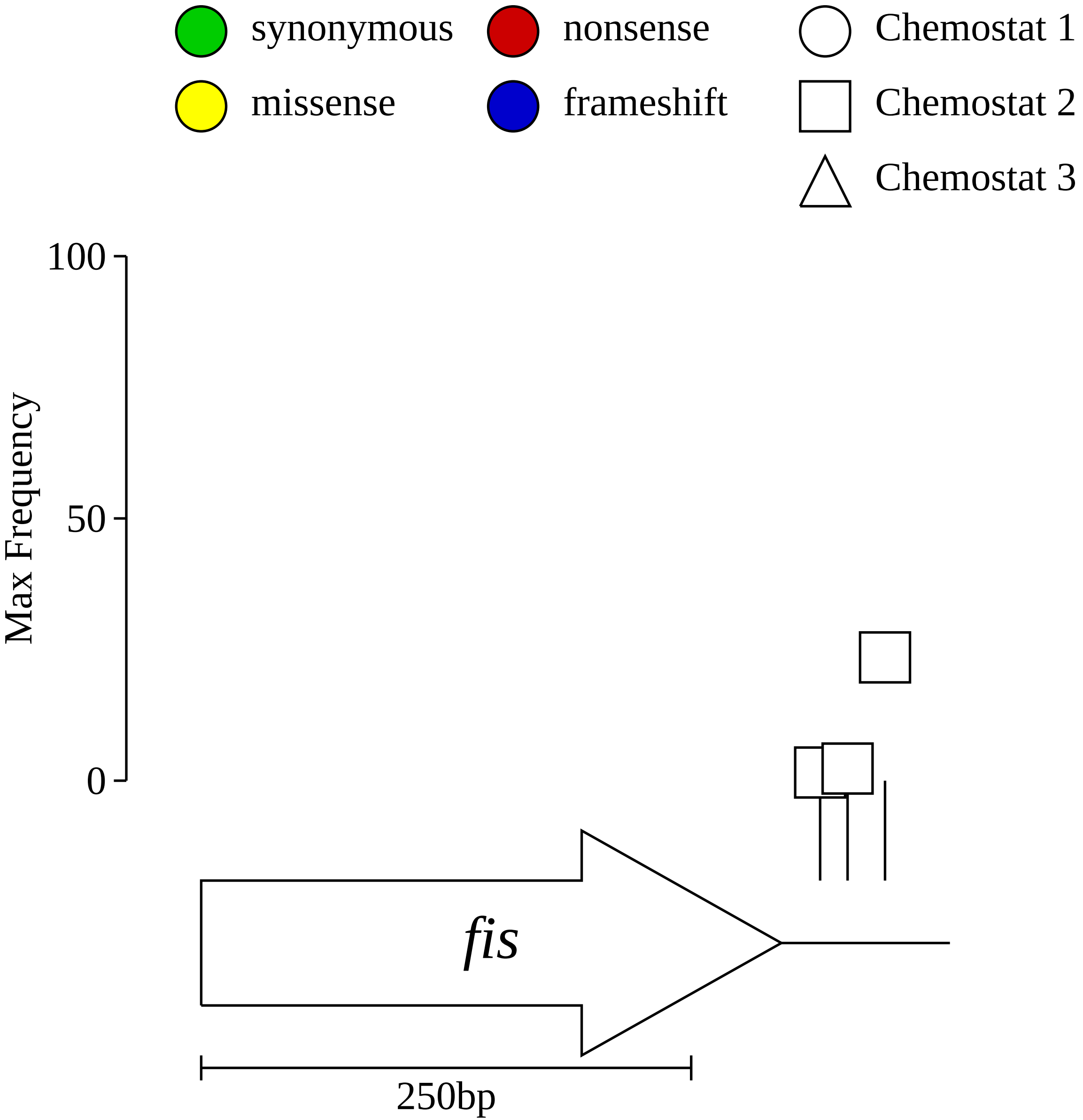
synonymous
nonsense
Chemostat 1
missense
frameshift
Chemostat 2
Chemostat 3
100
50
0
Max Frequency
fis
250bp

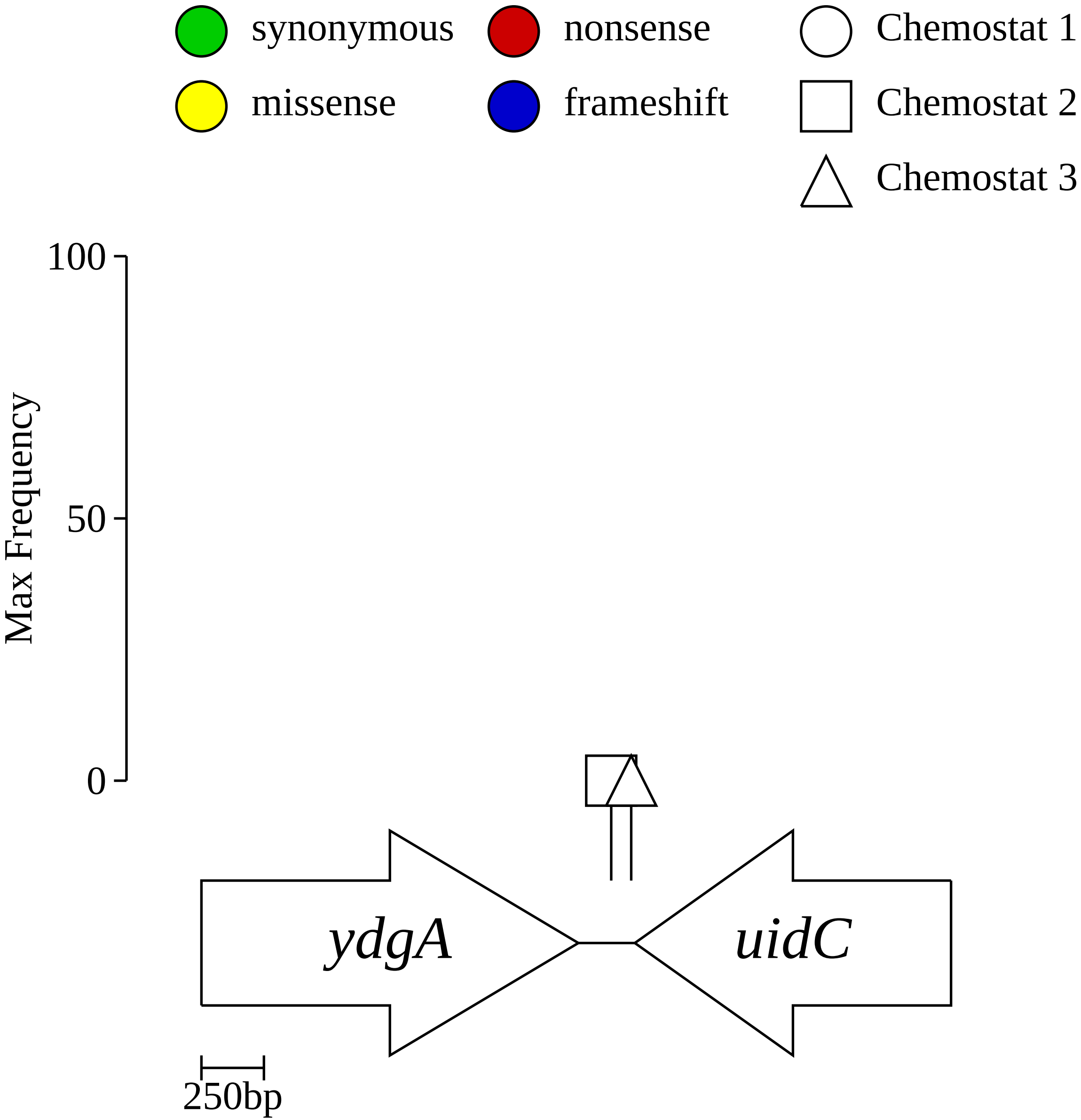
synonymous
nonsense
Chemostat 1
missense
frameshift
Chemostat 2
Chemostat 3
100
50
0
Max Frequency
ydgA
uidC
250bp

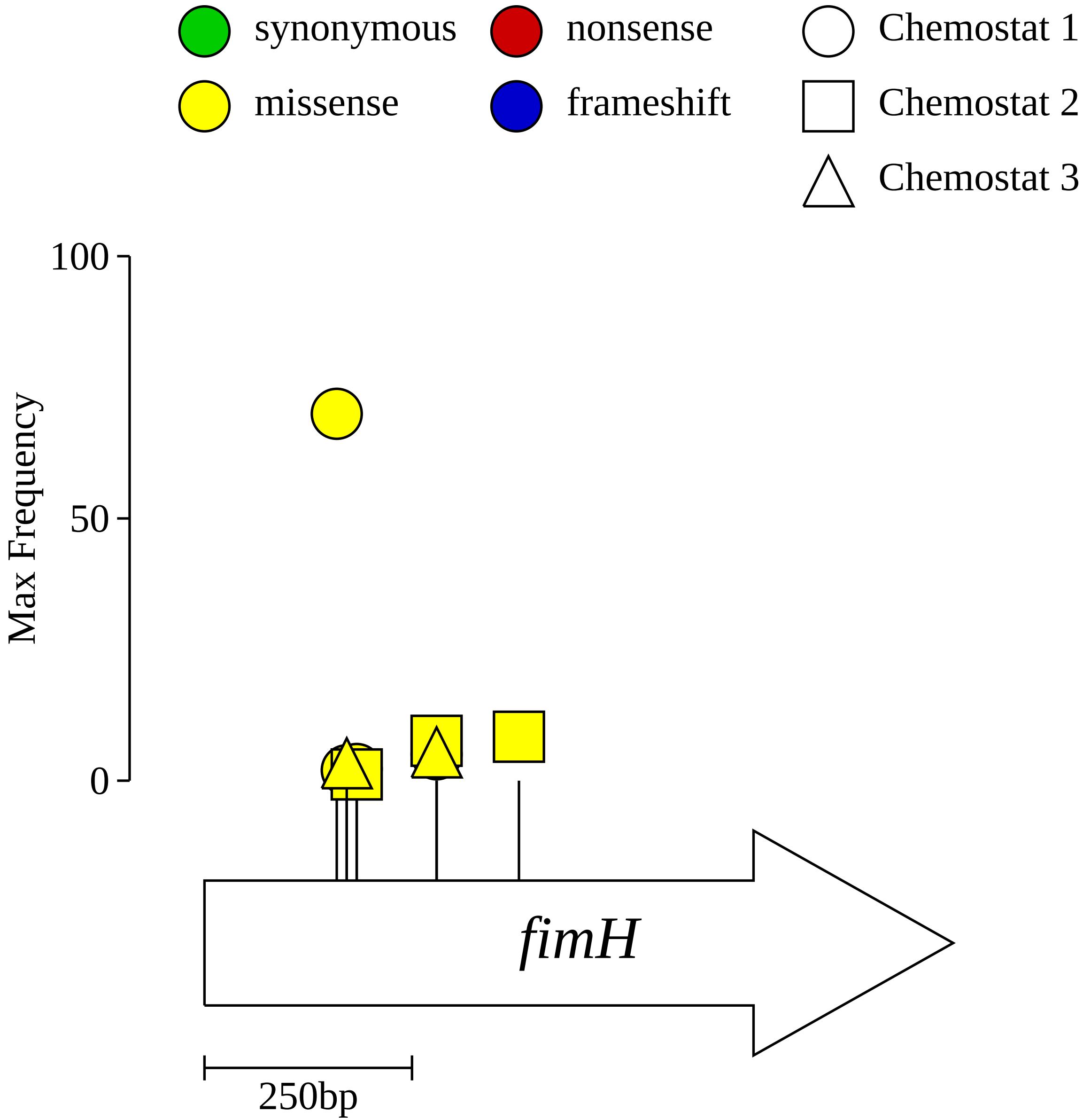
synonymous
missense
nonsense
frameshift
Chemostat 1
Chemostat 2
Chemostat 3
100
50
0
Max Frequency
fimH
250bp

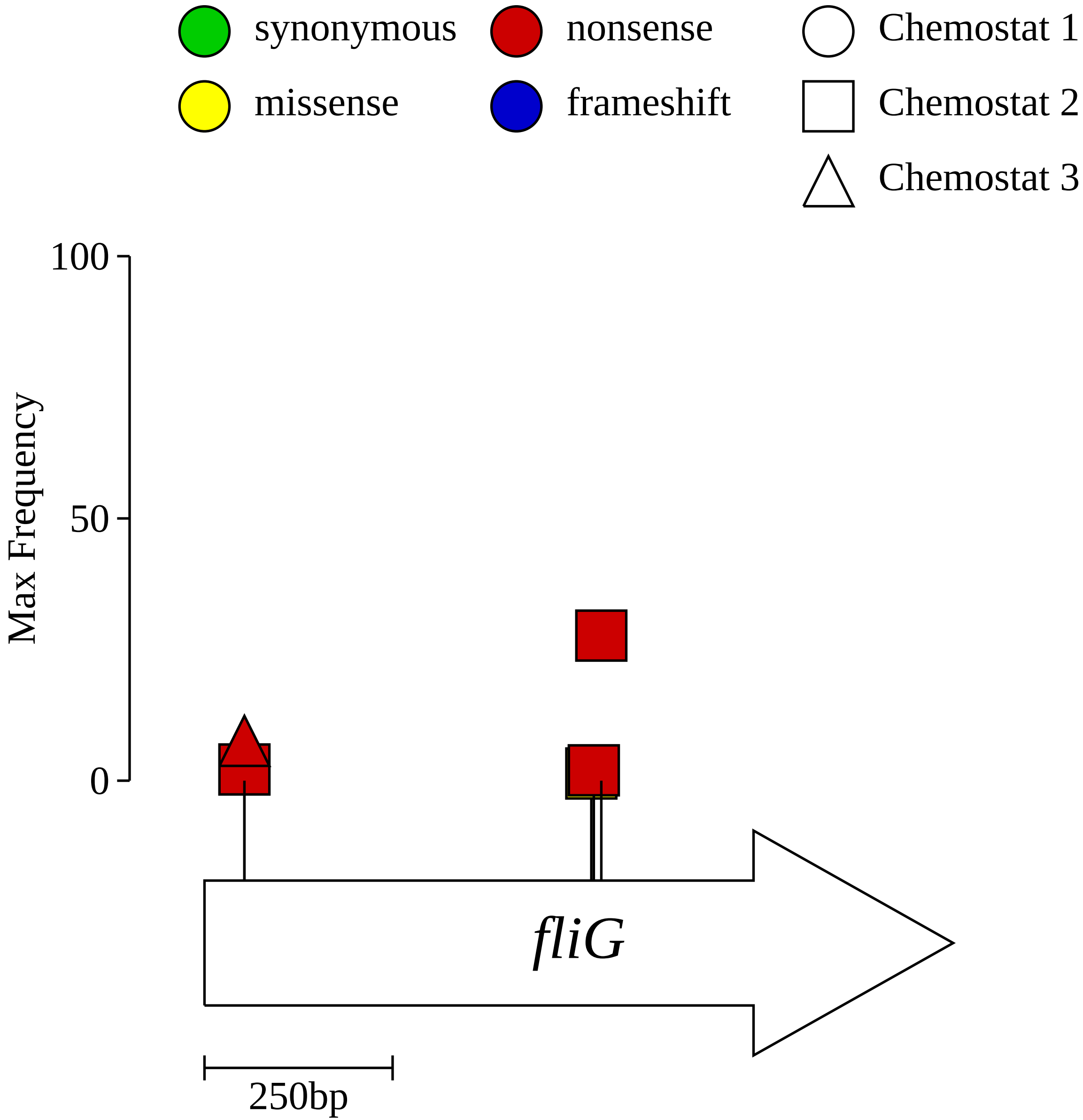

synonymous
missense
nonsense
frameshift
Chemostat 1
Chemostat 2
Chemostat 3
100
50
0
Max Frequency
fliG
250bp

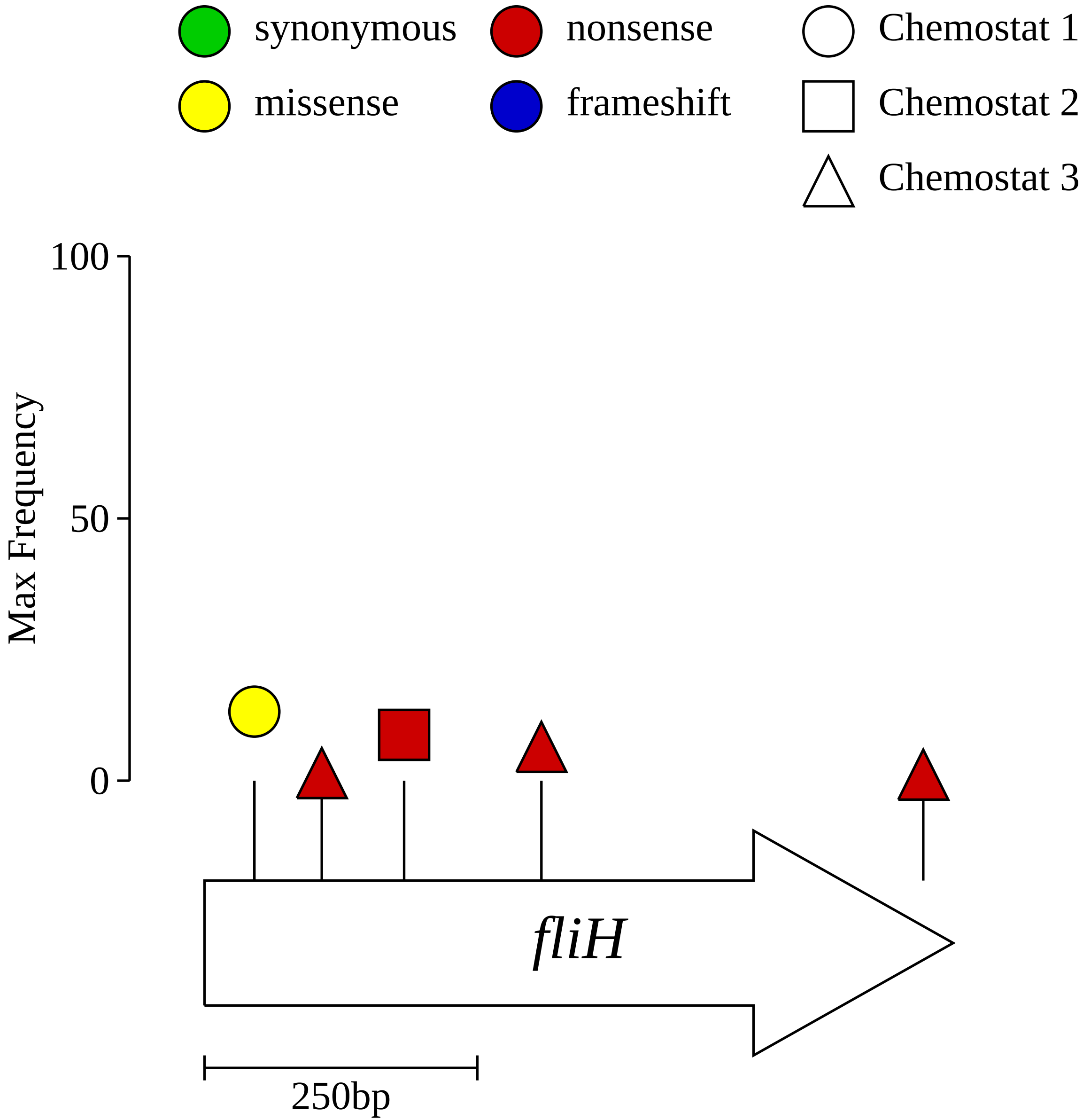
synonymous
nonsense
Chemostat 1
missense
frameshift
Chemostat 2
Chemostat 3
100
50
0
Max Frequency
fliH
250bp

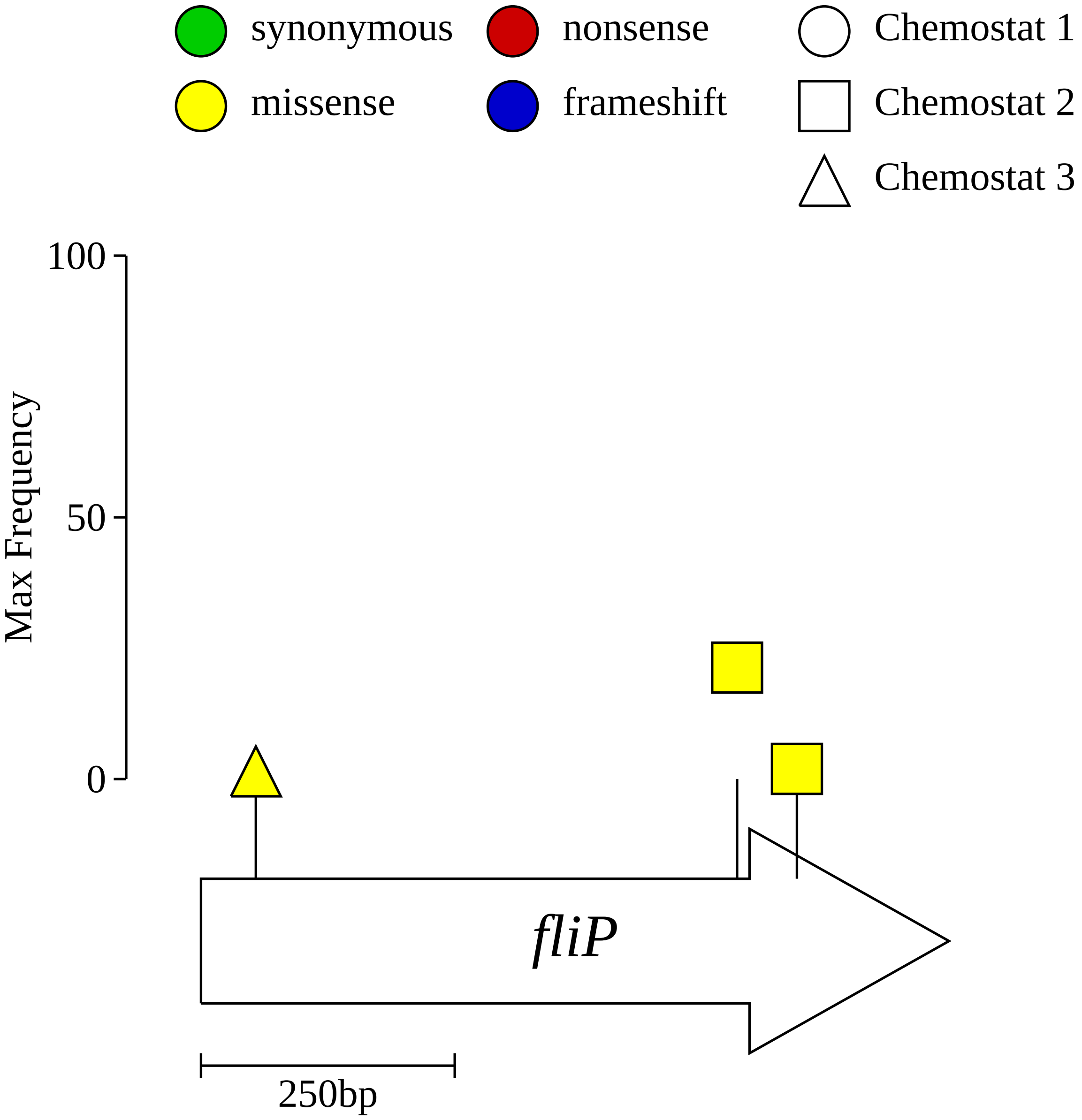

synonymous
nonsense
Chemostat 1
missense
frameshift
Chemostat 2
Chemostat 3
100
50
0
Max Frequency
fliP
250bp

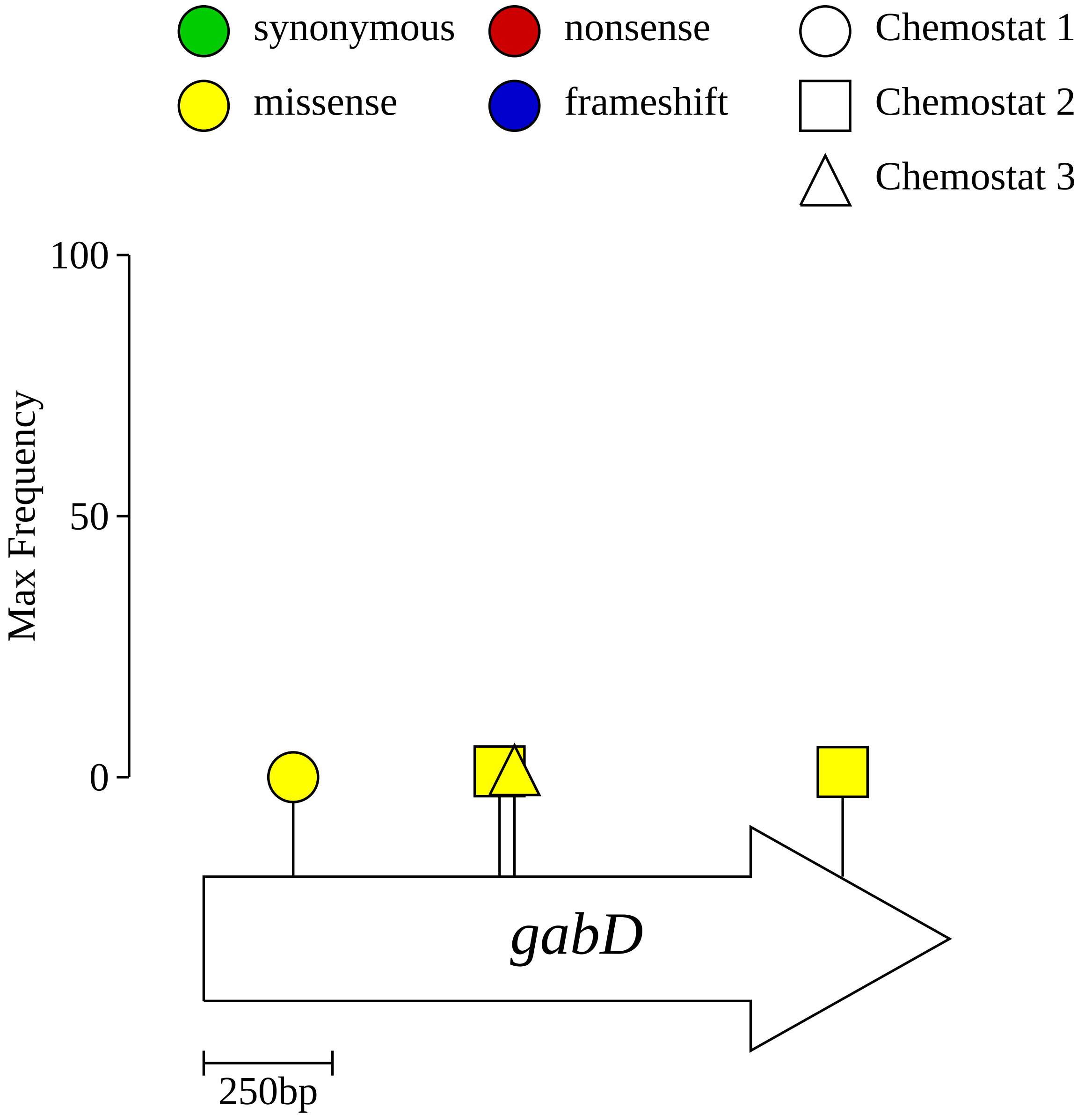
synonymous
missense
nonsense
frameshift
Chemostat 1
Chemostat 2
Chemostat 3
100
50
0
Max Frequency
gabD
250bp

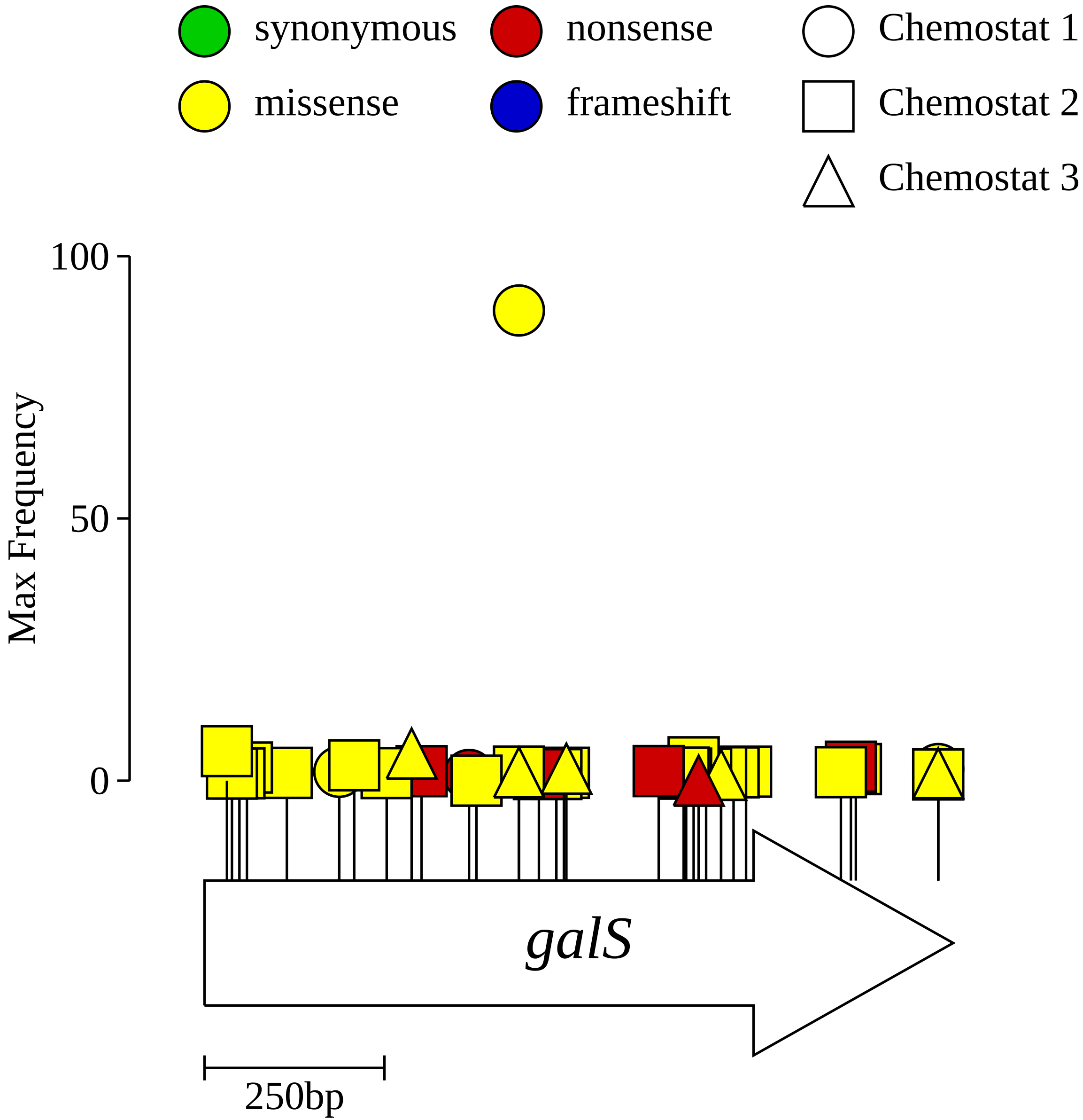

synonymous
nonsense
Chemostat 1
missense
frameshift
Chemostat 2
Chemostat 3
100
50
0
Max Frequency
galS
250bp

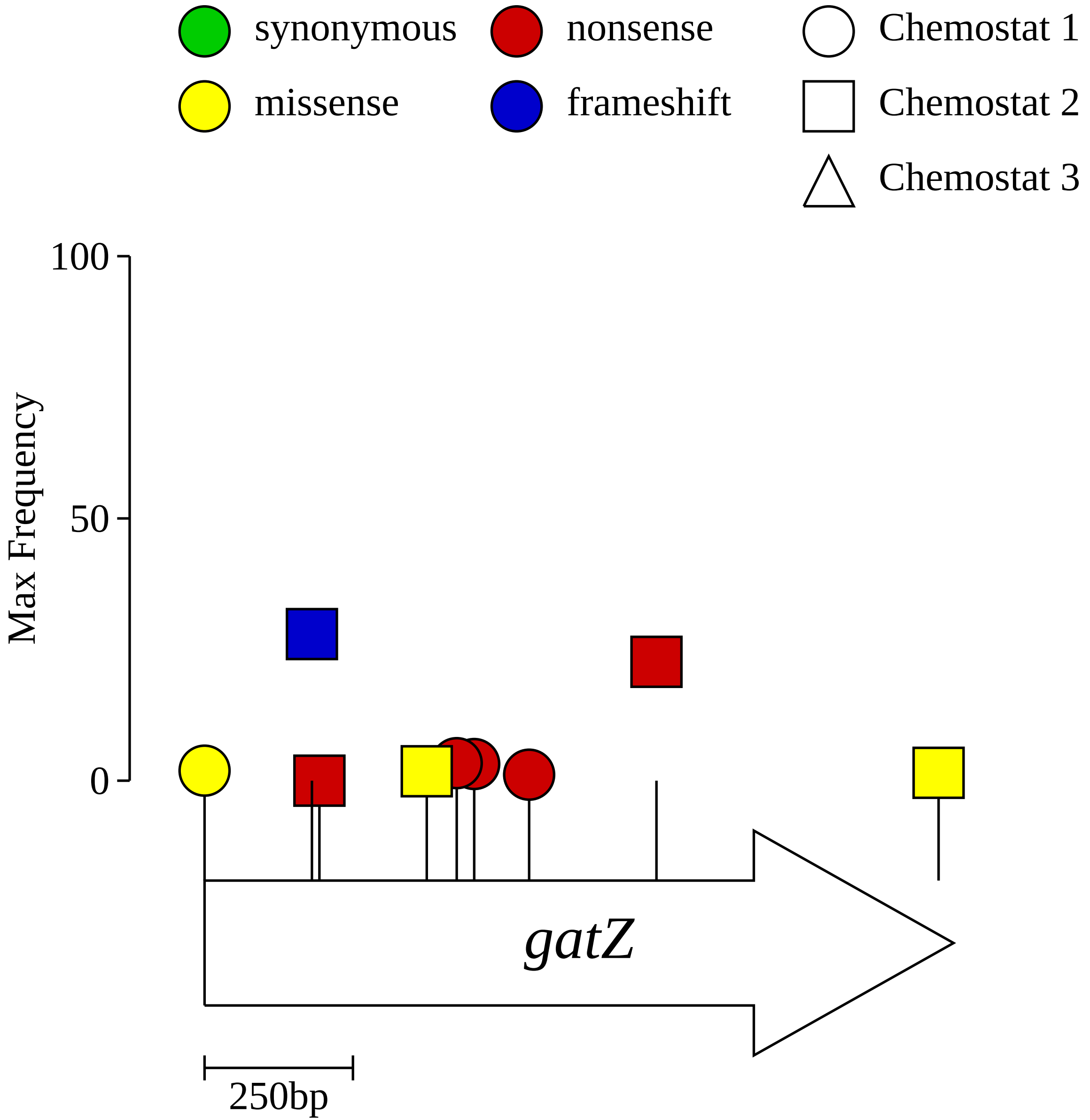
synonymous
nonsense
Chemostat 1
missense
frameshift
Chemostat 2
Chemostat 3
100
50
0
Max Frequency
gatZ
250bp

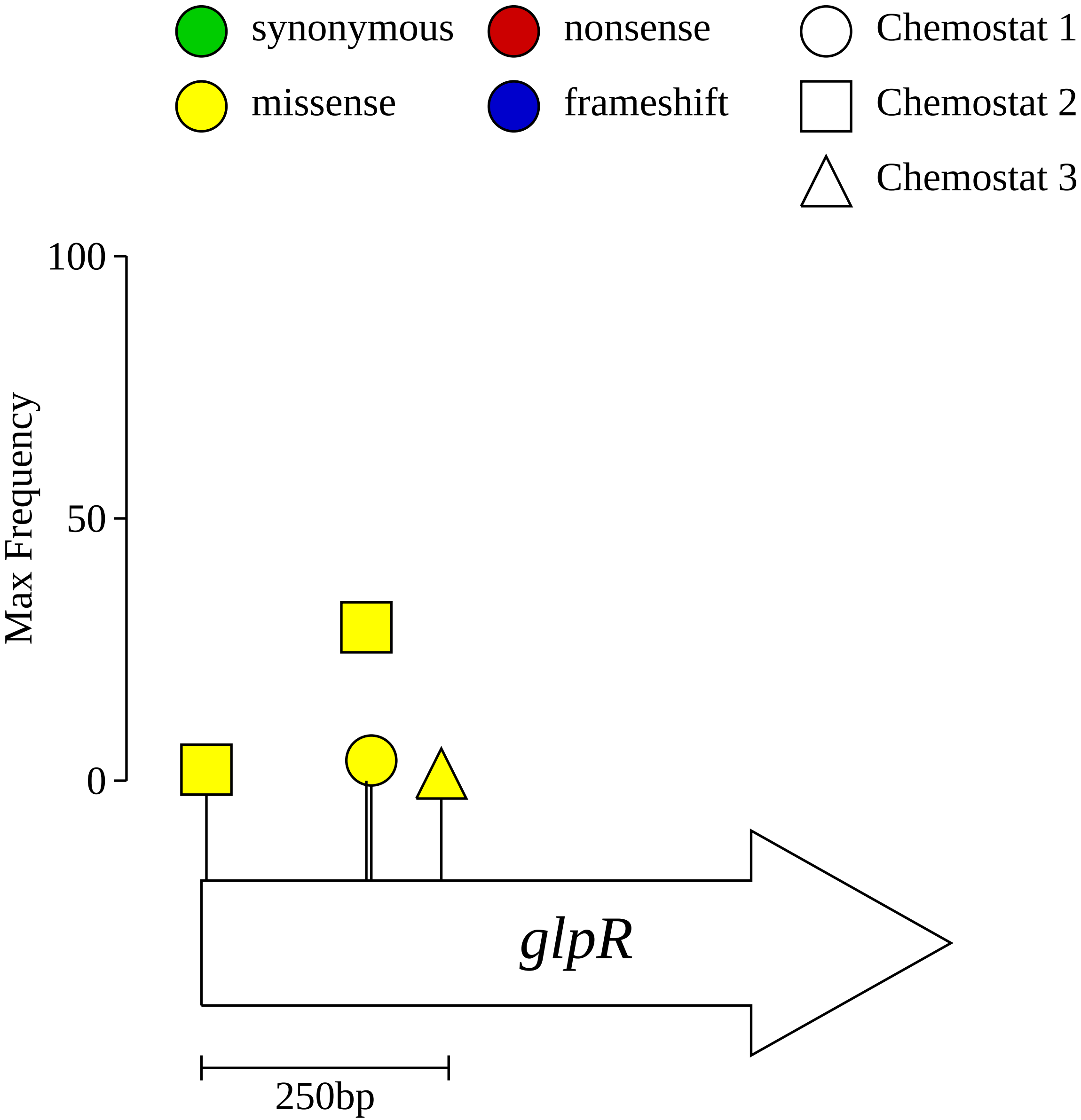

synonymous
missense
nonsense
frameshift
Chemostat 1
Chemostat 2
Chemostat 3
100
50
0
Max Frequency
glpR
250bp

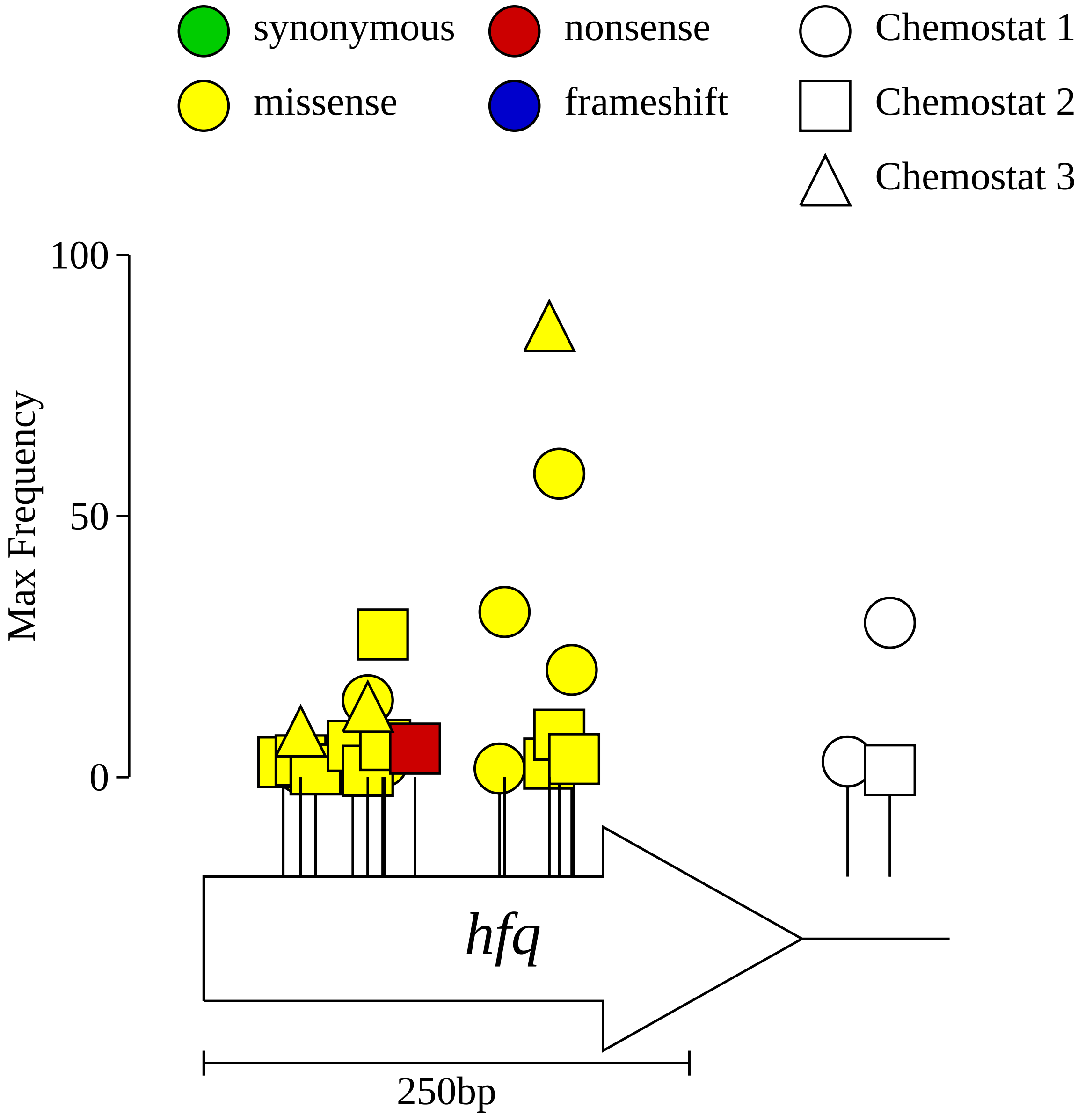
synonymous
nonsense
missense
frameshift
Chemostat 1
Chemostat 2
Chemostat 3
100
50
0
Max Frequency
hfq
250bp

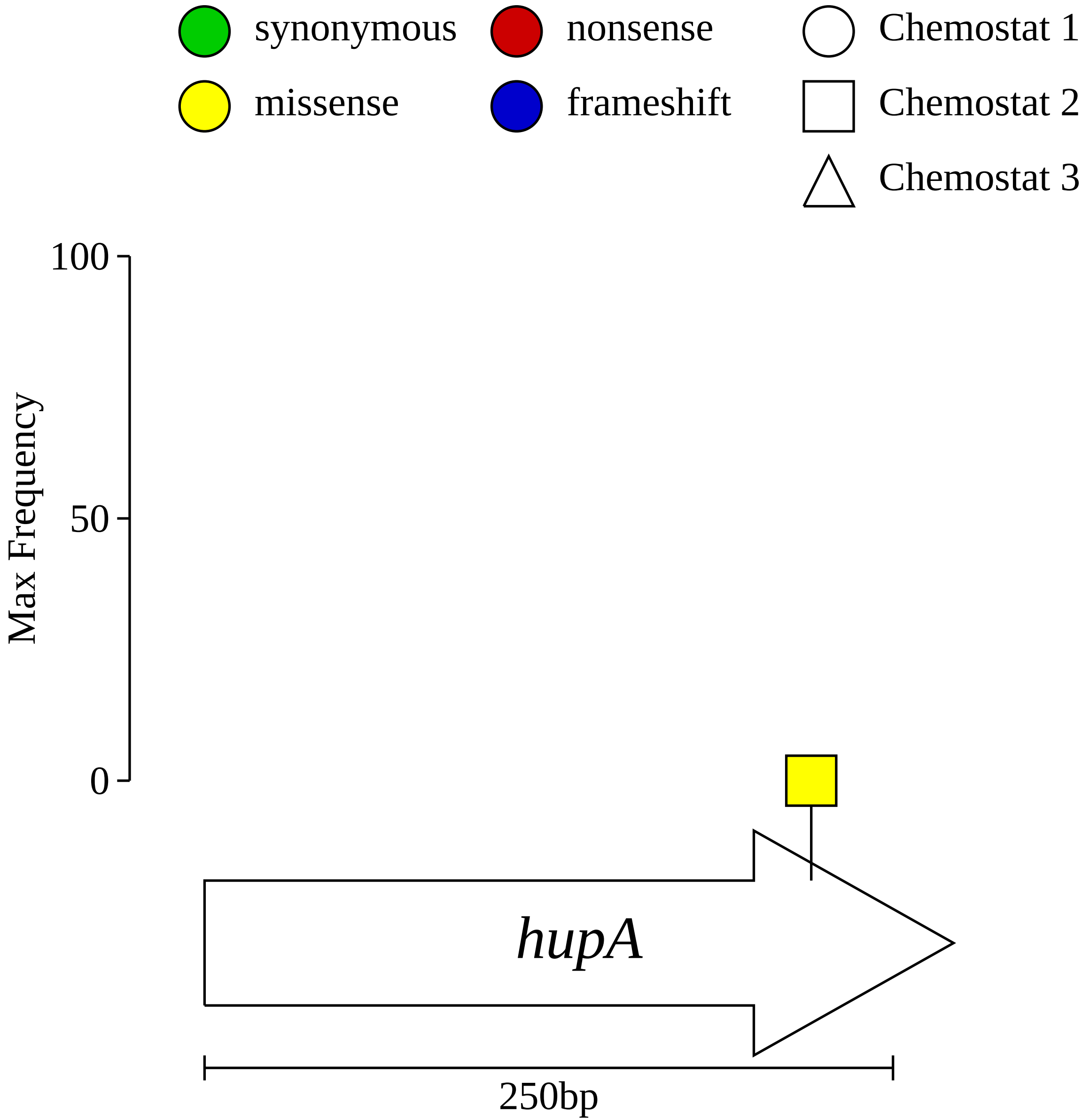
synonymous
nonsense
Chemostat 1
missense
frameshift
Chemostat 2
Chemostat 3
100
50
0
Max Frequency
hupA
250bp

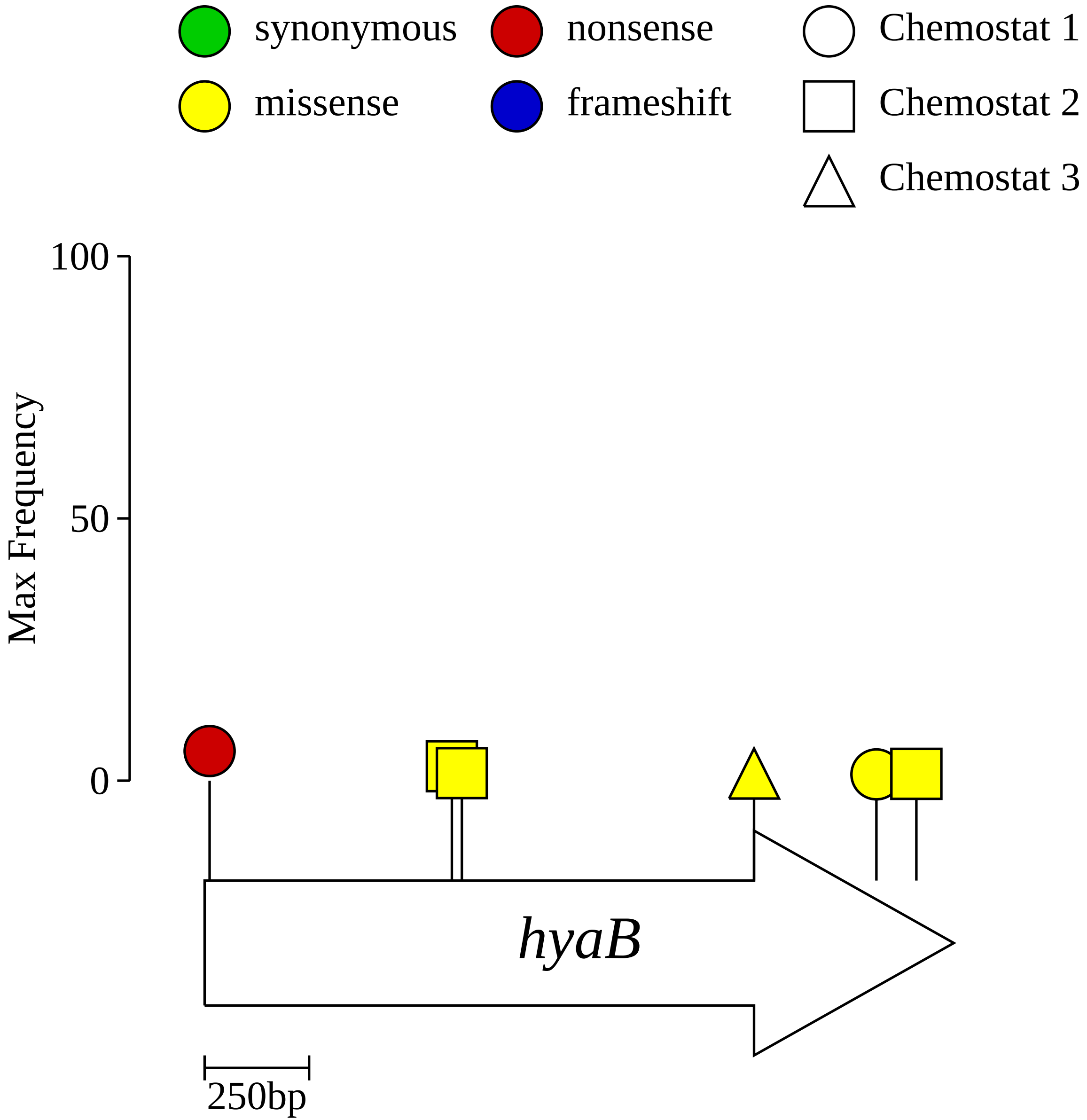
synonymous
nonsense
Chemostat 1
missense
frameshift
Chemostat 2
Chemostat 3
100
50
0
Max Frequency
hyaB
250bp

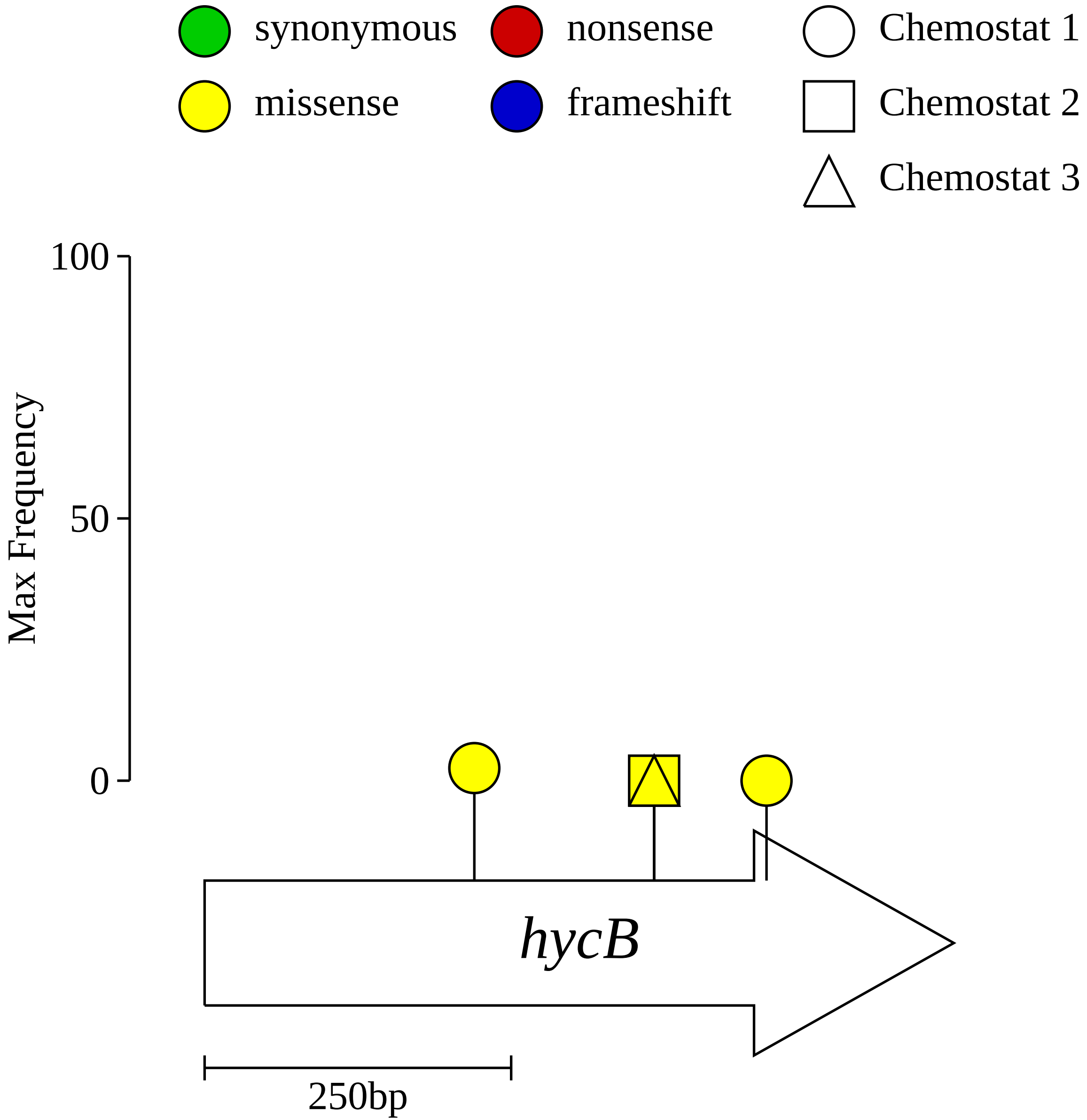
synonymous
nonsense
Chemostat 1
missense
frameshift
Chemostat 2
Chemostat 3
100
50
0
Max Frequency
hycB
250bp

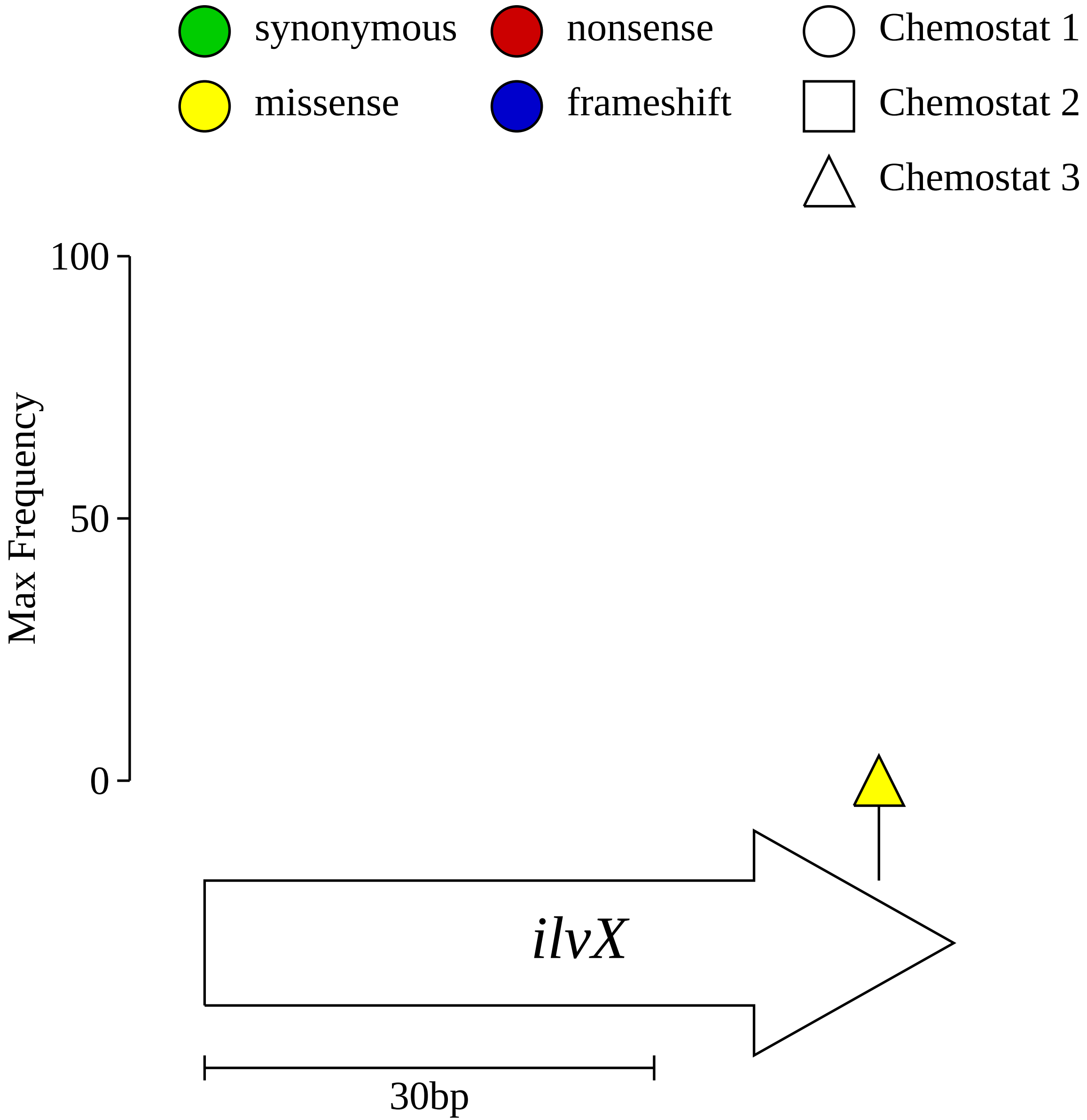

synonymous
nonsense
Chemostat 1
missense
frameshift
Chemostat 2
Chemostat 3
100
50
0
Max Frequency
ilvX
30bp

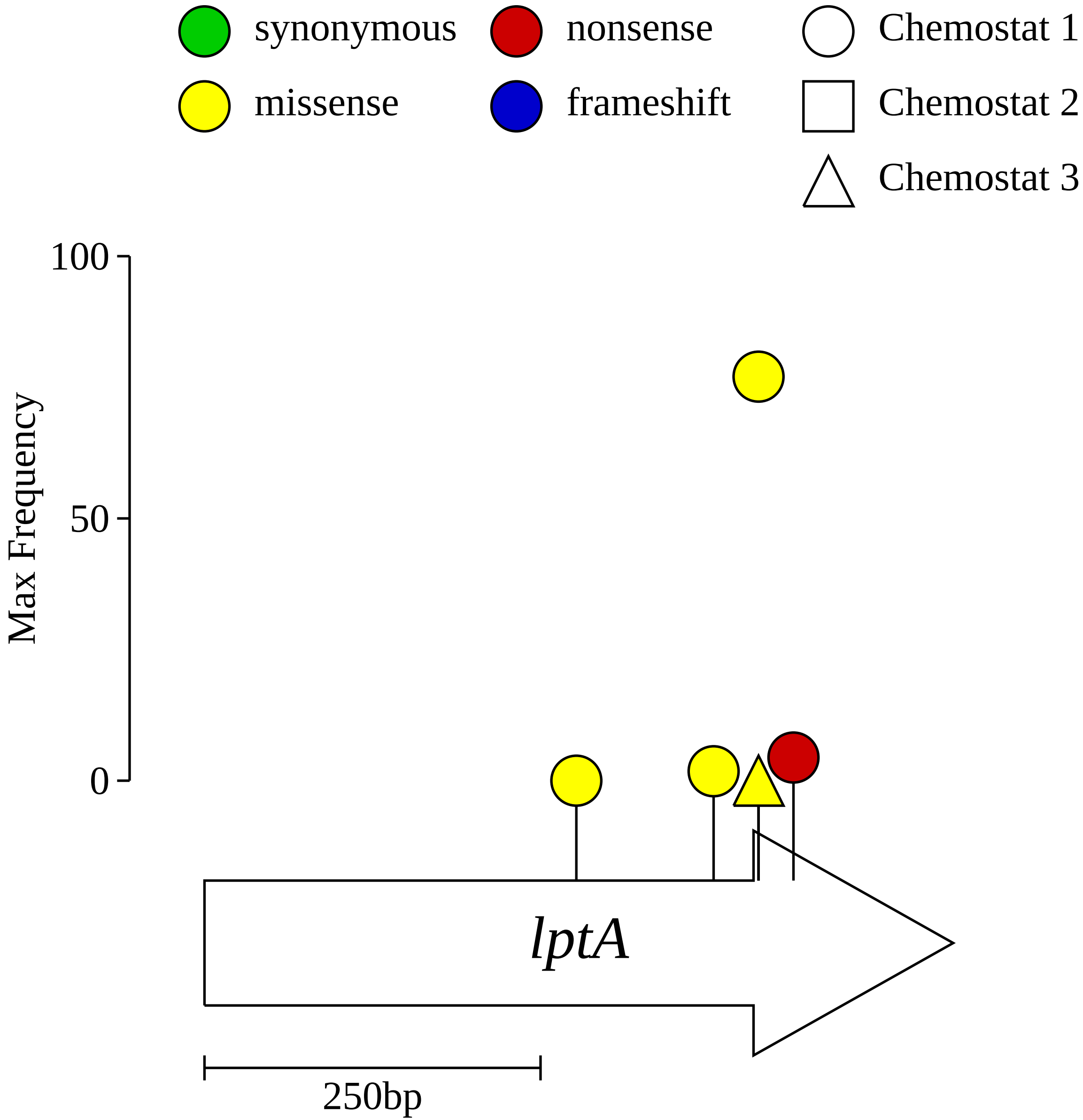
synonymous
nonsense
Chemostat 1
missense
frameshift
Chemostat 2
Chemostat 3
100
50
0
Max Frequency
lptA
250bp

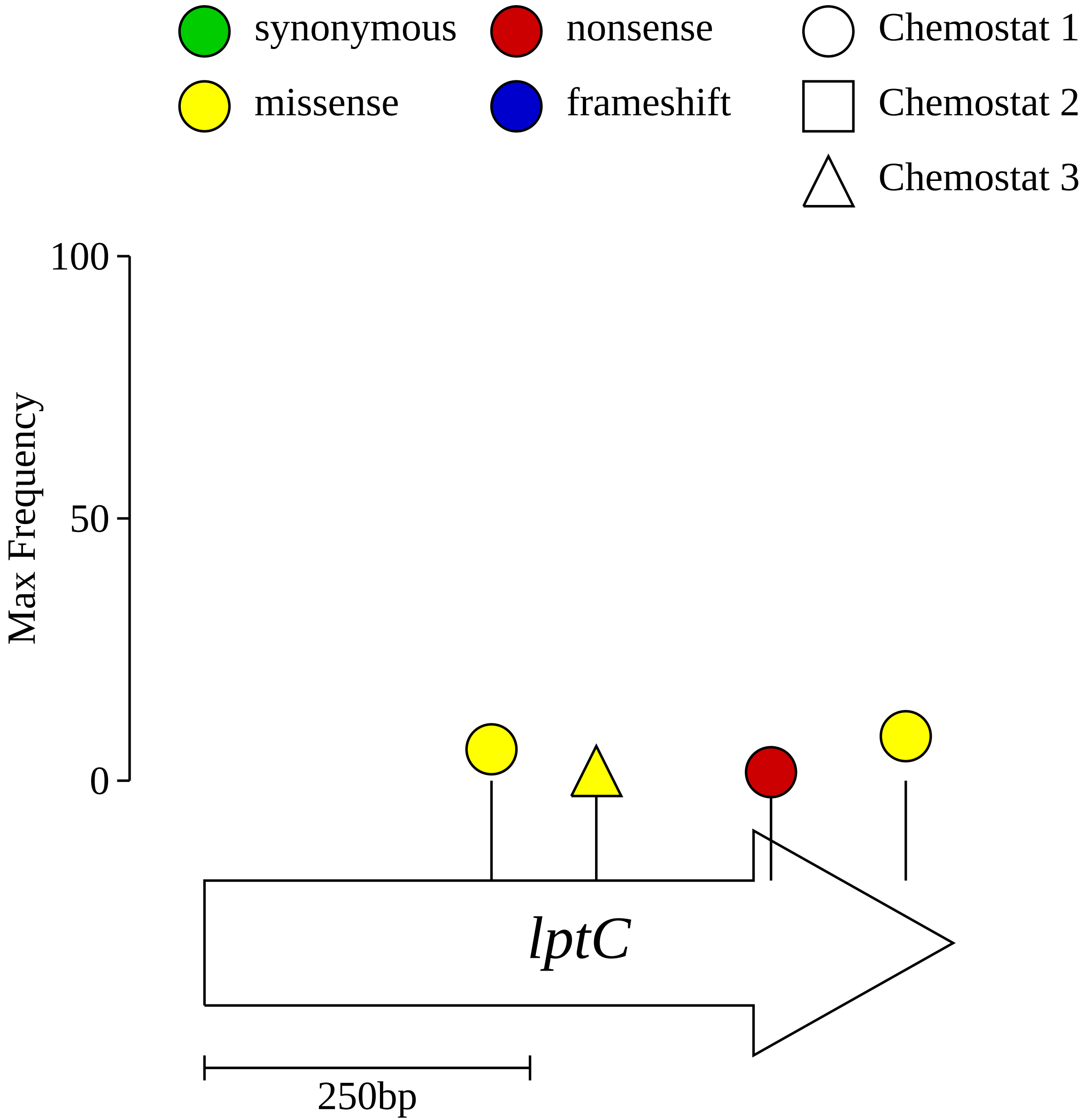
synonymous
nonsense
Chemostat 1
missense
frameshift
Chemostat 2
Chemostat 3
100
50
0
Max Frequency
lptC
250bp

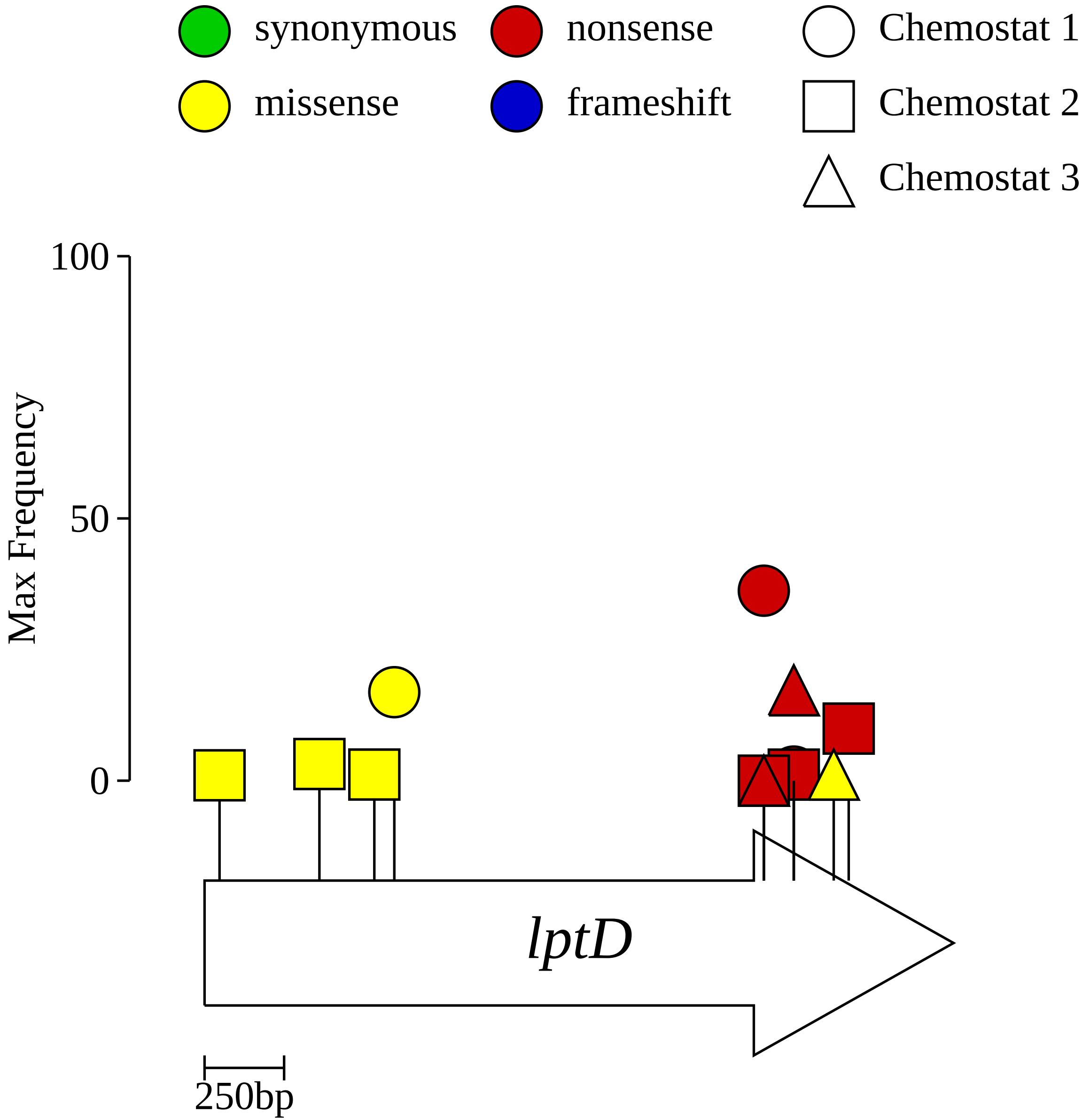

synonymous
nonsense
Chemostat 1
missense
frameshift
Chemostat 2
Chemostat 3
100
50
0
Max Frequency
lptD
250bp

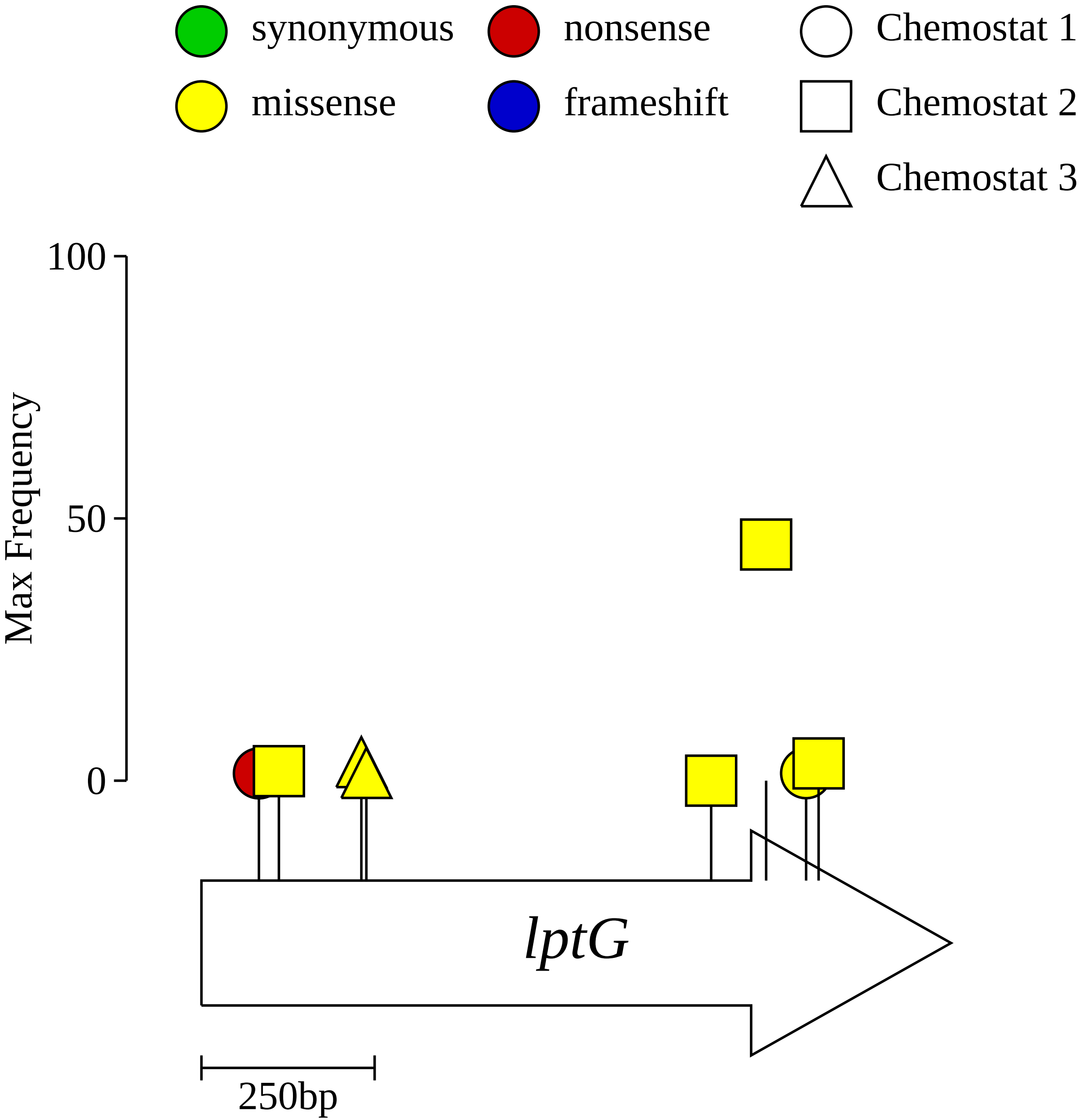
synonymous
nonsense
Chemostat 1
missense
frameshift
Chemostat 2
Chemostat 3
100
50
0
Max Frequency
lptG
250bp

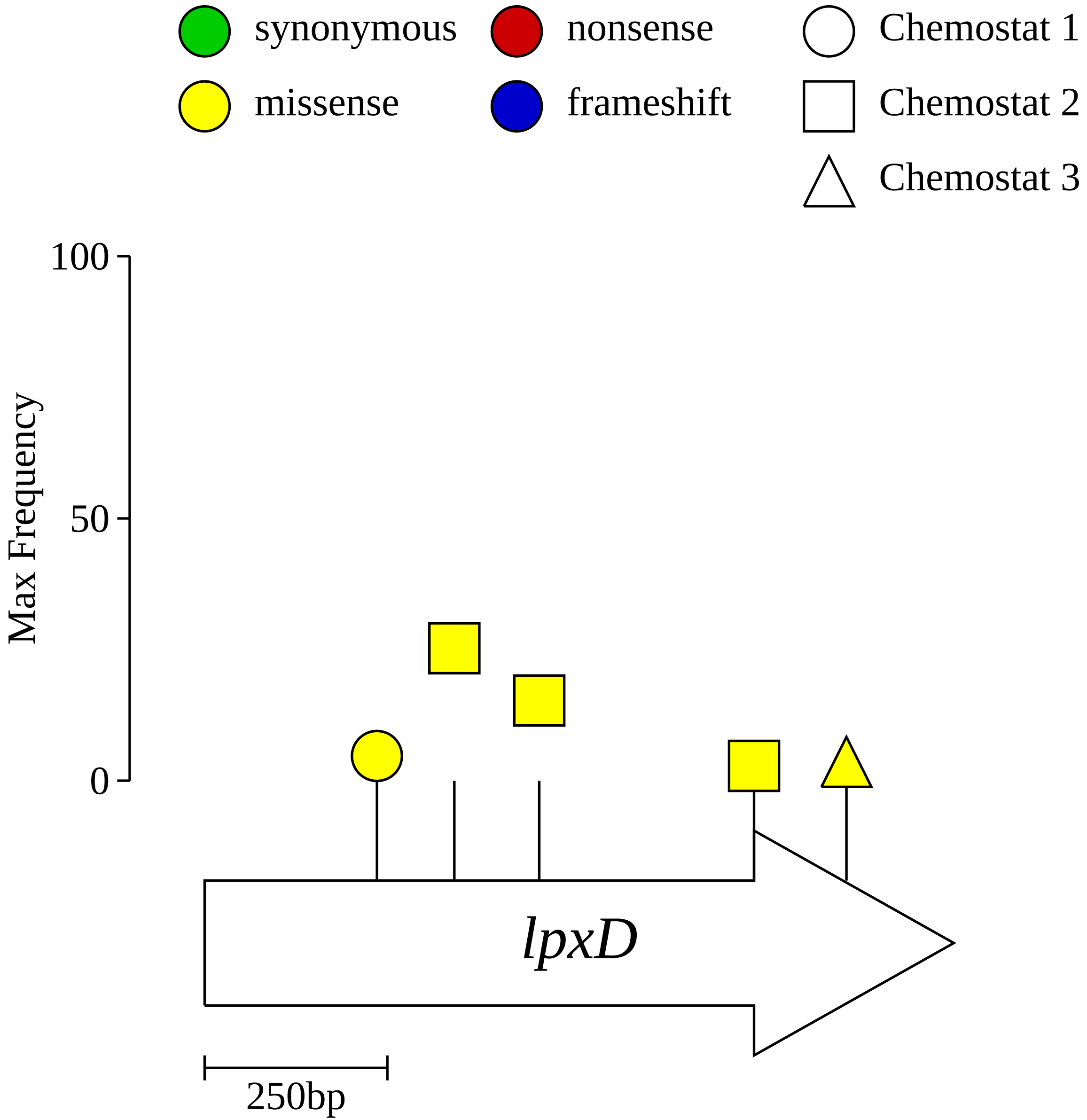
synonymous
missense
nonsense
frameshift
Chemostat 1
Chemostat 2
Chemostat 3
100
50
0
Max Frequency
lpxD
250bp

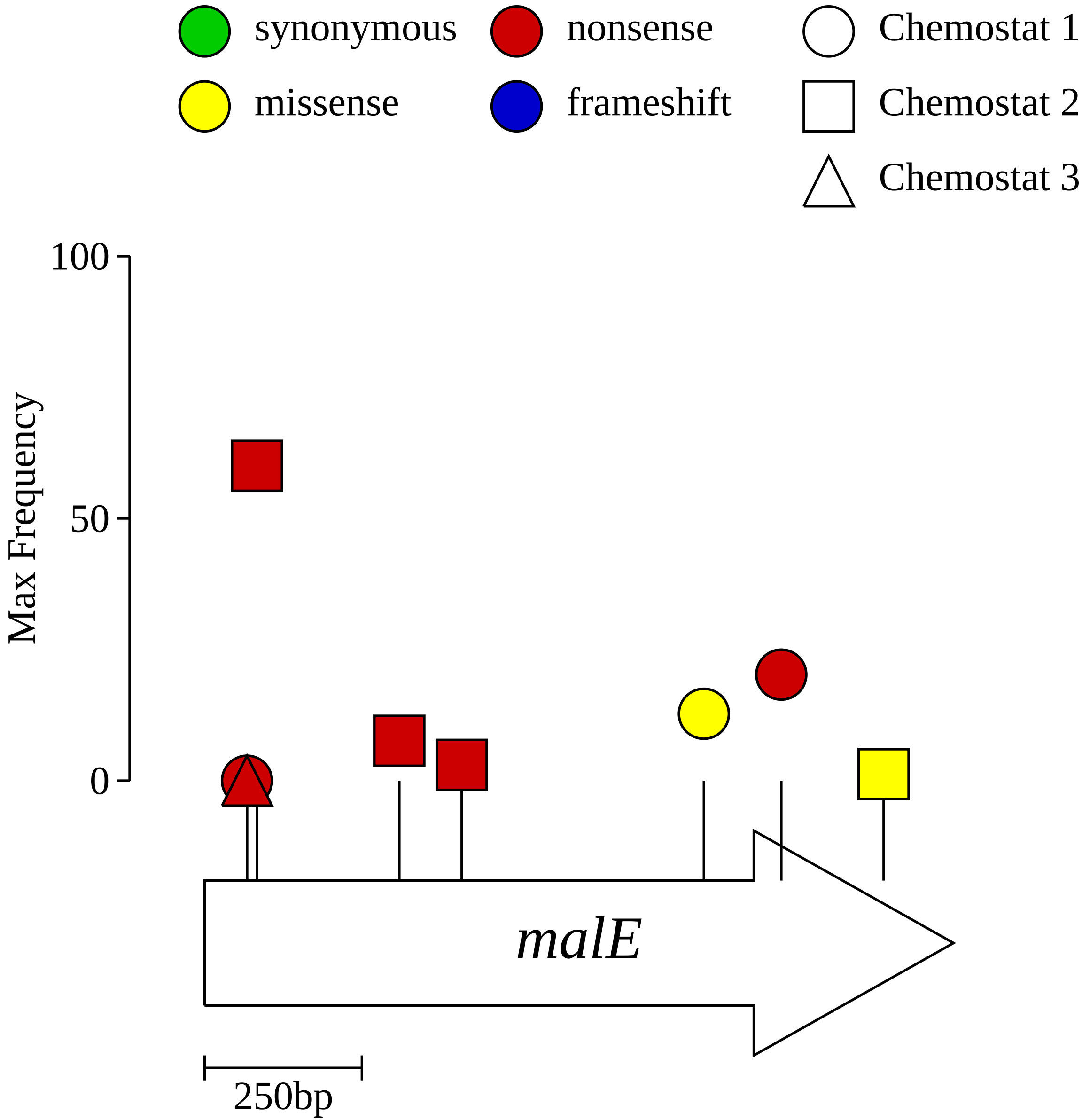
synonymous
nonsense
Chemostat 1
missense
frameshift
Chemostat 2
Chemostat 3
100
50
0
Max Frequency
malE
250bp

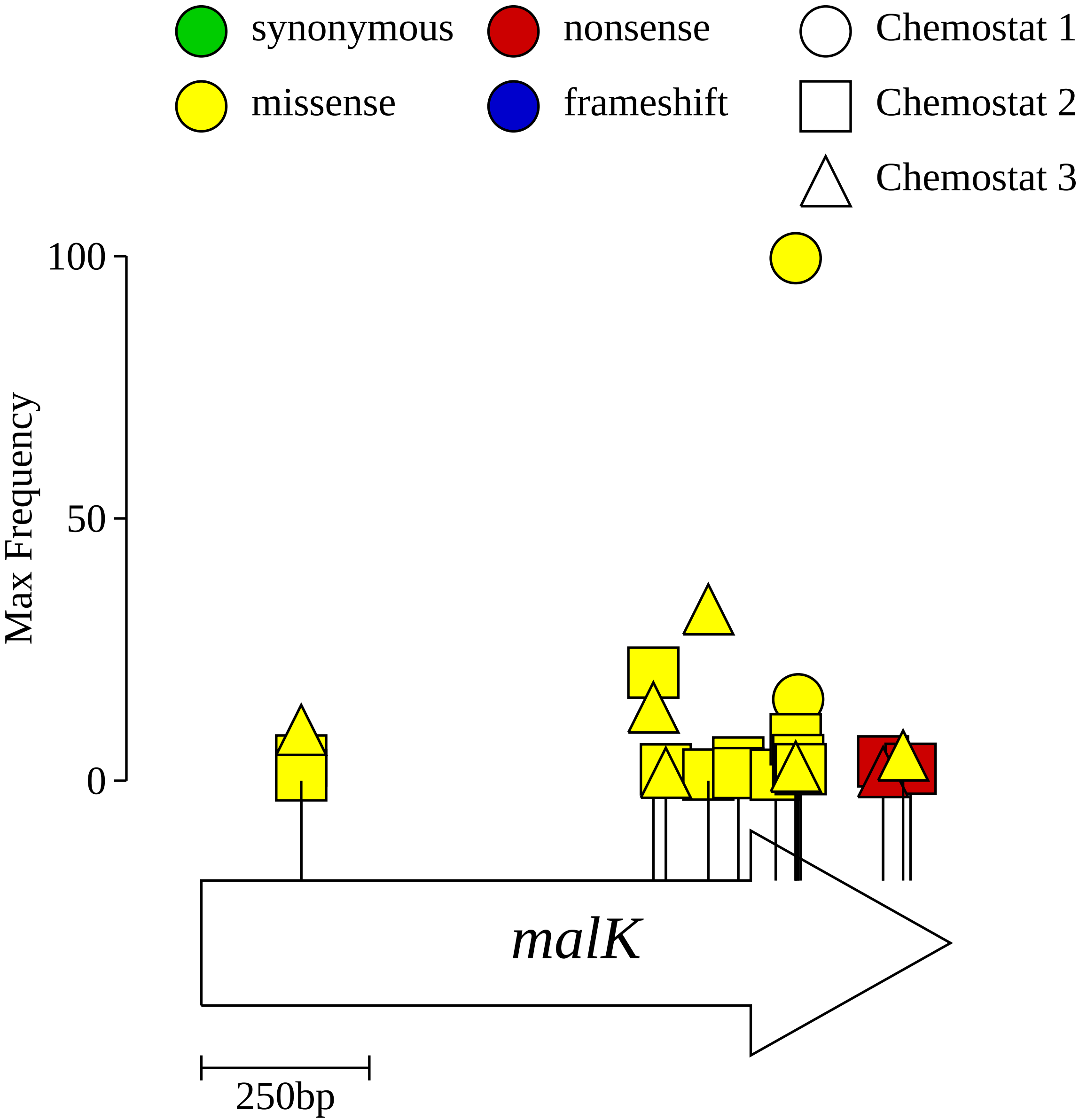

synonymous
missense
nonsense
frameshift
Chemostat 1
Chemostat 2
Chemostat 3
Max Frequency
100
50
0
malK
250bp

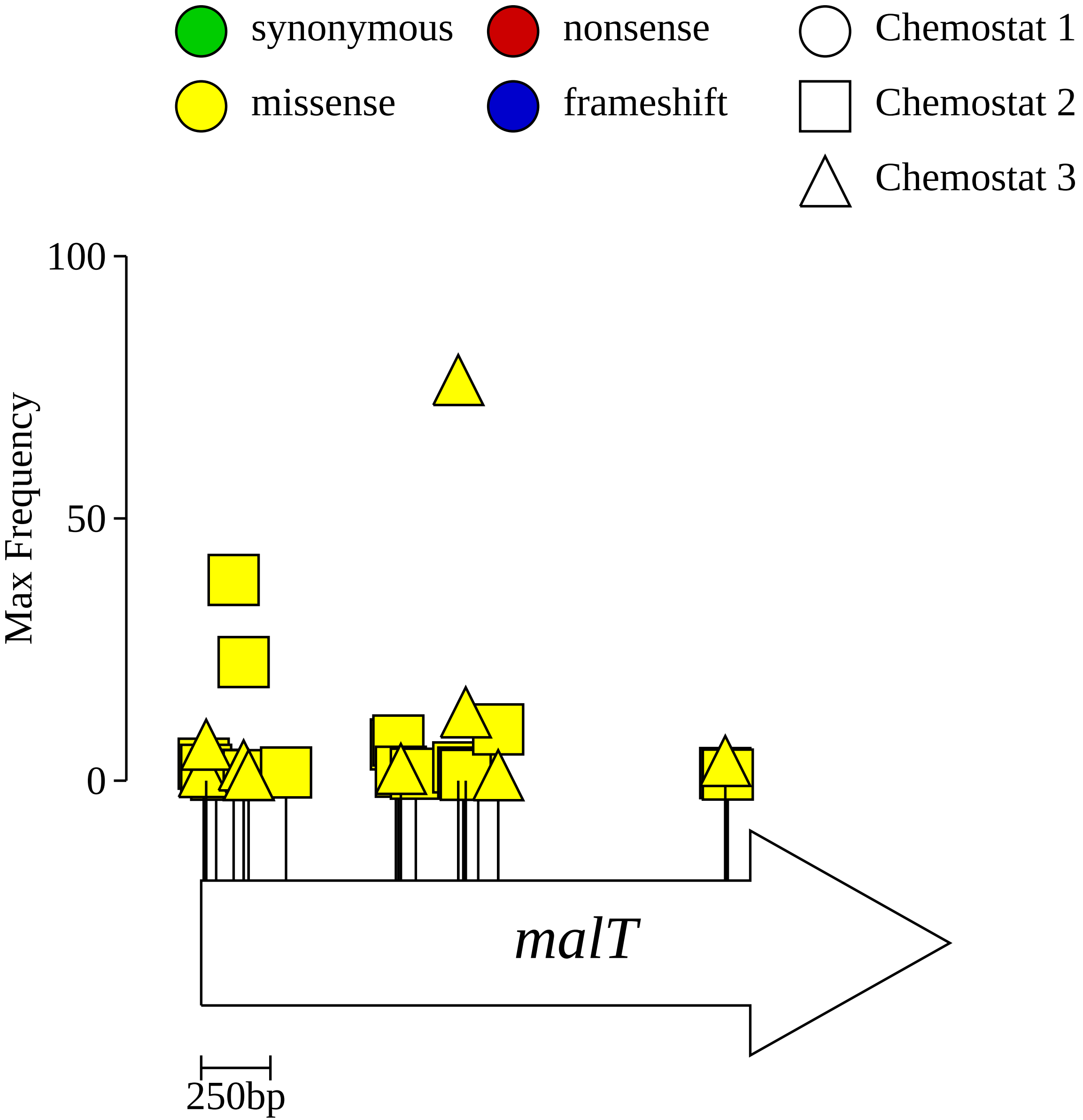
synonymous
nonsense
Chemostat 1
missense
frameshift
Chemostat 2
Chemostat 3
100
50
0
Max Frequency
malT
250bp

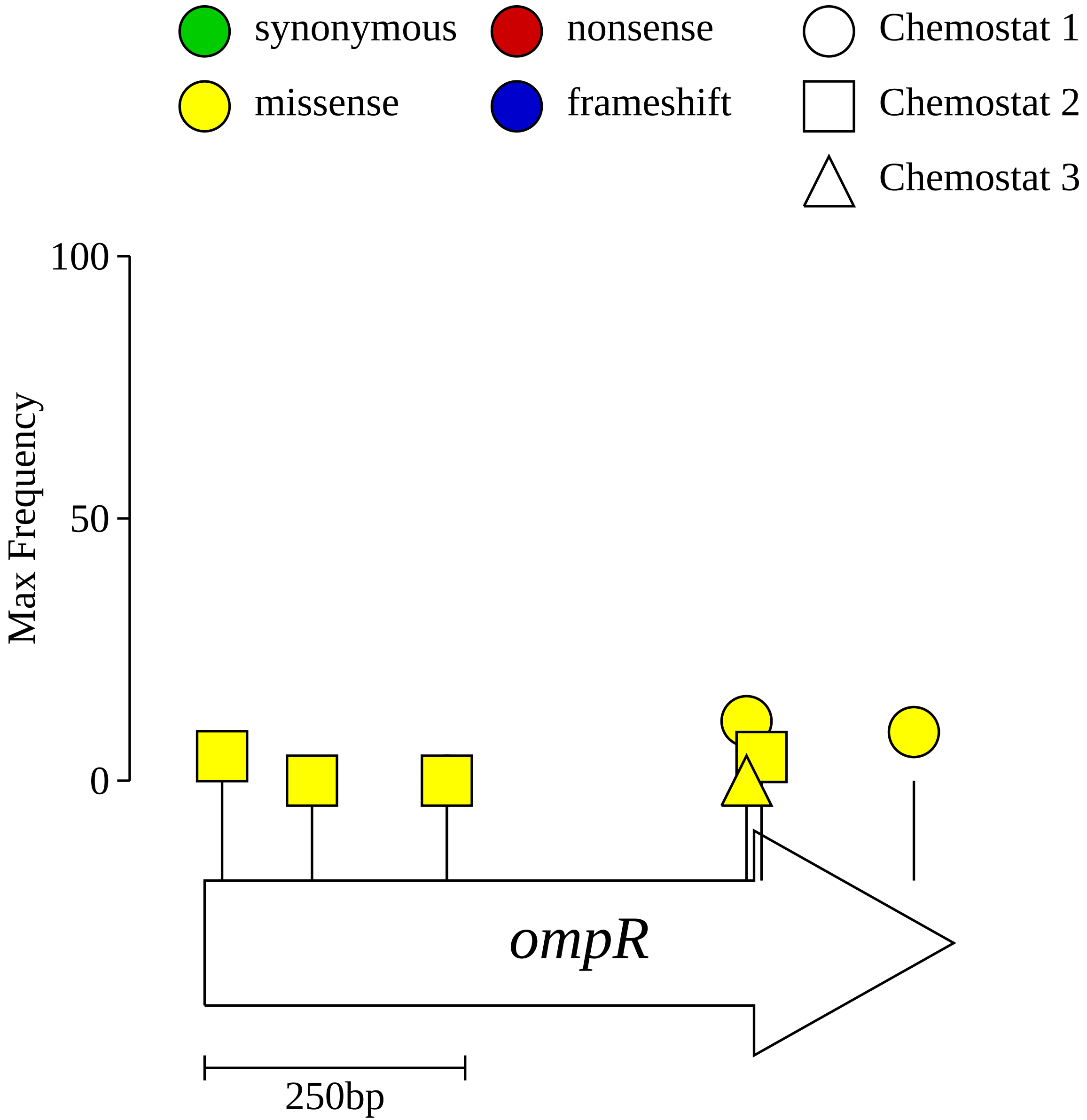
synonymous
missense
nonsense
frameshift
Chemostat 1
Chemostat 2
Chemostat 3
Max Frequency
100
50
0
ompR
250bp

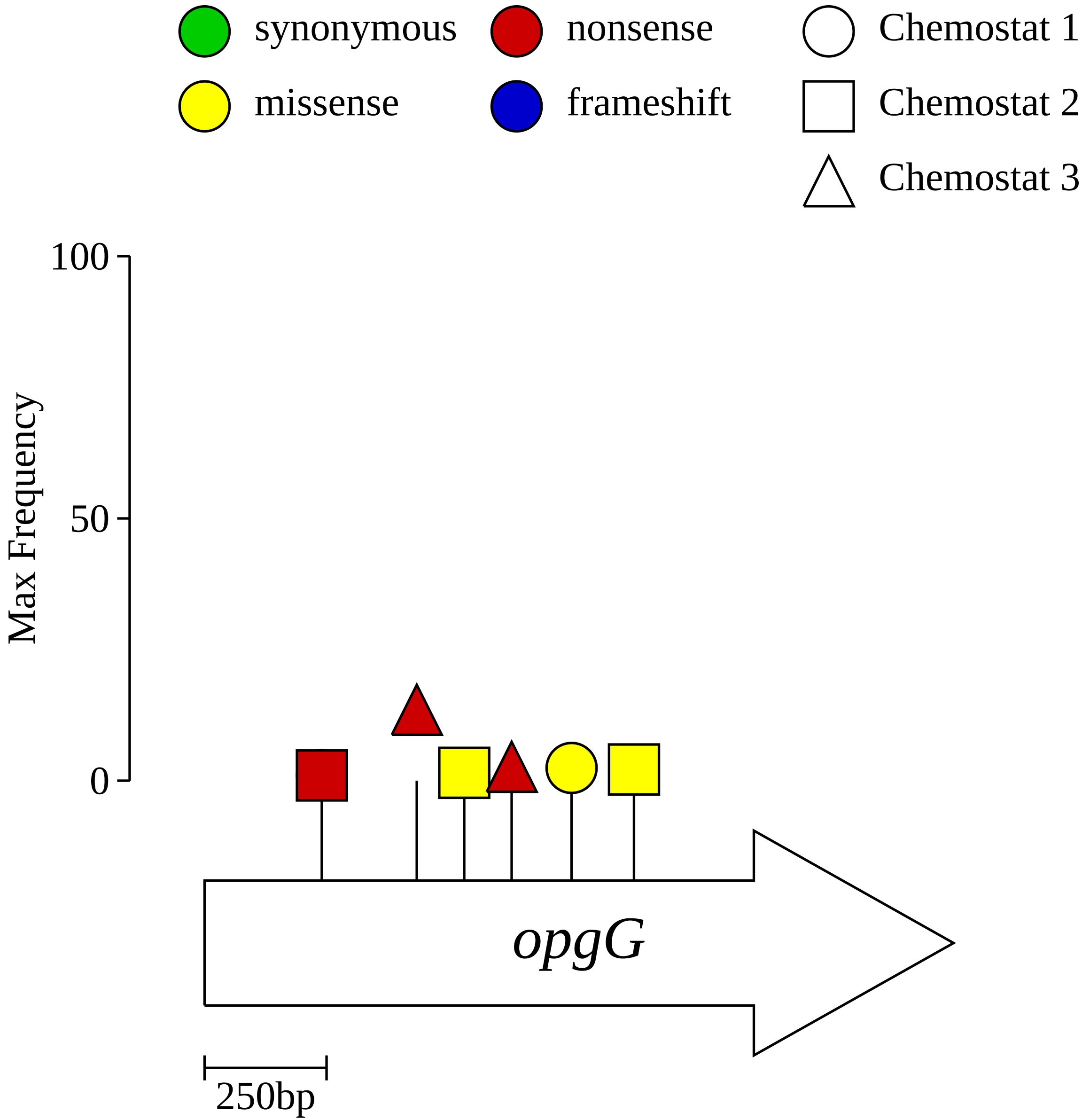

synonymous
nonsense
Chemostat 1
missense
frameshift
Chemostat 2
Chemostat 3
100
50
0
Max Frequency
opgG
250bp

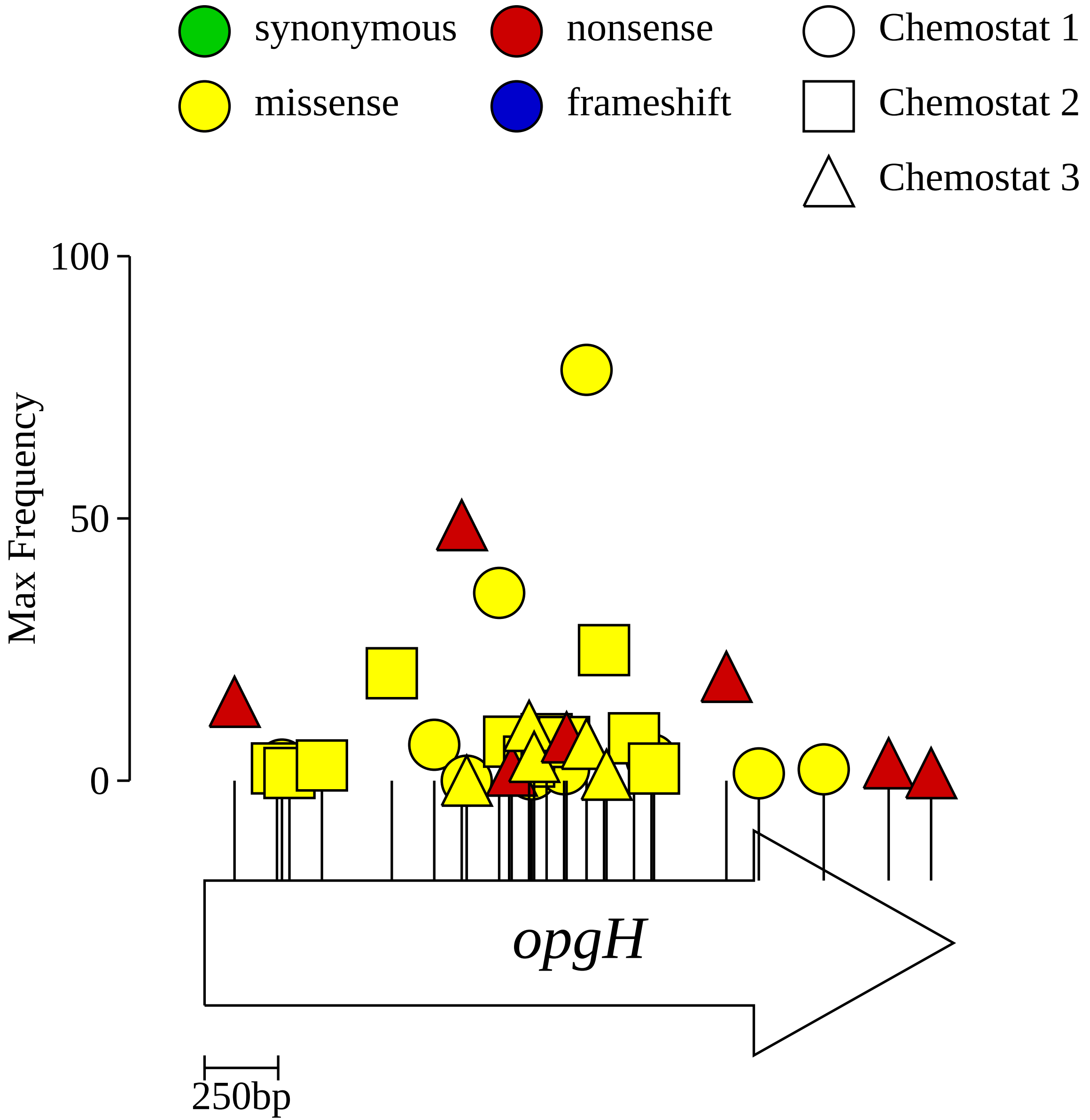

synonymous
nonsense
Chemostat 1
missense
frameshift
Chemostat 2
Chemostat 3
100
50
0
Max Frequency
opgH
250bp

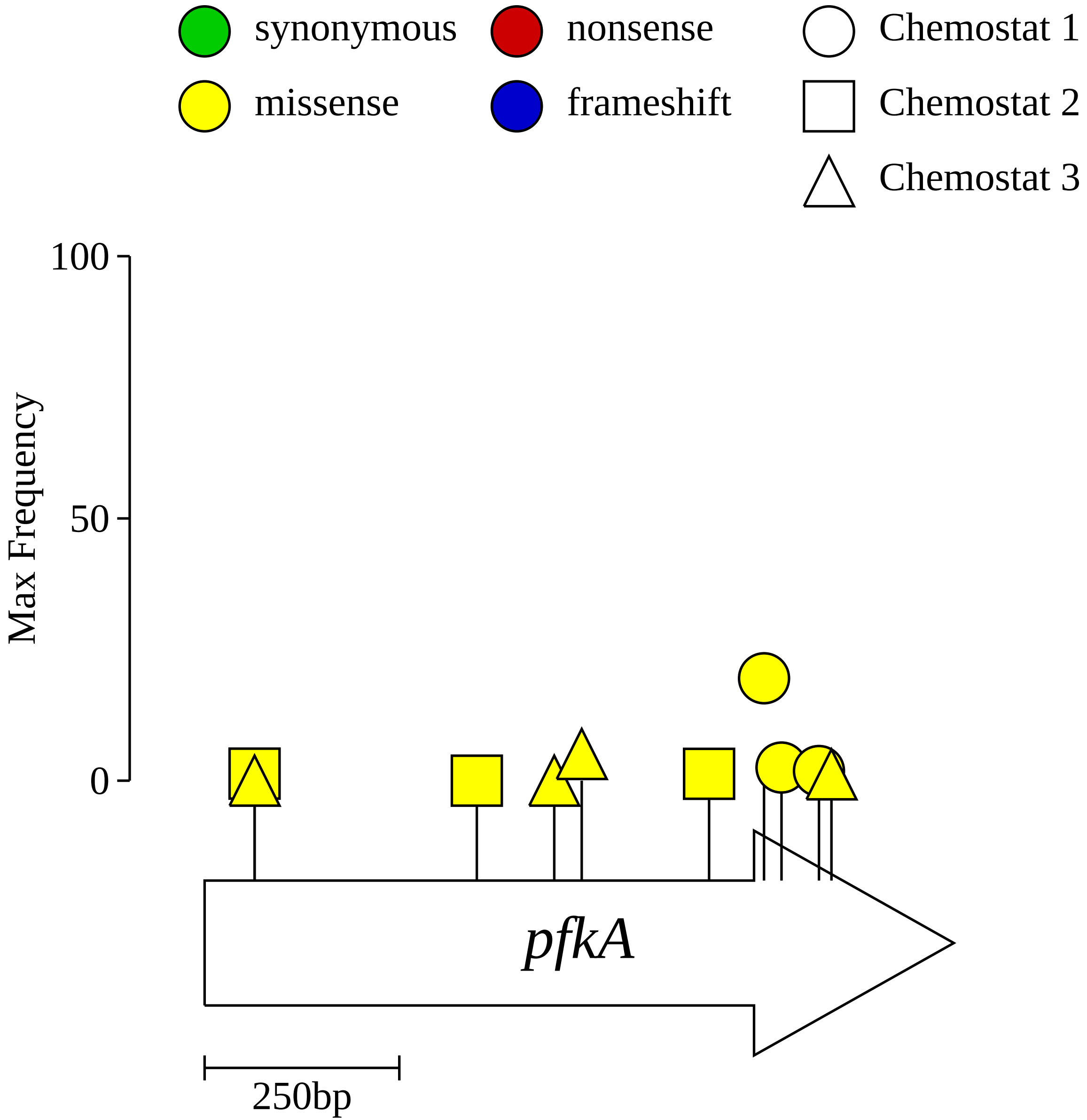
synonymous
nonsense
Chemostat 1
missense
frameshift
Chemostat 2
Chemostat 3
100
50
0
Max Frequency
pfkA
250bp

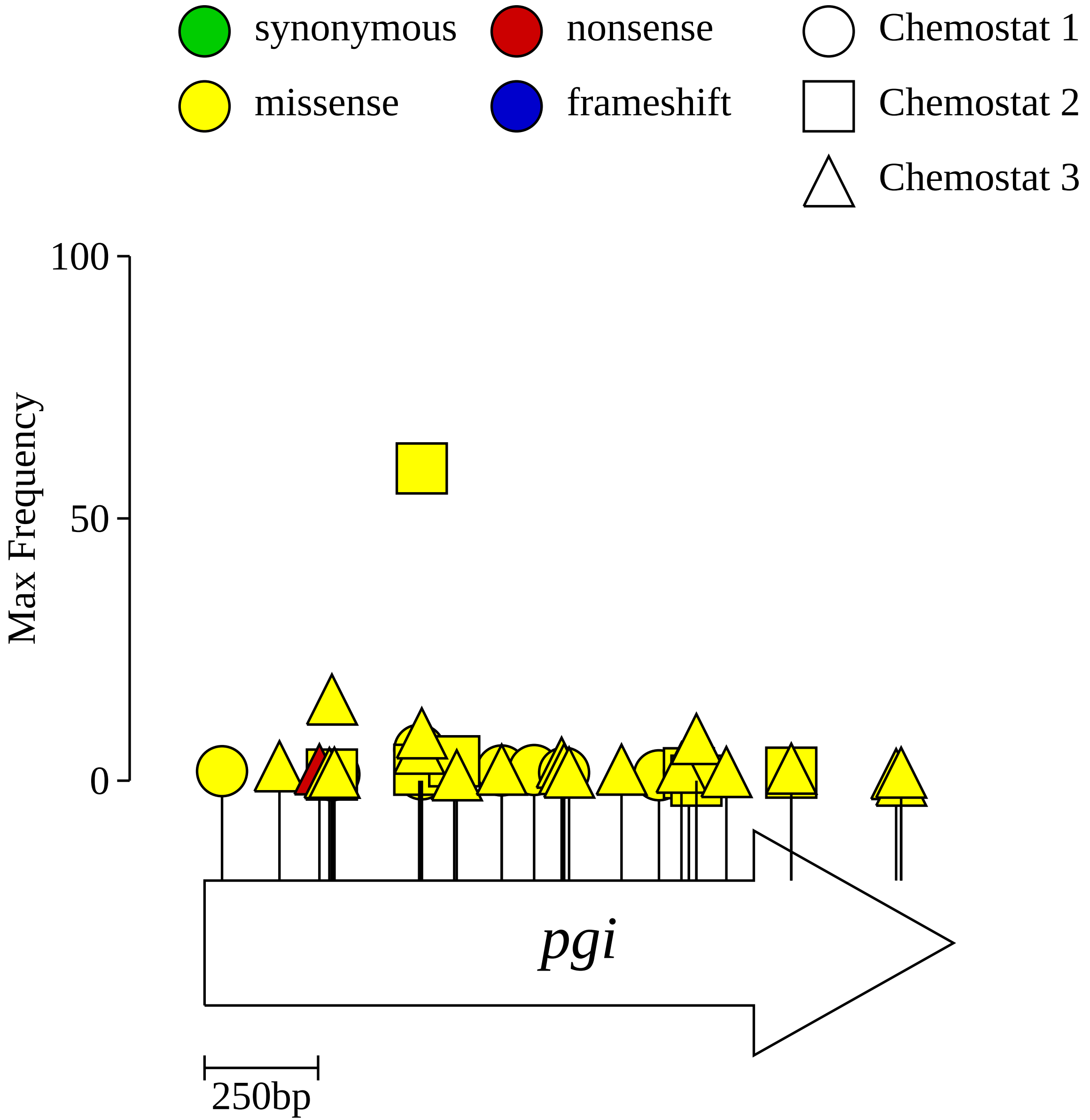
synonymous
nonsense
Chemostat 1
missense
frameshift
Chemostat 2
Chemostat 3
100
50
0
Max Frequency
pgi
250bp

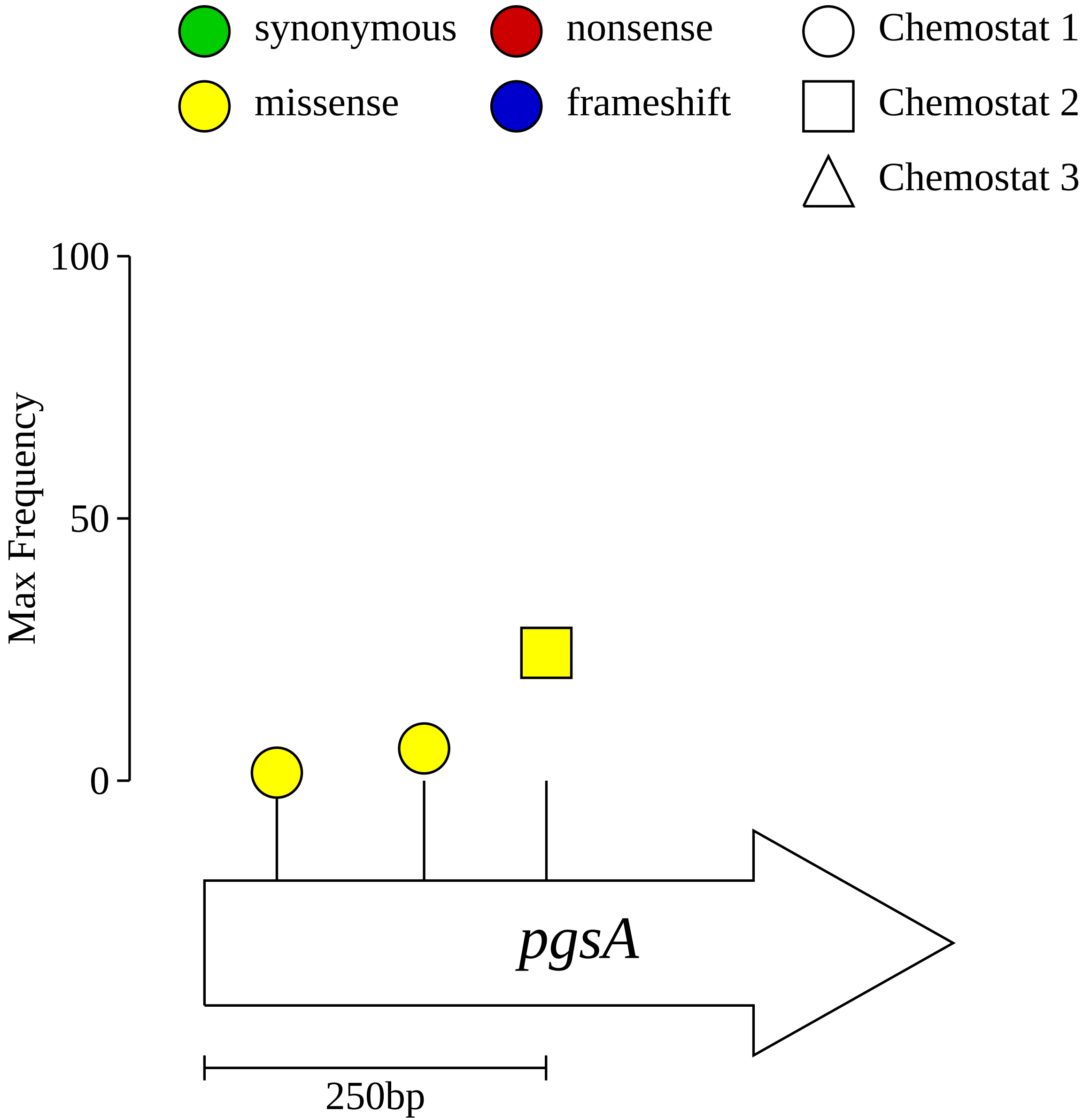

synonymous
nonsense
Chemostat 1
missense
frameshift
Chemostat 2
Chemostat 3
100
50
0
Max Frequency
pgsA
250bp

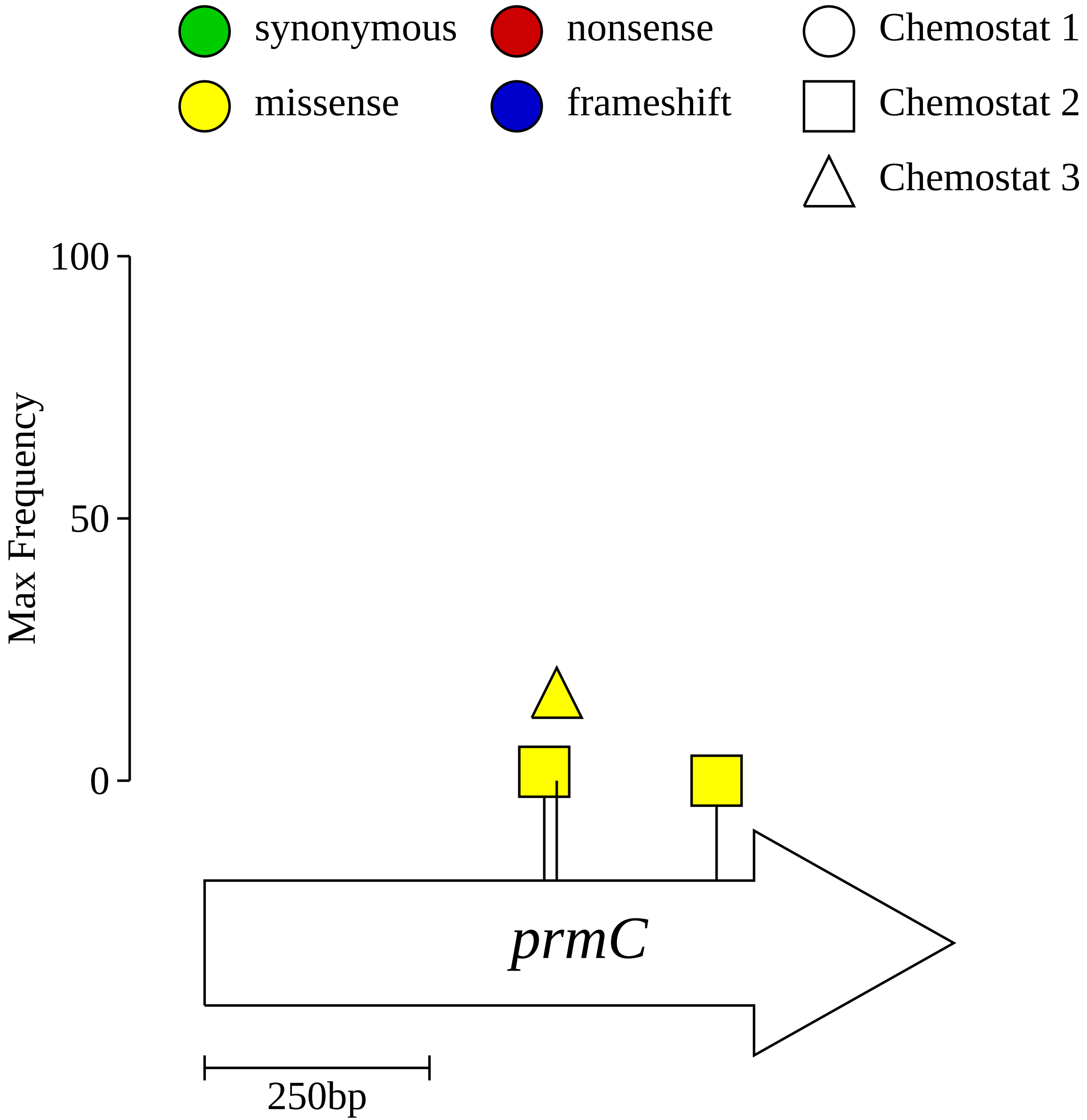

synonymous
nonsense
Chemostat 1
missense
frameshift
Chemostat 2
Chemostat 3
100
50
0
Max Frequency
prmC
250bp

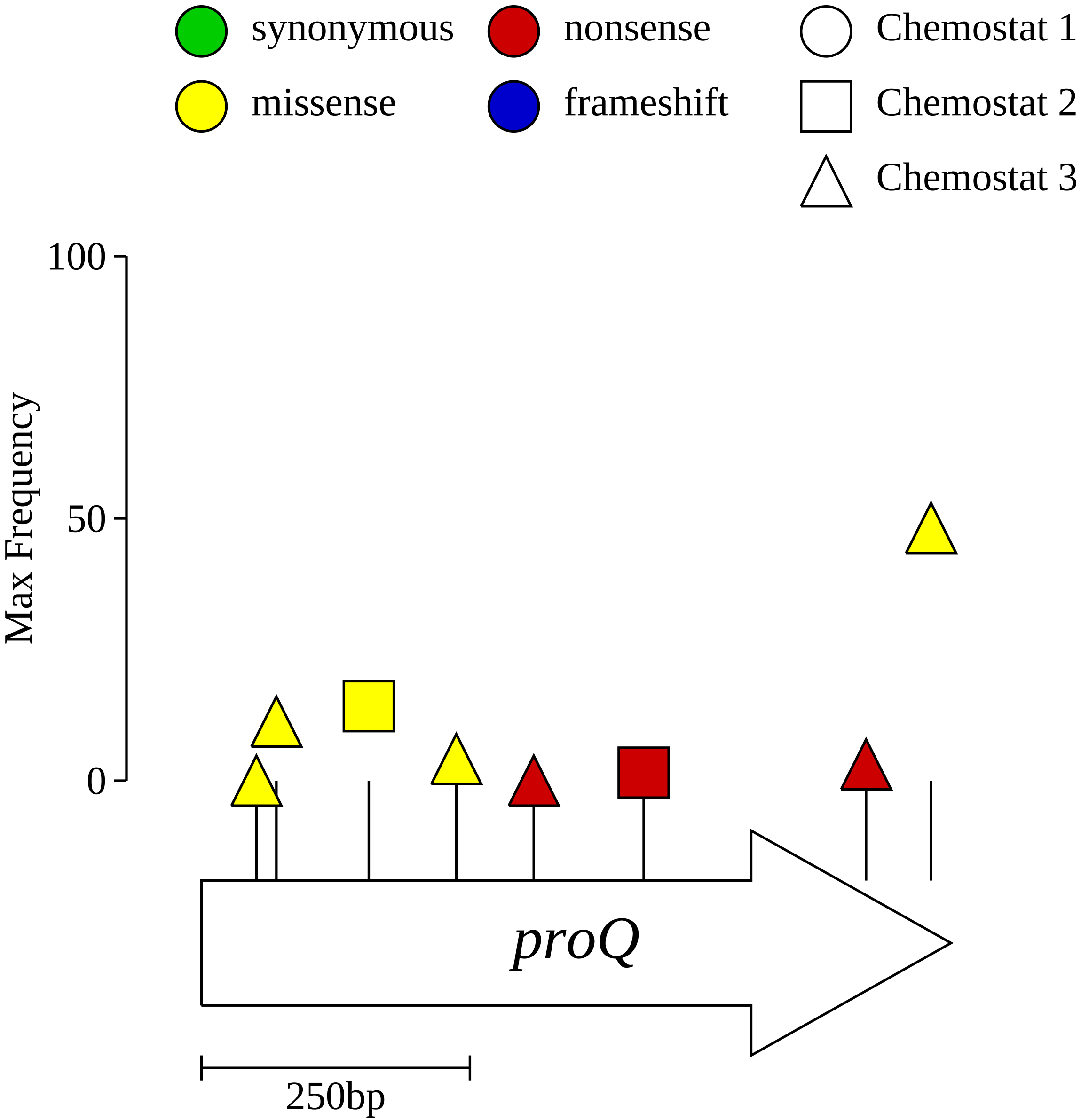
synonymous
missense
nonsense
frameshift
Chemostat 1
Chemostat 2
Chemostat 3
100
50
0
Max Frequency
proQ
250bp

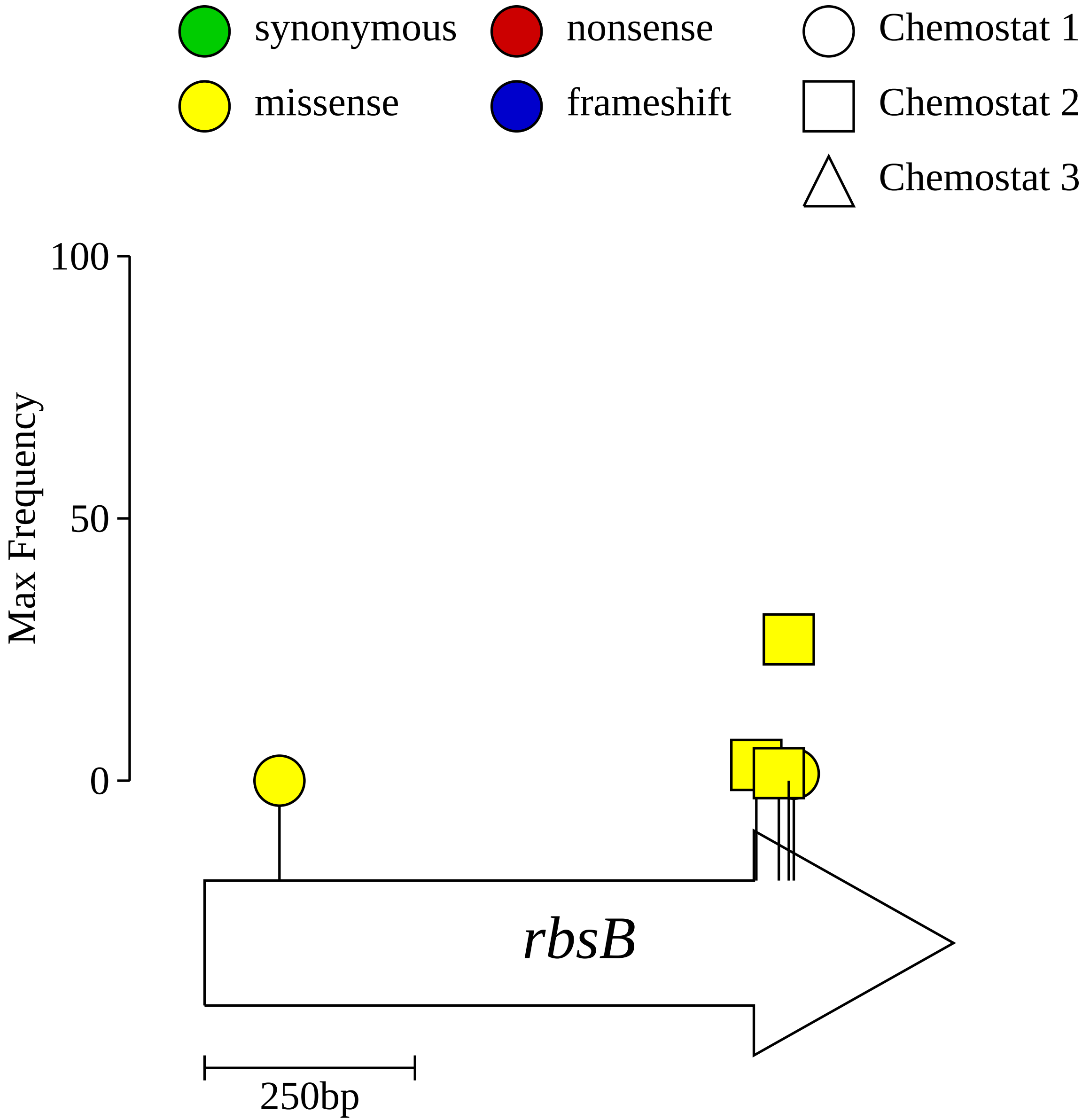
synonymous
nonsense
Chemostat 1
missense
frameshift
Chemostat 2
Chemostat 3
100
50
0
Max Frequency
rbsB
250bp

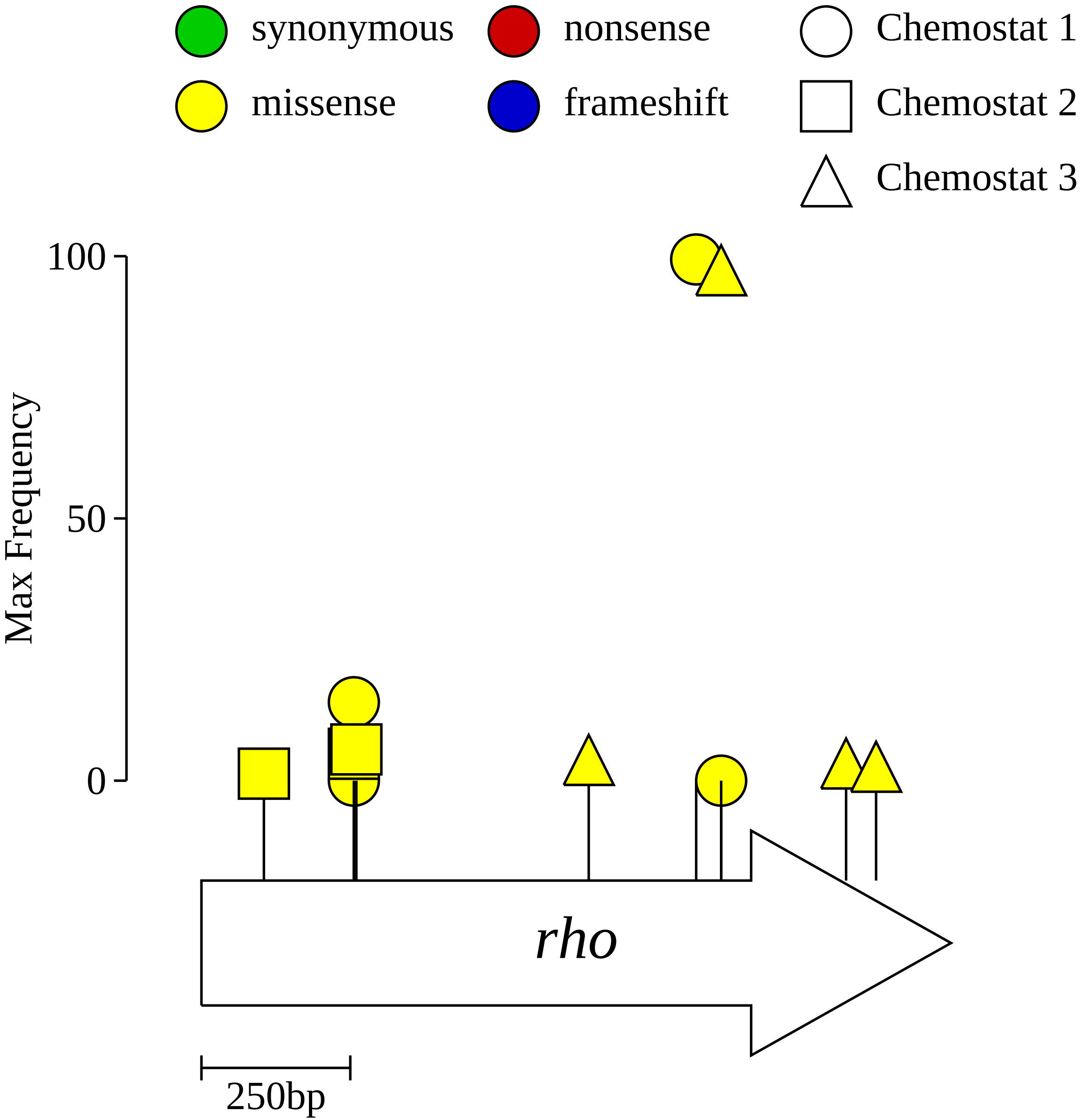
synonymous
nonsense
Chemostat 1
missense
frameshift
Chemostat 2
Chemostat 3
100
50
0
Max Frequency
rho
250bp

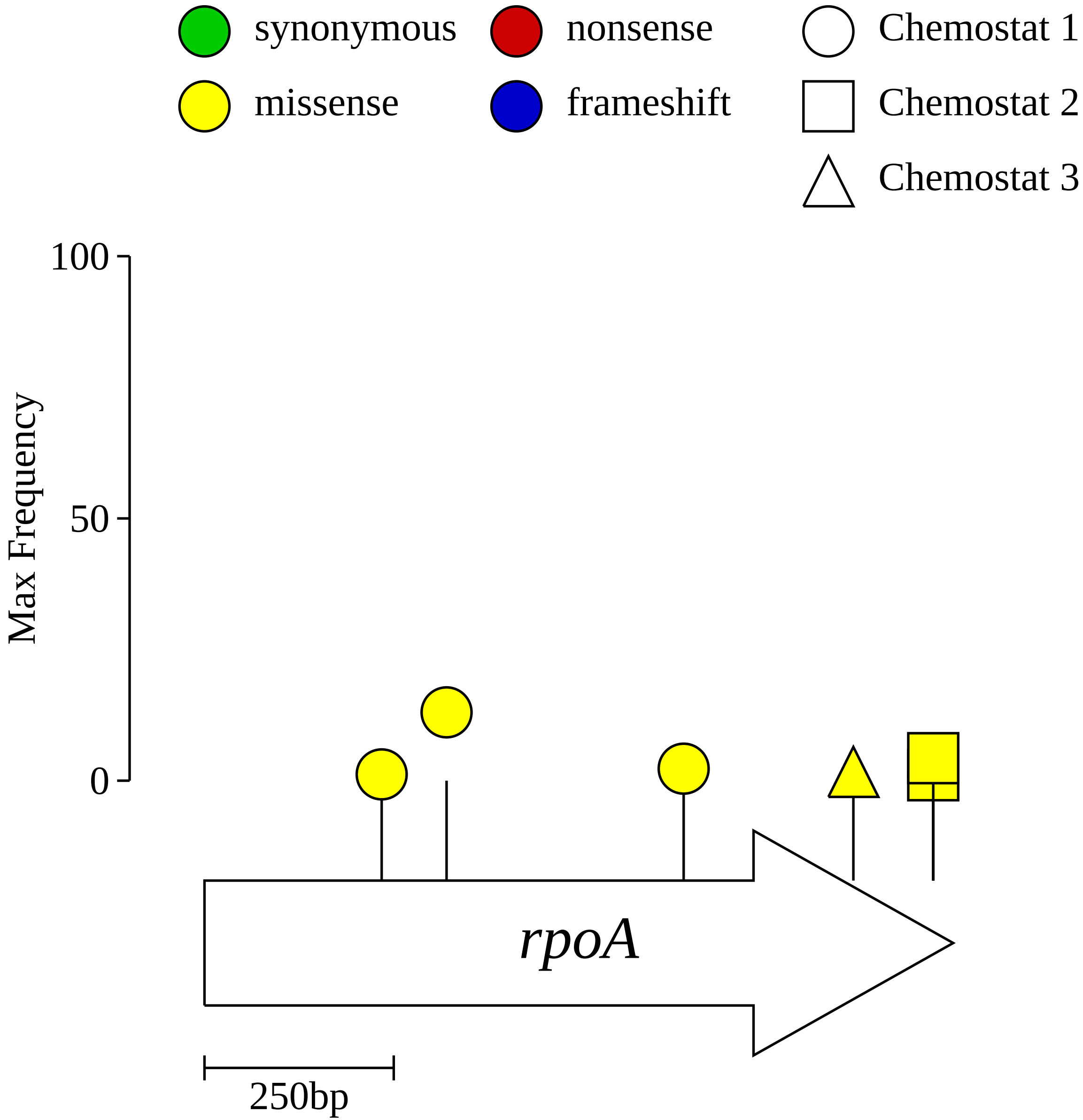
synonymous
nonsense
Chemostat 1
missense
frameshift
Chemostat 2
Chemostat 3
100
50
0
Max Frequency
rpoA
250bp

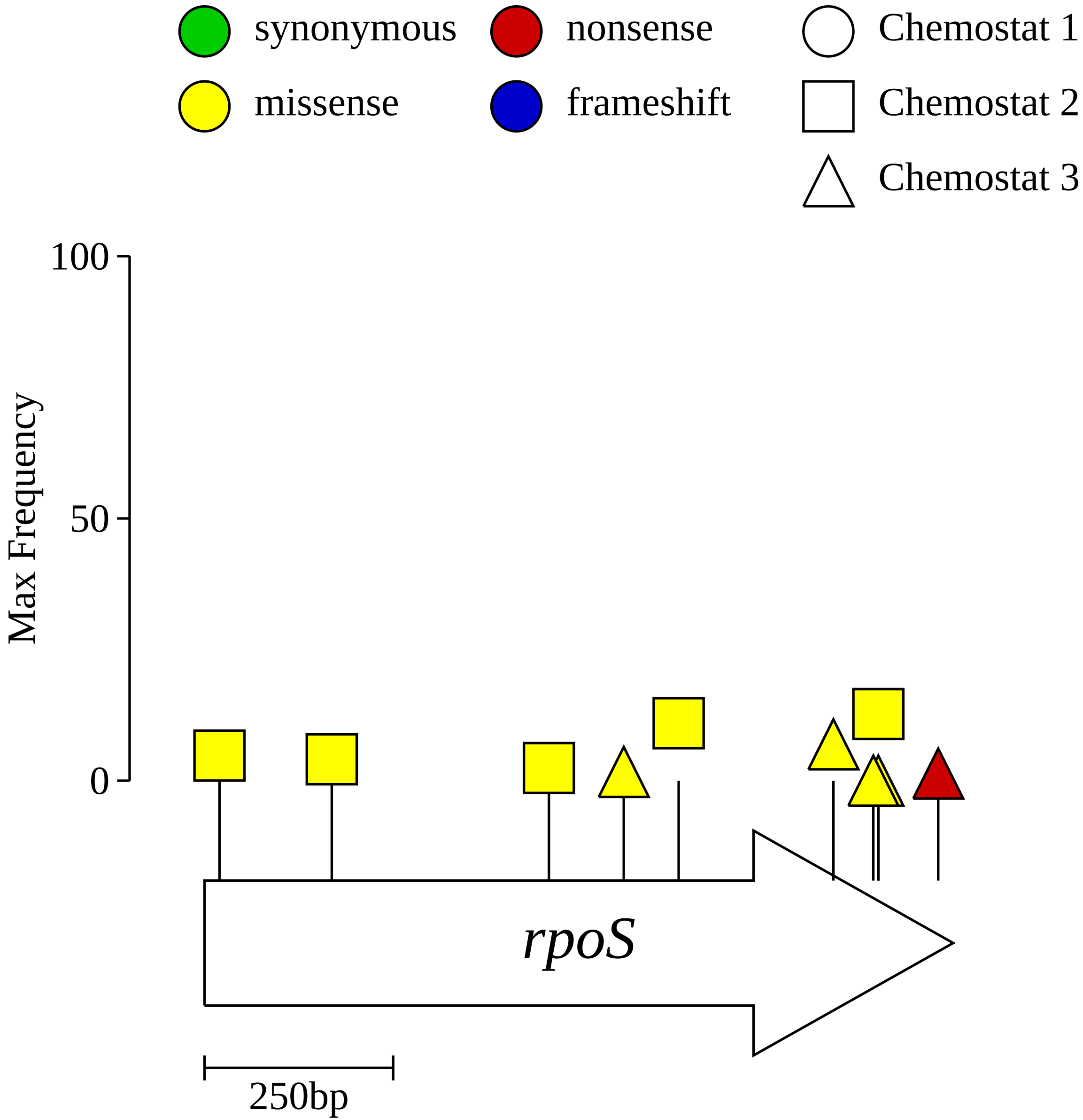

synonymous
nonsense
Chemostat 1
missense
frameshift
Chemostat 2
Chemostat 3
100
50
0
Max Frequency
rpoS
250bp

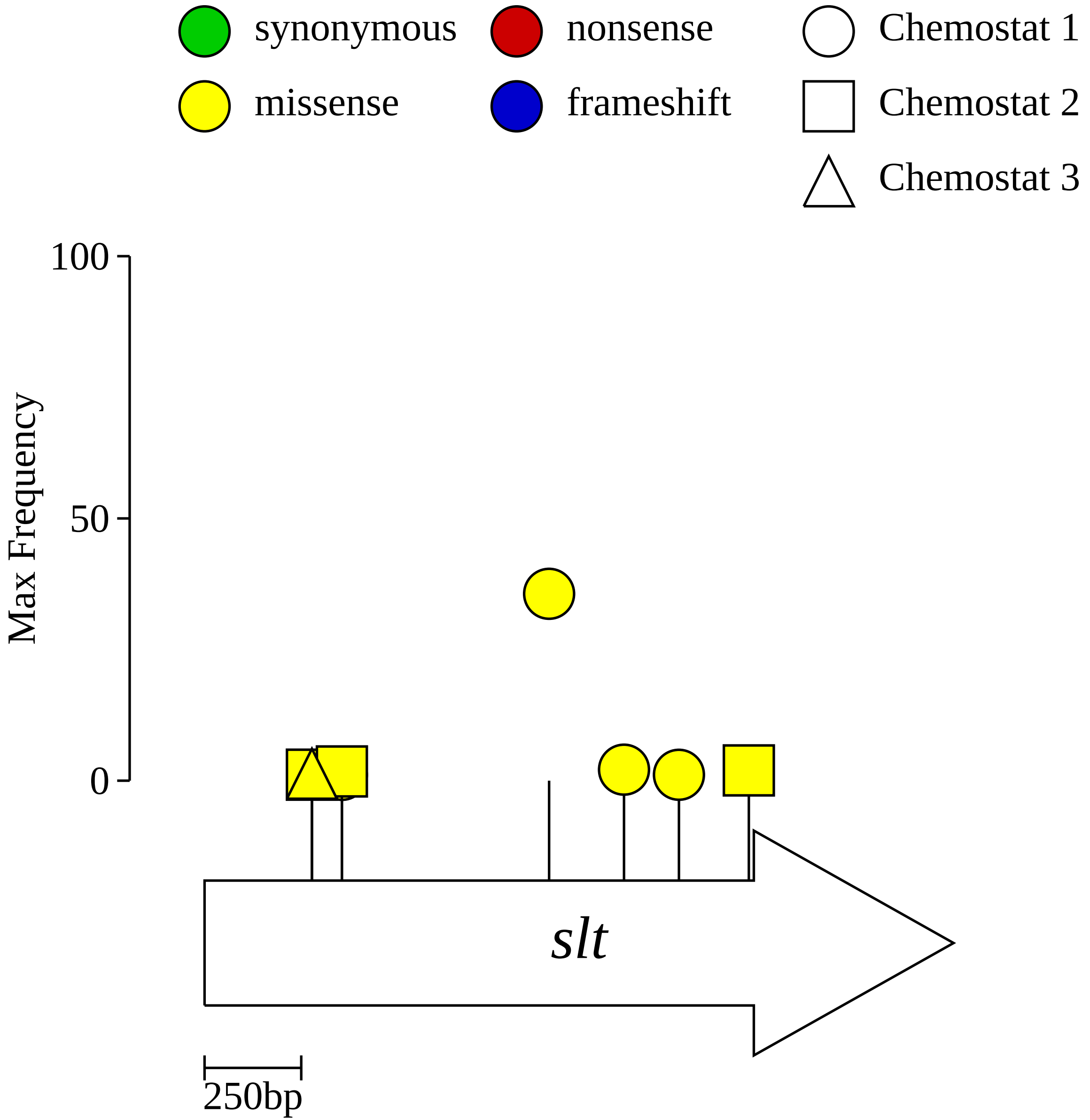
synonymous
nonsense
Chemostat 1
missense
frameshift
Chemostat 2
Chemostat 3
100
50
0
Max Frequency
slt
250bp

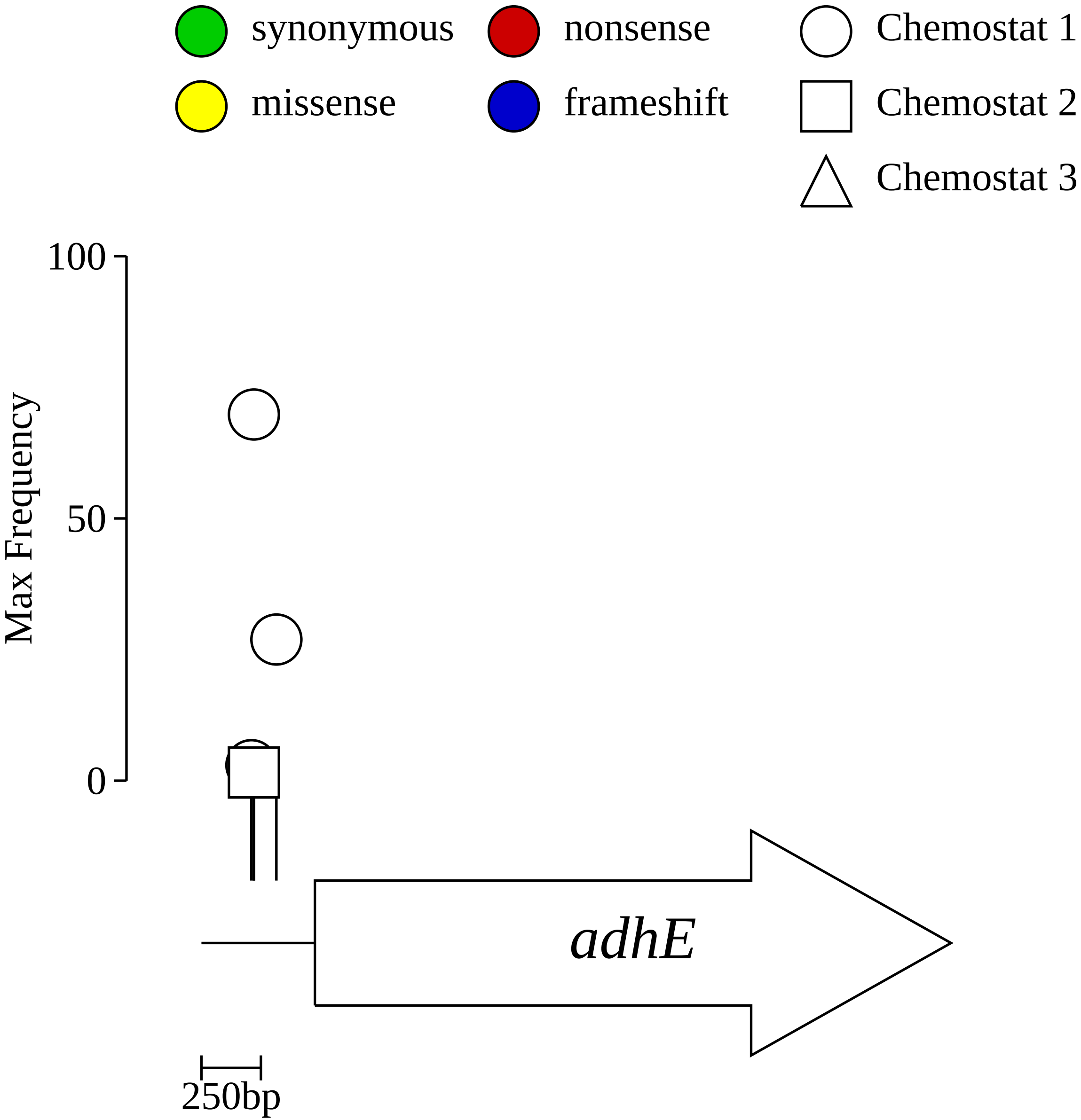
synonymous
nonsense
Chemostat 1
missense
frameshift
Chemostat 2
Chemostat 3
100
50
0
Max Frequency
adhE
250bp

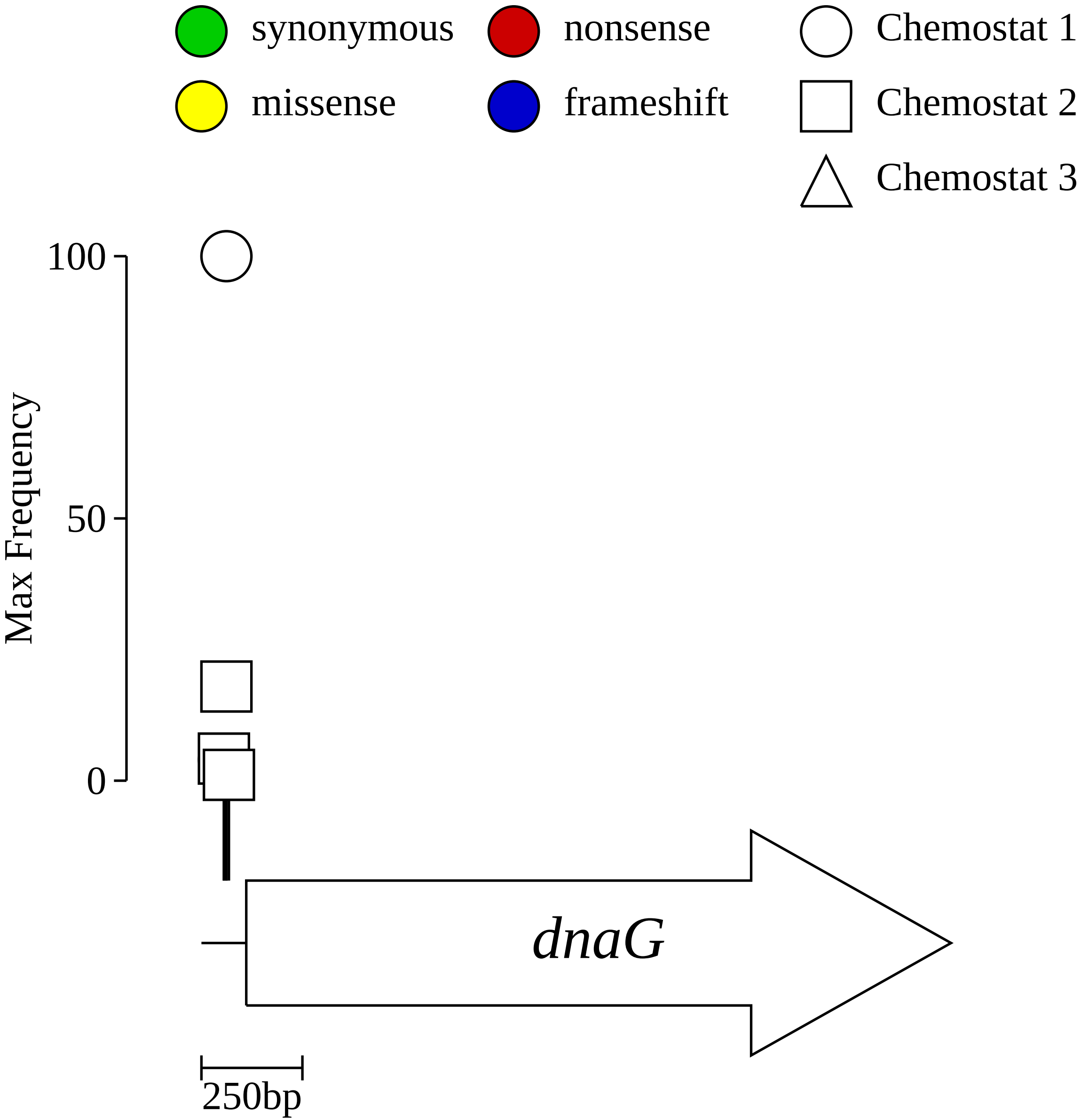
synonymous
missense
nonsense
frameshift
Chemostat 1
Chemostat 2
Chemostat 3
100
50
0
Max Frequency
dnaG
250bp

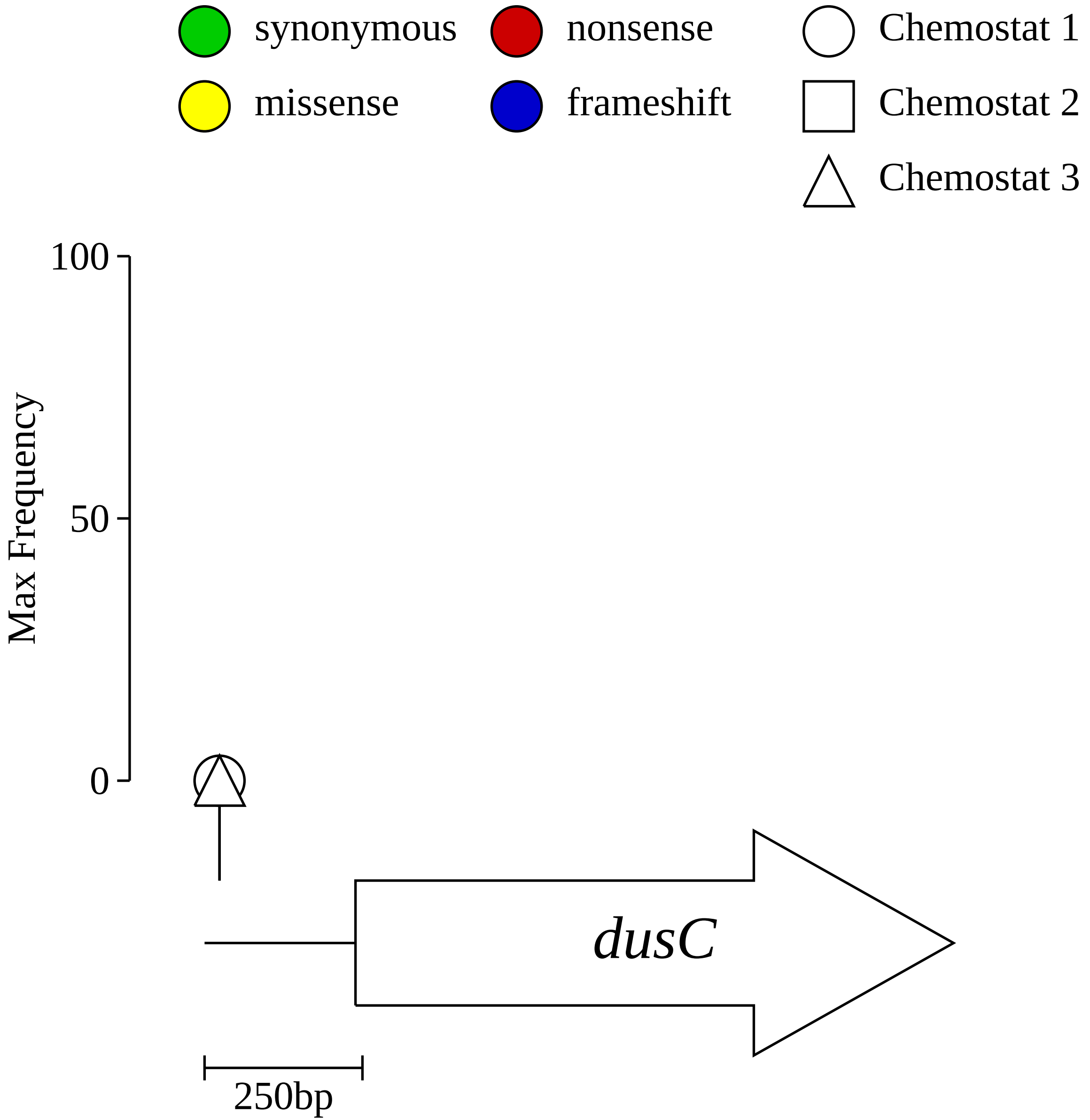
synonymous
nonsense
Chemostat 1
missense
frameshift
Chemostat 2
Chemostat 3
100
50
0
Max Frequency
dusC
250bp

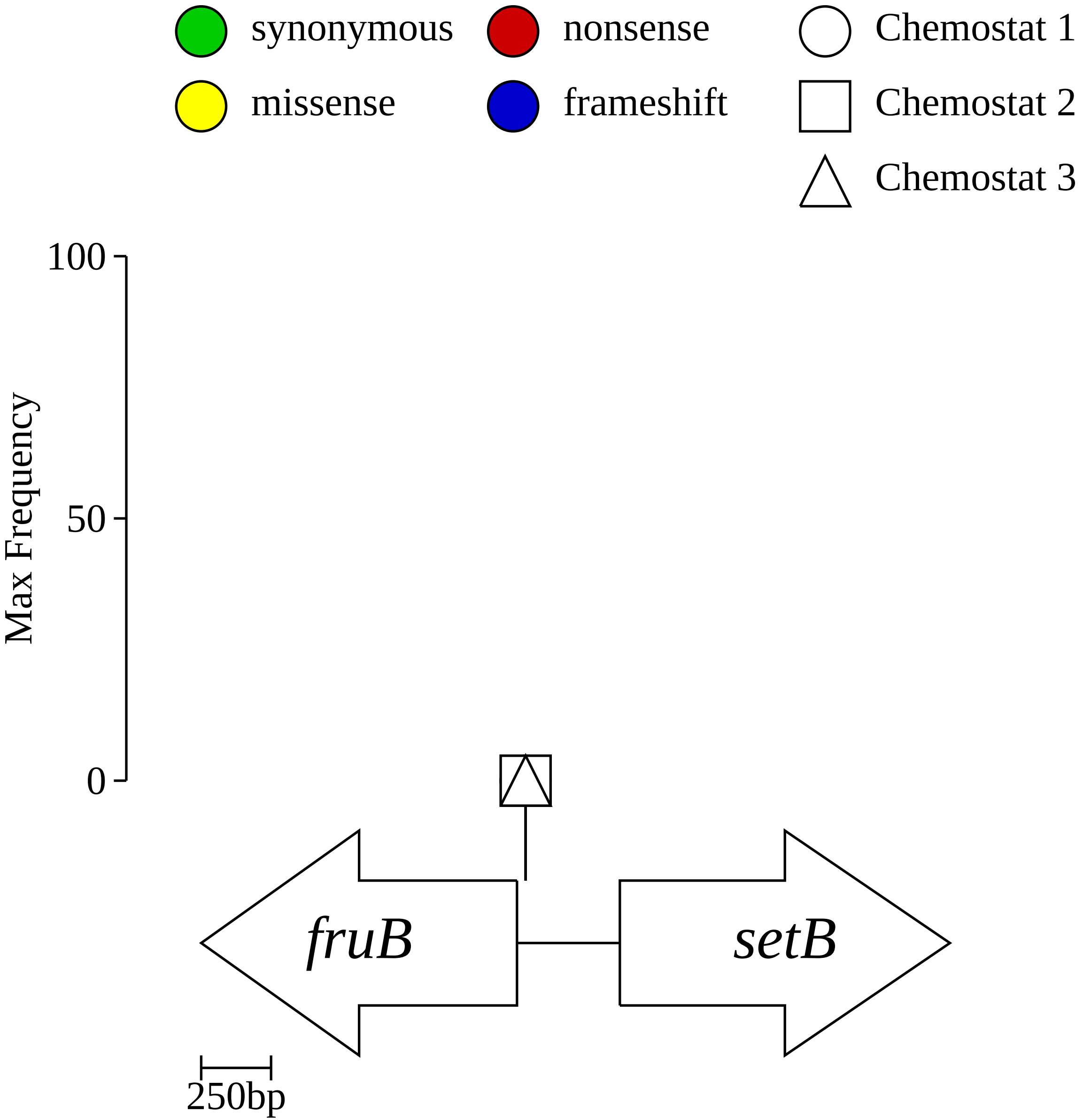
synonymous
nonsense
Chemostat 1
missense
frameshift
Chemostat 2
Chemostat 3
100
50
0
Max Frequency
fruB
setB
250bp

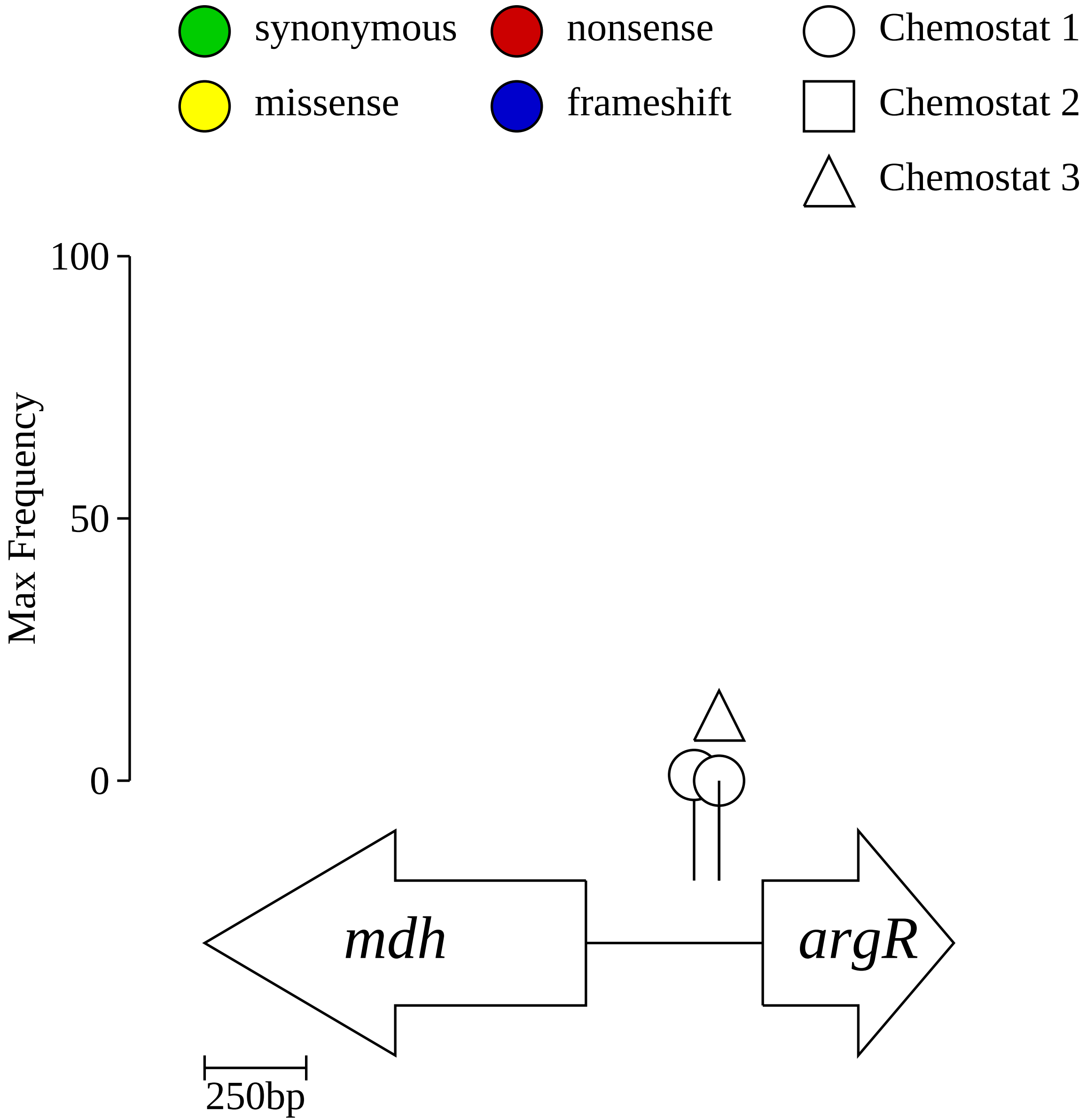

synonymous
missense
nonsense
frameshift
Chemostat 1
Chemostat 2
Chemostat 3
100
50
0
Max Frequency
mdh
argR
250bp

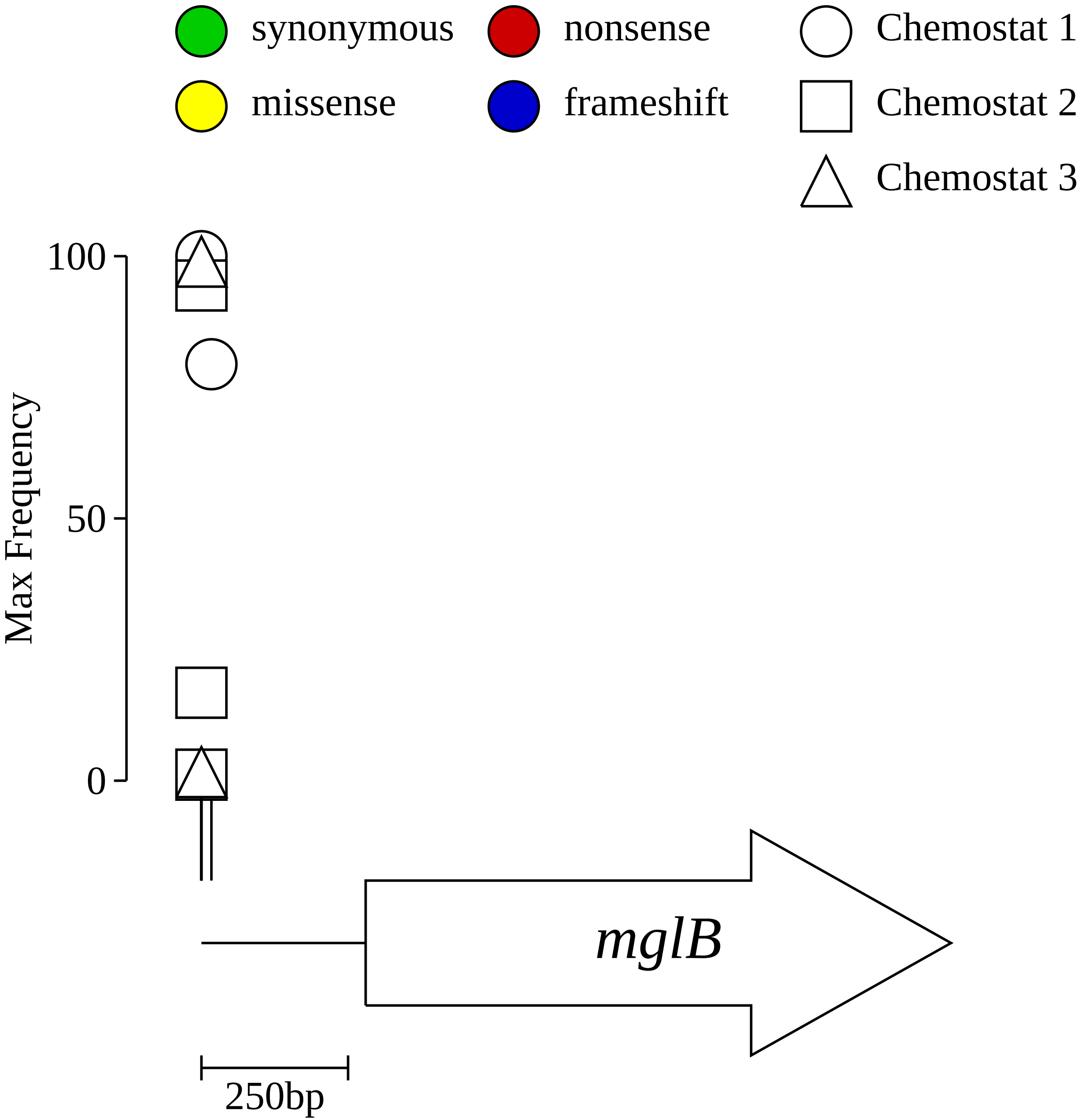

synonymous
nonsense
Chemostat 1
missense
frameshift
Chemostat 2
Chemostat 3
100
50
0
Max Frequency
mglB
250bp

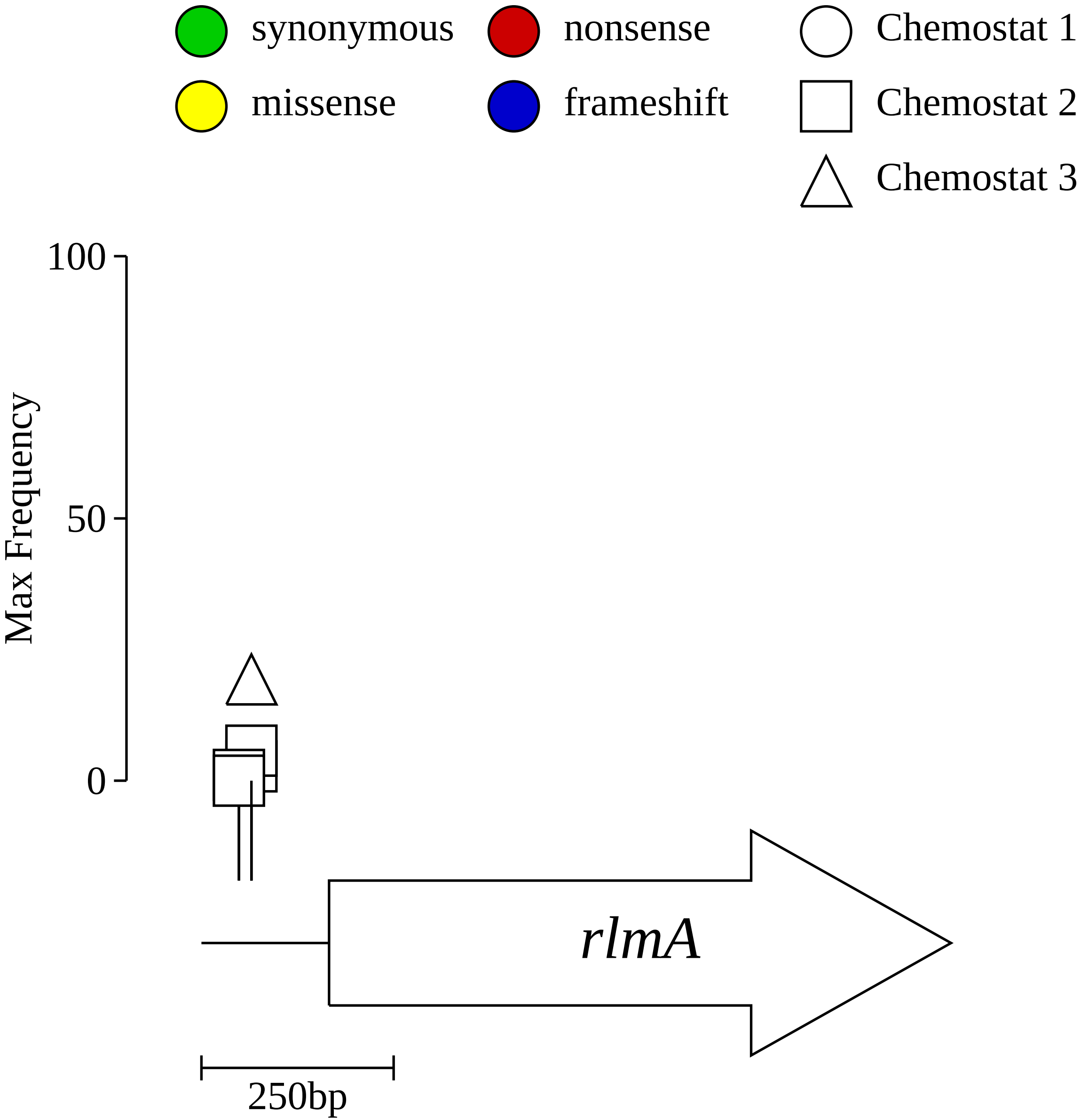

synonymous
nonsense
Chemostat 1
missense
frameshift
Chemostat 2
Chemostat 3
100
50
0
Max Frequency
rlmA
250bp

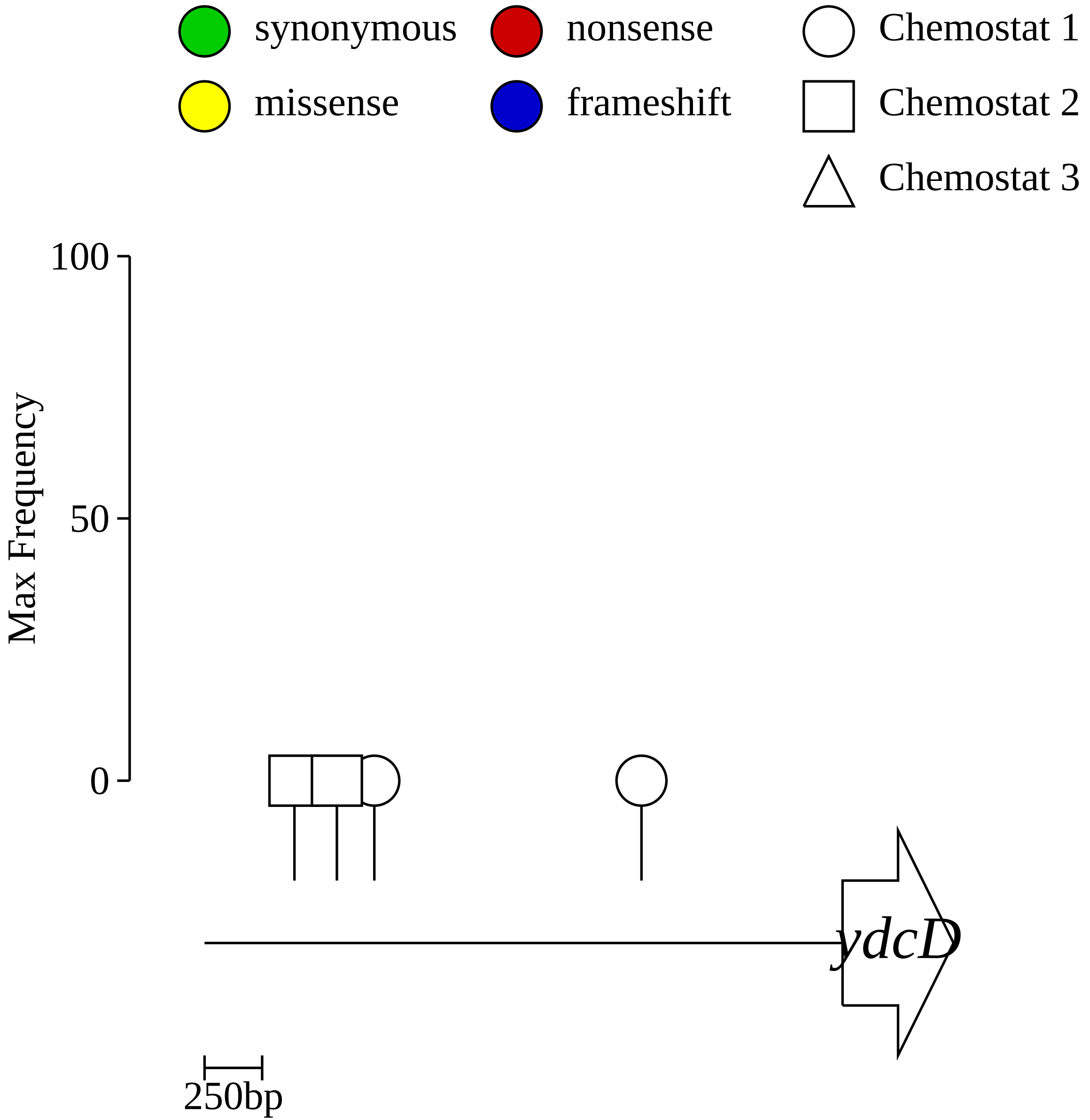
synonymous
nonsense
Chemostat 1
missense
frameshift
Chemostat 2
Chemostat 3
100
50
0
Max Frequency
ydcD
250bp

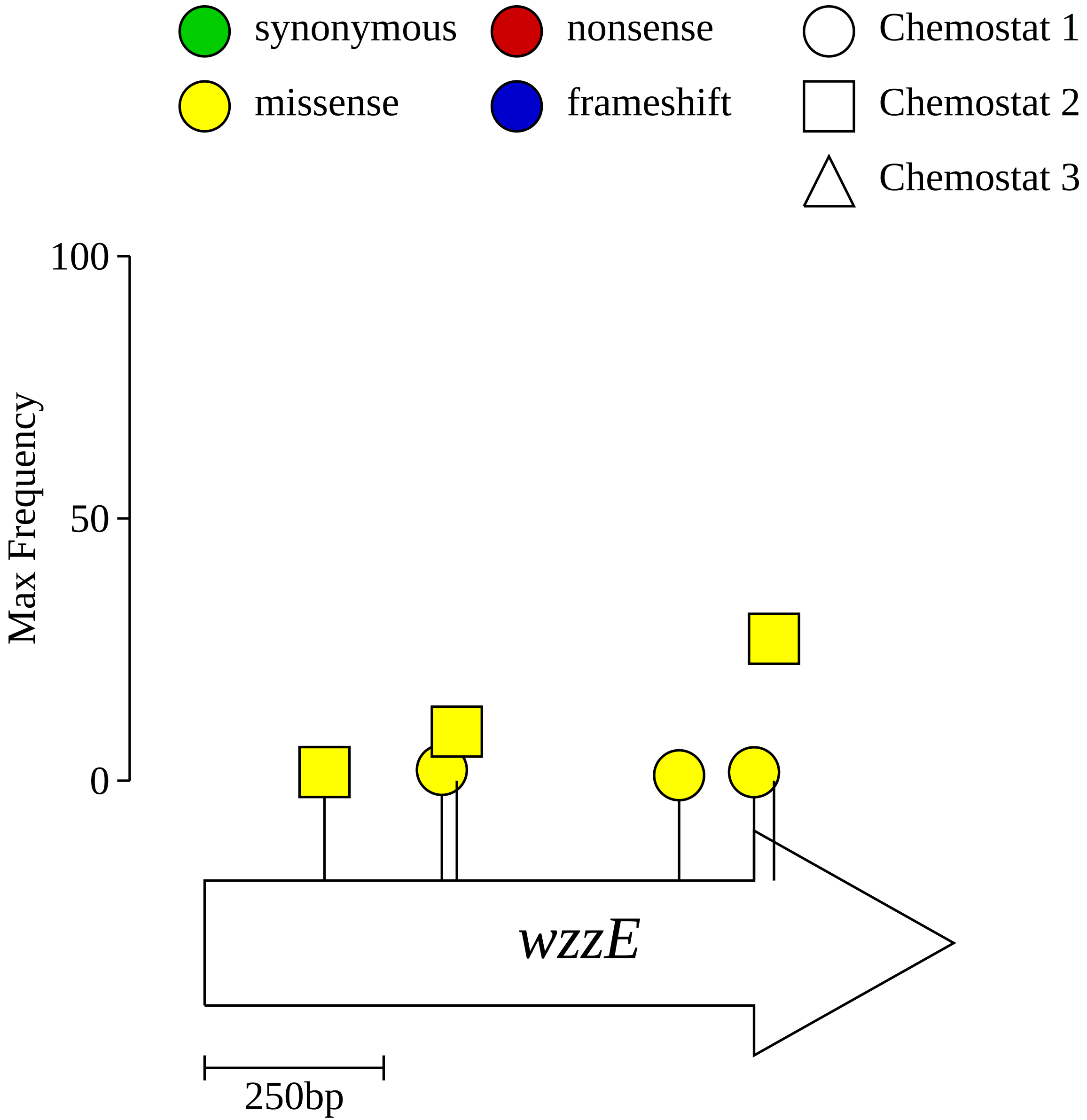

synonymous
nonsense
Chemostat 1
missense
frameshift
Chemostat 2
Chemostat 3
100
50
0
Max Frequency
wzzE
250bp

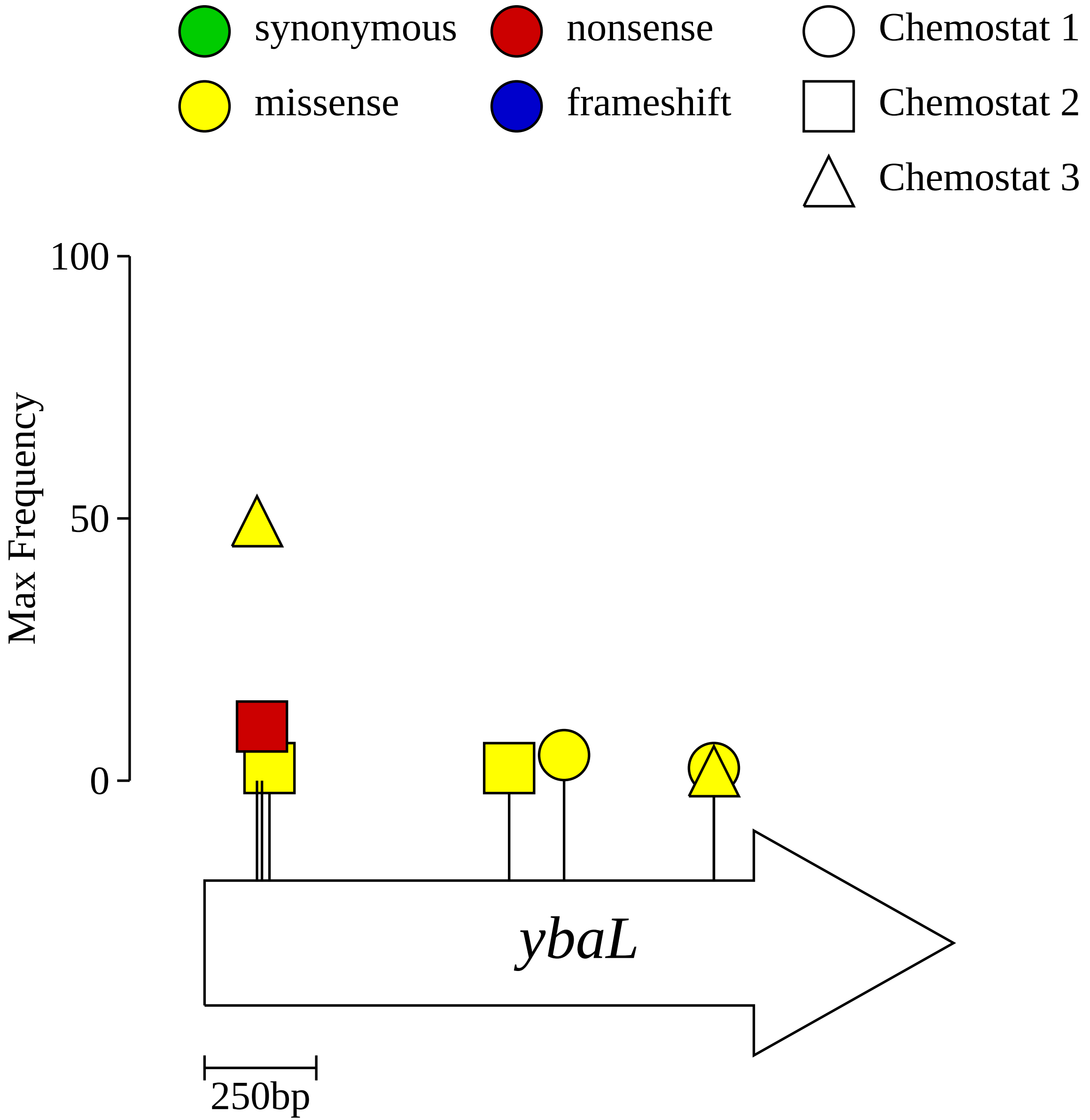

synonymous
nonsense
Chemostat 1
missense
frameshift
Chemostat 2
Chemostat 3
100
50
0
Max Frequency
ybaL
250bp

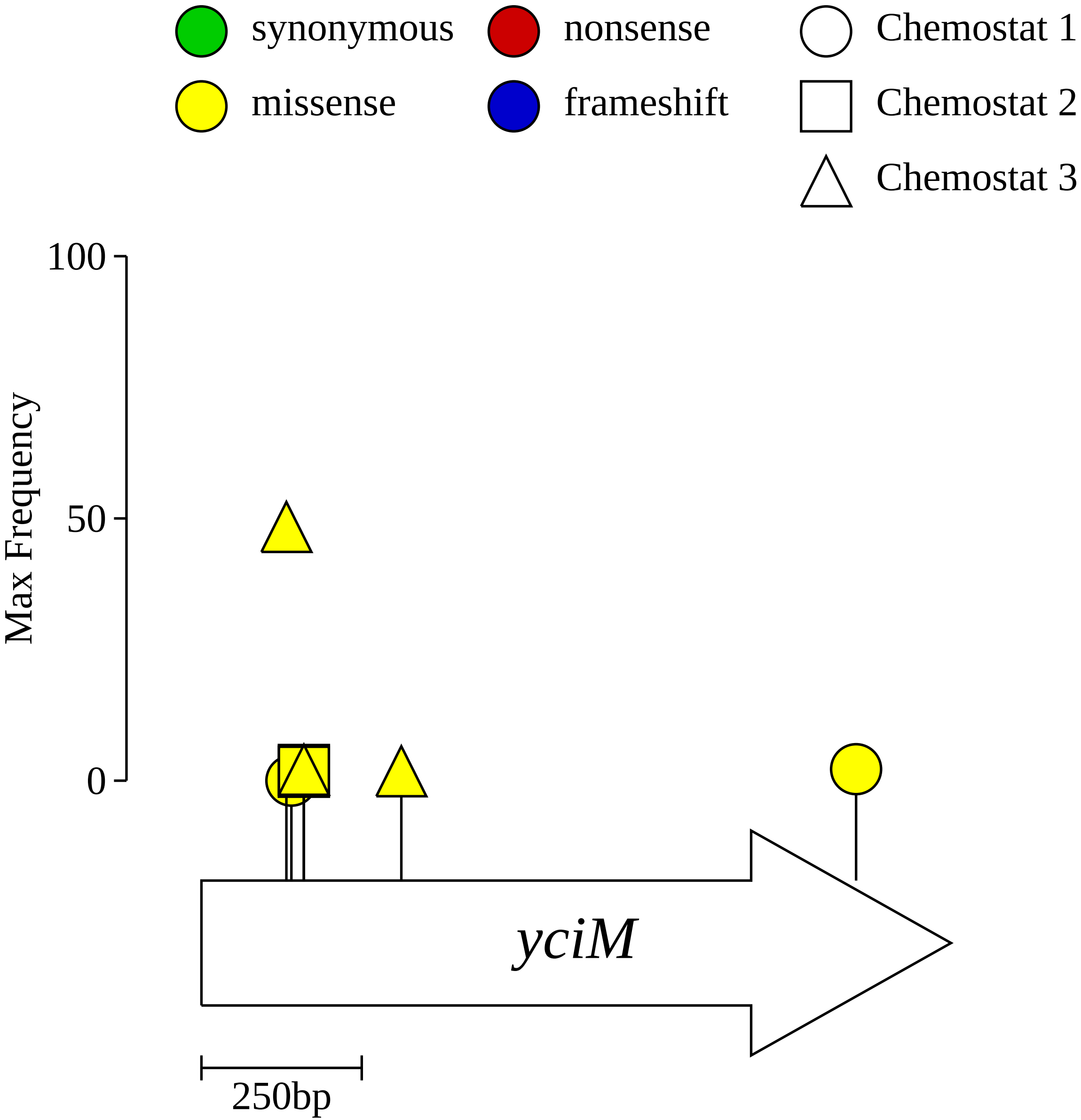

synonymous
nonsense
Chemostat 1
missense
frameshift
Chemostat 2
Chemostat 3
100
50
0
Max Frequency
yciM
250bp

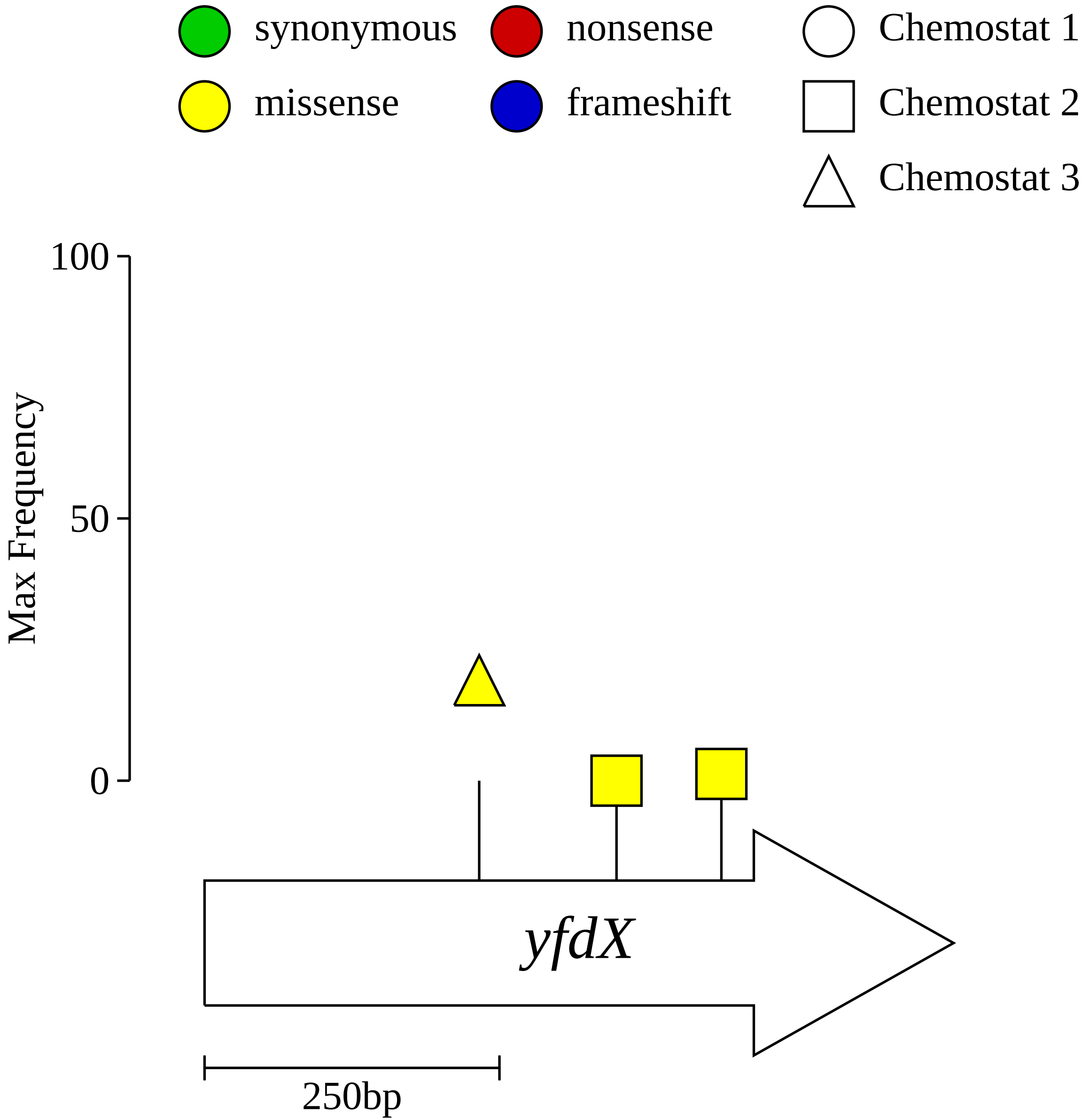
synonymous
nonsense
Chemostat 1
missense
frameshift
Chemostat 2
Chemostat 3
Max Frequency
100
50
0
yfdX
250bp

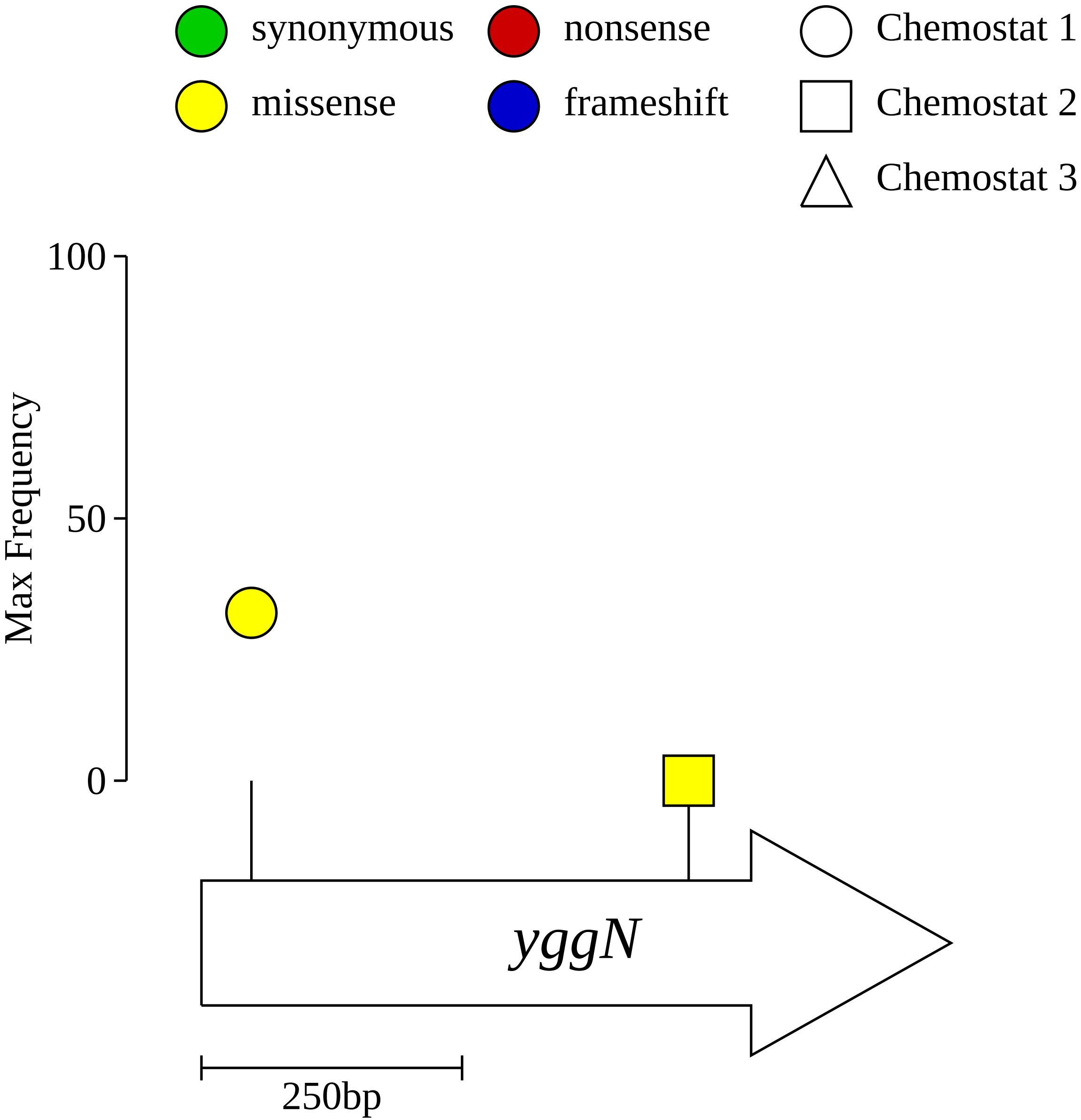
synonymous
nonsense
Chemostat 1
missense
frameshift
Chemostat 2
Chemostat 3
100
50
0
Max Frequency
yggN
250bp

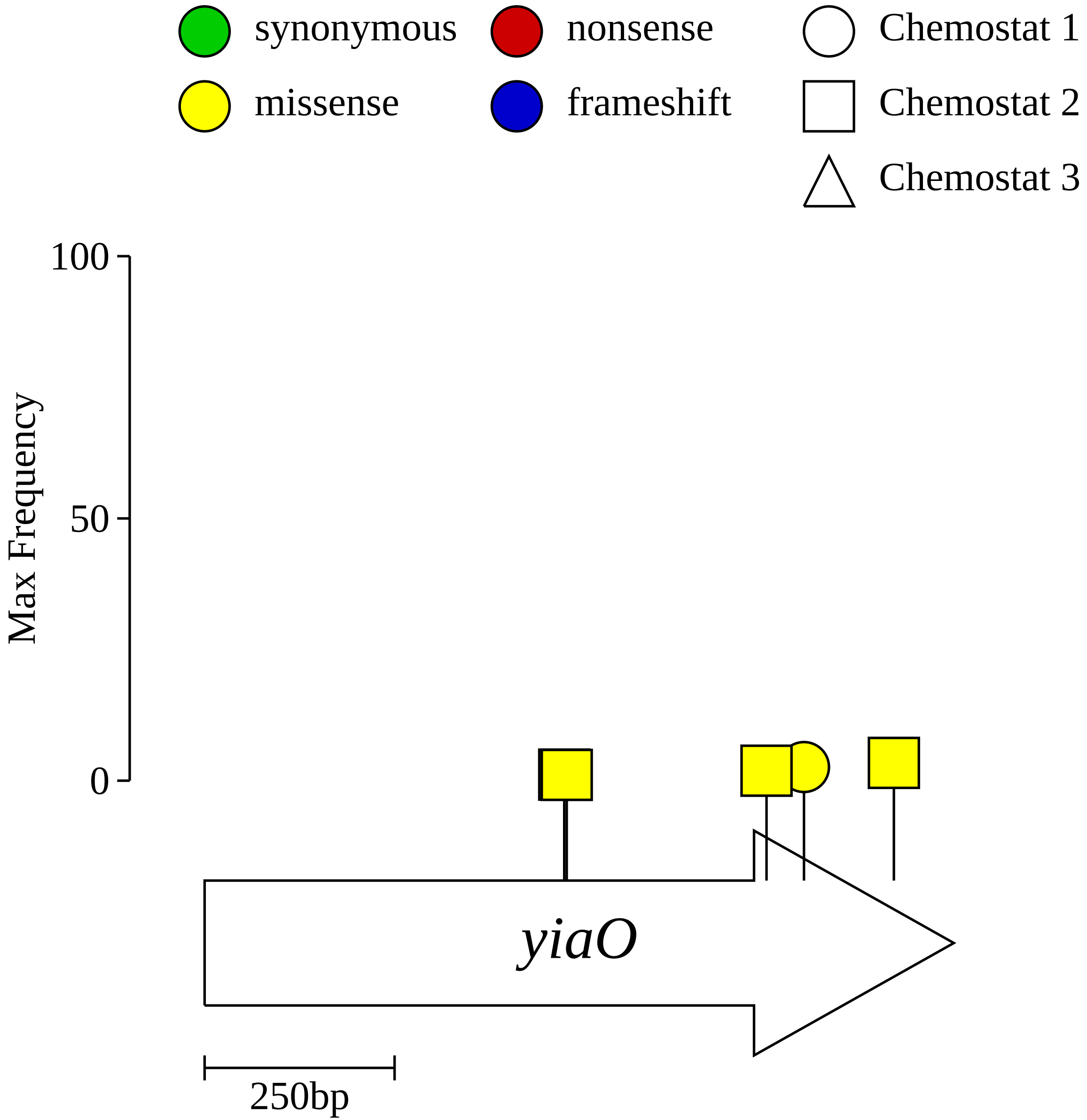
synonymous
nonsense
Chemostat 1
missense
frameshift
Chemostat 2
Chemostat 3
100
50
0
Max Frequency
yiaO
250bp

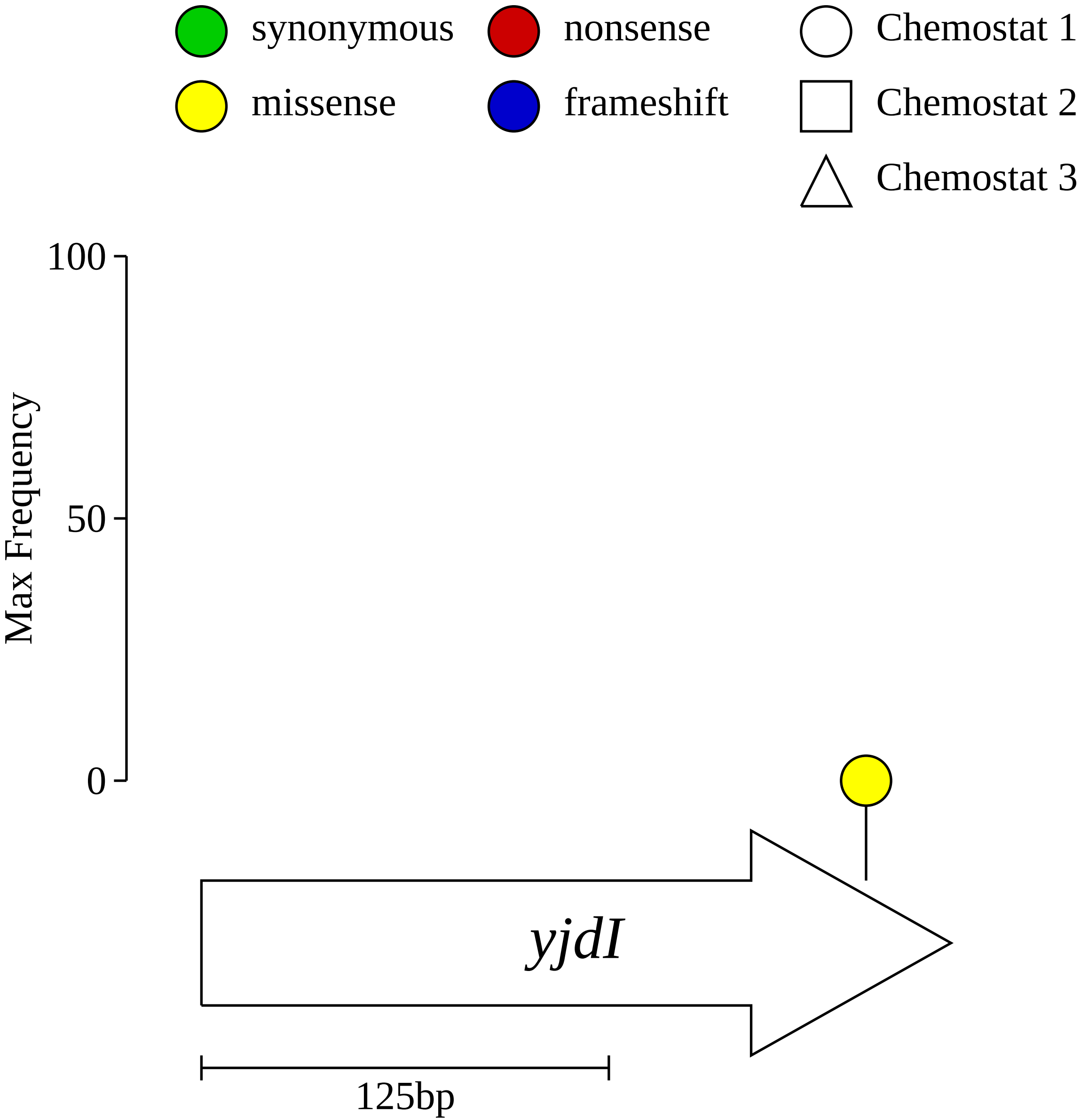

synonymous
nonsense
Chemostat 1
missense
frameshift
Chemostat 2
Chemostat 3
100
50
0
Max Frequency
yjdI
125bp

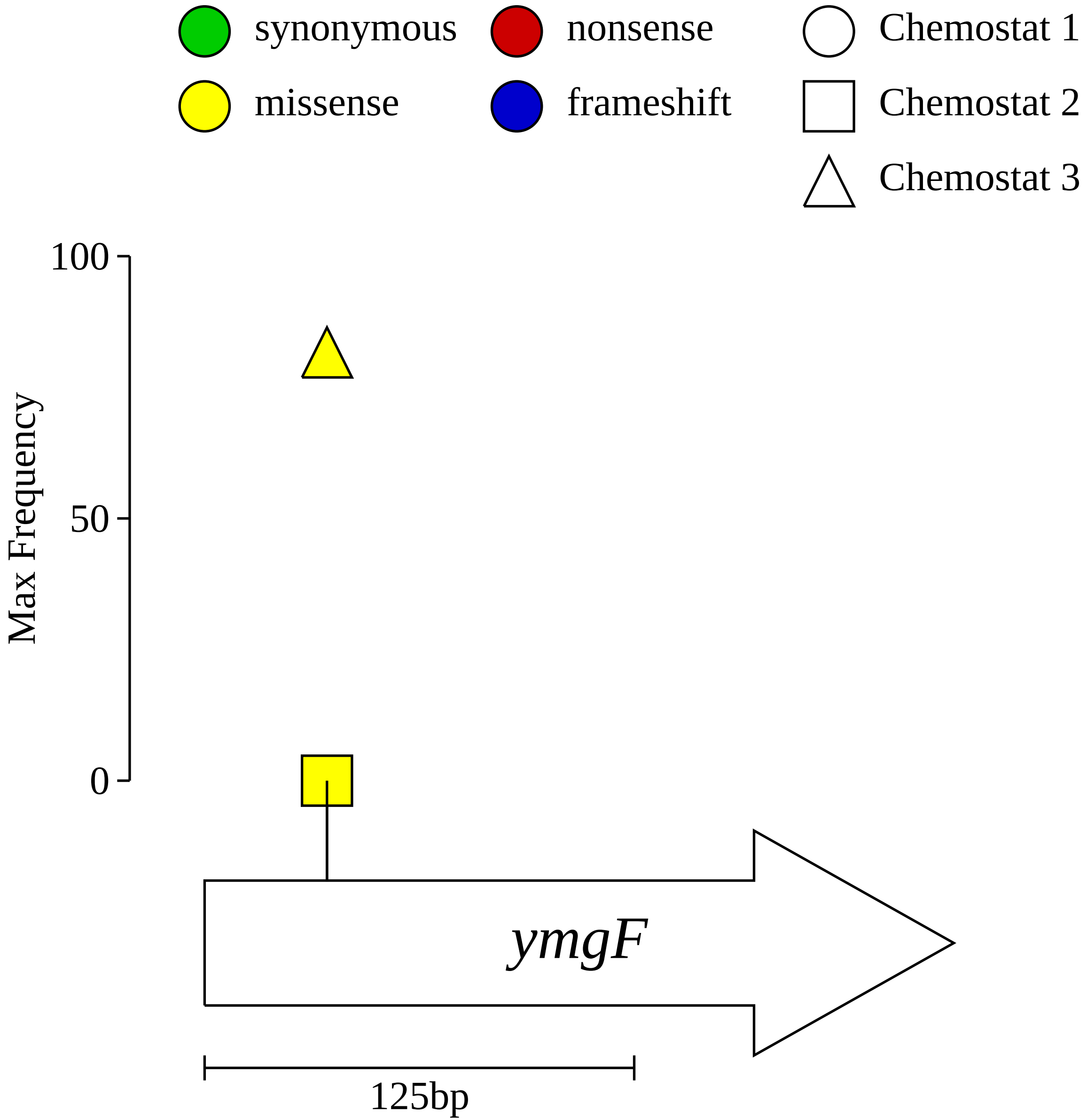
synonymous
nonsense
Chemostat 1
missense
frameshift
Chemostat 2
Chemostat 3
100
50
0
Max Frequency
ymgF
125bp

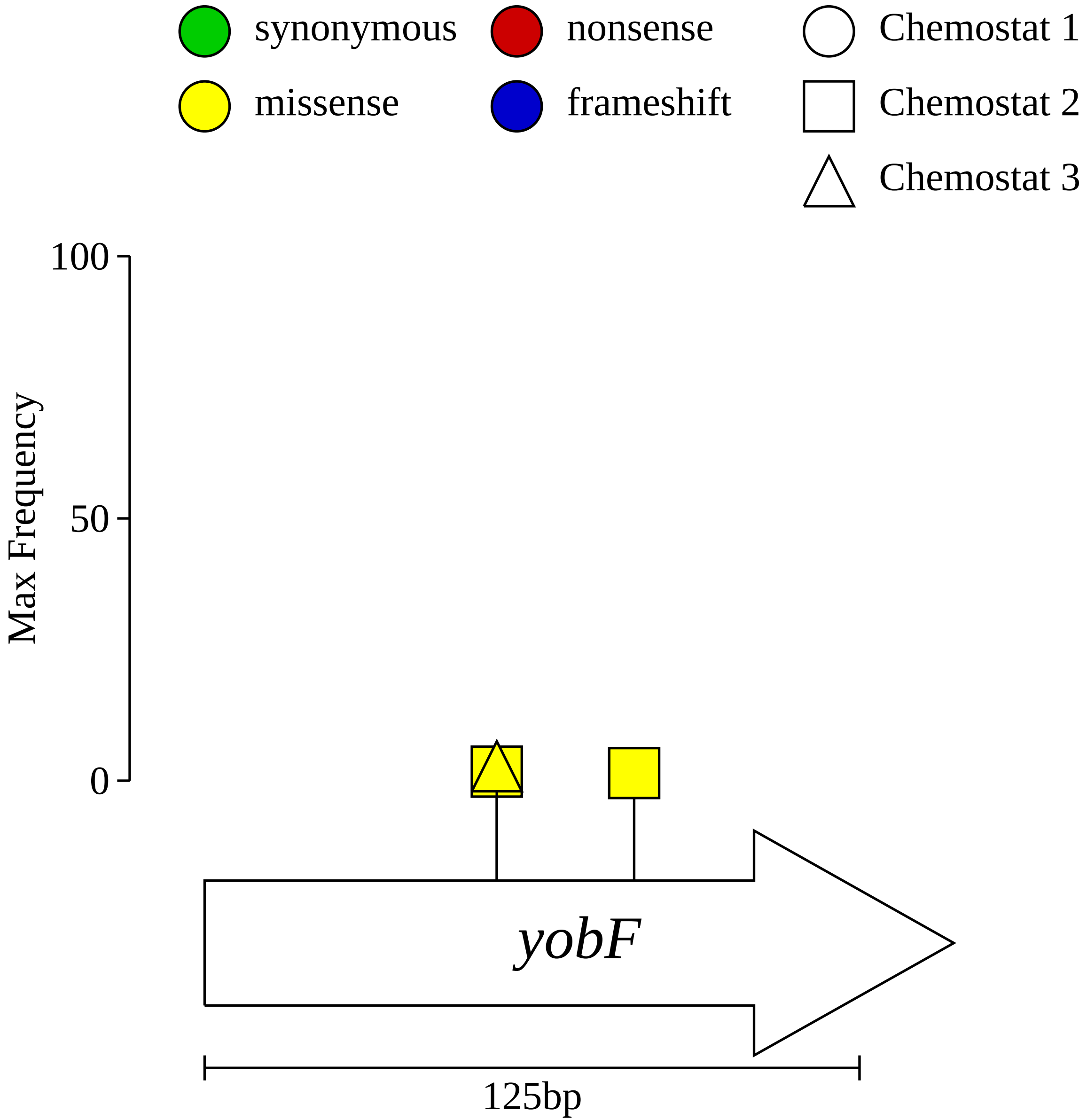

synonymous
missense
nonsense
frameshift
Chemostat 1
Chemostat 2
Chemostat 3
100
50
0
Max Frequency
yobF
125bp

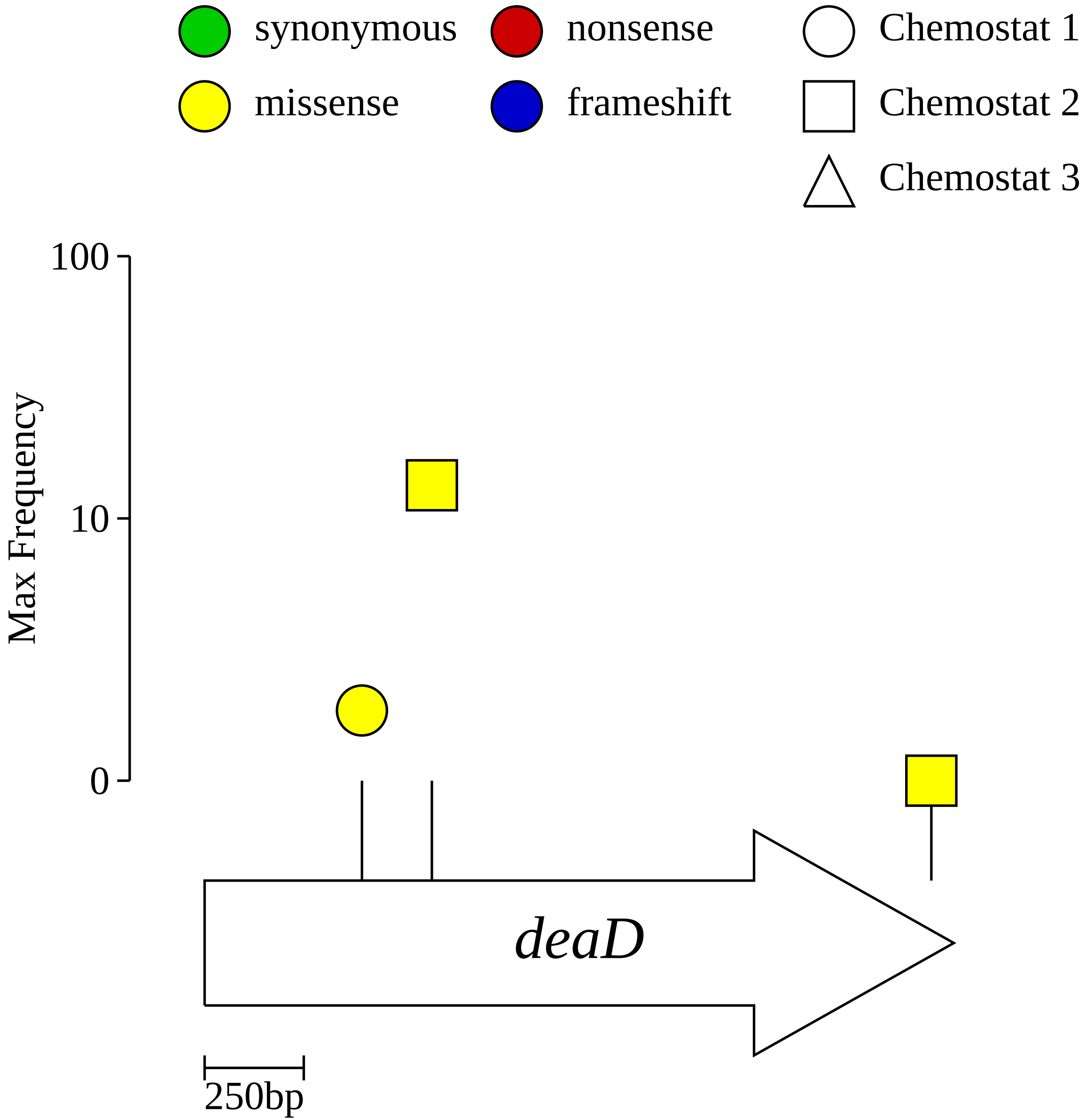
synonymous
nonsense
Chemostat 1
missense
frameshift
Chemostat 2
Chemostat 3
100
10
0
Max Frequency
deaD
250bp

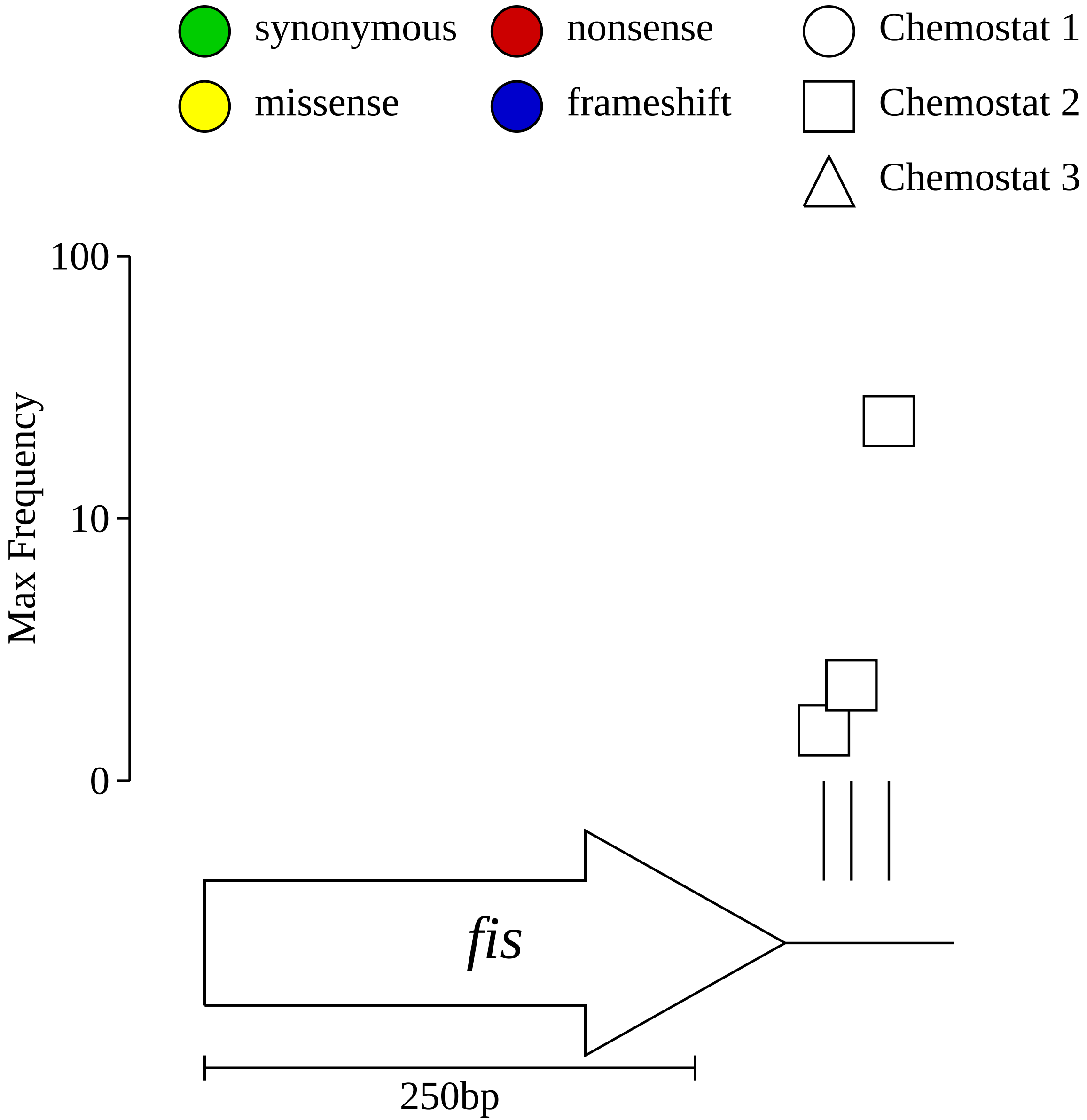
synonymous
nonsense
Chemostat 1
missense
frameshift
Chemostat 2
Chemostat 3
100
10
0
Max Frequency
fis
250bp

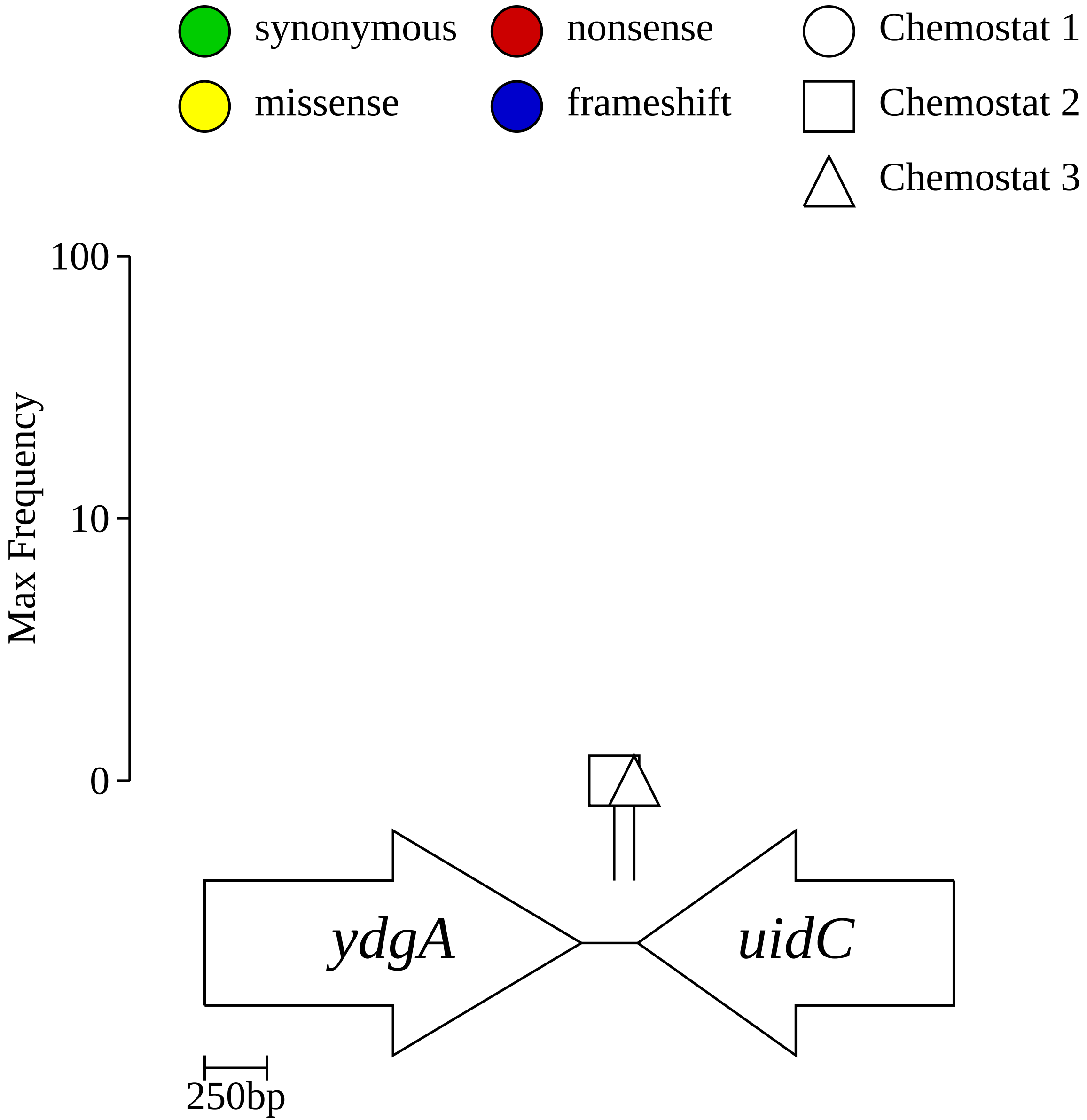
synonymous
nonsense
Chemostat 1
missense
frameshift
Chemostat 2
Chemostat 3
100
10
0
Max Frequency
ydgA
uidC
250bp

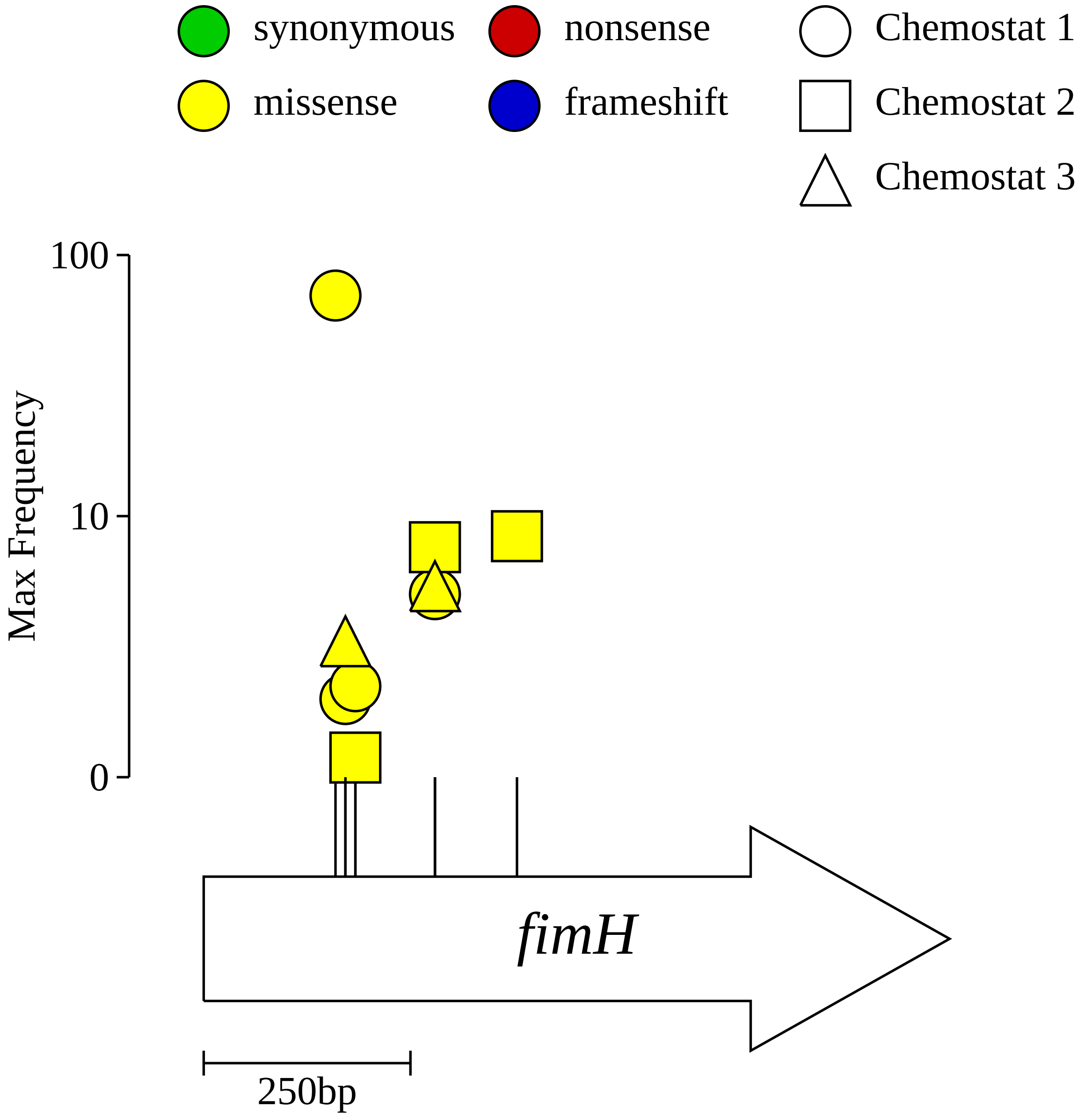

synonymous
nonsense
Chemostat 1
missense
frameshift
Chemostat 2
Chemostat 3
100
10
0
Max Frequency
fimH
250bp

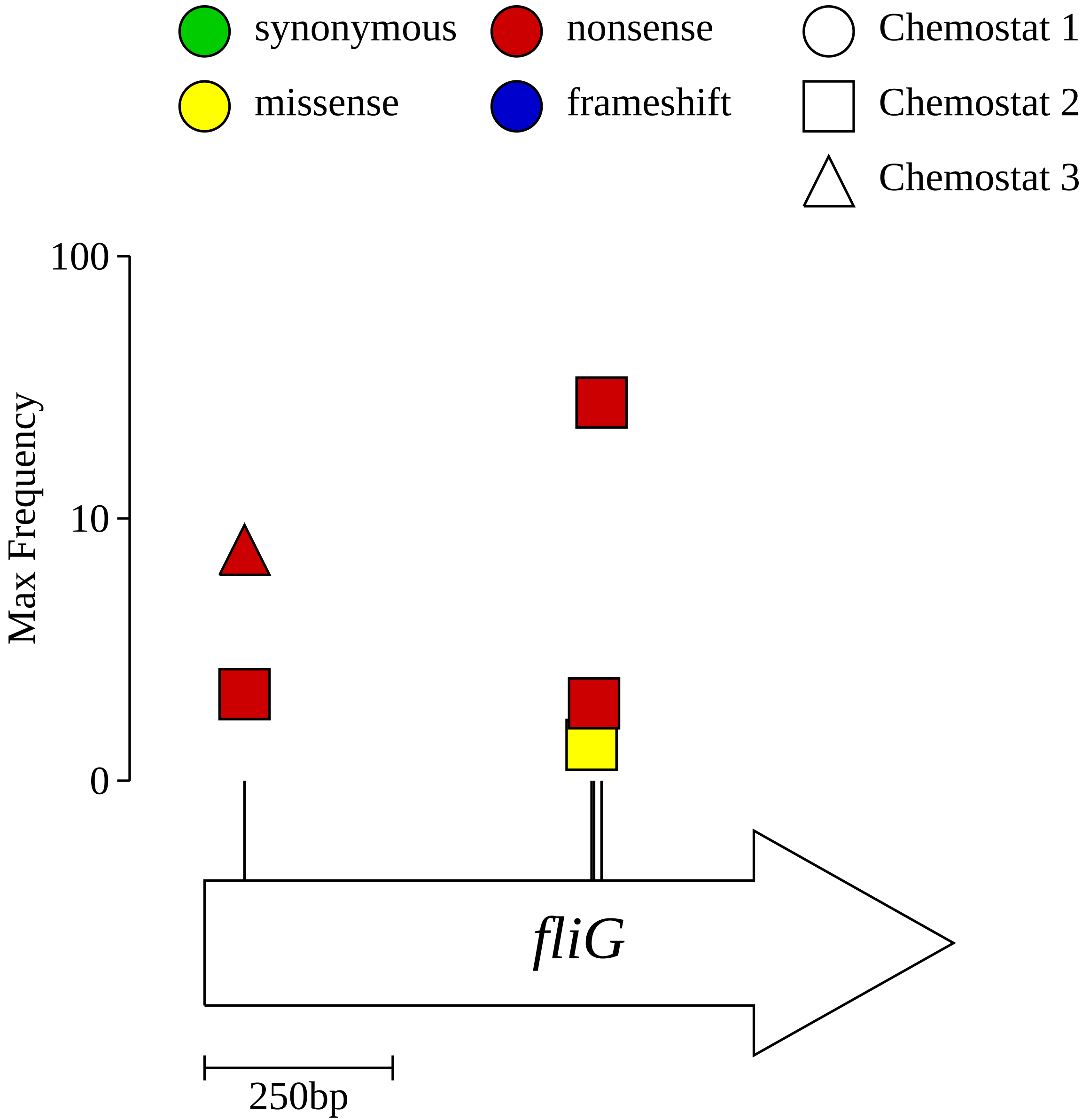

synonymous
nonsense
Chemostat 1
missense
frameshift
Chemostat 2
Chemostat 3
100
10
0
Max Frequency
fliG
250bp

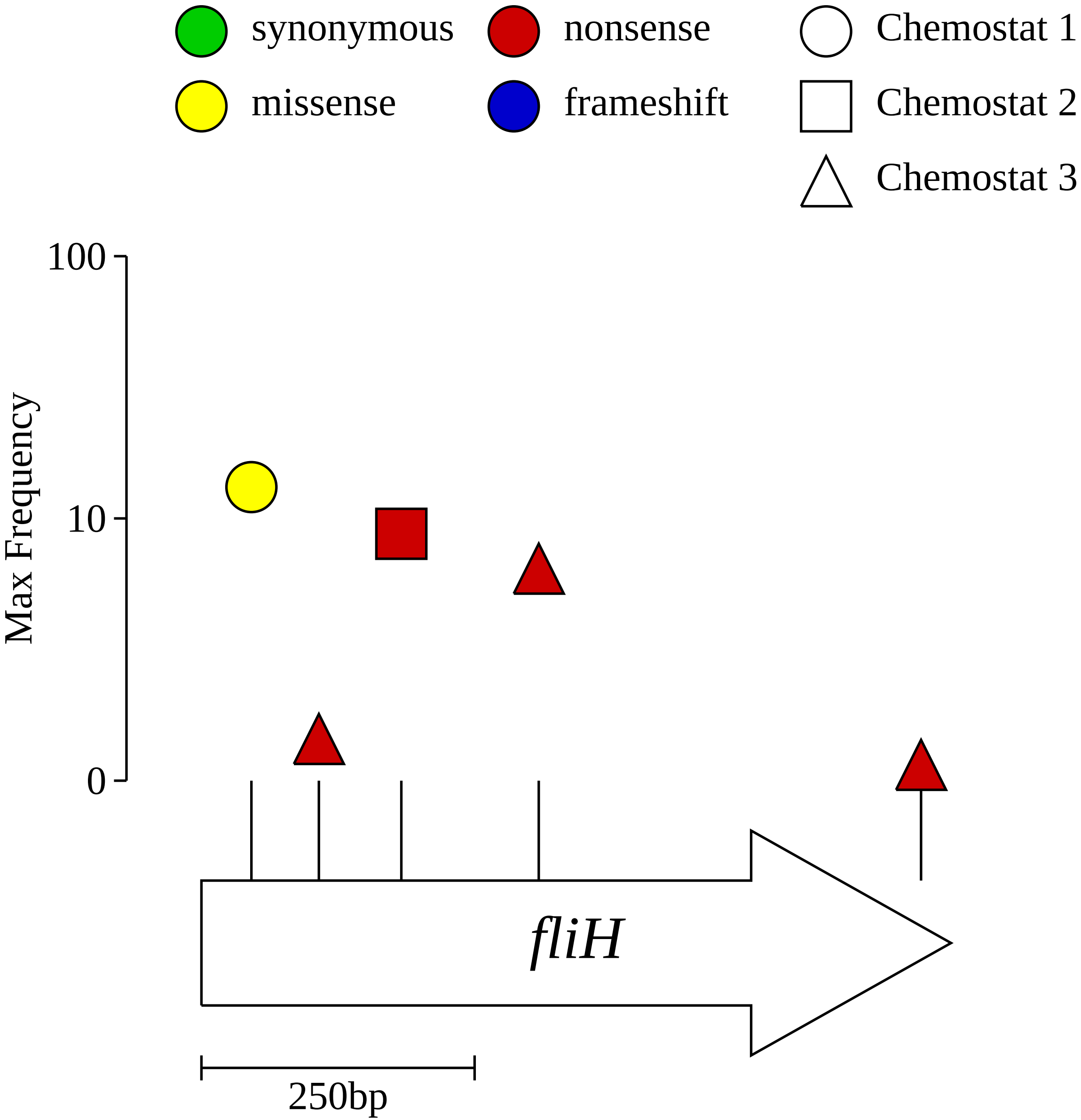
synonymous
nonsense
Chemostat 1
missense
frameshift
Chemostat 2
Chemostat 3
100
10
0
Max Frequency
fliH
250bp

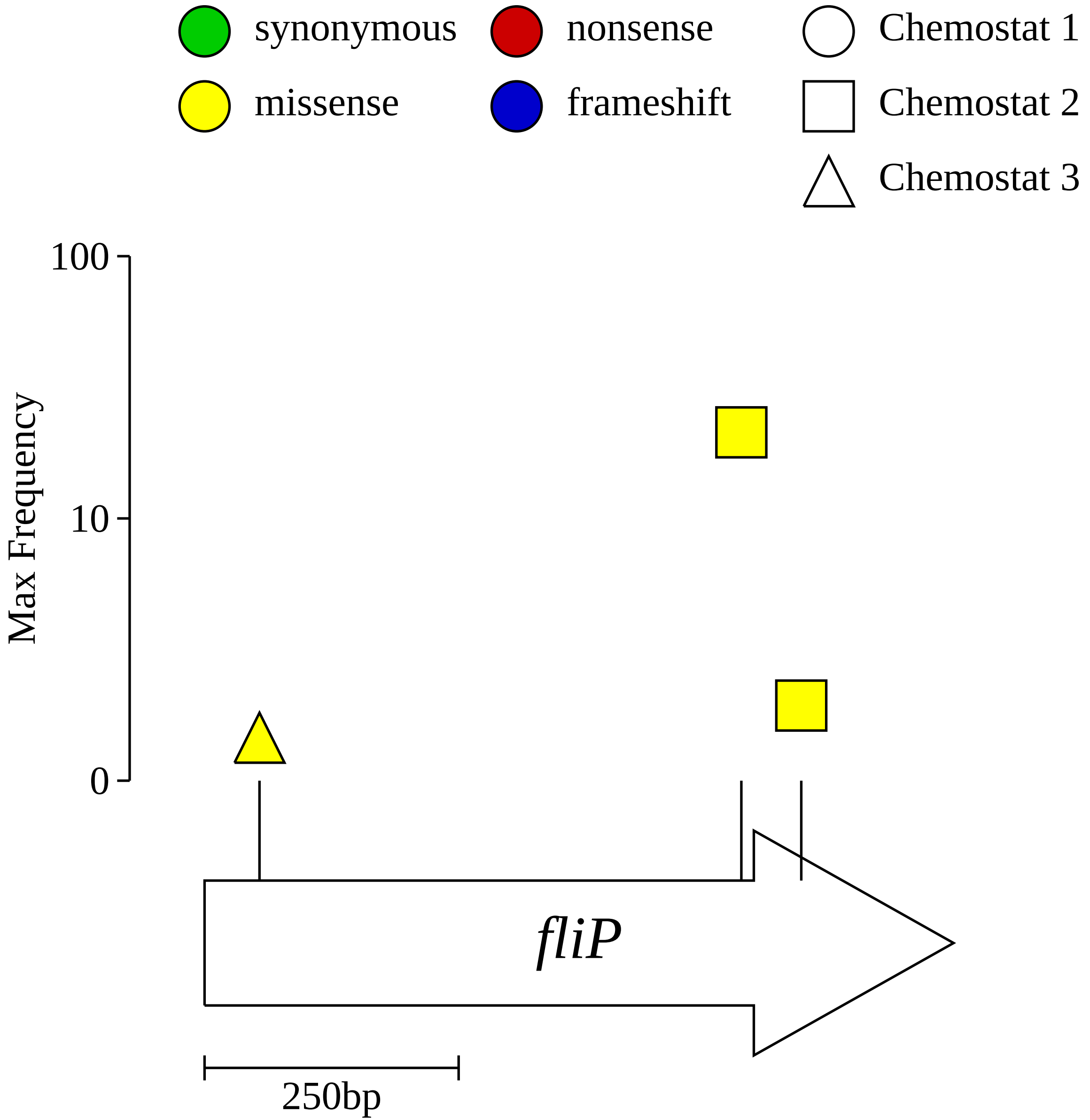

synonymous
missense
nonsense
frameshift
Chemostat 1
Chemostat 2
Chemostat 3
100
10
0
Max Frequency
fliP
250bp

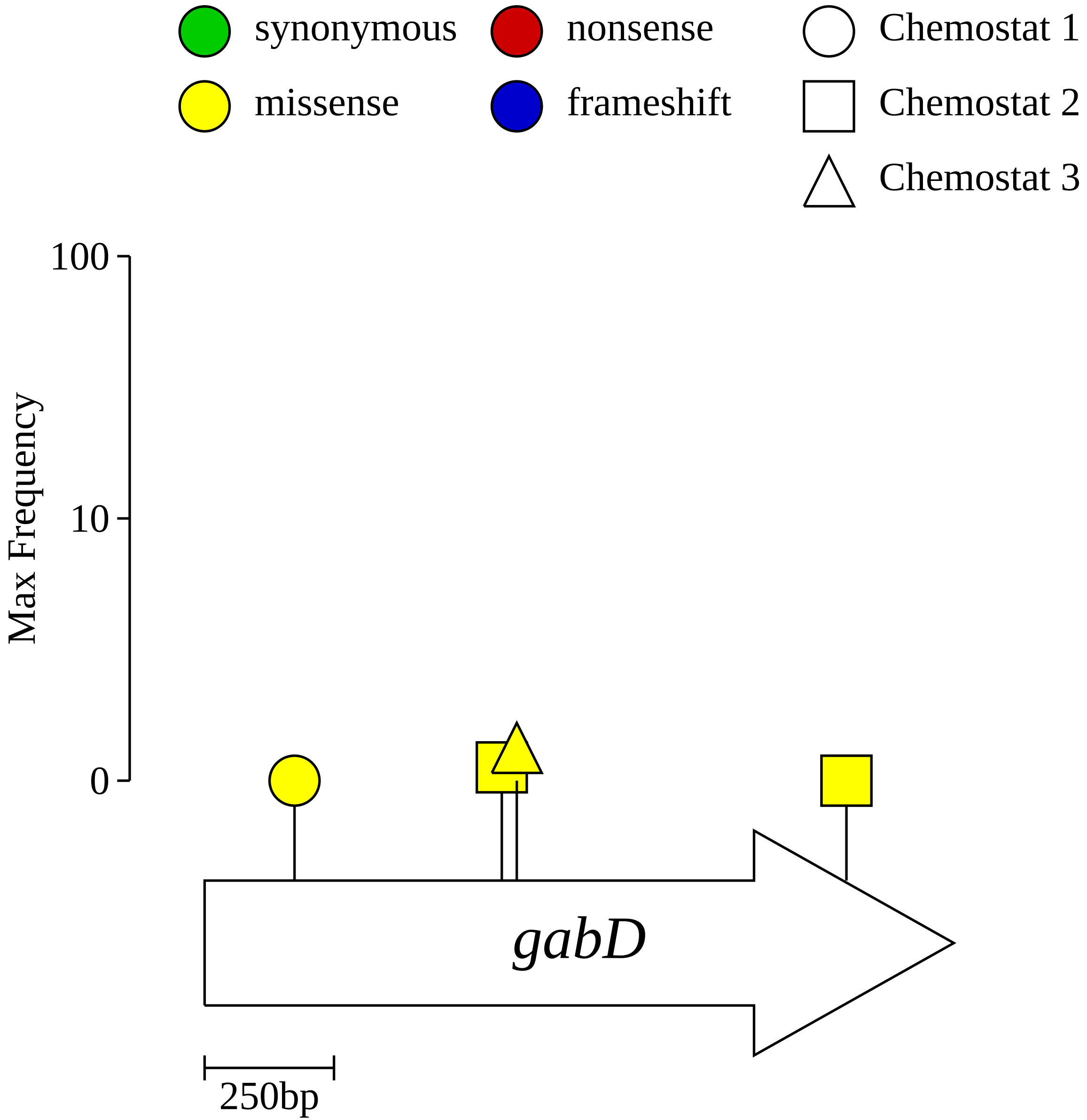

synonymous
nonsense
Chemostat 1
missense
frameshift
Chemostat 2
Chemostat 3
100
10
0
Max Frequency
gabD
250bp

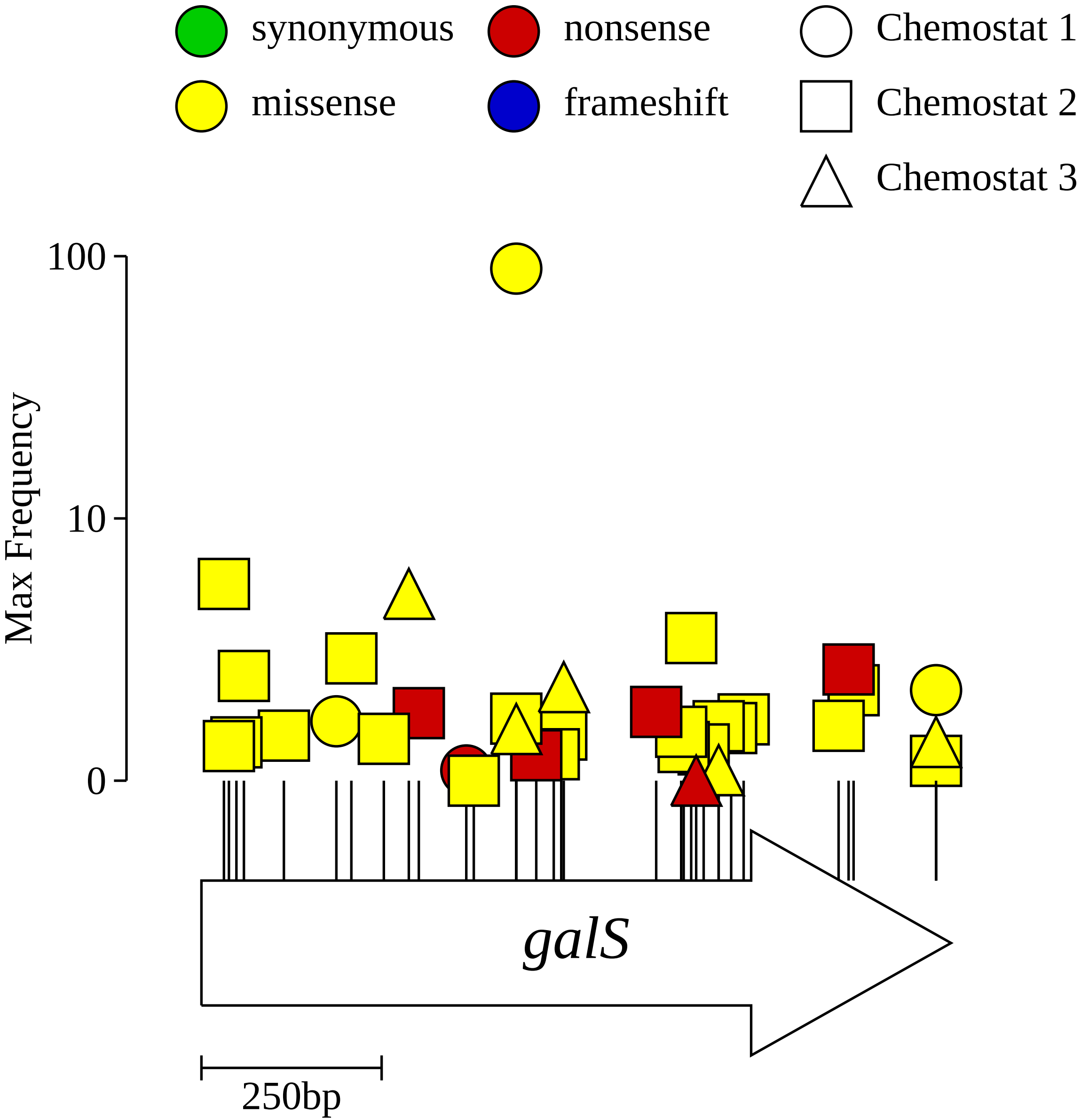
synonymous
nonsense
Chemostat 1
missense
frameshift
Chemostat 2
Chemostat 3
100
10
0
Max Frequency
galS
250bp

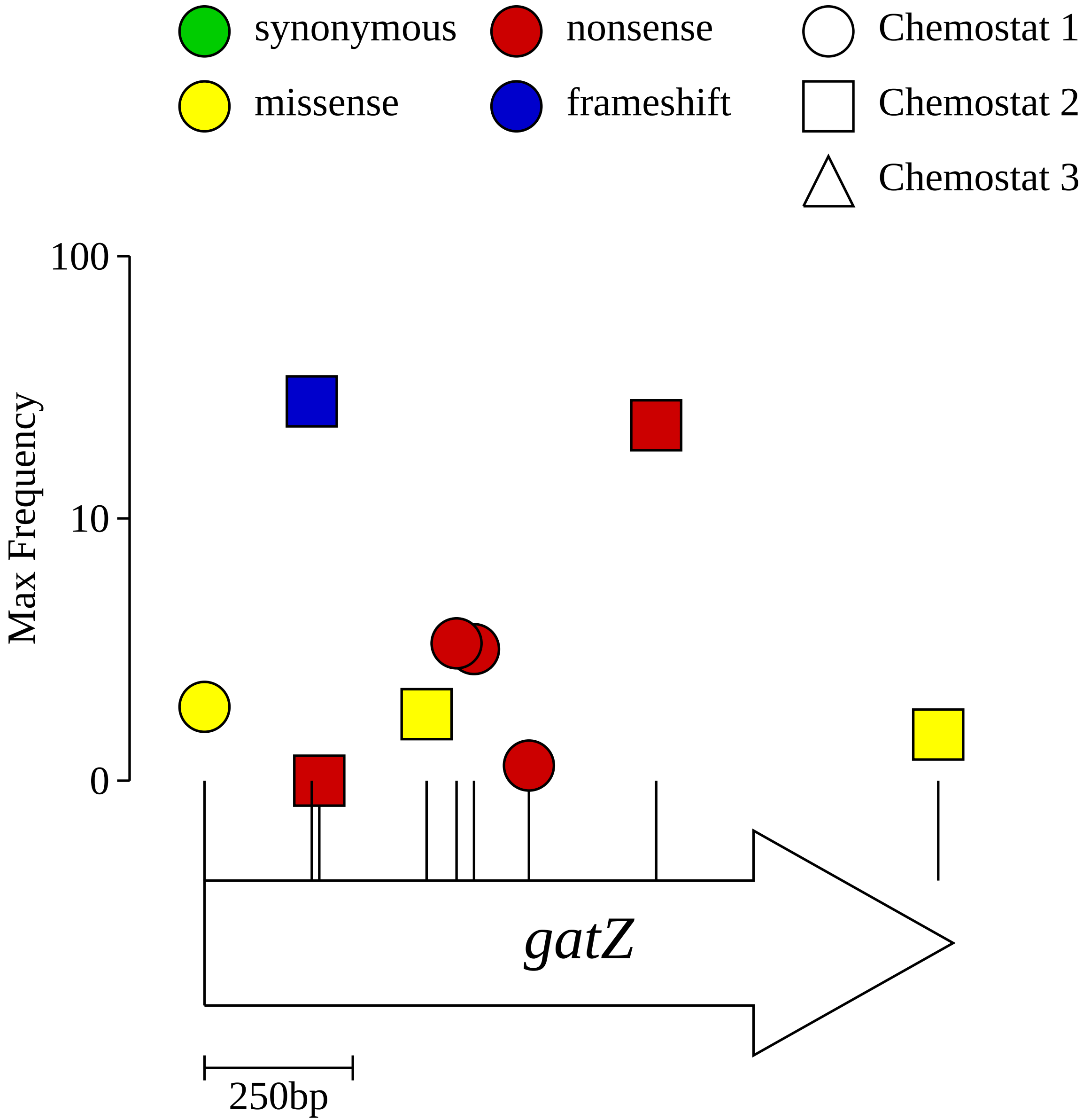
synonymous
nonsense
Chemostat 1
missense
frameshift
Chemostat 2
Chemostat 3
100
10
0
Max Frequency
gatZ
250bp

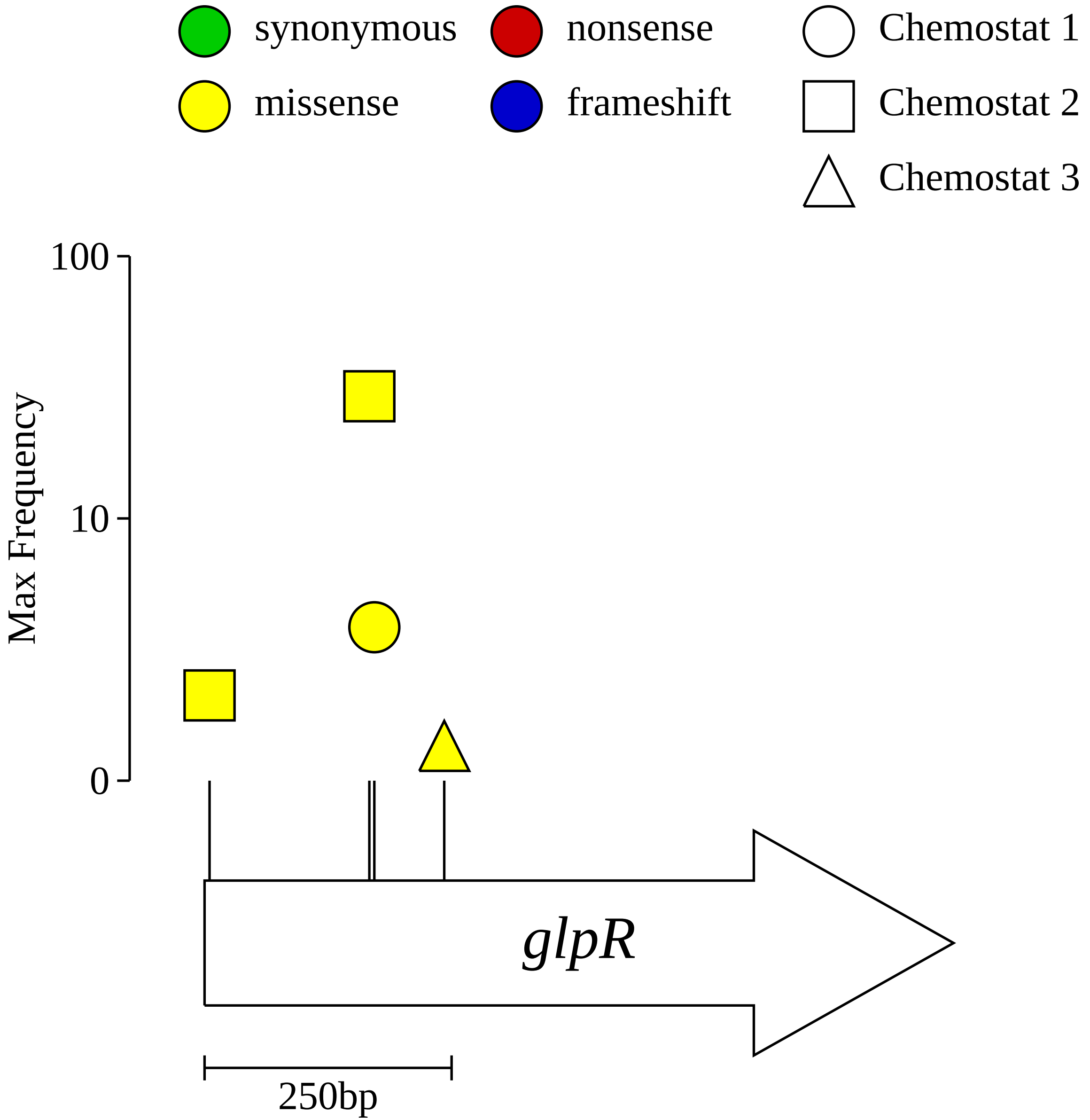
synonymous
nonsense
Chemostat 1
missense
frameshift
Chemostat 2
Chemostat 3
100
10
0
Max Frequency
glpR
250bp

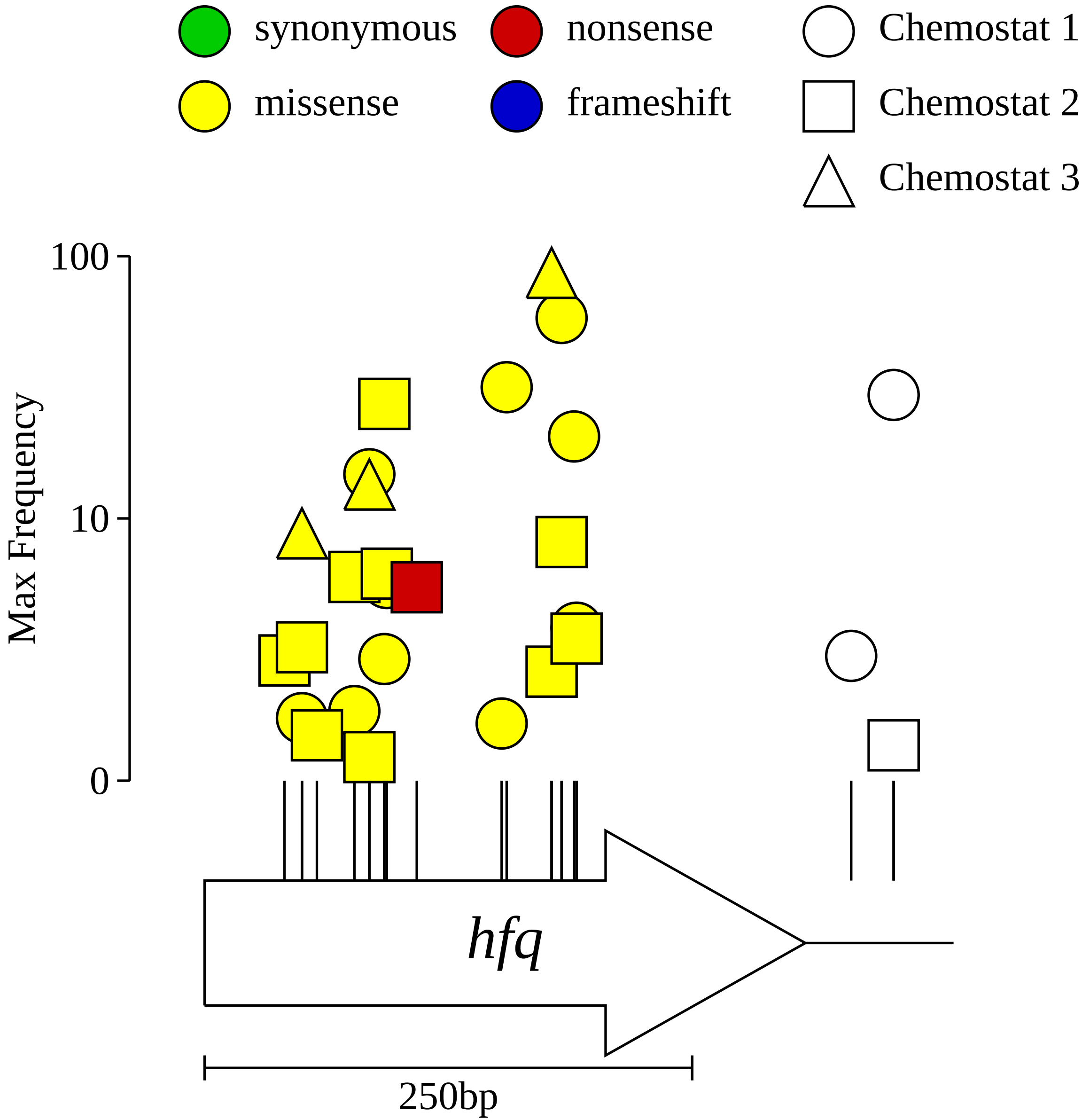
synonymous
nonsense
Chemostat 1
missense
frameshift
Chemostat 2
Chemostat 3
Max Frequency
100
10
0
hfq
250bp

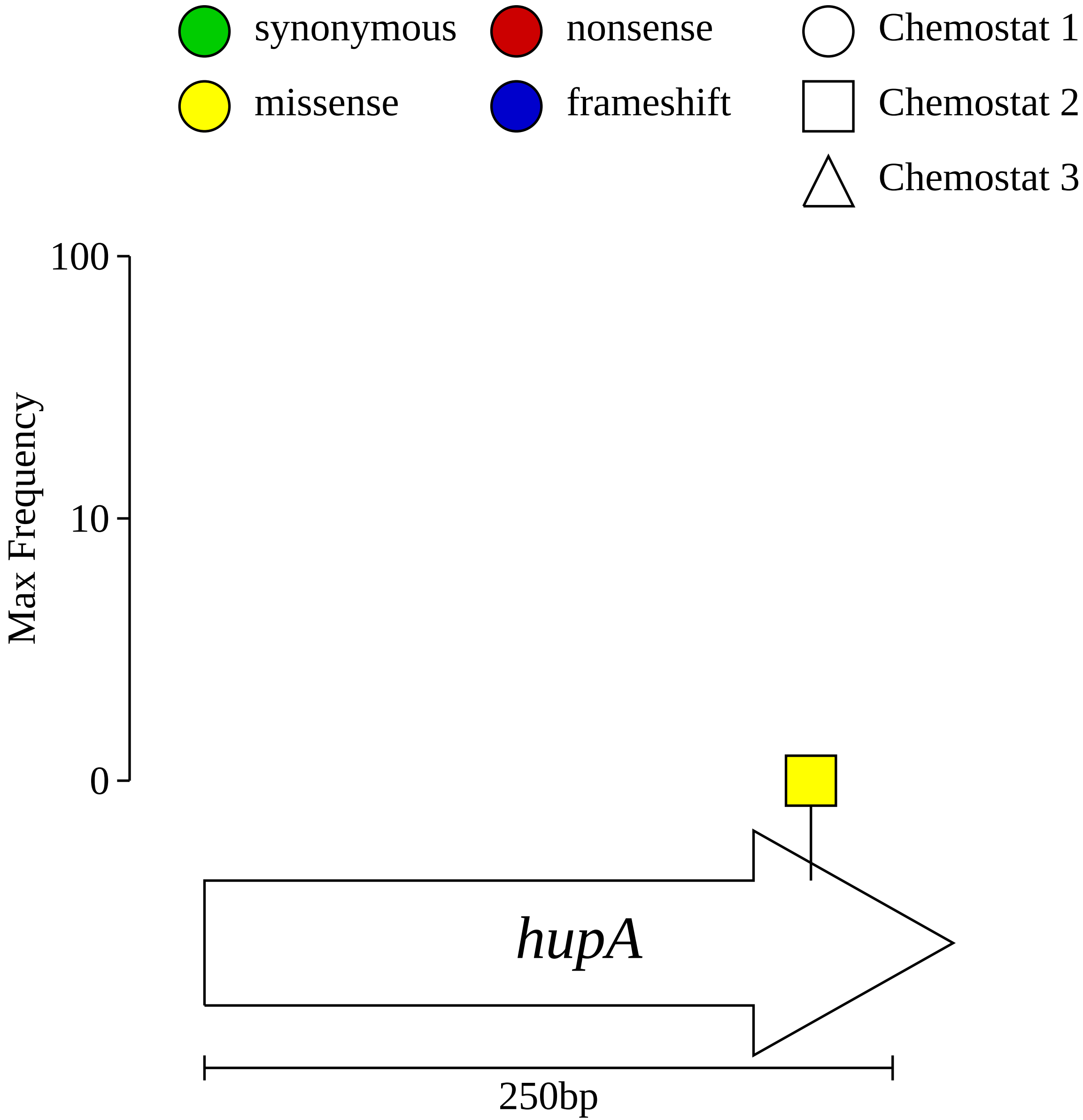
synonymous
nonsense
Chemostat 1
missense
frameshift
Chemostat 2
Chemostat 3
100
10
0
Max Frequency
hupA
250bp

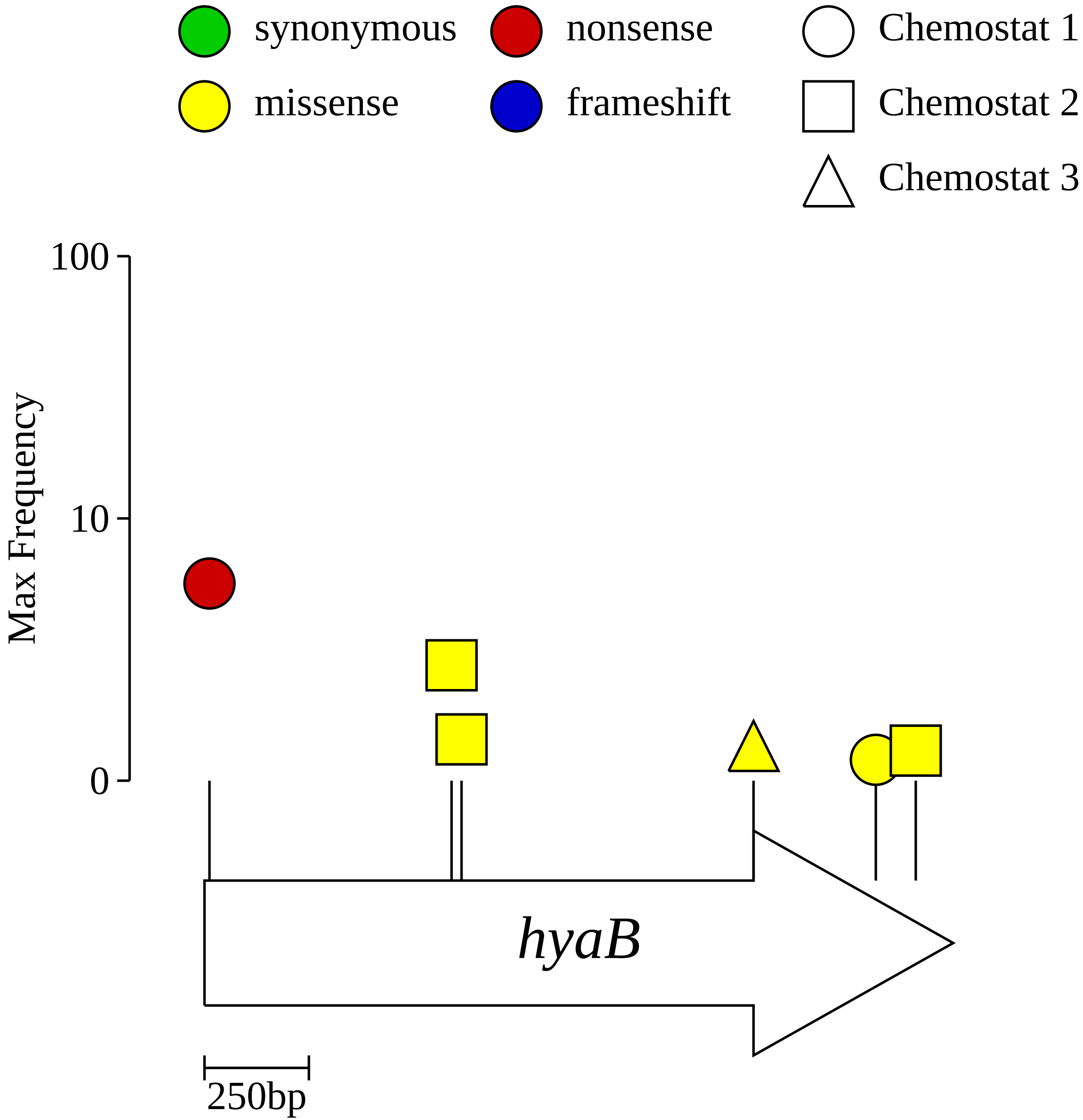
synonymous
nonsense
Chemostat 1
missense
frameshift
Chemostat 2
Chemostat 3
100
10
0
Max Frequency
hyaB
250bp

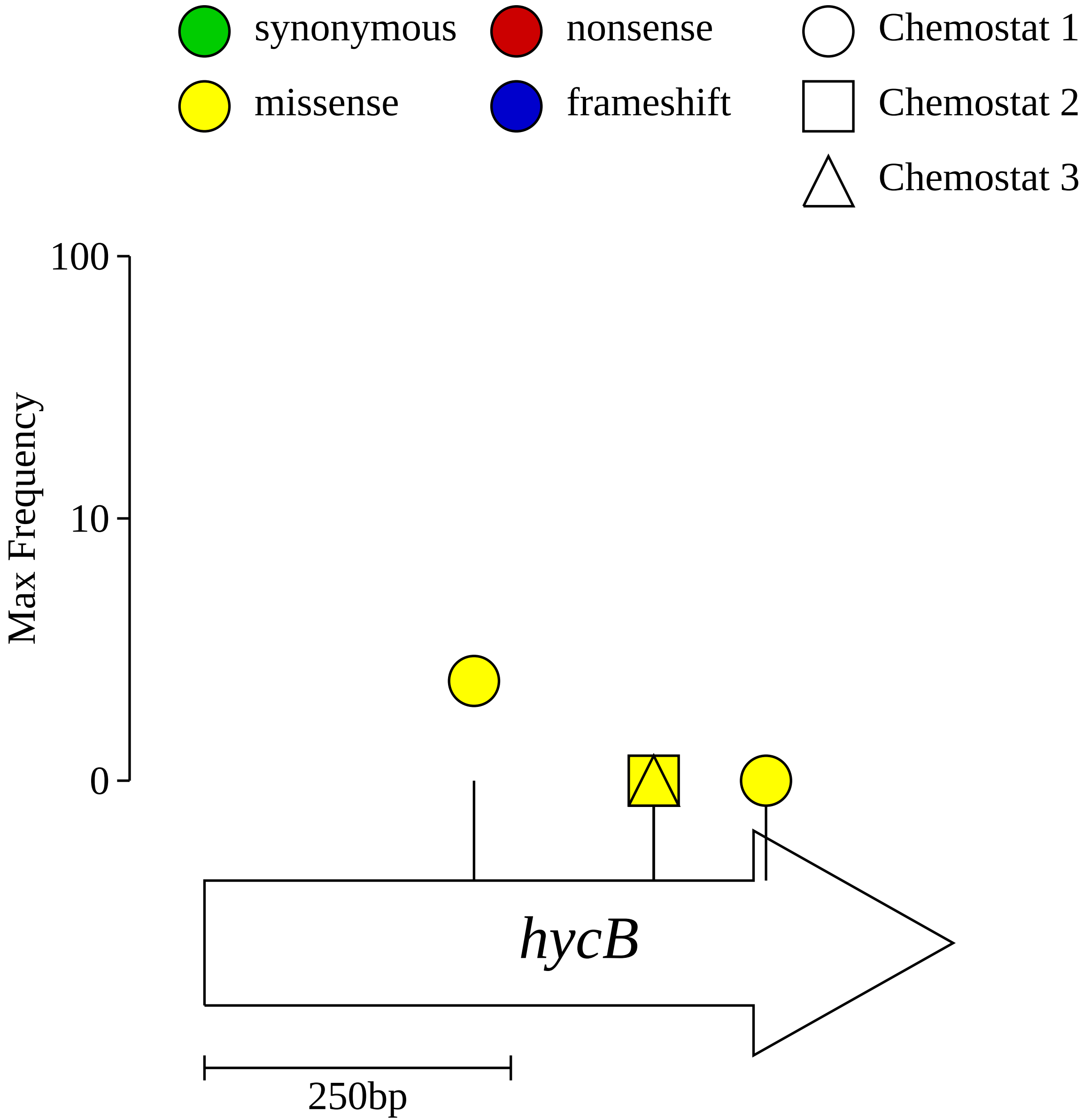

synonymous
nonsense
Chemostat 1
missense
frameshift
Chemostat 2
Chemostat 3
100
10
0
Max Frequency
hycB
250bp

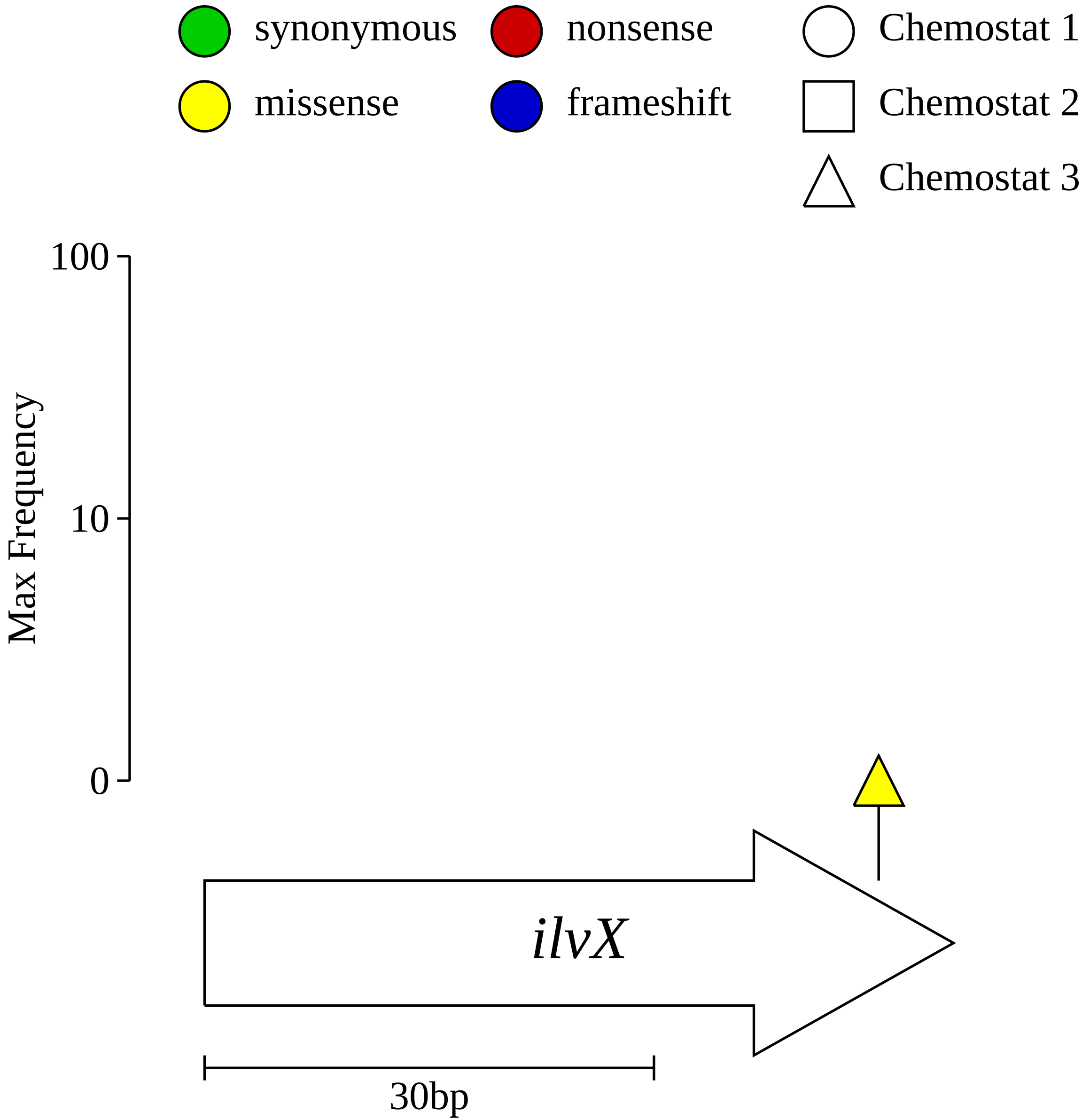
synonymous
missense
nonsense
frameshift
Chemostat 1
Chemostat 2
Chemostat 3
100
10
0
Max Frequency
ilvX
30bp

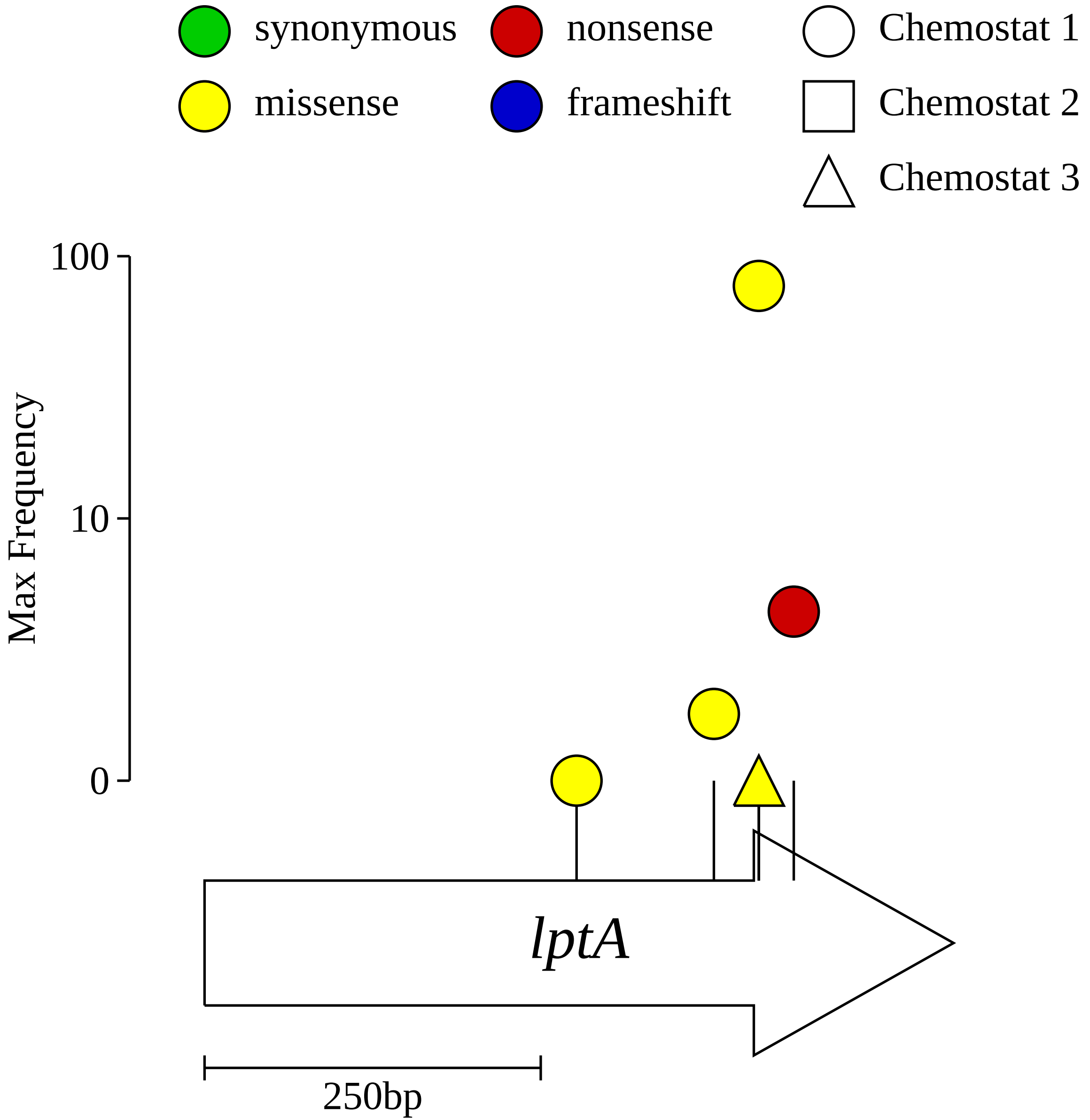
synonymous
nonsense
Chemostat 1
missense
frameshift
Chemostat 2
Chemostat 3
100
10
0
Max Frequency
lptA
250bp

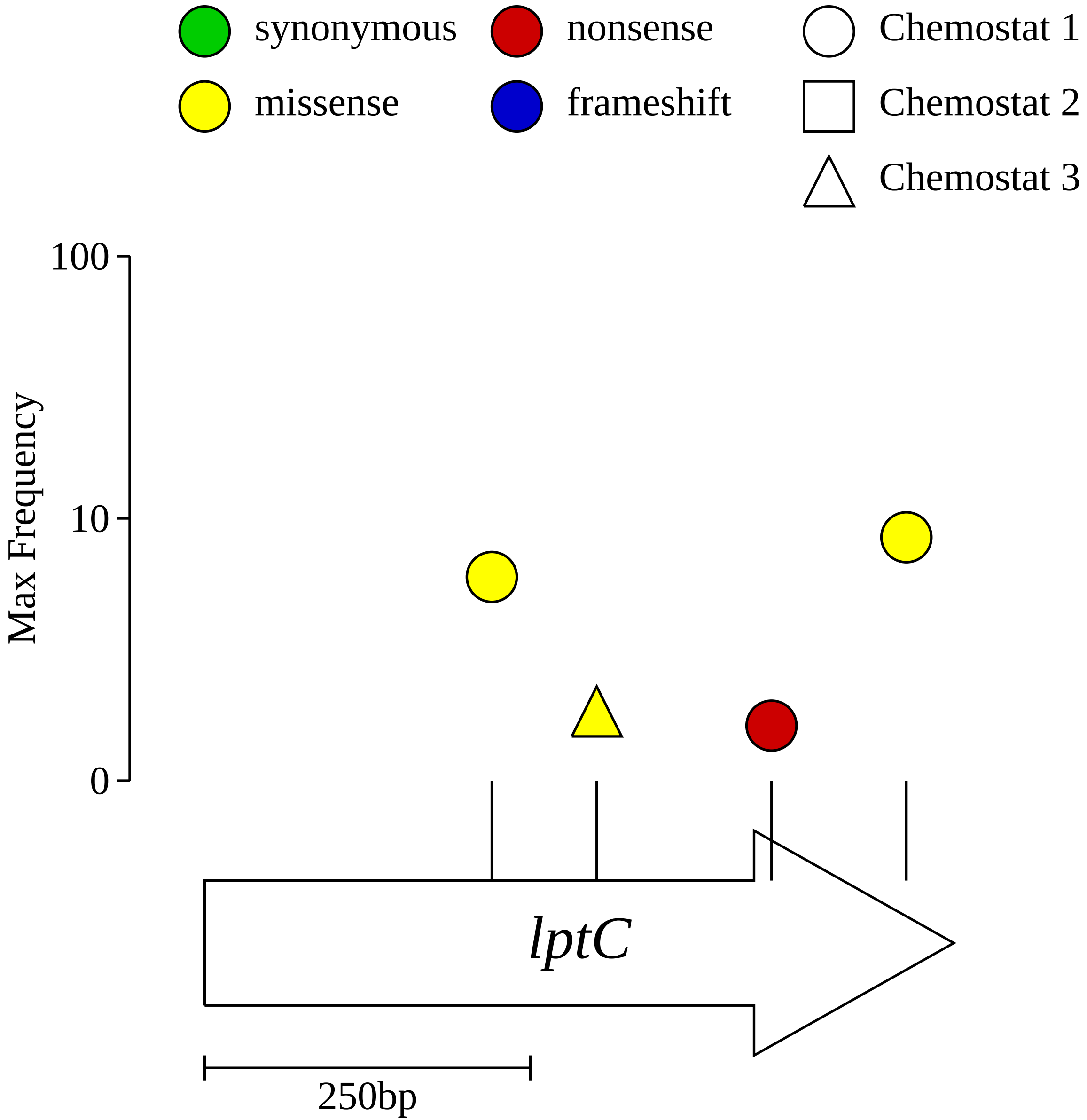

synonymous
nonsense
Chemostat 1
missense
frameshift
Chemostat 2
Chemostat 3
100
10
0
Max Frequency
lptC
250bp

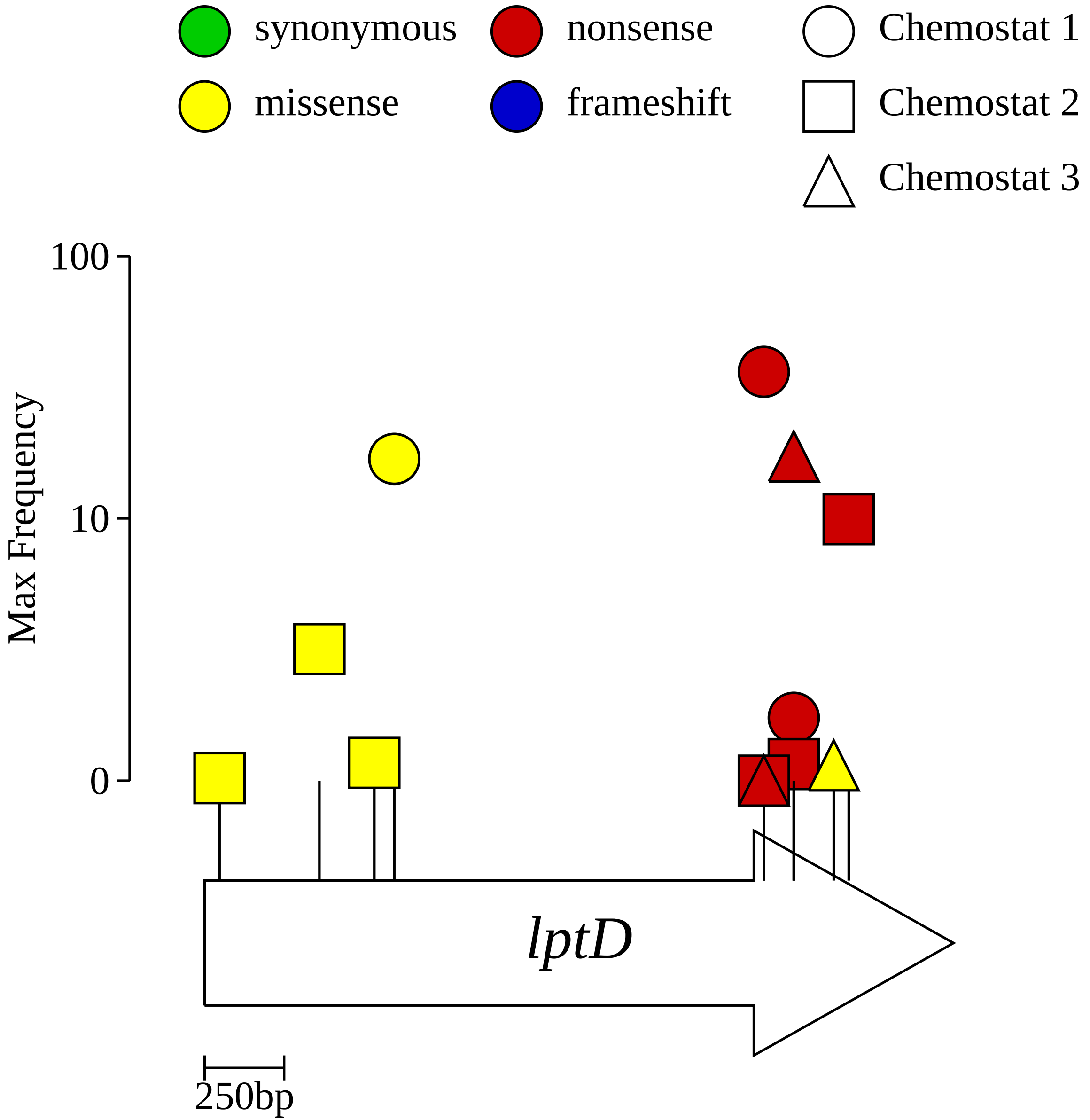
synonymous
nonsense
Chemostat 1
missense
frameshift
Chemostat 2
Chemostat 3
100
10
0
Max Frequency
lptD
250bp

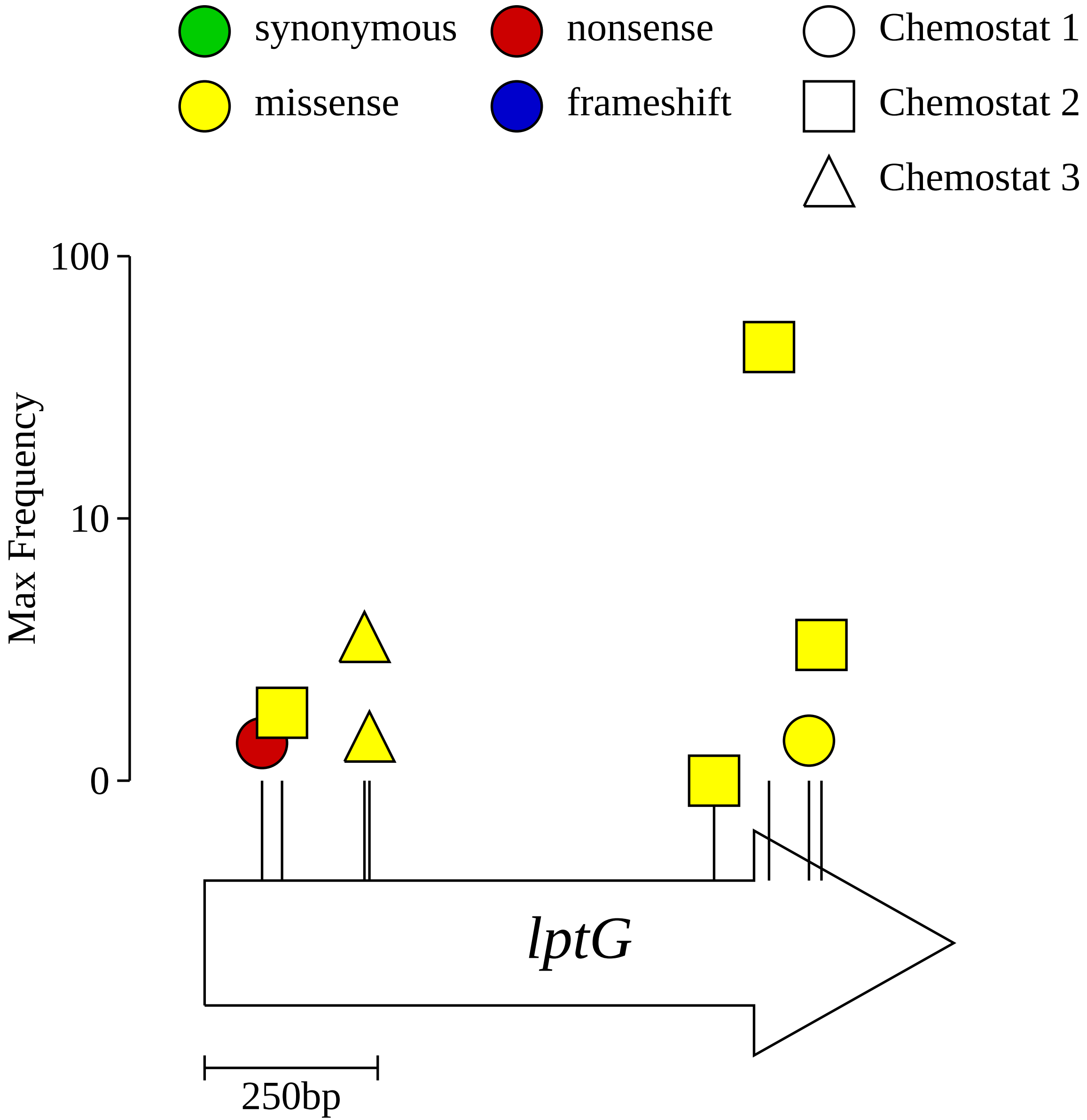

synonymous
nonsense
Chemostat 1
missense
frameshift
Chemostat 2
Chemostat 3
100
10
0
Max Frequency
lptG
250bp

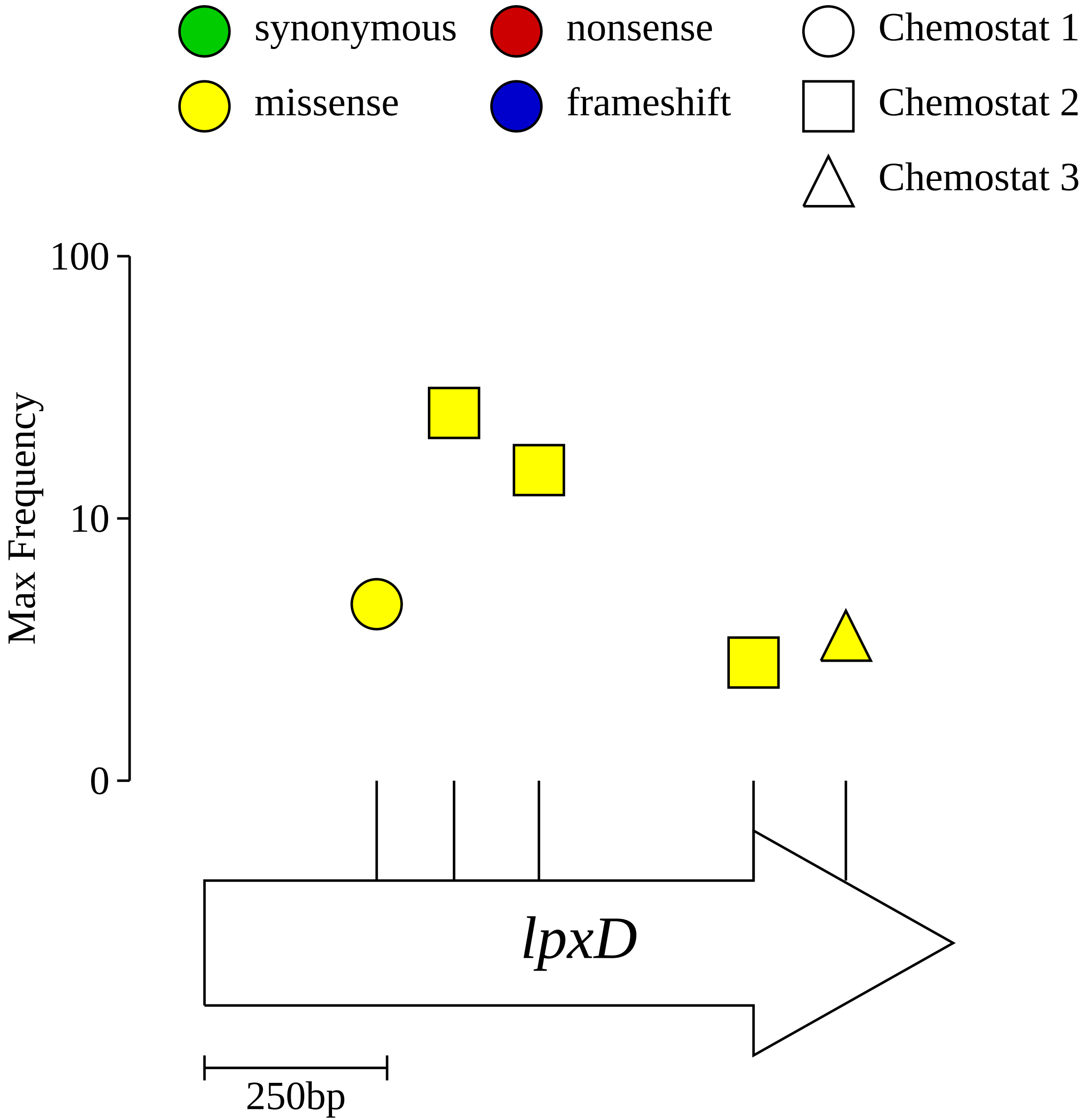
synonymous
missense
nonsense
frameshift
Chemostat 1
Chemostat 2
Chemostat 3
100
10
0
Max Frequency
lpxD
250bp

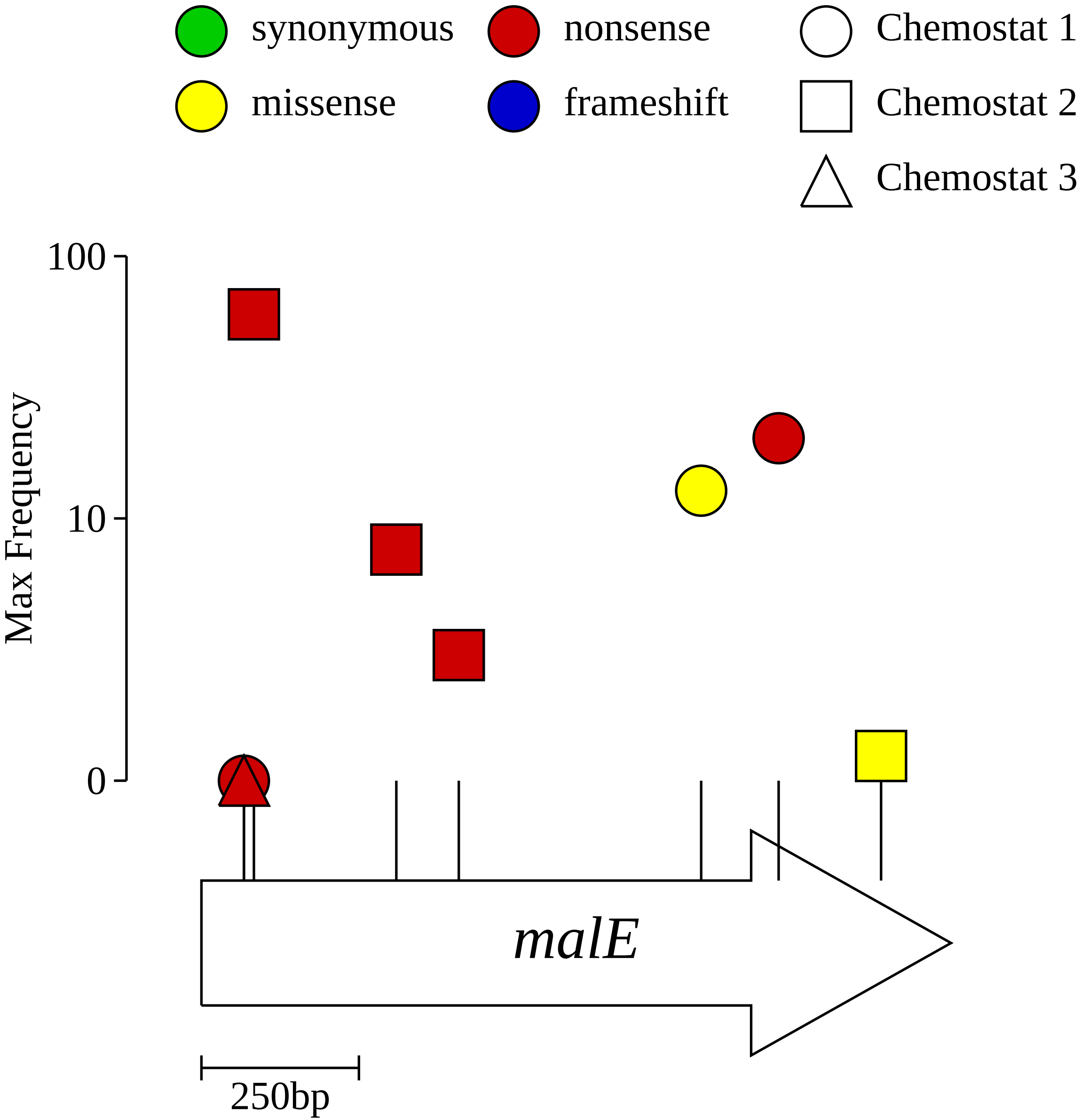

synonymous
missense
nonsense
frameshift
Chemostat 1
Chemostat 2
Chemostat 3
100
10
0
Max Frequency
malE
250bp

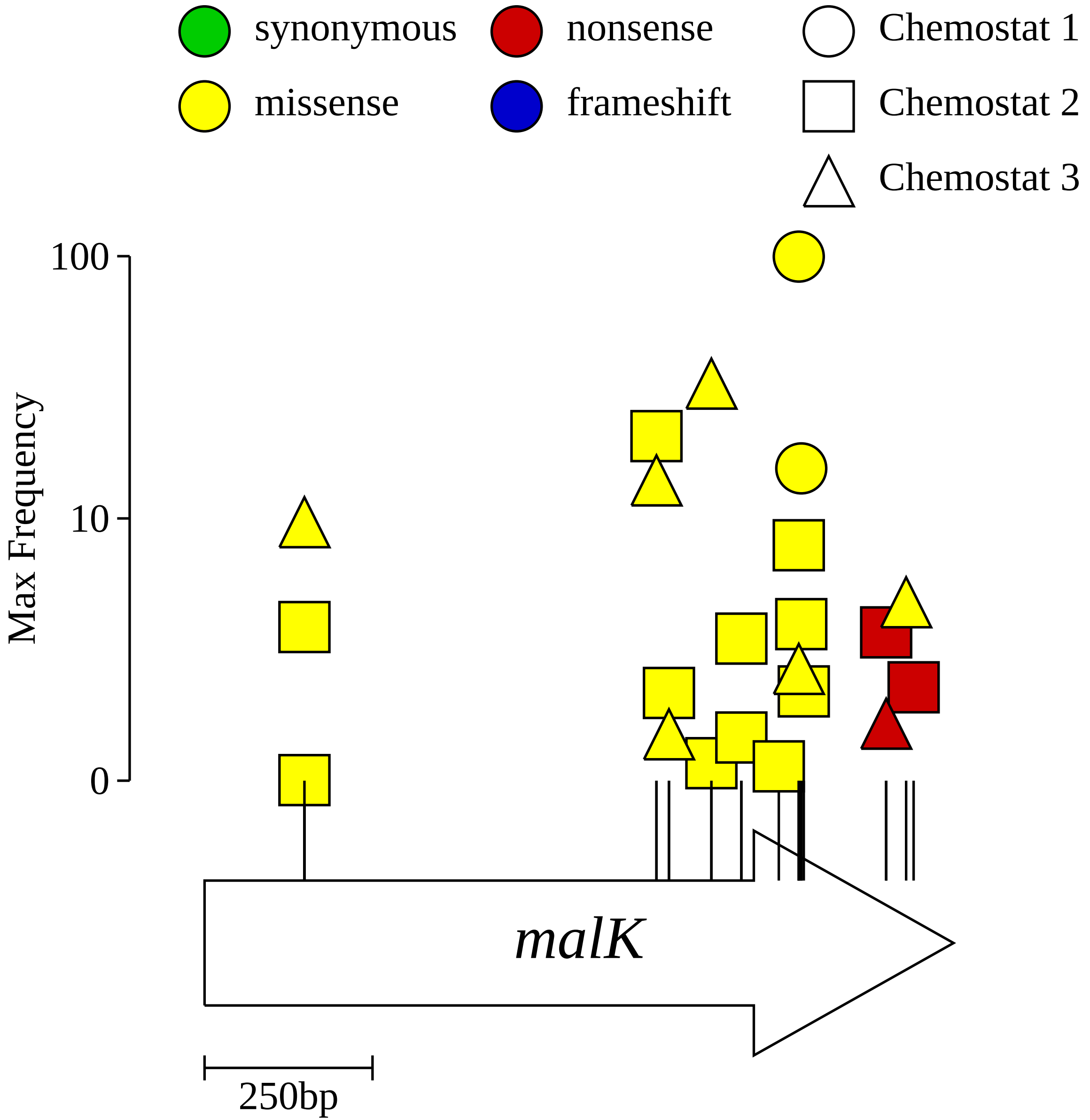
synonymous
missense
nonsense
frameshift
Chemostat 1
Chemostat 2
Chemostat 3
Max Frequency
100
10
0
malK
250bp

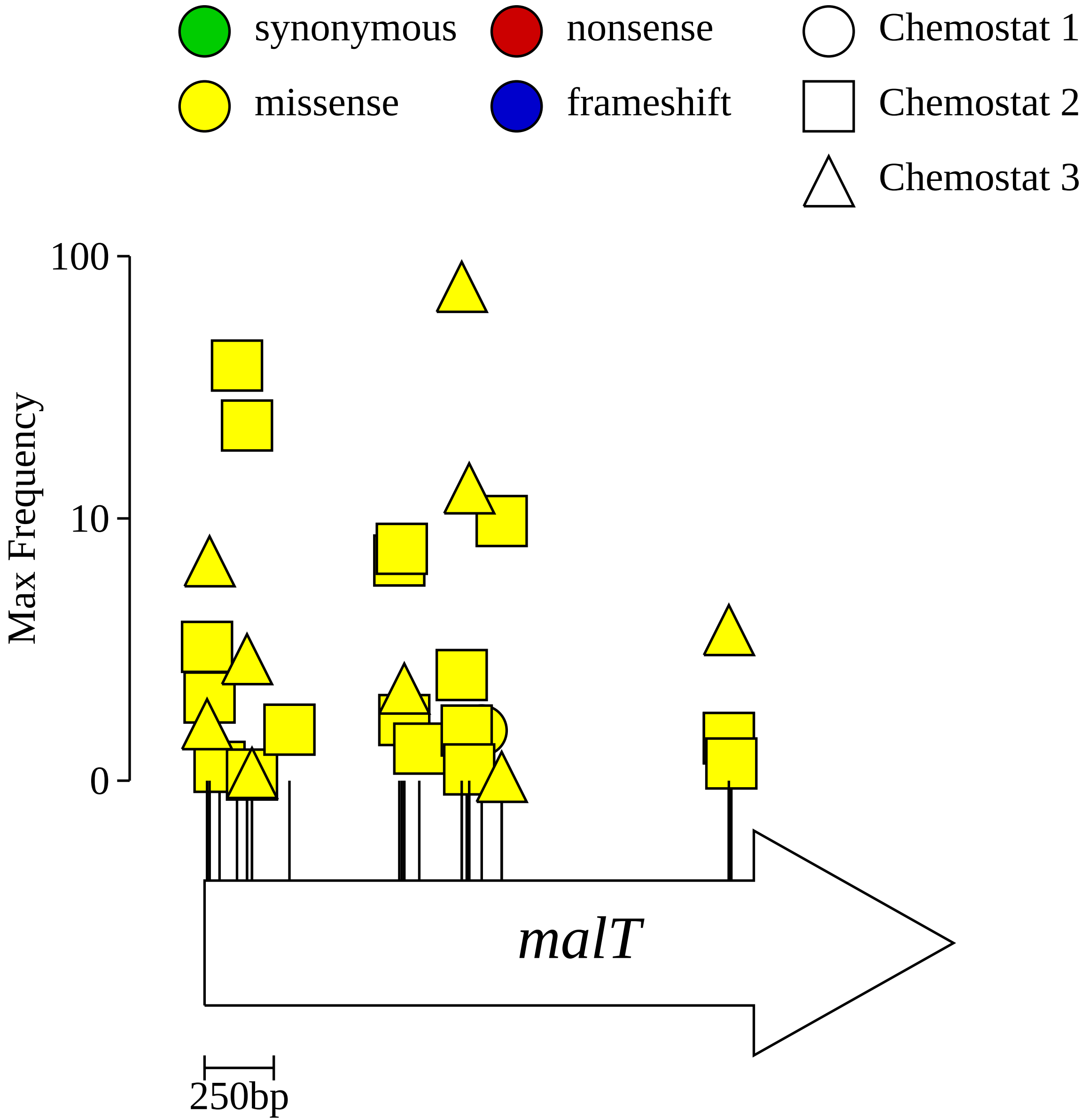
synonymous
nonsense
Chemostat 1
missense
frameshift
Chemostat 2
Chemostat 3
100
10
0
Max Frequency
malT
250bp

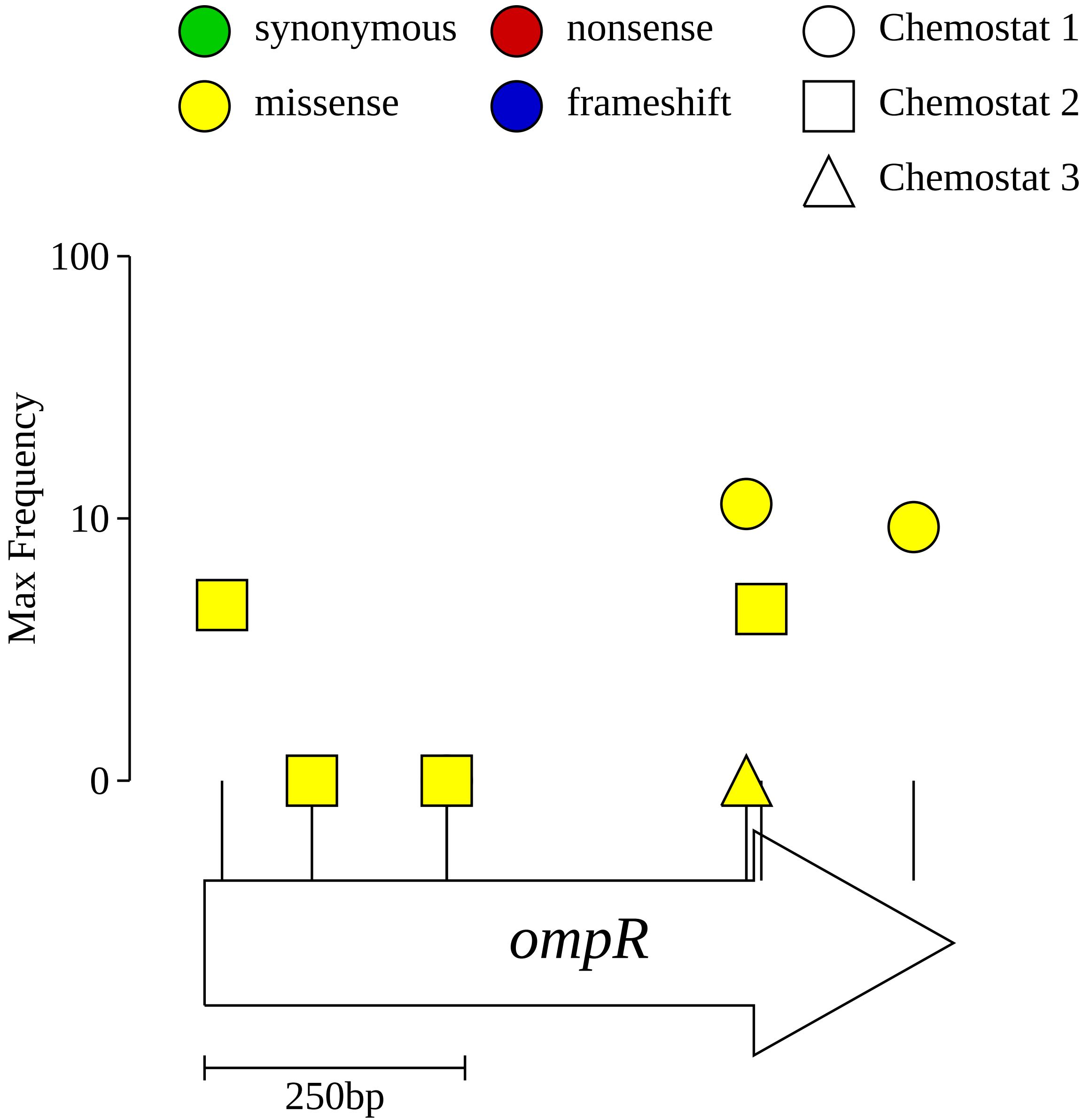

synonymous
nonsense
Chemostat 1
missense
frameshift
Chemostat 2
Chemostat 3
100
10
0
Max Frequency
ompR
250bp

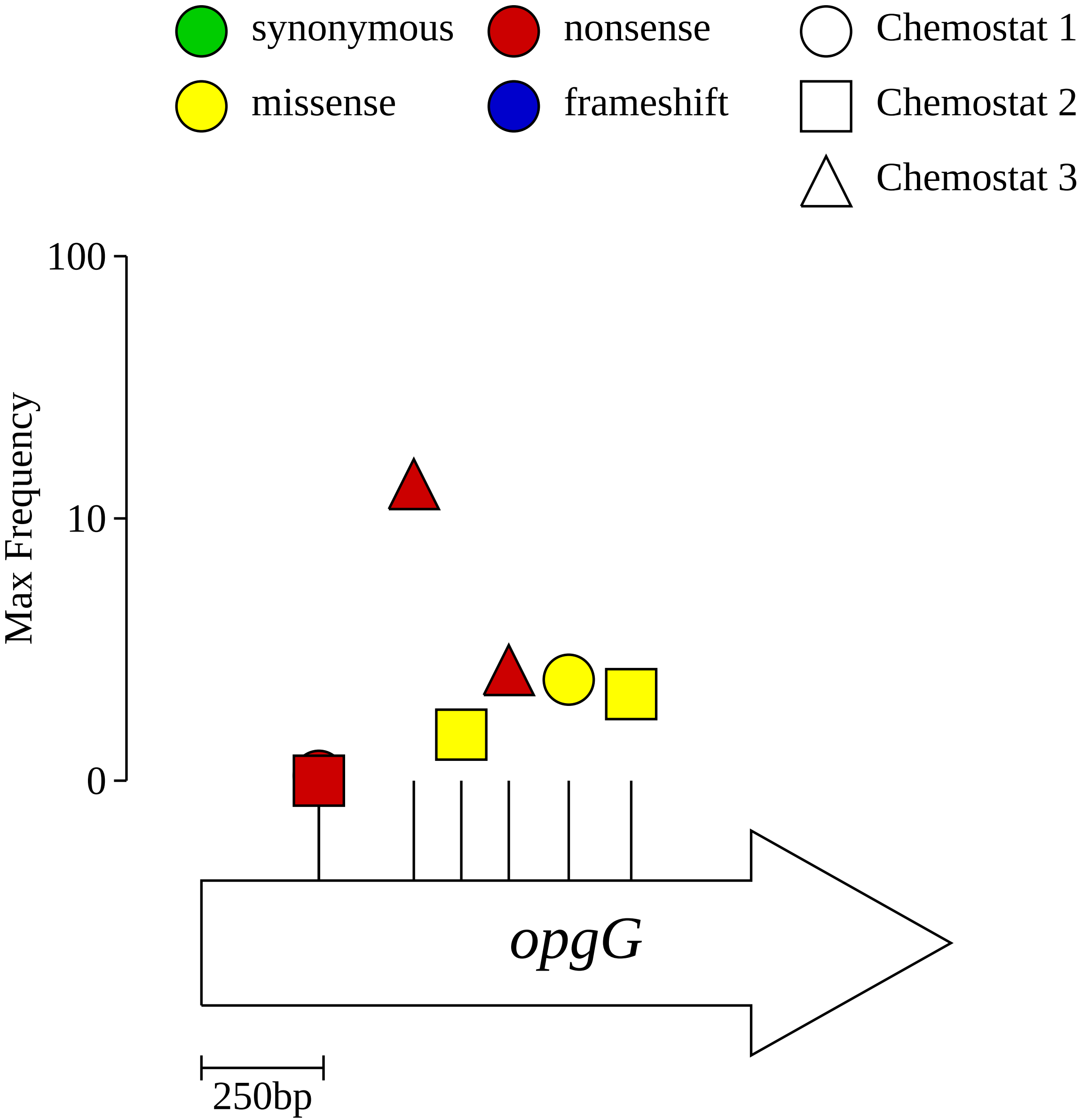

synonymous
nonsense
Chemostat 1
missense
frameshift
Chemostat 2
Chemostat 3
100
10
0
Max Frequency
opgG
250bp

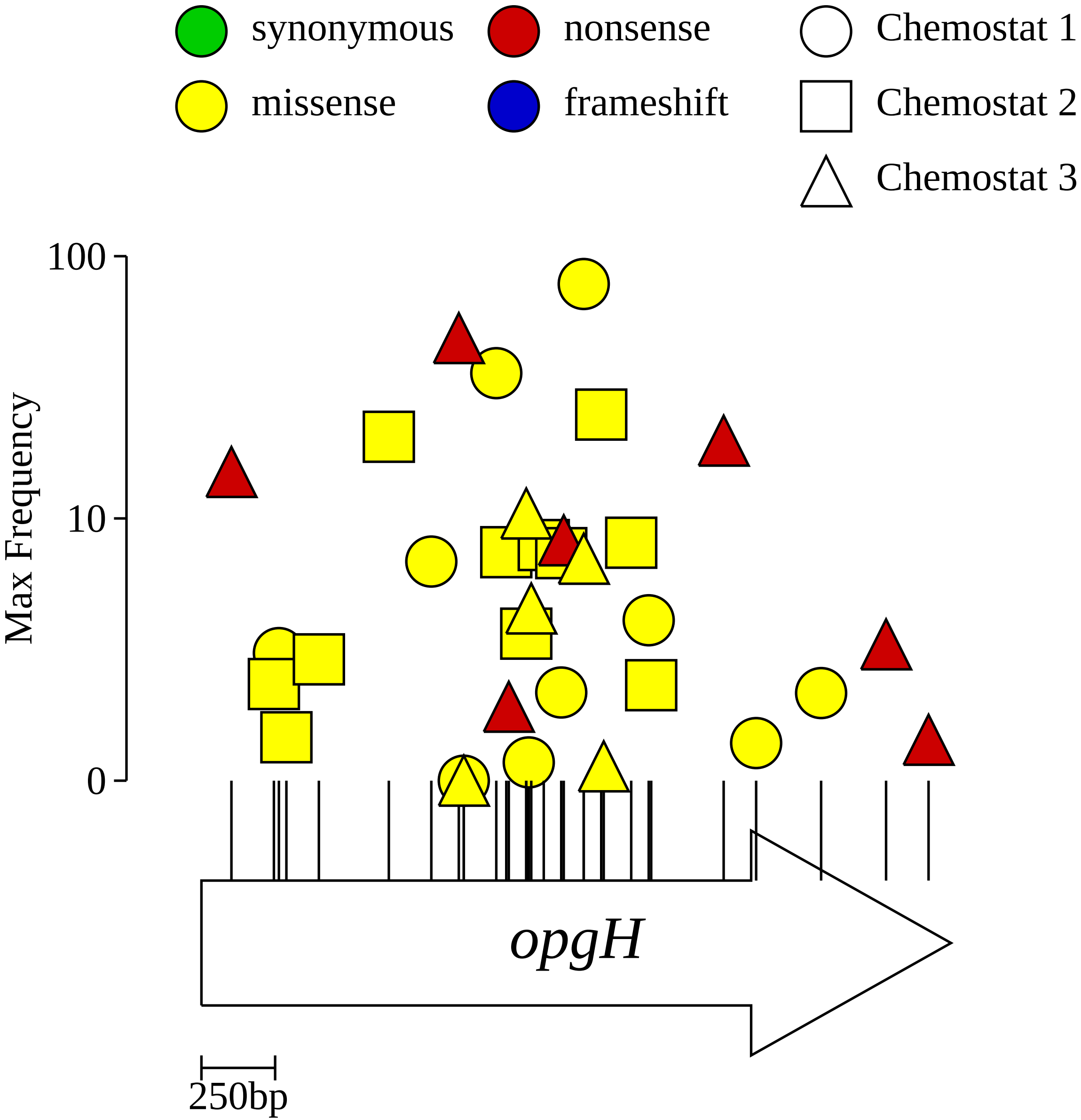
synonymous
nonsense
Chemostat 1
missense
frameshift
Chemostat 2
Chemostat 3
100
10
0
Max Frequency
opgH
250bp

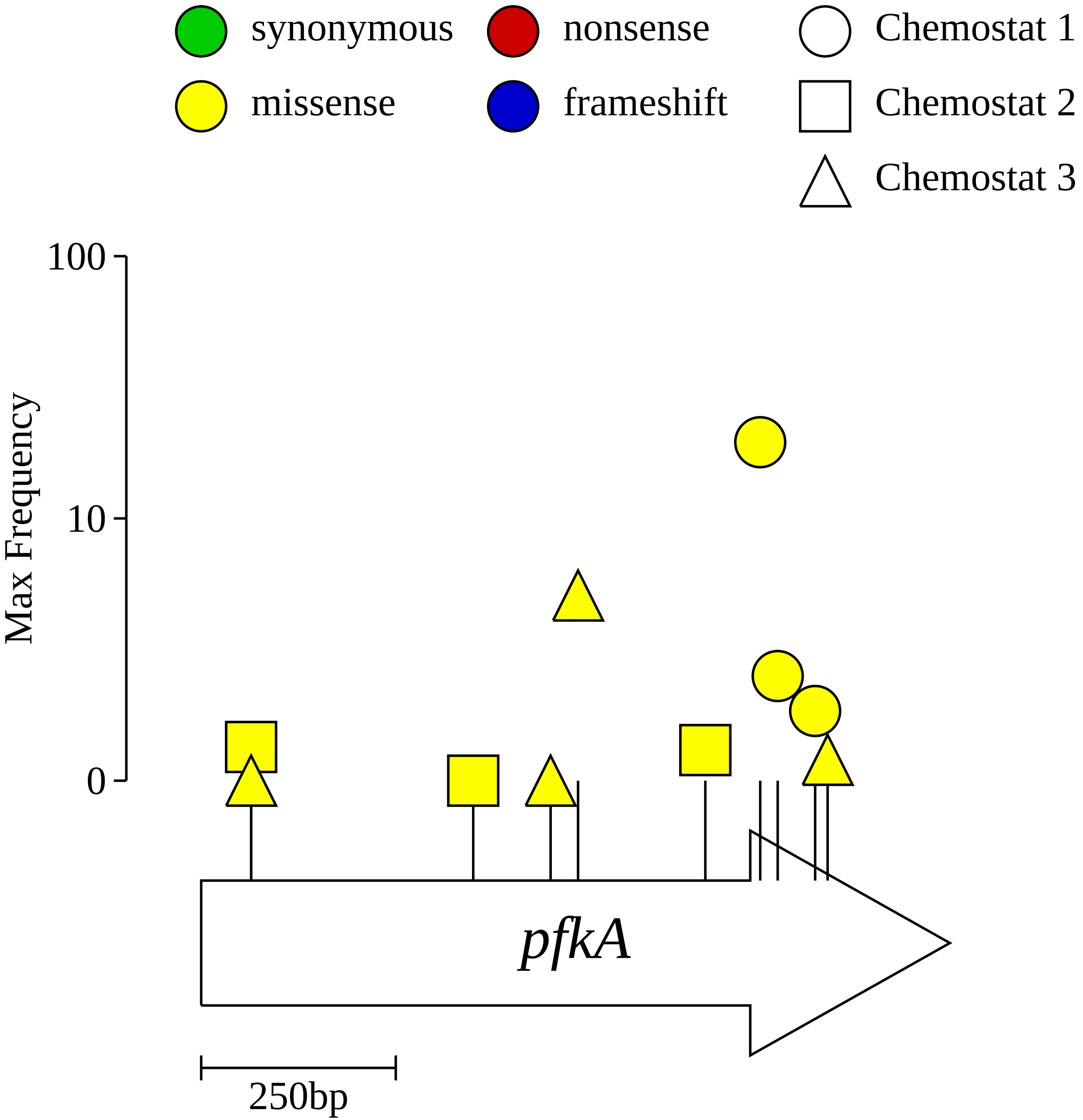

synonymous
nonsense
Chemostat 1
missense
frameshift
Chemostat 2
Chemostat 3
100
10
0
Max Frequency
pfkA
250bp

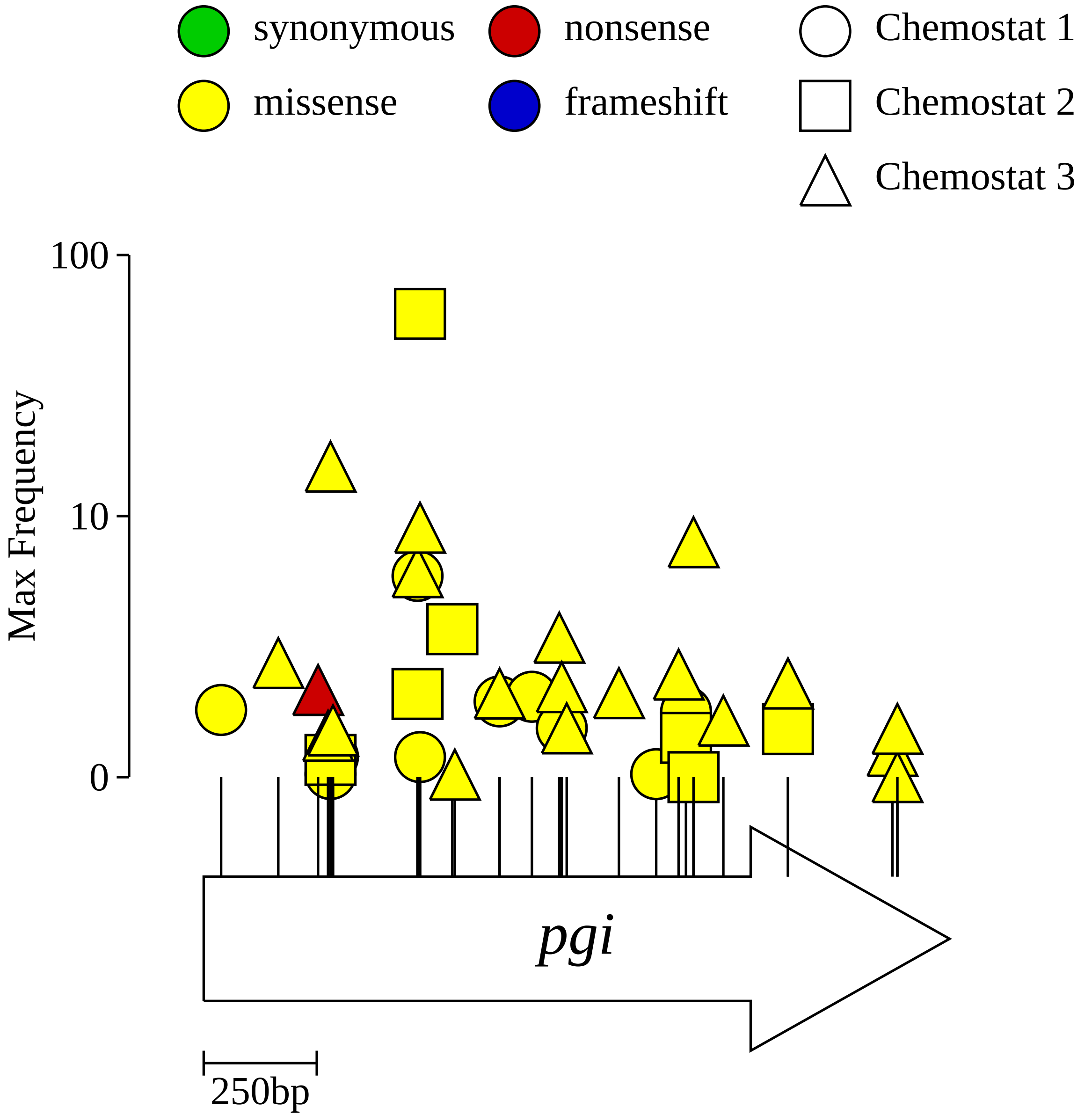
synonymous
nonsense
Chemostat 1
missense
frameshift
Chemostat 2
Chemostat 3
100
10
0
Max Frequency
pgi
250bp

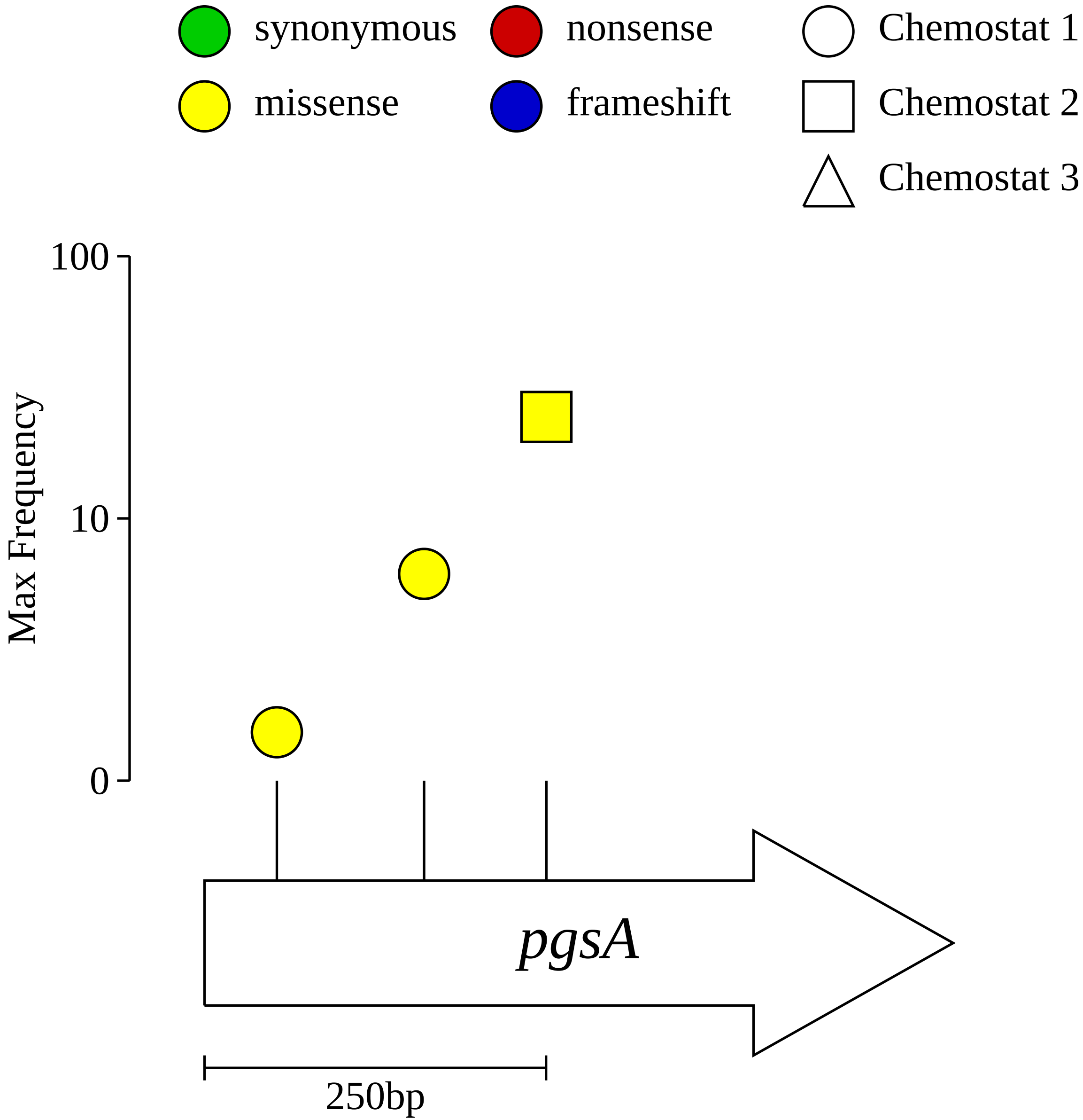
synonymous
nonsense
Chemostat 1
missense
frameshift
Chemostat 2
Chemostat 3
100
10
0
Max Frequency
pgsA
250bp

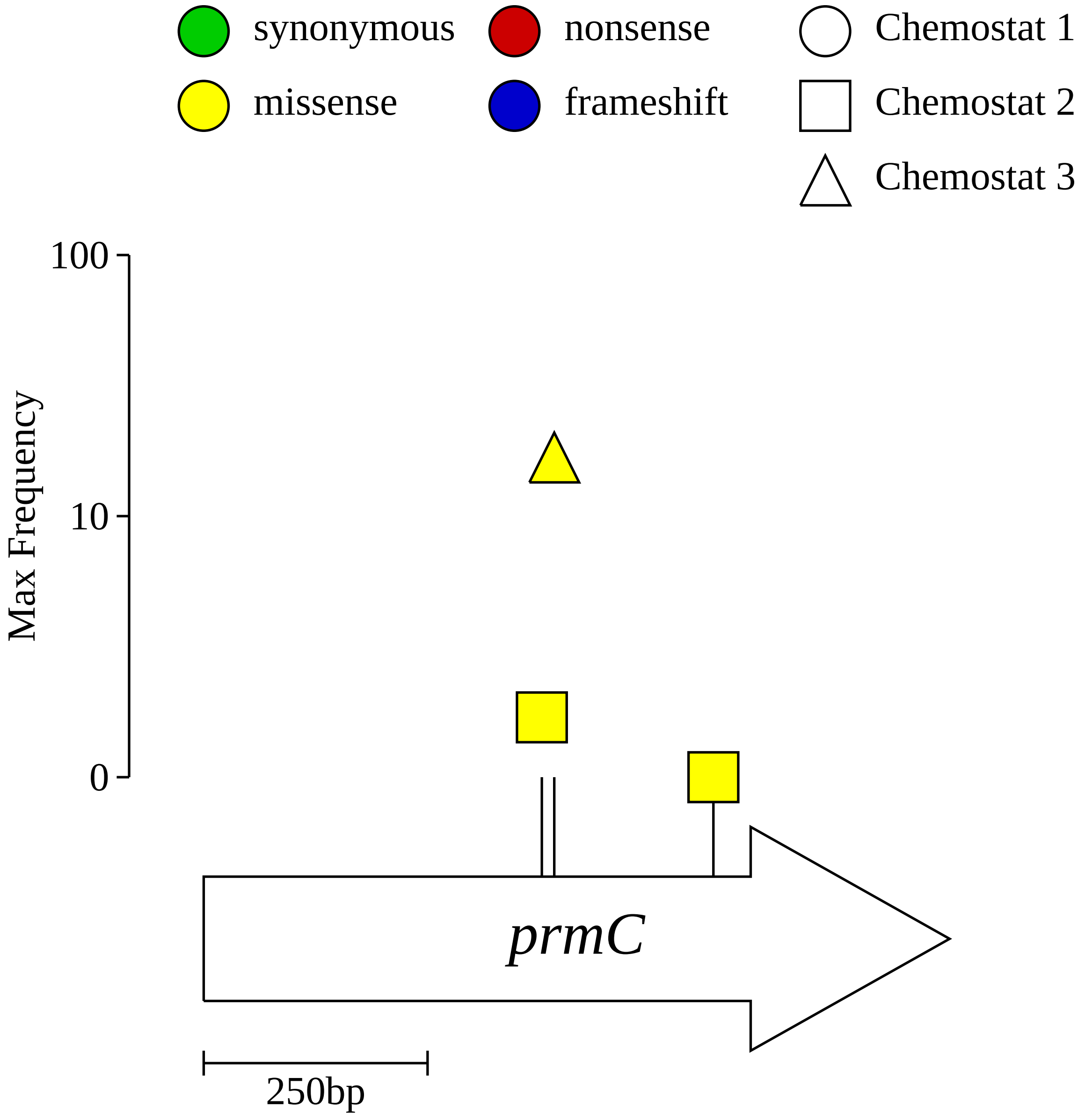
synonymous
nonsense
Chemostat 1
missense
frameshift
Chemostat 2
Chemostat 3
100
10
0
Max Frequency
prmC
250bp

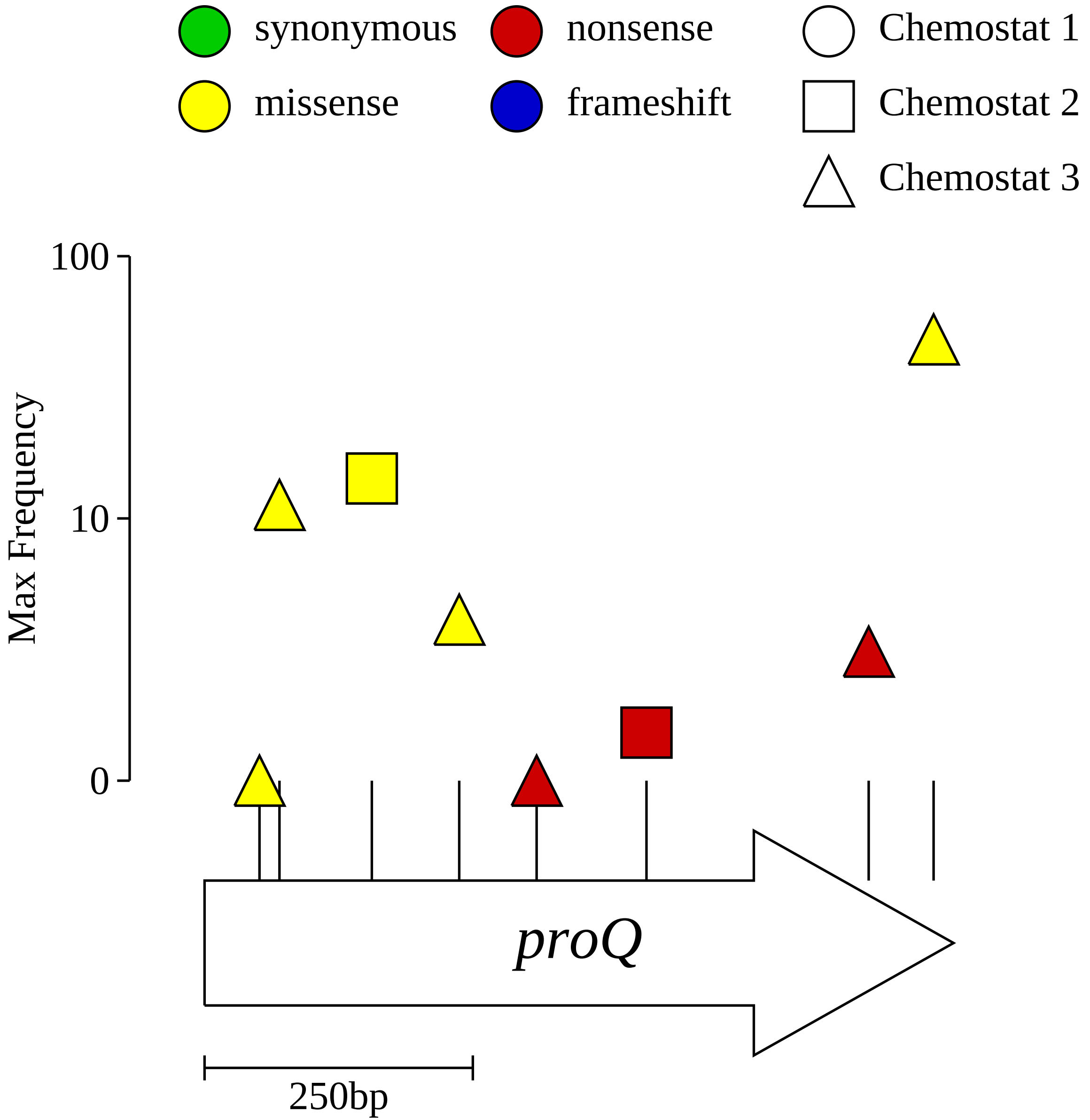

synonymous
missense
nonsense
frameshift
Chemostat 1
Chemostat 2
Chemostat 3
100
10
0
Max Frequency
proQ
250bp

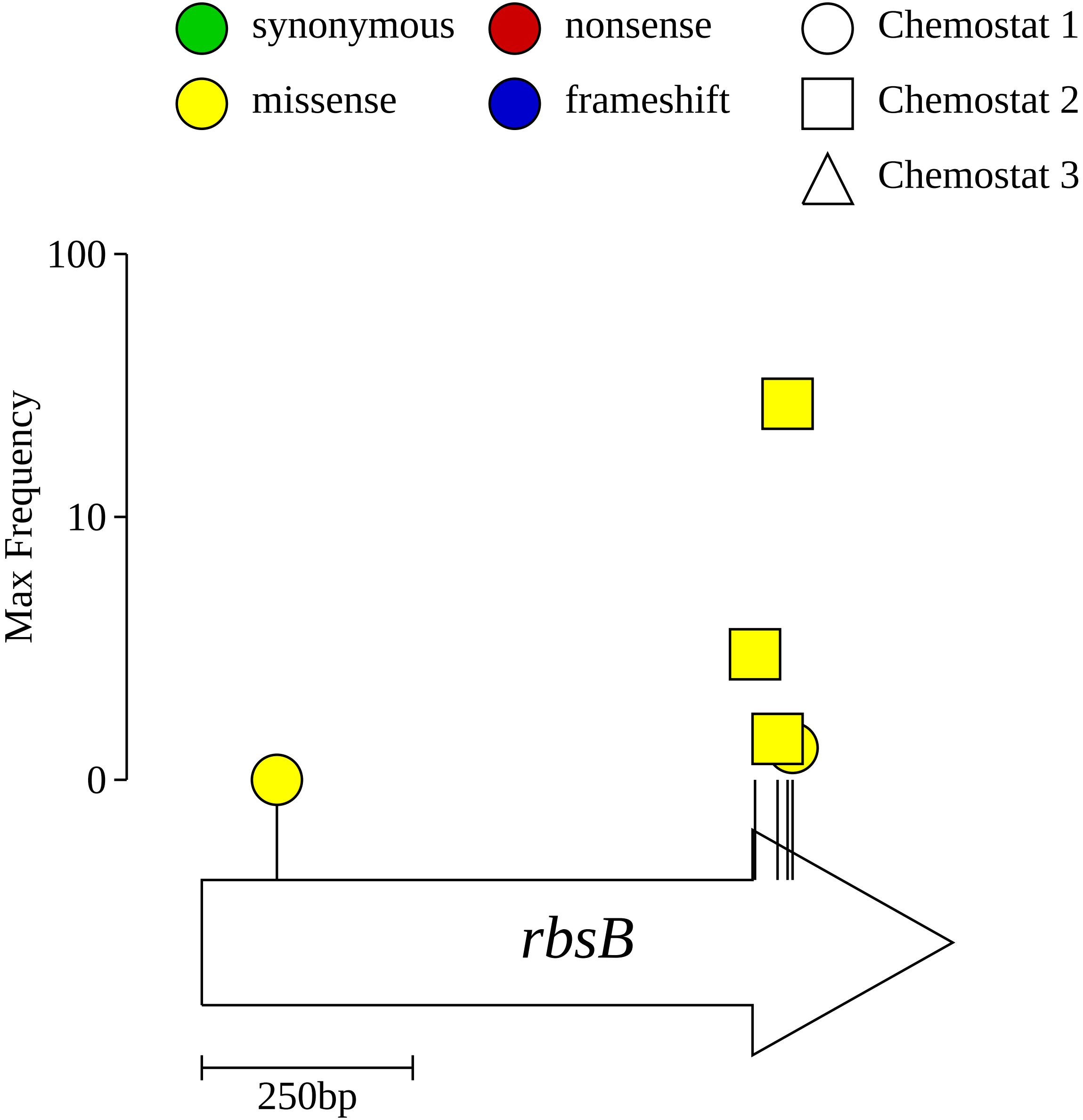
synonymous
nonsense
Chemostat 1
missense
frameshift
Chemostat 2
Chemostat 3
100
10
0
Max Frequency
rbsB
250bp

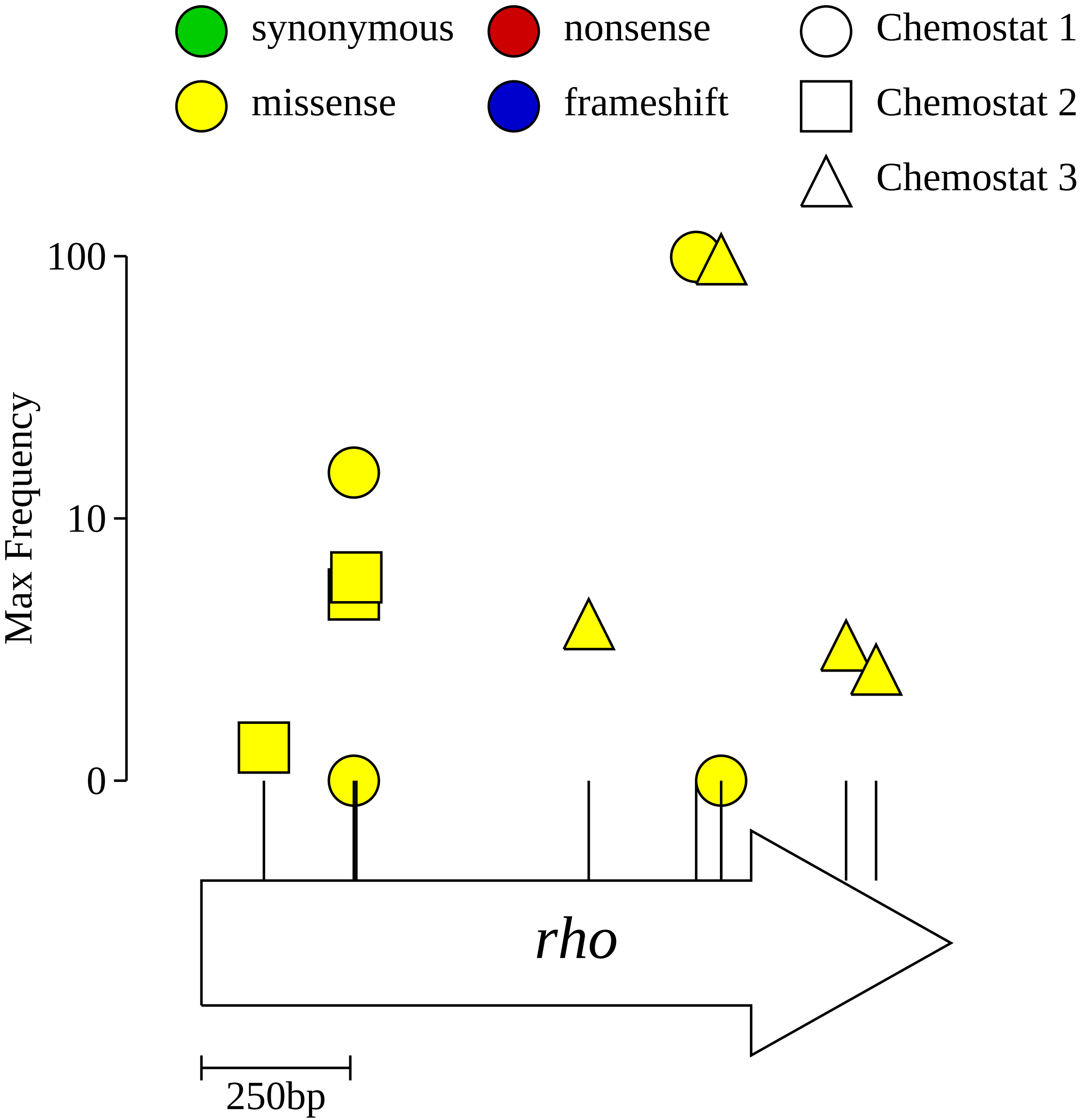
synonymous
nonsense
Chemostat 1
missense
frameshift
Chemostat 2
Chemostat 3
100
10
0
Max Frequency
rho
250bp

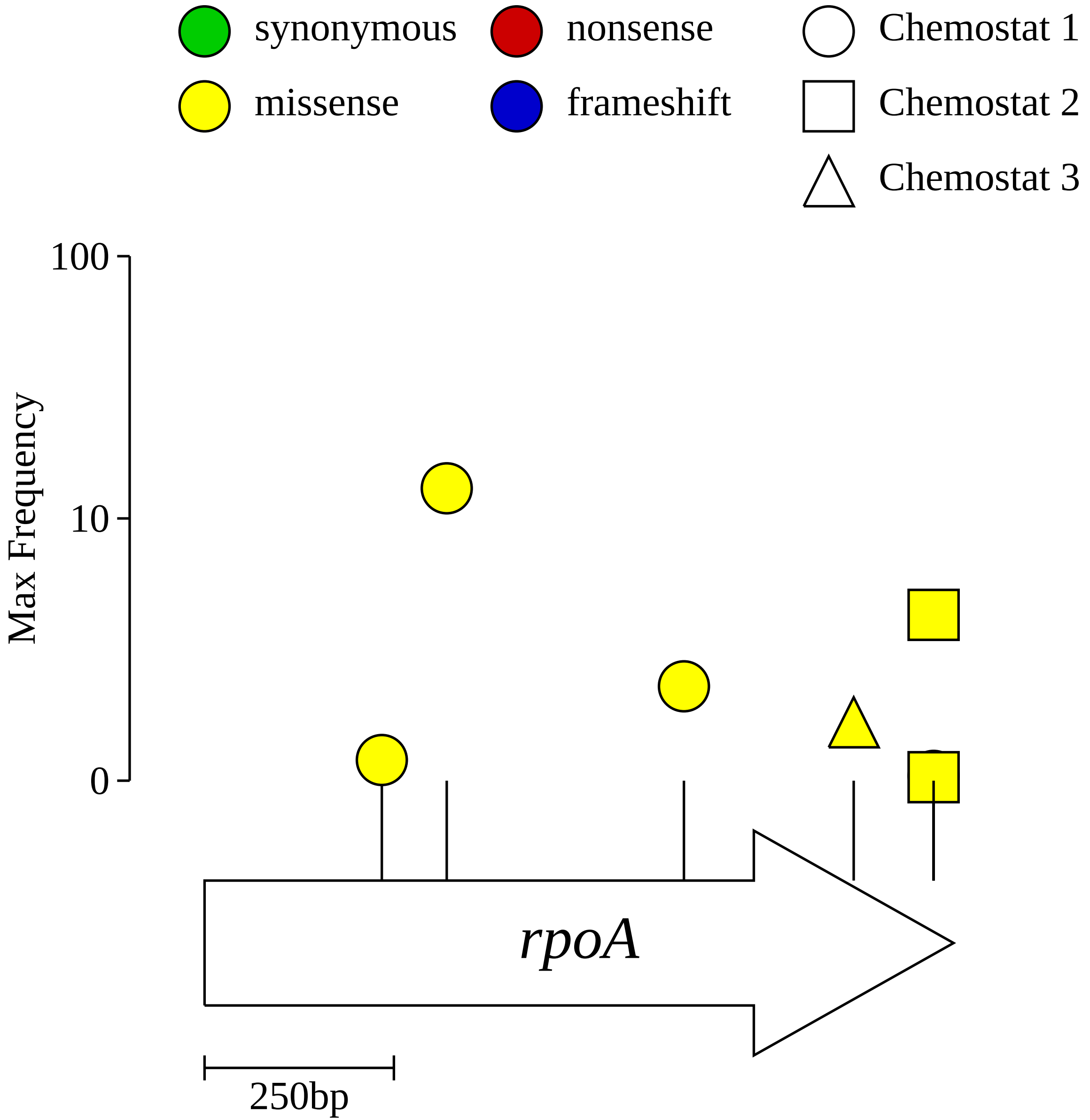

synonymous
nonsense
Chemostat 1
missense
frameshift
Chemostat 2
Chemostat 3
100
10
0
Max Frequency
rpoA
250bp

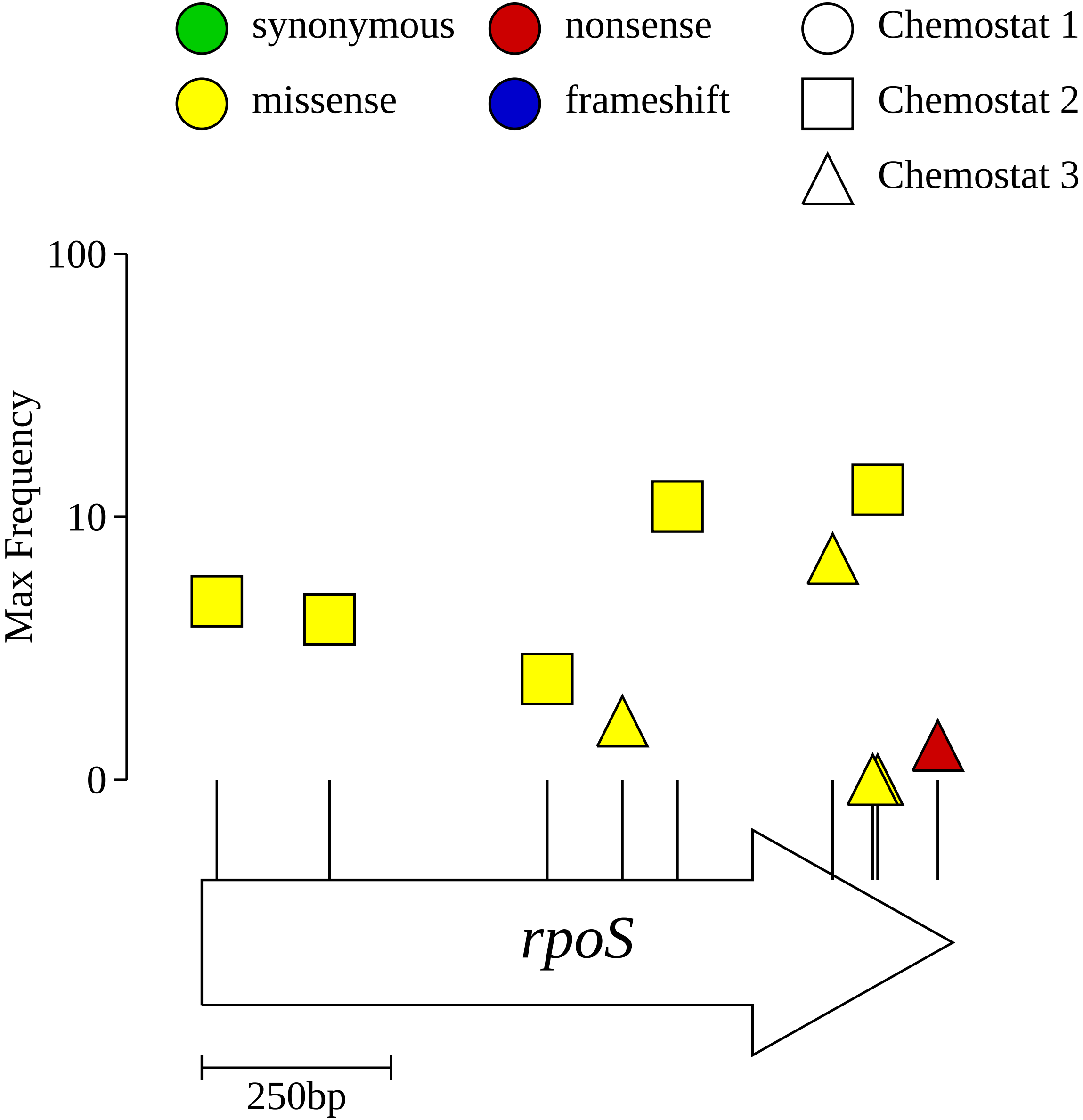

synonymous
nonsense
Chemostat 1
missense
frameshift
Chemostat 2
Chemostat 3
100
10
0
Max Frequency
rpoS
250bp

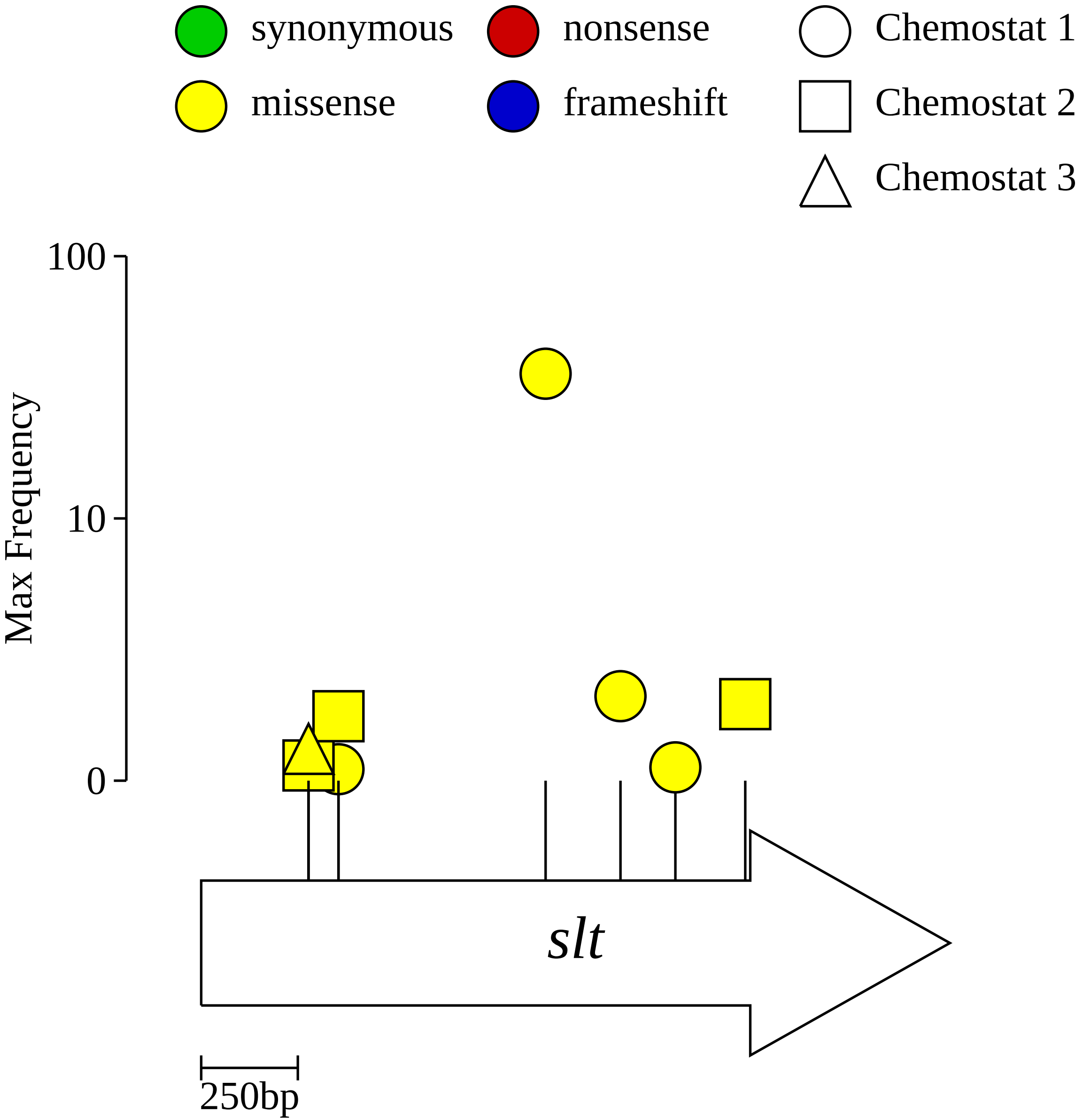
synonymous
nonsense
Chemostat 1
missense
frameshift
Chemostat 2
Chemostat 3
100
10
0
Max Frequency
slt
250bp

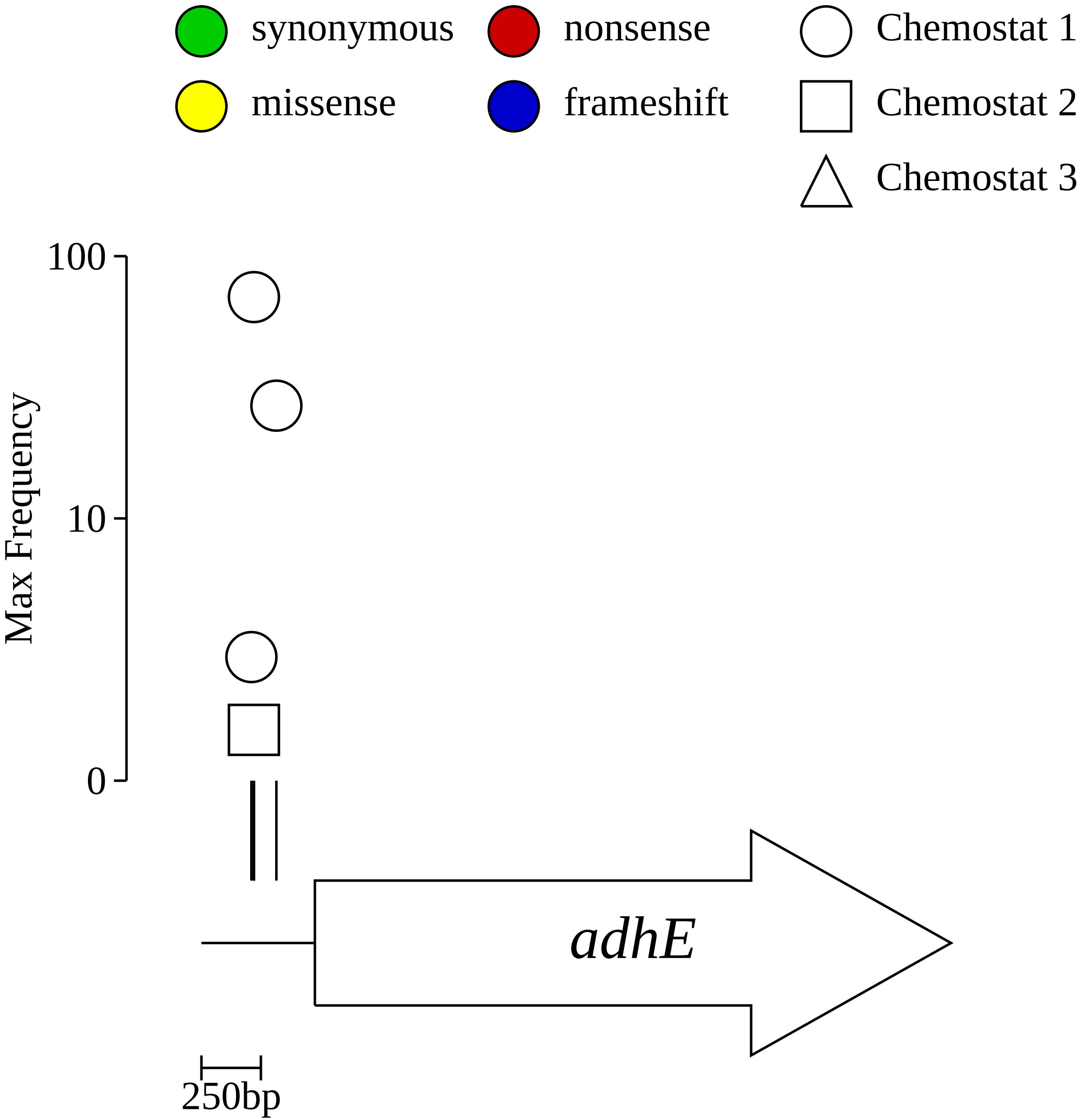

synonymous
missense
nonsense
frameshift
Chemostat 1
Chemostat 2
Chemostat 3
100
10
0
Max Frequency
adhE
250bp

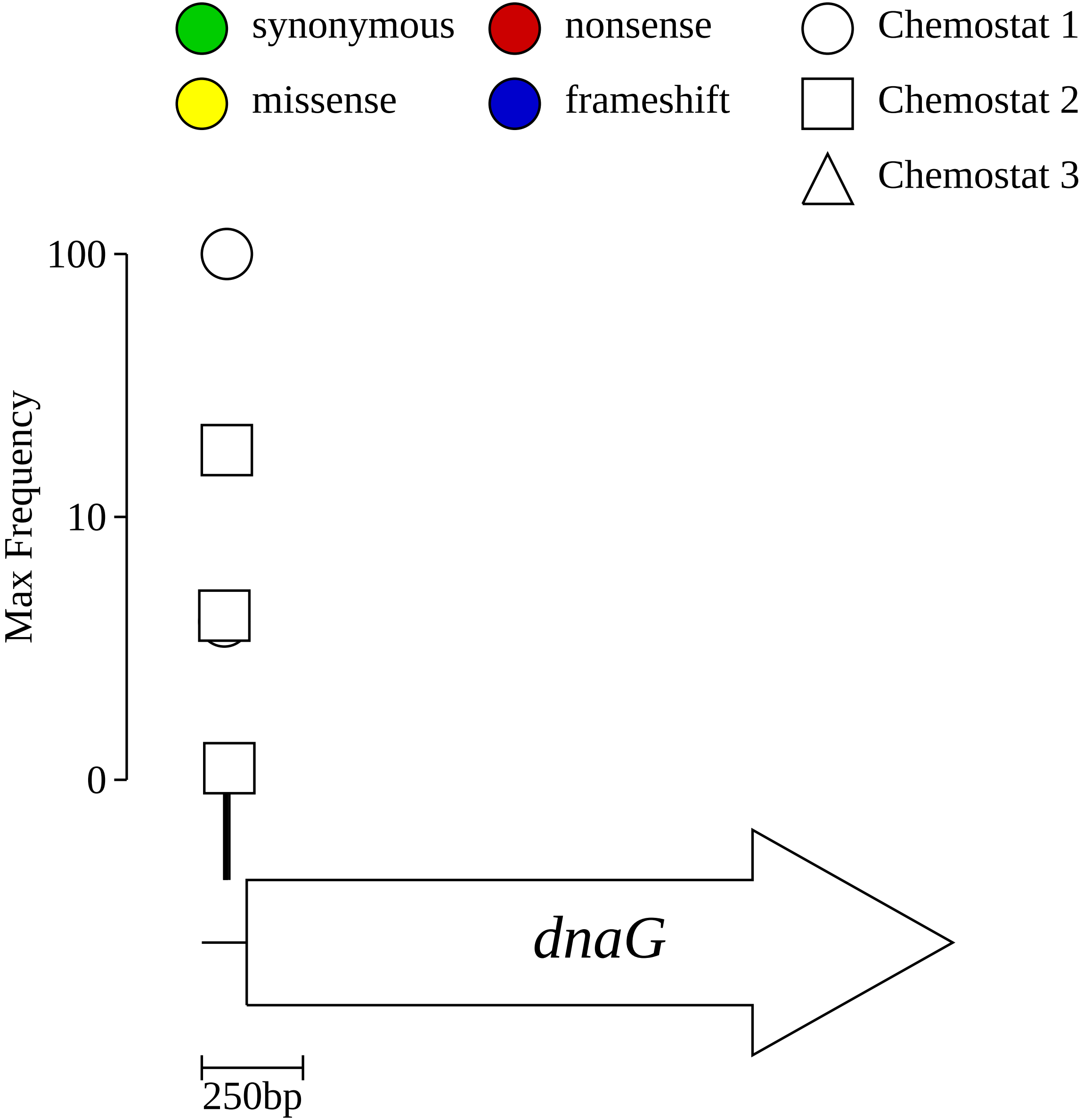
synonymous
missense
nonsense
frameshift
Chemostat 1
Chemostat 2
Chemostat 3
100
10
0
Max Frequency
dnaG
250bp

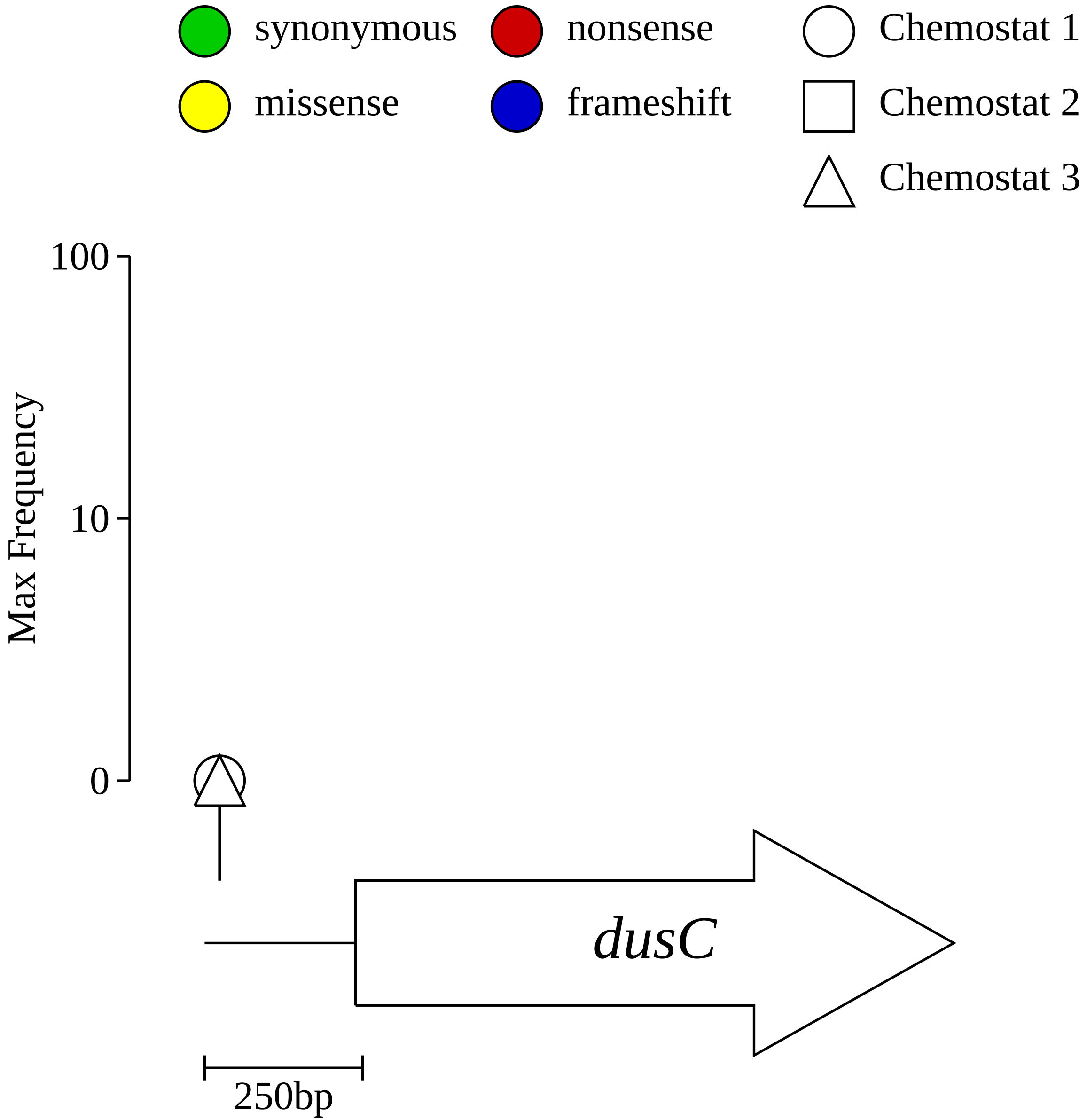
synonymous
missense
nonsense
frameshift
Chemostat 1
Chemostat 2
Chemostat 3
100
10
0
Max Frequency
dusC
250bp

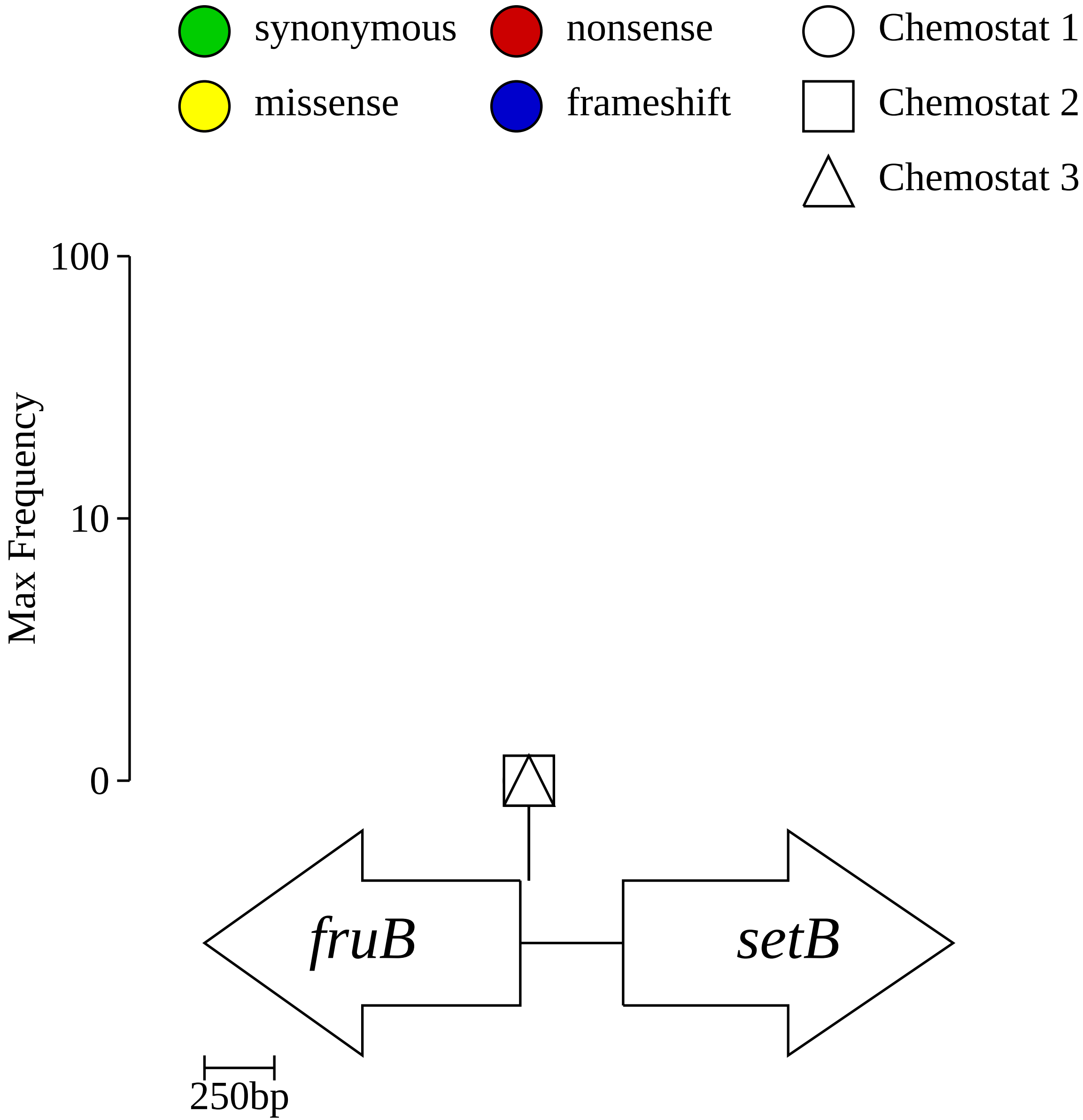
synonymous
missense
nonsense
frameshift
Chemostat 1
Chemostat 2
Chemostat 3
100
10
0
Max Frequency
fruB
setB
250bp

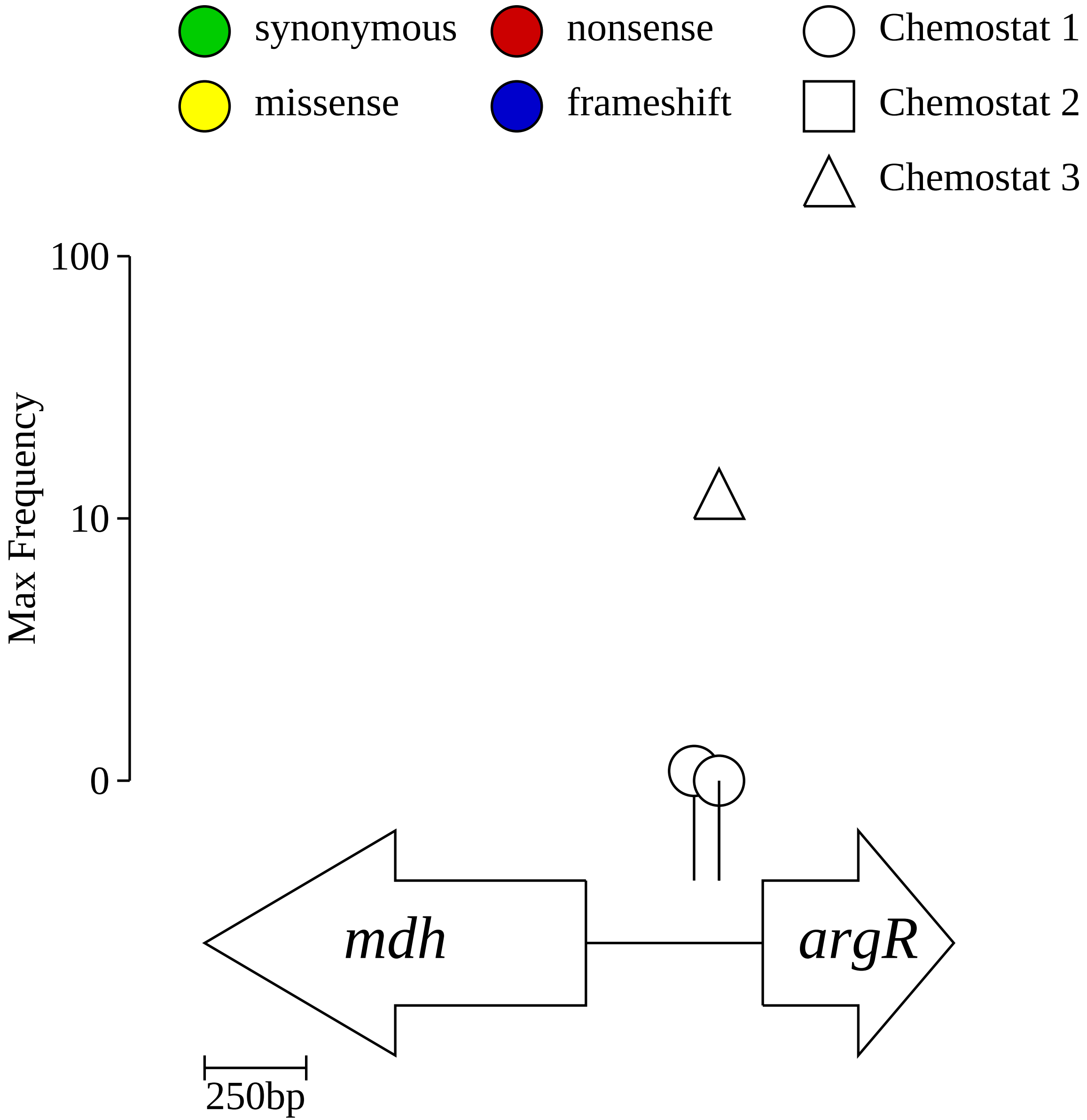
synonymous
nonsense
Chemostat 1
missense
frameshift
Chemostat 2
Chemostat 3
100
10
0
Max Frequency
mdh
argR
250bp

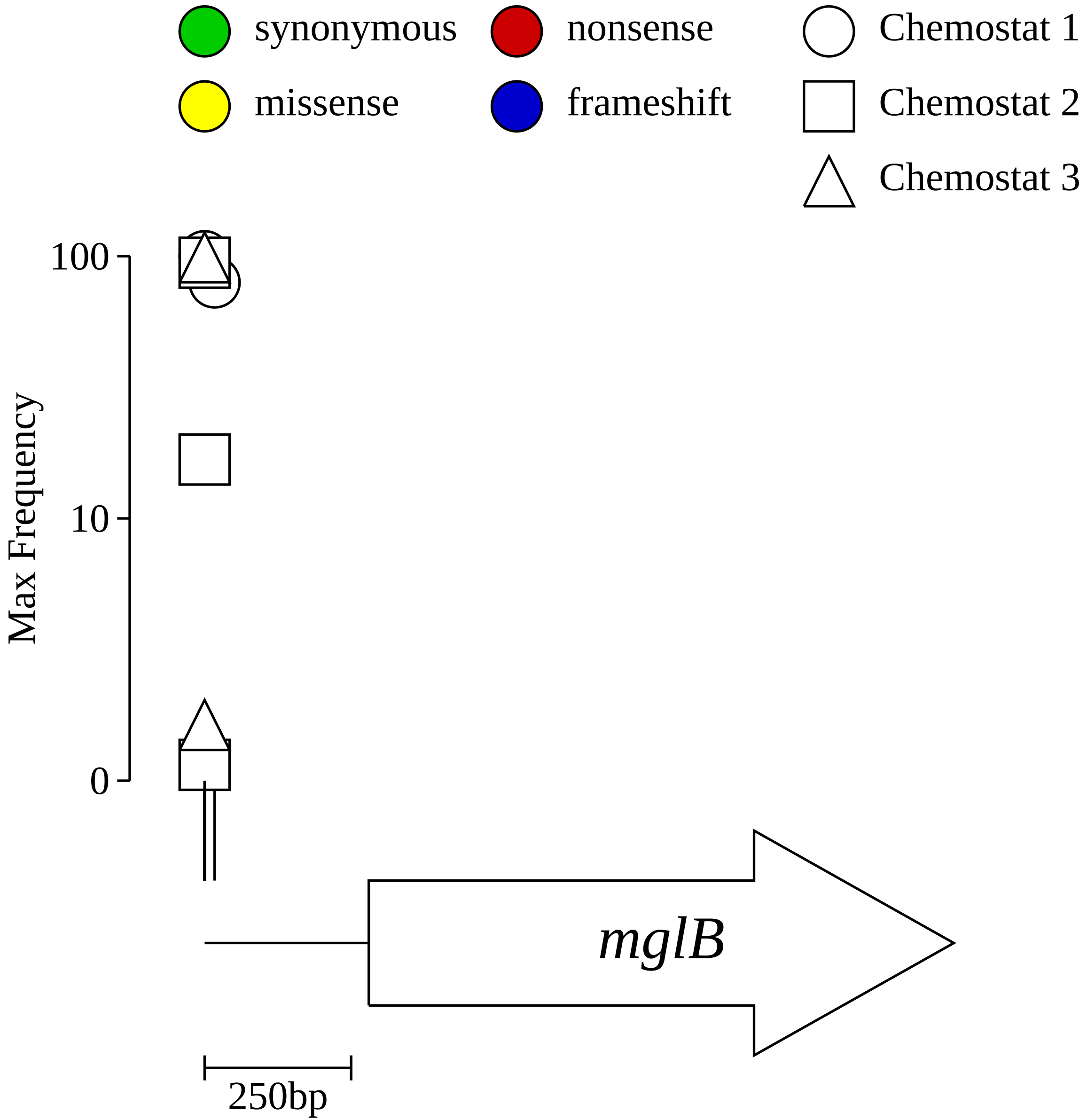
synonymous
nonsense
Chemostat 1
missense
frameshift
Chemostat 2
Chemostat 3
100
10
0
Max Frequency
mglB
250bp

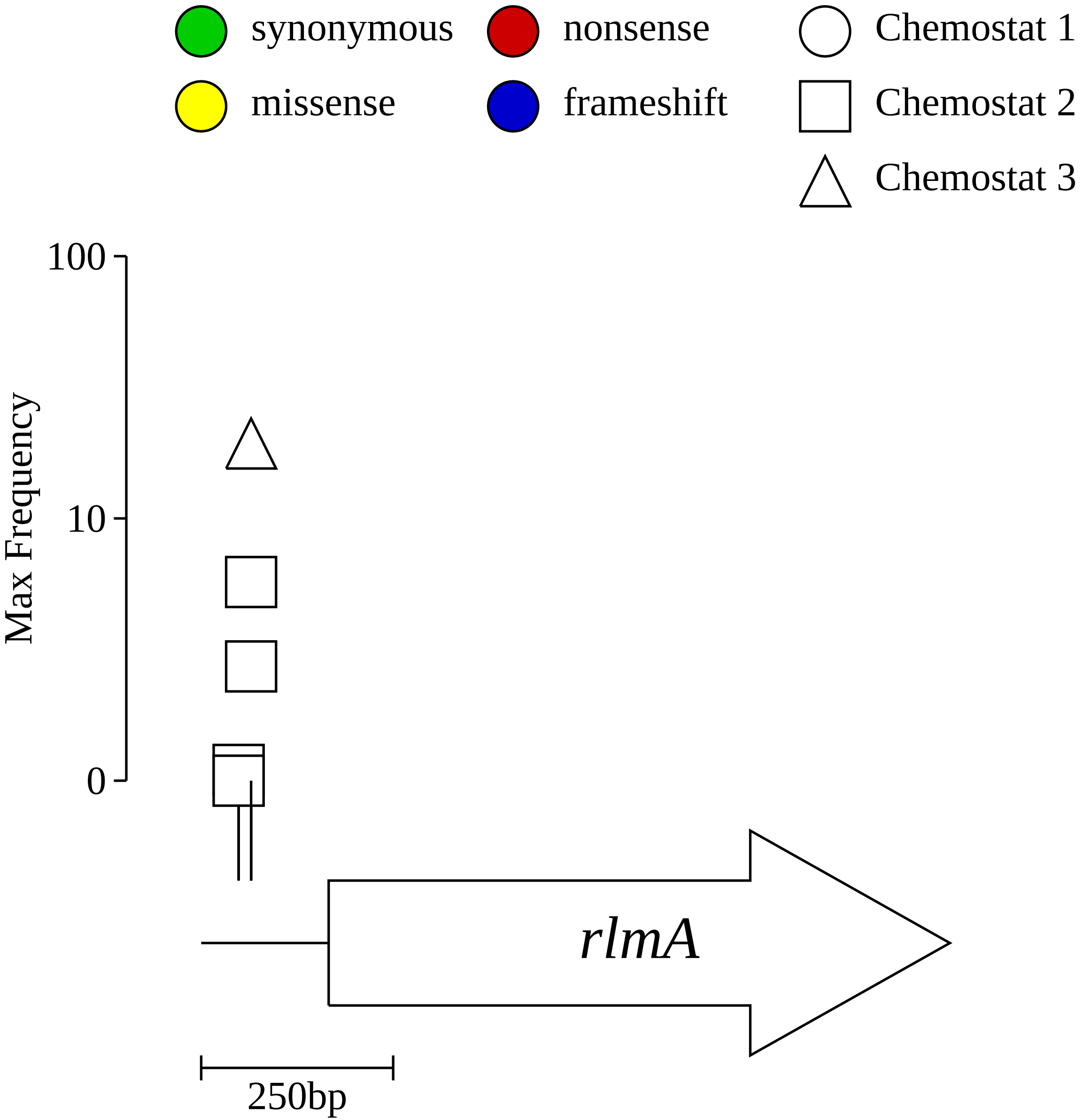
synonymous
nonsense
Chemostat 1
missense
frameshift
Chemostat 2
Chemostat 3
100
10
0
Max Frequency
rlmA
250bp

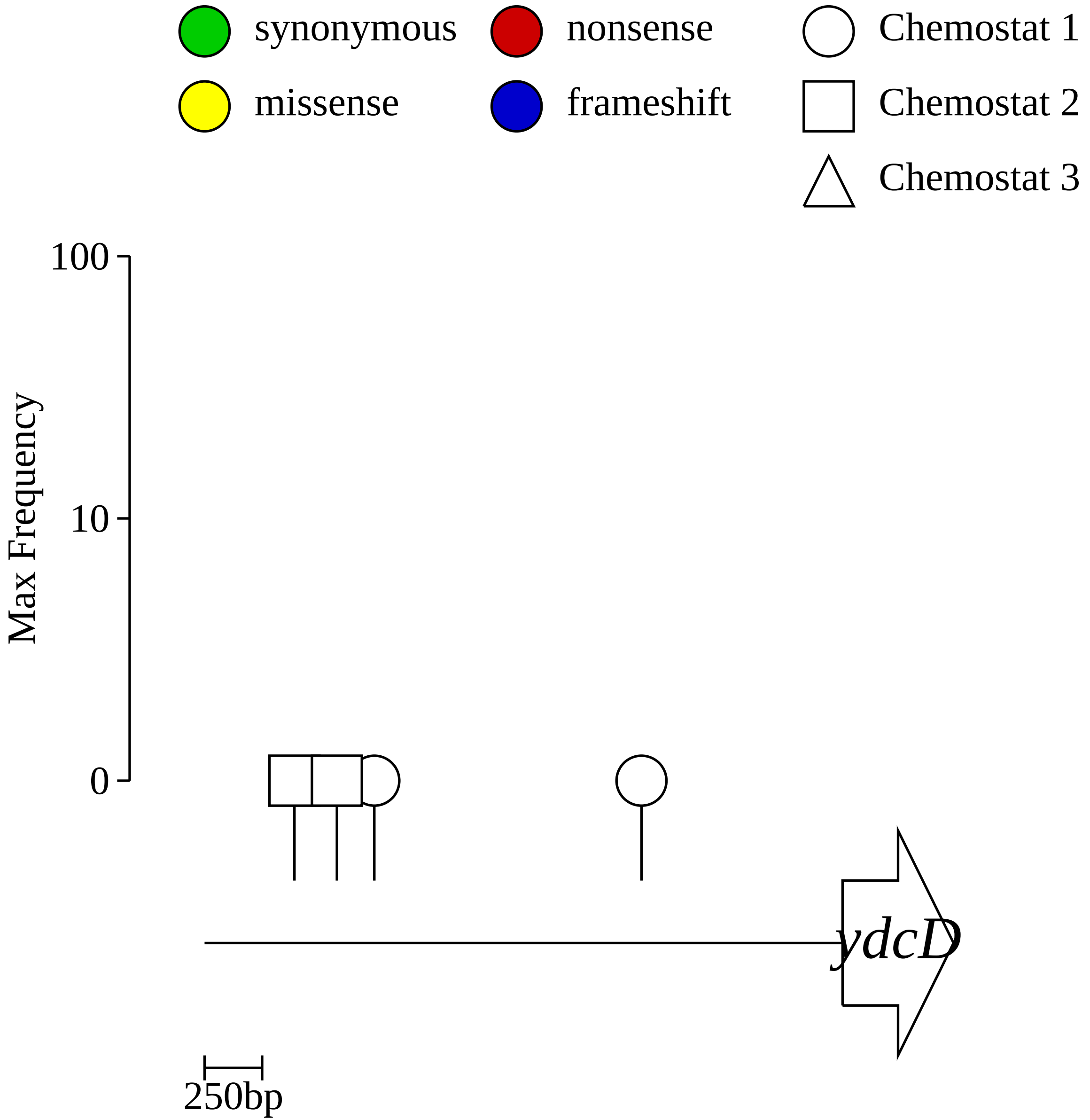
synonymous
nonsense
Chemostat 1
missense
frameshift
Chemostat 2
Chemostat 3
Max Frequency
100
10
0
ydcD
250bp

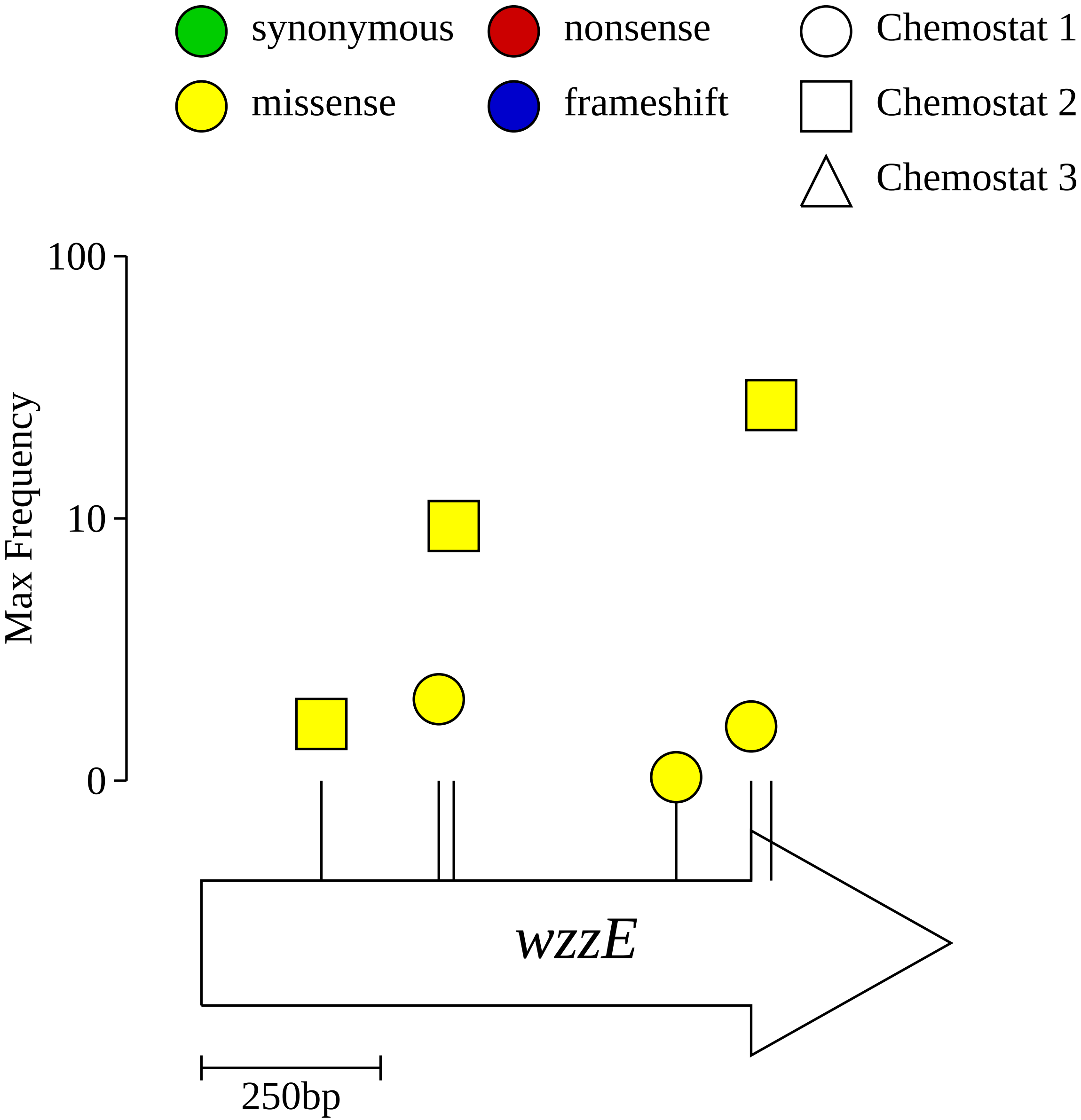
synonymous
nonsense
Chemostat 1
missense
frameshift
Chemostat 2
Chemostat 3
100
10
0
Max Frequency
wzzE
250bp

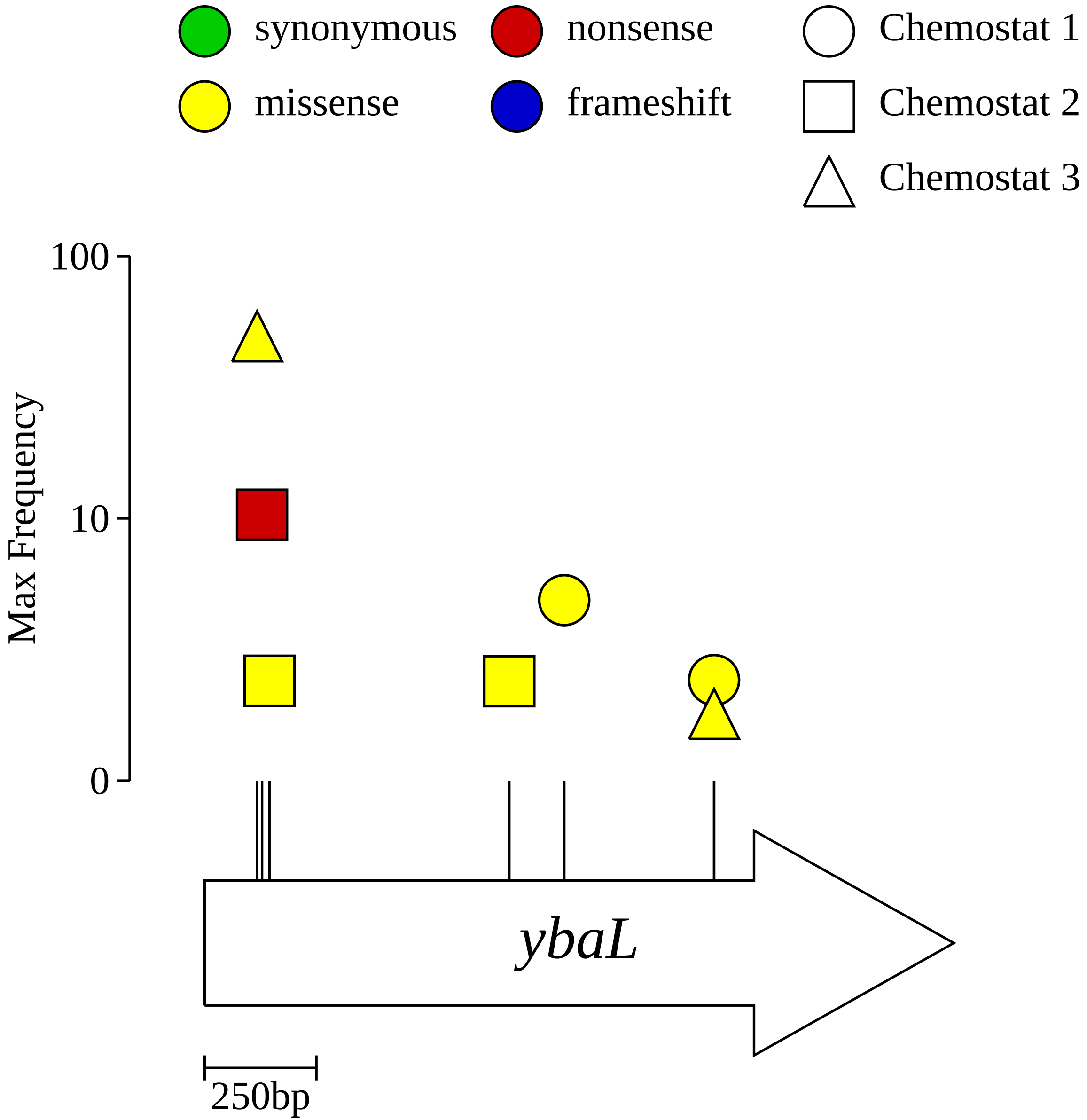
synonymous
nonsense
Chemostat 1
missense
frameshift
Chemostat 2
Chemostat 3
Max Frequency
100
10
0
ybaL
250bp

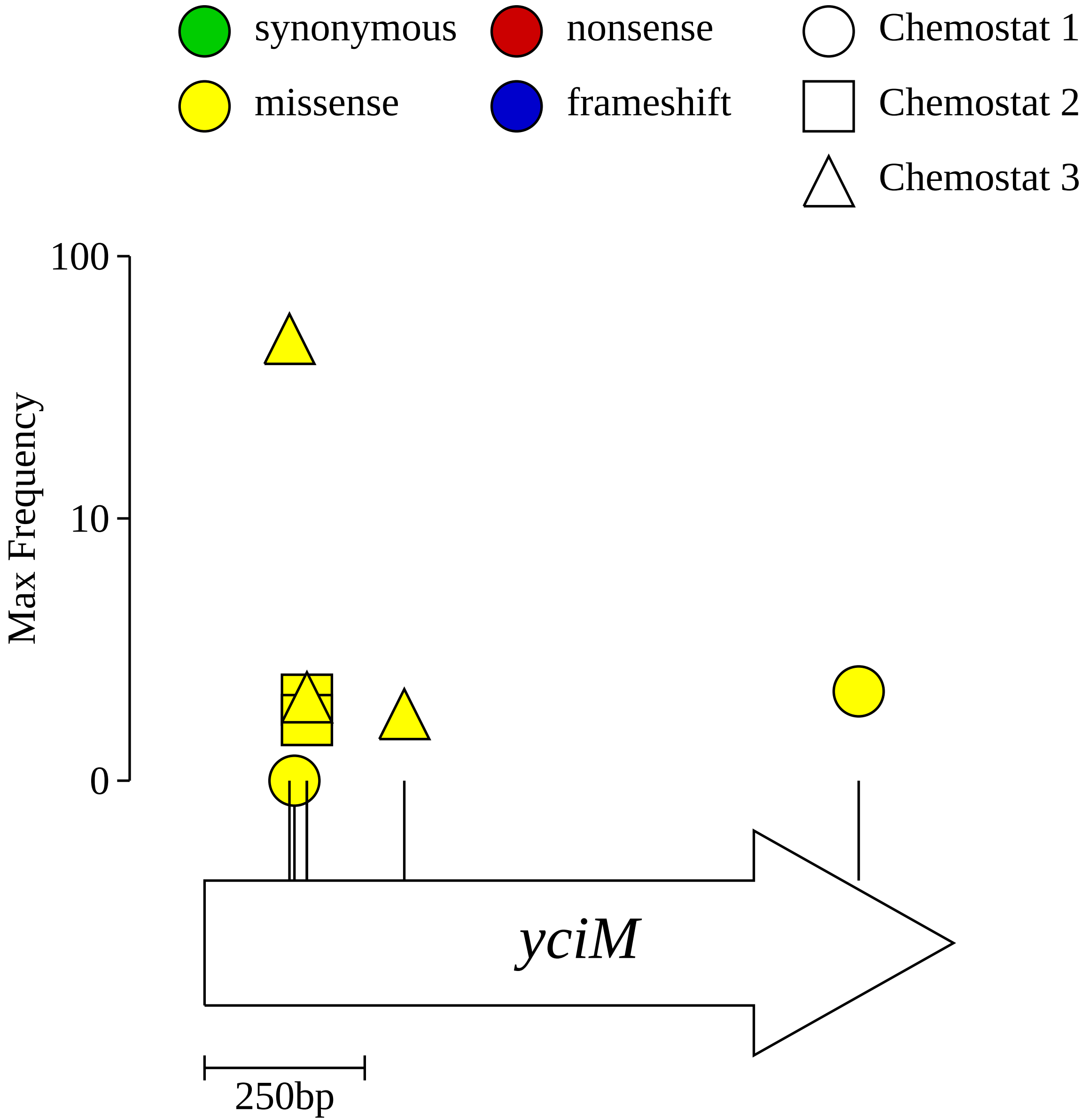

synonymous
nonsense
Chemostat 1
missense
frameshift
Chemostat 2
Chemostat 3
100
10
0
Max Frequency
yciM
250bp

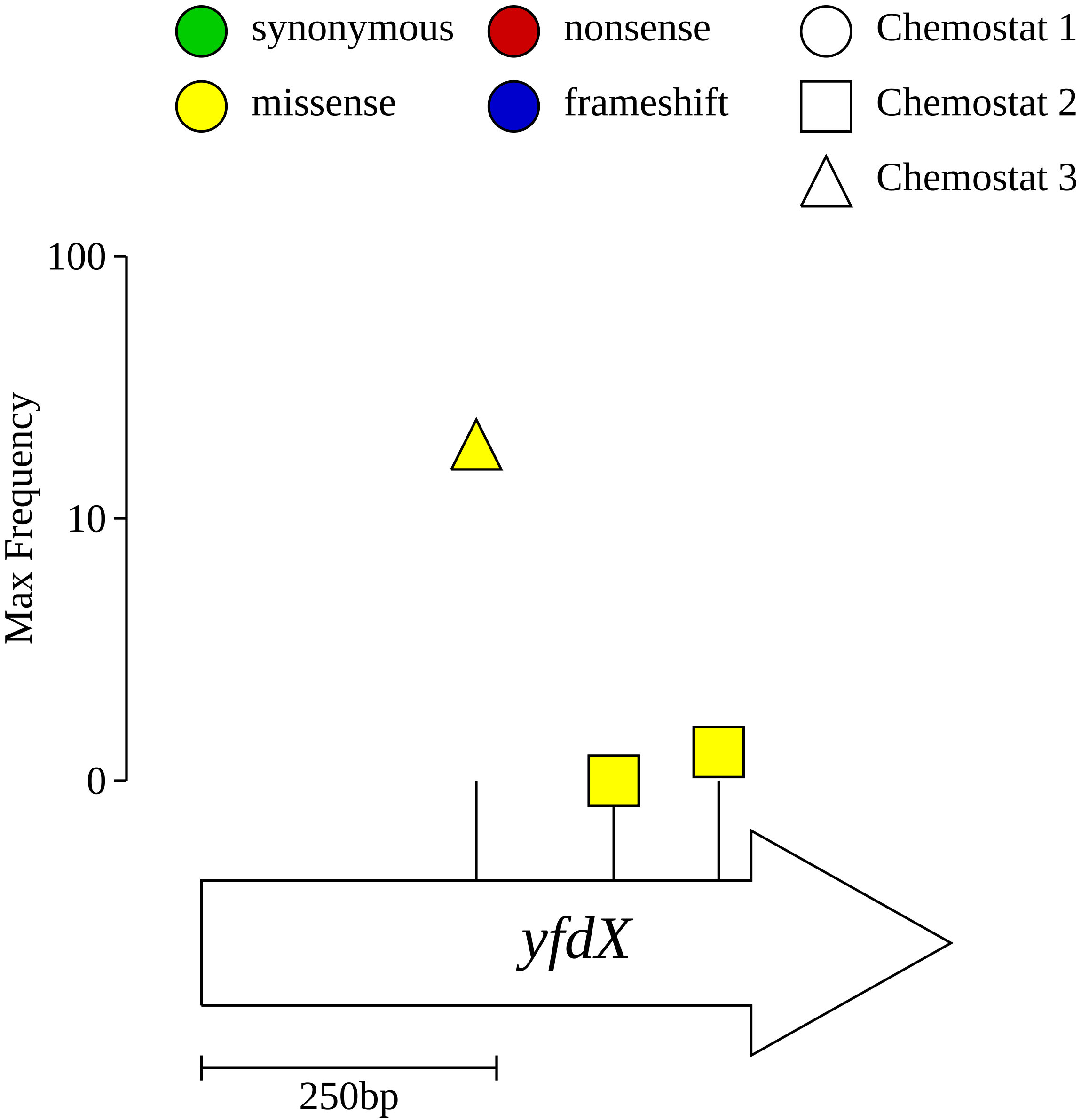
synonymous
nonsense
Chemostat 1
missense
frameshift
Chemostat 2
Chemostat 3
100
10
0
Max Frequency
yfdX
250bp

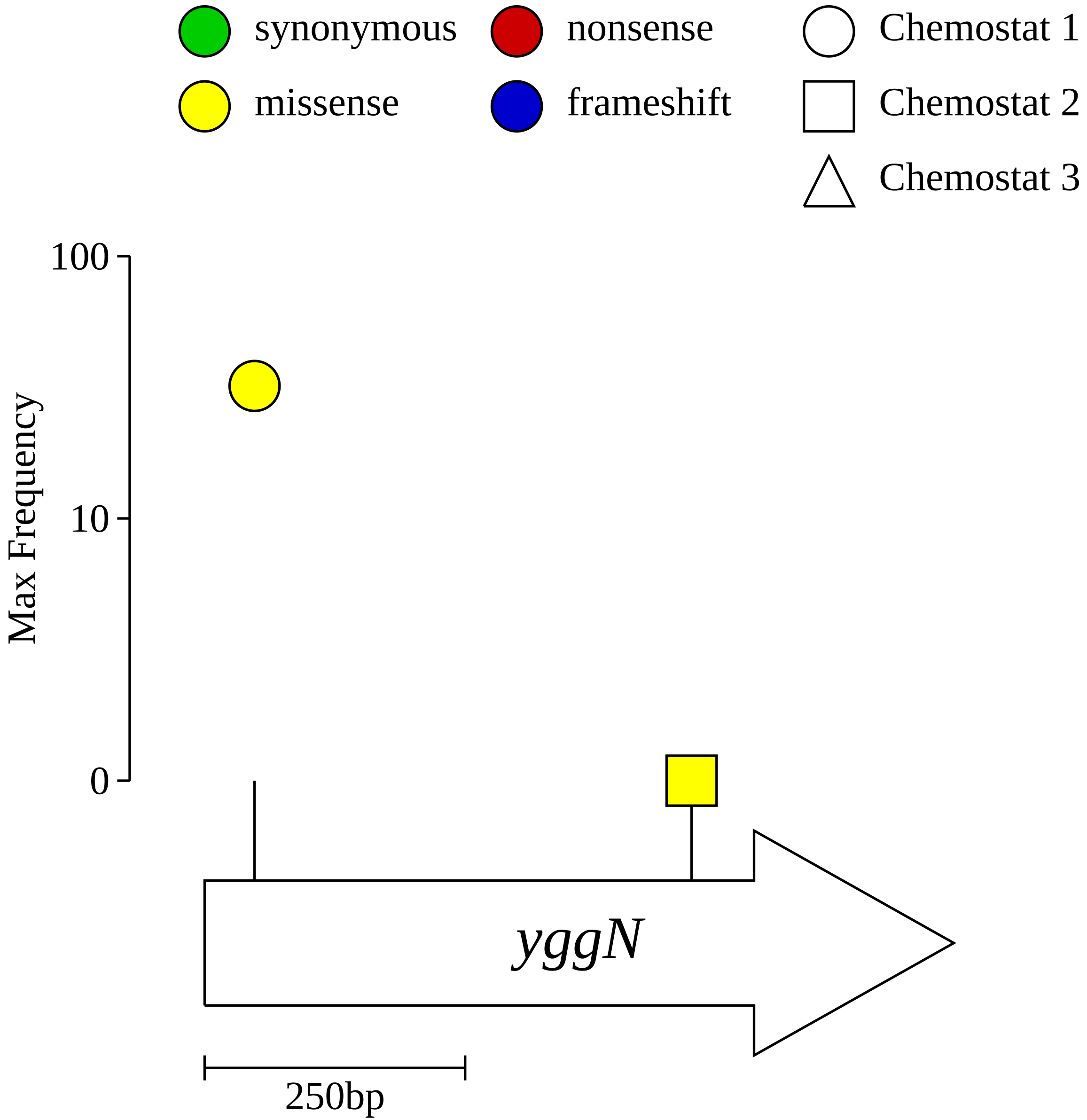
synonymous
nonsense
Chemostat 1
missense
frameshift
Chemostat 2
Chemostat 3
100
10
0
Max Frequency
yggN
250bp

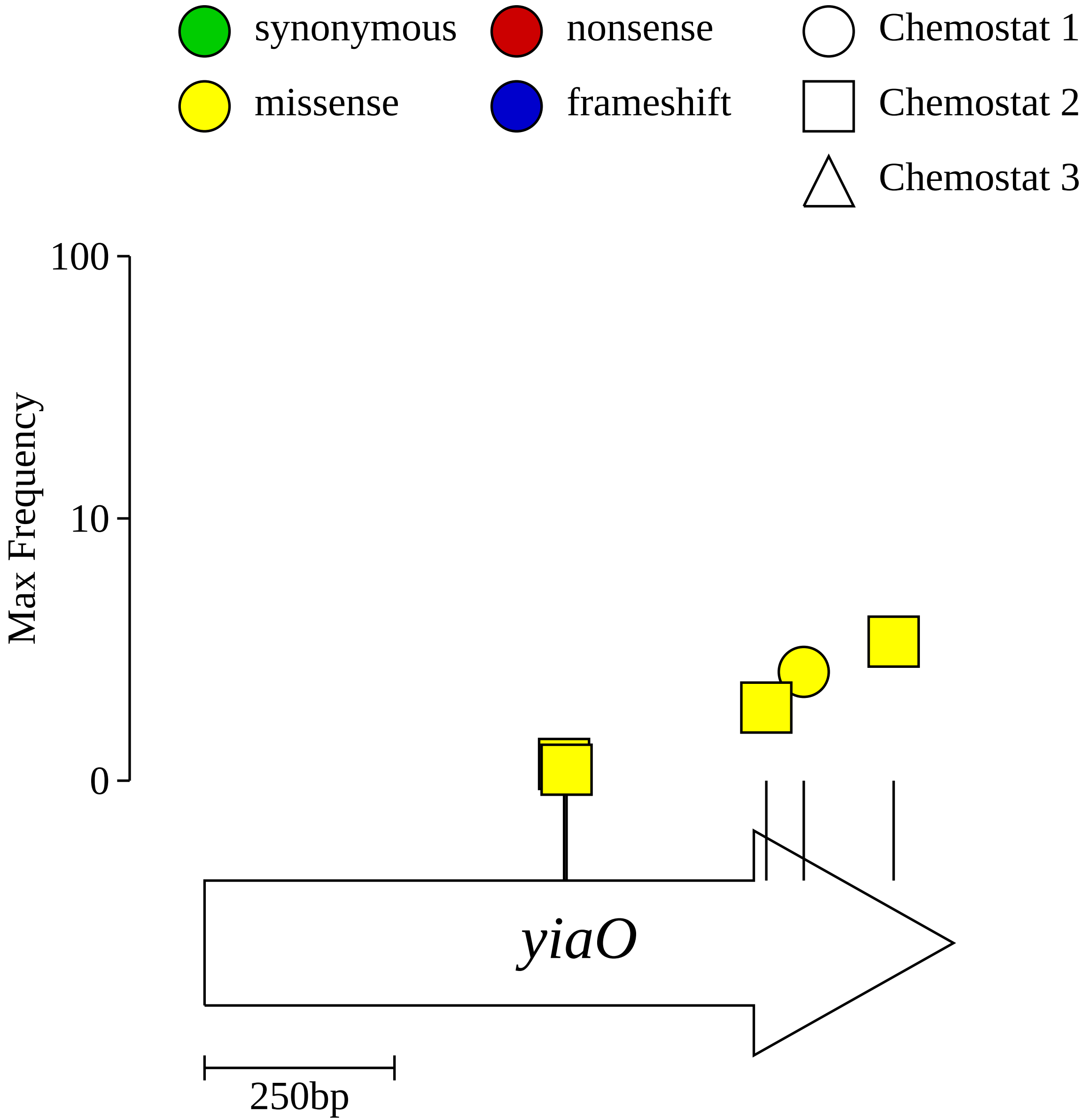
synonymous
nonsense
Chemostat 1
missense
frameshift
Chemostat 2
Chemostat 3
100
10
0
Max Frequency
yiaO
250bp

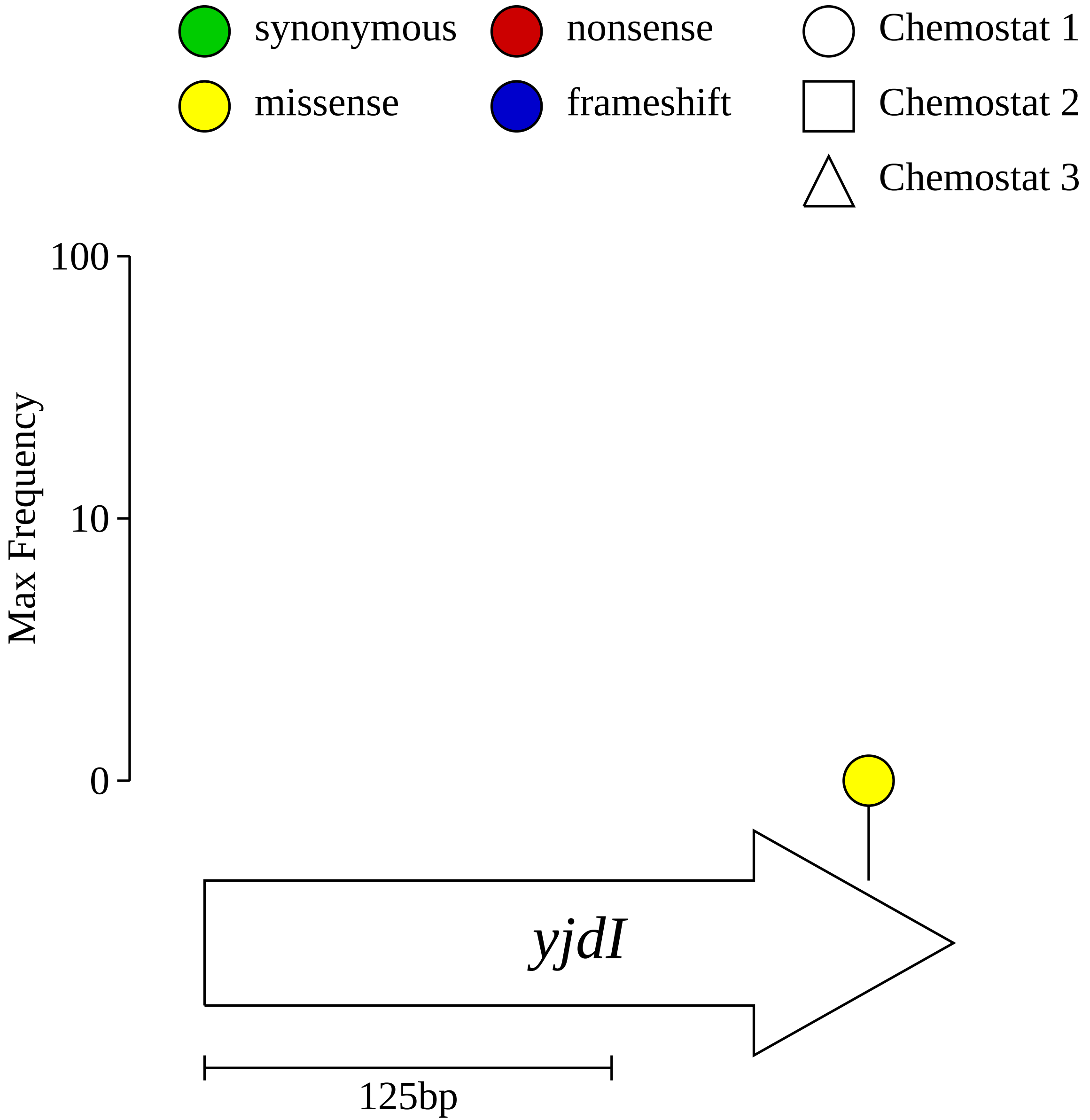

synonymous
nonsense
Chemostat 1
missense
frameshift
Chemostat 2
Chemostat 3
100
10
0
Max Frequency
yjdI
125bp

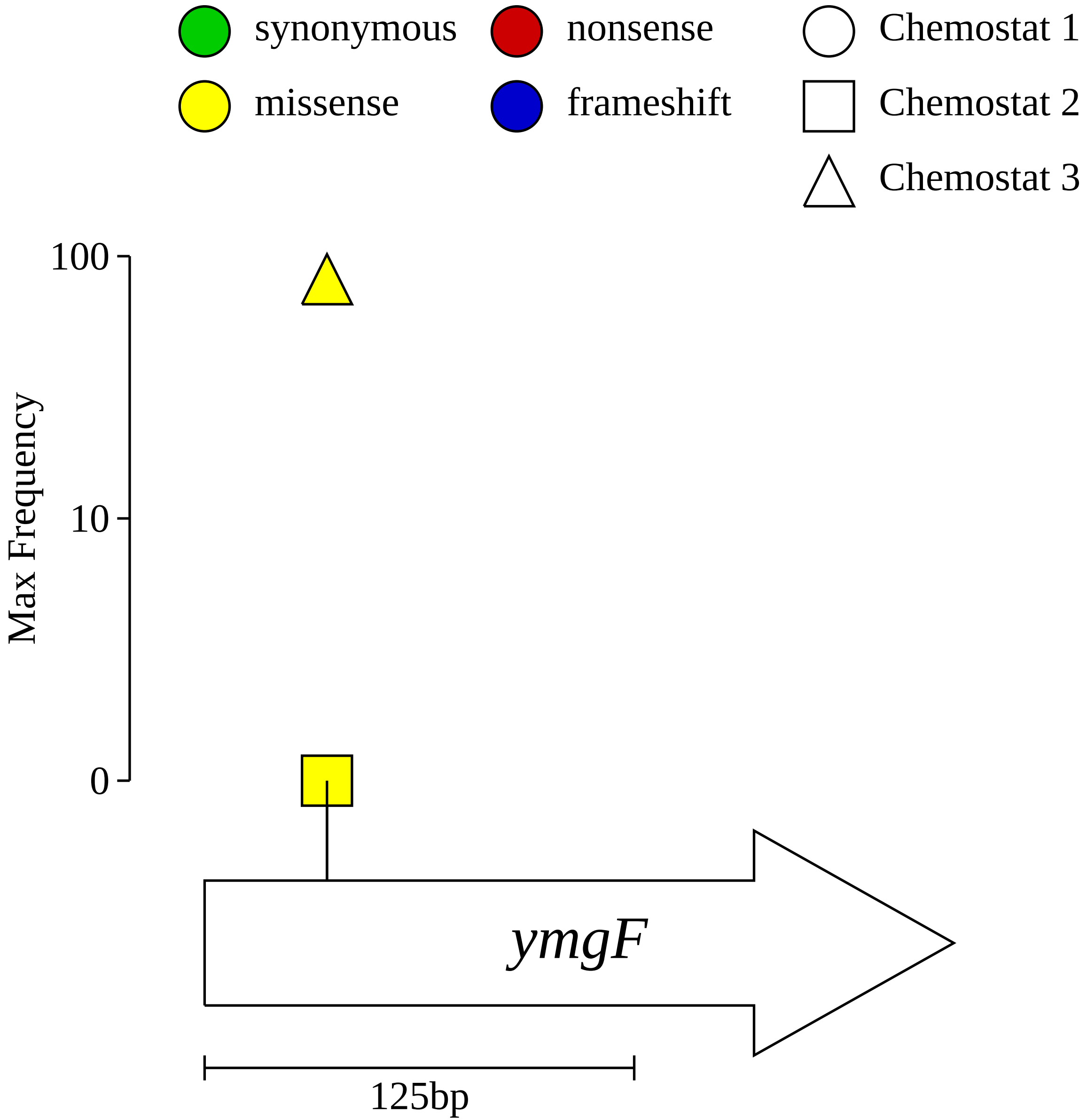

synonymous
nonsense
Chemostat 1
missense
frameshift
Chemostat 2
Chemostat 3
100
10
0
Max Frequency
ymgF
125bp

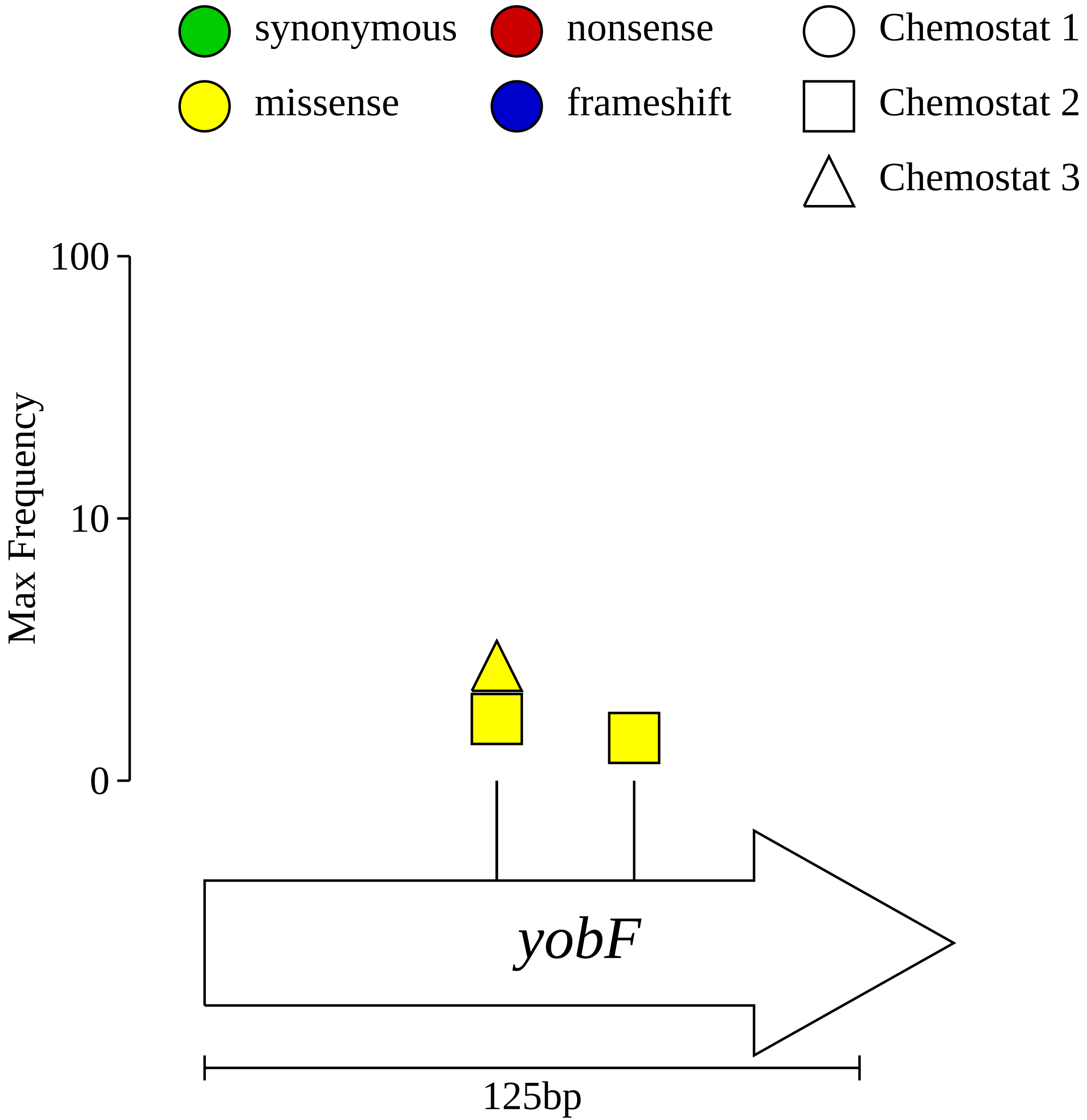
synonymous
nonsense
Chemostat 1
missense
frameshift
Chemostat 2
Chemostat 3
100
10
0
Max Frequency
yobF
125bp
